# Effectiveness of e-cigarettes as a stop smoking intervention in adults: a systematic review

**DOI:** 10.1186/s13643-024-02572-7

**Published:** 2024-06-29

**Authors:** Niyati Vyas, Alexandria Bennett, Candyce Hamel, Andrew Beck, Micere Thuku, Mona Hersi, Nicole Shaver, Becky Skidmore, Brian Hutton, Douglas Manuel, Matt Morrow, Smita Pakhale, Justin Presseau, Beverley J. Shea, Julian Little, David Moher, Adrienne Stevens

**Affiliations:** 1 School of Epidemiology and Public Health, Faculty of Medicine, University of Ottawa, Ottawa, ON Canada; 2 Knowledge Synthesis Group, Clinical Epidemiology Program, Ottawa Hospital Research Institute, Centre for Practice-Changing Research, Box 201, 501 Smyth Road, Ottawa, ON K1H 8L6 Canada; 3 Ottawa Hospital Research Institute, Ottawa, ON Canada; 4 The Ottawa Hospital, Ottawa, ON Canada; 5 Department of Otolaryngology, University of Ottawa, Ottawa, ON Canada; 6 Department of Family Medicine, University of Ottawa, Ottawa, ON Canada; 7 Patient Representative, Vancouver, BC Canada; 8 School of Psychology, University of Ottawa, Ottawa, ON Canada

**Keywords:** Electronic cigarettes, E-cigarettes, Smoking cessation, Systematic review smoking intervention

## Abstract

**Background:**

This systematic review aims to identify the benefits and harms of electronic cigarettes (e-cigarettes) as a smoking cessation aid in adults (aged ≥ 18 years) and to inform the development of the Canadian Task Force on Preventive Health Care’s (CTFPHC) clinical practice guidelines on e-cigarettes.

**Methods:**

We searched Ovid MEDLINE®, Ovid MEDLINE® Epub Ahead of Print, In-Process & Other Non-Indexed Citations, PsycINFO, Embase Classic + Embase, and the Cochrane Library on Wiley. Searches were conducted from January 2016 to July 2019 and updated on 24 September 2020 and 25 January 2024. Two reviewers independently performed title-abstract and full-text screening according to the pre-determined inclusion criteria. Data extraction, quality assessments, and the application of Grading of Recommendations Assessment, Development and Evaluation (GRADE) were performed by one independent reviewer and verified by another.

**Results:**

We identified 18 studies on 17 randomized controlled trials that compared e-cigarettes with nicotine to e-cigarettes without nicotine and e-cigarettes (with or without nicotine) to other interventions (i.e., no intervention, waitlist, standard/usual care, quit advice, or behavioral support). Considering the benefits of e-cigarettes in terms of smoking abstinence and smoking frequency reduction, 14 studies showed small or moderate benefits of e-cigarettes with or without nicotine compared to other interventions; although, with low, very low or moderate evidence certainty. With a focus on e-cigarettes with nicotine specifically, 12 studies showed benefits in terms of smoking abstinence when compared with usual care or non-nicotine e-cigarettes. In terms of harms following nicotine or non-nicotine e-cigarette use, 15 studies reported mild adverse events with little to no difference between groups and low to very low evidence certainty.

**Conclusion:**

The evidence synthesis on the e-cigarette’s effectiveness shows data surrounding benefits having low to moderate evidence certainty for some comparisons and very low certainty for others, indicating that e-cigarettes may or probably increase smoking cessation, whereas, for harms, there is low to very low evidence certainty. Since the duration for outcome measurement varied among different studies, it may not be long-term enough for Adverse Events (AEs) to emerge, and there is a need for more research to understand the long-term benefits and potential harms of e-cigarettes.

**Systematic review registration:**

PROSPERO CRD42018099692

**Supplementary Information:**

The online version contains supplementary material available at 10.1186/s13643-024-02572-7.

## Background

### Prevalence and burden of tobacco smoking

Tobacco use affects millions of people each year and over 8 million people died from tobacco-related diseases in 2019 [1]. The World Health Organization (WHO) member states adopted the WHO Framework Convention on Tobacco Control in 2003, which outlines different evidence-based actions that all member states should consider [1, 2]. The WHO global report (2019) on trends in tobacco use from 165 countries showed that in 2020, the global prevalence of current smokers declined to 22.3% from 32.7% in 2000 among those aged 15 years and older, with an expected decline to around 20.4% by 2025 [1]. In Canada, there has been an overall reduction in the prevalence of current smokers over the past years. The Canadian Tobacco and Nicotine Survey (CTNS) showed that in 2022 the cigarette smoking prevalence among adults aged 25 years and older was 11.7% [95% CI 10.8% to 12.7%], unchanged from 2021, with a higher prevalence among adult men than women (13.8% versus 9.8%) [3].

Smoking continues to contribute as one of the leading causes of preventable deaths. Data from the Institute for Health Metrics and Evaluation (IHME), showed that tobacco use was ranked first of the top ten risk factors driving the most death and disability combined in Canada [4]. The 2019 global health metrics showed that tobacco remained the third leading risk factor for global attributable disability-adjusted life-years (DALYs) despite the more than 1% per year decline in age-standardized tobacco use between 2010 and 2019 [5].

Smoking cessation has been shown to improve general, mental, and physical health [6–9]. A Canadian study found that men who had quit for 20 years had the same quality of life as those who had never smoked; this observation was even more beneficial for females, who only had to quit for 10 years [10]. Smoking cessation reduces over 90% of the mortality risk associated with continuous smoking if stopped before age 40 [11, 12]. More than two-thirds (68.4%) of smokers who intended to quit attempted to use some form of cessation assistance, one-third (31.8%) used nicotine replacement therapy (NRT), and 26.5% reported using electronic cigarettes (e-cigarettes) as a cessation aid [3, 13].

E-cigarettes are battery-powered devices that heat a solution to deliver an aerosolized vapor with or without nicotine [14, 15]. They are popular amongst non-smokers, those who wish to quit cigarette smoking, youths, and young adults [16]. E-cigarettes may act as a smoking cessation aid by satisfying the sensory and behavioral cues of holding and smoking a cigarette without providing the combustible harms associated with cigarettes, such as formaldehyde, acrolein, or acetaldehyde [16–18]. There are varied types of e-cigarettes with varying brands and models available. This variation exists in terms of device type and the composition of e-liquids (i.e., nicotine content, flavors, and other components) [19, 20]. As per the CTNS survey, those aged 15 to 24 years reported stress and curiosity as the reasons behind vaping, whereas for those aged 25 years old, the most common reasons were to help them quit smoking and cope with smoking relapse [3]. A 2022 Cochrane review on e-cigarettes for smoking cessation suggested that nicotine e-cigarettes could probably help more people quit smoking than using nicotine replacement therapy (risk ratio (RR) 1.63, 95% confidence interval (CI) 1.30 to 2.04; *I*^2^ = 10%; 6 studies, 2378 participants) with high level of evidence certainty. Additionally, there was a moderate level of evidence certainty that smoking quit rates were higher in nicotine e-cigarettes group than nicotine-free-e-cigarettes (RR 1.94, 95% CI 1.21 to 3.13; *I*^2^ = 0%; 5 studies, 1447 participants) and very low level of evidence certainty, although with higher quit rates in those randomized to nicotine e-cigarette compared to behavioral support only/no support group (RR = 2.66, 95% CI 1.52 to 4.65; *I*^2^ = 0%; 7 studies, 3126 participants) [21]. Although there is no official approval of vaping products in Canada under the Food and Drugs Act (FDA) as a smoking cessation aid, e-cigarettes may reduce health risks for smokers who would otherwise not quit on their own or while using counseling or approved pharmacotherapies [22]. Evidence on the use of e-cigarettes and their health risks is inconclusive, which calls for a vigorous investigation of their effects on health outcomes [14].

## Current guideline recommendations

### Guidelines from international organizations

E-cigarettes have been addressed by four international guideline organizations. The NICE guidelines recommend advising on use by health care professionals and giving clear information about nicotine-containing e-cigarettes to adults who smoke and are interested in using them to stop, including that they are not licensed medicines, and that there is not enough evidence to know whether there are long-term harms from e-cigarette use [23]*.* The New Zealand Ministry of Health guideline also recommends that vaping products with nicotine can be used for smoking cessation, but indicates that the long-term effects of e-cigarette use are unknown [24]*.* The United States Preventive Services Task Force (USPSTF) judged that the evidence on e-cigarettes is insufficient and recommends directing patients to interventions with proven effectiveness and safety [25]*.* The Royal Australian College of General Practitioners advises that nicotine-containing e-cigarettes may be considered for people who were unsuccessful with first-line therapies and have brought up e-cigarettes with their provider and that patients should be informed of the risks and conditions of use (i.e., avoiding dual use, only short-term use) [26, 27]*.*

Considering the growing interest in using e-cigarettes to quit conventional cigarette smoking, a systematic review was developed with a need to address guidance on whether e-cigarettes should be recommended as one of the smoking cessation strategies relevant to the Canadian context.

## Objective

Our objective was to review the evidence regarding the benefits and harms of e-cigarettes as a smoking cessation intervention among adults and to inform the development of the Canadian Task Force on Preventive Health Care’s (CTFPHC) clinical practice guidelines on e-cigarettes. The following key question will be answered: What are the benefits and harms of electronic cigarettes with or without nicotine for tobacco use abstinence in adults compared to usual care?

## Methods

We conducted an evidence review that occurred in two stages. The aim of stage 1 was to evaluate the benefits and harms of various smoking cessation interventions for adults and to identify a candidate review on e-cigarettes to update for stage 2, which is the subject of this paper. Eighteen systematic reviews, identified from stage 1, were first assessed for representativeness (e.g., population of interest, how recent the search was performed) [28–44]. Four reviews [29, 30, 36, 43] were further evaluated with AMSTAR 2 and discussed [45]. Due to poor reporting, three reviews [30, 36, 43] were removed from consideration. Hartmann-Boyce 2016 [29] was selected as the candidate review because it provided a complete list of excluded studies, included clinical trials registry protocols, and provided support for the risk of bias (RoB) judgments. The results of stage 1 are reported elsewhere.

Our evidence review was developed, conducted, and prepared according to the Preferred Reporting Items for Systematic Reviews and Meta-Analyses (PRISMA) statement (Additional file 1: Appendix 1) [46]. For additional quality control, we used AMSTAR 2 to guide the conduct of this review [45]. Details on how the topic was developed, eligibility criteria, and how outcomes were determined can be found in the protocol, which is published and registered with PROSPERO (https://www.crd.york.ac.uk/PROSPERO/) (CRD42018099692) [47].

### Eligibility criteria

Randomized controlled trials were selected for inclusion to evaluate the benefits of e-cigarettes, as specified in Additional file 3: Appendix 3. To explore harms associated with e-cigarettes, randomized and non-randomized trials, comparative observational studies (i.e., prospective, and retrospective cohort, case–control) were selected for inclusion. Briefly, the systematic review focuses on adults (≥ 18 years) who are current smokers in whom various interventions are compared with inactive, minimally active [i.e., non-nicotine-containing e-cigarettes (e.g., placebo e-cigarettes)] or usual care control. To determine the eligibility of interventions for a given analysis, we included interventions like nicotine or non-nicotine-containing e-cigarettes alone or combined with other interventions (i.e., behavioral, or pharmacological). Alternatively, we excluded studies if they explicitly examined short-term use of nicotine or non-nicotine-containing e-cigarettes (< 1 week). For the smoking cessation outcomes, we included tobacco abstinence, smoking reduction data, and other outcomes as mentioned in Additional file 3: Appendix 3. For the smoking reduction, we included outcomes if reported a minimum of 6 months from the quit date or intervention initiation (if the quit date is not specified).

### Literature sources and strategy

The search strategy was developed and tested through an iterative process by an experienced medical information specialist in consultation with the review team. We searched Ovid MEDLINE®, Ovid MEDLINE® Epub Ahead of Print, In-Process & Other Non-Indexed Citations, PsycINFO, Embase Classic + Embase, and the Cochrane Library on Wiley. As this was an update from the 2016 Hartmann-Boyce systematic review [29] whose search strategy was run in January 2016, databases were searched from January 2016 to July 3, 2019. The search strategy was peer-reviewed using the PRESS 2015 guideline [48]. The electronic search strategies were updated on 24 September 2020 and 25 January 2024. The final search strategy is provided in Additional file 2: Appendix 2.

The search for grey literature was the same as what was conducted for stage 1. We also scanned the bibliographies of relevant reviews for any studies not identified in our database search. Grey literature searching was restricted to English and French language documents and was limited to what could be completed within 1 week by one reviewer.

### Study selection

Duplicates were identified and removed using a reference manager (Reference Manager 12, Thomson Reuters, New York, USA) [49]. Title, abstract, and full-text screening were conducted using an online systematic review managing software (DistillerSR, Evidence Partners, Ottawa, Canada) [50]. Two reviewers independently screened the title and abstracts of citations using the liberal accelerated method (i.e., a second reviewer verifies records excluded by a first reviewer) [51]. References were sorted in random order to ensure that each reviewer could not determine whether a given reference was excluded by another reviewer. The full text of potentially relevant citations was retrieved, and two reviewers independently assessed the article for relevancy against the a priori-defined eligibility criteria. Conflicts were resolved by consensus or by consulting with a third team member. The reasons for exclusion at full-text screening were documented. Where study eligibility was unclear, authors were contacted by email twice over 2 weeks for additional information. Both screening forms were piloted by reviewers prior to the commencement of screening, with adjustments made, as needed, to maximize efficiency. If necessary, articles were ordered via interlibrary loan. Only those received within 30 days were included. Exclusions due to the unavailability of articles were noted in the list of excluded studies (Additional file 4: Appendix 4).

### Data extraction

One reviewer extracted data from all included studies, with a second reviewer verifying all extracted data. Conflicts were resolved through discussion. We collected both self-reported and biochemically validated tobacco abstinence and reduction results. Data for abstinence, reduction, and quality of life were collected at 6 months or later, whereas information on adverse events (AEs) and possible adverse outcomes were collected at all time points reported. Where needed, we converted data (e.g., standard error to standard deviation (SD), median (interquartile range [IQR]) to mean (SD)) to facilitate consistent presentation of results across studies. Authors were contacted by email twice over 2 weeks if any information was missing or was unclear.

### Risk of bias

The RoB of randomized controlled trials was assessed by one reviewer using the Cochrane RoB tool version 1 [52]. For assessing the quality of cohort studies, a modified version of the Scottish Intercollegiate Guidelines Network critical appraisal tool was used [53]. We considered industry funding under the ‘other sources of bias’ domain of the tool. Verification was performed by a second reviewer. Any disagreements were resolved by consensus. Some domains are outcome-specific (e.g., blinding of participants) and were assessed at the outcome level. Overall RoB for the body of evidence was evaluated according to the importance of domains, the likely direction of bias, and the likely magnitude of bias [52]. The Agency for Healthcare Research and Quality guidance was followed for evaluating RoB for outcome and analysis reporting bias [54].

### Analysis

Study characteristics were summarized narratively and presented in summary tables. Where possible, relative and absolute effects with 95% confidence intervals were calculated and presented in a GRADE summary of findings and evidence profile tables. RR and risk differences (RD) were used to report effects for dichotomous data. For continuous outcomes, MD (i.e., difference in means) effect measure was used. Due to clinical and methodological heterogeneity (i.e., different types of e-cigarettes, their doses and combinations, duration of interventions, varied outcome reporting) meta-analysis, subgroup analysis, sensitivity analysis, and small study effects were not performed. The Cochrane Review Manager software version 5.3 [55] was used to create forest plots.

### Certainty assessment

For all critical and important outcomes as defined in Additional file 3: Appendix 3, we used the GRADE framework to assess the certainty of the evidence. The GRADE assessments were performed by one person and verified by a second, with any remaining disagreements resolved via consensus [56, 57]. The RoB assessment, one reviewer performed the RoB assessment while another reviewed it, with any discrepancies resolved through a discussion. Eligibility criteria were used to guide our rating of indirectness. To assess imprecision and to establish the target of certainty ratings, extracted outcome data (i.e., including relative and absolute effects) were provided to the guideline Working Group to make their partially contextualized judgments on effect sizes (i.e., trivial, small, moderate, or large) for a given intervention or comparator and considering other contextual factors as necessary. Information on the procedure of effect size ratings and final effect size judgments can be found in Additional file 5: Appendix 5. The imprecision ratings for the outcomes extracted from the newly included studies based on the new search update were performed by study reviewers based on the effect size judgments mapping set by the Guideline Working Group.

### Changes from protocol

Firstly, for feasibility and with the consultation from the Working Group, we further excluded comparative effectiveness data (i.e., as mentioned under the KQ2b of the protocol [47]) as well as data on populations with comorbidities after all screening, data extraction, and RoB assessment [58–64]. Also, we initially excluded studies on e-cigarettes compared with usual care and where usual/standard care might have included other active interventions such as NRT. However, upon discussion and clarification of PICOS with the guideline Working Group, we later included these studies if the usual care was provided in both study arms. This is distinct from studies that directly compared e-cigarettes to another intervention (i.e., not usual care, and without isolating the effect of e-cigarettes), which were excluded.

## Results

### Search results

**Fig. 1:**
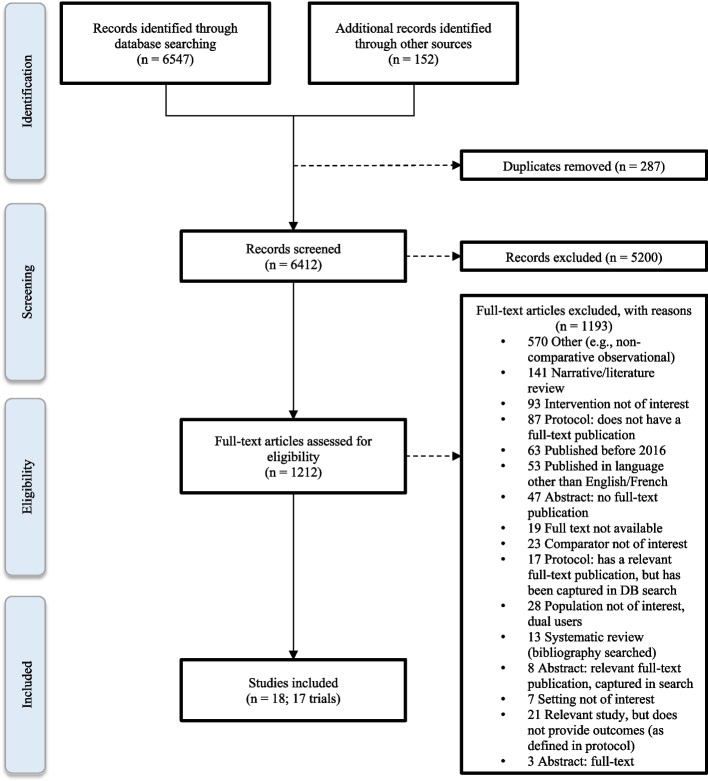
PRISMA flow diagram

The electronic searches resulted in 6547 citations. As this was an update from the 2016 Hartmann-Boyce systematic review [29], the 51 included studies and ongoing trials registries were uploaded, of which 18 were already captured in the database search and therefore quarantined. Additionally, 152 records from grey literature searching were added to the search results. A total of 6412 unique records were evaluated based on title and abstract, with 1212 full-text studies being reviewed. Among the 1212 full texts, we included 18 studies on 17 trials [65–82]. Protocols and abstracts registered or published prior to 2016 were considered, as any full-text publications may be published in 2016 or later. Additionally, the bibliographies from 12 systematic reviews published in 2016 or later (Additional file 2: Appendix 2) were searched for any potentially relevant studies published from 2016 onward. No new citations were added from searching systematic reviews. A PRISMA flow diagram is provided in Fig. 1 and a list of excluded studies is provided in Additional file 4: Appendix 4.

### Characteristics of included studies

Additional file 6: Appendix 6 provides details of the study characteristics of the included studies. Briefly, 5 studies were performed in the USA (68 participants) [66], (40 participants) [70], (837 participants) [78], (638 participants) [79] and (520 participants) [82], 5 in Italy (ECLAT trial) (300 participants) [67, 68], (1355 participants) [74], (73 participants) [75] and (210 participants) [81], 4 in the UK (408 participants) [65], (80 participants) [69], (135 participants) [77] and (80 participants) [80], 1 in Belgium (48 participants) [71], 2 in New Zealand (657 participants) [72] and (1124 participants) [76], and 1 in Canada (376 participants) [73]. Studies were published between 2013 and 2023. Twelve different e-cigarette models with varying nicotine concentrations were evaluated, including Joyetech eGo-C (18 mg/mL), Kanger T2-CC (18 mg/mL), eGo style 2nd generation (24 mg/mL), Categoria model 401 (7.2 mg/mL), BluCig 1st generation (16 mg/mL), BluPlus + (24 mg/mL), a prototype e-vapor product (2.0% nicotine; 2.7 mg/capsule), Vype 2nd generation (6, 12, 18 mg/mL), NJOY e-cig (15 mg/mL), E-cig (VP5 e-cigarette kit (8 mg/mL), 2nd generation eVOD (e-cig kit, 18 mg/mL) and Innokin T18E Smok and TECC mini with variable voltage. Comparator groups included no intervention, waitlist, placebo e-cigarette (0 mg/mL of nicotine), and usual/standard care. Among the six trials (Caponnetto 2013 [67] and Russo 2016 [68] reported on the same participants from one trial) the mean age (SD) ranged from 34.1 (10.6) years to 53 (10.1) years, 55.1% (520/944) of the participants were male, and all studies excluded pregnant women. Most studies took place in an academic research setting, with Holliday 2019 [69] taking place at a dental office among patients with periodontitis and Dawkins 2020 recruited participants from homeless centers [80].

### Risk of bias

The methods used for randomization were considered low risk in most studies; however, allocation concealment was poorly reported, leading to a judgment of unclear in nine of the 18 studies [65–68, 70, 71, 75, 81, 82]. In studies in which blinding was possible (e.g., e-cigarette with nicotine vs e-cigarette with no nicotine), a judgment of low RoB was given. In other studies, where blinding was not possible (e.g., e-cigarette vs no intervention), if the outcome was objective (e.g., abstinence validated with exhaled carbon monoxide reading), a judgment of low RoB was given. For all other comparisons and outcomes, where a lack of blinding could impact the outcome (e.g., AEs), a judgment of high RoB was given. There was a mix of judgments for incomplete outcome data, as some authors used intention to treat (ITT) analysis (i.e., low risk), did not report the number of participants contributing to an outcome (i.e., unclear risk), or reported only on those who contributed to the outcome with a high loss to follow-up (i.e., high risk). Most studies referred to a clinical trials registry, which allowed for better judgments around selective outcome reporting. However, several studies were rated at high RoB as they either did not include an outcome in the registry which was then reported in the publication, or they listed an outcome in the registry which was then not reported in the publication [69, 70, 75, 83]. One study reported a funder that was also the developer of the e-cigarette protocol used in the trial [65]. Another reason for a high RoB for the ‘other’ domain was an increased likelihood that participants in the control group were exposed to e-cigarettes with nicotine during the trial. Overall, outcomes from most studies were at high or unclear RoB. Smoking abstinence, reduction in tobacco use frequency, and AEs outcomes from three studies had a low risk of bias [72, 76, 77], while the other studies had those outcomes at a high RoB [66, 69, 73, 75, 78–83]. Furthermore, all the outcomes were judged to have an unclear RoB from five studies [66, 67, 70, 71, 73]. The results table and the RoB assessments can be found in Additional file 7: Appendix 7. The GRADE ratings can be found in Additional file 8: Appendix 8 and included analyses of intervention and comparator in Additional file 9: Appendix 9.

### Certainty of the evidence

As there was only one study for most comparisons, inconsistency was rated as no serious concern. Publication bias was rated as no serious concern as there were no concerns around suppression or non-publication of results. Due to the small sample sizes, variation in e-cigarette devices and liquids used, and variation in how AEs were reported, all AEs in the GRADE tables are reported narratively. Detailed reasons for ratings are provided in each GRADE summary of findings table footnote section, reported for each comparison in Additional file 8: Appendix 8.

### E-cigarettes with nicotine versus no intervention, usual care, waitlist, or other intervention

#### Benefits

Two RCTs compared e-cigarettes with nicotine to no intervention [73, 75], with behavioral support offered in both groups. Compared to no intervention, 86 more people per 1000 (95% CI 21 fewer to 338 more; *n* = 1140 participants) on e-cigarettes with nicotine were smoking abstinent at the 6-month follow-up, although evidence was of very low certainty (rating down twice for risk of bias and imprecision) [75]. Those two studies also assessed the impact of e-cigarettes on smoking reduction measures (i.e., in terms of exhaled carbon monoxide [eCO] levels or the number of daily cigarettes smoked); however, there was a very low level of evidence certainty of evidence.

Holliday 2019 compared e-cigarettes with nicotine to usual care. Compared to usual care, 100 more people per 1000 (95% CI 18 fewer to 649 more; *n* = 180 participants) receiving intervention reported smoking abstinence at 6 months follow-up; however, the level of evidence certainty was very low (rating down twice for risk of bias, imprecision and once for indirectness) [69]. Additionally, we have a very low level of evidence certainty for other outcome measures of smoking reduction (i.e., reduction in salivary cotinine, anabasine, and eCO levels) reported by the same study.

Walker 2020 compared e-cigarettes with nicotine in combination with behavioral therapy to another group that received only behavioral therapy, with nicotine patches offered under usual care in both groups [76]. Compared to the comparator group, 46 more people per 1000 (95% CI 2 fewer to 200 more; *n* = 1, 625 participants) in the intervention group (i.e., e-cigarette with nicotine plus behavioral therapy plus usual care) reported being smoking abstinent at 6 months follow-up. Also, at 6 months follow-up, 127 more people per 1000 (95% CI 30 more to 288 more; *n* = 1, 625 participants) receiving the intervention were point prevalence abstinent compared to those in the comparator group. The level of evidence certainty was rated as low. Additionally, 179 more people per 1000 (95% CI 61 more to 340 more; *n* = 1, 625 participants) receiving e-cigarettes with nicotine in combination with behavioral therapy and standard care reported > 50% reduction in the number of cigarettes per day (CPD) than the comparator group; the level of evidence certainty was rated as low (Additional file 8: Appendix 8: Table S3, S7, and S9).

Myers Smith 2022 compared Nicotine e-cigarettes to another group that received interventions like Nicotine Replacement Treatment (NRT) choices [nicotine patches, chewing gum, nasal spray, microtab, inhalator, and mouth spray] [77]. Compared to the comparator group, 161 more people per 1000 (95% CI 15 more to 785 more; *n* = 1, 135 participants) in the intervention group reported being smoking abstinent at 6 months follow-up. The level of evidence certainty was rated low. Also, at 6 months follow-up, 206 more people per 1000 (95% CI 36 more to 600 more; *n* = 1, 135 participants) in the intervention group self-reported being smoking abstinent with verified eCO levels of < 8 ppm than the comparator group. The level of evidence certainty was rated low. Also, 299 more people per 1000 (95% CI 112 more to 560 more; *n* = 1135 participants) receiving nicotine e-cigarettes reported a > 50% reduction in the number of daily cigarettes smoked than the comparator group. Additionally, 203 more people per 1000 (95% CI 36 more to 681 more; *n* = 1, 135 participants) in the nicotine e-cigarettes group reported smoking frequency reduction in terms of reduced eCO levels of ≥ 50% compared to baseline. The level of evidence certainty was rated low (Additional file 8: Appendix 8: Table S12).

Xu 2023 compared nicotine e-cigarettes to another group receiving Quit advice [78]. At 6 months follow-up, 136 more people per 1000 (95% CI 55 more to 289.3 more; *n* = 1, 837 participants) self-reported being smoking abstinent in the past 30 days compared to the comparator group. The level of evidence certainty was rated very low. At 12 months follow-up, 111 more people per 1000 (95% CI 42 more to 228 more; *n* = 1837 participants) self-reported being smoking abstinent in the past 30 days compared to the comparator group. The level of evidence certainty was rated very low. Additionally, 40 fewer people per 1000 (95% CI 47 fewer to 34 fewer; *n* = 1837 participants) in the intervention group self-reported reducing the number of daily cigarettes smoked than the comparator group at 6 months follow-up. The level of evidence certainty was rated very low. Also, 33 fewer people per 1000 (95% CI 53 fewer to 10 fewer; *n* = 1, 837 participants) in the intervention group self-reported reducing the number of daily cigarettes smoked than the comparator group at 12 months follow-up. The level of evidence certainty was rated very low (Additional file 8: Appendix 8: Table S13).

Dawkins 2020 compared the nicotine e-cigarette group with another group receiving usual care [80]. At 6 months follow-up, 178 more people per 1000 (95% CI 103 fewer to 975 more; *n* = 1, 80 participants) in the nicotine e-cigarettes group reduced the number of daily cigarettes smoked by at least 50% than the comparator group. The level of evidence certainty was rated very low. Also, at 6 months, 50 fewer people per 1000 (95% CI 190 fewer to 403 more; *n* = 1, 80 participants) in the nicotine e-cigarette group self-reported a 50% reduction in expired CO levels than the comparator group. The level of evidence certainty was rated very low. In terms of self-reported health-related quality of life (QoL) measured using the EQ5D-3L (i.e., descriptive system converted to a utility value ranging from 0 [death] to 1 [perfect health]), the mean (SD) QoL score at 6 months in the intervention group was 0.653 (0.36) and in the usual care group was 0.691 (0.238). Similarly, the self-reported QoL measured using the HRQoL-Visual Analogue Scale (VAS) [perceived health on the day of administration, ranging from 0 (death) to 100 (perfect health)] at 24 weeks showed the mean (SD) QoL score as 61.8 (21.6) in the intervention compared to 61 (22.5) in the comparator group. The level of evidence certainty was rated very low (Additional file 8: Appendix 8: Table S15).

#### Harms

Two RCTs (*n* = 1808) assessed AEs in e-cigarettes with nicotine group to no intervention group at 12 to 16 weeks of follow-up [66, 83] but the certainty of the evidence was very low (rated down twice for risk of bias and once or twice for imprecision)**.** One cohort study at 4 years of follow-up reported no serious AE (SAEs) and no higher risk with the nicotine e-cigarette group in comparison to traditional tobacco smoking [74]. When comparing e-cigarettes with nicotine to the waitlist or usual care, two RCTs provided evidence of very low certainty (rated down twice for risk of bias and once or twice for imprecision) (waitlist: no clear details on types of complaints and usual care: various dental events 20 vs 35 (e-cigarette vs usual care)) between groups in reporting AEs [69, 71]. One RCT examining nicotine e-cigarettes with behavioral support to no intervention and behavioral support provided a very low level of evidence certainty (rated down twice for risk of bias and imprecision) on serious and mild AEs at 12 to 24 weeks of follow-up [73]. Lastly, one RCT found eight fewer people per 1000 (95% CI 15 fewer to 84 more; *n* = 1, 625 participants) reporting a serious adverse event in nicotine e-cigarettes plus behavioral support plus nicotine patches group compared to behavioral support plus nicotine patches alone; the level of evidence certainty was rated low [76]. The same study found there is probably little to no difference in possible adverse events reporting at 6 months follow-up for both the intervention vs. control groups (i.e., vivid dreams 12 (4%) vs 6 (10%); itchiness 12 (4%) vs 2 (3%); redness, swelling at patch site 10 (3%) vs 5 (8%); dry mouth or throat 10 (3%) vs 0 (0%); cough 15 (4%) vs 0 (0%); nausea 6 (2%) vs 2 (3%); headache 6 (2%) vs 1 (2%); the level of evidence certainty was rated moderate. While compared to the other intervention or usual care group, nicotine e-cigarettes showed a change in body mass index (BMI) by MD 0.5 lower from baseline (95% CI 0.57 lower to 0.43 lower; *n* = 1, 625 participants) and weight change by MD 0.7 kg lower from baseline (95% CI 0.88 lower to 0.52 lower; *n* = 1, 625 participants) at 6 months follow-up. The level of evidence certainty was rated as moderate (rating down once for imprecision). The level of evidence certainty from the studies examining emotional state [69] and all-cause mortality [66] was very low (rated down once in one study and twice in the other for risk of bias, and once or twice for imprecision). Myers Smith 2022 captured mild adverse events [77]. At 6 months follow-up, in the EC arm, there was a report of dry mouth (*n* = 1) and cough/throat/chest irritation (*n* = 3), while in the comparator arm, there was a report of itchiness (*n* = 1) and nausea (*n* = 1). The level of evidence certainty was rated very low. Dawkins 2020 also captured mild adverse events using the 9-item patient health questionnaire (PHQ-9) for depression and the 7-item generalized anxiety disorder (GAD) questionnaire [80]. For the intervention group, the mean (SD) score for mental health at 24 weeks follow-up was 5.63 (6.34) for the GAD questionnaire and 7.12 (7.22) for the PHQ-9 questionnaire. For the comparator group, the mean (SD) score for mental health at 24 weeks follow-up was 12.70 (4.42) for the GAD questionnaire and 10.82 (7.23) for the PHQ-9 questionnaire. The level of evidence certainty was rated very low (Additional file 8: Appendix 8: Table S1, S2, S3, S7, S9, S12, and S15).

### E-cigarettes with nicotine versus e-cigarettes without nicotine

#### Benefits

Two studies compared e-cigarettes with nicotine to e-cigarettes without nicotine. In one study, 60 more people per 1000 (95% CI 7 fewer to 232 more; *n* = 1300 participants) reported being smoking abstinent in the nicotine e-cigarette group compared to the group assigned non-nicotine e-cigarettes at 24-week follow-up [67, 68]. In another study, 100 more people per 1000 (95% CI 59 fewer to 871 more; *n* = 140 participants) reported being smoking abstinent in the nicotine e-cigarettes group compared to the non-nicotine e-cigarettes group [70]. This study offered nicotine patches and counseling sessions as standard care therapy in both groups. The level of evidence certainty was rated very low in both studies (Additional file 8: Appendix 8: Table S4 and S5). Both studies also measured smoking reduction outcomes between the comparative groups. There was a small reduction by MD 2.54 in the mean number of cigarettes smoked/day at the 24-week follow-up, with the level of evidence certainty rated very low (rating down once for RoB, indirectness, and imprecision) [70]. Also, for the reduction in smoking frequency at 24 and 52 weeks, 30 more people per 1000 (95% CI 47 fewer to 162 more; *n* = 1300 participants), and 25 fewer people per 1000 (95% CI 72 fewer to 68 more; *n* = 1300 participants) in the nicotine e-cigarette group showed > 50% reduction in the number of cigarettes smoked per day from baseline [67, 68]. The level of evidence certainty was rated as very low (Additional file 8: Appendix 8: Table S4 and S5).

Three studies compared nicotine e-cigarettes to non-nicotine e-cigarettes with behavioral support offered as co-intervention in both groups [72, 73, 75]. Two studies measured continuous smoking abstinence (i.e., eCO levels verified as < 10 ppm or ≤ 7 ppm), and at 6 months of follow-up [72, 75]. In one study, 28 more people per 1000 (95% CI 68 fewer to 229 more; *n* = 1, 140 participants) in the nicotine e-cigarettes group reported being smoking abstinent at 6 months follow-up compared to the non-nicotine e-cigarettes group [75]. The level of evidence certainty was rated very low (rated down twice for RoB and imprecision). In another study, 32 more people per 1000 (95% CI 19 fewer to 196 more; *n* = 1, 657 participants) in the nicotine e-cigarettes group reported being smoking abstinent compared to the non-nicotine e-cigarettes group [72]. The level of evidence certainty was rated low (rating down twice for imprecision). Considering the reduction in smoking frequency, all three studies assessed this measure across both groups with different outcome measures, i.e., daily reduced cigarette consumption by 50% or greater, change in mean number of cigarettes smoked since baseline, or reduction in eCO levels [72, 73, 75]. In one study, 118 more people per 1000 (95% CI 18 fewer to 298 more; *n* = 1, 657 participants) reported a ≥ 50% reduction in daily cigarette consumption in the nicotine e-cigarettes group compared to the non-nicotine e-cigarettes group [72]. The level of evidence certainty was rated moderate (rating down once for imprecision). For the reduction in smoking frequency measured as change in mean number of daily cigarettes smoked since baseline at 24 weeks follow-up, the nicotine e-cigarette group had a − 10.7 change in mean number of daily cigarettes smoked since baseline with a − 9.1 change in the non-nicotine e-cigarette group [73]. The level of evidence certainty was rated very low (rated down twice for risk of bias and imprecision). Lastly, in Lucchiari 2020, the reduction in smoking frequency measured as a change in eCO levels, the MD was 3.27 parts per million (ppm) higher (95% CI 6.56 lower to 0.02 higher; *n* = 1,140 participants) in the nicotine e-cigarette group than the non-nicotine e-cigarettes group at 6 months of follow-up [75]; although, the level of evidence certainty was rated very low (Additional file 8: Appendix 8: Table S6).

Walker 2020 assessed nicotine e-cigarettes compared to non-nicotine e-cigarettes in addition to nicotine patches and behavioral support offered to both groups [76]. In terms of smoking abstinence defined as having eCO levels ≤ 9 ppm, 30 more people per 1000 (95% CI 1 more to 79 more; *n* = 1999 participants) were abstinent at 6 months follow-up in the nicotine e-cigarettes group compared to those with non-nicotine e-cigarettes. In the same trial, 72 more people per 1000 (95% CI 18 more to 140 more; *n* = 1999 participants) in the nicotine e-cigarettes group self-reported being point prevalent abstinent compared to the non-nicotine e-cigarettes group. Similarly, 72 more people per 1000 (95% CI 23 more to 138 more; *n* = 1999 participants) in the nicotine e-cigarettes group self-reported being continuously abstinent. With the measure of reduction in smoking frequency, 60 more per 1000 (95% CI 4 fewer to 132 more; *n* = 1, 999 participants) in the nicotine e-cigarettes reported ≥ 50% reduction in the number of cigarettes/day since baseline than the non-nicotine e-cigarette group. The level of evidence certainty was rated as moderate for all of these outcomes (rating down once for imprecision) (Additional file 8: Appendix 8: Table S8).

Carpenter 2023 compared e-cigarettes with the nicotine group to the other group provided with no e-cigarettes [79]. At 6-month follow-ups in the general population group, 55 more people per 1000 (95% CI 0 to 147 more; *n* = 1, 638 participants) in the nicotine e-cigs group were smoking abstinent than the comparator group. The level of evidence certainty was rated as very low. In the high motivation to quit group, at 6 months follow-up, 34 more people per 1000 (95% CI 67 fewer to 229 more; *n* = 1, 174 participants) reported smoking abstinence than the comparator group. Subsequently, in the low motivation to quit group, 63 more people per 1000 (95% CI 4 more to 195 more; *n* = 1, 464 participants) in the nicotine e-cigarette group reported smoking abstinence than the comparator group. The level of evidence certainty was rated very low. For floating abstinence, in the general group, 44 more people per 1000 (95% CI 12 fewer to 132 more; *n* = 1, 638 participants) reported having ever achieved 7 days of non-smoking throughout follow-up in the intervention group than the comparator group. For the high motivation to quit group, 121 more people per 1000 (95% CI 17 fewer to 378 more; *n* = 1, 174 participants) reported having ever achieved 7 days of non-smoking throughout follow-up in the intervention group compared to the control group. For the low-motivation group, 17 more per 1000 (95% CI 35 fewer to 107 more; *n* = 1, 464 participants) reported having ever achieved 7 days of non-smoking throughout follow-up in the intervention group compared to the control group. The level of evidence certainty was rated very low. For the reduction in smoking frequency, 97 more people per 1000 (95% CI 20 more to 205 more; *n* = 1, 638 participants) in the general intervention group self-reported a smoking reduction in the number of daily cigarettes smoked by at least 50% than the comparator group. For the reduction in smoking frequency, 119 more people per 1000 (95% CI 28 fewer to 362 more; *n* = 1, 174 participants) in the high motivation to quit group self-reported a smoking reduction in the number of daily cigarettes smoked by at least 50% than the comparator group. For the reduction in smoking frequency, 90 more people per 1000 (95% CI 6 more to 218 more; *n* = 1, 464 participants) in the low motivation to quit group self-reported a smoking reduction in the number of daily cigarettes smoked by at least 50% than the comparator group. The level of evidence certainty was rated very low (Additional file 8: Appendix 8: Table S14).

Lucchiari 2022 compared e-cigarettes with a nicotine group to e-cigarettes without nicotine, and support (i.e., psychological counseling) was offered in both groups [81]. At 12 months follow-up, ten fewer people per 1000 (95% CI 124 fewer to 204 more; *n* = 1, 140 participants) in the intervention group self-reported complete tobacco abstinence validated by eCO levels < / = 7 ppm than the comparator group. The level of evidence certainty was rated very low. Additionally, for a reduction in tobacco smoking frequency, smokers in the intervention arm smoked a mean of 16.18 tobacco cigarettes (SD = 7.23) versus a mean of 13.71 (7.22) cigarettes in the control arm. The level of evidence certainty was rated low. Lucchiari 2022 also compared the nicotine e-cigarette plus psychological counseling group to another group that offered psychological counseling only. At 12 months follow-up, 83 more people per 1000 (95% CI 45 fewer to 343 more; *n* = 1, 140 participants) in the intervention group self-reported being smoking abstinent than the control group. The level of evidence certainty was rated very low. Additionally, for the reduction in smoking frequency/quantity, smokers in the intervention arm smoked a mean of 16.18 tobacco cigarettes (SD = 7.23) versus a mean of 13.93 (7.20) cigarettes smoked in the control arm at month 12. The level of evidence certainty was rated low (Additional file 8: Appendix 8: Table S16 and S17).

Foulds 2022 compared nicotine e-cigarettes with non-nicotine cigarette substitutes (i.e., plastic tubes with no electronics or aerosol) [82]. At 6 months follow-up, 77 more people per 1000 (95% CI 6 more to 289 more; *n* = 1, 520 participants) in the intervention group self-reported smoking abstinence (i.e., validated by eCO < 10 ppm) than the comparator group. Additionally, 69 more people per 1000 (95% CI 3 more to 358 more; *n* = 1, 520 participants) in the intervention group self-reported 28 plus days of smoking abstinence than the control group at 6 months follow-up. The level of evidence certainty was rated very low. Lastly, at 6 months follow-up, the mean (SD) number of days in the e-cigs with nicotine group where the participants reported being abstinent was 15.6 (36.4), and that in the control group was 5.3 (18.5). The mean difference (95% CI) reported between both groups was 10.29 (3.2 to 17.4). The evidence certainty was rated low. Foulds 2022 also compared nicotine e-cigarettes with non-nicotine e-cigarettes. At 6 months follow-up, 100 more people per 1000 (95% CI 7 more to 799 more; *n* = 1, 520 participants) in the intervention group self-reported 7-day point prevalence abstinence (i.e., validated by eCO < 10 ppm) than the control group. Subsequently, 77 more people per 1000 (95% CI 3 more to 638 more; *n* = 1, 520 participants) in the intervention group self-reported 28 plus days of smoking abstinence than the control group. The level of evidence certainty was rated very low. Lastly, at 12 months follow-up, the mean (SD) number of days in the e-cigs with nicotine group where the participants reported being totally abstinent was 15.6 (36.4), and that in the control group was 4.7 (17). The mean difference (95% CI) reported between both groups was 10.87 (3.9 to 17.8). The level of evidence certainty was rated low (Additional file 8: Appendix 8: Table S18 and S19).

#### Harms

Two studies (i.e., Baldassari 2018 and Caponnetto 2013) reported no SAEs but some AE complaints following e-cigarette use (i.e., abnormal dreams, anxiety, fatigue, headache, insomnia nausea) at 24 and 52 weeks of follow-up; however, there was little to no difference between groups, and the level of evidence certainty was unable to be assessed [67, 70]. Bullen 2013 captured SAEs, and at 6 months follow-up, the nicotine e-cigarette group experienced 27 events (20%), and the control group had 5 events (14%); this was judged as little to no difference between groups as no events were related to product use. The level of evidence certainty was rated moderate (rating down once for imprecision) [72]. Likewise, Eisenberg 2020 also captured some SAEs adjudicated by an endpoint evaluation committee, and at 12 to 24 weeks follow-up, the nicotine e-cigarettes group had experienced 2 (1.6%), and the non-nicotine e-cigarette group had 2 (1.6%) events [73]; although, the level of evidence certainty was rated very low (rating down twice for risk of bias and imprecision). Two studies captured some mild non-serious AEs at different follow-up time points in both groups [73, 75]. At 12 weeks, the intervention group had experienced 120 (94%), and the control group had experienced 118 (93%) AEs (i.e., cough, dry mouth, headache, dizziness) [73]. Likewise, the Lucchiari 2020 trial also reported side effects likely related to e-cigarette use at 3 and 6 months of follow-up in both groups [75]. At 3 months, 5.7% of the intervention group had experienced side effects (10% burning throat, 1.4% cough, 1.4% headache, 1.4% stomachache) and 2.9% in the control group (2.9% burning throat). At 6 months, 15.9% of the intervention group had experienced side effects (5.8% burning throat, 5.8% cough, 1.4% headache, 4.3% insomnia, 1.4% stomachache) and 5.6% in the control group (2.8% burning throat, 7% cough, 1.4% headache, 4.2% insomnia). The level of evidence certainty was rated as very low in both cases (rating down twice for RoB and imprecision). Walker 2020 also reported on participants with a SAE and there were 12 fewer people per 1000 (95% CI 27 fewer to 16 more; *n* = 1, 999 participants) in the nicotine e-cigarettes group compared to the non-nicotine e-cigarettes group reporting them. This was judged as little to no difference as no events were related to the intervention in either group. The level of evidence certainty was rated as low (rating down once for imprecision). The same trial also reported possible adverse outcomes like change in BMI (i.e., MD 0.3 lower; 95% CI 0.32 lower to 0.28 lower; *n* = 1, 999 participants) and weight (i.e., MD 0.7 kg lower; 95% CI 0.76 lower to 0.64 lower; *n* = 1, 999 participants) from baseline at 6 months follow-up and the level of evidence certainty was rated as moderate (rating down once for imprecision). Some other possible AEs (i.e., vivid dreams, itchiness, dry cough, nausea) at 6 months were reported for the intervention and control group in the same trial; there was little to no difference in event rates in study arms. The level of evidence certainty was rated moderate (rated down once for imprecision). Carpenter 2023 captured mild adverse events [79]. Within the e-cigarette with nicotine group, 180 people (42%) reported a total of 360 adverse events (AEs), of which 7 (2%) were severe, 113 (31%) were moderate and 232 (64%) were mild. Most common AEs: Headaches (12%) and increased phlegm (12%). One serious event observed: asthma-induced hospitalization; possibly attributed to increased nebulizer use and/or e-cigs. Within the control group, 86 people (41%) reported a total of 197 AEs, of which 7 (4%) were severe, 60 (30%) were moderate, and 124 (63%) were mild/most common reported AEs: cough (20%), increased phlegm (18%) and headaches (8.1%). As a result, at 6 months follow-up, 12 more people per 1000 (95% CI 65 fewer to 102 more; *n* = 1, 638 participants) in the intervention group reported mild adverse events than the control group. The level of evidence certainty was rated very low. Lucchiari 2022 also captured mild adverse events. In the e-cigarette with nicotine plus support group the mean (SD) scores for mental health at 12 months follow-up was 12.17 (2.20) for Anxiety and 9.13 (1.57) for Depression in the Hospital Anxiety and Depression Scale (HADS). While in e-cigarette without nicotine plus support group, the mean (SD) scores for mental health at 12 months follow-up was 12.45 (2.37) for Anxiety and 8.90 (1.81) for Depression in the Hospital Anxiety and Depression Scale (HADS). The level of evidence certainty was rated very low (Additional file 8: Appendix 8: Table S4, S5, S6,S8,S14, and S16).

### E-cigarettes without nicotine versus other interventions

#### Benefits

Walker 2020 compared e-cigarettes without nicotine with the offered behavioral support and nicotine patches to those provided with behavioral support and nicotine patches alone [76]. For the tobacco use abstinence outcome, compared to the other interventions group, 26 more people per 1000 in the e-cigarettes without nicotine group reported being smoking abstinent at the 6-month follow-up (95% CI 24 fewer to 122 more; *n* = 1, 624 participants). In terms of smoking abstinence measured as eCO levels ≤ 9 ppm, 16 more people per 1000 in the e-cigarettes without nicotine group reported being smoking abstinent at 6 months follow-up (95% CI 12 fewer to 109 more; *n* = 1, 624 participants). Also, 55 more people per 1000 in the e-cigarettes without nicotine group reported being point prevalent abstinent compared to the control group (95% CI 15 fewer to 171 more; *n* = 1, 624 participants). For the reduction in smoking frequency measured, in the e-cigarettes without nicotine group 125 more people per 1000 reported ≥ 50% reduction in the number of cigarettes/days since baseline in comparison to those on other interventions and nicotine patches alone (95% CI 20 more to 269 more; *n* = 1, 624 participants). The level of evidence certainty was rated low (rating down twice for imprecision) (Additional file 8: Appendix 8: Table 10).

Two small RCTs compared e-cigarettes without nicotine with behavioral support to the group offered behavioral support alone [73, 75]. Considering smoking cessation, in one study, 57 more people per 1000 in the e-cigarette without nicotine group were smoking abstinent at the 6-month follow-up than the comparator group (95% CI 35 fewer to 282 more; *n* = 1, 140 participants) [75]. This study reported a reduction in smoking frequency based on eCO levels of MD 1.24 particles per million (ppm) lower in the intervention group than the control group (95% CI 2.38 lower to 4.86 higher; *n* = 1, 140 participants) [75]. Another study measured change in the mean number of daily cigarettes smoked since baseline, where the intervention group had a − 9.1 change and the control group had a − 5.5 change [73]. The level of evidence certainty was rated as very low for all these outcomes (rating down twice for RoB and twice for imprecision in all cases) (Additional file 8: Appendix 8: Table S11).

#### Harms

Walker 2020 assessed AE among participants on e-cigarettes without nicotine and those in other intervention groups [76]. For reporting of SAE, 20 more people per 1000 in the e-cigarette without nicotine group reported an SAE in comparison to the control group (95% CI 11 fewer to 121 more; *n* = 1, 624 participants). This was judged as little to no difference given that no events were related to the intervention. The level of evidence certainty was rated as low (rating down twice for imprecision). With other possible adverse outcomes (i.e., change in BMI and weight from baseline), there was little to no difference between groups, and the level of evidence certainty was rated moderate. One small RCT reported little to no difference between groups with SAEs (the intervention group had experienced 2 (1.7%) and the control group had experienced 2 (1.7%) and mild AEs (i.e., cough, dry mouth, headache, rhinitis, throat irritation, dyspnea, sore throat) observed at weeks 12 to 24; however, the level of evidence certainty was rated very low (rating down twice for RoB and imprecision) (Additional file 8: Appendix 8: Table S10 and S11) [73].

## Discussion

This review found 18 studies presenting results from 17 trials, comparing e-cigarettes with nicotine to e-cigarettes without nicotine and e-cigarette (with or without nicotine) to other interventions (i.e., no intervention, waitlist, standard/usual care, quit advice, or behavioral support). Overall, ten studies provided evidence of the benefits of e-cigarettes in terms of smoking abstinence (i.e., measured through a reduction in eCO levels, 7-day point prevalence, or continuous) measured at various follow-up time points [67, 70, 72, 75–79, 81, 82]. For the reduction in smoking frequency (i.e., measured as a reduction in biomarkers such as eCO levels, mean number of daily cigarettes, > 50% of reduction per day), measured at different follow-up time points, nine studies provided data [67, 69, 72, 75–77, 79–81] on benefits. Low, very low, or moderate evidence on smoking cessation from those studies indicates that e-cigarettes may or probably increase smoking cessation.

All studies reported on AEs are also labeled as side effects or complaints. AEs varied in how they were reported, for example, the total number of participants experiencing an event, the number of participants experiencing each event, the total number of events (i.e., where an individual participant may contribute to one or more events), if the event was related to the study intervention, and the level of severity of the event. There was also a variety of different devices and cartridges being used across studies. Although there is some guidance through the Core Outcome Measures in Effectiveness Trials (COMET) for abstinence in tobacco studies [84], there was currently no guidance for AEs or other outcomes identified in most included studies of this review, except in three [72, 73, 76]. In two studies, AEs were categorized as serious or non-serious based on the international guidelines or classifications in line with the recommended practice [72, 76] while evaluated by an end-point evaluation committee in the third study [73]. Two studies evaluated mild AEs using ad hoc items (i.e., 9-item patient health questionnaire [PHQ-9], 7-item generalized anxiety disorder questionnaire, and Hospital Anxiety and Depression scale [HADS]) [80, 81]. For AEs, 15 studies [66–73, 75–77, 79–81] reported mild AEs. Overall, there was little to no difference between groups in any of these outcomes, varying certainty of evidence.

Among the studies that reported serious AEs, none were suspected to be related to the study product, and the evidence was judged to be very low or low certainty depending on the study. There is some concern about the overall safety of e-cigarettes as those events are not attributable to the contents of the liquid, so considerable attention is being given to this as more information emerges. The duration of the studies included in our review was short, ranging from 12 weeks to 12 months, with most being less than 6 months. In some cases, the duration of the intervention (i.e., supplying e-cigarettes) was shorter than the follow-up period, and not all studies reported on how many individuals were still using the product at the longest follow-up time, which may not be enough for AEs attributed to long-term use to emerge.

The strengths of our work lie in the use of an a priori protocol, with any amendments reported and justified, and peer-review evaluation of our search strategies. Also, with input from the guideline working group, clinical experts, and patients, we assembled a group of outcomes of importance to those stakeholders. Although we aimed to update the 2016 Hartmann-Boyce systematic review [29], there has already been a 2022 update [21] of our candidate review with similar results as ours; however, it is a living systematic review with more updated and recent studies combined in its analyses. Also, with time and resource constraints, we could not formally analyze the results from those recent studies in our review. There are some important limitations to consider in our systematic review. Firstly, it became difficult to perform a meta-analysis and effect-size reporting, as we were unable to pool results due to clinical heterogeneity. Outcome reporting varied between studies. For example, reduction in smoking frequency using a self-report of ≥ 50% reduction in the number of cigarettes smoked daily or using biomarkers such as exhaled carbon monoxide, salivary cotinine, and salivary anabasine. Further, change in weight was reported as a percent change in weight from baseline and absolute weight change from baseline to follow-up (Additional file 10: Appendix 10).

Several studies did not provide sufficient details, leading to unclear judgments in the RoB assessments. Allocation concealment was of particular concern. Study authors would benefit from reporting guidelines, specifically the Consolidated Standards of Reporting Trials (CONSORT) checklist [85], which provides a minimum set of recommendations for reporting randomized trials. Outcome switching, the failure to report pre-specified outcomes without justification, is commonly observed among academic papers [86, 87] and can present problems in interpreting results. Several studies in this review had differing outcomes reported in the trial registry or methods section than what was reported in the results section. Any deviations from the protocol should be reported and justified [85]. For example, in Cravo 2016 [65], the only two outcomes reported in the clinical trials registry (NCT02029196) are AEs and exhaled carbon monoxide. This study also provided several other outcomes (e.g., vital signs, lung function tests, hematology) in the methods section under study outcomes. Likewise, Holliday 2019 mentioned some primary outcomes (e.g., Periodontal Inflamed Surface Area [PISA]) as listed in the trial registry and not in the study, while some were not (e.g., QoL) in the trial registry. Baldassari 2019 and Lucchiari 2020 had no information on the strategy for handling missing data, data collection of severe AE, and other missing/incomplete outcomes (e.g., results of relapse and AE, Activity and lifestyle, 12 months follow-up). Carpenter 2017 lacked details on the number of participants contributing to the data [66], and Adriaens 2014, Myers Smith 2022, Xu 2023, and Carpenter 2023 lacked reporting of AE [71, 77–79]. We could not find a justification for these changes. However, with the knowledge about how factors, including exposure to tobacco in the social environment and permissive attitude towards smoking, lower socioeconomic status, or higher levels of psychological distress led to smoking disparities in vulnerable population groups (i.e., Indigenous Canadians, those with incomplete high school education or with addiction disorders and mental illness), we did not focus on those groups and have considered it as another study limitation [88]. Also, it was out of our scope, and we might consider capturing the subgroup data in our future review.

It may be difficult to perform a trial in which all participants randomized to e-cigarette only used this and no other methods (e.g., behavioral therapy, pharmacotherapy) and where the control group only used what was assigned to them, but it is important to note that any other co-interventions received (by design) or used (unintended participant use) during the trial could impact any of the outcome results. We acknowledge another potential limitation as the chances of developing nicotine dependence post-e-cig use as a therapeutic intervention for smoking cessation; however, the evidence is limited [89, 90]. We also acknowledge a potential risk of missing trials in languages other than English and French, as there were 52 potentially relevant studies published in other languages. Additionally, we were unable to retrieve a full-text publication for 45 records; however, as a part of the verification, we searched the bibliographies of 12 relevant systematic reviews published from 2016 to 2019, none of which included any of these studies (Additional file 10: Appendix 10).

## Conclusions

This systematic review provides an evidence synthesis on e-cigarettes’ effectiveness; the data surrounding benefits (e.g., smoking cessation and reduction in smoking frequency) has small or moderate effect sizes with low to moderate evidence certainty for certain comparisons, and very low certainty for others, indicating that e-cigarettes may or probably increase smoking cessation. Likewise, for harms related to e-cigarette use, most studies showed little to no difference between the intervention and control group with low to very low evidence certainty. Also, the duration of studies varied from 12 weeks to 12 months, with most measuring outcomes under 6 months, which may not be long-term enough for attributed AEs to emerge. Consequently, the lack of evidence on long-term benefits (cessation) and potential harms of e-cigarette use in this review suggests an evidence gap that further necessitates more research.

### Supplementary Information

Additional file 1: Appendix 1. PRISMA checklist.

Additional file 2: Appendix 2. Search strategy.

Additional file 3: Appendix 3. Eligibility criteria (PICOS).

Additional file 4: Appendix 4. List of excluded studies.

Additional file 5: Appendix 5. Tobacco effect judgements.

Additional file 6: Appendix 6. Characteristics of included studies.

Additional file 7: Appendix 7. Results tables and RoB assessments.

Additional file 8: Appendix 8. GRADE Evidence Profile and Summary of Findings (SoF) tables.

Additional file 9: Appendix 9: Included analyses.

Additional file 10: Appendix 10. Stakeholder feedback.

## Data Availability

All data generated or analyzed during this study are included in this published article [and its supplementary information files].

## Abbreviations

AMSTAR: A Measurement Tool to Assess Systematic Reviews
AE: Adverse events
CO: Carbon monoxide
E-cigarettes: Electronic cigarettes
eCO/exCO: Exhaled carbon monoxide
GRADE: Grading of Recommendations, Assessment, Development and Evaluation
NRT: Nicotine replacement therapy
PRESS: Peer Review of Electronic Search Strategies
PRISMA: Preferred Reporting Items for Systematic Reviews and Meta-Analyses
RCT: Randomized controlled trial
ROB: Risk of bias
SAE: Serious adverse event
