# Supplementary material for: Effectiveness of e-cigarettes as a stop smoking intervention in adults: a systematic review
Source: Syst Rev. 2024 Jun 29;13:168. doi: 10.1186/s13643-024-02572-7 (PMC11218295; doi:10.1186/s13643-024-02572-7)
Supplement: Supplementary file 2 — Additional file 2: Appendix 2. Search strategy. [file 13643_2024_2572_MOESM2_ESM.docx]

# Appendix B. OVID Multifile search strategy

Database: Embase Classic+Embase <1947 to 2019 July 03>, Ovid MEDLINE(R) ALL <1946 to July 03, 2019>, PsycINFO <1806 to July Week 1 2019>

Search Strategy:

----------------------------------------------------------------------

1 (e-cig$ or ecig$).mp. (8583)

2 electr$ cigar$.mp. (8916)

3 electronic nicotine.mp. (3553)

4 (electronic adj3 vapori*).mp. (58)

5 (e-vape$1 or e-vaping or evape$1 or evaping).mp. (3)

6 (vape or vaper or vapers or vaping).ti,ab. (1752)

7 or/1-6 [E-CIGARETTES] (11998)

8 Smoking Cessation/ (95174)

9 "Tobacco Use Cessation"/ (56840)

10 Smoking/th [therapy] (2009)

11 exp Tobacco Smoking/th [therapy] (372)

12 "Tobacco Use Disorder"/th [therapy] (2848)

13 ((smoking or smoker* or tobacco* or nicotine or cigar? or cigarette* or cigarillo?) adj5 (abstain* or abstinen* or cease or ceased or ceases or cessation* or dehabituat* or desist* or discontinu* or end or ended or ending or ends or "give up" or "giving up" or "gives up" or "gave up" or halt* or quit* or stop*)).tw,kf. (107712)

14 or/8-13 [SMOKING CESSATION] (139311)

15 7 and 14 [E-CIGARETTES - SMOKING CESSATION] (5214)

16 (2016* or 2017* or 2018* or 2019*).dt. (4300188)

17 15 and 16 (1176)

18 17 use medall [MEDLINE RECORDS] (1176)

19 (e-cig$ or ecig$).mp. (8583)

20 electr$ cigar$.mp. (8916)

21 electronic nicotine.mp. (3553)

22 (electronic adj3 vapori*).mp. (58)

23 (e-vape$1 or e-vaping or evape$1 or evaping).mp. (3)

24 (vape or vaper or vapers or vaping).ti,ab. (1752)

25 or/19-24 [E-CIGARETTES] (11998)

26 smoking cessation/ (95174)

27 smoking cessation program/ (3151)

28 exp smoking/th [Therapy] (2119)

29 tobacco dependence/dm, th [Disease Management, Therapy] (2646)

30 ((smoking or smoker* or tobacco* or nicotine or cigar? or cigarette* or cigarillo?) adj5 (abstain* or abstinen* or cease or ceased or ceases or cessation*

or dehabituat* or desist* or discontinu* or end or ended or ending or ends or "give up" or "giving up" or "gives up" or "gave up" or halt* or quit* or stop*)).tw,kw. (108071)

31 or/26-30 [SMOKING CESSATION] (139747)

32 25 and 31 [E-CIGARETTES - SMOKING CESSATION] (5237)

33 (2016* or 2017* or 2018* or 2019*).dc. (6264044)

34 32 and 33 (1836)

35 34 use emczd [EMBASE RECORDS] (1836)

36 (e-cig$ or ecig$).mp. (8583)

37 electr$ cigar$.mp. (8916)

38 electronic nicotine.mp. (3553)

39 (electronic adj3 vapori*).mp. (58)

40 (e-vape$1 or e-vaping or evape$1 or evaping).mp. (3)

41 (vape or vaper or vapers or vaping).ti,ab. (1752)

42 or/36-41 [E-CIGARETTES] (11998)

43 smoking cessation/ (95174)

44 ((smoking or smoker* or tobacco* or nicotine or cigar? or cigarette* or cigarillo?) adj5 (abstain* or abstinen* or cease or ceased or ceases or cessation* or dehabituat* or desist* or discontinu* or end or ended or ending or ends or "give up" or "giving up" or "gives up" or "gave up" or halt* or quit* or stop*)).tw. (107453)

45 43 or 44 [SMOKING CESSATION] (138086)

46 42 and 45 [E-CIGARETTES - SMOKING CESSATION] (5167)

47 (2016* or 2017* or 2018* or 2019*).up. (37671405)

48 46 and 47 (4254)

49 48 use medall,emczd (3759)

50 48 not 49 [PSYCINFO RECORDS] (495)

51 18 or 35 or 50 [ALL DATABASES] (3507)

52 remove duplicates from 51 (2180) [TOTAL UNIQUE RECORDS]

53 52 use medall [MEDLINE UNIQUE RECORDS] (1139)

54 52 use emczd [EMBASE UNIQUE RECORDS] (867)

55 52 not (53 or 54) [PSYCINFO UNIQUE RECORDS] (174)

Hartmann-Boyce Update + Smoking Cessation

**Update**

**2020 Sep 24**

Ovid Multifile

Database: Embase Classic+Embase <1947 to 2020 September 23> , Ovid MEDLINE(R) ALL <1946 to September 23, 2020>, APA PsycInfo <1806 to September Week 2 2020>

Search Strategy:

--------------------------------------------------------------------------------

1 (e-cig$ or ecig$).mp. (12543)

2 electr$ cigar$.mp. (12557)

3 electronic nicotine.mp. (5321)

4 (electronic adj3 vapori*).mp. (66)

5 (e-vape$1 or e-vaping or evape$1 or evaping).mp. (11)

6 (vape or vaper or vapers or vaping).ti,ab. (3749)

7 or/1-6 [E-CIGARETTES] (17698)

8 Smoking Cessation/ (101800)

9 "Tobacco Use Cessation"/ (60843)

10 Smoking/th [therapy] (2216)

11 exp Tobacco Smoking/th [therapy] (490)

12 "Tobacco Use Disorder"/th [therapy] (3121)

13 ((smoking or smoker* or tobacco* or nicotine or cigar? or cigarette* or cigarillo?) adj5 (abstain* or abstinen* or cease or ceased or ceases or cessation* or dehabituat* or desist* or discontinu* or end or ended or ending or ends or "give up" or "giving up" or "gives up" or "gave up" or halt* or quit* or stop*)).tw,kf. (116469)

14 or/8-13 [SMOKING CESSATION] (149540)

15 7 and 14 [E-CIGARETTES - SMOKING CESSATION] (7091)

16 (2016* or 2017* or 2018* or 2019*).dt. (4929661)

17 15 and 16 (1540)

18 17 use medall [MEDLINE RECORDS] (1540)

19 (e-cig$ or ecig$).mp. (12543)

20 electr$ cigar$.mp. (12557)

21 electronic nicotine.mp. (5321)

22 (electronic adj3 vapori*).mp. (66)

23 (e-vape$1 or e-vaping or evape$1 or evaping).mp. (11)

24 (vape or vaper or vapers or vaping).ti,ab. (3749)

25 or/19-24 [E-CIGARETTES] (17698)

26 smoking cessation/ (101800)

27 smoking cessation program/ (3374)

28 exp smoking/th [Therapy] (2453)

29 tobacco dependence/dm, th [Disease Management, Therapy] (2753)

30 ((smoking or smoker* or tobacco* or nicotine or cigar? or cigarette* or cigarillo?) adj5 (abstain* or abstinen* or cease or ceased or ceases or cessation* or dehabituat* or desist* or discontinu* or end or ended or ending or ends or "give up" or "giving up" or "gives up" or "gave up" or halt* or quit* or stop*)).tw,kw. (116843)

31 or/26-30 [SMOKING CESSATION] (149983)

32 25 and 31 [E-CIGARETTES - SMOKING CESSATION] (7128)

33 (2016* or 2017* or 2018* or 2019*).dc. (6927797)

34 32 and 33 (2128)

35 34 use emczd [EMBASE RECORDS] (2128)

36 (e-cig$ or ecig$).mp. (12543)

37 electr$ cigar$.mp. (12557)

38 electronic nicotine.mp. (5321)

39 (electronic adj3 vapori*).mp. (66)

40 (e-vape$1 or e-vaping or evape$1 or evaping).mp. (11)

41 (vape or vaper or vapers or vaping).ti,ab. (3749)

42 or/36-41 [E-CIGARETTES] (17698)

43 smoking cessation/ (101800)

44 ((smoking or smoker* or tobacco* or nicotine or cigar? or cigarette* or cigarillo?) adj5 (abstain* or abstinen* or cease or ceased or ceases or cessation* or dehabituat* or desist* or discontinu* or end or ended or ending or ends or "give up" or "giving up" or "gives up" or "gave up" or halt* or quit* or stop*)).tw. (116115)

45 43 or 44 [SMOKING CESSATION] (148177)

46 42 and 45 [E-CIGARETTES - SMOKING CESSATION] (7013)

47 (2016* or 2017* or 2018* or 2019*).up. (37497345)

48 46 and 47 (4325)

49 48 use medall,emczd (3722)

50 48 not 49 [PSYCINFO RECORDS] (603)

51 18 or 35 or 50 [ALL DATABASES] (4271)

52 remove duplicates from 51 (2637)

53 52 use medall [MEDLINE UNIQUE RECORDS] (1483)

54 (201907* or 201908* or 201909* or 201910* or 201911* or 201912* or 2020*).dt. (1692418)

55 53 and 54 [MEDLINE UNIQUE RECORDS - UPDATE PERIOD] (294)

56 52 use emczd [EMBASE UNIQUE RECORDS] (964)

57 (201907* or 201908* or 201909* or 201910* or 201911* or 201912* or 2020*).dc. (2566829)

58 56 and 57 [EMBASE UNIQUE RECORDS - UPDATE PERIOD] (114)

59 52 not (53 or 56) [PSYCINFO RECORDS] (190)

60 (201907* or 201908* or 201909* or 201910* or 201911* or 201912* or 2020*).up. (34088633)

61 59 and 60 [PSYCINFO UNIQUE RECORDS - UPDATE PERIOD] (24)

62 55 or 58 or 61 (432) [TOTAL UNIQUE RECORDS – UPDATE PERIOD]

***************************

Cochrane Library

Search Name:

Date Run: 24/09/2020 19:47:08

Comment:

ID Search Hits

#1 (ecig* or (e next cig*)):ti,ab,kw 508

#2 (electr* next cigar*):ti,ab,kw 396

#3 "electronic nicotine":ti,ab,kw 172

#4 (electronic NEAR/3 vapori*):ti,ab,kw 12

#5 ("e-vape" or "e-vaped" or "e-vapes" or "e-vaping" or evape or evaped or evapes or evaping):ti,ab,kw 3

#6 (vape or vaper or vapers or vaping):ti,ab,kw 148

#7 {or #1-#6} 647

#8 [mh "Smoking Cessation"] 3997

#9 [mh "Tobacco Use Cessation"] 98

#10 [mh ^Smoking/TH] 494

#11 [mh "Tobacco Smoking"] 167

#12 [mh "Tobacco Use Disorder"/TH] 384

#13 ((smoking or smoker* or tobacco* or nicotine or cigar or cigars or cigarette* or cigarillo*) near/5 (abstain* or abstinen* or cease or ceased or ceases or cessation* or dehabituat* or desist* or discontinu* or end or ended or ending or ends or "give up" or "giving up" or "gives up" or "gave up" or halt* or quit* or stop*)):ti,ab,kw 12438

#14 {or #8-#13} 12530

#15 #7 and #14 with Cochrane Library publication date Between Jul 2019 and Dec 2020 103

DSR – 2

CENTRAL - 101

**Update**

**2024 Jan 25**

MEDLINE

Database: Embase Classic+Embase <1947 to 2024 January 24>, Ovid MEDLINE(R) ALL <1946 to January 24, 2024>, APA PsycInfo <1806 to January Week 3 2024>

Search Strategy:

--------------------------------------------------------------------------------

1 (e-cig$ or ecig$).mp. (22859)

2 electr$ cigar$.mp. (22083)

3 electronic nicotine.mp. (12337)

4 (electronic adj3 vapori*).mp. (81)

5 (e-vape$1 or e-vaping or evape$1 or evaping).mp. (57)

6 (vape or vaper or vapers or vaping).ti,ab. (9758)

7 or/1-6 [E-CIGARETTES] (33103)

8 Smoking Cessation/ (118875)

9 "Tobacco Use Cessation"/ (72078)

10 Smoking/th [therapy] (2389)

11 exp Tobacco Smoking/th [therapy] (574)

12 "Tobacco Use Disorder"/th [therapy] (3647)

13 ((smoking or smoker* or tobacco* or nicotine or cigar? or cigarette* or cigarillo?) adj5 (abstain* or abstinen* or cease or ceased or ceases or cessation* or dehabituat* or desist* or discontinu* or end or ended or ending or ends or "give up" or "giving up" or "gives up" or "gave up" or halt* or quit* or stop*)).tw,kf. (139963)

14 or/8-13 [SMOKING CESSATION] (176768)

15 7 and 14 [E-CIGARETTES - SMOKING CESSATION] (12424)

16 15 use medall [MEDLINE RECORDS] (4550)

17 (e-cig$ or ecig$).mp. (22859)

18 electr$ cigar$.mp. (22083)

19 electronic nicotine.mp. (12337)

20 (electronic adj3 vapori*).mp. (81)

21 (e-vape$1 or e-vaping or evape$1 or evaping).mp. (57)

22 (vape or vaper or vapers or vaping).ti,ab. (9758)

23 or/17-22 [E-CIGARETTES] (33103)

24 smoking cessation/ (118875)

25 smoking cessation program/ (3983)

26 exp smoking/th [Therapy] (2725)

27 tobacco dependence/dm, th [Disease Management, Therapy] (5088)

28 ((smoking or smoker* or tobacco* or nicotine or cigar? or cigarette* or cigarillo?) adj5 (abstain* or abstinen* or cease or ceased or ceases or cessation* or dehabituat* or desist* or discontinu* or end or ended or ending or ends or "give up" or "giving up" or "gives up" or "gave up" or halt* or quit* or stop*)).tw,kw. (138850)

29 or/24-28 [SMOKING CESSATION] (176858)

30 23 and 29 [E-CIGARETTES - SMOKING CESSATION] (12331)

31 30 use emczd [EMBASE RECORDS] (6091)

32 (e-cig$ or ecig$).mp. (22859)

33 electr$ cigar$.mp. (22083)

34 electronic nicotine.mp. (12337)

35 (electronic adj3 vapori*).mp. (81)

36 (e-vape$1 or e-vaping or evape$1 or evaping).mp. (57)

37 (vape or vaper or vapers or vaping).ti,ab. (9758)

38 or/32-37 [E-CIGARETTES] (33103)

39 smoking cessation/ (118875)

40 ((smoking or smoker* or tobacco* or nicotine or cigar? or cigarette* or cigarillo?) adj5 (abstain* or abstinen* or cease or ceased or ceases or cessation* or dehabituat* or desist* or discontinu* or end or ended or ending or ends or "give up" or "giving up" or "gives up" or "gave up" or halt* or quit* or stop*)).tw. (138564)

41 39 or 40 [SMOKING CESSATION] (174731)

42 38 and 41 [E-CIGARETTES - SMOKING CESSATION] (12175)

43 42 use psyh [PSYCINFO RECORDS] (1758)

44 16 or 31 or 43 [ALL DATABASES] (12399)

45 (2020* or 202011* or 202012* or 2021* or 2022* or 2023* or 2024*).dt. (6289553)

46 16 and 45 [MEDLINE RECORDS - UPDATE PERIOD] (2308)

47 (2020* or 2021* or 2022* or 2023* or 2024*).dc. (8198288)

48 31 and 47 [EMBASE RECORDS - UPDATE PERIOD] (3243)

49 (2020* or 2021* or 2022* or 2023* or 2024*).up. (47659410)

50 43 and 49 [PSYCINFO RECORDS - UPDATE PERIOD] (889)

51 46 or 48 or 50 [ALL DATABASES - UPDATE PERIOD] (6440)

52 limit 51 to yr="2023-current" (1582)

53 remove duplicates from 52 (904)

54 51 not 52 (4858)

55 remove duplicates from 54 (2910)

56 53 or 55 [**TOTAL UNIQUE RECORDS - UPDATE PERIOD**] (3814)

57 56 use medall [MEDLINE UNIQUE RECORDS - UPDATE PERIOD] (2266)

58 56 use emczd [EMBASE UNIQUE RECORDS - UPDATE PERIOD] (1316)

59 56 use psyh [PSYCINFO UNIQUE RECORDS - UPDATE PERIOD] (232)

***************************

Cochrane Library

Date Run: 25/01/2024 17:17:03

ID Search Hits

#1 (ecig* or (e next cig*)):ti,ab,kw 898

#2 (electr* next cigar*):ti,ab,kw 636

#3 "electronic nicotine":ti,ab,kw 464

#4 (electronic NEAR/3 vapori*):ti,ab,kw 13

#5 ("e-vape" or "e-vaped" or "e-vapes" or "e-vaping" or evape or evaped or evapes or evaping):ti,ab,kw 6

#6 (vape or vaper or vapers or vaping):ti,ab,kw 422

#7 {or #1-#6} 1246

#8 [mh "Smoking Cessation"] 5683

#9 [mh "Tobacco Use Cessation"] 161

#10 [mh ^Smoking/TH] 609

#11 [mh "Tobacco Smoking"] 508

#12 [mh "Tobacco Use Disorder"/TH] 479

#13 ((smoking or smoker* or tobacco* or nicotine or cigar or cigars or cigarette* or cigarillo*) near/5 (abstain* or abstinen* or cease or ceased or ceases or cessation* or dehabituat* or desist* or discontinu* or end or ended or ending or ends or "give up" or "giving up" or "gives up" or "gave up" or halt* or quit* or stop*)):ti,ab,kw 15182

#14 {or #8-#13} 15416

#15 #7 and #14 with Cochrane Library publication date Between Sep 2020 and Jan 2024 306

*CDSR – 9 reviews*

*CENTRAL – 296 trials*
