# Supplementary material for: Effectiveness of e-cigarettes as a stop smoking intervention in adults: a systematic review
Source: Syst Rev. 2024 Jun 29;13:168. doi: 10.1186/s13643-024-02572-7 (PMC11218295; doi:10.1186/s13643-024-02572-7)
Supplement: Supplementary file 3 — Additional file 3: Appendix 3. Eligibility criteria (PICOS). [file 13643_2024_2572_MOESM3_ESM.docx]

### **Appendix C - Eligibility criteria**

|  | **Inclusion** | **Exclusion** |
| --- | --- | --- |
| Population | Adults (≥18 years) who are current tobacco smokers (as defined by a given study) | - Studies exclusively in children/adolescents (i.e., under 18 years old) - Studies that involve interventions targeted to adults other than the tobacco smoker (e.g., partners, healthcare providers) - Pregnant individuals |
| Intervention | - Nicotine or non-nicotine containing e-cigarettes^a^ - Nicotine or non-nicotine containing e-cigarettes combined with other smoking cessation treatment (behaviour and/or pharmacological) | Studies exclusively examining short-term use of nicotine or non-nicotine containing e-cigarettes (i.e., < 1 week) |
| Comparator | KQ2a:   - Non-nicotine containing e-cigarettes (i.e., placebo e-cigarettes) - No intervention - Usual/standard care - Waitlist - Minimal intervention | Studies exclusively examining short-term use of nicotine or non-nicotine containing e-cigarettes (i.e., < 1 week) |
| Outcomes | **Benefits**  Critical   - Tobacco use abstinence (as defined in a given review)   Important   - Reduction in tobacco smoking frequency/quantity - Quality of life (using validated scales)   **Harms**  Important   - Adverse events (as defined in a given review) - Possible adverse outcomes: - Weight gain - Changes in emotional state (e.g., increases in anxiety, changes in mood, irritability) - Loss of social group^c^ |  |
| Timing of outcome assessment | For abstinence, reduction, and quality of life outcomes:  Minimum six months from quit date (if reported) or from initiation of intervention (if quit date not specified)  All other outcomes:   - Any point after initiation of intervention |  |
| Setting | Settings that could serve as the primary point of contact for individuals to receive smoking cessation advice, including:   - Family medicine clinics Walk-in clinics - Smoking cessation clinics - Urgent care facilities - Emergency departments - Public health units - Pharmacies - Dental offices - Behavioural health/substance use treatment facilities (ambulatory or outpatient) - Telehealth - Academic research settings | - Studies in settings not relevant to primary care including workplaces, schools, inpatient settings, and medical specialist settings - Studies that take place in countries “high”, “medium”, or “low” on the Human Development Index <http://hdr.undp.org/en/composite/HDI> |
| Study design | For benefits:   - Randomized controlled trials   For harms   - Randomized controlled trials - Non-randomized controlled trials - Comparative observational study designs (e.g., prospective and retrospective cohort studies, case-control studies) | For benefits:   - Non-randomized controlled trials - Observational study designs   For harms:   - Non-comparative studies - Cross-sectional studies   For benefits and harms:   - Systematic reviews - Case reports, case series - Editorials - Commentaries |
| Language | - English - French |  |
| Dates of publications | Date of last search of the review to January 2024 |  |
