# Supplementary material for: Effectiveness of e-cigarettes as a stop smoking intervention in adults: a systematic review
Source: Syst Rev. 2024 Jun 29;13:168. doi: 10.1186/s13643-024-02572-7 (PMC11218295; doi:10.1186/s13643-024-02572-7)
Supplement: Supplementary file 4 — Additional file 4: Appendix 4. List of excluded studies. [file 13643_2024_2572_MOESM4_ESM.docx]

# Appendix D. List of excluded study by reason (original search: January 2016 - July 3, 2019, September 2020, and January 2024)

## Published before 2016 (n=63)

1. Rudy, Susan F. and Durmowicz, Elizabeth L. Electronic nicotine delivery systems: overheating, fires and explosions. *Tobacco control* 2017; 26:10-18.
2. Brown, Jennifer, Brown, Brandon, Schwiebert, Peter, Ramakrisnan, Kalyanakrishnan, and McCarthy, Laine H. In adult smokers unwilling or unable to quit, does changing from tobacco cigarettes to electronic cigarettes decrease the incidence of negative health effects associated with smoking tobacco? A Clin-IQ. *Journal of patient-centered research and reviews* 2014; 1(2):99-101.
3. I'm trying to quit smoking. Should I try e-cigarettes? *The Johns Hopkins medical letter health after 50* 2011; 23(7):7-.
4. Page, F., Hamnett, N., Wearn, C., Hardwicke, J., and Moiemen, N. The acute effects of electronic cigarette smoking on the cutaneous circulation. *Journal of Plastic, Reconstructive and Aesthetic Surgery* 2016; 69(4):575-577.
5. WHO study group on tobacco product regulation: Report on the scientific basis of tobacco product regulation: Fifth report of a WHO study group. *WHO Tech Rep Ser.* 2015; 989:1-238.
6. Coleman, T., Chamberlain, C., Davey, M.-A., Cooper, S. E., and Leonardi-Bee, J. Pharmacological interventions for promoting smoking cessation during pregnancy. *Cochrane Database of Systematic Reviews* 2015; 2015(12):CD010078.
7. Buljubasich, D. E-Cigarette: A Modern Trojan Horse? *Archivos de bronconeumologia* 2015; 51(7):313-314.
8. Allam, J. S. and Ochoa, C. D. Pharmacological therapies in smoking cessation: An evidence-based update Topical Collection on Smoking Cessation. *Current Pulmonology Reports* 2015; 4(4):173-178.
9. Brose, L. S., Brown, J., Hitchman, S. C., and McNeill, A. Perceived relative harm of electronic cigarettes over time and impact on subsequent use. A survey with 1-year and 2-year follow-ups. *Drug and alcohol dependence* 2015; 157:106-111.
10. Wong, M. K., Barra, N. G., Alfaidy, N., Hardy, D. B., and Holloway, A. C. Adverse effects of perinatal nicotine exposure on reproductive outcomes. *Reproduction* 2015; 150(6):R185-R193.
11. Sklaroff, R. B. and Godshall, W. T. Electronic Cigarettes Are Efficacious. *Mayo Clinic Proceedings* 2015; 90(3):416-417.
12. Gratziou, C., Jimenez Ruiz, C. A., and Katsaounou, P. A. Smoking cessation using innovative techniques: Course report. *Breathe* 2015; 11(4):255-256.
13. Khan, J. E-cigarettes: A gateway to nicotine addiction? *Journal of Postgraduate Medical Institute* 2015; 29(4):213-214.
14. Glover, M. and McRobbie, H. Electronic cigarettes appealing quit aids for young adult smokers. *New Zealand Medical Journal* 2015; 128(1417):59-60.
15. Stubbs, B., Vancampfort, D., Bobes, J., De, Hert M., and Mitchell, A. J. How can we promote smoking cessation in people with schizophrenia in practice? A clinical overview. *Acta Psychiatrica Scandinavica* 2015; 132(2):122-130.
16. E-cigarettes could impact health in the developing world. *Cancer* 2015; 121(24):4275-.
17. Printz, C. AACR, ASCO issue statement on E-cigarette regulation. *Cancer* 2015; 121(11):1723-.
18. Printz, C. E-cigarette advertising linked to increased tobacco craving. *Cancer* 2015; 121(15):2479-.
19. Russi, E. W. E-cigarettes: Promise or peril? *Swiss medical weekly* 2015; 145:w14187-.
20. King, A. C., Smith, L. J., McNamara, P. J., Matthews, A. K., and Fridberg, D. J. Passive exposure to electronic cigarette (e-cigarette) use increases desire for combustible and e-cigarettes in young adult smokers. *Tobacco control* 2015; 24(5):501-504.
21. Alawsi, F., Nour, R., and Prabhu, S. Are e-cigarettes a gateway to smoking or a pathway to quitting? *British dental journal* 2015; 219(3):111-115.
22. Staff gain support on e-cigarette use. *Nursing standard* 2015; 29(52):10-.
23. D'Ruiz, C. D., Graff, D. W., and Yan, X. S. Nicotine delivery, tolerability and reduction of smoking urge in smokers following short-term use of one brand of electronic cigarettes. *BMC public health* 2015; 15:991-.
24. Levitz, S. Can electronic cigarettes assist patients with smoking cessation? No. *Canadian family physician Medecin de famille canadien* 2015; 61(6):500-505.
25. Glasser, A. M., Cobb, C. O., Teplitskaya, L., Ganz, O., Katz, L., Rose, S. W., Feirman, S., and Villanti, A. C. Electronic nicotine delivery devices, and their impact on health and patterns of tobacco use: a systematic review protocol. *BMJ open* 2015; 5(4):e007688-.
26. Bonevski, B., Guillaumier, A., and Twyman, L. Electronic nicotine devices considered through an equity lens. *Addiction* 2015; 110(7):1069-1070.
27. Chapman, S. and Daube, M. Ethical imperatives assuming ENDS effectiveness and safety are fragile. *Addiction* 2015; 110(7):1068-1069.
28. Hall, W., Gartner, C., and Forlini, C. Nuances in the ethical regulation of electronic nicotine delivery systems. *Addiction (Abingdon, England)* 2015; 110(7):1074-1075.
29. Dawkins, L., Kimber, C., Puwanesarasa, Y., and Soar, K. First- versus second-generation electronic cigarettes: predictors of choice and effects on urge to smoke and withdrawal symptoms. *Addiction* 2015; 110(4):669-677.
30. Douglas, H., Hall, W., and Gartner, C. E-cigarettes and the law in Australia. *Australian family physician* 2015; 44(6):415-418.
31. Weidman-Evans, E., Black, W. C., and Fort, A. My patient wants to try e-cigarettes to quit smoking. What should I say? *JAAPA* 2015; 28(8):22-24.
32. Lechner, W. V., Meier, E., Wiener, J. L., Grant, D. M., Gilmore, J., Judah, M. R., Mills, A. C., and Wagener, T. L. The comparative efficacy of first- versus second-generation electronic cigarettes in reducing symptoms of nicotine withdrawal. *Addiction* 2015; 110(5):862-867.
33. Antolin, V. M. and Barkley, T. W. Electronic cigarettes: What nurses need to know. *Nursing* 2015; 45(11):60-64.
34. Kirby, T. E-cigarettes declared 95% less harmful than tobacco by UK health body. *The Lancet Respiratory medicine* 2015; 3(10):750-751.
35. Gualano, M. R., Passi, S., Bert, F., La, Torre G., Scaioli, G., and Siliquini, R. Electronic cigarettes: assessing the efficacy and the adverse effects through a systematic review of published studies. *Journal of public health* 2015; 37(3):488-497.
36. Collier, R. E-cigarette data don't end debate. *CMAJ* 2015; 187(10):E291.
37. Fillon, M. Policymakers work to develop e-cigarette guidelines and restrictions. *Journal of the National Cancer Institute* 2015; 107(7).
38. Janceya, J., Binns, C., Smith, J. A., Maycock, B., and Howat, P. The rise of e-cigarettes: implications for health promotion. *Health promotion journal of Australia* 2015; 26(2):79-82.
39. Summaries for Patients. Behavioral and Pharmacologic Treatments to Help Adults Quit Smoking: U.S. Preventive Services Task Force Recommendation Statement. *Annals of internal medicine* 2015; 163(8):I-40.
40. Shantakumari, N., Muttappallymyalil, J., John, L. J., and Sreedharan, J. Cigarette Alternatives: Are they Safe? *Asian Pacific journal of cancer prevention* 2015; 16(8):3587-3590.
41. McRobbie, H., Bullen, C., Hartmann-Boyce, J., and Hajek, P. Electronic cigarettes for smoking cessation and reduction. *Cochrane Database of Systematic Reviews* 2014; 2014(12):CD010216.
42. Jimenez Ruiz, C. A., Solano, Reina S., de Granda Orive, J. I., Signes-Costa, Minaya J., de Higes, Martinez E., Riesco Miranda, J. A., Altet, Gomez N., Lorza Blasco, J. J., Barrueco, Ferrero M., and de Lucas, Ramos P. The Electronic Cigarette. Official Statement of the Spanish Society of Pneumology and Thoracic Surgery (SEPAR) on the Efficacy, Safety and Regulation of Electronic Cigarettes. *Archivos de bronconeumologia* 2014; 50(8):362-367.
43. McGraw, D. Current and future trends in electronic cigarette use. *International Journal of Psychiatry in Medicine* 2014; 48(4):325-332.
44. Schluger, N. W. The electronic cigarette: A knight in shining armour or a Trojan horse? *Psychiatric Bulletin* 2014; 38(5):201-203.
45. Printz, C. ASCO, AACR issue joint response to proposed FDA tobacco rule. *Cancer* 2014; 120(24):3848-3849.
46. Cummings, K. M., Dresler, C. M., Field, J. K., Fox, J., Gritz, E. R., Hanna, N. H., Ikeda, N., Jassem, J., Mulshine, J. L., Peters, M. J., Yamaguchi, N. H., Warren, G., and Zhou, C. E-cigarettes and cancer patients. *Journal of Thoracic Oncology* 2014; 9(4):438-441.
47. Chapman, S. E-cigarettes: The best and the worst case scenarios for public health - An essay by Simon Chapman. *BMJ (Online)* 2014; 349:g5512-.
48. O'Moore, E., Davies, K., and Mulholland, I. A partnership approach to implementing smoke-free prisons. *BMJ (Online)* 2014; 349:g5443-.
49. Kmietowicz, Z. E-cigarettes latest: Users on the up but rules tighten. *BMJ (Online)* 2014; 349:g6444-.
50. Veeraraghavan, S. Tobacco control: Up in E-smoke? *Cancer* 2014; 120(22):3430-3432.
51. Hajek, P., Etter, J.-F., Benowitz, N., Eissenberg, T., and McRobbie, H. Electronic cigarettes: review of use, content, safety, effects on smokers and potential for harm and benefit. *Addiction* 2014; 109(11):1801-1810.
52. McNeill, A., Etter, J.-F., Farsalinos, K., Hajek, P., le, Houezec J., and McRobbie, H. A critique of a World Health Organization-commissioned report and associated paper on electronic cigarettes. *Addiction* 2014; 109(12):2128-2134.
53. Callahan-Lyon, P. Electronic cigarettes: human health effects. *Tobacco control* 2014; 23(Supplement 2).
54. Peruga, A. and Fleck, F. Countries vindicate cautious stance on e-cigarettes. *Bulletin of the World Health Organization* 2014; 92(12):856-857.
55. Caponnetto, P., Russo, C., Bruno, C. M., Alamo, A., Amaradio, M. D., and Polosa, R. Electronic cigarette: A possible substitute for cigarette dependence. *Monaldi Archives for Chest Disease - Cardiac Series* 2013; 79(1):12-19.
56. Nardini, S. and Pacifici, R. E-cigarettes, smokers and health policies. *Monaldi Archives for Chest Disease - Cardiac Series* 2013; 79(1):6-7.
57. Balbi, B. and Spanevello, A. The fight against tobacco. *Monaldi Archives for Chest Disease - Cardiac Series* 2013; 79(1):5-.
58. Donzelli, Alberto E-cigarettes may impair ability to quit, but other explanations are possible. *American journal of public health* 2015; 105(11):e1.
59. Eversman, Michael H. Tobacco harm reduction: An emerging health issue for social work. *Journal of Social Work Practice in the Addictions* 2015; 15(4):341-351.
60. Wang, M. P., Li, W. H. C., Jiang, N., Chu, L. Y., Kwong, A., Lai, V., and Lam, T. H. E-cigarette awareness, perceptions and use among community-recruited smokers in Hong Kong. *PloS one* 2015; 10(10).
61. Papaseit, E., Perez-Mana, C., Mateus, J. A., Menoyo, E., Perez, M., Martin, S., Gibert, C., Peteiro, R., Garcia-Algar, O., and Farre, M. Pharmacodynamics of nicotine from secondgeneration electronic cigarette. *Basic and clinical pharmacology and toxicology.* 2014; 115:13-.
62. Perkins, K. A., Karelitz, J. L., and Michael, V. C. Reinforcement enhancing effects of acute nicotine via electronic cigarettes. *Drug and alcohol dependence* 2015; 153:104‐108-.
63. Wise, J. E-cigarettes as good as patches in helping to reduce smoking, randomised controlled trial concludes . 2013

## Published in language other than English (n=53)

1. Signes-Costa, Jaime, de Granda-Orive, Jose Ignacio, Ramos Pinedo, Angela, Camarasa Escrig, Ana, de Higes Martinez, Eva, Rabade Castedo, Carlos, Cabrera Cesar, Eva, and Jimenez-Ruiz, Carlos A. Official Statement of the Spanish Society of Pulmonology and Thoracic Surgery (SEPAR) on Electronic Cigarettes and IQOS. *Archivos de bronconeumologia* 2019.
2. Reynales-Shigematsu, Luz Myriam, Barrientos-Gutierrez, Inti, Zavala-Arciniega, Luis, and Arillo-Santillan, Edna [New tobacco products, a threat for tobacco control and public health of Mexico]. *Salud publica de Mexico* 2018; 60(5):598-604.
3. Zeng, D. C., Lu, L. M., Zhao, X. S., Yang, S. Y., Jiang, Y., Tong, Z., and Feng, Y. [Analysis of electronic cigarettes safety]. *Zhonghua jiehe he huxi zazhi = Chinese journal of tuberculosis and respiratory diseases* 2019; 42(5):393-397.
4. Kroger, Christoph B., Ofner, Sarah, and Piontek, Daniela [Use of E-cigarettes as an additional tool in a smoking cessation group intervention: Results after 12 months]. *Bundesgesundheitsblatt, Gesundheitsforschung, Gesundheitsschutz* 2018; 61(1):32-39.
5. Camporro, Fernando Astur, Gutierrez Magaldi, Ignacio, and Bulacio, Exequiel [Electronic Cigarette: not all that glitters is gold]. *Revista de la Facultad de Ciencias Medicas (Cordoba, Argentina)* 2017; 74(3):271-276.
6. Comissao de Combate ao Tabagismo da Associacao Medica Brasileira AMB warns against the use of electronic nicotine delivery devices: Electronic and heated cigarettes. *Revista da Associacao Medica Brasileira (1992)* 2017; 63(10):825-826.
7. Camporro, Fernando A. [Electronic nicotine release systems, the healthy evolution of smoking]. *Medicina* 2017; 77(3):250-251.
8. Chavannes, N. H., Meijer, E., Wind, L., van de Graaf, R. C., Rietbergen, C., and Croes, E. A. [Herziene richtlijn 'Behandeling van tabaksverslaving en stoppen met roken ondersteuning'.] Dutch. *Nederlands tijdschrift voor geneeskunde* 2017; 161:D1394-.
9. van de Graaf, R. C. and van Schayck, O. C. P. [Helping people to give up smoking; efficacy and safety of smoking cessation interventions]. *Nederlands tijdschrift voor geneeskunde* 2017; 161:D1131-.
10. Tarrazo, Marina, Perez-Rios, Monica, Santiago-Perez, Maria I., Malvar, Alberto, Suanzes, Jorge, and Hervada, Xurxo [Changes in tobacco consumption: boom of roll-your-own cigarettes and emergence of e-cigarettes]. *Gaceta sanitaria* 2017; 31(3):204-209.
11. Schaller, K. and Mons, U. [E-Cigarettes: Assessment of Health Effects and Potential Benefits for Smokers]. *Pneumologie* 2018; 72(6):458-472.
12. Aleknaite, Aiste, Andrijauskaite, Monika, Latauskiene, Juste, and Andrejevaite, Viktorija [Elektronines cigaretes: naujas budas mesti rukyti ar nauja gresme?] Lithuanian. *Acta medica Lituanica* 2016; 23(1):43-53.
13. Cselko, Zsuzsa and Penzes, Melinda [Summary of the existing knowledge about electronic cigarettes]. *Orvosi hetilap* 2016; 157(25):979-986.
14. Lenzen-Schulte, M. E-cigarettes: At full steam towards smoking cessation. *Deutsches Arzteblatt international* 2019; 116(7):A314-.
15. Jungmayr, P. More steam than effect? The success of e-cigarettes in smoking cessation is controversial. *Deutsche Apotheker Zeitung* 2019; 159(8).
16. Schneider, S. and Schilling, L. Are e-cigarettes an alternative for pregnant smokers? *Atemwegs- und Lungenkrankheiten* 2019; 45(5):232-238.
17. Reinhardt, C. and Andreas, S. New evidence on the use of e-cigarettes in smoking cessation from tobacco use. *Pneumologe* 2019.
18. Thirion-Romero, I., Perez-Padilla, R., Zabert, G., and Barrientos-Gutierrez, I. Respiratory impact of electronic cigarettes and low-risk tobacco. *Revista de Investigacion Clinica* 2019; 71(1):17-27.
19. Holzgreve, H. Smoking cessation: E-cigarette remains dubious. *MMW-Fortschritte der Medizin* 2019; 161(6):32-.
20. Bauer, C. M., Kreuter, M., and Herth, F. Review: The e-cigarette in smoking cessation. *Tumor Diagnostik und Therapie* 2016; 37(2):69-72.
21. Kastaun, S., Becker, S., and Kotz, D. How effective is the electronic (e-)cigarette for smoking cessation? *Gynakologische Praxis* 2018; 43(4):703-713.
22. Paumgartten, F. Heat-not-burn and electronic cigarettes: Truths and untruths about harm reduction. *Revista da Associacao Medica Brasileira* 2018; 64(2):104-105.
23. Schaller, K. and Mons, U. Tobacco smoking. *Pneumologe* 2018; 15(4):244-253.
24. Lee, C., Kim, S., and Cheong, Y. S. Issues of new types of tobacco (e-cigarette and heat-not-burn tobacco): From the perspective of 'tobacco harm reduction'. *Journal of the Korean Medical Association* 2018; 61(3):181-190.
25. Tottenborg, S. S., Holm, A. L., Wibholm, N. C., and Lange, P. Health consequences of smoking electronic cigarettes are poorly described. *Ugeskrift for laeger* 2014; 176(36).
26. Broekhuizen, L. Electronic cigarette for smoking cessation. *Huisarts en Wetenschap* 2017; 60(10):535-.
27. Jankowski, P., Kawecka-Jaszcz, K., Kopec, G., Podolec, J., Pajak, A., Sarnecka, A., Zdrojewski, T., Czarnecka, D., Malecki, M., Nowicka, G., Czlonkowska, A., Niewada, M., Stanczyk, J., Undas, A., Windak, A., Cedzynska, M., Zatonski, W., and Podolec, P. Polish forum for prevention guidelines on smoking: Update 2017. *Kardiologia Polska* 2017; 75(4):409-411.
28. Worth, H. Effects of cigarette smoking on pulmonary infections. *Atemwegs- und Lungenkrankheiten* 2017; 43(6):263-266.
29. Koneczny, N. and Sonnichsen, A. E-cigarettes for smoking cessation: Harm or benefit? *Zeitschrift fur Allgemeinmedizin* 2017; 93(2):51-53.
30. Zdrojewicz, Z., Pypno, D., Bugaj, B., and Burzynska, A. Electronic cigarettes: Health impact, nicotine replacement therapy, regulations. *Pediatria i Medycyna Rodzinna* 2017; 13(1):63-71.
31. Batra, A., Petersen, K. U., Hoch, E., Andreas, S., Bartsch, G., Gohlke, H., Jahne, A., Kroger, C., Lindinger, P., Muhlig, S., Neumann, T., Potschke-Langer, M., Ratje, U., Ruther, T., Schweizer, C., Thurauf, N., Ulbricht, S., and Mann, K. S3 guideline "screening, diagnostics, and treatment of harmful and addictive tobacco use": Short version. *Sucht* 2016; 62(3):139-152.
32. Finoulst, M., Vankrunkelsven, P., and Hendrickx, S. The role of electronic cigarettes in smoking cessation. *Tijdschrift voor Geneeskunde* 2016; 72(5):360-363.
33. Zaga, V. Is electronic cigarette always not recommended? No. *Rassegna di Patologia dell'Apparato Respiratorio* 2016; 31(6):338-342.
34. Lobaszewski, J. and Didkowska, J. Electronic cigarettes: A new challenge for Polish public health. *Polskie Archiwum Medycyny Wewnetrznej* 2016; 126(11):905-906.
35. Rogulj, T. and Zuntar, I. What do we know about safety of e-cigarettes? *Farmaceutski Glasnik* 2016; 72(6):395-406.
36. Gohlke, H. E-cigarettes hype: Magic cure or substitute drug? *Kardiologe* 2016; 10(4):236-241.
37. Willers, S. E-cigarettes should be considered for therapy-resistant smoke-induced COPD. *Lakartidningen* 2015; 112:#start page#-.
38. Theofilos, D., Marketos, C., Bisirtzoglou, D., Sakelaropoulou, A., Michalaki, V., Zetos, A., and Politis, G. The role of smoking cessation in lung cancer patients. *Pneumon* 2015; 28(4):327-339.
39. Nowak, D., Gohlke, H., Hering, T., Herth, F. J., Jany, B., Raupach, T., Welte, T., and Loddenkemper, R. Position Paper of the German Respiratory Society (DGP) on Electronic Cigarettes (E-Cigarettes) in Cooperation with the following Scientific Societies and Organisations: BVKJ, BdP, DGAUM, DGG, DGIM, DGK, DKG, DGSMP, GPP. *Gesundheitswesen (Bundesverband der Arzte des Offentlichen Gesundheitsdienstes (Germany))* 2015; 77(7):508-511.
40. Polosa, R. Vaping: a new strategy to prevent smoking-related diseases? *Epidemiologia e prevenzione* 2014; 38(3-4):264-267.
41. Knorst, M. M., Benedetto, I. G., Hoffmeister, M. C., and Gazzana, M. B. The electronic cigarette: the new cigarette of the 21st century? *Jornal brasileiro de pneumologia* 2014; 40(5):564-572.
42. Bleckwenn, M. [Smoker cessation with E-cigarettes - clinical evaluation]. [Review] [German]. 2019
43. Boleo-Tome, J. P.; Pamplona, P.; Rosa, P.; Cordeiro, C. R. [The Doctor, the Smoking Patient and the Challenge of Electronic Cigarettes]. [Portuguese]. 2019
44. Hering, T. [Smoking should be classified as a disease]. [German]. 2019
45. Hering, T. [Electronic cigarette in Patients with COPD or Asthma: Curse or Blessing?]. [Review] [German]. 2019
46. Hering, T. Smoking cessation in COPD is prevention and therapy - which effects can be expected? [German]. 2019
47. Hohmann-Jeddi, C. Smoking cessation: E-cigarettes help in quitting. [German]. 2019
48. Lenzen-Schulte, M. E-cigarettes: At full steam towards smoking cessation. [German] . 2019
49. Pommer, P. 2019
50. Rabade-Castedo, C.; de Granda-Orive, J. I.; Gonzalez-Barcala, F. J. Incremento de la prevalencia del tabaquismo:?causas y actuacion? . 2019
51. Steurer, J. Smoking cessation: According to a study, e-cigarettes are more effective than other nicotine replacement preparations. [German]. 2019
52. Tijdink, J.; Van Noorden, M. Are e-cigarettes effective in the treatment of nicotine dependence?. [Dutch]. 2019
53. Bar AL. Usage de la cigarette electronique et combustible. 2020;16(698):1272.

## Full text not available (n=24)

1. Kousta, Stavroula. E-cigarettes for smoking cessation. *Nature human behaviour* 2019; 3(4):322-.
2. Loewen, Jill M. and Relich, Erin E. Electronic Nicotine Delivery Systems: Current trends and patient education opportunities for dental hygienists. *Journal of dental hygiene* 2019; 93(1):43-51.
3. Bamidis, Panagiotis D., Paraskevopoulos, Evangelos, Konstantinidis, Evdokimos, Spachos, Dimitris, and Billis, Antonis Multimodal e-Health Services for Smoking Cessation and Public Health: The SmokeFreeBrain Project Approach. *Studies in health technology and informatics* 2017; 245:5-9.
4. Jacot Sadowski, Isabelle, Humair, Jean Paul, and Cornuz, Jacques. [Vaping (electronic cigarette): how to advise smokers in 2017?]. *Revue medicale suisse* 2017; 13(566):1181-1185.
5. Daily vaping increases heart attack risk. *Clinical Pharmacist* 2018; 10(11).
6. Hamee, R. H. Human health effects of electronic cigarettes: A review. *Indian Journal of Public Health Research and Development* 2018; 9(8):1387-1391.
7. Urban, T. and Hureaux, J. Electronic-cigarette. A smoking cessation tool? *Revue des Maladies Respiratoires Actualites* 2017; 9(2):100-105.
8. To vape or not to vape. *Australian Journal of Pharmacy* 2017; 98(1162):16-18.
9. Bullen, C., Lopez-Nunez, C., and Knight-West, O. E-cigarettes in smoking cessation: A harm reduction perspective. *Clinical Pharmacist* 2016; 8(4).
10. Glascoe, A. L. and Brown, R. S. A Review of E-Cigarettes and Related Health Issues. *Dentistry today* 2015; 34(9):46-47.
11. Are e-cigarettes really safe? *Pharmaceutical Journal* 2015; 295(7880):226-227.
12. E-cigarettes and smoking cessation. Similar efficacy to other nicotine delivery devices, but many uncertainties. *Prescrire international* 2015; 24(165):271-276.
13. Drobes, David J. Pharmacological treatment of tobacco use disorder. 2019:609-629.
14. Symes, Yael Rose. Smoking cessation in cancer survivors: Exploring psychosocial wellbeing, beliefs about smoking, and e-cigarette use. *Dissertation Abstracts International: Section B: The Sciences and Engineering* 2018; 79(7-B(E)).
15. Brady, Benjamin R., Crane, Tracy E., O'Connor, Patrick A., Nair, Uma S., and Yuan, Nicole P. Electronic cigarette use and tobacco cessation in a state-based quitline. *Journal of smoking cessation* 2019.
16. Brady, Benjamin Robert. Smoking cessation strategies and policy recommendations: Harm reduction, electronic cigarettes, and abstinence goal setting. *Dissertation Abstracts International: Section B: The Sciences and Engineering* 2018; 79(12-B(E)).
17. Shen, Chen. Smoking cessation strategies and policy recommendations: Harm reduction, electronic cigarettes, and abstinence goal setting. *Dissertation Abstracts International Section A: Humanities and Social Sciences* 2018; 79(12-A(E)).
18. Cahn, Zachary. Informing E-cigarette policy: Population effects and tobacco industry incentives. *Dissertation Abstracts International: Section B: The Sciences and Engineering* 2017; 78(3-B(E)).
19. EUCTR2017-003188-36-FI Keski-ikäisten pitkään tupakoineiden tupakasta vieroitus sähkötupakalla. *Http://www.who.int/trialsearch/trial2.aspx? Trialid=euctr2017-003188-36-fi*
20. Chaumont, M.; El Channan, M.; Bernard, A.; Lesage, A.; Deprez, G.; Van Muylem, A.; Schaefer, T.; Faoro, V.; Van De Borne, P. Short-term high wattage e-cigarette cessation improves cardiorespiratory outcomes in regular users: A randomized crossover trial . 2019
21. Ioakeimidis, N.; Vlachopoulos, C.; Georgakopoulos, C.; Dima, I.; Solomou, E.; Gardikioti, V.; Oikonomou, E.; Tousoulis, D. Two-year therapeutic effectiveness of pharmacotherapy versus electronic cigarettes for smoking cessation: A single-center experience . 2019
22. Zhuikova, E.; Durrant, P.; Macauley, E.; Goss, H.; Goldsmith, N.; Ioannides, C.; Marczylo, T.; Bailey, A. Monitoring the transition from cigarette smoking to electronic cigarette use: Nicotine intake, psychometric, and clinical outcomes . 2019
23. Skelton E, Robinson M, Lum A, Dunlop A, Baker A, Gartner C, et al. A pilot study of abrupt verse gradual smoking cessation in combination with electronic nicotine devices for smokers receiving alcohol and other drug treatment. Asia-Pacific Journal of Clinical Oncology. 2020;16(SUPPL 6):37–8.
24. Ikonomidis I, Katogiannis K, Kostelli G, Kourea K, Kyriakou E, Kypraiou A, et al. Effects of electronic cigarette on platelet and vascular function after one month of use. European Heart Journal. 2020;41(SUPPL 2):2359.

## Study design (n=884)

### Narrative/Literature Review (n=141)

1. Scott, James G., Jhetam, Sarah, Chen, Renee, and Daglish, Mark Should psychiatrists support the availability of nicotine e-cigarettes in Australia? *Australasian psychiatry* 2019.
2. Versella, Mark V. and Leyro, Teresa M. Electronic cigarettes and nicotine harm-reduction. *Current opinion in psychology* 2019; 30:29-34.
3. Indian Council of Medical Research White Paper on Electronic Nicotine Delivery System. *The Indian journal of medical research* 2019.
4. Underner, M., Perriot, J., Brousse, G., de Chazeron, I., Schmitt, A., Peiffer, G., Harika-Germaneau, G., and Jaafari, N. [Stopping and reducing smoking in patients with schizophrenia]. *L'Encephale* 2019.
5. Middlekauff, Holly R. Cardiovascular impact of electronic-cigarette use. *Trends in cardiovascular medicine* 2019.
6. Kitzen, Jan M., McConaha, Jamie L., Bookser, Megan L., Pergolizzi, Joseph V. J., Taylor, Robert Jr, and Raffa, Robert B. e-Cigarettes for smoking cessation: Do they deliver? *Journal of clinical pharmacy and therapeutics* 2019.
7. Sultan, Ahmed S., Jessri, Maryam, and Farah, Camile S. Electronic nicotine delivery systems: Oral health implications and oral cancer risk. *Journal of oral pathology & medicine* 2018.
8. Golbidi, Saeid, Edvinsson, Lars, and Laher, Ismail Smoking and Endothelial Dysfunction. *Current vascular pharmacology* 2018.
9. Warner, Kenneth E. How to Think - Not Feel - about Tobacco Harm Reduction. *Nicotine & tobacco research* 2018.
10. Blaha, Michael J. and Ratchford, Elizabeth V. Electronic cigarettes. *Vascular medicine (London, England)* 2019; 24(3):267-269.
11. Ioakeimidis, Nikolaos, Vlachopoulos, Charalambos, Katsi, Vasiliki, and Tousoulis, Dimitrios Smoking cessation strategies in pregnancy: Current concepts and controversies. *Hellenic journal of cardiology* 2019; 60(1):11-15.
12. Jenssen, Brian P. and Wilson, Karen M. What is new in electronic-cigarettes research? *Current opinion in pediatrics* 2019; 31(2):262-266.
13. Jancey, Jonine, Maycock, Bruce, McCausland, Kahlia, and Howat, Peter E-Cigarettes: Implications for Health Promotion in the Asian Pacific Region. *Asia-Pacific journal of public health* 2018; 30(4):321-327.
14. Henningfield, Jack E., Higgins, Stephen T., and Villanti, Andrea C. Are we guilty of errors of omission on the potential role of electronic nicotine delivery systems as less harmful substitutes for combusted tobacco use? *Preventive medicine* 2018; 117:83-87.
15. Smith, Tracy T., Hatsukami, Dorothy K., Benowitz, Neal L., Colby, Suzanne M., McClernon, F. Joseph, Strasser, Andrew A., Tidey, Jennifer W., White, Cassidy M., and Donny, Eric C. Whether to push or pull? Nicotine reduction and non-combusted alternatives - Two strategies for reducing smoking and improving public health. *Preventive medicine* 2018; 117:8-14.
16. Giles, Michelle L., Gartner, Coral, and Boyd, Mark A. Smoking and HIV: what are the risks and what harm reduction strategies do we have at our disposal? *AIDS research and therapy* 2018; 15(1):26-.
17. Bold, Krysten W., Krishnan-Sarin, Suchitra, and Stoney, Catherine M. E-cigarette use as a potential cardiovascular disease risk behavior. *The American psychologist* 2018; 73(8):955-967.
18. Sood, Amika K., Kesic, Matthew J., and Hernandez, Michelle L. Electronic cigarettes: One size does not fit all. *The Journal of allergy and clinical immunology* 2018; 141(6):1973-1982.
19. Fairchild, Amy Lauren, Bayer, Ronald, and Lee, Ju Sung The E-Cigarette Debate: What Counts as Evidence? *American journal of public health* 2019; 109(7):1000-1006.
20. Kaur, Jagdish and Rinkoo, Arvind Vashishta Getting real with the upcoming challenge of electronic nicotine delivery systems: The way forward for the South-East Asia region. *Indian journal of public health* 2017; 61(Suppl 1):S7-S11.
21. Kar, Murat, Emre, Ismet Emrah, Bayar Muluk, Nuray, and Cingi, Cemal Effect of Electronic Cigarettes on the Inner Mucosa of the Craniofacial Region. *The Journal of craniofacial surgery* 2019; 30(3):e235-e238.
22. Chaffee, Benjamin W. Electronic Cigarettes: Trends, Health Effects and Advising Patients Amid Uncertainty. *Journal of the California Dental Association* 2019; 47(2):85-92.
23. Darville, Audrey and Hahn, Ellen J. E-cigarettes and Atherosclerotic Cardiovascular Disease: What Clinicians and Researchers Need to Know. *Current atherosclerosis reports* 2019; 21(5):15-.
24. Eltorai, Adam Em, Choi, Ariel R., and Eltorai, Ashley Szabo Impact of Electronic Cigarettes on Various Organ Systems. *Respiratory care* 2019; 64(3):328-336.
25. Essenmacher, Carol, Naegle, Madeline, Baird, Carolyn, Vest, Bridgette, Spielmann, Rene, Smith-East, Marie, and Powers, Leigh Electronic Nicotine Delivery Systems (ENDS): What Nurses Need to Know. *Journal of the American Psychiatric Nurses Association* 2018; 24(2):145-152.
26. Bals, Robert, Boyd, Jeanette, Esposito, Susanna, Foronjy, Robert, Hiemstra, Pieter S., Jimenez-Ruiz, Carlos A., Katsaounou, Paraskevi, Lindberg, Anne, Metz, Carlos, Schober, Wolfgang, Spira, Avrum, and Blasi, Francesco Electronic cigarettes: a task force report from the European Respiratory Society. *The European respiratory journal* 2019; 53(2).
27. Livingston, Catherine J., Freeman, Randall J., Costales, Victoria C., Westhoff, John L., Caplan, Lee S., Sherin, Kevin M., and Niebuhr, David W. Electronic Nicotine Delivery Systems or E-cigarettes: American College of Preventive Medicine's Practice Statement. *American journal of preventive medicine* 2019; 56(1):167-178.
28. Brady, Benjamin R., De La Rosa, Jennifer S., Nair, Uma S., and Leischow, Scott J. Electronic Cigarette Policy Recommendations: A Scoping Review. *American journal of health behavior* 2019; 43(1):88-104.
29. Qasim, Hanan, Karim, Zubair A., Rivera, Jose O., Khasawneh, Fadi T., and Alshbool, Fatima Z. Impact of Electronic Cigarettes on the Cardiovascular System. *Journal of the American Heart Association* 2017; 6(9).
30. Glantz, Stanton A. and Bareham, David W. E-Cigarettes: Use, Effects on Smoking, Risks, and Policy Implications. *Annual review of public health* 2018; 39:215-235.
31. Huang, Shu Jie, Xu, Yan Ming, and Lau, Andy T. Y. Electronic cigarette: A recent update of its toxic effects on humans. *Journal of cellular physiology* 2018; 233(6):4466-4478.
32. Underner, M., Perriot, J., Peiffer, G., Harika-Germaneau, G., and Jaafari, N. [Why stopping smoking is difficult in patients suffering from schizophrenia? How better to take care of them?]. *Revue medicale de Liege* 2019; 74(1):23-27.
33. Lohler, Jan and Wollenberg, Barbara Are electronic cigarettes a healthier alternative to conventional tobacco smoking? *European archives of oto-rhino-laryngology* 2019; 276(1):17-25.
34. Franks, Andrea S., Sando, Karen, and McBane, Sarah Do Electronic Cigarettes Have a Role in Tobacco Cessation? *Pharmacotherapy* 2018; 38(5):555-568.
35. Bartsch, P., Delvaux, M., Englebert, E., Beaupain, M. H., and Louis, R. [E-cigarette and smoking cessation: current situation in Belgium]. *Revue medicale de Liege* 2017; 72(1):14-19.
36. Deville, M. and Charlier, C. [Electronic cigarette: state of the science about toxicological aspects]. *Revue medicale de Liege* 2017; 72(1):20-24.
37. Romijnders, Kim A. G. J., van Osch, Liesbeth, de Vries, Hein, and Talhout, Reinskje Perceptions and Reasons Regarding E-Cigarette Use among Users and Non-Users: A Narrative Literature Review. *International journal of environmental research and public health* 2018; 15(6).
38. Fearon, Ian M., Eldridge, Alison C., Gale, Nathan, McEwan, Mike, Stiles, Mitchell F., and Round, Elaine K. Nicotine pharmacokinetics of electronic cigarettes: A review of the literature. *Regulatory toxicology and pharmacology* 2018; 100:25-34.
39. Farsalinos, Konstantinos Electronic cigarettes: an aid in smoking cessation, or a new health hazard? *Therapeutic advances in respiratory disease* 2018; 12:1753465817744960-.
40. Chun, Lauren F., Moazed, Farzad, Calfee, Carolyn S., Matthay, Michael A., and Gotts, Jeffrey E. Pulmonary toxicity of e-cigarettes. *American journal of physiology Lung cellular and molecular physiology* 2017; 313(2):L193-L206.
41. Camenga, Deepa R. and Tindle, Hilary A. Weighing the Risks and Benefits of Electronic Cigarette Use in High-Risk Populations. *The Medical clinics of North America* 2018; 102(4):765-779.
42. Kaisar, Mohammad Abul, Prasad, Shikha, Liles, Tylor, and Cucullo, Luca A decade of e-cigarettes: Limited research & unresolved safety concerns. *Toxicology* 2016; 365:67-75.
43. Whittington, Julie R., Simmons, Pamela M., Phillips, Amy M., Gammill, Sarah K., Cen, Ruiqi, Magann, Everett F., and Cardenas, Victor M. The Use of Electronic Cigarettes in Pregnancy: A Review of the Literature. *Obstetrical & gynecological survey* 2018; 73(9):544-549.
44. Mendelsohn, Colin P. Electronic cigarettes in physician practice. *Internal medicine journal* 2018; 48(4):391-396.
45. Zare, Samane, Nemati, Mehdi, and Zheng, Yuqing A systematic review of consumer preference for e-cigarette attributes: Flavor, nicotine strength, and type. *PloS one* 2018; 13(3):e0194145-.
46. Rehan, Harmeet Singh, Maini, Jahnavi, and Hungin, Amrit Pal Singh Vaping versus Smoking: A Quest for Efficacy and Safety of E-cigarette. *Current drug safety* 2018; 13(2):92-101.
47. Scherer, Gerhard Suitability of biomarkers of biological effects (BOBEs) for assessing the likelihood of reducing the tobacco related disease risk by new and innovative tobacco products: A literature review. *Regulatory toxicology and pharmacology* 2018; 94:203-233.
48. Newton, John N., Dockrell, Martin, and Marczylo, Tim Making sense of the latest evidence on electronic cigarettes. *Lancet* 2018; 391(10121):639-642.
49. Zhang, Guangwei, Wang, Zhangli, Zhang, Kai, Hou, Rui, Xing, Chunli, Yu, Qi, and Liu, Enqi Safety Assessment of Electronic Cigarettes and Their Relationship with Cardiovascular Disease. *International journal of environmental research and public health* 2018; 15(1).
50. Tegin, Gulay, Mekala, Hema Madhuri, Sarai, Simrat Kaur, and Lippmann, Steven E-Cigarette Toxicity? *Southern medical journal* 2018; 111(1):35-38.
51. DeVito, Elise E. and Krishnan-Sarin, Suchitra E-cigarettes: Impact of E-Liquid Components and Device Characteristics on Nicotine Exposure. *Current neuropharmacology* 2018; 16(4):438-459.
52. Ziedonis, Douglas, Das, Smita, and Larkin, Celine Tobacco use disorder and treatment: new challenges and opportunities. *Dialogues in clinical neuroscience* 2017; 19(3):271-280.
53. Morjaria, J. B., Mondati, E., and Polosa, R. E-cigarettes in patients with COPD: current perspectives. *International journal of chronic obstructive pulmonary disease* 2017; 12:3203-3210.
54. Underner, M., Pourrat, O., Perriot, J., Peiffer, G., and Jaafari, N. [Smoking cessation and pregnancy]. *Gynecologie, obstetrique, fertilite & senologie* 2017; 45(10):552-557.
55. Drope, Jeffrey, Cahn, Zachary, Kennedy, Rosemary, Liber, Alex C., Stoklosa, Michal, Henson, Rosemarie, Douglas, Clifford E., and Drope, Jacqui Key issues surrounding the health impacts of electronic nicotine delivery systems (ENDS) and other sources of nicotine. *CA: a cancer journal for clinicians* 2017; 67(6):449-471.
56. Chiu Qua, Debbie Anne Tobacco Cessation Strategies: It Takes a Village. *South Dakota medicine* 2017; Spec No:54-58.
57. Das, Smita and Prochaska, Judith J. Innovative approaches to support smoking cessation for individuals with mental illness and co-occurring substance use disorders. *Expert review of respiratory medicine* 2017; 11(10):841-850.
58. Cai, Hua and Wang, Chen Graphical review: The redox dark side of e-cigarettes; exposure to oxidants and public health concerns. *Redox biology* 2017; 13:402-406.
59. Shields, Peter G., Berman, Micah, Brasky, Theodore M., Freudenheim, Jo L., Mathe, Ewy, McElroy, Joseph P., Song, Min Ae, and Wewers, Mark D. A Review of Pulmonary Toxicity of Electronic Cigarettes in the Context of Smoking: A Focus on Inflammation. *Cancer epidemiology, biomarkers & prevention* 2017; 26(8):1175-1191.
60. Dalkou, Sofia and Clair, Carole [Smoking, vaping and cardiovascular risk: an update]. *Revue medicale suisse* 2017; 13(566):1186-1190.
61. Jayakumar, Priyanga, Mekala, Hema Madhuri, Yeruva, Rajashekar Reddy, and Lippmann, Steven How to Stop Smoking. *The primary care companion for CNS disorders* 2017; 19(3).
62. Olenik, Angela and Mospan, Cortney M. Smoking cessation: Identifying readiness to quit and designing a plan. J*ournal of the American Academy of Physician Assistants* 2017; 30(7):13-19.
63. Baraona, L. Kim, Lovelace, Dawn, Daniels, Julie L., and McDaniel, Linda Tobacco Harms, Nicotine Pharmacology, and Pharmacologic Tobacco Cessation Interventions for Women. *Journal of midwifery & women's health* 2017; 62(3):253-269.
64. Ramoa, C. P., Eissenberg, T., and Sahingur, S. E. Increasing popularity of waterpipe tobacco smoking and electronic cigarette use: Implications for oral healthcare. *Journal of periodontal research* 2017; 52(5):813-823.
65. McCubbin, Andrea, Fallin-Bennett, Amanda, Barnett, Janine, and Ashford, Kristin Perceptions and use of electronic cigarettes in pregnancy. *Health education research* 2017; 32(1):22-32.
66. Hefner, Kathryn, Valentine, Gerald, and Sofuoglu, Mehmet Electronic cigarettes and mental illness: Reviewing the evidence for help and harm among those with psychiatric and substance use disorders. *The American journal on addictions* 2017; 26(4):306-315.
67. Zborovskaya, Yanina E-Cigarettes and Smoking Cessation: A Primer for Oncology Clinicians. *Clinical journal of oncology nursing* 2017; 21(1):54-63.
68. Bourke, Liam, Bauld, Linda, Bullen, Christopher, Cumberbatch, Marcus, Giovannucci, Edward, Islami, Farhad, McRobbie, Hayden, Silverman, Debra T., and Catto, James W. F. E-cigarettes and Urologic Health: A Collaborative Review of Toxicology, Epidemiology, and Potential Risks. *European urology* 2017; 71(6):915-923.
69. Huey, Sally and Granitto, Margaret Escape the vape: Health hazards of the latest nicotine craze. *Nursing* 2017; 47(1):46-51.
70. Glasser, Allison M., Collins, Lauren, Pearson, Jennifer L., Abudayyeh, Haneen, Niaura, Raymond S., Abrams, David B., and Villanti, Andrea C. Overview of Electronic Nicotine Delivery Systems: A Systematic Review. *American journal of preventive medicine* 2017; 52(2):e33-e66.
71. Ghosh, Sohini and Drummond, M. Bradley Electronic cigarettes as smoking cessation tool: are we there? *Current opinion in pulmonary medicine* 2017; 23(2):111-116.
72. Morphett, Kylie, Carter, Adrian, Hall, Wayne, and Gartner, Coral Medicalisation, smoking and e-cigarettes: evidence and implications. *Tobacco control* 2017; 26(e2):e134-e139.
73. Correa, John B., Ariel, Idan, Menzie, Nicole S., and Brandon, Thomas H. Documenting the emergence of electronic nicotine delivery systems as a disruptive technology in nicotine and tobacco science. *Addictive behaviors* 2017; 65:179-184.
74. Dautzenberg, Bertrand and Garelik, Daniel Patients with lung cancer: Are electronic cigarettes harmful or useful? *Lung cancer* 2017; 105:42-48.
75. Mathur, A. and Dempsey, O. J. Electronic cigarettes: a brief update. *The journal of the Royal College of Physicians of Edinburgh* 2018; 48(4):346-351.
76. Scherman, Ashley, Tolosa, Jorge E., and McEvoy, Cindy Smoking cessation in pregnancy: a continuing challenge in the United States. *Therapeutic advances in drug safety* 2018; 9(8):457-474.
77. Unger, Michael and Unger, Darian W. E-cigarettes/electronic nicotine delivery systems: a word of caution on health and new product development. *Journal of thoracic disease* 2018; 10(Suppl 22):S2588-S2592.
78. Formanek, Perry, Salisbury-Afshar, Elizabeth, and Afshar, Majid Helping Patients With ESRD and Earlier Stages of CKD to Quit Smoking. *American journal of kidney diseases* 2018; 72(2):255-266.
79. Naskar, Subrata and Jakati, Praveen Kumar "Vaping:" Emergence of a New Paraphernalia. *Indian journal of psychological medicine* 2017; 39(5):566-572.
80. Varlet, V. Drug Vaping: From the Dangers of Misuse to New Therapeutic Devices. *Toxics* 2016; 4(4).
81. Chatkin, Jose Miguel and Dullius, Cynthia Rocha The management of asthmatic smokers. *Asthma research and practice* 2016; 2:10-.
82. Knight-West, Oliver and Bullen, Christopher E-cigarettes for the management of nicotine addiction. *Substance abuse and rehabilitation* 2016; 7:111-118.
83. Chatterjee, Kshitij, Alzghoul, Bashar, Innabi, Ayoub, and Meena, Nikhil Is vaping a gateway to smoking: a review of the longitudinal studies. *International journal of adolescent medicine and health* 2016; 30(3).
84. Nansseu, Jobert Richie and Bigna, Jean Joel Electronic Cigarettes for Curbing the Tobacco-Induced Burden of Noncommunicable Diseases: Evidence Revisited with Emphasis on Challenges in Sub-Saharan Africa. *Pulmonary medicine* 2016; 2016:4894352-.
85. Xu, Ying, Guo, Yanfang, Liu, Kaiqian, Liu, Zheng, and Wang, Xiaobo E-Cigarette Awareness, Use, and Harm Perception among Adults: A Meta-Analysis of Observational Studies. *PloS one* 2016; 11(11):e0165938-.
86. Sharma, Ratika, Gartner, Coral E., and Hall, Wayne D. The challenge of reducing smoking in people with serious mental illness. *The Lancet.Respiratory medicine* 2016; 4(10):835-844.
87. Smith, Maurice R., Clark, Bruce, Ludicke, Frank, Schaller, Jean Pierre, Vanscheeuwijck, Patrick, Hoeng, Julia, and Peitsch, Manuel C. Evaluation of the Tobacco Heating System 2.2. Part 1: Description of the system and the scientific assessment program. *Regulatory toxicology and pharmacology* 2016; 81 Suppl 2:S17-S26.
88. Beard, Emma, Shahab, Lion, Cummings, Damian M., Michie, Susan, and West, Robert New Pharmacological Agents to Aid Smoking Cessation and Tobacco Harm Reduction: What Has Been Investigated, and What Is in the Pipeline? *CNS drugs* 2016; 30(10):951-983.
89. Cobb, Nathan K. and Sonti, Rajiv E-Cigarettes: The Science Behind the Smoke and Mirrors. *Respiratory care* 2016; 61(8):1122-1128.
90. Behavioral and Pharmacotherapy Interventions for Tobacco Smoking Cessation in Adults, Including Pregnant Women: Recommendation Statement. *American family physician* 2016; 93(10).
91. Mulhall, Patrick and Criner, Gerard Non-pharmacological treatments for COPD. *Respirology (Carlton, Vic.)* 2016; 21(5):791-809.
92. Stobbs, N., Lillis, A., and Kumar, N. E-cigarettes in ENT: what do we need to know? *The Journal of laryngology and otology* 2016; 130(6):512-515.
93. Benowitz, Neal L. and Burbank, Andrea D. Cardiovascular toxicity of nicotine: Implications for electronic cigarette use. *Trends in cardiovascular medicine* 2016; 26(6):515-523.
94. Das, Smita, Tonelli, Makenzie, and Ziedonis, Douglas Update on Smoking Cessation: E-Cigarettes, Emerging Tobacco Products Trends, and New Technology-Based Interventions. *Current psychiatry reports* 2016; 18(5):51-.
95. Berlin, Ivan [Smoking and electronic cigarettes in France]. *Presse medicale* 2016; 45(12 Pt 1):1141-1146.
96. Couch, Elizabeth T., Chaffee, Benjamin W., Gansky, Stuart A., and Walsh, Margaret M. The changing tobacco landscape: What dental professionals need to know. *Journal of the American Dental Association (1939)* 2016; 147(7):561-569.
97. Ribisl, Kurt M., Seidenberg, Andrew B., and Orlan, Elizabeth N. RECOMMENDATIONS FOR U.S. PUBLIC POLICIES REGULATING ELECTRONIC CIGARETTES. *Journal of policy analysis and management* 2016; 35(2):479-489.
98. Zellweger, Jean Pierre [Smoking cessation: how do we help the smokers?]. *Revue medicale suisse* 2016; 12(503):204-205.
99. Patel, Manish S. and Steinberg, Michael B. In the Clinic. Smoking Cessation. *Annals of internal medicine* 2016; 164(5):ITC33-ITC48.
100. Ioakeimidis, Nikolaos, Vlachopoulos, Charalambos, and Tousoulis, Dimitris Efficacy and Safety of Electronic Cigarettes for Smoking Cessation: A Critical Approach. *Hellenic journal of cardiology* 2016; 57(1):1-6.
101. Spindel, Eliot R. and McEvoy, Cindy T. The Role of Nicotine in the Effects of Maternal Smoking during Pregnancy on Lung Development and Childhood Respiratory Disease. Implications for Dangers of E-Cigarettes. *American journal of respiratory and critical care medicine* 2016; 193(5):486-494.
102. Steliga, M. A. and Yang, P. Integration of smoking cessation and lung cancer screening. *Translational lung cancer research* 2019; 8(Supplement1):S88-S94.
103. Helen, G. S. and Eaton, D. L. Public health consequences of e-cigarette use. *JAMA Internal Medicine* 2018; 178(7):984-986.
104. Andrikopoulos, G. I., Zagoriti, Z., Topouzis, S., and Poulas, K. Oxidative stress induced by electronic nicotine delivery systems (ENDS): Focus on respiratory system. *Current Opinion in Toxicology* 2019; 13:81-89.
105. Gulati, G. K. and Hinds, B. J. Smoking cessation potential of smartphone-assisted behavioral therapy coupled to programmable carbon nanotube membrane nicotine delivery device. *Critical Reviews in Therapeutic Drug Carrier Systems* 2018; 35(6):495-520.
106. Barboza, J. Pharmaceutical strategies for smoking cessation during pregnancy. *Expert Opinion on Pharmacotherapy* 2018; 19(18):2033-2042.
107. Kalkhoran, S., Benowitz, N. L., and Rigotti, N. A. Reprint of: Prevention and Treatment of Tobacco Use: JACC Health Promotion Series. *Journal of the American College of Cardiology* 2018; 72(23):2964-2979.
108. Abrams, D. B., Glasser, A. M., Villanti, A. C., Pearson, J. L., Rose, S., and Niaura, R. S. Managing nicotine without smoke to save lives now: Evidence for harm minimization. *Preventive medicine* 2018; 117:88-97.
109. Dawson, A. and Verweij, M. No smoke without fire: Harm reduction, e-cigarettes and the smoking endgame. *Public Health Ethics* 2017; 10(1):1-4.
110. Kaur, G., Pinkston, R., McLemore, B., Dorsey, W. C., and Batra, S. Immunological and toxicological risk assessment of e-cigarettes. *European Respiratory Review* 2018; 27(147):170119-.
111. Lindblom, E. N. Should FDA try to move smokers to e-cigarettes and other less-harmful tobacco products and, if so, how? *Food and Drug Law Journal* 2018; 73(2):276-318.
112. Perloff, A., Smeltzer, K., Cox, W. J., and Desai, G. J. E-cigarettes: Facts for the osteopathic family physician. *Osteopathic Family Physician* 2018; 10(2):17-20.
113. Bhatnagar, A. Cardiovascular Perspective of the Promises and Perils of E-Cigarettes. *Circulation Research* 2016; 118(12):1872-1875.
114. Derefinko, K. J., Salgado Garcia, F. I., and Sumrok, D. D. Smoking Cessation for Those Pursuing Recovery from Substance Use Disorders. *Medical Clinics of North America* 2018; 102(4):781-796.
115. Ismail, A. F. and Ghazali, A. F. Electronic cigarettes and oral health: A narrative review. *International Journal of Pharmaceutical Research* 2018; 10(2):84-86.
116. Onor, I. O., Stirling, D. L., Williams, S. R., Bediako, D., Borghol, A., Harris, M. B., Darensburg, T. B., Clay, S. D., Okpechi, S. C., and Sarpong, D. F. Clinical effects of cigarette smoking: Epidemiologic impact and review of pharmacotherapy options. *International journal of environmental research and public health* 2017; 14(10):1147-.
117. Amin, Z., Zulkifly, S., and Iskandar, S. D. Vaping as smoking cessation methods: Is it a solution or a new problem? *Indian Journal of Public Health Research and Development* 2017; 8(3):104-108.
118. Noble, M. J. The New Dangers of Electronic Cigarettes. *Clinical Pediatric Emergency Medicine* 2017; 18(3):163-172.
119. Sadowski, I. J., Humai, J.-P., and Cornuz, J. Vaping (electronic cigarette): How to advise smokers in 2017? *Revue medicale suisse* 2017; 13(566):1181-1185.
120. Gaznick, N. V. and Anthenelli, R. M. Do they work for smoking cessation and should we be recommending their use? *Current Psychiatry* 2017; 16(5):30-39.
121. Pang, E. and Stern, M. Effective ways of supporting patients to stop smoking. *Prescriber* 2017; 28(2):14-20.
122. Ratchford, E. V. and Evans, N. S. Smoking cessation. *Vascular Medicine* 2016; 21(5):477-479.
123. Leduc, C. and Quoix, E. Is there a role for e-cigarettes in smoking cessation? *Therapeutic advances in respiratory disease* 2016; 10(2):130-135.
124. Bhatnagar, A. E-Cigarettes and Cardiovascular Disease Risk: Evaluation of Evidence, Policy Implications, and Recommendations. *Current Cardiovascular Risk Reports* 2016; 10(7):24-.
125. Mendelsohn, C. P. Electronic cigarettes: What can we learn from the UK experience?: Electronic cigarettes have the potential for substantial improvements in public health. *Medical Journal of Australia* 2016; 204(1):14-.
126. Marsot, A. and Simon, N. Nicotine and Cotinine Levels with Electronic Cigarette. *International Journal of Toxicology* 2016; 35(2):179-185.
127. Jhanjee, S. Putting tobacco harm reduction in perspective: Is it a viable alternative? *Indian Journal of Medical Research* 2016; 143(JANUARY):25-29.
128. Drugs for tobacco dependence. *Medical Letter on Drugs and Therapeutics* 2016; 58(1489):27-31.
129. Polosa, R., Campagna, D., and Sands, M. F. Counseling patients with asthma and allergy about electronic cigarettes: An evidence-based approach. *Annals of Allergy, Asthma and Immunology* 2016; 116(2):106-111.
130. Gray, N. Why we should remain sceptical about e-cigarettes. *Pharmaceutical Journal* 2016; 296(7890):355-356.
131. Reid, R. D., Pritchard, G., Walker, K., Aitken, D., Mullen, K.-A., and Pipe, A. L. Managing smoking cessation. *Canadian Medical Association journal* 2016; 188(17-18):E484-E492.
132. Nayir, E., Karacabey, B., Kirca, O., and Ozdogan, M. Electronic cigarette (e-cigarette). *Journal of Oncological Science* 2016; 2(1):16-20.
133. Vijayaraghavan, M., Schroeder, S. A., and Kushel, M. The effectiveness of tobacco control policies on vulnerable populations in the USA: A review. *Postgraduate medical journal* 2016; 92(1093):670-676.
134. David, T. and Tharyan, P. Electronic cigarettes for smoking cessation and reduction. Summary of the evidence and implications for public health programmes. *Clinical Epidemiology and Global Health* 2016; 4(3):146-150.
135. Perret, J. L., Bonevski, B., McDonald, C. F., and Abramson, M. J. Smoking cessation strategies for patients with asthma: Improving patient outcomes. *Journal of Asthma and Allergy* 2016; 9:117-128.
136. Borgne, Anne How to integrate support and tools for lower risk consumption in daily practice? The case of tobacco. *Alcoologie et Addictologie* 2017; 39(4):373-380.
137. Kenkel, Donald S. Healthy innovation: Vaping, smoking, and public policy. *Journal of Policy Analysis and Management* 2016; 35(2):473-479.
138. The Ontario Tobacco Research Unit Interventions to Prevent Harms from Vaping.
139. Public Health England E-cigarettes and heated tobacco products: an evidence review.
140. Anonymous Drugs for smoking cessation. [Review]. 2019
141. van Teijlingen E, Mahato P, Simkhada P, van Teijlingen C, Asim M, Sathian B. Vaping and e-cigarettes: A public health warning or a health promotion tool? Nepal Journal of Epidemiology. 2019;9(4):792–4.

### Other (incl. non-comparative obs) (n=570)

1. Lorkiewicz, Pawel, Riggs, Daniel W., Keith, Rachel J., Conklin, Daniel J., Xie, Zhengzhi, Sutaria, Saurin, Lynch, Blake, Srivastava, Sanjay, and Bhatnagar, Aruni Comparison of Urinary Biomarkers of Exposure in Humans Using Electronic Cigarettes, Combustible Cigarettes, and Smokeless Tobacco. *Nicotine & tobacco research* 2018.
2. McDonald, Christine F. E-cigarettes for smoking cessation: Current state of play. *Respirology* 2019.
3. Graham, Amanda L., Jacobs, Megan A., and Amato, Michael S. Engagement and 3-month outcomes from a digital e-cigarette cessation program in a cohort of 27,000 teens and young adults. *Nicotine & tobacco research* 2019.
4. Rosen, Rachel L. and Steinberg, Marc L. Interest in Quitting E-Cigarettes among Adults in the United States. *Nicotine & tobacco research* 2019.
5. Johnson, Linda, Ma, Yinjiao, Fisher, Sherri L., Ramsey, Alex T., Chen, Li Shiun, Hartz, Sarah M., Culverhouse, Robert C., Grucza, Richard A., Saccone, Nancy L., Baker, Timothy B., and Bierut, Laura J. E-cigarette Usage Is Associated With Increased Past-12-Month Quit Attempts and Successful Smoking Cessation in Two US Population-Based Surveys. *Nicotine & tobacco research* 2018.
6. Jackson, Sarah, Kotz, Daniel, West, Robert, and Brown, Jamie Moderators of real-world effectiveness of smoking cessation aids: a population study. *Addiction* 2019.
7. Jackson, Sarah E., Shahab, Lion, Garnett, Claire, and Brown, Jamie Trends in and correlates of use of roll-your-own cigarettes: a population study in England 2008-2017. *Nicotine & tobacco research* 2019.
8. De La Garza, Richard, Shuman, Samuel L., Yammine, Luba, Yoon, Jin Ho, Salas, Ramiro, and Holst, Manuela A Pilot Study of E-Cigarette Naive Cigarette Smokers and the Effects on Craving After Acute Exposure to E-Cigarettes in the Laboratory. *The American journal on addictions* 2019.
9. Piper, Megan E., Baker, Timothy B., Benowitz, Neal L., and Jorenby, Douglas E. Changes in Use Patterns OVER ONE YEAR Among Smokers and Dual Users of Combustible and electronic cigarettes. *Nicotine & tobacco research* 2019.
10. Cheng, Kai Wen, Chaloupka, Frank J., Shang, Ce, Ngo, Anh, Fong, Geoffrey T., Borland, Ron, Heckman, Bryan W., Levy, David T., and Cummings, K. Michael Prices, use restrictions and electronic cigarette use-evidence from wave 1 (2016) US data of the ITC Four Country Smoking and Vaping Survey. *Addiction* 2019.
11. Collins, Susan E., Nelson, Lonnie A., Stanton, Joey, Mayberry, Nigel, Ubay, Tatiana, Taylor, et al. Harm reduction treatment for smoking (HaRT-S): findings from a single-arm pilot study with smokers experiencing chronic homelessness. *Substance abuse* 2019:1-11.
12. Piper, Megan E., Baker, Timothy B., Benowitz, Neal L., Smith, Stevens S., and Jorenby, Douglas E. E-cigarette Dependence Measures in Dual Users: Reliability and Relations with Dependence Criteria and E-Cigarette Cessation. *Nicotine & tobacco research* 2019.
13. Soule, Eric K., Plunk, Andrew D., Harrell, Paul T., Hayes, Rashelle B., and Edwards, Kathryn C. Longitudinal analysis of associations between reasons for electronic cigarette use and change in smoking status among adults in the Population Assessment of Tobacco and Health Study. *Nicotine & tobacco research* 2019.
14. Martinez, Ursula, Martinez-Loredo, Victor, Simmons, Vani N., Meltzer, Lauren R., Drobes, David J., Brandon, Karen O., Palmer, Amanda M., Eissenberg, Thomas, Bullen, Christopher R., Harrell, Paul T., and Brandon, Thomas H. How Does Smoking and Nicotine Dependence Change after Onset of Vaping? A Retrospective Analysis of Dual Users. *Nicotine & tobacco research* 2019.
15. Watkins, Shannon Lea, Thrul, Johannes, Max, Wendy, and Ling, Pamela Cold Turkey and Hot Vapes? A national study of young adult cigarette cessation strategies. *Nicotine & tobacco research* 2018.
16. Levy, David T., Yuan, Zhe, Li, Yameng, Alberg, Anthony J., and Cummings, K. Michael A modeling approach to gauging the effects of nicotine vaping product use on cessation from cigarettes: what do we know, what do we need to know? *Addiction* 2018.
17. Chan, Gary, Morphett, Kylie, Gartner, Coral, Leung, Janni, Yong, Hua Hie, Hall, Wayne, and Borland, Ron Predicting vaping uptake, vaping frequency and ongoing vaping among daily smokers using longitudinal data from the International Tobacco Control (ITC) Four Country Surveys. *Addiction* 2018.
18. Oh, HyunSoo, Im, BoAe, and Seo, WhaSook Comparisons of the stages and psychosocial factors of smoking cessation and coping strategies for smoking cessation in college student smokers: Conventional cigarette smokers compared to dual smokers of conventional and e-cigarettes. *Japan journal of nursing science* 2018.
19. Piper, Megan E., Baker, Timothy B., Benowitz, Neal L., Kobinsky, Kate, and Jorenby, Douglas E. Dual Users Compared to Smokers: Demographics, Dependence, and Biomarkers. *Nicotine & tobacco research* 2018.
20. Verplaetse, Terril L., Moore, Kelly E., Pittman, Brian P., Roberts, Walter, Oberleitner, Lindsay M., Peltier, MacKenzie R., Hacker, Robyn, Cosgrove, Kelly P., and McKee, Sherry A. Intersection of e-cigarette use and gender on transitions in cigarette smoking status: Findings across waves 1 and 2 of the Population Assessment of Tobacco and Health (PATH) study. *Nicotine & tobacco research* 2018.
21. Masiero, Marianna, Lucchiari, Claudio, Mazzocco, Ketti, Veronesi, Giulia, Maisonneuve, Patrick, Jemos, Costantino, Sale, Emanuela Omodeo, Spina, Stefania, Bertolotti, Raffaella, and Pravettoni, Gabriella E-Cigarettes May Support Smokers With High Smoking-Related Risk Awareness to Stop Smoking in the Short Run: Preliminary Results by Randomized Controlled Trial. *Nicotine & tobacco research* 2018.
22. Harlow, Alyssa, Stokes, Andrew, and Brooks, Daniel Socio-economic and racial/ethnic differences in e-cigarette uptake among cigarette smokers: Longitudinal analysis of the Population Assessment of Tobacco and Health (PATH) study. *Nicotine & tobacco research* 2018.
23. Blank, Mei Ling, Hoek, Janet, George, Mark, Gendall, Philip, Conner, Tamlin S., Thrul, Johannes, Ling, Pamela M., and Langlotz, Tobias An Exploration of Smoking-to-Vaping Transition Attempts Using a "Smart" Electronic Nicotine Delivery System. *Nicotine & tobacco research* 2018.
24. Zvolensky, Michael J., Mayorga, Nubia A., and Garey, Lorra Positive Expectancies for E-Cigarette Use and Anxiety Sensitivity among Adults. *Nicotine & tobacco research* 2018.
25. DeVito, Elise E., Buta, Eugenia, and Sofuoglu, Mehmet E-cigarette nicotine dose and flavor: Relationship with appeal, choice, and tobacco use amongst veterans with comorbid psychiatric disorders. *Addictive behaviors* 2019; 92:53-57.
26. Wallace, Alison M. and Foronjy, Robert E. Electronic cigarettes: not evidence-based cessation. *Translational lung cancer research* 2019; 8(Suppl 1):S7-S10.
27. Coleman, Blair, Rostron, Brian, Johnson, Sarah E., Persoskie, Alexander, Pearson, Jennifer, et al. Transitions in electronic cigarette use among adults in the Population Assessment of Tobacco and Health (PATH) Study, Waves 1 and 2 (2013-2015). *Tobacco control* 2019; 28(1):50-59.
28. Zvolensky, Michael J., Manning, Kara, Garey, Lorra, Mayorga, Nubia A., and Peraza, Natalia Fatigue severity and electronic cigarette beliefs and use behavior. *Addictive behaviors* 2019; 97:1-6.
29. Bhatnagar, Aruni, Payne, Thomas J., and Robertson, Rose Marie Is There A Role for Electronic Cigarettes in Tobacco Cessation? *Journal of the American Heart Association* 2019; 8(12):e012742-.
30. Owens, Victoria L., Ha, Trung, and Soulakova, Julia N. Widespread use of flavored e-cigarettes and hookah tobacco in the United States. *Preventive medicine reports* 2019; 14:100854-.
31. Bhatta, Dharma N. and Glantz, Stanton A. Electronic Cigarette Use and Myocardial Infarction Among Adults in the US Population Assessment of Tobacco and Health. *Journal of the American Heart Association* 2019; 8(12):e012317-.
32. Court, Arlene E-cigarettes: Guiding patients in a time of uncertainty. *Canadian oncology nursing journal = Revue canadienne de nursing oncologique* 2016; 26(1):83-85.
33. Camenga, Deepa R., Kong, Grace, Cavallo, Dana A., and Krishnan-Sarin, Suchitra Current and Former Smokers' Use of Electronic Cigarettes for Quitting Smoking: An Exploratory Study of Adolescents and Young Adults. *Nicotine & tobacco research* 2019; 21(3):395-.
34. Snow, Erika, Johnson, Tye, Ossip, Deborah J., Williams, Geoffrey C., Ververs, Duncan, Rahman, Irfan, and McIntosh, Scott Does E-cigarette Use at Baseline Influence Smoking Cessation Rates among 2-Year College Students? *Journal of smoking cessation* 2018; 13(2):110-120.
35. Choi, Kelvin, Bestrashniy, Jessica, and Forster, Jean Trends in Awareness, Use of, and Beliefs About Electronic Cigarette and Snus Among a Longitudinal Cohort of US Midwest Young Adults. *Nicotine & tobacco research* 2018; 20(2):239-245.
36. Pulvers, Kim, Emami, Ashley S., Nollen, Nicole L., Romero, Devan R., Strong, David R., Benowitz, Neal L., and Ahluwalia, Jasjit S. Tobacco Consumption and Toxicant Exposure of Cigarette Smokers Using Electronic Cigarettes. *Nicotine & tobacco research* 2018; 20(2):206-214.
37. Erly, Brian K. and Prochazka, Allan V. E-cigarettes were more effective than nicotine replacement for smoking cessation at 1 year. *Annals of internal medicine* 2019; 170(10):JC50-.
38. Kurti, Allison N., Redner, Ryan, Bunn, Janice Y., Tang, Katherine, Nighbor, Tyler, Lopez, Alexa A., et al. Examining the relationship between pregnancy and quitting use of tobacco products in a U.S. national sample of women of reproductive age. *Preventive medicine* 2018; 117:52-60.
39. Berg, Carla J., Haardorfer, Regine, Payne, Jackelyn B., Getachew, Betelihem, Vu, Milkie, Guttentag, Alexandra, and Kirchner, Thomas R. Ecological momentary assessment of various tobacco product use among young adults. *Addictive behaviors* 2019; 92:38-46.
40. Carroll, Dana Mowls, Wagener, Theodore L., Stephens, Lancer D., Brame, Lacy S., Thompson, David M., and Beebe, Laura A. The relationship between nicotine metabolism and nicotine and carcinogen exposure among American Indian commercial cigarette smokers and electronic nicotine delivery system users. *Addictive behaviors* 2019; 92:58-63.
41. Goniewicz, Maciej L. and Smith, Danielle M. Are Some E-Cigarette Users "Blowing Smoke"?: Assessing the Accuracy of Self-Reported Smoking Abstinence in Exclusive E-Cigarette Users. *Nicotine & tobacco research* 2019; 21(5):699-700.
42. Spears, Claire Adams, Jones, Dina M., Weaver, Scott R., Pechacek, Terry F., and Eriksen, Michael P. Motives and perceptions regarding electronic nicotine delivery systems (ENDS) use among adults with mental health conditions. *Addictive behaviors* 2018; 80:102-109.
43. Berry, Kaitlyn M., Reynolds, Lindsay M., Collins, Jason M., Siegel, Michael B., Fetterman, Jessica L., Hamburg, Naomi M., Bhatnagar, Aruni, Benjamin, Emelia J., and Stokes, Andrew E-cigarette initiation and associated changes in smoking cessation and reduction: the Population Assessment of Tobacco and Health Study, 2013-2015. *Tobacco control* 2019; 28(1):42-49.
44. Filippidis, Filippos T., Laverty, Anthony A., Mons, Ute, Jimenez-Ruiz, Carlos, and Vardavas, Constantine I. Changes in smoking cessation assistance in the European Union between 2012 and 2017: pharmacotherapy versus counselling versus e-cigarettes. *Tobacco control* 2019; 28(1):95-100.
45. Watson, Michael Craig and Lloyd, John Why the rules should not be relaxed for e-cigarettes to encourage people to stop smoking. *BMJ* 2019; 364:l555-.
46. Russell, Christopher, Haseen, Farhana, and McKeganey, Neil Factors associated with past 30-day abstinence from cigarette smoking in a non-probabilistic sample of 15,456 adult established current smokers in the United States who used JUUL vapor products for three months. *Harm reduction journal* 2019; 16(1):22-.
47. Wise, Jacqui E-cigarettes: relax rules to help more smokers quit, say MPs. *BMJ* 2018; 362:k3571-.
48. Hajek, Peter, Peerbux, Sarrah, Phillips-Waller, Anna, Smith, Charlotte, Pittaccio, Kate, and Przulj, Dunja Are 'dual users' who smoke and use e-cigarettes interested in using varenicline to stop smoking altogether, and can they benefit from it? A cohort study of UK vapers. *BMJ open* 2019; 9(3):e026642-.
49. Mayor, Susan E-cigarettes help twice as many smokers quit as nicotine replacement therapy, trial finds. *BMJ* 2019; 364:l473-.
50. E-cigarettes Best Other Cessation Tools. *Cancer discovery* 2019; 9(4):OF3-.
51. Hair, Elizabeth C., Romberg, Alexa R., Niaura, Raymond, Abrams, David B., Bennett, Morgane A., Xiao, Haijun, Rath, Jessica M., Pitzer, Lindsay, and Vallone, Donna Longitudinal Tobacco Use Transitions Among Adolescents and Young Adults: 2014-2016. *Nicotine & tobacco research* 2019; 21(4):458-468.
52. E-cigarette Report Reveals Research Gaps. *Cancer discovery* 2018; 8(3):OF2-.
53. Kock, Loren, Shahab, Lion, West, Robert, and Brown, Jamie E-cigarette use in England 2014-17 as a function of socio-economic profile. *Addiction* 2019; 114(2):294-303.
54. Beard, Emma, Brown, Jamie, Michie, Susan, and West, Robert Is prevalence of e-cigarette and nicotine replacement therapy use among smokers associated with average cigarette consumption in England? A time-series analysis. *BMJ open* 2018; 8(6):e016046-.
55. Slomski, Anita e-Cigarettes for Smoking Cessation. *JAMA* 2019; 321(12):1149-.
56. Chen, Julia Cen Flavored E-cigarette Use and Cigarette Smoking Reduction and Cessation-A Large National Study among Young Adult Smokers. *Substance use & misuse* 2018; 53(12):2017-2031.
57. Rohsenow, Damaris J., Tidey, Jennifer W., Martin, Rosemarie A., Colby, Suzanne M., and Eissenberg, Thomas Effects of six weeks of electronic cigarette use on smoking rate, CO, cigarette dependence, and motivation to quit smoking: A pilot study. *Addictive behaviors* 2018; 80:65-70.
58. Soule, Eric K., Maloney, Sarah F., Guy, Mignonne C., Eissenberg, Thomas, and Fagan, Pebbles User-identified electronic cigarette behavioral strategies and device characteristics for cigarette smoking reduction. *Addictive behaviors* 2018; 79:93-101.
59. Valentine, Gerald W., Hefner, Kathryn, Jatlow, Peter I., Rosenheck, Robert A., Gueorguieva, Ralitza, and Sofuoglu, Mehmet Impact of E-cigarettes on Smoking and Related Outcomes in Veteran Smokers With Psychiatric Comorbidity. *Journal of dual diagnosis* 2018; 14(1):2-13.
60. Prochaska, Judith J. The public health consequences of e-cigarettes: a review by the National Academies of Sciences. A call for more research, a need for regulatory action. *Addiction (Abingdon, England)* 2019; 114(4):587-589.
61. Das, Manjulika E-cigarettes and smoking cessation. *The Lancet.Oncology* 2019; 20(3):e136-.
62. Garey, Lorra, Mayorga, Nubia Angelina, Peraza, Natalia, Smit, Tanya, Nizio, Pamella, Otto, Michael W., and Zvolensky, Michael J. Distinguishing Characteristics of E-Cigarette Users Who Attempt and Fail to Quit: Dependence, Perceptions, and Affective Vulnerability. *Journal of studies on alcohol and drugs* 2019; 80(1):134-140.
63. Sweet, Laura, Brasky, Theodore M., Cooper, Sarah, Doogan, Nathan, Hinton, Alice, Klein, Elizabeth G., Nagaraja, Haikady, Quisenberry, Amanda, Xi, Wenna, and Wewers, Mary Ellen Quitting Behaviors Among Dual Cigarette and E-Cigarette Users and Cigarette Smokers Enrolled in the Tobacco User Adult Cohort. *Nicotine & tobacco research* 2019; 21(3):278-284.
64. Loukas, Alexandra, Marti, C. Nathan, and Perry, Cheryl L. Trajectories of Tobacco and Nicotine Use Across Young Adulthood, Texas, 2014-2017. *American journal of public health* 2019; 109(3):465-471.
65. Cooper, Maria R., Case, Kathleen R., Hebert, Emily T., Vandewater, Elizabeth A., Raese, Kristen A., Perry, Cheryl L., and Businelle, Michael S. Characterizing ENDS use in young adults with ecological momentary assessment: Results from a pilot study. *Addictive behaviors* 2019; 91:30-36.
66. Mead, Erin L., Duffy, Valerie, Oncken, Cheryl, and Litt, Mark D. E-cigarette palatability in smokers as a function of flavorings, nicotine content and propylthiouracil (PROP) taster phenotype. *Addictive behaviors* 2019; 91:37-44.
67. Perry, Cheryl L., Perez, Adriana, Bluestein, Meagan, Garza, Nicholas, Obinwa, Udoka, Jackson, Christian, Clendennen, Stephanie L., Loukas, Alexandra, and Harrell, Melissa B. Youth or Young Adults: Which Group Is at Highest Risk for Tobacco Use Onset? *The Journal of adolescent health* 2018; 63(4):413-420.
68. Wiernik, Emmanuel, Airagnes, Guillaume, Lequy, Emeline, Gomajee, Ramchandar, Melchior, Maria, Le Faou, Anne Laurence, Limosin, Frederic, Goldberg, Marcel, Zins, Marie, and Lemogne, Cedric Electronic cigarette use is associated with depressive symptoms among smokers and former smokers: Cross-sectional and longitudinal findings from the Constances cohort. *Addictive behaviors* 2019; 90:85-91.
69. Warner, Kenneth E. and Mendez, David E-cigarettes: Comparing the Possible Risks of Increasing Smoking Initiation with the Potential Benefits of Increasing Smoking Cessation. *Nicotine & tobacco research* 2019; 21(1):41-47.
70. Marti, Joachim, Buckell, John, Maclean, Johanna Catherine, and Sindelar, Jody To "vape" or smoke? Experimental evidence on adult smokers. *Economic inquiry* 2019; 57(1):705-725.
71. The, Lancet Oncology E-cigarettes-new product, old tricks. *The Lancet Oncology* 2018; 19(12):1543-.
72. Carr, Ellen Targeting Nicotine Addiction. *Clinical journal of oncology nursing* 2018; 22(3):243-244.
73. Pattemore, Philip E-cigarettes: NRT or new commercial addictive agents? *The New Zealand medical journal* 2018; 131(1472):102-.
74. Zhang, Yuqing and Upson, Dona E-Cigarettes versus Nicotine-Replacement Therapy for Smoking Cessation. *The New England journal of medicine* 2019; 380(20):1973-.
75. Pommer, Peter E-Cigarettes and Quitting Tobacco. *Deutsches Arzteblatt international* 2018; 115(27-28):479-.
76. Cook, Rob, Davidson, Peter, Martin, Rosie, and NIHR Dissemination Centre E-cigarettes helped more smokers quit than nicotine replacement therapy. *BMJ (Clinical research ed.)* 2019; 365:l2036-.
77. Chanchlani, Neil E-cigarettes: friend or foe? *BMJ* 2019; 364:j5150-.
78. Leventhal, Adam M., Goldenson, Nicholas I., Aguirre, Claudia G., Huh, Jimi, and Kirkpatrick, Matthew G. Initial application of a human laboratory model for estimating the motivational substitutability of e-cigarettes for combustible cigarettes. *Experimental and clinical psychopharmacology* 2019; 27(2):125-135.
79. Agarwal, Deepti, Loukas, Alexandra, and Perry, Cheryl L. Examining College Students' Social Environment, Normative Beliefs, and Attitudes in Subsequent Initiation of Electronic Nicotine Delivery Systems. *Health education & behavior* 2018; 45(4):532-539.
80. Stower, Hannah E-cigarettes to help smoking cessation. *Nature medicine* 2019; 25(3):358-.
81. Chang, Sam S. Re: The Health Effects of Electronic Cigarettes. *The Journal of urology* 2017; 198(6):1207-.
82. Lamb, Norman E-cigarettes. *Lancet* 2019; 393(10174):876-.
83. Ikonomidis, Ignatios, Vlastos, Dimitrios, Kourea, Kallirrhoe, Kostelli, Gavriela, Varoudi, Maria, Pavlidis, George, Efentakis, Panagiotis, Triantafyllidi, Helen, Parissis, John, Andreadou, Ioanna, Iliodromitis, Efstathios, and Lekakis, John Electronic Cigarette Smoking Increases Arterial Stiffness and Oxidative Stress to a Lesser Extent Than a Single Conventional Cigarette: An Acute and Chronic Study. *Circulation* 2018; 137(3):303-306.
84. Jackson, Sarah E., Beard, Emma, Michie, Susan, Shahab, Lion, Raupach, Tobias, West, Robert, and Brown, Jamie Are smokers who are regularly exposed to e-cigarette use by others more or less motivated to stop or to make a quit attempt? A cross-sectional and longitudinal survey. *BMC medicine* 2018; 16(1):206-.
85. Kasza, Karin A., Coleman, Blair, Sharma, Eva, Conway, Kevin P., Cummings, K. Michael, Goniewicz, Maciej L., et al. Correlates of Transitions in Tobacco Product Use by U.S. Adult Tobacco Users between 2013-2014 and 2014-2015: Findings from the PATH Study Wave 1 and Wave 2. *International journal of environmental research and public health* 2018; 15(11).
86. Young-Wolff, Kelly C., Klebaner, Daniella, Folck, Bruce, Tan, Andy S. L., Fogelberg, Renee, Sarovar, Varada, and Prochaska, Judith J. Documentation of e-cigarette use and associations with smoking from 2012 to 2015 in an integrated healthcare delivery system. *Preventive medicine* 2018; 109:113-118.
87. Chapman, Simon The UK is hopelessly smitten with e-cigarettes. *BMJ* 2018; 361:k2279-.
88. Hemsing, Natalie and Greaves, Lorraine New Challenges: Developing Gendered and Equitable Responses to Involuntary Exposures to Electronic Nicotine Delivery Systems (ENDS) and Cannabis Vaping. *International journal of environmental research and public health* 2018; 15(10).
89. Burki, Talha Khan Public Health England publishes e-cigarette review. *The Lancet Oncology* 2018; 19(3):e150-.
90. Kasza, Karin A., Borek, Nicolette, Conway, Kevin P., Goniewicz, Maciej L., Stanton, Cassandra A., et al. Transitions in Tobacco Product Use by U.S. Adults between 2013-2014 and 2014-2015: Findings from the PATH Study Wave 1 and Wave 2. *International journal of environmental research and public health* 2018; 15(11).
91. George, Johnson and Thomas, Dennis E-cigarettes for harm minimisation: absence of evidence or evidence of absence? *The International journal of pharmacy practice* 2018; 26(5):377-379.
92. Polosa, Riccardo, Morjaria, Jaymin Bhagwanji, Prosperini, Umberto, Russo, Cristina, Pennisi, Alfio, Puleo, Rosario, Caruso, Massimo, and Caponnetto, Pasquale Health effects in COPD smokers who switch to electronic cigarettes: a retrospective-prospective 3-year follow-up. *International journal of chronic obstructive pulmonary disease* 2018; 13:2533-2542.
93. Kuehn, Bridget M. Emerging Data Show E-Cigarettes May Pose Heart Risk. *Circulation* 2017; 136(2):232-233.
94. Menakuru, Sasmith and Inzamam Ali, Mir Beliefs and reality of e-cigarette smoking. *BMJ case reports* 2018.
95. Khorassani, Farah, Kaufman, Milena, and Lopez, Leonardo V. Supatherapeutic Serum Clozapine Concentration After Transition From Traditional to Electronic Cigarettes. *Journal of clinical psychopharmacology* 2018; 38(4):391-392.
96. Kocar, Thomas, Freudenmann, Roland W., Spitzer, Manfred, and Graf, Heiko Switching From Tobacco Smoking to Electronic Cigarettes and the Impact on Clozapine Levels. *Journal of clinical psychopharmacology* 2018; 38(5):528-529.
97. Weaver, Scott R., Huang, Jidong, Pechacek, Terry F., Heath, John Wesley, Ashley, David L., and Eriksen, Michael P. Are electronic nicotine delivery systems helping cigarette smokers quit? Evidence from a prospective cohort study of U.S. adult smokers, 2015-2016. *PloS one* 2018; 13(7):e0198047-.
98. Wu, Socrates Yong-da, Wang, Man Ping, Li, William H., Kwong, Antonio C., Lai, Vienna W., and Lam, Tai Hing Does Electronic Cigarette Use Predict Abstinence from Conventional Cigarettes among Smokers in Hong Kong? *International journal of environmental research and public health* 2018; 15(3).
99. Levy, David T., Borland, Ron, Villanti, Andrea C., Niaura, Raymond, Yuan, Zhe, Zhang, Yian, Meza, Rafael, Holford, Theodore R., Fong, Geoffrey T., Cummings, K. Michael, and Abrams, David B. The Application of a Decision-Theoretic Model to Estimate the Public Health Impact of Vaporized Nicotine Product Initiation in the United States. *Nicotine & tobacco research* 2017; 19(2):149-159.
100. Beckert, Lutz and Jones, Stuart E-cigarettes-peering into the mist of uncertainty. *The New Zealand medical journal* 2018; 131(1470):8-10.
101. Borrelli, Belinda and O'Connor, George T. E-Cigarettes to Assist with Smoking Cessation. *The New England journal of medicine* 2019; 380(7):678-679.
102. Drazen, Jeffrey M., Morrissey, Stephen, and Campion, Edward W. The Dangerous Flavors of E-Cigarettes. *The New England journal of medicine* 2019; 380(7):679-680.
103. Delgado-Ron, Jorge Andres E-Cigarettes Are Less Dangerous Than Cigarettes but Not Entirely Safe. *Pediatrics* 2018; 141(6).
104. Britton, John Electronic cigarettes and smoking cessation in England. *BMJ* 2016; 354:i4819-.
105. Mohamed, Mohamad Haniki Nik, Rahman, Azizur, Jamshed, Shazia, and Mahmood, Syed Effectiveness and safety of electronic cigarettes among sole and dual user vapers in Kuantan and Pekan, Malaysia: a six-month observational study. *BMC public health* 2018; 18(1):1028-.
106. Jorenby, Douglas E., Smith, Stevens S., Fiore, Michael C., and Baker, Timothy B. Nicotine levels, withdrawal symptoms, and smoking reduction success in real world use: A comparison of cigarette smokers and dual users of both cigarettes and E-cigarettes. *Drug and alcohol dependence* 2017; 170:93-101.
107. Lopez, Alexa A., Hiler, Marzena, Maloney, Sarah, Eissenberg, Thomas, and Breland, Alison B. Expanding clinical laboratory tobacco product evaluation methods to loose-leaf tobacco vaporizers. *Drug and alcohol dependence* 2016; 169:33-40.
108. Soule, Eric K., Lopez, Alexa A., Guy, Mignonne C., and Cobb, Caroline O. Reasons for using flavored liquids among electronic cigarette users: A concept mapping study. *Drug and alcohol dependence* 2016; 166:168-176.
109. Oncken, Cheryl, Ricci, Karen A., Kuo, Chia Ling, Dornelas, Ellen, Kranzler, Henry R., and Sankey, Heather Z. Correlates of Electronic Cigarettes Use Before and During Pregnancy. *Nicotine & tobacco research* 2017; 19(5):585-590.
110. Spindle, Tory R., Talih, Soha, Hiler, Marzena M., Karaoghlanian, Nareg, Halquist, Matthew S., Breland, Alison B., Shihadeh, Alan, and Eissenberg, Thomas Effects of electronic cigarette liquid solvents propylene glycol and vegetable glycerin on user nicotine delivery, heart rate, subjective effects, and puff topography. *Drug and alcohol dependence* 2018; 188:193-199.
111. Barna, Sandor, Rozsa, David, Varga, Jozsef, Fodor, Andrea, Szilasi, Maria, Galuska, Laszlo, and Garai, Ildiko First comparative results about the direct effect of traditional cigarette and e-cigarette smoking on lung alveolocapillary membrane using dynamic ventilation scintigraphy. *Nuclear medicine communications* 2019; 40(2):153-158.
112. Yang, Bo, Liu, Jiaying, and Popova, Lucy Targeted Versus Nontargeted Communication About Electronic Nicotine Delivery Systems in Three Smoker Groups. *International journal of environmental research and public health* 2018; 15(10).
113. McKeganey, Neil, Miler, Joanna Astrid, and Haseen, Farhana The Value of Providing Smokers with Free E-Cigarettes: Smoking Reduction and Cessation Associated with the Three-Month Provision to Smokers of a Refillable Tank-Style E-Cigarette. *International journal of environmental research and public health* 2018; 15(9).
114. Park, Su Hyun, Duncan, Dustin T., Shahawy, Omar El, Lee, Lily, Shearston, Jenni A., Tamura, Kosuke, Sherman, Scott E., and Weitzman, Michael Characteristics of Adults Who Switched From Cigarette Smoking to E-cigarettes. *American journal of preventive medicine* 2017; 53(5):652-660.
115. Mantey, Dale S., Cooper, Maria R., Loukas, Alexandra, and Perry, Cheryl L. E-cigarette Use and Cigarette Smoking Cessation among Texas College Students. *American journal of health behavior* 2017; 41(6):750-759.
116. Carroll, Dana Mowls, Wagener, Theodore L., Thompson, David M., Stephens, Lancer D., Peck, Jennifer D., Campbell, Janis E., and Beebe, Laura A. Electronic nicotine delivery system use behaviour and loss of autonomy among American Indians: results from an observational study. *BMJ open* 2017; 7(12):e018469-.
117. Johnson, Matthew W., Johnson, Patrick S., Rass, Olga, and Pacek, Lauren R. Behavioral economic substitutability of e-cigarettes, tobacco cigarettes, and nicotine gum. *Journal of psychopharmacology* 2017; 31(7):851-860.
118. Zhuang, Yue Lin, Cummins, Sharon E., Sun, Jessica Y., and Zhu, Shu Hong Long-term e-cigarette use and smoking cessation: a longitudinal study with US population. *Tobacco control* 2016; 25(Suppl 1):i90-i95.
119. Filippidis, Filippos T., Laverty, Anthony A., Gerovasili, Vasiliki, and Vardavas, Constantine I. Two-year trends and predictors of e-cigarette use in 27 European Union member states. *Tobacco control* 2017; 26(1):98-104.
120. Chamberlain, Catherine, Perlen, Susan, Brennan, Sue, Rychetnik, Lucie, Thomas, David, Maddox, Raglan, Alam, Noore, Banks, Emily, Wilson, Andrew, and Eades, Sandra Evidence for a comprehensive approach to Aboriginal tobacco control to maintain the decline in smoking: an overview of reviews among Indigenous peoples. *Systematic reviews* 2017; 6(1):135-.
121. Kaisar, Mohammad A., Villalba, Heidi, Prasad, Shikha, Liles, Taylor, Sifat, Ali Ehsan, Sajja, Ravi K., Abbruscato, Thomas J., and Cucullo, Luca Offsetting the impact of smoking and e-cigarette vaping on the cerebrovascular system and stroke injury: Is Metformin a viable countermeasure? *Redox biology* 2017; 13:353-362.
122. Hatsukami, Dorothy K., Zaatari, Ghazi, and Donny, Eric The case for the WHO Advisory Note, Global Nicotine Reduction Strategy. *Tobacco control* 2017; 26(e1):e29-e30.
123. Allem, Jon Patrick and Ferrara, Emilio The Importance of Debiasing Social Media Data to Better Understand E-Cigarette-Related Attitudes and Behaviors. *Journal of medical Internet research* 2016; 18(8):e219-.
124. Soule, Eric K., Rosas, Scott R., and Nasim, Aashir Reasons for electronic cigarette use beyond cigarette smoking cessation: A concept mapping approach. *Addictive behaviors* 2016; 56:41-50.
125. Eichler, Martin, Blettner, Maria, and Singer, Susanne The Use of E-Cigarettes. *Deutsches Arzteblatt international* 2016; 113(50):847-854.
126. Wills, Thomas A. and Sargent, James D. Do E-cigarettes reduce smoking? *Preventive medicine* 2017; 100:285-286.
127. Pratt, Sarah I., Sargent, James, Daniels, Luke, Santos, Meghan M., and Brunette, Mary Appeal of electronic cigarettes in smokers with serious mental illness. *Addictive behaviors* 2016; 59:30-34.
128. Giovenco, Daniel P. and Delnevo, Cristine D. Prevalence of population smoking cessation by electronic cigarette use status in a national sample of recent smokers. *Addictive behaviors* 2018; 76:129-134.
129. Curry, Elana, Nemeth, Julianna M., Wermert, Amy, Conroy, Sara, Shoben, Abigail, Ferketich, Amy K., and Wewers, Mary Ellen A Descriptive Report of Electronic Cigarette Use After Participation in a Community-Based Tobacco Cessation Trial. *Nicotine & tobacco research* 2017; 20(1):135-139.
130. Halpern, Scott D. and Volpp, Kevin G. E-Cigarettes, Incentives, and Drugs for Smoking Cessation. *The New England journal of medicine* 2018; 379(10):992-.
131. Robinson, R. J., Hensel, E. C., Al-Olayan, A. A., Nonnemaker, J. M., and Lee, Y. O. Effect of e-liquid flavor on electronic cigarette topography and consumption behavior in a 2-week natural environment switching study. *PloS one* 2018; 13(5):e0196640-.
132. Guerrero-Cignarella, Andrea, Luna Diaz, Landy V., Balestrini, Kira, Holt, Gregory, Mirsaeidi, Mehdi, Calderon-Candelario, Rafael, Whitney, Philip, Salathe, Matthias, and Campos, Michael A. Differences in vaping topography in relation to adherence to exclusive electronic cigarette use in veterans. *PloS one* 2018; 13(4):e0195896.
133. Pourchez, Jeremie and Forest, Valerie E-cigarettes: from nicotine to cannabinoids, the French situation. *The Lancet.Respiratory medicine* 2018; 6(5):e16-.
134. Li Volti, Giovanni, Polosa, Riccardo, and Caruso, Massimo Assessment of E-cigarette impact on smokers: The importance of experimental conditions relevant to human consumption. *Proceedings of the National Academy of Sciences of the United States of America* 2018; 115(14):E3073-E3074.
135. Truman, Penelope, Gilmour, Moira, and Robinson, Geoffrey Acceptability of electronic cigarettes as an option to replace tobacco smoking for alcoholics admitted to hospital for detoxification. *The New Zealand medical journal* 2018; 131(1470):22-28.
136. Pisinger, Charlotta, Jorgensen, Torben, and Toft, Ulla A multifactorial approach to explaining the stagnation in national smoking rates. *Danish medical journal* 2018; 65(2).
137. Harris, Curtis C. Tobacco smoking, E-cigarettes, and nicotine harm. *Proceedings of the National Academy of Sciences of the United States of America* 2018; 115(7):1406-1407.
138. Fairchild, Amy L., Lee, Ju Sung, Bayer, Ronald, and Curran, James E-Cigarettes and the Harm-Reduction Continuum. *The New England journal of medicine* 2018; 378(3):216-219.
139. Walele, Tanvir, Bush, Jim, Koch, Annelize, Savioz, Rebecca, Martin, Claire, and O'Connell, Grant Evaluation of the safety profile of an electronic vapour product used for two years by smokers in a real-life setting. *Regulatory toxicology and pharmacology* 2018; 92:226-238.
140. Bareham, David, Ahmadi, Keivan, Elie, Mathieu, Jones, Arwel W., and McKee, Martin E-cigarettes: further flavours of controversy within the controversy. *The Lancet.Respiratory medicine* 2018; 6(1):16-17.
141. Flint, Stuart W. and Jones, Arwel W. The irresponsible promotion of e-cigarettes and Swaptober. *The Lancet.Respiratory medicine* 2018; 6(1):e3-e4.
142. Subialka Nowariak, Emily N., Lien, Rebecca K., Boyle, Raymond G., Amato, Michael S., and Beebe, Laura A. E-cigarette use among treatment-seeking smokers: Moderation of abstinence by use frequency. *Addictive behaviors* 2018; 77:137-142.
143. Browne, Matthew and Todd, Daniel G. Then and now: Consumption and dependence in e-cigarette users who formerly smoked cigarettes. *Addictive behaviors* 2018; 76:113-121.
144. Tousoulis, Dimitris Tobacco smoking and electronic cigarette: two sides of the same coin? *Hellenic journal of cardiology* 2017; 58(4):253-255.
145. Mishra, Sundeep Are e-cigarettes beneficial for public health: Hume's guillotine - The debate continues? *Indian heart journal* 2017; 69(6):810-813.
146. Rodu, Brad and Plurphanswat, Nantaporn Quit Methods Used by American Smokers, 2013-2014. *International journal of environmental research and public health* 2017; 14(11).
147. Burki, Talha Khan Tobacco control in the UK. *The Lancet.Oncology* 2017; 18(12):e715-.
148. Patients Should Stop e-Cigarette Use Before Plastic Surgery. *Journal of the California Dental Association* 2017; 45(2):69-.
149. Shahab, Lion, Goniewicz, Maciej L., Blount, Benjamin C., Brown, Jamie, and West, Robert E-Cigarettes and Toxin Exposure. *Annals of internal medicine* 2017; 167(7):525-526.
150. Aubin, Henri Jean, Guenel, Pascal, Boutron-Ruault, Marie Christine, Matrat, Mireille, Luquiens, Amandine, and Dupont, Patrick E-Cigarettes and Toxin Exposure. *Annals of internal medicine* 2017; 167(7):524-525.
151. Hawkes, Nigel The mixed messages that led to an e-cigarette shambles. *BMJ* 2017; 358:j4460-.
152. Young-Wolff, Kelly C., Klebaner, Daniella, Folck, Bruce, Carter-Harris, Lisa, Salloum, Ramzi G., Prochaska, Judith J., Fogelberg, Renee, and Tan, Andy S. L. Do you vape? Leveraging electronic health records to assess clinician documentation of electronic nicotine delivery system use among adolescents and adults. *Preventive medicine* 2017; 105:32-36.
153. McCarthy, Michael US plan gives greater role to electronic cigarettes in tobacco harm reduction. *BMJ (Clinical research ed.)* 2017; 358:j3689-.
154. Zhu, Shu Hong, Zhuang, Yue Lin, Wong, Shiushing, Cummins, Sharon E., and Tedeschi, Gary J. E-cigarette use and associated changes in population smoking cessation: evidence from US current population surveys. *BMJ (Clinical research ed.)* 2017; 358:j3262-.
155. Bullen, Christopher Rise in e-cigarette use linked to increase in smoking cessation rates. *BMJ* 2017; 358:j3506-.
156. Wilson, Nick, Hoek, Janet, Thomson, George, and Edwards, Richard Should e-cigarette use be included in indoor smoking bans? *Bulletin of the World Health Organization* 2017; 95(7):540-541.
157. Perkins, Kenneth A., Karelitz, Joshua L., and Michael, Valerie C. Effects of nicotine versus placebo e-cigarette use on symptom relief during initial tobacco abstinence. *Experimental and clinical psychopharmacology* 2017; 25(4):249-254.
158. Burki, Talha Khan Electronic cigarette use in the UK. *The Lancet.Oncology* 2017; 18(6):e311-.
159. The United States must act quickly to control the use of e-cigarettes. *Nature* 2017; 545(7654):265-266.
160. Pasquereau, Anne, Guignard, Romain, Andler, Raphael, and Nguyen-Thanh, Viet Electronic cigarettes, quit attempts and smoking cessation: a 6-month follow-up. *Addiction* 2017; 112(9):1620-1628.
161. Yong, Hua Hie, Hitchman, Sara C., Cummings, K. Michael, Borland, Ron, Gravely, Shannon M. L., McNeill, Ann, and Fong, Geoffrey T. Does the Regulatory Environment for E-Cigarettes Influence the Effectiveness of E-Cigarettes for Smoking Cessation? Longitudinal Findings From the ITC Four Country Survey. *Nicotine & tobacco research* 2017; 19(11):1268-1276.
162. Pierre, Sebastien, Rivera, Caroline, Le Maitre, Beatrice, Ruppert, Anne Marie, Bouaziz, Herve, Wirth, Nathalie, Saboye, Jacques, Sautet, Alain, Masquelet, Alain Charles, Tournier, Jean Jacques, Martinet, Yves, Chaput, Benoit, and Dureuil, Bertrand Guidelines on smoking management during the perioperative period. *Anaesthesia, critical care & pain medicine* 2017; 36(3):195-200.
163. Cant, A., Collard, B., and Cunliffe, D. Electronic cigarettes: Necrotic ulcer. *British dental journal* 2017; 222(4):226-.
164. Van Heel, Martijn, Van Gucht, Dinska, Vanbrabant, Koen, and Baeyens, Frank The Importance of Conditioned Stimuli in Cigarette and E-Cigarette Craving Reduction by E-Cigarettes. *International journal of environmental research and public health* 2017; 14(2)-.
165. Gorski, Pawel Are e-cigarettes good or bad? *Advances in respiratory medicine* 2017; 85(1):1-2.
166. Reynolds, C. M. E., Egan, B., McKeating, A., Daly, N., Sheehan, S. R., and Turner, M. J. Five year trends in maternal smoking behaviour reported at the first prenatal appointment. *Irish journal of medical science* 2017; 186(4):971-979.
167. Dautzenberg, B., Adler, M., Garelik, D., Loubrieu, J. F., Mathern, G., Peiffer, G., Perriot, J., Rouquet, R. M., Schmitt, A., Underner, M., and Urban, T. Practical guidelines on e-cigarettes for practitioners and others health professionals. A French 2016 expert's statement. *Revue des maladies respiratoires* 2017; 34(2):155-164.
168. Zablotsky, Nevin Electronic cigarette hazards. *Journal of the American Dental Association* 2017; 148(2):60-.
169. Brikmanis, Kristin, Petersen, Angela, and Doran, Neal E-cigarette use, perceptions, and cigarette smoking intentions in a community sample of young adult nondaily cigarette smokers. *Psychology of addictive behaviors* 2017; 31(3):336-342.
170. Holliday, R., Preshaw, P., and Bauld, L. Smoking cessation: The role of e-cigarettes. *British dental journal* 2017; 222(1):3-.
171. O'Gara, Erin, Sharma, Eva, Boyle, Raymond G., and Taylor, Kristie A. Exploring Exclusive and Poly-tobacco Use among Adult Cigarette Smokers in Minnesota. *American journal of health behavior* 2017; 41(1):84-91.
172. Safety and Efficacy of Electronic Cigarettes: Update for the Clinical Nurse Specialist. *Clinical nurse specialist CNS* 2017; 31(1):E10-.
173. Huerta, Timothy R., Walker, Daniel M., Mullen, Deborah, Johnson, Tyler J., and Ford, Eric W. Trends in E-Cigarette Awareness and Perceived Harmfulness in the U.S. *American journal of preventive medicine* 2017; 52(3):339-346.
174. Ruhe, Melanie, Haller-Stevenson, Erica, Roulston, Katherine, and Jourdan, Megan Strengthening the Capacity of Local Health Departments to Reduce Exposure to Electronic Nicotine Delivery Systems. *Journal of public health management and practice* 2017; 23(1):93-94.
175. Amato, Michael S., Boyle, Raymond G., and Levy, David E-cigarette use 1 year later in a population-based prospective cohort. *Tobacco control* 2017; 26(e2):e92-e96.
176. Glover, Marewa, Breier, Bernhard H., and Bauld, Linda Could Vaping be a New Weapon in the Battle of the Bulge? *Nicotine & tobacco research* 2017; 19(12):1536-1540.
177. Wagener, Theodore L., Floyd, Evan L., Stepanov, Irina, Driskill, Leslie M., Frank, Summer G., Meier, Ellen, Leavens, Eleanor L., Tackett, Alayna P., Molina, Neil, and Queimado, Lurdes Have combustible cigarettes met their match? The nicotine delivery profiles and harmful constituent exposures of second-generation and third-generation electronic cigarette users. *Tobacco control* 2017; 26(e1):e23-e28.
178. Soneji, Samir, Primack, Brian A., Pierce, John P., Sung, Hai Yen, and Sargent, James D. Re: Modeling the Effects of E-cigarettes on Smoking Behavior: Implications for Future Adult Smoking Prevalence. *Epidemiology (Cambridge, Mass.)* 2017; 28(1):e1-.
179. Zawertailo, Laurie, Pavlov, Dmytro, Ivanova, Anna, Ng, Ginnie, Baliunas, Dolly, and Selby, Peter Concurrent E-Cigarette Use During Tobacco Dependence Treatment in Primary Care Settings: Association With Smoking Cessation at Three and Six Months. *Nicotine & tobacco research* 2017; 19(2):183-189.
180. Goniewicz, Maciej L., Gawron, Michal, Smith, Danielle M., Peng, Margaret, Jacob, Peyton, and Benowitz, Neal L. Exposure to Nicotine and Selected Toxicants in Cigarette Smokers Who Switched to Electronic Cigarettes: A Longitudinal Within-Subjects Observational Study. *Nicotine & tobacco research* 2017; 19(2):160-167.
181. Manzoli, Lamberto, Flacco, Maria Elena, Ferrante, Margherita, La Vecchia, Carlo, Siliquini, Roberta, Ricciardi, Walter, Marzuillo, Carolina, Villari, Paolo, Fiore, Maria, and ISLESE Working Group Cohort study of electronic cigarette use: effectiveness and safety at 24 months. *Tobacco control* 2017; 26(3):284-292.
182. Gartner, Coral The potential impact of vaporized nicotine products on vulnerable subpopulations. *Addiction* 2017; 112(1):18-19.
183. Vickerman, K. A., Schauer, G. L., Malarcher, A. M., Zhang, L., Mowery, P., and Nash, C. M. Reasons for Electronic Nicotine Delivery System use and smoking abstinence at 6 months: a descriptive study of callers to employer and health plan-sponsored quitlines. *Tobacco control* 2017; 26(2):126-134.
184. Study finds that cash is effective, but e-cigarettes are not in helping smokers quit. *Cancer* 2018; 124(18):3632-3633.
185. Hinton, Alice, Nagaraja, Haikady N., Cooper, Sarah, and Wewers, Mary Ellen Tobacco product transition patterns in rural and urban cohorts: Where do dual users go? *Preventive medicine reports* 2018; 12:241-244.
186. Nocella, Cristina, Biondi-Zoccai, Giuseppe, Sciarretta, Sebastiano, Peruzzi, Mariangela, Pagano, Francesca, Loffredo, Lorenzo, Pignatelli, Pasquale, Bullen, Chris, Frati, Giacomo, and Carnevale, Roberto Impact of Tobacco Versus Electronic Cigarette Smoking on Platelet Function. *The American journal of cardiology* 2018; 122(9):1477-1481.
187. Benmarhnia, Tarik, Pierce, John P., Leas, Eric, White, Martha M., Strong, David R., Noble, Madison L., and Trinidad, Dennis R. Can E-Cigarettes and Pharmaceutical Aids Increase Smoking Cessation and Reduce Cigarette Consumption? Findings From a Nationally Representative Cohort of American Smokers. *American journal of epidemiology* 2018; 187(11):2397-2404.
188. Motooka, Yumi, Matsui, Toshinobu, Slaton, Rachel M., Umetsu, Ryogo, Fukuda, Akiho, Naganuma, Misa, Hasegawa, Shiori, Sasaoka, Sayaka, Hatahira, Haruna, Iguchi, Kazuhiro, and Nakamura, Mitsuhiro Adverse events of smoking cessation treatments (nicotine replacement therapy and non-nicotine prescription medication) and electronic cigarettes in the Food and Drug Administration Adverse Event Reporting System, 2004-2016. *SAGE open medicine* 2018; 6:2050312118777953-.
189. Use of E-Cigarettes Among Smokers Who Plan to Quit After a Hospitalization. *Annals of internal medicine* 2018; 168(9).
190. Primack, Brian A., Shensa, Ariel, Sidani, Jaime E., Hoffman, Beth L., Soneji, Samir, Sargent, James D., Hoffman, Robert M., and Fine, Michael J. Initiation of Traditional Cigarette Smoking after Electronic Cigarette Use Among Tobacco-Naive US Young Adults. *The American journal of medicine* 2018; 131(4):443-443.
191. Selya, Arielle S., Dierker, Lisa, Rose, Jennifer S., Hedeker, Donald, and Mermelstein, Robin J. The Role of Nicotine Dependence in E-Cigarettes' Potential for Smoking Reduction. *Nicotine & tobacco research* 2018; 20(10):1272-1277.
192. Etter, Jean Francois Electronic Cigarette: A Longitudinal Study of Regular Vapers. *Nicotine & tobacco research* 2018; 20(8):912-922.
193. Smiley, Sabrina L., DeAtley, Teresa, Rubin, Leslie F., Harvey, Emily, Kierstead, Elexis C., Webb Hooper, Monica, Niaura, Raymond S., Abrams, David B., and Pearson, Jennifer L. Early Subjective Sensory Experiences with "Cigalike" E-cigarettes Among African American Menthol Smokers: A Qualitative Study. *Nicotine & tobacco research* 2018; 20(9):1069-1075.
194. Campagna, Davide, Amaradio, Maria Domenica, Sands, Mark F., and Polosa, Riccardo Respiratory infections and pneumonia: potential benefits of switching from smoking to vaping. *Pneumonia* 2016; 8:4-.
195. E-cigarettes associated with higher rate of smoking cessation. *Nursing standard (Royal College of Nursing (Great Britain) : 1987)* 2016; 31(6):16-.
196. England, Lucinda J., Tong, Van T., Koblitz, Amber, Kish-Doto, Julia, Lynch, Molly M., and Southwell, Brian G. Perceptions of emerging tobacco products and nicotine replacement therapy among pregnant women and women planning a pregnancy. *Preventive medicine reports* 2016; 4:481-485.
197. E-cigarettes are a useful tool in getting people to stop smoking. *Nursing standard (Royal College of Nursing* 2016; 30(39):14-.
198. Dinakar, Chitra and O'Connor, George T. The Health Effects of Electronic Cigarettes. *The New England journal of medicine* 2016; 375(26):2608-2609.
199. Tatullo, Marco, Gentile, Stefano, Paduano, Francesco, Santacroce, Luigi, and Marrelli, Massimo Crosstalk between oral and general health status in e-smokers. *Medicine* 2016; 95(49):e5589-.
200. Camenga, Deepa E-cigarette use associated with tobacco smoking. *The Journal of pediatrics* 2016; 178:304-.
201. Shi, Yuyan, Pierce, John P., White, Martha, Vijayaraghavan, Maya, Compton, Wilson, Conway, Kevin, Hartman, Anne M., and Messer, Karen E-cigarette use and smoking reduction or cessation in the 2010/2011 TUS-CPS longitudinal cohort. *BMC public health* 2016; 16(1):1105-.
202. Bareham, David, Ahmadi, Keivan, Elie, Mathieu, and Jones, Arwel W. E-cigarettes: controversies within the controversy. *The Lancet.Respiratory medicine* 2016; 4(11):868-869.
203. Fong, Amy Should Health Care Providers Support Electronic Cigarettes for Smoking Cessation? *The American journal of nursing* 2016; 116(10):11-.
204. Dunlop, Sally, Dessaix, Anita, and Currow, David How are tobacco smokers using e-cigarettes? Patterns of use, reasons for use and places of purchase in New South Wales. *The Medical journal of Australia* 2016; 205(7):336-.
205. Mendelsohn, Colin P. How are tobacco smokers using e-cigarettes? Patterns of use, reasons for use and places of purchase in New South Wales. *The Medical journal of Australia* 2016; 205(7):335-336.
206. Potential quitters turn to e-cigarettes. *British dental journal* 2016; 221(6):284-.
207. Hershberger, Alexandra R., VanderVeen, J. Davis, Karyadi, Kenny A., and Cyders, Melissa A. Transitioning From Cigarettes to Electronic Cigarettes Increases Alcohol Consumption. *Substance use & misuse* 2016; 51(14):1838-1845.
208. Litt, Mark D., Duffy, Valerie, and Oncken, Cheryl Cigarette smoking and electronic cigarette vaping patterns as a function of e-cigarette flavourings. *Tobacco control* 2016; 25(Suppl 2):ii67-ii72.
209. Gulland, Anne E-cigarettes help smokers quit, Cochrane review confirms. *BMJ* 2016; 354:i4993-.
210. Beard, Emma, West, Robert, Michie, Susan, and Brown, Jamie Association between electronic cigarette use and changes in quit attempts, success of quit attempts, use of smoking cessation pharmacotherapy, and use of stop smoking services in England: time series analysis of population trends. *BMJ* 2016; 354:i4645-.
211. Brown-Johnson, Cati G., Burbank, Andrea, Daza, Eric J., Wassmann, Arianna, Chieng, Amy, Rutledge, Geoffrey W., and Prochaska, Judith J. Online Patient-Provider E-cigarette Consultations: Perceptions of Safety and Harm. *American journal of preventive medicine* 2016; 51(6):882-889.
212. Green, Sharon H., Bayer, Ronald, and Fairchild, Amy L. Evidence, Policy, and E-Cigarettes. *The New England journal of medicine* 2016; 375(5):e6-.
213. Hickner, John What do we really know about e-cigarettes? *The Journal of family practice* 2016; 65(6):372-.
214. E-cigs 'could reduce deaths from cigarette smoking'. *Community practitioner* 2016; 89(6):8.
215. Abbasi, Jennifer FDA Extends Authority to e-Cigarettes: Implications for Smoking Cessation? *JAMA* 2016; 316(6):572-574.
216. Meernik, Clare and Goldstein, Adam O. Should Clinicians Recommend E-cigarettes to Their Patients Who Smoke? No. *Annals of family medicine* 2016; 14(4):302-303.
217. McNeill, Ann Should Clinicians Recommend E-cigarettes to Their Patients Who Smoke? Yes. *Annals of family medicine* 2016; 14(4):300-301.
218. McCarthy, Michael US study adds to debate over whether e-cigarettes lead to smoking in young people. *BMJ* 2016; 353:i3321-.
219. Yeh, James S., Bullen, Christopher, and Glantz, Stanton A. CLINICAL DECISIONS. E-Cigarettes and Smoking Cessation. *The New England journal of medicine* 2016; 374(22):2172-2174.
220. Cagney, Hannah E-cigarettes classified as tobacco products in the USA. *The Lancet Respiratory medicine* 2016; 4(6):437-.
221. McKee, Martin, Daube, Mike, and Chapman, Simon E-cigarettes should be regulated. *The Medical journal of Australia* 2016; 204(9):331-.
222. Orellana-Barrios, Menfil Andres, Payne, Drew, and Nugent, Kenneth E-cigarettes and smoking cessation. *The Lancet Respiratory medicine* 2016; 4(6):e24-.
223. Ta, Yinting, Bhowmik, Angshu, and Jose, Ricardo J. E-cigarettes and smoking cessation. *The Lancet Respiratory medicine* 2016; 4(6):e25-.
224. Hajek, Peter, McRobbie, Hayden, and Bullen, Chris E-cigarettes and smoking cessation. *The Lancet Respiratory medicine* 2016; 4(6):e23-.
225. Torjesen, Ingrid E-cigarettes are a "gateway from smoking," RCP concludes. *BMJ* 2016; 353:i2392-.
226. Green, Sharon H., Bayer, Ronald, and Fairchild, Amy L. Evidence, Policy, and E-Cigarettes--Will England Reframe the Debate? *The New England journal of medicine* 2016; 374(14):1301-1303.
227. Gartner, Coral and Hall, Wayne Assessing the place of nicotine vaporisers in tobacco control. *Tobacco control* 2016; 25(e1):e1-e2.
228. Wagoner, Kimberly G., Cornacchione, Jennifer, Wiseman, Kimberly D., Teal, Randall, Moracco, Kathryn E., and Sutfin, Erin L. E-cigarettes, Hookah Pens and Vapes: Adolescent and Young Adult Perceptions of Electronic Nicotine Delivery Systems. *Nicotine & tobacco research* 2016; 18(10):2006-2012.
229. Polosa, Riccardo, Morjaria, Jaymin B., Caponnetto, Pasquale, Caruso, Massimo, Campagna, Davide, Amaradio, Maria Domenica, Ciampi, Giovanni, Russo, Cristina, and Fisichella, Alfredo Persisting long term benefits of smoking abstinence and reduction in asthmatic smokers who have switched to electronic cigarettes. *Discovery medicine* 2016; 21(114):99-108.
230. Bond, Kristin and Nunes, Natalie Electronic Cigarettes for Smoking Cessation. *American family physician* 2016; 93(6):492-.
231. James, Shirley A., Meier, Ellen M., Wagener, Theodore L., Smith, Katherine M., Neas, Barbara R., and Beebe, Laura A. E-Cigarettes for Immediate Smoking Substitution in Women Diagnosed with Cervical Dysplasia and Associated Disorders. *International journal of environmental research and public health* 2016; 13(3).
232. Wise, Jacqui E-cigarettes help up to 22,000 smokers in England quit each year. *BMJ* 2016; 352:i1243-.
233. Bui, Quynh Electronic Cigarettes for Smoking Cessation. *American family physician* 2016; 93(3):178-179.
234. Laugesen, Murray and Grace, Randolph C. Excise, electronic cigarettes and nicotine reduction to reduce smoking prevalence in New Zealand by 2025. *The New Zealand medical journal* 2016; 129(1430):94-95.
235. Braillon, Alain Electronic Nicotine Delivery Systems, From Both Sides of the Atlantic. *Pediatrics* 2016; 137(3):e20154356-.
236. Wells, Eden V. E-cigarettes: A Physician's Dilemma. *Michigan medicine* 2016; 115(1):8-9.
237. Nolan, Margaret, Leischow, Scott, Croghan, Ivana, Kadimpati, Sandeep, Hanson, Andrew, Schroeder, Darrell, and Warner, David O. Feasibility of Electronic Nicotine Delivery Systems in Surgical Patients. *Nicotine & tobacco research* 2016; 18(8):1757-1762.
238. Auf, Rehab, Trepka, Mary Jo, Cano, Miguel Angel, De La Rosa, Mario, Selim, Marah, and Bastida, Elena Electronic cigarettes: the renormalisation of nicotine use. *BMJ* 2016; 352:i425-.
239. Clancy, L. and Babineau, K. E-cigarettes: effective cessation tools or public health threat? *QJM* 2016; 109(2):79-81.
240. Zablotsky, Nevin Electronic Cigarettes. *Journal of the American Dental Association (1939)* 2016; 147(2):80-81.
241. Etter, Jean Francois A longitudinal study of cotinine in long-term daily users of e-cigarettes. *Drug and alcohol dependence* 2016; 160:218-221.
242. Cross, Elfriede, Garrison, Scott, and Kolber, Michael R. Electronic cigarettes: help, hurt, or hype? *Canadian family physician Medecin de famille canadien* 2016; 62(1):51-.
243. Bernstein, Steven L. Electronic cigarettes: more light, less heat needed. *The Lancet Respiratory medicine* 2016; 4(2):85-87.
244. Mendelsohn, Colin P. Electronic cigarettes: what can we learn from the UK experience? *The Medical journal of Australia* 2016; 204(1):14-15.
245. Gmel, Gerhard, Baggio, Stephanie, Mohler-Kuo, Meichun, Daeppen, Jean Bernard, and Studer, Joseph E-cigarette use in young Swiss men: is vaping an effective way of reducing or quitting smoking? *Swiss medical weekly* 2016; 146:w14271-.
246. Chong, C., Rahman, A., Loonat, K., Sagar, R. C., and Selinger, C. P. Current smoking habits in British IBD patients in the age of e-cigarettes. *BMJ Open Gastroenterology* 2019; 6(1):e000309-.
247. Balbo, S. and Stepanov, I. The Wild West of E-Cigarettes. *Chemical Research in Toxicology* 2018; 31(9):823-824.
248. de Oliveira, G. M. M., Mendes, M., Dutra, O. P., Achutti, A., Fernandes, M., Azevedo, V., Ferreira, M. B. S. E., Coelho, A. S., Soares, M. B. P. C., Evora, M. A. B. L., Mariotto, M. G., and Morais, J. A. 2019: Recommendations for reducing tobacco consumption in Portuguese-speaking countries-positioning of the federation of Portuguese language cardiology societies. *Arquivos Brasileiros de Cardiologia* 2019; 112(4):477-486.
249. The, Lancet Smoke and mirrors: new tobacco products and Formula 1. *The Lancet* 2019; 393(10185):2010-.
250. Wylie, C., Heffernan, A., Brown, J. A., Cairns, R., Lynch, A.-M., and Robinson, J. Exposures to e-cigarettes and their refills: calls to Australian Poisons Information Centres, 2009-2016. *Medical Journal of Australia* 2019; 210(3):126-.
251. Enlighten e-cigarettes. *Nature medicine* 2019; 25(4):531-.
252. Siddiqui, F., Mishu, M., Marshall, A.-M., and Siddiqi, K. E-cigarette use and subsequent smoking in adolescents and young adults: a perspective. *Expert review of respiratory medicine* 2019; 13(5):403-405.
253. Hickey, S., Goverman, J., Friedstat, J., Sheridan, R., and Schulz, J. Thermal injuries from exploding electronic cigarettes. *Burns* 2018; 44(5):1294-1301.
254. Simpson, L. J. and Lye, G. Burns injuries from e-cigarettes kept in pockets. *BMJ* 2019; 364:554-.
255. Hopkinson, N. S. The prominence of e-cigarettes is a symptom of decades of failure to tackle smoking properly. *BMJ* 2019; 364:l647-.
256. Braillon, A. E-cigarettes and the Youngest, Not a Problem in Europe: No Data Yet. *American journal of preventive medicine* 2018; 54(4):e79-.
257. Taylor, G. M. J. and Munafo, M. R. Does smoking cause poor mental health? *The Lancet Psychiatry* 2019; 6(1):2-3.
258. Sly, J. R., Miller, S. J., Li, Y., Bolutayo, K., and Jandorf, L. Low-dose computed tomography lung cancer screening as a teachable moment for smoking cessation among African American smokers: A feasibility study. *Journal of Psychosocial Oncology* 2018; 36(6):784-792.
259. Bozier, J., Rutting, S., Xenaki, D., Peters, M., Adcock, I., and Oliver, B. G. Heightened response to e-cigarettes in COPD. *ERJ Open Research* 2019; 5(1):00192-02018.
260. Brown, E. S. and Green, A. I. Tobacco Use, Medical Illness, and Service Utilization. *Journal of dual diagnosis* 2018; 14(1):1-.
261. Versella, M. V., Borges, A. M., Lin, C., and Leyro, T. M. Co-use of Electronic Nicotine Delivery Systems and Combustible Cigarettes, and Their Association with Internalizing Pathology and Vulnerabilities. *Cognitive Therapy and Research* 2019; 43(1):114-120.
262. Pierce, J. P. Helping smokers to quit. *BMJ*  2018; 361:k2806-.
263. Smokers who use e-cigarettes no more likely to quit in the long term, study finds. *Clinical Pharmacist* 2019; 11(2).
264. Fetterman, J. L. and Hamburg, N. M. A cautionary note on electronic cigarettes and vascular health. *Vascular Medicine* 2018; 23(5):426-427.
265. Douglas, C. E., Henson, R., Drope, J., and Wender, R. C. The American Cancer Society public health statement on eliminating combustible tobacco use in the United States. *CA Cancer Journal for Clinicians* 2018; 68(4):240-245.
266. Hepworth, S. and Fell, G. Polarising the e-cigarette debate confuses people. *BMJ* 2018; 361:k2282-.
267. Evans, C. M., Dickey, B. F., and Schwartz, D. A. E-cigarettes: Mucus measurements make marks. *American journal of respiratory and critical care medicine* 2018; 197(4):420-422.
268. Nagelhout, G. E., Popova, L., and Kuipers, M. A. G. Why are new tobacco control interventions needed? *International journal of environmental research and public health* 2018; 15(4):658-.
269. Flannery, J. S. Disregarding the impact of nicotine on the developing brain when evaluating costs and benefits of noncombustible nicotine products. *Preventive medicine* 2019; 120:157-.
270. Kotz, D., Bockmann, M., and Kastaun, S. The use of tobacco, e-cigarettes, and methods to quit smoking in Germany - A representative study using 6 waves of data over 12 months (the DEBRA study). *Deutsches Arzteblatt international* 2018; 115(14):235-242.
271. Shorrock, P. and Bakerly, N. Effects of smoking on health and anaesthesia. *Anaesthesia and Intensive Care Medicine* 2019; 20(2):95-97.
272. Harhay, M. O., Troxel, A. B., Brophy, C., Saulsgiver, K., Volpp, K. G., and Halpern, S. D. Financial incentives promote smoking cessation directly, not by increasing use of cessation AIDS. *Annals of the American Thoracic Society* 2019; 16(2):280-282.
273. Kilibarda, B., Krstev, S., Milovanovic, M., and Foley, K. E-cigarette use in Serbia: Prevalence, reasons for trying and perceptions. *Addictive behaviors* 2019; 91:61-67.
274. Yingst, J., Foulds, J., Veldheer, S., and Du, P. Device characteristics of long term electronic cigarette users: A follow-up study. *Addictive behaviors* 2019; 91:238-243.
275. Cummings, K. M. and Polosa, R. E-Cigarette and COPD: Unreliable Conclusion About Health Risks. *Journal of general internal medicine* 2018; 33(6):784-785.
276. Aveyard, P., Arnott, D., and Johnson, K. C. Should we recommend e-cigarettes to help smokers quit? *BMJ (Online)* 2018; 361:k1759-.
277. Foundation funding to prevent Smoking. *Health Affairs* 2017; 36(8):1515.
278. Thomas, S. P., Chow, C., and Thiagalingam, A. Regulation of e-cigarettes. *Internal medicine journal* 2018; 48(10):1279-.
279. Merrill, J. K., Alberg, A. J., Goffin, J. R., Ramalingam, S. S., Simmons, V. N., and Warren, G. W. American society of clinical oncology policy brief: FDA's regulation of electronic nicotine delivery systems and tobacco products. *Journal of Oncology Practice* 2017; 13(1):58-60.
280. Middlekauff, H. R. and Gornbein, J. Association of Electronic Cigarette Use With Myocardial Infarction: Persistent Uncertainty. *American journal of preventive medicine* 2019; 56(1):159-160.
281. Etter, J.-F. E-cigarettes and the obsolescence of combustion. *Expert review of respiratory medicine* 2018; 12(5):345-347.
282. Buu, A., Hu, Y.-H., Piper, M. E., and Lin, H.-C. The association between e-cigarette use characteristics and combustible cigarette consumption and dependence symptoms: Results from a national longitudinal study. *Addictive behaviors* 2018; 84:69-74.
283. Koval, R., Willett, J., and Briggs, J. Potential Benefits and Risks of High-Nicotine e-Cigarettes. *Journal of the American Medical Association* 2018; 320(14):1429-1430.
284. Liu, J. and Halpern-Felsher, B. The Juul Curriculum Is Not the Jewel of Tobacco Prevention Education. *Journal of Adolescent Health* 2018; 63(5):527-528.
285. Hatsukami, D. K. Reducing Nicotine in Cigarettes to Minimally Addictive Levels: A New Frontier for Tobacco Control. *JAMA Psychiatry* 2018; 75(10):987-988.
286. Rigotti, N. A. Monitoring the rapidly changing landscape of e-cigarettes. *Annals of internal medicine* 2018; 169(7):494-495.
287. Toy, J., Dong, F., Lee, C., Zappa, D., Le, T., Archambeau, B., Culhane, J. T., and Neeki, M. M. Alarming increase in electronic nicotine delivery systems-related burn injuries: A serious unregulated public health issue. *American Journal of Emergency Medicine* 2017; 35(11):1781-1782.
288. Britton, J. Denicotinised cigarettes. *The Lancet* 2018; 392(10142):104-105.
289. The, Lancet Nicotine addiction, reduction, and smoking cessation. *The Lancet* 2017; 390(10096):716-.
290. Cox, S. and Jakes, S. Nicotine and e-cigarettes: Rethinking addiction in the context of reduced harm. *International Journal of Drug Policy* 2017; 44:84-85.
291. Lundback, B., Katsaounou, P., and Lotvall, J. The up-rise in e-cigarette use - friend or foe? *Respiratory research* 2016; 17(1):52-.
292. Ghamri, R. A. Identification of the most effective pharmaceutical products for smoking cessation: A literature review. *Journal of substance use* 2018; 23(6):670-674.
293. Wakhlu, S., Chapman, L., and Dela Cruz, A. M. Electronic cigarettes: Review of safety and use. *American Journal on Addictions* 2018; 27(4):307.
294. Heiss, C. Electronic cigarettes increase EPCs. *Atherosclerosis* 2016; 255:119-121.
295. Stacey, S. K. E-cigarettes: What you need to know. *Osteopathic Family Physician* 2018; 10(2):35-.
296. Michael, Cummings K., Morris, P. B., and Benowitz, N. L. Another article about e-cigarettes: Why should I care? *Journal of the American Heart Association* 2018; 7(14):e009944-.
297. Bursac, Z., Klesges, R. C., Little, M. A., Linde, B. D., Popova, L., Kaplan, C. M., and Talcott, G. W. The comparative effectiveness of two brief tobacco interventions in the U.S. Air Force: Perceived harm and intentions-to-use of tobacco products. *Tobacco induced diseases* 2018; 16(June):26-.
298. Reiner, Z. The importance of smoking cessation in patients with coronary heart disease. *International Journal of Cardiology* 2018; 258:26-27.
299. Garza, A. Smoking cessation: Where to begin? *Pharmacy Times* 2018; 2018(April).
300. Rigotti, N. A. Balancing the benefits and harms of E-cigarettes: A national academies of science, engineering, and medicine report. *Annals of internal medicine* 2018; 168(9):666-667.
301. Braillon, A. E-Cigarette, a Shipwreck! a Scandal? *American Journal of Medicine* 2018; 131(5):e225-.
302. Hirschhorn, N. Another perspective on the Foundation for a Smoke-Free World. *The Lancet* 2018; 391(10115):25-.
303. Greenberg, A. and Jose, R. J. Public Health England prematurely endorses e-cigarettes. *BMJ* 2018; 360:k1262-.
304. Barry, A. R. Do the benefits of electronic cigarettes outweigh the risks?: The "cON" side. *Canadian Journal of Hospital Pharmacy* 2018; 71(1):45-47.
305. Bond, C. M. Do the benefits of electronic cigarettes outweigh the risks?: The "pRO" side. *Canadian Journal of Hospital Pharmacy* 2018; 71(1):44-45.
306. Jiang, N., Lee, L., Zelikoff, J. T., and Weitzman, M. E-Cigarettes: Effects on the fetus. *Pediatrics in Review* 2018; 39(3):156-158.
307. Dyer, O. E-cigarettes are beneficial in short term but longer forecast is uncertain, landmark US report finds. *BMJ (Online)* 2018; 360:k355-.
308. Greener, M. E-cigarettes: making healthcare professionals myth busters. *Prescriber* 2018; 29(4):20-24.
309. Ikonomidis, I., Vlastos, D., Kostelli, G., Kourea, K., Kondylopoulou, O., Vlachos, S., Benas, D., Varoudi, M., Pavlidis, G., Dede, V., Triantafyllidi, H., Andreadou, I., and Lekakis, J. Electronic cigarette smoking increases of arterial stifness and oxidative stress to a lesser extent than a single normal cigarette: An acute and chronic study. *European Heart Journal* 2017; 38(Supplement 1):1072-.
310. Boas, Z., Gupta, P., Moheimani, R., Bhetraratana, M., Yin, F., Peters, K., Gornbein, J., Araujo, J., Czernin, J., and Middlekauff, H. Activation of the "splenocardiac axis" by electronic and tobacco cigarettes in otherwise healthy young adults. *Circulation* 2017; 136(Supplement 1).
311. Delnevo, C. D., Villanti, A. C., Wackowski, O. A., Gundersen, D. A., and Giovenco, D. P. The influence of menthol, e-cigarettes and other tobacco products on young adults' self-reported changes in past year smoking. *Tobacco control* 2016; 25(5):571-574.
312. Moreno, M. A. What parents need to know about electronic cigarettes. *JAMA pediatrics* 2017; 171(12):1236-.
313. Glantz, S. A. Need for examination of broader range of risks when predicting the effects of new tobacco products. *Nicotine and Tobacco Research* 2017; 19(2):266-267.
314. McRobbie, H. Modelling the population health effects of e-cigarettes use: Current data can help guide future policy decisions. *Nicotine and Tobacco Research* 2017; 19(2):131-132.
315. Bruijnzeel, W. Reducing the prevalence of smoking: Policy measures and focusing on specific populations. *Nicotine and Tobacco Research* 2017; 19(9):1003-1004.
316. Printz, C. Global tobacco problem far from solved, new report indicates: Study shows the need for progress in smoking prevention and cessation despite reductions in prevalence. *Cancer* 2017; 123(20):3869-3870.
317. Anderson, H., Richie, C., and Bernard, A. A Surprisingly Volatile Smoking Alternative: Explosion and Burns as Risks of E-Cigarette Use. *Journal of Burn Care and Research* 2017; 38(5):e884-.
318. Shariat, S. F. Electronic Cigarettes: A Wolf in Sheep's Clothing. *European urology* 2017; 71(6):924-925.
319. Cummings, K. M. and Carpenter, M. J. Selling smoking cessation. *The Lancet* 2017; 389(10071):768-770.
320. Gottlieb, S. and Zeller, M. A nicotine-focused framework for public health. *New England Journal of Medicine* 2017; 377(12):1111-1114.
321. Bullen, C. Rise in e-cigarette use linked to increase in smoking cessation rates: New evidence supports a liberal approach to e-cigarette regulation. *BMJ* 2017; 358:j3506-.
322. Mayor, S. Smoking prevalence falls among adults in England. *BMJ (Online)* 2017; 357:j2953.
323. Akhavan, S., Nguyen, L.-C., Chan, V., Saleh, J., and Bozic, K. J. Impact of smoking cessation counseling prior to total joint arthroplasty. *Orthopedics* 2017; 40(2):e323-e328.
324. Quitting smoking isn't easy, even with the advent of e-cigarettes. *British Journal of Psychiatry* 2017; 210(4):307-308.
325. Kooblall, M. and Nash, D. E - Cigarette - Why do we forget to document in the medical notes? *Irish Medical Journal* 2017; 110(2):521-.
326. Farsalinos, K. E. and Polosa, R. Endothelial progenitor cell release is usually considered a beneficial effect: Problems in interpreting the acute effects of e-cigarette use. *Atherosclerosis* 2017; 258:162-163.
327. Davidson, S. M., Boldt, G., and Louie, A. V. How can we better help cancer patients quit smoking? *International Journal of Radiation Oncology Biology Physics* 2017; 99(2 Supplement 1):E393-.
328. Boulay, M.-E., Henry, C., Bosse, Y., Boulet, L.-P., and Morissette, M. C. Acute effects of electronic cigarette use on lung functions in healthy and asthmatic individuals. *American journal of respiratory and critical care medicine* 2017; 195.
329. Bartram, A., Jones, N., and Endersby, S. Lichenoid eruption associated with use of an e-cigarette. *British Journal of Oral and Maxillofacial Surgery* 2016; 54(4):475-.
330. Ganasegeran, K. and Rashid, A. Clearing the clouds-Malaysia's vape epidemic. *The Lancet Respiratory Medicine* 2016; 4(11):854-856.
331. Shapiro, S. D. and Kaynar, A. M. Electronic cigarettes: The lesser of two evils, but how much less? *Thorax* 2016; 71(12):1080-1081.
332. Arnold, C. On the vapor trail: Examining the chemical content of E-Cigarette flavorings. *Environmental health perspectives* 2016; 124(6):A115-.
333. Chague, F., Rochette, L., Gudjoncik, A., Cottin, Y., and Zeller, M. Electronic cigarettes and sports: Dangerous liaisons? *International Journal of Cardiology* 2016; 215:400-401.
334. Kassim, S. and Farsalinos, K. E. E-cigarette as a harm reduction approach among tobacco smoking khat chewers: A promising bullet of multiple gains. *International journal of environmental research and public health* 2016; 13(2).
335. Smith, L., Brar, K., Srinivasan, K., Enja, M., and Lippmann, S. E-cigarettes: How "safe" are they? *Journal of Family Practice* 2016; 65(6):380-385.
336. Rassouli, F. and Schoch, O. D. Tobacco Smoking Kills Slowly, while Smoking on Oxygen is a Burning Issue. *Respiration* 2016; 91(4):271-272.
337. Stein, M. D., Caviness, C., Grimone, K., Audet, D., Anderson, B. J., and Bailey, G. L. An open trial of electronic cigarettes for smoking cessation among methadone-maintained smokers. *Nicotine and Tobacco Research* 2016; 18(5):1157-1162.
338. Printz, C. UCSF study: E-cigarettes are not helping smokers quit. *Cancer* 2016; 122(11):1636-1637.
339. Davies, C. S. and Ismail, A. Nicotine has deleterious effects on wound healing through increased vasoconstriction. *BMJ* 2016; 353:i2709-.
340. Wagener, T. L., Meier, E., Tackett, A. P., Matheny, J. D., and Pechacek, T. F. A Proposed Collaboration Against Big Tobacco: Common Ground Between the Vaping and Public Health Community in the United States. *Nicotine and Tobacco Research* 2016; 18(5):730-736.
341. Little, M. A., Talcott, G. W., Linde, B. D., Pagano, L. A., Messler, E. C., Ebbert, J. O., and Klesges, R. C. Efficacy of a Brief Tobacco Intervention for Tobacco and Nicotine Containing Product Use in the US Air Force. *Nicotine and Tobacco Research* 2016; 18(5):1142-1149.
342. Britton, J., Arnott, D., McNeill, A., and Hopkinson, N. Nicotine without smoke-putting electronic cigarettes in context: John britton and colleagues set out why a new royal college of physicians report supports the role of electronic cigarettes as part of a comprehensive tobacco control strategy. *BMJ (Online)* 2016; 353:i1745-.
343. Garza, A. E-cigarettes: Clearing the air. *Pharmacy Times* 2016; 82(4).
344. Hartmann-Boyce, J. and Aveyard, P. Drugs for smoking cessation. *BMJ* 2016; 352:i571-.
345. Hsieh, F. H. Evidence vs advocacy in the e-cigarette debate: To vape or not to vape, that is the question. *Annals of Allergy, Asthma and Immunology* 2016; 116(2):89-90.
346. Nutt, D. J., Phillips, L. D., Balfour, D., Curran, H. V., Dockrell, M., Foulds, J., Fagerstrom, K., Letlape, K., Polosa, R., Ramsey, J., and Sweanor, D. E-cigarettes are less harmful than smoking. *The Lancet* 2016; 387(10024):1160-1162.
347. Tousoulis, D. Smoking cessation and health economics. *Hellenic Journal of Cardiology* 2016; 57(JANUARY-FEBRUARY):67-69.
348. Andalo, D. Study finds association between e-cigarettes and successful quitting. *Pharmaceutical Journal* 2016; 297(7893):140-.
349. Culverhouse, P. Unintended costs of EU e-cigarette tax hike. *Pharmaceutical Journal* 2016; 296(7889):296-.
350. Hassan, S., Anwar, M. U., Muthayya, P., and Jivan, S. Burn injuries from exploding electronic cigarette batteries: An emerging public health hazard. *Journal of Plastic, Reconstructive and Aesthetic Surgery* 2016; 69(12):1716-1718.
351. Bonnet, N. Reduction of risks linked to smoking. *Actualites Pharmaceutiques* 2016; 55(560):34-37.
352. Printz, C. "Project EASE" to follow E-cigarette smokers. *Cancer* 2016; 122(21):3259-.
353. Berg, C. D. Just say no! Smoking abstinence works. *American journal of respiratory and critical care medicine* 2016; 193(5):476-477.
354. Maloney, E. K. and Cappella, J. N. Does Vaping in E-Cigarette Advertisements Affect Tobacco Smoking Urge, Intentions, and Perceptions in Daily, Intermittent, and Former Smokers? *Health communication* 2016; 31(1):129-138.
355. Naughton, F. Daily e-cigarette use increases quit attempts and reduces smoking with no effect on cessation. *Evidence-based nursing* 2016; 19(1):18-.
356. Farquhar, B., Mark, K., Terplan, M., and Chisolm, M. S. Demystifying electronic cigarette use in pregnancy. *Journal of addiction medicine* 2015; 9(2):157-158.
357. Hrabovsky, Sharilee Myer Adult cigarette smokers: How they learn about and use electronic cigarettes. *Dissertation Abstracts International Section A: Humanities and Social Sciences* 2018; 79(12-A(E)).
358. Baldassarri, Stephen R., Hillmer, Ansel T., Anderson, Jon Mikael, Jatlow, Peter, Nabulsi, Nabeel, Labaree, David, Cosgrove, Kelly P., O'Malley, Stephanie S., Eissenberg, Thomas, Krishnan-Sarin, Suchitra, and Esterlis, Irina Use of electronic cigarettes leads to significant beta2-nicotinic acetylcholine receptor occupancy: Evidence from a PET imaging study. *Nicotine & Tobacco Research* 2018; 20(4):425-433.
359. Martinez, Diane J. What may be associated with young adult e-cigarette use? Application of the Integrated Behavior Model and affect heuristic to examine key correlates. *Dissertation Abstracts International: Section B: The Sciences and Engineering* 2018; 78(8-B(E)).
360. Sharma, Ratika, Gartner, Coral E., Castle, David J., and Mendelsohn, Colin P. Should we encourage smokers with severe mental illness to switch to electronic cigarettes? *Australian and New Zealand Journal of Psychiatry* 2017; 51(7):663-664.
361. Tracy, Derek K., Joyce, Dan W., and Shergill, Sukhwinder S. Kaleidoscope. *The British Journal of Psychiatry* 2017; 210(4):307-308.
362. Tucker, Megan R., Kivell, Bronwyn M., Laugesen, Murray, and Grace, Randolph C. Using a cigarette purchase task to assess demand for tobacco and nicotine-containing electronic cigarettes for New Zealand European and Maori/Pacific Island smokers. *New Zealand Journal of Psychology* 2017; 46(2):108-115.
363. Jones, Dina M., Majeed, Ban A., Weaver, Scott R., Sterling, Kymberle, Pechacek, Terry F., and Eriksen, Michael P. Prevalence and factors associated with smokeless tobacco use, 2014-2016. *American journal of health behavior* 2017; 41(5):608-617.
364. Spindle, Tory R., Hiler, Marzena M., Breland, Alison B., Karaoghlanian, Nareg V., Shihadeh, Alan L., and Eissenberg, Thomas The influence of a mouthpiece-based topography measurement device on electronic cigarette user's plasma nicotine concentration, heart rate, and subjective effects under directed and ad libitum use conditions. *Nicotine & Tobacco Research* 2017; 19(4):469-476.
365. Mitsuhashi-Acs, Yuki J. Personality factor correlates of smoking cessation efficacy among electronic cigarette users. *Dissertation Abstracts International: Section B: The Sciences and Engineering* 2017; 77(10-B(E)).
366. Bauld, Linda Electronic cigarettes and smoking cessation. *Nicotine & Tobacco Research* 2016; 18(10):1925-.
367. West, Robert, Shahab, Lion, and Brown, Jamie Estimating the population impact of e-cigarettes on smoking cessation in England. *Addiction* 2016; 111(6):1118-1119.
368. Branston, J. Robert and Sweanor, David Big tobacco, E-cigarettes, and a road to the smoking endgame. *International Journal of Drug Policy* 2016; 29:14-18.
369. Kassim, Saba, Al-Haboubi, Mustafa, and Croucher, Ray Short-term smoking cessation in English resident adults of Bangladeshi origin: A service review. *Nicotine & Tobacco Research* 2016; 18(4):410-415.
370. Wellman, Robert J. and O'Loughlin, Jennifer E-cigarettes: Addressing gaps in knowledge. *International journal of public health* 2016; 61(2):149-150.
371. Haddad, Ashley and Davis, Andrew M. Tobacco smoking cessation in adults and pregnant women: Behavioral and pharmacotherapy interventions. *JAMA* 2016; 315(18):2011-2012.
372. Pearson, J. L., Stanton, C. A., Cha, S., Niaura, R. S., Luta, G., and Graham, A. L. E-Cigarettes and Smoking Cessation: insights and Cautions From a Secondary Analysis of Data From a Study of Online Treatment-Seeking Smokers. *Nicotine & Tobacco Research* 2015; 17(10):1219‐1227-.
373. Rigotti, N. A., Chang, Y., Tindle, H. A., Kalkhoran, S. M., Levy, D. E., Regan, S., Kelley, J. H. K., Davis, E. M., and Singer, D. E. Association of E-cigarette use with smoking cessation among smokers who plan to quit after a hospitalization a prospective study. *Annals of internal medicine* 2018; 168(9):613‐620-.
374. NCT01782599 Electronic Cigarettes and Reactivity to Smoking Cues. [*Https://clinicaltrials.gov/show/nct01782599* 2013](Https://clinicaltrials.gov/show/nct01782599%202013).
375. ISRCTN89151172 Acute effects of electronic cigarette on lung function and airway inflammation in patients with asthma. *Http://www.who.int/trialsearch/trial2.aspx? Trialid=isrctn89151172* 2018.
376. H McRobbie, A Phillips, ML Goniewicz, KM Smith, O Knight-West, D Przulj, P Hajek Effects of Switching to Electronic Cigarettes with and without Concurrent Smoking on Exposure to Nicotine, Carbon Monoxide, and Acrolein.. *Cancer prevention research (Philadelphia, Pa.)* 2015; 8(9).
377. R Pacifici, S Pichini, S Graziano, M Pellegrini, G Massaro, F Beatrice Successful Nicotine Intake in Medical Assisted Use of E-Cigarettes: A Pilot Study.. *International journal of environmental research and public health* 2015; 12(7).
378. L Manzoli, ME Flacco, M Fiore, C La Vecchia, C Marzuillo, MR Gualano, G Liguori, G Cicolini, L Capasso, C D'Amario, S Boccia, R Siliquini, W Ricciardi, P Villari Electronic Cigarettes Efficacy and Safety at 12 Months: Cohort Study.. *PloS one* 2015; 10(6).
379. LS Brose, SC Hitchman, J Brown, R West, A McNeill Is the use of electronic cigarettes while smoking associated with smoking cessation attempts, cessation and reduced cigarette consumption? A survey with a 1-year follow-up. *Addiction* 2015; 110(7).
380. WK Al-Delaimy, MG Myers, EC Leas, DR Strong, CR Hofstetter E-cigarette use in the past and quitting behavior in the future: a population-based study. *American journal of public health* 2015; 105(6).
381. R Polosa, P Caponnetto, F Cibella, J Le-Houezec Quit and smoking reduction rates in vape shop consumers: a prospective 12-month survey. *International journal of environmental research and public health* 2015; 12(4).
382. JJ Prochaska, RA Grana E-cigarette use among smokers with serious mental illness. *PloS one* 2014; 9(11).
383. R Polosa, P Caponnetto, M Maglia, JB Morjaria, C Russo Success rates with nicotine personal vaporizers: a prospective 6-month pilot study of smokers not intending to quit. *BMC public health* 2014; 14.
384. SP Borderud, Y Li, JE Burkhalter, CE Sheffer, JS Ostroff Electronic cigarette use among patients with cancer: characteristics of electronic cigarette users and their smoking cessation outcomes. *Cancer* 2014; 120(22).
385. R Polosa, J Morjaria, P Caponnetto, M Caruso, S Strano, E Battaglia, C Russo Effect of smoking abstinence and reduction in asthmatic smokers switching to electronic cigarettes: evidence for harm reversal. *International journal of environmental research and public health* 2014; 11(5).
386. RA Grana, L Popova, PM Ling A longitudinal analysis of electronic cigarette use and smoking cessation. *JAMA internal medicine* 2014; 174(5).
387. MA Nides, SJ Leischow, M Bhatter, M Simmons Nicotine blood levels and short-term smoking reduction with an electronic nicotine delivery system. *American journal of health behavior* 2014; 38(2).
388. JF Etter, C Bullen A longitudinal study of electronic cigarette users. *Addictive behaviors* 2014; 39(2).
389. SR van Staden, M Groenewald, R Engelbrecht, PJ Becker, LT Hazelhurst Carboxyhaemoglobin levels, health and lifestyle perceptions in smokers converting from tobacco cigarettes to electronic cigarettes. *South African medical journal* 2013; 103(11).
390. P Caponnetto, R Auditore, C Russo, GC Cappello, R Polosa Impact of an electronic cigarette on smoking reduction and cessation in schizophrenic smokers: a prospective 12-month pilot study. *International journal of environmental research and public health* 2013; 10(2).
391. R Polosa, P Caponnetto, JB Morjaria, G Papale, D Campagna, C Russo Effect of an electronic nicotine delivery device (e-Cigarette) on smoking reduction and cessation: a prospective 6-month pilot study. *BMC public health* 2011; 11.
392. Ely, J Evaluation of the use of electric cigarettes in a rural smoking cessation program. *digitalunc.coalliance.org/fedora/repository/cogru:4161* 2013.
393. Hajek, P, Corbin, L, Ladmore, D, and Spearing, E Adding ecigarettes to specialist stop-smoking treatment: City of London pilot project. *Journal of Addiction Research & Therapy* 2015; 6(244).
394. Choi, K and Forster, JL Response to: Context on use is needed before public health recommendations are made about e-cigarettes. *Americal Journal of Preventive Medicine* 2019; 46(6):e58-59.
395. Canadian Partnership Against Cancer Commercial Tobacco Policy Pack.
396. The Ontario Tobacco Research Unit Research on Electronic Cigarettes and Waterpipes (RECIG-WP).
397. Trivers et al., Prevalence of Cannabis Use in Electronic Cigarettes Among US Youth.
398. Sampasa-Kanyinga et al., Use of social networking sites, electronic cigarettes, and waterpipes among adolescents.
399. Volesky et al., The influence of three e-cigarette models on indoor fine and ultrafine particulate matter concentrations under real-world conditions.
400. Richmond et al., E-cigarettes: A new hazard for children and adolescents.
401. McKelvey et al., Adolescents’ and Young Adults’ Use and Perceptions of Pod-Based Electronic Cigarettes.
402. Baskerville et al., Tobacco use cessation interventions for lesbian, gay, bisexual, transgender and queer youth and young adults: A scoping review.
403. Moheimani et al., Increased Cardiac Sympathetic Activity and Oxidative Stress in Habitual Electronic Cigarette Users Implications for Cardiovascular Risk.
404. The Ontario Tobacco Research Unit Promotion of Flavoured Vaping Products That Appeal to Youth.
405. The Ontario Tobacco Research Unit Conversations About Vaping: A Focus Group Study.
406. The Ontario Tobacco Research Unit E-Cigarette Use for Smoking Cessation: Scientific Evidence and Smokers’ Experiences.
407. The Ontario Tobacco Research Unit Youth and Young Adult Vaping in Canada.
408. The Ontario Tobacco Research Unit The Tobacco and Vaping Products Act: Implications for E-cigarettes Point-of-Sale Promotion.
409. The Ontario Tobacco Research Unit Analysis of Secondhand E-cigarette Aerosol Compounds in an Indoor Setting.
410. The Ontario Tobacco Research Unit Vaping in Ontario: Preliminary Findings from RECIG-WP.
411. The Ontario Tobacco Research Unit Monitoring and Evaluating Ontario's New E-Cigarette Policy Measures: Sales and Supply to Youth.
412. The Ontario Tobacco Research Unit Monitoring and Evaluating Ontario's New Tobacco and E-Cigarette Policy Measures.
413. Global and Public Health/Population Health/HB/ cost centre Smoke-free generation: Tobacco control plan for England.
414. Public Health England Vaping in England: an evidence update February 2019.
415. Public Health England E-cigarettes: a developing public health consensus.
416. Public Health England Tobacco control plan: delivery plan 2017 to 2022.
417. Public Health England Use of e-cigarettes in public places and workplaces.
418. Truth Initiative Behind the explosive growth of JUUL.
419. Truth Initiative 6 important facts about JUUL.
420. Truth Initiative Action needed on e-cigarettes.
421. Truth Initiative What would happen if new tobacco products ‘akin to iPhones’ were sold in the U.S.?
422. Truth Initiative Visited a vape shop? Prevalence and correlates from a national sample of U.S. young adults.
423. Truth Initiative Flavored Tobacco Product Use in Youth and Adults: Findings From the First Wave of the PATH Study (2013–2014).
424. Truth Initiative Most of JUUL's Twitter followers are underage.
425. Truth Initiative CDC data strengthens concerns over vaping teens turning to cigarettes.
426. Physicians for a Smoke Free Canada Submission on S-5, the proposed Tobacco and Vaping Products Act. (2017).
427. Canadian Cancer Society University and College 100% Smoke-Free Campuses.
428. WHO WHO TobReg: report on the scientific basis of tobacco product regulation: 6th report of a WHO study group WHO Technical report series, n. 1001.
429. WHO Electronic Nicotine Delivery Systems and Electronic Non-Nicotine Delivery Systems (ENDS/ENNDS).
430. Farsalinos et al., Carbonyl emissions from a novel heated tobacco product (IQOS): comparison with an e‐cigarette and a tobacco cigarette.
431. Pacitto et al., Characterization of airborne particles emitted by an electrically heated tobacco smoking system.
432. Farsalinos et al., Nicotine Delivery to the Aerosol of a Heat-Not-Burn Tobacco Product: Comparison With a Tobacco Cigarette and E-Cigarettes.
433. Li et al., Chemical Analysis and Simulated Pyrolysis of Tobacco Heating System 2.2 Compared to Conventional Cigarettes.
434. Mallock et al., Levels of selected analytes in the emissions of “heat not burn” tobacco products that are relevant to assess human health risks.
435. Miyazaki et al., Educational gradients in the use of electronic cigarettes and heat-not-burn tobacco products in Japan.
436. Liu et al., Heat-not-burn tobacco products: concerns from the Italian experience.
437. Tabuchi et al., Heat-not-burn tobacco product use in Japan: its prevalence, predictors and perceived symptoms from exposure to secondhand heat-not-burn tobacco aerosol.
438. Caputi et al., They’re heating up: Internet search query trends reveal significant public interest in heat-not-burn tobacco products.
439. Prodanchuk et al., Potential risk assessment of the electrically heated tobacco system (EHTS) use.
440. Bekki et al., Comparison of Chemicals in Mainstream Smoke in Heat-not-burn Tobacco and Combustion Cigarettes.
441. Stephens et al., Comparing the cancer potencies of emissions from vapourised nicotine products including e-cigarettes with those of tobacco smoke.
442. Protano et al., Second-hand smoke generated by combustion and electronic smoking devices used in real scenarios: Ultrafine particle pollution and age-related dose assessment.
443. Auer et al., Heat-Not-Burn Tobacco Cigarettes Smoke by Any Other Name.
444. Ruprecht et al., Environmental pollution and emission factors of electronic cigarettes, heat-not-burn tobacco products, and conventional cigarettes.
445. Kamada et al., Acute eosinophilic pneumonia following heat‐not‐burn cigarette smoking.
446. Moheimani et al., Increased Cardiac Sympathetic Activity and Oxidative Stress in Habitual Electronic Cigarette Users.
447. Schwartz et al., E-Cigarettes in 2017: Where do we stand?
448. Kalkhoran, S.; Chang, Y.; Rigotti, N. A. Electronic Cigarette Use and Cigarette Abstinence Over 2 Years Among U.S. Smokers in the Population Assessment of Tobacco and Health Study . 2020
449. Gomajee, R.; El-Khoury, F.; Goldberg, M.; Zins, M.; Lemogne, C.; Wiernik, E.; Lequy-Flahault, E.; Romanello, L.; Kousignian, I.; Melchior, M. Association Between Electronic Cigarette Use and Smoking Reduction in France . 2019
450. Beaglehole, R.; Bates, C.; Youdan, B.; Bonita, R. Nicotine without smoke: fighting the tobacco epidemic with harm reduction . 2019
451. Berlin, I. [Electronic cigarette: What are its benefits and risks?]. [French] . 2019
452. Bhuyan, A. Foundation backed by Philip Morris funds schoolchildren's global science contest . 2019
453. Bover Manderski, M. T.; Singh, B.; Delnevo, C. D. E-Cigarette Use and Myocardial Infarction: Importance of a Sound Evidence Base in the E-Cigarette Risks-Benefits Debate . 2019
454. Braillon, A. Electronic Cigarette Use among Populations of Women during Reproductive Years . 2019
455. Braillon, A. The Use of e-Cigarettes in Patients with Cancer - A Double Shipwreck . 2019
456. Bridgeman, M. B.; Mansukhani, R. P. OTC case studies: Respiratory health . 2019
457. Brown, J. E. H.; Gartner, C.; Carter, A. Can e-cigarettes improve the well-being of people with mental health disorders? . 2019
458. Bush, A.; Bhatt, J.; Grigg, J. E cigarettes: Tar Wars: The (Tobacco) Empire Strikes Back . 2019
459. Cadet, M. Are E-cigarettes more effective in supporting smoking cessation than nicotine-replacement therapy? . 2019
460. Carson, J. Public Health England Annual Conference 2019 . 2019
461. Ebell, M. H. e-Cigarettes More Effective Than Nicotine Replacement for Cessation of Tobacco Use in Adults . 2019
462. El-Awa, F.; Tageldin, M. A.; Prasad, V.; Al-Mulla, A.; Heydari, G.; Alebshehy, R. Lung health in the Eastern Mediterranean Region: The need to end designated smoking areas in public places . 2019
463. Gesinde, B. Community-Based Smoking Cessation Programs: A Way Forward? . 2019
464. Ghebreyesus, T. A. Progress in beating the tobacco epidemic . 2019
465. Henry, T. S.; Kanne, J. P.; Kligerman, S. J. Imaging of vaping-associated lung disease . 2019
466. Jackson, S. E.; Hill, E.; Shahab, L.; Beard, E.; Michie, S.; Brown, J. Prevalence and correlates of long-term e-cigarette and nicotine replacement therapy use: a prospective study in England . 2019
467. Jackson, S. E.; Kotz, D.; West, R.; Brown, J. Reply to 'Smoke free, but dependent on nicotine' (Karam-Hage 2019) . 2019
468. Jankowski, M.; Lawson, J. A.; Shpakou, A.; Poznanski, M.; Zielonka, T. M.; Klimatckaia, L.; Loginovich, Y.; Rachel, M.; Gereova, J.; Minarowski, L.; Naumau, I.; Kornicki, K.; Peplowska, P.; Kovalevskiy, V.; Raskiliene, A.; Bielewicz, K.; Kristufkova, Z.; Mroz, R.; Majek, P.; Skoczynski, S.; Zejda, J. E.; Brozek, G. M. Smoking Cessation and Vaping Cessation Attempts among Cigarette Smokers and E-Cigarette Users in Central and Eastern Europe . 2019
469. Jha, P. Smoking cessation and e-cigarettes in China and India . 2019
470. Kalkhoran, S.; Chang, Y.; Rigotti, N. A. E-cigarettes and Smoking Cessation in Smokers With Chronic Conditions . 2019
471. Kamerow, D. Are e-cigarettes killing people in the US? . 2019
472. Koh, H. K.; Douglas, C. E. The San Francisco Ban and the Future of e-Cigarettes . 2019
473. Kuehn, B. Evaluating e-Cigarette Lung Disease . 2019
474. Langley, T. Viewing E-cigarette Research Through a Broad Lens . 2019
475. Liu, B.; Bao, W. Electronic Cigarette Use among Populations of Women during Reproductive Years - Reply . 2019
476. Lozano, P.; Arillo-Santillan, E.; Barrientos-Gutierrez, I.; Zavala-Arciniega, L.; Reynales-Shigematsu, L. M.; Thrasher, J. F. E-cigarette use and its association with smoking reduction and cessation intentions among Mexican smokers . 2019
477. Mamudu, H. M.; Sanborn, T.; Dobbs, P. D. Electronic Nicotine Delivery Systems: Recommendations to Regulate Their Use . 2019
478. Mendes, A. Vaping for smoking cessation . 2019
479. Mensah, P. K. E-Cigarettes and the Danger They Pose to Society . 2019
480. Newton, J. N. Time for The Lancet to realign with the evidence on e-cigarettes? . 2019
481. Painter, K. Outdoor smoking: Fair or foul? . 2019
482. Parmar, C. D.; Lee, L.; Sufi, P. E-cigarette, Obesity and Bariatric Surgery: Guidelines for the Bariatric Societies . 2019
483. Piepoli, M. F. Editor's presentation . 2019
484. Piper, M. E.; Mermelstein, R.; Baker, T. B. Progress in Treating Youth Smoking: Imperative, Difficult, Slow . 2019
485. Pisinger, C.; Dagli, E.; Filippidis, F. T.; Hedman, L.; Janson, C.; Loukides, S.; Ravara, S.; Saraiva, I.; Vestbo, J. ERS and tobacco harm reduction . 2019
486. Pisinger, C.; Mackay, J. New tobacco products do not protect public health . 2019
487. Russell, C.; Haseen, F.; McKeganey, N. Factors associated with past 30-day abstinence from cigarette smoking in adult established smokers who used a JUUL vaporizer for 6 months . 2019
488. Sahu, K. K.; Lal, A.; Kumar Mishra, A.; Sahu, S. A. E-Cigarettes and Methemoglobinemia: A Wolf in Sheep's Clothing . 2019
489. Sanford, N. N.; Mahal, B. A. The Use of e-Cigarettes in Patients with Cancer - A Double Shipwreck - In Reply . 2019
490. Stoebner, A.; Le Faou, A. L.; Huteau, M. E.; Gricourt, Y.; Cuvillon, P. E-cigarette or Vaping product use Associated Lung Injury (EVALI): Health issues going beyond anaesthetic and surgical perioperative procedures . 2019
491. The, Lancet E-cigarettes: time to realign our approach? . 2019
492. The, Lancet Philip Morris International: money over morality? . 2019
493. Wu, J. C.; Rhee, J. W.; Sallam, K. Electronic Cigarettes: Where There Is Smoke There Is Disease . 2019
494. Beard, E.; Jackson, S. E.; West, R.; Kuipers, M. A. G.; Brown, J. Population-level predictors of changes in success rates of smoking quit attempts in England: a time series analysis . 2020
495. Beard, E.; West, R.; Michie, S.; Brown, J. Association of prevalence of electronic cigarette use with smoking cessation and cigarette consumption in England: a time-series analysis between 2006 and 2017 . 2020
496. Benowitz, N. L. E-cigarettes and dual nicotine replacement therapy for smoking cessation . 2020
497. Benowitz, N. L. Seizures After Vaping Nicotine in Youth: A Canary or a Red Herring? . 2020
498. Cheng, D. What's hot that the other lot got . 2020
499. Gilbody, S.; Peckham, E. What does the rise and rise of electronic nicotine delivery systems mean for mental health services? . 2020
500. Goldberg, R. J.; Lapane, K.; Lemon, S.; Hirsh, M. P. The Smoking Gun: Can We Do for Gun Control What We Are Doing to Control the Vaping and E-Cigarettes Epidemic? . 2020
501. Hadland, S. E.; Chadi, N. Through the Haze: What Clinicians Can Do to Address Youth Vaping . 2020
502. Kalkhoran, S.; Chang, Y.; Rigotti, N. A. Response to: A Source of Bias in Studies of E-cigarettes and Smoking Cessation . 2020
503. Kurti, A. N.; Bunn, J. Y.; Tang, K.; Nighbor, T.; Gaalema, D. E.; Coleman-Cowger, V.; Coleman, S. R. M.; Higgins, S. T. Impact of electronic nicotine delivery systems and other respondent characteristics on tobacco use transitions among a U.S. national sample of women of reproductive age . 2020
504. Bergen, A. W.; Do, E. K.; Chen, L. S.; David, S. P. Tobacco genomics: Complexity and translational challenges . 2018
505. Hummel, K.; Nagelhout, G. E.; Fong, G. T.; Vardavas, C. I.; Papadakis, S.; Herbec, A.; Mons, U.; van den Putte, B.; Borland, R.; Fernandez, E.; de Vries, H.; McNeill, A.; Gravely, S.; Przewozniak, K.; Kovacs, P.; Trofor, A. C.; Willemsen, M. C. Quitting activity and use of cessation assistance reported by smokers in eight European countries: Findings from the EUREST-PLUS ITC Europe Surveys . 2018
506. Isrctn Exploring the use and uptake of e-cigarettes for homeless smokers . 2018
507. Sumner, W.; Farsalinos, K. Lessons and guidance from the special issue on electronic cigarette use and public health . 2018
508. Actrn Project NEAT: nicotinE As Treatment for tobacco smoking following discharge from residential withdrawal services . 2019
509. Ajiboye, A. S.; Gordon, J. S.; Fox, C. H.; Garcia, R. I. Oral Health Effects of Tobacco Products: Science and Regulatory Policy . 2019
510. Akinboro, O.; Nwabudike, S.; Elias, R.; Balasire, O.; Ola, O.; Ostroff, J. S. Electronic Cigarette Use among Survivors of Smoking-Related Cancers in the United States . 2019
511. Aloosh, M.; Johnston, J.; Harvey, B. E-cigarettes . 2019
512. Anonymous Funders Supporting Smoking Prevention . 2019
513. Anonymous Use of E-Cigarettes and Vaping . 2019
514. Anonymous e-cigarettes, Vaping and Juuls: What You Need to Know . 2019
515. Ashton, J. Framing the question: electronic cigarettes and harm reduction . 2019
516. Baca-Atlas, M.; Mounsey, A.; Goldstein, A. O. Electronic Cigarettes: More Questions Than Answers . 2019
517. Balaji, S. M. Electronic cigarettes and its ban in India . 2019
518. Barkat, S. S.; Tellier, S. M.; Eloma, A. S. Varenicline for cessation from nicotine-containing electronic cigarettes . 2019
519. Masiero, M.; Lucchiari, C.; Mazzocco, K.; Veronesi, G.; Maisonneuve, P.; Jemos, C.; Sale, E. O.; Spina, S.; Bertolotti, R.; Pravettoni, G. Corrigendum: e-Cigarettes May Support Smokers With High Smoking-Related Risk Awareness to Stop Smoking in the Short Run: preliminary Results by Randomized Controlled Trial . 2020
520. Nct Metabolic Syndrome in Diabetic Smokers Using Cigarettes & Combustion-Free Nicotine Delivery Systems . 2020
521. Nct Salt-Based E-cigarette and IQOS Study . 2020
522. Nguyen Zarndt, A.; Donaldson, E. A.; Bernat, J. K.; Henrie, J. A.; Portnoy, D. B. Adult Use of and Transitions From Nicotine and Non-nicotine-Containing E-cigarettes: Data From the Population Assessment of Tobacco and Health (PATH) Study, 2013-2016 . 2020
523. Pierce, J. P.; Messer, K.; Leas, E. C.; Kealey, S.; White, M. M.; Benmarhnia, T. A Source of Bias in Studies of E-Cigarettes and Smoking Cessation . 2020
524. Polosa, R.; Farsalinos, K.; Prisco, D. A double-edged sword: e-cigarettes, and other electronic nicotine delivery systems (ENDS): reply . 2020
525. Sahu, K. K.; Mishra, A. K.; Lal, A.; Abraham, G. A double-edged sword: E-cigarettes, and other electronic nicotine delivery systems (ENDS) . 2020
526. Soule, E. K.; Lee, J. G. L.; Jenson, D. Major online retailers selling electronic cigarettes as smoking cessation products in the USA . 2020
527. Watkins, S. L.; Thrul, J.; Max, W.; Ling, P. M. Real-World Effectiveness of Smoking Cessation Strategies for Young and Older Adults: Findings From a Nationally Representative Cohort . 2020
528. Physicians for a Smoke-Free Canada Preliminary results of Canada's first Randomized Clinical Trial for e-cigarettes . 2020
529. Kousta, Stavroula E-cigarettes for smoking cessation . 2019
530. Zhang, Yuqing; Upson, Dona E-Cigarettes versus Nicotine-Replacement Therapy for Smoking Cessation. . 2019
531. Lee, Peter N; Fry, John S Investigating the effect of e-cigarette use on quitting smoking in adults aged 25 years or more using the PATH study . 2020
532. Gmel, Gerhard; Wicki, Matthias; Marmet, Simon; Studer, Joseph E-cigarette Use for Smoking Reduction and Cessation in a Four-year Follow-up Study Among Young Swiss Men: Some may Benefit, but they are Few. . 2020
533. 1.
534. Kasza KA, Edwards KC, Kimmel HL, Anesetti-Rothermel A, Cummings KM, Niaura RS, et al. Association of e-Cigarette Use With Discontinuation of Cigarette Smoking Among Adult Smokers Who Were Initially Never Planning to Quit. JAMA network open. 2021;4(12):e2140880.
535. Wang Y, Sung HY, Max WB. Changes in e-cigarette use and subsequent cigarette smoking cessation in the USA: evidence from a prospective PATH study, 2013-2018. Tobacco control [Internet]. 2022; Available from: <http://ovidsp.ovid.com/ovidweb.cgi?T=JS&PAGE=reference&D=medp&NEWS=N&AN=36601780>
536. Boozary LK, Frank-Pearce SG, Alexander AC, Waring JJC, Ehlke SJ, Businelle MS, et al. Correlates of e-cigarette use among adults initiating smoking cessation treatment. Drug and alcohol dependence. 2021; 224:108724.
537. Airagnes G, Lemogne C, Le Faou AL, Matta J, Romanello L, Wiernik E, et al. Do the associations between the use of electronic cigarettes and smoking reduction or cessation attempt persist after several years of use? Longitudinal analyses in smokers of the CONSTANCES cohort. Addictive behaviors. 2021; 117:106843.
538. Wang RJ, Bhadriraju S, Glantz SA. E-Cigarette Use and Adult Cigarette Smoking Cessation: A Meta-Analysis. American journal of public health. 2021;111(2):230–46.
539. Azagba S, Qeadan F, Shan L, Latham K, Wolfson M. E-Cigarette Use and Transition in Adult Smoking Frequency: A Longitudinal Study. American journal of preventive medicine. 2020;59(3):367–76.
540. Tattan-Birch H, Kock L, Brown J, Beard E, Bauld L, West R, et al. E-cigarettes With Varenicline Versus Varenicline for Smoking Cessation: A Pragmatic Randomised Controlled Trial. medRxiv [Internet]. 2022; Available from: [https://www.medrxiv.org/http://ovidsp.ovid.com/ovidweb.cgi?T=JS&PAGE=reference&D=empp&NEWS=N&AN=2018694436](https://www.medrxiv.org/http:/ovidsp.ovid.com/ovidweb.cgi?T=JS&PAGE=reference&D=empp&NEWS=N&AN=2018694436)
541. Chen R, Pierce JP, Leas EC, Benmarhnia T, Strong DR, White MM, et al. Effectiveness of e-cigarettes as aids for smoking cessation: evidence from the PATH Study cohort, 2017-2019. Tobacco control. 2023;32(e2):e145–52.
542. Kaplan B, Galiatsatos P, Breland A, Eissenberg T, Cohen JE. Effectiveness of ENDS, NRT and medication for smoking cessation among cigarette-only users: a longitudinal analysis of PATH Study wave 3 (2015-2016) and 4 (2016-2017), adult data. Tobacco control. 2023;32(3):302–7.
543. Villalobos RE, Ambrocio GPL, Fernandez L. Electronic cigarettes for smoking cessation: An individual patient meta-analysis of randomized controlled trials. European Respiratory Journal [Internet]. 2019;54(Supplement 63). Available from: [https://erj.ersjournals.com/content/54/suppl_63/OA5135http://ovidsp.ovid.com/ovidweb.cgi?T=JS&PAGE=reference&D=emed20&NEWS=N&AN=630917547](https://erj.ersjournals.com/content/54/suppl_63/OA5135http:/ovidsp.ovid.com/ovidweb.cgi?T=JS&PAGE=reference&D=emed20&NEWS=N&AN=630917547)
544. Adriaens K, Belmans E, Van Gucht D, Baeyens F. Electronic cigarettes in standard smoking cessation treatment by tobacco counselors in Flanders: E-cigarette users show similar if not higher quit rates as those using commonly recommended smoking cessation aids. Harm reduction journal. 2021;18(1):28.
545. Comiford AL, Rhoades DA, Spicer P, Dvorak JD, Ding K, Wagener TL, et al. Impact of e-cigarette use among a cohort of American Indian cigarette smokers: associations with cigarette smoking cessation and cigarette consumption. Tobacco control. 2021;30(1):103–7.
546. Pearson JL, Zhou Y, Smiley SL, Rubin LF, Harvey E, Koch B, et al. Intensive Longitudinal Study of the Relationship Between Cigalike E-cigarette Use and Cigarette Smoking Among Adult Cigarette Smokers Without Immediate Plans to Quit Smoking. Nicotine & tobacco research: official journal of the Society for Research on Nicotine and Tobacco. 2021;23(3):527–34.
547. Lee PN, Fry JS. Investigating the effect of e-cigarette use on quitting smoking in adults aged 25 years or more using the PATH study. F1000Research. 2020; 9:1099.
548. Sun T, Lim CCW, Rutherford BN, Johnson B, Leung J, Gartner C, et al. Is smoking reduction and cessation associated with increased e-cigarette use? Findings from a nationally representative sample of adult smokers in Australia. Addictive behaviors. 2022;127:107217.
549. Glasser AM, Vojjala M, Cantrell J, Levy DT, Giovenco DP, Abrams D, et al. Patterns of E-cigarette Use and Subsequent Cigarette Smoking Cessation Over 2 Years (2013/2014-2015/2016) in the Population Assessment of Tobacco and Health Study. Nicotine & tobacco research : official journal of the Society for Research on Nicotine and Tobacco. 2021;23(4):669–77.
550. Harlow AF, Stokes AC, Brooks DR, Benjamin EJ, Leventhal AM, McConnell RS, et al. Prospective association between e-cigarette use frequency patterns and cigarette smoking abstinence among adult cigarette smokers in the United States. Addiction (Abingdon, England). 2022;117(12):3129–39.
551. Han MA. Smoking cessation and mental health according to use of e-cigarettes and heated tobacco products by korean adults. International Journal of Mental Health and Addiction. 2021; No-Specified.
552. Chen R, Pierce JP, Leas EC, White MM, Kealey S, Strong DR, et al. Use of Electronic Cigarettes to Aid Long-Term Smoking Cessation in the United States: Prospective Evidence From the PATH Cohort Study. American journal of epidemiology. 2020;189(12):1529–37.
553. Rose JE, Behm FM, Willette PN, Botts TL, Botts DR. Using varenicline in combination with electronic nicotine delivery systems (ENDS). Drug and alcohol dependence. 2023; 251:110916.
554. Vanfrank B, Presley-Cantrell L. A Comprehensive Approach to Increase Adult Tobacco Cessation. JAMA - Journal of the American Medical Association. 2021;325(3):232–3.
555. Schlimpert V. Do e-cigarettes help in smoking cessation? MMW-Fortschritte der Medizin. 2020;162(7):14.
556. Knura M, Kurowski T, Lubanski J, Majek P, Jankowski M. E-cigarette: An effective tool to quit smoking or an additional source of nicotine?: PS211. Porto biomedical journal. 2017;2(5):188.
557. Masone MC. E-cigarettes and erectile dysfunction. Nature Reviews Urology. 2022;19(2):67.
558. Hammond S, Phillips J. E-Cigarettes and Vaping. Workplace health & safety. 2020;68(6):301.
559. Hopkinson NS. E-Cigarettes as a Smoking Cessation Aid - Toward Common Ground and a Rational Approach. American journal of respiratory and critical care medicine. 2023;208(10):1007–9.
560. Tonstad S. E-cigarettes for smokers trying to quit. The European respiratory journal [Internet]. 2020;56(4). Available from: <http://ovidsp.ovid.com/ovidweb.cgi?T=JS&PAGE=reference&D=med18&NEWS=N&AN=33122290>
561. Robinson J. E-cigarettes nearly twice as effective as nicotine-replacement therapy to stop smoking, study finds. Pharmaceutical Journal [Internet]. 2019;302(7922). Available from: [https://pharmaceutical-journal.com/article/news/e-cigarettes-nearly-twice-as-effective-as-nicotine-replacement-therapy-to-stop-smoking-study-findshttp://ovidsp.ovid.com/ovidweb.cgi?T=JS&PAGE=reference&D=emexb&NEWS=N&AN=2020348129](https://pharmaceutical-journal.com/article/news/e-cigarettes-nearly-twice-as-effective-as-nicotine-replacement-therapy-to-stop-smoking-study-findshttp:/ovidsp.ovid.com/ovidweb.cgi?T=JS&PAGE=reference&D=emexb&NEWS=N&AN=2020348129)
562. Jackson CD, Carter J, Kansagara D. E-Cigarettes Versus Nicotine Replacement Therapy for Smoking Cessation : Hajek P, Phillips-waller A, Przulj D, et al. A Randomized Trial of E-Cigarettes versus Nicotine-Replacement Therapy. N Engl J Med. 2019;380(7):629-637. Journal of general internal medicine. 2021;36(5):1481–3.
563. Shahab L, Goniewicz M. Electronic cigarettes are at least as effective as nicotine patches for smoking cessation. Evidence based medicine. 2014;19(4):133‐133.
564. Huhndorf J, Curtis T, Neher J, Safranek S. Electronic Cigarettes for Smoking Cessation. American family physician. 2021;103(1):53–4.
565. Katsaounou PA. Electronic cigarettes for smoking cessation: an opportunity to readdress smoking cessation treatment. The European respiratory journal [Internet]. 2020;56(4). Available from: <http://ovidsp.ovid.com/ovidweb.cgi?T=JS&PAGE=reference&D=med18&NEWS=N&AN=33122291>
566. He D, Niu W. More Explorations Needed on Association of Electronic Cigarette Use and Smoking Reduction. JAMA internal medicine. 2020;180(1):160.
567. Warner KE, Benowitz NL, McNeill A, Rigotti NA. Nicotine e-cigarettes as a tool for smoking cessation. Nature medicine. 2023;29(3):520–4.
568. Brown J, Shahab L. Smoking cessation support for dual users of cigarettes and electronic cigarettes. The Lancet Public health. 2021;6(7):e441–2.

### Systematic review (bibliography searched) (n=13)

1. Villanti, Andrea C., Feirman, Shari P., Niaura, Raymond S., Pearson, Jennifer L., Glasser, Allison M., Collins, Lauren K., and Abrams, David B. How do we determine the impact of e-cigarettes on cigarette smoking cessation or reduction? Review and recommendations for answering the research question with scientific rigor. *Addiction* 2018; 113(3):391-404.
2. Gentry, Sarah, Forouhi, Nita G., and Notley, Caitlin Are Electronic Cigarettes an Effective Aid to Smoking Cessation or Reduction Among Vulnerable Groups? A Systematic Review of Quantitative and Qualitative Evidence. *Nicotine & tobacco research* 2019; 21(5):602-616.
3. Hartmann-Boyce, Jamie, McRobbie, Hayden, Bullen, Chris, Begh, Rachna, Stead, Lindsay F., and Hajek, Peter Electronic cigarettes for smoking cessation. *The Cochrane database of systematic reviews* 2016; 9:CD010216-.
4. Lindson-Hawley, Nicola, Hartmann-Boyce, Jamie, Fanshawe, Thomas R., Begh, Rachna, Farley, Amanda, and Lancaster, Tim Interventions to reduce harm from continued tobacco use. *The Cochrane database of systematic reviews* 2016; 10:CD005231-.
5. El Dib, Regina, Suzumura, Erica A., Akl, Elie A., Gomaa, Huda, Agarwal, Arnav, Chang, Yaping, Prasad, Manya, Ashoorion, Vahid, Heels-Ansdell, Diane, Maziak, Wasim, and Guyatt, Gordon Electronic nicotine delivery systems and/or electronic non-nicotine delivery systems for tobacco smoking cessation or reduction: a systematic review and meta-analysis. *BMJ open* 2017; 7(2):e012680-.
6. Liu, Xing, Lu, Wan, Liao, Sheng, Deng, Zhongliang, Zhang, Zhongrong, Liu, Yun, and Lu, Weizhong Efficiency and adverse events of electronic cigarettes: A systematic review and meta-analysis (PRISMA-compliant article). *Medicine* 2018; 97(19):e0324-.
7. Orellana-Barrios, Menfil A., Payne, Drew, Medrano-Juarez, Rita M., Yang, Shengping, and Nugent, Kenneth Electronic Cigarettes for Smoking Cessation. *The American journal of the medical sciences* 2016; 352(4):420-426.
8. Vanderkam, Paul, Boussageon, Remy, Underner, Michel, Langbourg, Nicolas, Brabant, Yann, Binder, Philippe, Freche, Bernard, and Jaafari, Nematollah [Efficacy and security of electronic cigarette for tobacco harm reduction: Systematic review and meta-analysis]. *Presse medicale* 2016; 45(11):971-985.
9. Malas, Muhannad, van der Tempel, Jan, Schwartz, Robert, Minichiello, Alexa, Lightfoot, Clayton, Noormohamed, Aliya, Andrews, Jaklyn, Zawertailo, Laurie, and Ferrence, Roberta Electronic Cigarettes for Smoking Cessation: A Systematic Review. *Nicotine & tobacco research* 2016; 18(10):1926-1936.
10. Tomashefski, Amy The perceived effects of electronic cigarettes on health by adult users: A state of the science systematic literature review. *Journal of the American Association of Nurse Practitioners* 2016; 28(9):510-515.
11. Khoudigian, S., Devji, T., Lytvyn, L., Campbell, K., Hopkins, R., and O'Reilly, D. The efficacy and short-term effects of electronic cigarettes as a method for smoking cessation: a systematic review and a meta-analysis. *International journal of public health* 2016; 61(2):257-267.
12. Kalkhoran, Sara and Glantz, Stanton A. E-cigarettes and smoking cessation in real-world and clinical settings: a systematic review and meta-analysis. *The Lancet Respiratory medicine* 2016; 4(2):116-128.
13. Hartmann-Boyce, Jamie; McRobbie, Hayden; Lindson, Nicola; Bullen, Chris; Begh, Rachna; Theodoulou, Annika; Notley, Caitlin; Rigotti, Nancy A; Turner, Tari; Butler, Ailsa R; Hajek, Peter Electronic cigarettes for smoking cessation . 2020

### Protocol: has a relevant full-text publication, but has been captured in DB search (n=17)

1. Lopez, Alexa A., Cobb, Caroline O., Yingst, Jessica M., Veldheer, Susan, Hrabovsky, Shari, Yen, Miao Shan, Foulds, Jonathan, and Eissenberg, Thomas A transdisciplinary model to inform randomized clinical trial methods for electronic cigarette evaluation. *BMC public health* 2016; 16:217-.
2. Lucchiari, Claudio, Masiero, Marianna, Veronesi, Giulia, Maisonneuve, Patrick, Spina, Stefania, Jemos, Costantino, Omodeo Sale, Emanuela, and Pravettoni, Gabriella Benefits of E-Cigarettes Among Heavy Smokers Undergoing a Lung Cancer Screening Program: Randomized Controlled Trial Protocol. *JMIR research protocols* 2016; 5(1):e21-.
3. ISRCTN60477608 The efficacy of e-cigarettes compared with nicotine replacement therapy, when used within the UK stop smoking service. *Http://www.who.int/trialsearch/trial2.aspx? Trialid=isrctn60477608* 2015.
4. NCT01989923 Smoking Cessation in Women With Gynecological Conditions. *Https://clinicaltrials.gov/show/nct01989923* 2013.
5. NCT02417467 Evaluating the Efficacy of E-Cigarette Use for Smoking Cessation (E3) Trial. *Https://clinicaltrials.gov/show/nct02417467* 2015.
6. ISRCTN16931827 A trial to assess the benefit of offering an e-cigarette starter kit to smokers attempting to stop smoking with varenicline. *Http://www.who.int/trialsearch/trial2.aspx? Trialid=isrctn16931827* 2018.
7. NCT03358953 The Cardiovascular Impacts of Electronic Cigarettes in Comparison to the Use of Nicotine Replacement Patches. *Https://clinicaltrials.gov/show/nct03358953* 2017.
8. ACTRN12617001324303 Comparing electronic-cigarettes to traditional oral nicotine replacement therapy for smoking cessation among low-socioeconomic status smokers: a randomised controlled trial. *Http://www.who.int/trialsearch/trial2.aspx? Trialid=actrn12617001324303* 2017.
9. NCT02498145 Short Term Effects of Electronic Cigarettes in Tobacco Dependent Adults. *Https://clinicaltrials.gov/show/nct02498145* 2015.
10. NCT02328794 Randomized Clinical Trial to Reduce Harm From Tobacco. *Https://clinicaltrials.gov/show/nct02328794* 2014.
11. NCT02521662 The Use of Nicotine Patches Together With E-cigarettes (With and Without Nicotine) for Smoking Cessation. *Https://clinicaltrials.gov/show/nct02521662* 2015.
12. NCT02575885 Nicotine Delivery From Novel Non-Tobacco Electronic System in Smokers. *Https://clinicaltrials.gov/show/nct02575885* 2015.
13. ISRCTN17731903 Feasibility study of e-cigarettes in periodontitis. *Http://www.who.int/trialsearch/trial2.aspx? Trialid=isrctn17731903* 2016.
14. NCT03277495 Predictors and Consequences of Combustible Cigarette Smokers' Switch to Standardized Research E-Cigarettes. *Https://clinicaltrials.gov/show/nct03277495* 2017.
15. A randomised, parallel group, multi-centre study to evaluate the safety profile of the ITG EVP G1 product. *clinicaltrials.gov/show/NCT02029196* 2019.
16. Assessing the use of electronic cigarettes (e-cigarettes) as a harm reduction strategy. *clinicaltrials.gov/show/NCT02628964* 2019.
17. Walker, Natalie; Verbiest, Marjolein; Kurdziel, Tomasz; Laking, George; Laugesen, Murray; Parag, Varsha; Bullen, Chris Effectiveness and safety of nicotine patches combined with e-cigarettes (with and without nicotine) for smoking cessation: study protocol for a randomised controlled trial . 2019

### Protocol: does not have a full-text publication (n=87)

1. Berlin, Ivan, Dautzenberg, Bertrand, Lehmann, Blandine, Palmyre, Jessica, Liegey, Emmanuelle, De Rycke, Yann, and Tubach, Florence Randomised, placebo-controlled, double-blind, double-dummy, multicentre trial comparing electronic cigarettes with nicotine to varenicline and to electronic cigarettes without nicotine: the ECSMOKE trial protocol. *BMJ open* 2019; 9(5):e028832-.
2. Hersi, Mona, Traversy, Gregory, Thombs, Brett D., Beck, Andrew, Skidmore, Becky, et al. Effectiveness of stop smoking interventions among adults: protocol for an overview of systematic reviews and an updated systematic review. *Systematic reviews* 2019; 8(1):28-.
3. Walker, Natalie, Verbiest, Marjolein, Kurdziel, Tomasz, Laking, George, Laugesen, Murray, Parag, Varsha, and Bullen, Chris Effectiveness and safety of nicotine patches combined with e-cigarettes (with and without nicotine) for smoking cessation: study protocol for a randomised controlled trial. *BMJ open* 2019; 9(2):e023659-.
4. Bullen, Chris, Verbiest, Marjolein, Galea-Singer, Susanna, Kurdziel, Tomasz, Laking, George, Newcombe, David, Parag, Varsha, and Walker, Natalie. The effectiveness and safety of combining varenicline with nicotine e-cigarettes for smoking cessation in people with mental illnesses and addictions: study protocol for a randomised-controlled trial. *BMC public health* 2018; 18(1):596-.
5. Thomas, Kyla H., Caldwell, Deborah, Dalili, Michael N., Gunnell, David, Munafo, Marcus R., Stevenson, Matt, and Welton, Nicky J. How do smoking cessation medicines compare with respect to their neuropsychiatric safety? A protocol for a systematic review, network meta-analysis and cost-effectiveness analysis. *BMJ open* 2017; 7(6):e015414-.
6. Kinouani, Sherazade, Castera, Philippe, Laporte, Catherine, Petregne, Francois, and Gay, Bernard Factors and motivations associated with use of e-cigarette among primary care patients in a prospective cohort study: e-TAC study protocol. *BMJ open* 2016; 6(6):e011488-.
7. MacDonald, Marjorie, O'Leary, Renee, Stockwell, Tim, Reist, Dan, and Clearing the Air project team Clearing the air: protocol for a systematic meta-narrative review on the harms and benefits of e-cigarettes and vapour devices. *Systematic reviews* 2016; 5:85-.
8. Bell, Stephanie, Dean, Judith, Gilks, Charles, Boyd, Mark A., Fitzgerald, Lisa, Mutch, Allyson, Baker, Peter, Neilsen, Graham, and Gartner, Coral E. Tobacco Harm Reduction with Vaporised Nicotine (THRiVe): The Study Protocol of an Uncontrolled Feasibility Study of Novel Nicotine Replacement Products among People Living with HIV Who Smoke. *International journal of environmental research and public health* 2017; 14(7)-.
9. Klonizakis, Markos, Crank, Helen, Gumber, Anil, and Brose, Leonie S. Smokers making a quit attempt using e-cigarettes with or without nicotine or prescription nicotine replacement therapy: Impact on cardiovascular function (ISME-NRT) - a study protocol. *BMC public health* 2017; 17(1):293-.
10. Fraser, D., Borland, R., and Gartner, C. Protocol for a randomised pragmatic policy trial of nicotine products for quitting or long-term substitution in smokers. *BMC public health* 2015; 15:1026-.
11. NCT03589989 The ESTxENDS Trial- Electronic Nicotine Delivery Systems (ENDS/Vaporizer/E-cigarette) as an Aid for Smoking Cessation. *Https://clinicaltrials.gov/show/nct03589989* 2018.
12. NCT03603340 The ESTxENDS Trial- Effects of Using Electronic Nicotine Delivery Systems (ENDS/Vaporizer/E-cig) on Depression. *Https://clinicaltrials.gov/show/nct03603340* 2018-.
13. NCT03603353 The ESTxENDS Trial- Effects of Using Electronic Nicotine Delivery Systems (ENDS/Vaporizer/E-cig) on Sleep Quality. *Https://clinicaltrials.gov/show/nct03603353* 2018.
14. NCT02004171 Electronic Cigarettes or Nicotine Inhaler for Smoking Cessation. *Https://clinicaltrials.gov/show/nct02004171* 2013.
15. NCT03632421 The ESTxENDS Trial-effects of Using Electronic Nicotine Delivery Systems (ENDS/Vaporizer/E-cig) on Respiratory Symptoms. *Https://clinicaltrials.gov/show/nct03632421* 2018.
16. NCT03612336 The ESTxENDS Trial- Metabolic Effects of Using Electronic Nicotine Delivery Systems (ENDS/Vaporizer/E-cig). *Https://clinicaltrials.gov/show/nct03612336* 2018.
17. NCT02398487 Personal Vaporizer vs Cigalike. *Https://clinicaltrials.gov/show/nct02398487* 2014.
18. Caponnetto, P., Polosa, R., Auditore, R., Minutolo, G., Signorelli, M., Maglia, M., Alamo, A., Palermo, F., and Aguglia, E. Smoking cessation and reduction in schizophrenia (SCARIS) with e-cigarette: study protocol for a randomized control trial. *Trials* 2014; 15:88-.
19. NCT02487953 Electronic Nicotine Delivery Systems as a Smoking Cessation Treatment. *Https://clinicaltrials.gov/show/nct02487953* 2015.
20. NCT03612453 ESTxENDS Trial- Oxidative Stress Induced by Electronic Nicotine Delivery Systems (ENDS/Vaporizer/E-cig) Measured in EBC. *Https://clinicaltrials.gov/show/nct03612453* 2018.
21. NCT03612544 The ESTxENDS Trial- Toxins From Using Electronic Nicotine Delivery Systems (ENDS/Vaporizer/E-cig). *Https://clinicaltrials.gov/show/nct03612544* 2018.
22. NCT03612375 ESTxENDS Trial-Oxidative Stress Induced by Electronic Nicotine Delivery Systems (ENDS/Vaporizer/E-cig) Measured in Urine. *Https://clinicaltrials.gov/show/nct03612375* 2018.
23. NCT01925781 e-Cigarettes Versus NRT Gum for Smoking Cessation. *Https://clinicaltrials.gov/show/nct01925781* 2013.
24. NCT03938298 The ESTxENDS Trial: pulmonary Function Substudy. *Https://clinicaltrials.gov/show/nct03938298* 2019.
25. ACTRN12610000866000 ASCEND: a Study of Cessation using Electronic Nicotine Devices. *Http://www.who.int/trialsearch/trial2.aspx? Trialid=actrn12610000866000* 2010.
26. NCT02124187 Smoking Cessation And Reduction in Depression. *Https://clinicaltrials.gov/show/nct02124187* 2014.
27. NCT03575468 Enhanced E-cigarette Coaching Intervention for Dual Users of Cigarettes and E-cigarettes. *Https://clinicaltrials.gov/show/nct03575468* 2018.
28. NCT03635333 Effects of E-Cigarette Flavors on Adults. *Https://clinicaltrials.gov/show/nct03635333* 2018.
29. NCT03185546 REN-Project 2 Cigarette and E-cigarette Nicotine Content and E-liquid Flavors. *Https://clinicaltrials.gov/show/nct03185546* 2017.
30. NCT01842828 UK-Czech E-cigarette Study. *Https://clinicaltrials.gov/show/nct01842828* 2013.
31. ISRCTN13288677 Can electronic cigarettes and nicotine replacement treatment help reduce smoking in smokers who struggle to quit? *Http://www.who.int/trialsearch/trial2.aspx? Trialid=isrctn13288677* 2017.
32. ISRCTN59404712 GP/nurse promotion of e-cigarettes in supporting reduced smoking and cessation in smokers. *Http://www.who.int/trialsearch/trial2.aspx? Trialid=isrctn59404712* 2017.
33. ISRCTN62025374 Helping pregnant smokers quit: a multi-centre study of electronic cigarettes and nicotine patches. *Http://www.who.int/trialsearch/trial2.aspx? Trialid=isrctn62025374* 2017.
34. NCT03630614 Randomized Trial of Electronic Cigarettes With or Without Nicotine in Smoking Cessation. *Https://clinicaltrials.gov/show/nct03630614* 2018.
35. ACTRN12616001355460 Varenicline alone or with nicotine e-cigarettes for smoking cessation in people with mental illness and drug and/or alcohol dependence. *Http://www.who.int/trialsearch/trial2.aspx? Trialid=actrn12616001355460* 2016.
36. NCT03691350 Bronchoscopy in Determining the Effect of E-Cigarette Smoking on Biomarkers in the Lungs. *Https://clinicaltrials.gov/show/nct03691350* 2018.
37. NCT03050853 The Appeal and Impact of E-cigarettes in Smokers With SMI. *Https://clinicaltrials.gov/show/nct03050853* 2017.
38. NCT03569748 Heated Tobacco Products vs Electronic Cigarettes. *Https://clinicaltrials.gov/show/nct03569748* 2018.
39. NCT03249428 E-Cigarette Inner City RCT. *Https://clinicaltrials.gov/show/nct03249428* 2017.
40. NCT03962660 Harm Reduction for Tobacco Smoking With Support of Tobacco-Replacing Electronic Nicotine Delivery Systems. *Https://clinicaltrials.gov/show/nct03962660* 2019.
41. NCT02422914 Benefits of Tobacco Free Cigarette. *Https://clinicaltrials.gov/show/nct02422914* 2015.
42. NCT01733706 Early Smoking Reduction or Cessation by Means of no Nicotine Electronic Cigarette Added to Standard Counselling. *Https://clinicaltrials.gov/show/nct01733706* 2012.
43. NCT03593239 Comparing the Pharmacokinetics of Nicotine Salt Based ENDS in Healthy Smokers. *Https://clinicaltrials.gov/show/nct03593239* 2018.
44. A trial of e-cigarettes: natural uptake, patterns and impact of use. *clinicaltrials.gov/show/NCT02357173* 2019.
45. E-cigarettes: dynamic patterns of use and health effects. *clinicaltrials.gov/show/NCT02527980* 2019.
46. The role of nicotine and non-nicotine alkaloids in e-cigarette use and dependence. *clinicaltrials.gov/show/NCT02590393* 2019.
47. Changes in lung function parameters, bronchial reactivity, state of health and smoking behaviour associated with changing from conventional smoking to electronic cigarettes. *clinicaltrials.gov/show/NCT02635620* 2019.
48. Evaluation of appeal and impact of e-cigarettes among chronic smokers with smoking-related cancers. *clinicaltrials.gov/show/NCT02648178* 2019.
49. Besaratinia et al., Are Smokers Switching to Vaping at Lower Risk for Cancer? NCT03750825.
50. Cox et al., Exploring the uptake and use of electronic cigarettes provided to smokers accessing homeless centres: a feasibility study, ISRCTN14140672.
51. Yingst et al., Does Switching to Nicotine Containing Electronic Cigarettes Reduce Health Risk Markers, NCT03625986.
52. Kalkhoran et al., Effects of Electronic Cigarettes on Smokers With Mild to Moderate Chronic Obstructive Pulmonary Disease, NCT03379025.
53. Burri et al., e-Cigarette - DNA Adducts, NCT03284632.
54. Joseph et al., Changes in Biomarkers Associated With Use of Electronic Cigarettes, NCT03084315.
55. Crystal et al., Biology of the Oral Epithelium of E-Cigarette Smokers, NCT03028558.
56. Cinciripini et al., Concomitant Use of Very Low Nicotine Content Cigarettes and e-Cigarettes, NCT02964182.
57. Yoon et al., E-cigarettes to Promote Smoking Reduction Among Individuals With Schizophrenia, NCT02918630.
58. Sellares et al., Influence of Electronic Cigarettes in the Evaluation of the Inflammatory Response in Patients With a Diagnosis of COPD, NCT02892396.
59. Hopkinson et al., Vascular EffectS of regUlar Cigarettes Versus electronIc Cigarette Use, ISRCTN59133298.
60. Begh, R.; Coleman, T.; Yardley, L.; Barnes, R.; Naughton, F.; Gilbert, H.; Ferrey, A.; Madigan, C.; Williams, N.; Hamilton, L.; Warren, Y.; Grabey, J.; Clark, M.; Dickinson, A.; Aveyard, P. Examining the effectiveness of general practitioner and nurse promotion of electronic cigarettes versus standard care for smoking reduction and abstinence in hardcore smokers with smoking-related chronic disease: protocol for a randomised controlled trial . 2019
61. Caponnetto, P.; Maglia, M.; Polosa, R. Efficacy of smoking cessation with varenicline plus counselling for e-cigarettes users (VAREVAPE): A protocol for a randomized controlled trial. 2019
62. Nct Electronic Cigarettes as a Harm Reduction Strategy in Individuals With Substance Use Disorder. 2019
63. Nct Low Nicotine Cigarettes Plus Electronic Cigarettes. 2019
64. Nct Low Nicotine Content Cigarettes in Vulnerable Populations: opioid Use Disorder.2019
65. Nct Impact of Alternative Nicotine-Delivery Products on Combustible Cigarette Use. 2019
66. Caponnetto, P.; Caruso, M.; Maglia, M.; Emma, R.; Saitta, D.; Busa, B.; Polosa, R.; Prosperini, U.; Pennisi, A.; Benfatto, F.; et al. Non-inferiority trial comparing cigarette consumption, adoption rates, acceptability, tolerability, and tobacco harm reduction potential in smokers switching to Heated Tobacco Products or electronic cigarettes: study protocol for a randomized controlled trial. 2020
67. Hebert-Losier, A.; Filion, K. B.; Windle, S. B.; Eisenberg, M. J. A Randomized Controlled Trial Evaluating the Efficacy of E-Cigarette Use for Smoking Cessation in the General Population: E3 Trial Design. 2020
68. Nct Efficacy and Safety of an Electronic Nicotine Delivery Device (E-Cigarette). 2010
69. Nct Antismoking Effects of Electronic Cigarettes in Subjects With Schizophrenia and Their Potential Influence on Cognitive Functioning. 2013
70. Nct E-cigarettes and Cardiovascular Function. 2017
71. Nct The ESTxENDS Trial- Electronic Nicotine Delivery Systems as an Aid for Smoking Cessation-extension of Follow-up . 2020
72. Nct Electronic Cigarettes as a Harm Reduction Strategy Among Patients With COPD . 2020
73. Nct Electronic Cigarettes as a Harm Reduction Strategy Among People Living With HIV/AIDS . 2020
74. Pope, Ian Cessation of Smoking Trial in the Emergency Department (CoSTED).
75. Myers Smith, Katie Do e-cigarettes help smokers quit when not accompanied by intensive behavioural support?
76. Nct. A Randomized Study to Evaluate Harm Reduction Products as a Second Line Intervention for Adult Smokers Who Do Not Quit With Nicotine Replacement Therapy (NRT). https://clinicaltrials.gov/ct2/show/NCT06088862 [Internet]. 2023; Available from: <https://www.cochranelibrary.com/central/doi/10.1002/central/CN-02603824/full>
77. Nct. Cytisine and E-cigarettes With Supportive Text-messaging for Smoking Cessation (Cess@Tion). https://clinicaltrials.gov/show/NCT05311085 [Internet]. 2022; Available from: <https://www.cochranelibrary.com/central/doi/10.1002/central/CN-02388079/full>
78. Isrctn. E-cigarette support for smoking cessation: identifying the effectiveness of intervention components in an online randomised optimisation experiment. https://trialsearch.who.int/Trial2.aspx?TrialID=ISRCTN54776958 [Internet]. 2022; Available from: <https://www.cochranelibrary.com/central/doi/10.1002/central/CN-02486522/full>
79. Isrctn. E-cigarettes vs usual care for smoking cessation when offered at homeless centres. https://trialsearch.who.int/Trial2.aspx?TrialID=ISRCTN18566874 [Internet]. 2021; Available from: <https://www.cochranelibrary.com/central/doi/10.1002/central/CN-02351753/full>
80. Elling JM, Crutzen R, Talhout R, de Vries H. Effects of Providing Tailored Information About e-Cigarettes in a Web-Based Smoking Cessation Intervention: Protocol for a Randomized Controlled Trial. JMIR research protocols. 2021;10(5):e27088.
81. Nct. Electronic Cigarettes and Nicotine Pouches for Smoking Cessation. https://clinicaltrials.gov/show/NCT05715164 [Internet]. 2023; Available from: <https://www.cochranelibrary.com/central/doi/10.1002/central/CN-02522726/full>
82. Cox S, Bauld L, Brown R, Carlise M, Ford A, Hajek P, et al. Evaluating the effectiveness of e-cigarettes compared with usual care for smoking cessation when offered to smokers at homeless centres: protocol for a multi-centre cluster randomised controlled trial in Great Britain. Addiction (Abingdon, England) [Internet]. 2022; Available from: <https://www.cochranelibrary.com/central/doi/10.1002/central/CN-02372783/full>
83. Cox S, Bauld L, Brown R, Carlisle M, Ford A, Hajek P, et al. Evaluating the effectiveness of e-cigarettes compared with usual care for smoking cessation when offered to smokers at homeless centres: protocol for a multi-centre cluster-randomized controlled trial in Great Britain. Addiction (Abingdon, England). 2022;117(7):2096–107.
84. Nct. Quit or Switch: e-cigarette Study. https://clinicaltrials.gov/show/NCT05525078 [Internet]. 2022; Available from: <https://www.cochranelibrary.com/central/doi/10.1002/central/CN-02457112/full>
85. Holliday R, Preshaw P, McColl E, Ryan V, Cherlin S, Wilson N, et al. Research protocol for the ENHANCE-D trial: Enhancing dental health advice. Journal of Clinical Periodontology. 2022;49(Supplement 23):79.
86. El-Khoury F, El Aarbaoui T, Heron M, Hejblum G, Metadieu B, Le Faou AL, et al. Smoking cessation using preference-based tools among socially disadvantaged smokers: study protocol for a pragmatic, multicentre randomised controlled trial. BMJ open. 2021;11(6):e048859.
87. Nct. Using ANDS to Reduce Harm for Low SES Cigarette Smokers. https://clinicaltrials.gov/show/NCT05327439 [Internet]. 2022; Available from: <https://www.cochranelibrary.com/central/doi/10.1002/central/CN-02388329/full>

### Abstract: has a relevant full-text publication, but captured in DB search (n=8)

1. Vardavas, C., Filippidis, F., Laverty, A., Mons, U., and Jimenez-Ruiz, C. Smoking cessation in europe: Trends in methods used in the european union between 2012 and 2014. *European Respiratory Journal* 2017; 50(Supplement 61).
2. Lee, S. M., Tenney, R., Wallace, A., and Arjojmandi, M. The end perioperative smoking pilot study: A randomized trial comparing e-cigarettes versus nicotine patch. *Canadian Journal of Anesthesia* 2017; 64(1 Supplement 1):S48-S49.
3. Donny, E. Reducing harm by targeting the addictiveness of combusted tobacco products through regulated reductions in nicotine content. *Cancer Research* 2017; 77(13 Supplement 1).
4. Pravettoni, G., Masiero, M., Lucchiari, C., Maisenneuve, P., Mazzocco, K., and Veronesi, G. The role of electronic cigarettes in smoking cessation among heavy smokers undergoing a lung cancer screening program: Preliminary results of a randomized controlled study. *Psycho-oncology* 2016; 25(SUPPL. 2):72-.
5. Demichelis, S., Rapetti, S. G., Galetta, D., Bruno, A., Bria, E., Pilotto, S., Valmadre, G., Catino, A., Gianetta, M., Vallone, S., Pacchiana, M. V., and Novello, S. Italian multicentric survey on smoking cessation in lung disease patients and the role of healthcare workers in this contest. *Journal of Thoracic Oncology* 2015; 10(9 SUPPL. 2):S486-S487.
6. Durmowicz, E. L. Other tobacco products electronic devices/water pipes/hookas. *Journal of Thoracic Oncology* 2015; 10(9 SUPPL. 2):S131-.
7. Lopez, A. A., Hiler, M., Ratner, T., Pettaway, K., Breland, A., and Eissenberg, T. ECIG-induced suppression of nicotine/tobacco abstinence symptoms. *Drug and alcohol dependence* 2015; 156:e133-.
8. Gubner, N. R., Le, T., Tajima, B., Andrews, B., Passalacqua, E., and Guydish, J. Use of electronic cigarettes among smokers in addiction treatment. *Drug and alcohol dependence* 2015; 156:e85-.

### Abstract: no full-text publication (n=47)

1. Prakash, S., Wissmann, R., Augustson, E., and Vose, J. G. PMH26 IDENTIFYING PREDICTIVE ATTRIBUTES OF ADULT SMOKERS WHO CEASE COMBUSTIBLE SMOKING USING THE JUUL ELECTRONIC NICOTINE DELIVERY SYSTEM (ENDS) VIA LOGISTIC REGRESSION AND CART. *Value in Health* 2019; 22(Supplement 2):S230-.
2. Ford, A., Sinclair, L., McKell, J., Harrow, S., Macphee, J., Morrison, A., and Bauld, L. Feasibility and acceptability of e-cigarettes as an aid to quitting smoking among lung cancer patients: A pilot study. *Tobacco induced diseases* 2018; 16(Supplement 1):195-196.
3. Mohamed, M. H. N., Rahman, A., and Jamshed, S. Long-term effectiveness, safety, perception, satisfaction and cost of electronic cigarette among single and dual users in Malaysia. *Tobacco induced diseases* 2018; 16(Supplement 1):120-.
4. Loukas, A., Marti, C. N., Creamer, M. R., and Perry, C. L. Does use of electronic nicotine delivery systems predict current cigarette use among young adults? *Tobacco induced diseases* 2018; 16(Supplement 1):88-.
5. Perez, C., Cavalcante, T., Mendes, F., Szklo, A., Fong, G. T., Craig, L., Yan, M., and Li, G. Reasons for e-cigarette use and perceptions of harm in Brazil: Findings from the ITC Brazil Wave 2 (2012-13) and 3 (2016-17) surveys. *Tobacco induced diseases* 2018; 16(Supplement 1):86-.
6. Brandon, T., Martinez, U., Simmons, V., Meltzer, L., Sutton, S., Drobes, D., Unrod, M., and Harrell, P. Dual use of combustible and electronic cigarettes: Patterns and associations between products. *Tobacco induced diseases* 2018; 16(Supplement 1):90-.
7. Ioakeimidis, N., Vlachopoulos, C., Georgakopoulos, C., Abdelrasoul, M., Skliros, N., Katsi, V., Vaina, S., and Tousoulis, D. Smoking cessation rates with varenicline and electronic cigarettes in relapsed smokers with a history of acute coronary syndrome. *European Heart Journal* 2018; 39(Supplement 1):242-.
8. Bozier, J., Xenaki, D., Adcock, I., and Oliver, B. Lung cells from people with COPD are hyperresponsive to E-cigarette vapour. *European Respiratory Journal* 2018; 52(Supplement 62).
9. Bailey, A., Durrant, P., Goldsmith, N., Zhuikova, E., Liu, L., Ioannides, A., Ioannides, C., Kovatsi, L., and Marczylo, T. Monitoring the neurobehavioral and toxicological effects of the transition from smoking to e-cigarette use. *Brain and Neuroscience Advances* 2017; 1:54-.
10. Crippa, G., Balordi, V., Bravi, E., and Fontana, M. Electronic cigarette acutely increases blood pressure in hypertensive patients. Evaluation by non-invasive continuous (beat-to-beat) blood pressure monitoring. *Journal of Hypertension* 2018; 36(Supplement 3):e168-.
11. Albano, C., Yang, F., Buckley, B., and Williams, L. A systematic review on the health and safety of electronic cigarettes. *Journal of Managed Care and Specialty Pharmacy* 2016; 22(4-A SUPPL.):S88-S89.
12. Ulmer, A. Addressing smoking three times a year. *Heroin Addiction and Related Clinical Problems* 2018; 20(Supplement 2):74-.
13. Peters, M. E-Cigarettes - What Do Lung Cancer Advocates Need to Know? *Journal of Thoracic Oncology* 2018; 13(10 Supplement):S257-.
14. Cummings, K. M. PRO- Electronic Cigarettes: A Cessation Tool. *Journal of Thoracic Oncology* 2018; 13(10 Supplement):S270-S271.
15. Mayel, M. Are vapes an effective device for smoking cessation or a gateway to conventional tobacco smoking? *Canadian Journal of Respiratory Therapy* 2018; 54(2):55-.
16. Miaw, J. L., Hock, Y. L. K., Yusoff, M. F. M., Ab, Rahman J., Ab Rahman, N. S., Draman, S. B., et al. Low nicotine addiction level, older age, and being female are associated with contemplation and preparation stages of smoking cessation among adult current smokers in Malaysia. *Medical Journal of Malaysia* 2017; 72(Supplement 1):69-.
17. Crippa, G., Bergonzi, M., Bravi, E., Balordi, V., and Cassi, A. Effect of electronic cigarette smoking on blood pressure in hypertensive patients. Evaluation by non-invasive continuous ambulatory blood pressure measurement. *Journal of Hypertension* 2018; 36(Supplement 1):e4-.
18. Sribna, O. and Kvasha, O. Electronic nicotine delivery systems (ENDS) usage and cardiovascular diseases risk. *European Journal of Preventive Cardiology* 2018; 25(2 Supplement 1):S84-.
19. Davoren, K., Rashid, M., Nafeh, F., Moy, N., Elashoff, R., Lindner, J., and Victor, R. G. Electronic cigarettes impair human coronary endothelial function. *FASEB Journal* 2018; 32(1 Supplement 1).
20. Mendelsohn, C., Castle, D., McRobbie, H., and Bullen, C. How to help your patients quit smoking or reduce harm from Tobacco. *Australian and New Zealand Journal of Psychiatry* 2018; 52(1 Supplement 1):12-.
21. Ashford, K. B., Chavan, N. R., Wiggins, A., Barnett, J., McCubbin, A., Ducas, L., and O'Brien, J. M. Prenatal electronic cigarette, dual use and nicotine dependency. *American Journal of Obstetrics and Gynecology* 2018; 218(1 Supplement 1):S431-S432.
22. Steliga, M. Nicotine dependence and cessation in lung cancer patients. *Journal of Thoracic Oncology* 2017; 12(11 Supplement 2):S1691-.
23. Goniewicz, M. Role of electronic cigarettes in lung cancer prevention among smokers. *Journal of Thoracic Oncology* 2017; 12(11 Supplement 2):S1878-.
24. Kerr, D., Touyz, R., and Delles, C. The immediate effects of electronic cigarette use and tobacco smoking on vascular and respiratory function in healthy volunteers: A crossover study. *Journal of Human Hypertension* 2017; 31(10):662-.
25. Kim, S. J. and Marsch, L. A. Fear-eliciting antismoking ads unintentionally motivate smokers to consider an e-cigarette: The boomerang effect. *Drug and alcohol dependence* 2017; 171:e103-.
26. Liang, J., Abramson, M. J., Zwar, N., Russell, G., Holland, A. E., Bonevski, B., Mahal, A., Van, Hecke B., Phillips, K., Eustace, P., Petrie, K., Wilson, S., and George, J. Quitting experiences and smoking cessation preferences of smokers in australian general practices. *Respirology (Carlton, Vic.)* 2017; 22(Supplement 2):34-.
27. Paredes, Aller S., Aguiar, C., Guerrero, A. C., Holt, G., Campos, M., Salathe, M., and Schmid, A. E-cigarette vaping impairs electrolyte transport over the apical nasal mucosa. *American journal of respiratory and critical care medicine* 2017; 195.
28. Guerrero, A. M., Schweitzer, M. D., Balestrini, K., Luna Diaz, L. V., Holt, G., Mirsaeidi, M., Salathe, M., and Campos, M. Vaping characteristics of veterans replacing tobacco smoking with electronic cigarettes. *American journal of respiratory and critical care medicine* 2017; 195.
29. Iaccarino, J. M., Duran, C., Wiener, R. S., and Kathuria, H. Smoking cessation interventions in the setting of low dose computed tomography (LDCT) lung cancer screening: A systematic review. *American journal of respiratory and critical care medicine* 2017; 195.
30. Hirschi, K. M., Lewis, J. B., Ostergar, A. S., Hall, P. D., Broberg, D. S., Arroyo, J. A., and Reynolds, P. R. Involvement of RAGE signaling and inflammatory cytokine elaboration following in vitro exposure to electronic cigarette liquid. *FASEB Journal* 2017; 31(1 Supplement 1).
31. Anand, V. Unique issues with e-cigarettes, smokeless tobacco, and hookah. *Journal of the American Academy of Child and Adolescent Psychiatry* 2016; 55(10 Supplement 1):S21-.
32. De La Garza, R., Yoon, J., Yammine, L., Holst, M., and Salas, R. Preliminary evaluation of the effects of electronic cigarettes versus own cigarette on withdrawal, craving, and smoking severity in tobacco-dependent volunteers. *Neuropsychopharmacology* 2016; 41(Supplement 1):S380-.
33. Salas, R., Stewart, C., De La Garza, R., and Curtis, K. Resting state functional connectivity in tobacco smokers and electronic cigarette users: Correlations with gut bacterial diversity. *Neuropsychopharmacology* 2016; 41(Supplement 1):S427-.
34. Feemster, L. E-cigarettes update. *Respirology* 2016; 21(Supplement 3):7-.
35. Humair, J.-P. and Tango, R. Electronic cigarette use for smoking reduction and cessation in primary care. *Journal of general internal medicine* 2016; 31(2 SUPPL. 1):S877-.
36. Campagna, D., Morjaria, J. B., Caponnetto, P., Caruso, M., Amaradio, M. D., Ciampi, G., Russo, C., and Polosa, R. Persisting long term benefits of smoking abstinence and reduction in asthmatic smokers who have switched to electronic cigarettes. *Journal of Allergy and Clinical Immunology* 2016; 137(2 SUPPL. 1):AB5-.
37. Foley, N. C. and Lindsay, P. The use of electronic cigarettes is not associated with cessation of smoking: A systematic review and meta-analysis. *International Journal of Stroke* 2015; 10(SUPPL. 4):64-65.
38. Cummings, K. M. Electronic nicotine delivery devices (ENDS): Ecigarettes. *Journal of Thoracic Oncology* 2015; 10(9 SUPPL. 2):S151-.
39. Harvanko, A., Martin, C. A., Fogel, J. S., Lile, J., and Kelly, T. H. A comparison of the behavioral effects of electronic and tobacco cigarettes following 24-h tobacco deprivation. *Drug and alcohol dependence* 2015; 156:e92-.
40. Eisenhofer, J., Makanjuola, T., Martinez, V., Thompson-Lake, D. G., Rodgman, C., DeBrule, D. S., Graham, D. P., and De La Garza, I. I. R. Efficacy of electronic cigarettes for smoking cessation in veterans. *Drug and alcohol dependence* 2015; 156:e63-e64.
41. Humair, J-P and Tango, R Can e-cigarette help patients to reduce or stop smoking in primary care practice. *Journal of General Internal Medicine* 2014; 29(S480).
42. The Ontario Tobacco Research Unit E-liquids Products Labels: The Good, the bad, and the Ugly.
43. Chaumont, M.; El Channan, M.; Bernard, A.; Lesage, A.; Deprez, G.; Van Muylem, A.; Schaefer, T.; Faoro, V.; Van De Borne, P. Short-term high wattage e-cigarette cessation improves cardiorespiratory outcomes in regular users: A randomized crossover trial. 2019
44. Ioakeimidis, N.; Vlachopoulos, C.; Georgakopoulos, C.; Dima, I.; Solomou, E.; Gardikioti, V.; Oikonomou, E.; Tousoulis, D. Two-year therapeutic effectiveness of pharmacotherapy versus electronic cigarettes for smoking cessation: A single-center experience. 2019
45. Zhuikova, E.; Durrant, P.; Macauley, E.; Goss, H.; Goldsmith, N.; Ioannides, C.; Marczylo, T.; Bailey, A. Monitoring the transition from cigarette smoking to electronic cigarette use: Nicotine intake, psychometric, and clinical outcomes. 2019
46. Skelton E, Robinson M, Lum A, Dunlop A, Baker A, Gartner C, et al. A pilot study of abrupt verse gradual smoking cessation in combination with electronic nicotine devices for smokers receiving alcohol and other drug treatment. Asia-Pacific Journal of Clinical Oncology. 2020;16(SUPPL 6):37–8.
47. Ikonomidis I, Katogiannis K, Kostelli G, Kourea K, Kyriakou E, Kypraiou A, et al. Effects of electronic cigarette on platelet and vascular function after one month of use. European Heart Journal. 2020;41(SUPPL 2):2359.

### Abstract: with a full-text publication, but is not relevant (n=3)

1. Van Der Eijk, Y., Petersen, A. B., and Bialous, S. E-cigarette use in pregnancy: A human rights-based approach to policy and practice. *Annals of Global Health* 2017; 83(1):204-205.
2. Barna, S., Garai, I., Rozsa, D., Varga, J., Fodor, A., Szilasi, M., and Galuska, L. Comparison of cigarette and e-cigarette smoking to alveolocapillary membrane by dynamic ventilation scintigraphy. *Nuclear Medicine Review* 2017; 20(2):117.
3. Fuller, T., Acharya, A., Bhaskar, G., Yu, M., Little, S., and Tarin, T. Evaluation of E-cigarettes users urine for known bladder carcinogens. *Journal of Urology* 2017; 197(4 Supplement 1):e1179.

## Population not of interest (n=13)

1. Bowler, Russell P., Hansel, Nadia N., Jacobson, Sean, Graham Barr, R., Make, Barry J., et al. Electronic Cigarette Use in US Adults at Risk for or with COPD: Analysis from Two Observational Cohorts. *Journal of general internal medicine* 2017; 32(12):1315-1322.
2. Soneji, Samir, Barrington-Trimis, Jessica L., Wills, Thomas A., Leventhal, Adam M., Unger, et al. Association Between Initial Use of e-Cigarettes and Subsequent Cigarette Smoking Among Adolescents and Young Adults: A Systematic Review and Meta-analysis. *JAMA pediatrics* 2017; 171(8):788-797.
3. Bar-Zeev, Y., Bovill, M., Bonevski, B., Gruppetta, M., Oldmeadow, C., Palazzi, K., Atkins, L., Reath, J., and Gould, G. S. Improving smoking cessation care in pregnancy at Aboriginal Medical Services: ICAN QUIT in Pregnancy' step-wedge cluster randomised study. *BMJ open* 2019; 9(6):e025293-.
4. Riley, H. E. M., Berry-Bibee, E., England, L. J., Jamieson, D. J., Marchbanks, P. A., and Curtis, K. M. Hormonal contraception among electronic cigarette users and cardiovascular risk: A systematic review. *Contraception* 2016; 93(3):190-208.
5. Cox, S., Kosmider, L., McRobbie, H., Goniewicz, M., Kimber, C., Doig, M., and Dawkins, L. E-cigarette puffing patterns associated with high and low nicotine e-liquid strength: effects on toxicant and carcinogen exposure. *BMC public health* 2016; 16:999-.
6. Ramoa, C. P., Hiler, M. M., Spindle, T. R., Lopez, A. A., Karaoghlanian, N., Lipato, T., Breland, A. B., Shihadeh, A., and Eissenberg, T. Electronic cigarette nicotine delivery can exceed that of combustible cigarettes: a preliminary report. *Tobacco control* 2016; 25(e1):e6-e9.
7. Wen X, Chung MS, Liszewski K, Todoro L, Giancarlo EVE, Zhang W, et al. Cigarette smoking abstinence among pregnant women using e-cigarettes or nicotine replacement therapy. Chest. 2023;164(4 Supplement): A6373–4.
8. Doherty LK. Electronic cigarette use among pregnant cigarette smokers in the Quit4Baby trial: Predictive factors of use, the efficacy of e-cigarettes for smoking cessation, and associations between combined cigarette and e-cigarette use and the risk of adverse neonat. Dissertation Abstracts International: Section B: The Sciences and Engineering. 2022;83(10-B):No-Specified.
9. Hajek P, Przulj D, Pesola F, Griffiths C, Walton R, McRobbie H, et al. Electronic cigarettes versus nicotine patches for smoking cessation in pregnancy: a randomized controlled trial. Nature medicine. 2022;28(5):958–64.
10. Przulj D, Pesola F, Myers Smith K, McRobbie H, Coleman T, Lewis S, et al. Helping pregnant smokers quit: a multi-centre randomised controlled trial of electronic cigarettes versus nicotine replacement therapy. Health technology assessment (Winchester, England). 2023;27(13):1–53.
11. Pesola F, Smith KM, Phillips-Waller A, Przulj D, Griffiths C, Walton R, et al. Safety of e-cigarettes and nicotine patches as stop-smoking aids in pregnancy: Secondary analysis of the Pregnancy Trial of E-cigarettes and Patches (PREP) randomized controlled trial. Addiction (Abingdon, England) [Internet]. 2024; Available from: <http://ovidsp.ovid.com/ovidweb.cgi?T=JS&PAGE=reference&D=medp&NEWS=N&AN=38229538>
12. Gonzalez-Roz A, MacKillop J. No evidence of differences in smoking levels, nicotine dependence, carbon monoxide or motivational indices between cigarette smokers and cigarette+e-cigarette dual users in two samples. Addictive behaviors. 2021; 112:106543.
13. Yoon W, Cho I, Cho SI. Understanding the role of e-cigarette use in smoking cessation based on the stages of change model. PloS one. 2022;17(9):e0274311.

## Dual-user (both cigarettes and ecigs) (n=11)

1. Czoli, Christine D., Fong, Geoffrey T., Goniewicz, Maciej L., and Hammond, David Biomarkers of exposure among "dual users" of tobacco cigarettes and electronic cigarettes in Canada. *Nicotine & tobacco research* 2018.
2. Palmer, Amanda M. and Brandon, Thomas H. Nicotine or expectancies? Using the balanced-placebo design to test immediate outcomes of vaping. *Addictive behaviors* 2019; 97:90-96.
3. Palmer, Amanda M. and Brandon, Thomas H. How do electronic cigarettes affect cravings to smoke or vape? Parsing the influences of nicotine and expectancies using the balanced-placebo design. *Journal of consulting and clinical psychology* 2018; 86(5):486-491.
4. Meltzer, Lauren R., Simmons, Vani N., Sutton, Steven K., Drobes, David J., Quinn, Gwendolyn P., et al. A randomized controlled trial of a smoking cessation self-help intervention for dual users of tobacco cigarettes and E-cigarettes: Intervention development and research design. *Contemporary clinical trials* 2017; 60:56-62.
5. NTR6224 Electronic cigarettes: an intervention for dual-users. *Http://www.who.int/trialsearch/trial2.aspx? Trialid=ntr6224* 2017.
6. NCT02792426 Nicotine Pharmacokinetics From Research Electronic Nicotine Delivery System S-TA-U001 in Smokers and E-Cigarette Users. [*Https://clinicaltrials.gov/show/nct02792426* 2016](Https://clinicaltrials.gov/show/nct02792426%202016).
7. Ozga-Hess, J. E.; Felicione, N. J.; Ferguson, S. G.; Dino, G.; Elswick, D.; Whitworth, C.; Turiano, N.; Blank, M. D. Piloting a clinical laboratory method to evaluate the influence of potential modified risk tobacco products on smokers' quit-related motivation, choice, and behavior. 2019
8. Persoskie, A.; O'Brien, E. K.; Poonai, K. Perceived relative harm of using e-cigarettes predicts future product switching among US adult cigarette and e-cigarette dual users. 2019
9. Wang-Schweig, M.; Jason, L. A.; Stevens, E.; Chaparro, J. Tobacco Use among Recovery Home Residents: Vapers Less Confident to Quit. 2019
10. Jackson, S. E.; Shahab, L.; West, R.; Brown, J. Associations between dual use of e-cigarettes and smoking cessation: A prospective study of smokers in England. 2020
11. Kim J Ph.D, M. P. H.; Lee, S. Daily Cigarette Consumption and Urine Cotinine Level between Dual Users of Electronic and Conventional Cigarettes, and Cigarette-Only Users . 2020

## Does not include the intervention of interest (n=93)

1. Bianco, Cynthia L., Pratt, Sarah I., Ferron, Joelle C., and Brunette, Mary F. Electronic cigarette use during a randomized trial of interventions for smoking cessation among Medicaid beneficiaries with mental illness. *Journal of dual diagnosis* 2019:1-8.
2. Maloney, Sarah F., Breland, Alison, Soule, Eric K., Hiler, Marzena, Ramoa, Carolina, Lipato, Thokozeni, and Eissenberg, Thomas Abuse liability assessment of an electronic cigarette in combustible cigarette smokers. *Experimental and clinical psychopharmacology* 2019.
3. Ni, Katherine, Wang, Binhuan, Link, Alissa, and Sherman, Scott Does smoking intensity predict cessation rates? A study of light-intermittent, light-daily, and heavy smokers enrolled in two telephone-based counseling interventions. *Nicotine & tobacco research* 2018.
4. National Academies of Sciences, Engineering and Medicine, Health and Medicine Division, Board on Population Health and Public Health Practice, and Committee on the Review of the Health Effects of Electronic Nicotine Delivery Systems Public Health Consequences of E-Cigarettes. *Washington (DC): National Academies Press (US)* 2018.
5. King, Andrea C., Smith, Lia J., McNamara, Patrick J., and Cao, Dingcai Second Generation Electronic Nicotine Delivery System Vape Pen Exposure Generalizes as a Smoking Cue. *Nicotine & tobacco research* 2018; 20(2):246-252.
6. Sumartiningsih, Sri, Lin, Hsin Fu, and Lin, Jung Charng Cigarette Smoking Blunts Exercise-Induced Heart Rate Response among Young Adult Male Smokers. *International journal of environmental research and public health* 2019; 16(6).
7. Yang, Bo, Spears, Claire Adams, and Popova, Lucy Psychological distress and responses to comparative risk messages about electronic and combusted cigarettes. *Addictive behaviors* 2019; 91:141-148.
8. Adriaens, Karolien, Gucht, Dinska Van, and Baeyens, Frank IQOSTM vs. e-Cigarette vs. Tobacco Cigarette: A Direct Comparison of Short-Term Effects after Overnight-Abstinence. *International journal of environmental research and public health* 2018; 15(12).
9. D'Ruiz, Carl D., O'Connell, Grant, Graff, Donald W., and Yan, X. Sherwin Measurement of cardiovascular and pulmonary function endpoints and other physiological effects following partial or complete substitution of cigarettes with electronic cigarettes in adult smokers. *Regulatory toxicology and pharmacology* 2017; 87:36-53.
10. Fearon, Ian M., Eldridge, Alison, Gale, Nathan, Shepperd, Christopher J., McEwan, Mike, Camacho, Oscar M., Nides, Mitch, McAdam, Kevin, and Proctor, Christopher J. E-cigarette Nicotine Delivery: Data and Learnings from Pharmacokinetic Studies. *American journal of health behavior* 2017; 41(1):16-32.
11. Hatsukami, Dorothy K., Luo, Xianghua, Dick, Laura, Kangkum, Margarita, Allen, Sharon S., Murphy, Sharon E., Hecht, Stephen S., Shields, Peter G., and al'Absi, Mustafa Reduced nicotine content cigarettes and use of alternative nicotine products: exploratory trial. *Addiction* 2017; 112(1):156-167.
12. Ludicke, Frank, Baker, Gizelle, Magnette, John, Picavet, Patrick, and Weitkunat, Rolf Reduced Exposure to Harmful and Potentially Harmful Smoke Constituents With the Tobacco Heating System 2.1. *Nicotine & tobacco research* 2017; 19(2):168-175.
13. Meier, Ellen, Wahlquist, Amy E., Heckman, Bryan W., Cummings, K. Michael, Froeliger, Brett, and Carpenter, Matthew J. A Pilot Randomized Crossover Trial of Electronic Cigarette Sampling Among Smokers. *Nicotine & tobacco research* 2017; 19(2):176-182.
14. Caponnetto, Pasquale, Maglia, Marilena, Cannella, Maria Concetta, Inguscio, Lucio, Buonocore, Mariachiara, Scoglio, Claudio, Polosa, Riccardo, and Vinci, Valeria Impact of Different e-Cigarette Generation and Models on Cognitive Performances, Craving and Gesture: A Randomized Cross-Over Trial (CogEcig). *Frontiers in psychology* 2017; 8:127-.
15. Martin, Florian, Talikka, Marja, Ivanov, Nikolai V., Haziza, Christelle, Hoeng, Julia, and Peitsch, Manuel C. Evaluation of the tobacco heating system 2.2. Part 9: Application of systems pharmacology to identify exposure response markers in peripheral blood of smokers switching to THS2.2. *Regulatory toxicology and pharmacology* 2016; 81 Suppl 2:S151-S157.
16. Haziza, Christelle, de La Bourdonnaye, Guillaume, Skiada, Dimitra, Ancerewicz, Jacek, Baker, Gizelle, Picavet, Patrick, and Ludicke, Frank Evaluation of the Tobacco Heating System 2.2. Part 8: 5-Day randomized reduced exposure clinical study in Poland. *Regulatory toxicology and pharmacology* 2016; 81 Suppl 2:S139-S150.
17. Haziza, Christelle, de La Bourdonnaye, Guillaume, Merlet, Sarah, Benzimra, Muriel, Ancerewicz, Jacek, Donelli, Andrea, Baker, Gizelle, Picavet, Patrick, and Ludicke, Frank Assessment of the reduction in levels of exposure to harmful and potentially harmful constituents in Japanese subjects using a novel tobacco heating system compared with conventional cigarettes and smoking abstinence: A randomized controlled study in confinement. *Regulatory toxicology and pharmacology* 2016; 81:489-499.
18. D'Ruiz, Carl D., Graff, Donald W., and Robinson, Edward Reductions in biomarkers of exposure, impacts on smoking urge and assessment of product use and tolerability in adult smokers following partial or complete substitution of cigarettes with electronic cigarettes. *BMC public health* 2016; 16:543-.
19. O'Connell, Grant, Graff, Donald W., and D'Ruiz, Carl D. Reductions in biomarkers of exposure (BoE) to harmful or potentially harmful constituents (HPHCs) following partial or complete substitution of cigarettes with electronic cigarettes in adult smokers. *Toxicology mechanisms and methods* 2016; 26(6):443-454.
20. McPherson, Sterling, Howell, Donelle, Lewis, Jennifer, Barbosa-Leiker, Celestina, Bertotti Metoyer, Patrick, and Roll, John Self-reported smoking effects and comparative value between cigarettes and high dose e-cigarettes in nicotine-dependent cigarette smokers. *Behavioural pharmacology* 2016; 27(2-3 Spec Issue):301-307.
21. Ludicke, Frank, Haziza, Christelle, Weitkunat, Rolf, and Magnette, John Evaluation of Biomarkers of Exposure in Smokers Switching to a Carbon-Heated Tobacco Product: A Controlled, Randomized, Open-Label 5-Day Exposure Study. *Nicotine & tobacco research* 2016; 18(7):1606-1613.
22. Antoniewicz, L., Brynedal, A., Hedman, L., Lundback, M., and Bosson, J. A. Acute Effects of Electronic Cigarette Inhalation on the Vasculature and the Conducting Airways. *Cardiovascular Toxicology* 2019.
23. Shiffman, S., Kurland, B. F., Scholl, S. M., and Mao, J. M. Nondaily Smokers' Changes in Cigarette Consumption with Very Low-Nicotine-Content Cigarettes: A Randomized Double-blind Clinical Trial. *JAMA Psychiatry* 2018; 75(10):995-1002.
24. Nollen, N. L., Cox, L. S., Mayo, M. S., Ellerbeck, E. F., Madhusudhana, S., and Ahluwalia, J. S. A randomized clinical trial of counseling and nicotine replacement therapy for treatment of African American non-daily smokers: Design, accrual, and baseline characteristics. *Contemporary clinical trials* 2018; 70:72-82.
25. Walele, T., Sharma, G., Savioz, R., Martin, C., and Williams, J. A randomised, crossover study on an electronic vapour product, a nicotine inhalator and a conventional cigarette. Part A: Pharmacokinetics. *Regulatory Toxicology and Pharmacology* 2016; 74:187-192.
26. King, A. C., Smith, L. J., Fridberg, D. J., Matthews, A. K., McNamara, P. J., and Cao, D. Exposure to Electronic Nicotine Delivery Systems (ENDS) Visual Imagery Increases Smoking Urge and Desire. *Psychology of Addictive Behaviors* 2016; 30(1):106-112.
27. Lopez, A. A., Hiler, M. M., Soule, E. K., Ramoa, C. P., Karaoghlanian, N. V., Lipato, T., Breland, A. B., Shihadeh, A. L., and Eissenberg, T. Effects of electronic cigarette liquid nicotine concentration on plasma nicotine and puff topography in tobacco cigarette smokers: A preliminary report. *Nicotine and Tobacco Research* 2016; 18(5):720-723.
28. Dawkins, L., Munafo, M., Christoforou, G., Olumegbon, N., and Soar, K. The Effects of E-Cigarette Visual Appearance on Craving and Withdrawal Symptoms in Abstinent Smokers. *Psychology of Addictive Behaviors* 2016; 30(1):101-105.
29. Rose, Jed E., Willette, Perry N., Loeback, Tanaia H., and Botts, David R. Evaluation of a botanical extract that mimics the respiratory cues of cigarette smoke. *Journal of smoking cessation* 2018.
30. Lochbuehler, Kirsten, Wileyto, E. Paul, Tang, Kathy Z., Mercincavage, Melissa, Cappella, Joseph N., and Strasser, Andrew A. Do current and former cigarette smokers have an attentional bias for e-cigarette cues? *Journal of Psychopharmacology* 2018; 32(3):316-323.
31. NCT03463837 Identify Biomarkers of Tobacco Exposure and Nicotine Uptake From JUUL 5% in Adult Smokers. *Https://clinicaltrials.gov/show/nct03463837* 2018.
32. Franzen, K. F., Willig, J., Cayo Talavera, S., Meusel, M., Sayk, F., Reppel, M., Dalhoff, K., Mortensen, K., and Droemann, D. E-cigarettes and cigarettes worsen peripheral and central hemodynamics as well as arterial stiffness: a randomized, double-blinded pilot study. *Vascular medicine* 2018; 23(5):419‐425-.
33. ACTRN12613000854730 TALANOA Samoa: a randomised controlled trial to evaluate the efficacy of a cessation support programme for smokers delivered via radio. *Http://www.who.int/trialsearch/trial2.aspx? Trialid=actrn12613000854730* 2013.
34. NTR5113 BLENDED SMOKING CESSATION TREATMENT. *Http://www.who.int/trialsearch/trial2.aspx? Trialid=ntr5113* 2015.
35. NCT02206737 The Effects of Electronic Cigarettes on the Microcirculation of the Hand. *Https://clinicaltrials.gov/show/nct02206737* 2014.
36. NCT03719391 United States Pre-Market Tobacco Application Pharmacokinetics. *Https://clinicaltrials.gov/show/nct03719391* 2018.
37. NCT03170674 CSD170501: study to Assess Biomarkers of Tobacco Exposure in Smokers During In-Clinic Confinement Switch to an Electronic Cigarette. *Https://clinicaltrials.gov/show/nct03170674* 2017.
38. NCT01454362 Effect of the Electronic Cigarette on Withdrawal Symptoms. *Https://clinicaltrials.gov/show/nct01454362* 2011.
39. ISRCTN69827722 Nicotine absorption from electronic cigarettes. *Http://www.who.int/trialsearch/trial2.aspx? Trialid=isrctn69827722* 2014.
40. ACTRN12618000849291 Assessment of the JUUL 5% Nicotine Salt Based ENDS Product, When Used by Current Smokers. *Http://www.who.int/trialsearch/trial2.aspx? Trialid=actrn12618000849291* 2018.
41. NCT01735487 CogEcig: cognitive Functioning and Electronic Cigarette. *Https://clinicaltrials.gov/show/nct01735487* 2012.
42. NCT02522156 Looming Vulnerability and Smoking Cessation Attempts. *Https://clinicaltrials.gov/show/nct02522156* 2015.
43. NCT03836573 Feasibility of a Decision Aid for E-Cigarettes in Primary Care. *Https://clinicaltrials.gov/show/nct03836573* 2019.
44. NCT03402243 Effect of Banning Menthol Flavorant on Cigarette and E-Cigarette Use. *Https://clinicaltrials.gov/show/nct03402243* 2018.
45. ISRCTN33423896 Study of effectiveness of a smartphone app for stopping smoking focused on use of nicotine replacement therapy. *Http://www.who.int/trialsearch/trial2.aspx? Trialid=isrctn33423896* 2015.
46. NCT03098004 Effects of e-Cigarettes on Nicotine Withdrawal. *Https://clinicaltrials.gov/show/nct03098004* 2017.
47. NCT01775787 Effects of Electronic Cigarettes on Nicotine Concentrations. *Https://clinicaltrials.gov/show/nct01775787* 2013.
48. ACTRN12618000800224 Assessment of JUUL 5% nicotine salt based Electronic Nicotine Delivery System (ENDS) products, when used by healthy adult smokers. *Http://www.who.int/trialsearch/trial2.aspx? Trialid=actrn12618000800224* 2018.
49. NCT01414998 Sensorimotor Replacement With Electronic and De-nicotinised Cigarettes. *Https://clinicaltrials.gov/show/nct01414998* 2011.
50. NCT02794220 A Study of Blood Levels of Nicotine Following an Electronic Cigarette. *Https://clinicaltrials.gov/show/nct02794220* 2016.
51. NCT02000921 Reduced Nicotine Content Cigarettes and Tobacco Switching Behaviors. *Https://clinicaltrials.gov/show/nct02000921* 2013.
52. NCT01665066 Pharmacokinetic Profile of Toxic Substances and Nicotine in Electronic Cigarettes. *Https://clinicaltrials.gov/show/nct01665066* 2012.
53. Stiles, M. F., Campbell, L. R., Jin, T., Graff, D. W., Fant, R. V., and Henningfield, J. E. Assessment of the abuse liability of three menthol Vuse Solo electronic cigarettes relative to combustible cigarettes and nicotine gum. *Psychopharmacology* 2018; 235(7):2077‐2086-.
54. Villanti, A. C., Rath, J. M., Williams, V. F., Pearson, J. L., Richardson, A., Abrams, D. B., Niaura, R. S., and Vallone, D. M. Impact of Exposure to Electronic Cigarette Advertising on Susceptibility and Trial of Electronic Cigarettes and Cigarettes in US Young Adults: a Randomized Controlled Trial. *Nicotine & Tobacco Research* 2016; 18(5):1331‐1339-.
55. NCT03037775 The Influence of Electronic and Traditional Cigarettes Smoking on Hemodynamic Parameters. *Https://clinicaltrials.gov/show/nct03037775* 2017.
56. NCT03060083 Switching to Very Low Nicotine Content Cigarettes vs Reducing Cigarettes Per Day. *Https://clinicaltrials.gov/show/nct03060083* 2017.
57. NCT00831155 Extinction Based Treatment for Nicotine Dependence. *Https://clinicaltrials.gov/show/nct00831155* 2009.
58. ISRCTN80651909 A study to examine changes in exposure to cigarette smoke chemicals when a smoker switches to using a tobacco heating product or an e-cigarette. *Http://www.who.int/trialsearch/trial2.aspx? Trialid=isrctn80651909*
59. Lüdicke, F., Baker, G., Magnette, J., Picavet, P., and Weitkunat, R. Reduced Exposure to Harmful and Potentially Harmful Smoke Constituents With the Tobacco Heating System 2.1. *Nicotine & Tobacco Research* 2017; 19(2):168‐175-.
60. Walele, T., Sharma, G., Savioz, R., Martin, C., and Williams, J. A randomised, crossover study on an electronic vapour product, a nicotine inhalator and a conventional cigarette. Part B: safety and subjective effects. *Regulatory toxicology and pharmacology* 2016; 74:193‐199-.
61. CA Oncken, MD Litt, LD McLaughlin, NA Burki Nicotine concentrations with electronic cigarette use: effects of sex and flavor. *Nicotine & tobacco research* 2015; 17(4).
62. Jahagirdar et al., Smoking Cessation Interventions for Patients with Severe Mental Illnesses: A Review of Clinical Effectiveness and Guidelines [Internet].
63. Scholtes et al., Effects of E-Cigarette Power and Nicotine Content in Dual Users and Vapers, NCT03830892.
64. Breland et al., The Effect of Electronic Cigarette Liquid Characteristics in Smokers, NCT03861078.
65. Whitted et al., Effects of e-Cigarettes on Perceptions and Behavior, NCT03742817.
66. Breland et al., Effects of Electronic Cigarette Settings and Liquid Concentrations in Cigarette Smokers and Electronic Cigarette Users, NCT03710590.
67. Breland et al., Assessment of Two New Electronic Cigarettes in Cigarette Smokers, NCT03435562.
68. Yoon et al., CSD170302: Study to Assess Nicotine Uptake in Smokers From Electronic Cigarettes, NCT03233997.
69. Lee et al., An Unblinded, Parallel, Randomized Study to Assess Nicotine Uptake in Smokers From Four Different Electronic Cigarettes, NCT03105804.
70. Middlekauff et al., E-cigarettes, Nicotine Inhaler, and Blood Vessel Function, NCT03072628.
71. Shingarev et al., Investigation of the Effects of Electronic Cigarettes on Vascular Health, NCT03041493.
72. Van de Borne et al., Comparison of Electronic Cigarettes and Tobacco Cigarettes on Cardiovascular Function and Oxidative Stress, NCT03036644.
73. Semmelweis University Epidemiology and Acute Oral Effects Electronic Cigarette, NCT03011710.
74. Bosson et al., Acute Cardiorespiratory Effects of E-cigarette Inhalation, NCT02899234.
75. Dupont et al., Determination of Carcinogens Nicotine in Electronic Cigarettes Users, NCT02897401.
76. MOUBARAK et al., Study of the Immediate Cardiovascular Effects of Electronic Cigarette in Subjects With Cardiovascular Disease, NCT02777515.
77. Middlekauff et al., Arterial Inflammation and E-Cigarettes, NCT02734888.
78. Williams et al., Comparison of Pharmacokinetic Parameters Between Adopters of Electronic Cigarettes and a Historical Sample of Combustible Cigarette Smokers, NCT02730676.
79. Shyamsundar et al., The Study of ELEctronic Cigarette Toxicity in a Human Model in Vivo Model of Inflammation and Vascular Dysfunction, NCT02739438.
80. Middlekauff et al., E-cigarettes and Blood Vessel Function, NCT02740595.
81. Katz et al., Effect of Electronic Cigarettes on Platelets, Endothelium and Inflammation, NCT02662075.
82. Middlekauff et al., E-Cigarettes and SNA, NCT02724241.
83. See, J. H. J.; Yong, T. H.; Poh, S. L. K.; Lum, Y. C. Smoker motivations and predictors of smoking cessation: lessons from an inpatient smoking cessation programme. 2019
84. Mahabee-Gittens EM, Ammerman RT, Khoury JC, Tabangin ME, Ding L, Merianos AL, et al. A Parental Smoking Cessation Intervention in the Pediatric Emergency Setting: A Randomized Trial. International journal of environmental research and public health [Internet]. 2020;17(21). Available from: <http://ovidsp.ovid.com/ovidweb.cgi?T=JS&PAGE=reference&D=med18&NEWS=N&AN=33158230>
85. Morphett K, Fraser D, Borland R, Hall W, Walker N, Bullen C, et al. A Pragmatic Randomized Comparative Trial of e-Cigarettes and Other Nicotine Products for Quitting or Long-Term Substitution in Smokers. Nicotine & tobacco research: official journal of the Society for Research on Nicotine and Tobacco. 2022;24(7):1079–88.
86. Li X, Holahan CK, Loukas A, Holahan CJ, Pasch KE, Marti CN. Alternative Tobacco Use and Cigarette Smoking Transitions among College Students in Texas. Substance use & misuse. 2023;58(3):389–96.
87. Saxena A, Baskerville NB, Garcia JM. Association of e-cigarette use and smoking cessation among Canadian young adult smokers: Secondary analysis of data from a randomised controlled trial. Journal of Smoking Cessation. 2020;15(1):50–8.
88. Graham AL, Amato MS, Cha S, Jacobs MA, Bottcher MM, Papandonatos GD. Effectiveness of a Vaping Cessation Text Message Program Among Young Adult e-Cigarette Users: A Randomized Clinical Trial. JAMA internal medicine. 2021;181(7):923–30.
89. Elling JM, Crutzen R, Talhout R, de Vries H. Effects of providing tailored information about e-cigarettes in a digital smoking cessation intervention: randomized controlled trial. Health education research. 2023;38(2):150–62.
90. Bricker JB, Watson NL, Mull KE, Sullivan BM, Heffner JL. Efficacy of Smartphone Applications for Smoking Cessation: A Randomized Clinical Trial. JAMA internal medicine. 2020;180(11):1472–80.
91. McRobbie HJ, Phillips-Waller A, El Zerbi C, McNeill A, Hajek P, Pesola F, et al. Nicotine replacement treatment, e-cigarettes and an online behavioural intervention to reduce relapse in recent ex-smokers: a multinational four-arm RCT. Health technology assessment (Winchester, England). 2020;24(68):1–82.
92. Watson NL, Mull KE, Bricker JB. The association between frequency of e-cigarette use and long-term smoking cessation outcomes among treatment-seeking smokers receiving a behavioral intervention. Drug and alcohol dependence. 2021; 218:108394.
93. Santiago-Torres M, Mull KE, Sullivan BM, Bricker JB. Use of e-Cigarettes in Cigarette Smoking Cessation: Secondary Analysis of a Randomized Controlled Trial. JMIR mHealth and uHealth. 2023;11:e48896.

## Does not include a comparator of interest (n=20)

1. Pearson, Jennifer L., Smiley, Sabrina L., Rubin, Leslie F., Anesetti-Rothermel, Andrew, Elmasry, Hoda, Davis, Megan, DeAtley, Teresa, Harvey, Emily, Kirchner, Thomas, and Abrams, David B. The Moment Study: protocol for a mixed method observational cohort study of the Alternative Nicotine Delivery Systems (ANDS) initiation process among adult cigarette smokers. *BMJ open* 2016; 6(4):e011717-.
2. NCT01194583 Efficacy and Safety of an Electronic Nicotine Delivery Device (E-Cigarette) Without Nicotine Cartridges. [*https://clinicaltrials.gov/show/nct01194583*](https://clinicaltrials.gov/show/nct01194583)
3. NCT03743532 Preliminary Evaluation of Alternative Approaches to Combustible Cigarette Cessation (Exchange Project Sub-Study). [*https://clinicaltrials.gov/show/nct03743532*](https://clinicaltrials.gov/show/nct03743532)
4. A multi-centre study to evaluate the safety of use of electronic vapour products for two years. *clinicaltrials.gov/show/NCT02143310*
5. Acceptability, patterns of use and safety of electronic cigarette in people with mental illness: a pilot study. *clinicaltrials.gov/show/NCT02212041*
6. Bailey et al., Monitoring the Transition From Smoking to E-cigarettes, NCT02752022
7. Martner, S. G.; Dallery, J. Technology-based contingency management and e-cigarettes during the initial weeks of a smoking quit attempt. 2019
8. Yingst, J.; Foulds, J.; Zurlo, J.; Steinberg, M. B.; Eissenberg, T.; Du, P. Acceptability of electronic nicotine delivery systems (ENDS) among HIV positive smokers. 2019
9. Smith, T. T.; Heckman, B. W.; Wahlquist, A. E.; Cummings, K. M.; Carpenter, M. J. The Impact of E-liquid Propylene Glycol and Vegetable Glycerin Ratio on Ratings of Subjective Effects, Reinforcement Value, and Use in Current Smokers . 2020
10. Lee, S. H.; Ahn, S. H.; Cheong, Y. S. Effect of Electronic Cigarettes on Smoking Reduction and Cessation in Korean Male Smokers: A Randomized Controlled Study . 2019
11. Hajek, P.; Phillips-Waller, A.; Przulj, D.; Pesola, F.; Smith, K. M.; Bisal, N.; Li, J.; Parrott, S.; Sasieni, P.; Dawkins, L.; Ross, L.; Goniewicz, M.; Wu, Q.; McRobbie, H. J. E-cigarettes compared with nicotine replacement therapy within the UK Stop Smoking Services: the TEC RCT . 2019
12. Cox, S.; Dawkins, L.; Doshi, J.; Cameron, J. Effects of e-cigarettes versus nicotine replacement therapy on short-term smoking abstinence when delivered at a community pharmacy . 2019
13. Caponnetto, P.; DiPiazza, J.; Cappello, G. C.; Demma, S.; Maglia, M.; Polosa, R. Multimodal Smoking Cessation in a Real-Life Setting: Combining Motivational Interviewing With Official Therapy and Reduced Risk Products . 2019
14. Peechatka, A. L.; Molokotos, E. K.; Zegel, M.; Lukas, S. E.; Janes, A. C. A Preliminary Examination of Nicotine-Free Electronic Cigarette Use During Cessation From Combustible Cigarettes . 2019
15. Chiang, S. C.; Abroms, L. C.; Cleary, S. D.; Pant, I.; Doherty, L.; Krishnan, N. E-cigarettes and smoking cessation: a prospective study of a national sample of pregnant smokers . 2019
16. Cioe, P. A.; Mercurio, A. N.; Lechner, W.; Costantino, C. C.; Tidey, J. W.; Eissenberg, T.; Kahler, C. W. A pilot study to examine the acceptability and health effects of electronic cigarettes in HIV-positive smokers . 2020
17. McEwan M, Gale N, Ebajemito JK, Camacho OM, Hardie G, Proctor CJ, et al. A randomized controlled study in healthy participants to explore the exposure continuum when smokers switch to a tobacco heating product or an E-cigarette relative to cessation. Toxicology reports. 2021;8:994–1001.
18. Kimber C, Sideropoulos V, Cox S, Frings D, Naughton F, Brown J, et al. E-cigarette support for smoking cessation: Identifying the effectiveness of intervention components in an on-line randomized optimization experiment. Addiction (Abingdon, England). 2023;118(11):2105–17.
19. Rubenstein D, Sokolovsky AW, Aston ER, Nollen NL, Schmid CH, Rice M, et al. Predictors of smoking reduction among African American and Latinx smokers in a randomized controlled trial of JUUL e-cigarettes. Addictive behaviors. 2021;122:107037.
20. Bonevski B, Manning V, Wynne O, Gartner C, Borland R, Baker AL, et al. QuitNic: A Pilot Randomized Controlled Trial Comparing Nicotine Vaping Products With Nicotine Replacement Therapy for Smoking Cessation Following Residential Detoxification. Nicotine & tobacco research : official journal of the Society for Research on Nicotine and Tobacco. 2021;23(3):462–70.

## Setting not of interest (n=7)

1. Guillaumier, Ashleigh, Manning, Victoria, Wynne, Olivia, Gartner, Coral, Borland, Ron, Baker, Amanda L., Segan, Catherine J., Skelton, Eliza, Moore, Lyndell, Bathish, Ramez, Lubman, Dan I., and Bonevski, Billie Electronic nicotine devices to aid smoking cessation by alcohol- and drug-dependent clients: protocol for a pilot randomised controlled trial. *Trials* 2018; 19(1):415-.
2. Halpern, Scott D., Harhay, Michael O., Saulsgiver, Kathryn, Brophy, Christine, Troxel, Andrea B., and Volpp, Kevin G. A Pragmatic Trial of E-Cigarettes, Incentives, and Drugs for Smoking Cessation. *The New England journal of medicine* 2018; 378(24):2302-2310.
3. Kastaun, Sabrina, Brown, Jamie, Brose, Leonie S., Ratschen, Elena, Raupach, Tobias, Nowak, Dennis, Cholmakow-Bodechtel, Constanze, Shahab, Lion, West, Robert, and Kotz, Daniel Study protocol of the German Study on Tobacco Use (DEBRA): a national household survey of smoking behaviour and cessation. *BMC public health* 2017; 17(1):378-.
4. Lee, Susan M., Tenney, Rachel, Wallace, Arthur W., and Arjomandi, Mehrdad E-cigarettes versus nicotine patches for perioperative smoking cessation: a pilot randomized trial. *PeerJ* 2018; 6:e5609-.
5. Kumral, T. L., Salturk, Z., Yildirim, G., Uyar, Y., Berkiten, G., Atar, Y., and Inan, M. How does electronic cigarette smoking affect sinonasal symptoms and nasal mucociliary clearance? *B-ENT* 2016; 12(1):17-21.
6. KCT0001277 Effect of An Electronic Cigarette for Smoking Reduction and Cessation in Korean Male Smokers: a Randomized, Controlled Study. *Http://www.who.int/trialsearch/trial2.aspx? Trialid=kct0001277*.
7. Heiden BT, Baker TB, Smock N, Pham G, Chen J, Bierut LJ, et al. Assessment of formal tobacco treatment and smoking cessation in dual users of cigarettes and e-cigarettes. Thorax [Internet]. 2022; Available from: <http://ovidsp.ovid.com/ovidweb.cgi?T=JS&PAGE=reference&D=medp&NEWS=N&AN=35863765>

## Relevant study, but does not provide outcomes (as defined in protocol) (n=21)

1. Tucker, Megan R., Laugesen, Murray, Bullen, Chris, and Grace, Randolph C. Predicting Short-Term Uptake of Electronic Cigarettes: Effects of Nicotine, Subjective Effects, and Simulated Demand. *Nicotine & tobacco research* 2018; 20(10):1265-1271.
2. Li, J.; Hajek, P.; Pesola, F.; Wu, Q.; Phillips-Waller, A.; Przulj, D.; Myers Smith, K.; Bisal, N.; Sasieni, P.; Dawkins, L.; et al. Cost-effectiveness of e-cigarettes compared with nicotine replacement therapy in stop smoking services in England (TEC study): a randomised controlled trial. 2019
3. Li, J.; Hajek, P.; Pesola, F.; Wu, Q.; Phillips-Waller, A.; Przulj, D.; Myers Smith, K.; Bisal, N.; Sasieni, P.; Dawkins, L.; Ross, L.; Goniewicz, M. L.; McRobbie, H.; Parrott, S. Cost-effectiveness of e-cigarettes compared with nicotine replacement therapy in stop smoking services in England (TEC study): a randomized controlled trial. 2020
4. Li, J.; Hajek, P.; Pesola, F.; Wu, Q.; Phillips‐Waller, A.; Przulj, D.; Myers Smith, K.; Bisal, N.; Sasieni, P.; Dawkins, L.; et al. Cost‐effectiveness of e‐cigarettes compared with nicotine replacement therapy in stop smoking services in England (TEC study): a randomized controlled trial. 2020.
5. Krishnan N, Berg CJ, Le D, Ahluwalia J, Graham AL, Abroms LC. A pilot randomized controlled trial of automated and counselor-delivered text messages for e-cigarette cessation. Tobacco prevention & cessation. 2023;9:04.
6. Okuyemi KS, Ojo-Fati O, Aremu TO, Friedrichsen SC, Grude L, Oyenuga M, et al. A Randomized Trial of Nicotine versus No-nicotine E-cigarettes Among African American Smokers: Changes in Smoking and Tobacco Biomarkers. Nicotine & tobacco research : official journal of the Society for Research on Nicotine and Tobacco. 2022;24(4):555–63.
7. Mahoney MC, Rivard C, Kimmel HL, Hammad HT, Sharma E, Halenar MJ, et al. Cardiovascular Outcomes among Combustible-Tobacco and Electronic Nicotine Delivery System (ENDS) Users in Waves 1 through 5 of the Population Assessment of Tobacco and Health (PATH) Study, 2013-2019. International journal of environmental research and public health [Internet]. 2022;19(7). Available from: <http://ovidsp.ovid.com/ovidweb.cgi?T=JS&PAGE=reference&D=med22&NEWS=N&AN=35409819>
8. Yingst J, Wang X, Lopez AA, Breland A, Soule E, Barnes A, et al. Changes in Nicotine Dependence Among Smokers Using Electronic Cigarettes to Reduce Cigarette Smoking in a Randomized Controlled Trial. Nicotine & tobacco research : official journal of the Society for Research on Nicotine and Tobacco. 2023;25(3):372–8.
9. Nga JDL, Hakim SL, Bilal S. Comparison of End Tidal Carbon Monoxide Levels between Conventional Cigarette, Electronic Cigarette and Heated Tobacco Product among Asiatic Smokers. Substance use & misuse. 2020;55(12):1943–8.
10. Goldberg Scott S, Feigelson H, Powers J, Clennin M, Lyons J, Gray M, et al. DEMOGRAPHIC, CLINICAL, AND BEHAVIORAL FACTORS ASSOCIATED WITH E-CIGARETTE USE IN A LARGE COHORT IN THE UNITED STATES. Chest. 2021;160(4 Supplement):A2451.
11. Goldberg Scott S, Feigelson HS, Powers JD, Clennin MN, Lyons JA, Gray MT, et al. Demographic, Clinical, and Behavioral Factors Associated With Electronic Nicotine Delivery Systems Use in a Large Cohort in the United States. Tobacco use insights. 2023;16:1179173X221134855.
12. Ng G, Attwells S, Selby P, Zawertailo L. Effectiveness of Non-Nicotinic E-Cigarettes to Reduce Cue- and Abstinence-Induced Cigarette Craving in Non-Treatment Seeking Daily Dependent Smokers. Psychopharmacology. 2021;238(6):1461–72.
13. Gravely S, Meng G, Hammond D, Reid JL, Seo YS, Hyland A, et al. Electronic nicotine delivery systems (ENDS) flavours and devices used by adults before and after the 2020 US FDA ENDS enforcement priority: findings from the 2018 and 2020 US ITC Smoking and Vaping Surveys. Tobacco control. 2022;31(Suppl 3):s167–75.
14. Prell C, Hebert-Losier A, Filion KB, Reynier P, Eisenberg MJ. Evaluating the impact of varying expired carbon monoxide thresholds on smoking relapse identification: insights from the E3 trial on e-cigarette efficacy for smoking cessation. BMJ open. 2023;13(10):e071099.
15. Goebel I, Mohr T, Axt PN, Watz H, Trinkmann F, Weckmann M, et al. Impact of Heated Tobacco Products, E-Cigarettes, and Combustible Cigarettes on Small Airways and Arterial Stiffness. Toxics [Internet]. 2023;11(9). Available from: <http://ovidsp.ovid.com/ovidweb.cgi?T=JS&PAGE=reference&D=medp&NEWS=N&AN=37755768>
16. Klonizakis M, Gumber A, McIntosh E, Brose LS. Medium- and longer-term cardiovascular effects of e-cigarettes in adults making a stop-smoking attempt: a randomized controlled trial. BMC medicine. 2022;20(1):276.
17. Nyilas S, Bauman G, Korten I, Pusterla O, Singer F, Ith M, et al. MRI Shows Lung Perfusion Changes after Vaping and Smoking. Radiology. 2022;304(1):195–204.
18. Guo Y, Li S, Wang Z, Jiang F, Guan Y, Huang M, et al. Nicotine Delivery and Pharmacokinetics of an Electronic Cigarette Compared With Conventional Cigarettes in Chinese Adult Smokers: A Randomized Open-Label Crossover Clinical Study. Nicotine and Tobacco Research. 2022;24(12):1881–8.
19. Heron M, Le Faou AL, Ibanez G, Metadieu B, Melchior M, El-Khoury Lesueur F. Smoking cessation using preference-based tools: a mixed method pilot study of a novel intervention among smokers with low socioeconomic position. Addiction science & clinical practice. 2021;16(1):43.
20. Rose JE, Frisbee S, Campbell D, Salley A, Claerhout S, Davis JM. Smoking reduction using electronic nicotine delivery systems in combination with nicotine skin patches. Psychopharmacology. 2023;240(9):1901–9.
21. Manning K, Mayorga NA, Garey L, Kauffman BY, Buckner JD, Zvolensky MJ. The Role of Anxiety Sensitivity and Fatigue Severity in Predicting E-Cigarette Dependence, Barriers to Cessation, and Cravings among Young Adults. Substance use & misuse. 2021;56(13):2059–65.

## Exclusions after amendments to the protocol

### Comparator not of interest (n=3)

1. Tseng, Tuo Yen, Ostroff, Jamie S., Campo, Alena, Gerard, Meghan, Kirchner, Thomas, Rotrosen, John, and Shelley, Donna. A Randomized Trial Comparing the Effect of Nicotine Versus Placebo Electronic Cigarettes on Smoking Reduction Among Young Adult Smokers. *Nicotine & Tobacco Research* 2016; 18(10):1937-1943. *Control group received telephone counseling session with a trained tobacco cessation counselor. The purpose of the telephone counseling was to review current smoking patterns and offer behavioural and environmental change strategies. These included specific smoking reduction options.*
2. Hajek, Peter, Phillips-Waller, Anna, Przulj, Dunja, Pesola, Francesca, Myers Smith, Katie, Bisal, Natalie, Li, Jinshuo, Parrott, Steve, Sasieni, Peter, Dawkins, Lynne, Ross, Louise, Goniewicz, Maciej, Wu, Qi, and McRobbie, Hayden J. A Randomized Trial of E-Cigarettes versus Nicotine-Replacement Therapy. *NEJM* 2019; 380(7):629-637. *Comparison group received nicotine-replacement of their choice (e.g., patch, gum, lozenge, nasal spray, inhalator, mouth spray, mouth strip, and microtabs).*
3. Veldheer, Susan, Yingst, Jessica, Midya, Vishal, Hummer, Breianna, Lester, Court, Krebs, Nicolle, Hrabovsky, Shari, Wilhelm, Ashley, Liao, Jason, Yen, Miao Shan, Cobb, Caroline, Eissenberg, Thomas, and Foulds, Jonathan. Pulmonary and other health effects of electronic cigarette use among adult smokers participating in a randomized controlled smoking reduction trial. *Addictive Behaviors* 2019; 91:95-101. *Comparison group was given a non-electronic cigarette substitute (a plastic tube that resembles a cigarette). It has an adjustable draw, but it does not have any electronic parts and it does not contain nicotine or emit any aerosol.*

### Participants with co-morbidities (n=4)

1. Felicione, Nicholas J., Enlow, Paul, Elswick, Daniel, Long, Dustin, Sullivan, C. R., Blank, Melissa D. A pilot investigation of the effect of electronic cigarettes on smoking behavior among opioid-dependent smokers. *Addictive Behaviors* 2019; 91:45-50. *Comorbidity: Opioid-dependent smokers.*
2. Polosa, Riccardo, Morjaria, Jaymin Bhagwanji, Caponnetto, Pasquale, Prosperini, Umberto, Russo, Cristina, Pennisi, Alfio, and Bruno, Cosimo Marcello. Evidence for harm reduction in COPD smokers who switch to electronic cigarettes. *Respiratory Research* 2016; 17:166. *Comorbidity: Patients with COPD.*
3. Polosa, Riccardo, Morjaria, Jaymin B., Caponnetto, Pasquale, Battaglia, Eliana, Russo, Cristina, Ciampi, Claudio, Adams, George, and Bruno, Cosimo M. Blood Pressure Control in Smokers with Arterial Hypertension Who Switched to Electronic Cigarettes. *International Journal of Environmental Research and Public Health* 2016; 13:1123. *Comorbidity: Patients with arterial hypertension.*
4. Russo, Cristina, Cibella, Fabio, Mondati, Enrico, Caponnetto, Pasquale, Frazzetto, Evelise, Caruso, Massimo, Caci, Grazia, and Polosa, Riccardo. Lack of Substantial Post-Cessation Weight Increase in Electronic Cigarettes Users. *International Journal of Environmental Research and Public Health* 2018; 15:581. *Comorbidity: Patients with cardiorespiratory conditions*
