# Supplementary material for: Effectiveness of e-cigarettes as a stop smoking intervention in adults: a systematic review
Source: Syst Rev. 2024 Jun 29;13:168. doi: 10.1186/s13643-024-02572-7 (PMC11218295; doi:10.1186/s13643-024-02572-7)
Supplement: Supplementary file 5 — Additional file 5: Appendix 5. Tobacco effect judgements. [file 13643_2024_2572_MOESM5_ESM.docx]

# Objectives

- The goal of this exercise is to make contextualized judgments on effect size, taking into account all outcomes for a given intervention/comparator, patient and WG outcome rating, and other contextual factors as necessary (e.g., nature of the intervention, demands of intervention on all parties, etc.).
- The judgments made during this exercise inform two important elements to the SR:
  - GRADE imprecision: If the point estimate and confidence intervals represent different effect sizes (e.g., the CI ranges from a small benefit to a large benefit), then the estimate is considered imprecise.
  - GRADE summary statements: The size of the point estimate is what determines our GRADE summary statement, as it dictates what we are determining our certainty in. For example, if the point estimate is judged to be a moderate benefit, we are rating our certainty that intervention x results in a moderate benefit for the outcome. If, for example, the point estimate is moderate, but the CI ranges from small benefit to large benefit, the ERSC may rate down by one for imprecision, and assuming no other issues with GRADE we would conclude that we have moderate certainty that intervention x results in a moderate benefit for the outcome.

# Procedure

- Judgments on effect size are to be made using the **absolute difference** between intervention and control groups. GRADE discourages the use of relative effects to make these judgments, since the same relative effect could have drastically different absolute impacts depending on the baseline risk of an outcome.
- Judgments must be made for the point estimate, lower confidence interval (CI), and upper CI **separately**. The rater should judge the size of effect for each estimate, **as if that given integer represents the true effect.**
- This is done for each outcome for a given intervention/comparator and population.
- Contextualizing the judgment of effect size – Factors that may impact the judgment:
  - **Magnitude of health benefit from successful quitting.** To assess the benefit for the smoking cessation outcome, it is important to consider the downstream health benefits of successful quitting. Quitting at 30 years of age increases life expectancy by a decade while quitting at 40 and 50 years of age increases expectancy by 9 and 6 years, respectively (1). For every two individuals who quit smoking tobacco, one will avoid a tobacco-related death (2). Quitting before age 40 reduces the excess risk of premature disease and death by 90% (3). Ten years after quitting smoking, the risk of developing lung cancer is cut in half and the risk of cancers of the mouth, throat, esophagus, bladder, cervix, and pancreas are decreased (4,5). Risk for coronary heart disease decreases by half after 1 year and is at normal levels by 15 years (4). Risk of stroke is also greatly reduced after 2-4 years (4). Quitting smoking also leads to improvements in mental health, overall quality of life (4). People who quit smoking after having a heart attack reduce their risk of having a second heart attack by about half (6).

Therefore, the judgment of benefit for smoking cessation for a given intervention is in large part a product of the increased likelihood of quitting, and the large anticipated health benefit that would follow (e.g., a 10 in 1000 increased chance of quitting is a 10 in 1000 chance of obtaining the above health benefits and may therefore be considered an important benefit despite the small absolute increase in likelihood of quitting). The judgement may also be influenced by other risk factors. For example, there may be a differential benefit for those with mental health conditions (e.g., schizophrenia) given higher rates of smoking and smoking-related mortality, greater levels of nicotine dependence, and greater challenges with quitting in some of these populations (7).

- - **Harms related to smoking.** Related to the above, previous discussions with our experts have indicated that almost *any* increase in quit rate is important given how harmful it is to continue smoking. Men who smoke are 22 times more likely to get cancer, while women who smoke are at 12 times the risk (4). Tobacco smoking is a cause of cancers of the mouth and throat, lungs, liver, colon and rectum, stomach, bladder, blood, pancreas, kidneys, and cervix (8). Tobacco smoking is also a cause of metabolic disorders such as diabetes (8). The long list of conditions linked to smoking also includes blindness, cataracts, pneumonia, erectile dysfunction, reduced immune function, and overall diminished health (8). These harms are not captured in our review, but should be considered. This should also be considered in the case where an intervention may reduce the likelihood of quitting (i.e., effect is on the side of harms).
  - **Trade off of benefits versus harms/ adverse effects of the intervention.** For example, if two interventions both increase smoking quit rate by 5%, but one has more identified harms than the other, you may rate the effect sizes differently. Similarly, if two interventions have similar adverse events, but one has a great effect on benefits, you may judge the effect sizes for those adverse event outcomes differently.
  - **Importance and nature of the outcome** (see outcome rating below). For example, a 10% absolute increase in a very important outcome (e.g., death) may be considered large, whereas a 10% increase in a less important outcome (e.g., headache) may be considered trivial.
  - **Other elements of the intervention not captured under recorded harms/adverse events**. For example, the same absolute effect might be judged differently for two different interventions if one is clearly more invasive or resource intensive.

**References**

1. Jha P, Peto R. Global effects of smoking, of quitting, and of taxing tobacco. N Engl J Med. 2014 Jan 2; 370(1):60-8.
2. Lam TH. Absolute risk of tobacco deaths: one in two smokers will be killed by smoking: comment on "Smoking and all-cause mortality in older people". Arch Intern Med. 2012 Jun 11; 172(11):845-6.
3. Reid R, Pritchard G, Walker K, Aitken D, Mullen K, Pipe A. Managing smoking cessation. CMAJ. 2016 Dec;188(17-18):E484-E492.
4. Patnode CD, Henderson JT, Thompson JH, Senger CA, Fortmann SP, Whitlock EP. Behavioral Counseling and Pharmacotherapy Interventions for Tobacco Cessation in Adults, Including Pregnant Women: A Review of Reviews for the U.S. Preventive Services Task Force. 2015 Sep.
5. Word Health Organization. Tobacco Free Initiative (TFI): Fact sheet about health benefits of smoking cessation. 2018. Available at: <http://www.who.int/tobacco/quitting/benefits/en/>
6. Stead LF, Buitrago D, Preciado N, Sanchez G, Hartmann-Boyce J, Lancaster T. Physician advice for smoking cessation. Cochrane Database of Systematic Reviews 2013, Issue 5. Art. No.: CD000165.
7. Caponnetto, P., Polosa, R., Robson, D., & Bauld, L. (2020). Tobacco smoking, related harm and motivation to quit smoking in people with schizophrenia spectrum disorders. Health psychology research, 8(1), 9042.
8. U.S. Department of Health and Human Services. The Health Consequences of Smoking—50 Years of Progress: A Report of the Surgeon General. Atlanta, GA: U.S. Department of Health and Human Services, Centers for Disease Control and Prevention, National Center for Chronic Disease Prevention and Health Promotion, Office on Smoking and Health; 2014. Accessed at www.surgeongeneral.gov/library/reports/50-years-of-progress/full-report.pdf

Table of Contents

[Objectives 1](#_Toc91758221)

[Procedure 1](#_Toc91758222)

[E-cigarettes 4](#_Toc91758223)

[1. E-cigarettes with nicotine versus No intervention or waitlist in general/mixed population 4](#_Toc91758224)

[2. E-cigarettes with nicotine plus usual care versus Usual care in patients with periodontitis 6](#_Toc91758225)

[3. E-cigarettes with nicotine plus standard care^a^ versus E-cigarettes without nicotine plus standard care in smokers willing to quit 8](#_Toc91758226)

[4. E-cigarettes with nicotine^a^ versus E-cigarettes without nicotine (placebo) in smokers NOT motivated/wishing to quit 9](#_Toc91758227)

[5. E-cigarettes with nicotine^a^ versus E-cigarettes without nicotine in smokers NOT willing to quit 10](#_Toc91758228)

[6. E-cigarette with nicotine + other smoking cessation treatment (behavioural support) vs E-cigarette with no nicotine + other smoking cessation treatment (behavioural support) in smokers willing to quit 12](#_Toc91758229)

[7. E-cigarette with nicotine + other smoking cessation treatment (behavioural support) vs No intervention + other smoking cessation treatment (behavioural support) in smokers willing to quit 15](#_Toc91758230)

[8. E-cigarette with nicotine + other smoking cessation treatment (behavioural support) + standard care (nicotine patch) vs. E-cigarette with no nicotine + other smoking cessation treatment (behavioural support) + standard care (nicotine patch) in smokers willing to quit within the next 2 weeks 16](#_Toc91758231)

[9. E-cigarette with nicotine + other smoking cessation treatment (behavioural support) + standard care (nicotine patch) vs. Other smoking cessation treatment (behavioural support) + standard care (nicotine patch) in smokers willing to quit within the next 2 weeks 17](#_Toc91758232)

[10. E-cigarette with no nicotine + other smoking cessation treatment (behavioural support) + standard care (nicotine patch) vs. Other smoking cessation treatment (behavioural support) + standard care (nicotine patch) in smokers willing to quit within the next 2 weeks 19](#_Toc91758233)

[11. E-cigarette with no nicotine + other smoking cessation treatment (behavioural support) vs. Other smoking cessation treatment (behavioural support) in smokers willing to quit 20](#_Toc91758234)

# E-cigarettes

| E-cigarettes with nicotine versus no intervention, waitlist, or usual care, in general/mixed population willing to quit | | | | | | | | | | | | | |
| --- | --- | --- | --- | --- | --- | --- | --- | --- | --- | --- | --- | --- | --- |
| **Outcome(s)**  **(Follow up (fu): time)**  **# of participants (# studies)** | | **Median Patient rating** | **Mean WG rating** | **Relative effect (95%CI)** | **Baseline (control) risk** | | | **Absolute difference with intervention** | **Assessment of absolute difference** | | | | **Certainty** |
|  |  |  |  |  |  |  |  |  | **Point estimate** | **Lower 95% CI** | **Upper 95%CI** | **Comments on judgment** |  |
| 1 | *Versus usual care, participants had periodontitis*  Smoking cessation (fu: 6 mo)^a^  80 (1 RCT) | 8/10 (critical) | 8.8/10 (critical) | RR 3.00 (0.64 to 13.98) | 50 per 1,000 | | | **100 more per 1,000** (from 18 fewer to 649 more) | Large benefit | Small but important harm | Large benefit | Point estimate and upper CI represent large benefits given baseline risk, and health benefits associated with quitting. Lower CI results in fewer individuals quitting smoking and is therefore judged as a small harm due to the harms of continued smoking. Small likelihood of adverse events identified below, but not considered sufficient to change judgment for point estimate or upper CI. | ⨁◯◯◯  VERY LOW |
| 2 | *Versus usual care, participants had periodontitis*  Smoking reduction (salivary cotinine)^a^ (fu: 6 mo)  58 (1 RCT) | 8/10 (critical) | 8.8/10 (critical) | **-** | The mean reduction from baseline in tobacco smoking frequency/quantity - Salivary cotinine was 37.1 ng/mL | | | MD **25.1 ng/mL lower**  (93.48 lower to 43.28 higher) | Little to no difference | Small but important benefit | Small but important harm | Point estimate is judged as little to no difference as it appears to indicate only a very small reduction in nicotine intake. Upper and lower CI could represent more important changes in smoking and are judged as a small but important benefit and harm, respectively, taking into account few additional potential harms of the intervention.  A daily smoker will typically have a level of about 300ng/mL and a cut-off for non-smoking is 15ng/mL  https://academic.oup.com/ntr/article/4/2/149/1013220  Note that cotinine is a metabolite of nicotine and is thus a biomarker of nicotine exposure which gives high readings in those using tobacco, e-cigarettes or other nicotine replacement products. So is an indicator of nicotine intake, not necessarily smoking reduction in the case where tobacco is replaced with nicotine e-cigarettes. | ⨁◯◯◯  VERY LOW |
|  | *Versus usual care, participants had periodontitis*  Smoking reduction (salivary anabasine)^a^  (fu: 6 mo)  58 (1 RCT) | 8/10 (critical) | 8.8/10 (critical) | **-** | The mean increase from baseline in tobacco smoking frequency/ quantity - Salivary anabasine was 0.5 ng/mL | | | MD **0.9 ng/mL lower** (1.54 lower to 0.34 higher) | Small but important benefit | Small but important benefit | Little to no difference | Point estimate and lower CI are judged as a small benefit as it could indicate an important change in tobacco smoking (see below), and taking into account few potential harms. Upper CI is judged as little to no difference.  Anabasine is an alkaloid with a very similar chemical structure to nicotine; it is found in tobacco but not NRT or e-cigarettes. Anabasine can be used to confirm if a participant using NRT/e-cigarettes had also obtained nicotine from tobacco.  Study does not provide any useful cut-off values, or interpretation of the findings. Indirect evidence suggests that 1ng/mL can be used as a cut-off to indicate not smoking tobacco: <https://trialsjournal.biomedcentral.com/articles/10.1186/s13063-019-3850-1#Sec5>  Baseline rates in this study were in the range of 1.2ng/mL so this difference could be important as it potentially could take someone from smokers to non-smoker levels. | ⨁◯◯◯  VERY LOW |
|  | *Versus usual care, participants had periodontitis*  Smoking reduction (eCO)^a^  (fu: 6 mo)  58 (1 RCT) | 8/10 (critical) | 8.8/10 (critical) | **-** | The mean reduction from baseline in tobacco smoking frequency/quantity - eCO levels was 5.8 ppm | | | MD **6.2 ppm lower** (12.21 lower to 0.19 lower) | Small but important benefit | Small but important benefit | Little to no difference | Considering small likelihood of adverse events, and harms associated with smoking, point estimate and lower CI were judged as small benefit given that the difference could correspond to a reduction of about 10+ cigarettes per day (although correlation is moderate between CO and cigarettes per day), see:  https://ajph.aphapublications.org/doi/pdf/10.2105/AJPH.67.6.545  Judgement for upper CI suggests little to no difference. | ⨁◯◯◯  VERY LOW |
| 3 | *Versus usual care, participants had periodontitis*  Quality of life (UK Oral Health-Related QoL)^a^  (fu: 6 mo)  58 (1 RCT) | 8/10 (critical) | 5.5/10 (important) | **-** | The mean change from baseline quality of life was 8.2 points higher | | | MD **1.4 points higher** (5.9 lower to 8.7 higher) | Little to no difference | Little to no difference | Little to no difference | Based on the total possible score of 80 on this scale, and given the control group change, the point estimate and CI are judged as little to no difference. | ⨁◯◯◯  VERY LOW |
|  | *Versus no intervention*  Quality of life: Change in self-reported health (Assessed with: final item of the Italian version of the EuroQol EQ-D5L; fu: 4 years)  1355 (1 cohort) | 8/10 (critical) | 5.5/10 (important) | - | The mean change was **0.1** | | | MD **0.2 lower**  (0.49 lower to 0.09 higher) | Little to no difference | Little to no difference | Little to no difference | <https://euroqol.org/eq-5d-instruments/eq-5d-5l-about/>  Note that each item on the EuroQol EQ-5D-5L scale is scored from 1 to 5, indicating no problems, slight problems, moderate problems, severe problems and extreme problems, respectively.  Based on the total possible score of 5 for the final item on the scale, the point estimate and CI are judged as little to no difference. | unable to assess |
| 5 | *Versus no intervention*  Adverse events^b^ (fu: 12-16 wks)  Up to 1808 (2 RCT) | 7/10 (important) | 6.5/10 (important) | As some adverse events may be device-specific and type of vaping liquid may affect type of adverse events, a quantitative analysis between studies has not been performed.  Both studies. Five serious adverse events in e-cigarette group of one study (n=1740); no events occurred in other study (n=68). A higher frequency of adverse events reported with e-cigarettes in both studies.  One study (n=1740). AEs were analyzed in different ways with a potential for double counting participants experiencing multiple events. The proportion of mild (29.6% vs 28.4%), moderate (54.6% vs 57.3%), and severe (15.8% vs 14.2%) adverse events were similar between groups. Authors also report varying degrees of attribution of adverse events to the e-cigarette intervention, with 50% accounting for events that are possibly related to the intervention, while 28% were unrelated; all events experienced in the no intervention group were considered not related to allocation. Only two events in the e-cigarette group led to withdrawal (none in no intervention group). | | | | | Little to no difference | Unable to assess | Unable to assess | Insufficient data from review to determine imprecision. Data suggests slightly more AEs among e-cigarette users and may represent a small harm but is difficult to interpret and judged as little to no difference due to potential smoking cessation benefits. | ⨁◯◯◯  VERY LOW |
|  | *Versus no intervention*  Adverse events (all cause mortality)^b^  (fu: 12-16 wks)  68 (1 RCT) | 7/10 (important) | 6.5/10 (important) | No mortality events occurred in either group. | | | | | Little to no difference | Unable to assess | Unable to assess | Insufficient data from review to determine imprecision. Data suggests little to no difference between groups but is difficult to interpret. | unable to assess |
|  | *Versus waitlist*  Adverse events – Complaints ^c^  (fu: 1-8 wks)  NR (1 RCT) | 7/10 (important) | 6.5/10 (important) | Approximately 2 mean complaints in either e-cigarette group and about 3 mean complaints in the waitlist group, as measured in weekly or biweekly interval across the 8-week period.  Small sample size in each group (n=16 in each), and no clear details on which types of complaints were reported for the e-cigarette and waitlist groups. | | | | | Little to no difference | Unable to assess | Unable to assess | Insufficient data from review to determine imprecision. Data suggests little to no difference between groups but is difficult to interpret. | ⨁◯◯◯  VERY LOW |
|  | *Versus usual care, participants had periodontitis*  Adverse events^a^ (fu: 6 mo)  NR (1 RCT) | 7/10 (important) | 6.5/10 (important) | No serious adverse events in groups.  E-cigarettes vs usual care: Various dental adverse events reported, 20 (range 2 to 11) vs 35 total events (range 0 to 5) for: toothache, dentine hypersensitivity, dental/periodontal abscess, tooth loss, mouth ulceration, soreness of soft tissues, fractured or caries filling or tooth, and other miscellaneous events (not reported). A patient may have reported more than one AE. | | | | | Little to no difference | Unable to assess | Unable to assess | Insufficient data from review to determine imprecision. Data suggests little to no difference between groups but is difficult to interpret. | ⨁◯◯◯  VERY LOW |
|  | *Versus no intervention*  Adverse events (fu: 4 years)  1355 (1 cohort) | 7/10 (important) | 6.5/10 (important) | Study authors reported that “no serious adverse events and no higher risk were reported for e-cig when compared to traditional tobacco smoking.” | | | | | Little to no difference | Unable to assess | Unable to assess | Insufficient data from review to determine imprecision. Data suggests little to no difference between groups. | unable to assess |
| 6 | *Versus no intervention*  Weight gain^d^  (fu: 12 wks)  408 (1 RCT) | 6.1/10 (important) | 5/10 (important) | - | | The mean change in body weight was **0.1** kg | MD **0.2 kg higher** (3.53 lower to 3.93 higher) | | Little to no difference | Little to no difference | Little to no difference | Point estimate suggests little to no difference between groups. Upper and lower CI suggest small changes (plus or minus 3.5-4kg of weight gained) judged as trivial amounts of weight change (little to no difference). | ⨁◯◯◯  VERY LOW |
| 7 | *Versus usual care, participants had periodontitis*  Change in mental state (Mood and Physical Symptoms Scale)^a^  (fu: 6 mo)  58 (1 RCT) | 8/10 (important) | 5.5/10 (important) | **-** | | The mean change from baseline in emotional state was 2.8 points lower | MD **0 points**  (4.4 lower to 4.4 higher) | | Little to no difference | Little to no difference | Little to no difference | Higher score represents worse symptoms; scale from 5-35.  Point estimate suggests no difference between groups. Upper and lower CI suggest little to no difference given the baseline change and SD on values at baseline for both groups.  Baseline mean and SD for control and intervention groups were:  MPSS, mean (SD)  Control: 22.8 (7.0)  Intervention: 22.8 (5.9) | ⨁◯◯◯  VERY LOW |

^a^ **Intervention:** E-cigarette starter kit (2 weeks supply of liquid) with the choice of nicotine dosage (0, 6, 12, or 18 mg/mL) and flavour. No participants selected the 0 mg/mL dose. The starter kit included a 2-week supply of liquid. Follow-up was at six months at which point participants may have changed dosages of the cartridge or changed use of e-cigarettes. No co-interventions. **Usual care (provided to both arms):** smoking cessation advice, a referral to stop smoking services was available, and standard non-surgical periodontal therapy.

^b^ **Intervention:** E-cigarette nicotine concentrations varied from 2.7 mg/capsule to 16-24 mg/mL for study duration. No co-interventions mentioned in either study.

^c^ **Intervention:** the two e-cigarette groups evaluated different brand names but at 18 mg/mL nicotine dosing each and were used ad libitum with or without cigarettes. **Control:** The waitlist control were instructed to continue smoking. **Both arms**: No co-interventions reported.

^d^ **Intervention:** E-cigarette nicotine concentration 2.7 mg/capsule. No co-interventions.

| E-cigarettes with nicotine plus standard care^a^ versus E-cigarettes without nicotine plus standard care in smokers willing to quit | | | | | | | | | | | |
| --- | --- | --- | --- | --- | --- | --- | --- | --- | --- | --- | --- |
| **Outcome(s)**  **(Follow up (fu): time)**  **# of participants (# studies)** | | **Median Patient rating** | **Mean WG rating** | **Relative effect (95%CI)** | **Baseline (control) risk** | **Absolute difference with intervention** | **Assessment of absolute difference** | | | | **Certainty** |
|  |  |  |  |  |  |  | **Point estimate** | **Lower 95% CI** | **Upper 95%CI** | **Comments on judgment** |  |
| 1 | Smoking cessation (7-day point prevalence confirmed by exCO ≤6ppm measured; fu: 24 wks)  40 (1 RCT) | 8/10 (critical) | 8.8/10 (critical) | RR 2.00 (0.41 to 9.71) | 100 per 1,000 | **100 more per 1,000** (from 59 fewer to 871 more) | Large benefit | Moderate harm | Large benefit | Point estimate and upper CI represent large benefits given baseline risk and health benefits of quitting. Lower CI results in fewer individuals quitting smoking and is therefore judged as a moderate harm due to harms of continuing to smoke. Few harms were identified for this comparison and population. | ⨁◯◯◯  VERY LOW |
| 2 | Smoking reduction (cigarettes per day)  (fu: 24 wks)  40 (1 RCT) | 7/10 (critical) | 5.0/10 (critical) | **-** | The mean reduction in tobacco smoking frequency/quantity was 8.04 cigarettes per day | Smaller reduction by MD **2.54** (4.62 greater reduction to 9.7 smaller reduction) | Little to no difference | Little to no difference | Little to no difference | Baseline level of smoking is unknown, but a smaller reduction by 2.54 cigarettes per day judged as little to no difference given harms of continuing to smoke. Upper and lower CI could be considered small but important harm or benefit, respectively (from ~13 cigarettes per day less to ~2 more per day), which is partly dependent on baseline level of smoking. However reduction in smoking [still causes significantly increased morbidity and mortality](https://www.ncbi.nlm.nih.gov/pmc/articles/PMC2865193/), as opposed to quitting, so reduction is considered little to no difference (trivial benefit/harm). | ⨁◯◯◯  VERY LOW |
| 5 | Adverse events  (fu: 24 wks)  40 (1 RCT) | 7/10 (important) | 6.5/10 (important) | E-cigarette with nicotine vs e-cigarette with no nicotine: Various side effects reported, 28 (range 0 to 7) vs 21 total events (range 0 to 5) for: abnormal dreams, anxiety, fatigue, headache, insomnia, nausea, palpitations, pruritus, cough, shortness of breath, sore throat, and increased appetite. A participant may have reported more than one AE. Small sample sizes (n=20) in each group. | | | Little to no difference | Unable to assess | Unable to assess | Harms identified appear to be minor and only slightly more common among e-cigarette users. Considering potential offset of smoking cessation, these are judged as trivial. If the true effect on smoking cessation was a harm (i.e., lower CI for smoking cessation outcome) these harms may be considered small but important, suggesting uncertainty. | ⨁◯◯◯  VERY LOW |

^a^ **Intervention and control:** Both groups received the same brand of e-cigarette, with one group receiving cartridge with 24 mg/mL of nicotine and the other group receiving cartridge with 0 mg/mL of nicotine. Both groups also received standard care which consisted of nicotine patch and counselling sessions. No co-interventions.

| E-cigarettes with nicotine^a^ versus E-cigarettes without nicotine (placebo) in smokers NOT motivated/wishing to quit | | | | | | | | | | | |
| --- | --- | --- | --- | --- | --- | --- | --- | --- | --- | --- | --- |
| **Outcome(s)**  **(Follow up (fu): time)**  **# of participants (# studies)** | | **Median Patient rating** | **WG rating** | **Relative effect (95%CI)** | **Baseline (control) risk** | **Absolute difference with intervention** | **Assessment of absolute difference** | | | | **Certainty** |
|  |  |  |  |  |  |  | **Point estimate** | **Lower 95% CI** | **Upper 95%CI** | **Comments on judgment** |  |
| 1 | Smoking cessation (fu: 12 mo)  300 (1 RCT) | 8/10 (critical) | 8.8/10 (critical) | RR 2.76  (0.97 to 7.76) | 40 per 1,000 | **70 more per 1,000** (from 1 fewer to 270 more) | Moderate benefit | Little to no difference | Large benefit | Point estimate represents moderate benefit given baseline risk, lack of identified harms of intervention, and health benefits of quitting. Upper CI judged as a large benefit for the same reasons. Lower CI is judged as little to no difference (1 less person per 1,000 quitting with intervention). Little to no difference in adverse events identified in this population, and in those motivated to quit for a similar comparison (nicotine versus no nicotine e-cigs). | ⨁⨁◯◯  LOW |
| 2 | Smoking reduction (>50% of baseline or cessation)  (fu: 12 mo)  300 (1 RCT) | 7/10 (critical) | 5.0/10 (critical) | RR 1.28 (0.76 to 2.17) | 160 per 1,000 | **45 more per 1,000** (from 38 fewer to 187 more) | Small but important harm | Small but important harm | Moderate benefit | Point estimate and lower CI suggest small benefit and harm, respectively, given the reduction in smoking [still causes significantly increased morbidity and mortality](https://www.ncbi.nlm.nih.gov/pmc/articles/PMC2865193/), as opposed to quitting (analysis does not include quitters). Upper CI is a moderate benefit given baseline risk and fact that patients are still continuing to smoke, and given little data on adverse events. | ⨁◯◯◯  VERY LOW |
|  | Smoking reduction (# of cigarettes per day)  (fu: 12 mo)  300 (1 RCT) | 7/10 (critical) | 5.0/10 (critical) | No statistically significant difference in number of cigarettes/day between groups. Median CPD = 12 - 14 in all groups. | | | Little to no difference | Unable to asses | Unable to asses | Insufficient data from review to determine imprecision. Data suggests little to no difference between groups but is difficult to interpret as it only provides end-point data, not difference from baseline. | unable to assess |
|  | Smoking reduction (CO)  (fu: 12 mo)  300 (1 RCT) | 7/10 (critical) | 5.0/10 (critical) | No significant difference in CO between groups. Median CO = 15-17 ppm in all groups. | | | Little to no difference | Unable to asses | Unable to asses | Insufficient data from review to determine imprecision. Difficult to assess due to lack of data on change from baseline (only have baseline and end of follow-up for each group, not difference between groups). However, baseline values were similar, as were follow-up values, suggesting little to no difference.  Results, excluding quitters, from results table:  E-cig group 1  Baseline: 19.0 ppm, 52-weeks: 15.0 ppm (8.8 to 29.0); n=52  Ecig group 2  Baseline: 22.0 ppm, 52-weeks: 18.0 ppm (10.0 to 26.0); n=54  Control:  Baseline: 19.5 ppm, 52-weeks: 19.0 ppm (13.0 to 28.0); n=51 | unable to assess |
| 5 | Adverse events  (fu: baseline, 3 mo, 12 mo)  NR (1 RCT) | 7/10 (important) | 6.5/10 (important) | Similar frequency of AEs across groups at all time points (baseline, week 12, week 52). Reduction in all symptoms from baseline to 12-month follow-up across groups (P < 0.001). Rates of shortness of breath were reduced from 20% to 4% from baseline to 2 weeks. | | | Little to no difference | Unable to assess | Unable to assess | Insufficient data from review to determine imprecision. Data suggests little to no difference between groups but is difficult to interpret. | unable to assess |
|  | Serious adverse events  (fu: 12 mo)  NR (1 RCT) | 7/10 (important) | 6.5/10 (important) | No serious adverse events occurred. | | | Little to no difference | Unable to assess | Unable to assess | Insufficient data from review to determine imprecision. Data suggests little to no difference between groups. | unable to assess |
| 6 | Weight gain (fu: NR)  NR (1 RCT) | 6.1/10 (important) | 5/10 (important) | No significant difference in weight change within or between groups. Uncertain but unlikely that the outcome is post-cessation weight gain (i.e., does not appear to be assessed in abstinent smokers only). | | | Little to no difference | Unable to assess | Unable to assess | Insufficient data from review to determine imprecision. Data suggests little to no difference between groups but is difficult to interpret. | unable to assess |

^a^ **Intervention and control:** All groups received the same Categoria e-cig (model 401), with difference in dosage of nicotine (i.e., 7.2 mg/mL for 12 weeks, 7.2 mg/mL for six weeks followed by 5.4 mg/mL for six weeks, no nicotine for 12 weeks). No co-interventions.

| E-cigarettes with nicotine^a^ versus E-cigarettes without nicotine in smokers NOT willing to quit | | | | | | | | | | | |
| --- | --- | --- | --- | --- | --- | --- | --- | --- | --- | --- | --- |
| **Outcome(s)**  **(Follow up (fu): time)**  **# of participants (# studies)** | | **Median Patient rating** | **Mean WG rating** | **Relative effect (95%CI)** | **Baseline (control) risk** | **Absolute difference with intervention** | **Assessment of absolute difference** | | | | **Certainty** |
|  |  |  |  |  |  |  | **Point estimate** | **Lower 95% CI** | **Upper 95%CI** | **Comments on judgment** |  |
| 1 | Smoking cessation (fu: 24 wks)  300 (1 RCT) | 8/10 (critical) | 8.8/10 (critical) | RR 2.20 (0.86 to 5.64) | 50 per 1,000 | **60 more per 1,000** (from 7 fewer to 232 more) | Moderate benefit | Small but important harm | Large benefit | Point estimate represents moderate benefits given baseline risk, lack of identified harms of intervention, and health benefits of quitting. Upper CI judged as a large benefit for the same reasons. Lower CI suggests small harm due to harms of continuing to smoke. Little to no difference in adverse events identified in this population, and in those motivated to quit for a similar comparison (nicotine versus no nicotine e-cigs). | ⨁◯◯◯  VERY LOW |
|  | Smoking cessation (fu: 52 wks)  300 (1 RCT) | 8/10 (critical) | 8.8/10 (critical) | RR 2.75 (0.97 to 7.76) | 40 per 1,000 | **70 more per 1,000** (from 1 fewer to 270 more) | Moderate benefit | Little to no difference | Large benefit | Point estimate represents moderate benefits given baseline risk, lack of identified harms of intervention, and health benefits of quitting. Upper CI judged as a large benefit for the same reasons. Lower CI is judged as little to no difference (1 less person per 1,000 quitting with intervention). Little to no difference adverse events identified in this population, and in those motivated to quit for a similar comparison (nicotine versus no nicotine e-cigs). | ⨁◯◯◯  VERY LOW |
| 2 | Smoking reduction (reduction in cigarettes per day of >50% since baseline)  (fu: 24 wks)  300 (1 RCT) | 7/10 (critical) | 5.0/10 (critical) | RR 1.20 (0.69 to 2.08) | 150 per 1,000 | **30 more per 1,000** (from 47 fewer to 162 more) | Small but important benefit | Small but important harm | Moderate benefit | Point estimate suggests small benefit given that those reducing by 50% are still continuing to smoke (reduction in smoking [still causes significantly increased morbidity and mortality](https://www.ncbi.nlm.nih.gov/pmc/articles/PMC2865193/), as opposed to quitting) and analysis may include quitters, but considering more challenging population (i.e., not willing/motivated to quit), and little to no difference in adverse events. Upper CI is judged as a moderate benefit for the same reasons. Lower CI is a small harm given fewer patients would be reducing their smoking. | ⨁◯◯◯  VERY LOW |
|  | Smoking reduction (reduction in cigarettes per day of >50% since baseline)  (fu: 52 wks)  300 (1 RCT) | 7/10 (critical) | 5.0/10 (critical) | RR 0.79 (0.40 to 1.57) | 120 per 1,000 | **25 fewer per 1,000** (from 72 fewer to 68 more) | Small but important harm | Moderate harm | Small but important benefit | Point estimate and lower CI suggest small and moderate harms, respectively, given that fewer patients are reducing their smoking and the harms associated with smoking (analysis does not include quitters). Upper CI is a small benefit given baseline risk and fact that patients are still continuing to smoke, and given little data on adverse events. | ⨁◯◯◯  VERY LOW |
|  | Smoking reduction (assessed by eCO)  (fu: 52 wks)  NR (1 RCT) | 7/10 (critical) | 5.0/10 (critical) | Among all participants in the per protocol analysis there was no statistically significant difference between groups in eCO levels at 52 weeks (Group A&B: pooled mean 17.55 ppm, Group C: 17.77 ppm). There was a high number of missing participants (35/100 in group A, 37/100 in group B, and 45/100 in group C). After excluding quitters, there was no statistically significant difference between groups (Group A&B: 17.8 ppm, Group C: 20 ppm). | | | Little to no difference | Unable to assess | Unable to assess | Insufficient data from review to determine imprecision. Difficult to assess due to lack of data on change from baseline (only have baseline and end of follow-up for each group, not difference between groups). However, baseline values were similar, as were follow-up values, suggesting little to no difference.  Results, excluding quitters, from results table:  E-cig group 1  Baseline: 19.0 ppm, 52-weeks: 15.0 ppm (8.8 to 29.0); n=52  Ecig group 2  Baseline: 22.0 ppm, 52-weeks: 18.0 ppm (10.0 to 26.0); n=54  Control:  Baseline: 19.5 ppm, 52-weeks: 19.0 ppm (13.0 to 28.0); n=51 | unable to assess |
| 5 | Adverse events (fu: 52 wks)  NR (1 RCT) | 7/10 (important) | 6.5/10 (important) | There were no serious AEs reported. Various side effects reported, ranging from 2 to 6% in the groups who were given nicotine and 2 to 7% in the no nicotine group for: hunger, insomnia, irritability, anxiety, and depression. AEs were reported as the most frequently reported before using e-cigarettes and noted in the AE page of the study diary. AEs reported between weeks 12 and 52 remained fairly stable within groups with some exceptions (e.g., shortness of breath increased in all groups). There were small sample sizes in each group (n=100). | | | Little to no difference | Unable to assess | Unable to assess | Insufficient data from review to determine imprecision. Data suggests little to no difference between groups but is difficult to interpret. | unable to assess |
| 6 | Weight gain (change from baseline)  (fu: 52 wks)  NR (1 RCT) | 6.1/10 (important) | 5/10 (important) | The study authors present the data as a percent of baseline weight, and have made the data unit-less as they have set the baseline as 100%. As we do not have individual patient data, and a percent change would depend on starting weight, no formal analysis has been done on this outcome. There was some fluctuation in weight gain from baseline to weeks 12, 24 and 52, ranging from 0.93 to 0.99% from baseline weight in Group A, 0.62 to 1.64% of baseline weight in Group B, and 0.28 to 0.76% of baseline weight in Group C. However, depending on the actual baseline weight in pounds/kg, these percentages may vary in actual weight gain. | | | Little to no difference | Unable to assess | Unable to assess | Insufficient data from review to determine imprecision. Data suggests little to no difference between groups but is difficult to interpret. | ⨁◯◯◯  VERY LOW |

^a^ **Intervention and control:** All groups received the same Categoria e-cig (model 401), with difference in dosage of nicotine (i.e., 7.2 mg/mL for 12 weeks, 7.2 mg/mL for six weeks followed by 5.4 mg/mL for six weeks, no nicotine for 12 weeks). No co-interventions.

| E-cigarette with nicotine + other smoking cessation treatment (behavioural support) vs E-cigarette with no nicotine + other smoking cessation treatment (behavioural support) in smokers willing to quit | | | | | | | | | | | |
| --- | --- | --- | --- | --- | --- | --- | --- | --- | --- | --- | --- |
| **Outcome(s)**  **(Follow up (fu): time)**  **# of participants (# studies)** | | **Median Patient rating** | **Mean WG rating** | **Relative effect (95%CI)** | **Baseline (control) risk** | **Absolute difference with intervention** | **Assessment of absolute difference** | | | | **Certainty** |
|  |  |  |  |  |  |  | **Point estimate** | **Lower 95% CI** | **Upper 95%CI** | **Comments on judgment** |  |
| 1 | Smoking cessation (continuous abstinence, ≤5 cigarettes total allowed, eCO <10ppm)  (fu: 6 months)  657 (1 RCT) | 8/10 (critical) | 8.8/10 (critical) | RR 1.77  (0.54 to 5.77) | 41 per 1,000 | **32 more per 1,000**  (from 19 fewer to 196 more) | Small but important benefit | Small but important harm | Large benefit | Point estimate represents a small but important benefit given baseline risk and health benefits of quitting. Upper CI judged as a large benefit for the same reasons. Lower CI suggests small harm due to harms of continuing to smoke. | ⨁⨁⨁◯  MODERATE |
|  | Smoking cessation (7-day point prevalence abstinence: self-reported no smoking of cigarettes in past 7 days)  (fu: 6 months)  657 (1 RCT) | 8/10 (critical) | 8.8/10 (critical) | RR 0.96  (0.59 to 1.57) | 219 per 1,000 | **9 fewer per 1,000**  (from 90 fewer to 125 more) | Small but important harm | Large harm | Large benefit | Point estimate is judged as a small but important harm given that fewer patients would quit smoking, and given the harms associated with continuing to smoke. Lower CI is judged as a large harm based on the same rationale. The upper CI is judged as a large benefit given the health benefits of quitting. | ⨁⨁⨁◯  MODERATE |
|  | Smoking cessation  (continuous abstinence, eCO ≤7ppm)  (fu: 6 months)  140 (1 RCT) | 8/10 (critical) | 8.8/10 (critical) | RR 1.18  (0.57 to 2.46) | 157 per 1,000 | **28 more per 1,000**  (from 68 fewer to 229 more) | Small but important benefit | Moderate harm | Large benefit | Point estimate represents a small but important benefit given baseline risk and health benefits of quitting. Upper CI judged as a large benefit for the same reasons. Lower CI judged as moderate harm due to harms of continuing to smoke. | ⨁◯◯◯  VERY LOW |
| 2 | Smoking reduction (Proportion of participants who reduced daily cigarettes by 50% or greater)  (fu: 6 months)  657 (1 RCT) | 7/10  (critical) | 5.0/10  (important) | RR 1.26  (0.96 to 1.66) | 452 per 1,000 | **118 more per 1,000**  (from 18 fewer to 298 more) | Moderate benefit | Small but important harm | Moderate benefit | Point estimate suggests moderate benefit given that those reducing by 50% are still continuing to smoke ([still significantly increased morbidity and mortality](https://www.ncbi.nlm.nih.gov/pmc/articles/PMC2865193/), unless this leads to quitting, which could potentially follow). Upper CI suggest a moderate benefit based on the same rationale. Lower CI suggests small harm (2% less individuals reducing their smoking) given harms of continuing to smoke at greater levels. | ⨁⨁⨁◯  MODERATE |
|  | Smoking reduction (Change in mean number of daily cigarettes smoked since baseline)  (fu: 24 weeks)  249 (1 RCT) | 7/10  (critical) | 5.0/10  (important) | - | At 24 weeks, the intervention group had a -10.7 change in mean number of daily cigarettes smoked since baseline and the control group had -9.1. SD were not reported. | | Little to no difference | Unable to assess | Unable to assess | Insufficient data from review to determine imprecision. Data suggests little to no difference between groups. | ⨁◯◯◯  VERY LOW |
|  | Smoking reduction (Number of daily cigarettes smoked) (fu: 6 months)  140 (1 RCT) | 7/10  (critical) | 5.0/10  (important) | - | The mean number of daily cigarettes smoked was 14.03 (SD 7.92) | MD 3.02 lower  (5.42 lower to 0.62 lower) | Little to no difference | Little to no difference | Little to no difference | A reduction by 3.02 cigarettes per day judged as little to no difference given harms of continuing to smoke. The lower CI could be considered small but important benefit, which is partly dependent on baseline level of smoking. However reduction in smoking [still causes significantly increased morbidity and mortality](https://www.ncbi.nlm.nih.gov/pmc/articles/PMC2865193/), as opposed to quitting, so reduction is considered little to no difference (trivial benefit/harm). | ⨁◯◯◯  VERY LOW |
|  | Smoking reduction (eCO levels)  (fu: 6 months)  140 (1 RCT) | 7/10  (critical) | 5.0/10  (important) | - | The mean change in eCO levels was 15.28 (SD 11.43) | MD 3.27 higher  (6.56 lower to 0.02 higher) | Little to no difference | Small but important benefit | Little to no difference | Point estimate and upper CI are judged as little to no difference. Lower CI was judged as a small benefit given that the difference could correspond to a reduction of about 10 cigarettes per day (although correlation is moderate between CO and cigarettes per day), see:  <https://ajph.aphapublications.org/doi/pdf/10.2105/AJPH.67.6.545>. Note that [little impact on health would be anticipated unless this reduction ultimately leads to quitting](https://www.ncbi.nlm.nih.gov/pmc/articles/PMC2865193/). | ⨁◯◯◯  VERY LOW |
| 5 | Serious adverse events (fu: 12 weeks)  249 (1 RCT) | 7/10 (important) | 6.5/10 (important) | Serious adverse events were adjudicated by an end points evaluation committee and include death, respiratory, cardiovascular, neuropsychiatric or other events. At 12 weeks, the intervention group had experienced 1 (0.8%) and the control group had experienced 4 (3.1%). | | | Little to no difference | Unable to assess | Unable to assess | Insufficient data to determine imprecision. Data suggests little to no difference between groups. | ⨁◯◯◯  VERY LOW |
|  | Serious adverse events (fu: 12 to 24 weeks)  249 (1 RCT) | 7/10 (important) | 6.5/10 (important) | Serious adverse events were adjudicated by an end points evaluation committee and include death, respiratory, cardiovascular, neuropsychiatric or other events. At 12 to 24 weeks, the intervention group had experienced 2 (1.6%) and the control group had experienced 2 (1.6%). | | | Little to no difference | Unable to assess | Unable to assess | Insufficient data to determine imprecision. Data suggests little to no difference between groups. | ⨁◯◯◯  VERY LOW |
|  | Serious adverse events (fu: 6 months)  657 (1 RCT) | 7/10 (important) | 6.5/10 (important) | Serious adverse events included death, life threatening illness, admission to hospital or prolongation of hospital stay persistent or significant disability or incapacity, congenital abnormality, or other medically important events. At 6 months, the intervention group had experienced 27 (20%) and the control group had experienced 5 (14%). | | | Little to no difference | Unable to assess | Unable to assess | Insufficient data to determine imprecision. Considering severity of outcome, difference could be considered little to no difference or a potentially small important effect (20% in intervention group versus 14% in control group). However, study authors note that none of the serious adverse events in any group were related to product use. As such we judge the results to represent little to no difference in serious adverse effects over the follow-up period. | ⨁⨁⨁◯  MODERATE |
|  | Mild adverse events (fu: 12 weeks)  249 (1 RCT) | 7/10 (important) | 6.5/10 (important) | Mild adverse events included cough, dry mouth, headache, rhinitis, throat irritation, dyspnea, sore throat, light headedness, dizziness, mouth irritation, nausea, indigestion, mouth ulcers, or vertigo. Only the first event for each participant in each category was counted. At 12 weeks, the intervention group had experienced 120 (94%) and the control group had experienced 118 (93%). | | | Little to no difference | Unable to assess | Unable to assess | Insufficient data to determine imprecision. Data suggest little to no difference between groups but is difficult to interpret. Possible increase in relatively mild harms that could be important to patients, but still considered trivial given potential benefit on smoking cessation. | ⨁◯◯◯  VERY LOW |
|  | Adverse events likely to be related to e-cig use (fu: 3 & 6 months)  140 (1 RCT) | 7/10 (important) | 6.5/10 (important) | At 3 months, 5.7% of the intervention group had experienced side effects (10% burning throat, 1.4% cough, 1.4% headache, 1.4% stomach-ache) and 2.9% in the control group (2.9% burning throat).  At 6 months, 15.9% of the intervention group had experienced side effects (5.8% burning throat, 5.8% cough, 1.4% headache, 4.3% insomnia, 1.4% stomach-ache) and 5.6% in the control group (2.8% burning throat, 7% cough, 1.4% headache, 4.2% insomnia). | | | Small but important harm | Unable to assess | Unable to assess | Insufficient data to determine imprecision. Data suggest small but important harm given higher rates of side effects likely related to e-cig use in the intervention group. However, harms are considered relatively mild given potential benefit on smoking cessation. | ⨁◯◯◯  VERY LOW |
|  | Non-serious adverse events (fu: 6 months)  657 (1 RCT) | 7/10 (important) | 6.5/10 (important) | At 6 months, the intervention group had experienced 110 (80%) and the control group had experienced 31 (86%). | | | Little to no difference | Unable to assess | Unable to assess | Insufficient data to determine imprecision. Data suggests little to no difference between groups. | ⨁⨁⨁◯  MODERATE |

| E-cigarette with nicotine + other smoking cessation treatment (behavioural support) vs No intervention + other smoking cessation treatment (behavioural support) in smokers willing to quit | | | | | | | | | | | |
| --- | --- | --- | --- | --- | --- | --- | --- | --- | --- | --- | --- |
| **Outcome(s)**  **(Follow up (fu): time)**  **# of participants (# studies)** | | **Median Patient rating** | **Mean WG rating** | **Relative effect (95%CI)** | **Baseline (control) risk** | **Absolute difference with intervention** | **Assessment of absolute difference** | | | | **Certainty** |
|  |  |  |  |  |  |  | **Point estimate** | **Lower 95% CI** | **Upper 95%CI** | **Comments on judgment** |  |
| 1 | Smoking cessation (fu: 6 months)  140 (1 RCT) | 8/10 (critical) | 8.8/10 (critical) | **RR 1.86**  (0.79 to 4.38) | 100 per 1,000 | **86 more per 1,000**  (from 21 fewer to 338 more) | Moderate benefit | Small but important harm | Large benefit | Increase in quit rate moderate when considering health benefits of quitting and baseline risk. Upper CI suggests large benefit based on same rationale. Lower CI suggests small but important harm due to reduction in quit rate. Data on adverse events in this population suggests trivial (little to no difference) serious harms and small but important mild adverse events. | ⨁◯◯◯  VERY LOW |
| 2 | Smoking reduction  (Change in mean number of daily cigarettes smoked since baseline)  (fu: 24 weeks)  249 (1 RCT) | 7/10  (critical) | 5.0/10  (important) | At 24 weeks, the intervention group had a -10.7 change in mean number of daily cigarettes smoked since baseline and the control group had -5.5. SD were not reported. | | |  |  |  |  | ⨁◯◯◯  VERY LOW |
|  | Smoking reduction  (Number of daily cigarettes smoked)  (fu: 6 months)  140 (1 RCT) | 7/10  (critical) | 5.0/10  (important) | - | The mean number of daily cigarettes smoked was 13.45 (SD 6.49) | **MD 2.44 lower**  (4.59 lower to 0.29 lower) | Little to no difference | Little to no difference | Little to no difference | A reduction by 2.44 cigarettes per day judged as little to no difference given harms of continuing to smoke. The lower CI could be considered small but important benefit, which is partly dependent on baseline level of smoking. However reduction in smoking [still causes significantly increased morbidity and mortality](https://www.ncbi.nlm.nih.gov/pmc/articles/PMC2865193/), as opposed to quitting, so reduction is considered little to no difference (trivial benefit/harm). | ⨁◯◯◯  VERY LOW |
|  | Smoking reduction (eCO levels)  (fu: 6 months)  140 (1 RCT) | 7/10  (critical) | 5.0/10  (important) | - | The mean change in eCO levels was 6.52 (SD 10.24) | **MD 5.49 higher**  (2.43 higher to 8.55 higher) | Small but important harm | Little to no difference | Small but important harm | Point estimate and upper CI are judged as small but important harms given that the difference could correspond to an increase in smoking. Lower CI was judged as little to no difference. | ⨁◯◯◯  VERY LOW |
| 5 | Serious adverse events (fu: 12 weeks)  249 (1 RCT) | 7/10 (important) | 6.5/10 (important) | Serious adverse events were adjudicated by an end points evaluation committee and include death, respiratory, cardiovascular, neuropsychiatric or other events. At 12 weeks, the intervention group had experienced 1 (0.8%) and the control group had experienced 2 (1.7%). | | | Little to no difference | Unable to assess | Unable to assess | Insufficient data to determine imprecision. Data suggests little to no difference between groups. | ⨁◯◯◯  VERY LOW |
|  | Serious adverse events (fu: 12 to 24 weeks)  249 (1 RCT) | 7/10 (important) | 6.5/10 (important) | Serious adverse events were adjudicated by an end points evaluation committee and include death, respiratory, cardiovascular, neuropsychiatric or other events. At 12 to 24 weeks, the intervention group had experienced 2 (1.6%) and the control group had experienced 2 (1.7%). | | | Little to no difference | Unable to assess | Unable to assess | Insufficient data to determine imprecision. Data suggests little to no difference between groups. | ⨁◯◯◯  VERY LOW |
|  | Mild adverse events (fu: 12 weeks)  249 (1 RCT) | 7/10 (important) | 6.5/10 (important) | Mild adverse events included cough, dry mouth, headache, rhinitis, throat irritation, dyspnea, sore throat, light headedness, dizziness, mouth irritation, nausea, indigestion, mouth ulcers, or vertigo. Only the first event for each participant in each category was counted. At 12 weeks, the intervention group had experienced 120 (94%) and the control group had experienced 88 (73%). | | | Small but important harm | Unable to assess | Unable to assess | Insufficient data to determine imprecision. Data suggest small but important harm given higher rates of mild adverse events in the intervention group. However, harms are considered relatively mild given potential benefit on smoking cessation. | ⨁◯◯◯  VERY LOW |

| E-cigarette with nicotine + other smoking cessation treatment (behavioural support) + standard care (nicotine patch) vs. E-cigarette with no nicotine + other smoking cessation treatment (behavioural support) + standard care (nicotine patch) in smokers willing to quit within the next 2 weeks | | | | | | | | | | | |
| --- | --- | --- | --- | --- | --- | --- | --- | --- | --- | --- | --- |
| **Outcome(s)**  **(Follow up (fu): time)**  **# of participants (# studies)** | | **Median Patient rating** | **Mean WG rating** | **Relative effect (95%CI)** | **Baseline (control) risk** | **Absolute difference with intervention** | **Assessment of absolute difference** | | | | **Certainty** |
|  |  |  |  |  |  |  | **Point estimate** | **Lower 95% CI** | **Upper 95%CI** | **Comments on judgment** |  |
| 1 | Smoking cessation  (continuous; self-reported; allowing ≤5 cigarettes in total, eCO verified ≤9ppm)  (fu: 6 months)  999 (1 RCT) | 8/10 (critical) | 8.8/10 (critical) | **RR 1.75**  (1.02 to 2.98) | 40 per 1,000 | **30 more per 1,000**  (from 1 more to 79 more) | Small but important benefit | Little to no difference | Moderate benefit | Increase in quit rate is judged as a small but important benefit when considering health benefits of quitting and baseline risk. Upper CI suggests moderate benefit based on same rationale. Lower CI suggests little to no difference. Data on harms in this population suggests little to no difference in harms, but is hard to interpret for certain outcomes. | ⨁⨁⨁◯  MODERATE |
|  | Smoking cessation  (continuous; self-reported)  (fu: 6 months)  999 (1 RCT) | 8/10 (critical) | 8.8/10 (critical) | **RR 1.68**  (1.22 to 2.30) | 106 per 1,000 | **72 more per 1,000**  (from 23 more to 138 more) | Moderate benefit | Small but important benefit | Large benefit | Increase in quit rate moderate when considering health benefits of quitting and baseline risk. Upper CI suggests large benefit based on same rationale. Lower CI suggests small but important benefit. Data on harms in this population suggests little to no difference in harms, but is hard to interpret for certain outcomes. | ⨁⨁⨁◯  MODERATE |
|  | Smoking cessation  (7-day point prevalence abstinence; self-reported)  (fu: 6 months)  999 (1 RCT) | 8/10 (critical) | 8.8/10 (critical) | **RR 1.43**  (1.11 to 1.84) | 166 per 1,000 | **72 more per 1,000**  (from 18 more to 140 more) | Moderate benefit | Small but important benefit | Large benefit | Increase in quit rate moderate when considering health benefits of quitting and baseline risk. Upper CI suggests large benefit based on same rationale. Lower CI suggests small but important benefit. Data on harms in this population suggests little to no difference in harms, but is hard to interpret for certain outcomes. | ⨁⨁⨁◯  MODERATE |
| 2 | Smoking reduction  (Change in mean number of daily cigarettes smoked since baseline; self-reported)  (fu: 6 months)  999 (1 RCT) | 7/10  (critical) | 5.0/10  (important) | - | The mean number of cigarettes smoked per day was 8.3 (SD 0.4) | **MD 0 lower**  (0.06 lower to 0.06 higher) | Little to no difference | Little to no difference | Little to no difference | Point estimate, lower CI, and upper CI suggest little to no difference. | ⨁⨁⨁◯  MODERATE |
|  | Smoking reduction  (≥50% reduction in the number of cigarettes/day since baseline; self-reported)  (fu: 6 months)  999 (1 RCT) | 7/10  (critical) | 5.0/10  (important) | **RR 1.15**  (0.99 to 1.33) | 399 per 1,000 | **60 more per 1,000**  (from 4 fewer to 132 more) | Small but important benefit | Little to no difference | Moderate benefit | Point estimate suggests small benefit given that those reducing by 50% are still continuing to smoke (reduction in smoking [still causes significantly increased morbidity and mortality](https://www.ncbi.nlm.nih.gov/pmc/articles/PMC2865193/), as opposed to quitting), but considering little to no difference in harms. Upper CI is judged as a moderate benefit for the same reasons. Lower CI suggests little to no difference. | ⨁⨁⨁◯  MODERATE |
| 5 | Adverse events: Participants with a serious adverse event (follow up: 6 months)  999 (1 RCT) | 7/10 (important) | 6.5/10 (important) | **RR 0.73**  (0.39 to 1.37) | 44 per 1,000 | **12 fewer per 1,000**  (from 27 fewer to 16 more) | Little to no difference | Moderate benefit | Small but important harm | Considering the reduction in a serious outcome and smoking cessation benefit, point estimate and lower CI suggest small but important benefit and moderate benefit, respectively. Upper CI suggests small but important harm given the increase in serious adverse event. However, study authors note that none of the serious adverse events in any group were related to product use. As such we judge the results to represent little to no difference in serious adverse effects over the follow-up period. | ⨁⨁◯◯ LOW |
|  | Adverse events: Total serious adverse events (follow up: 6 months)  999 (1 RCT) | 7/10 (important) | 6.5/10 (important) | At 6 months, there was a total of 18 SAEs in the intervention group (11 hospitalizations, 4 otherwise medically important, 2 life-threatening, 1 persistent, significant disability or incapacity) and 27 in the control group (19 hospitalizations, 6 otherwise medically important, 1 life-threatening, 1 death). | | | Unable to assess | Unable to assess | Unable to assess | Insufficient data to determine imprecision. There was a lower absolute number of SAEs in the intervention group compared to the control group, but the rate of SAEs in each group is unclear. | ⨁⨁⨁◯  MODERATE |
|  | Other adverse events (follow up: 6 months)  999 (1 RCT) | 7/10 (important) | 6.5/10 (important) | At 6 months, various possible adverse events were reported for the intervention and control groups:  vivid dreams 12 (4%) vs 11 (3%);  itchiness 12 (4%) vs 10 (3%);  redness, swollen at patch site 10 (3%) vs 11 (3%);  dry mouth or throat 10 (3%) vs 5 (2%);  cough 15 (4%) vs 4 (1%);  nausea 6 (2%) vs 4 (1%);  headache 6 (2%) vs 7 (2%); | | | Little to no difference | Unable to assess | Unable to assess | Increase in adverse events identified considered trivial (little to no difference) when taking into account relative severity (not severe enough to prompt discontinuation) and potential benefits of quitting smoking. Possible increase in cough could be important to patients. However, difficult to interpret due to lack of baseline risk to compare for absolute differences. Insufficient data to determine imprecision. | ⨁⨁⨁◯  MODERATE |
|  | Possible adverse outcome: Change in BMI from baseline (follow up: 6 months)  999 (1 RCT) | 5/10 (important) | 6.0/10 (important) | - | The mean change in BMI from baseline was -0.1 (SD 0.2) | **MD 0.3 lower**  (0.32 lower to 0.28 lower) | Little to no difference | Little to no difference | Little to no difference | Point estimate, lower CI, and upper CI are considered trivial (little to no difference) BMI change and could be due to daily fluctuations. | ⨁⨁⨁◯  MODERATE |
|  | Possible adverse outcome: Change in weight from baseline (follow up: 6 months)  999 (1 RCT) | 5/10 (important) | 6.0/10 (important) | - | The mean change in weight from baseline was -0.4 (SD 0.5) | **MD 0.7 lower**  (0.76 lower to 0.64 lower) | Little to no difference | Little to no difference | Little to no difference | Point estimate, lower CI, and upper CI are considered trivial (little to no difference) weight change and could be due to daily fluctuations. | ⨁⨁⨁◯  MODERATE |

| E-cigarette with nicotine + other smoking cessation treatment (behavioural support) + standard care (nicotine patch) vs. Other smoking cessation treatment (behavioural support) + standard care (nicotine patch) in smokers willing to quit within the next 2 weeks | | | | | | | | | | | |
| --- | --- | --- | --- | --- | --- | --- | --- | --- | --- | --- | --- |
| **Outcome(s)**  **(Follow up (fu): time)**  **# of participants (# studies)** | | **Median Patient rating** | **Mean WG rating** | **Relative effect (95%CI)** | **Baseline (control) risk** | **Absolute difference with intervention** | **Assessment of absolute difference** | | | | **Certainty** |
|  |  |  |  |  |  |  | **Point estimate** | **Lower 95% CI** | **Upper 95%CI** | **Comments on judgment** |  |
| 1 | Smoking cessation  (continuous; self-reported; allowing ≤5 cigarettes in total, eCO verified ≤9ppm)  (fu: 6 months)  625 (1 RCT) | 8/10 (critical) | 8.8/10 (critical) | **RR 2.92**  (0.91 to 9.33) | 24 per 1,000 | **46 more per 1,000**  (from 2 fewer to 200 more) | Moderate benefit | Little to no difference | Large benefit | Increase in quit rate moderate when considering health benefits of quitting and baseline risk. Upper CI suggests large benefit based on same rationale. Lower CI suggests little to no difference. Data on harms in this population suggests little to no difference in harms, but is hard to interpret for certain outcomes. | ⨁⨁◯◯ LOW |
|  | Smoking cessation  (continuous; self-reported)  (fu: 6 months)  625 (1 RCT) | 8/10 (critical) | 8.8/10 (critical) | **RR 2.23**  (1.19 to 4.15) | 80 per 1,000 | **98 more per 1,000**  (from 15 more to 252 more) | Moderate benefit | Small but important benefit | Large benefit | Increase in quit rate moderate when considering health benefits of quitting and baseline risk. Upper CI suggests large benefit based on same rationale. Lower CI suggests small but important benefit. Data on harms in this population suggests little to no difference in harms, but is hard to interpret for certain outcomes. | ⨁⨁◯◯ LOW |
|  | Smoking cessation  (7-day point prevalence abstinence; self-reported)  (fu: 6 months)  625 (1 RCT) | 8/10 (critical) | 8.8/10 (critical) | **RR 2.13**  (1.27 to 3.57) | 112 per 1,000 | **127 more per 1,000**  (from 30 more to 288 more) | Large benefit | Small but important benefit | Large benefit | Increase in quit rate judged as large benefit when considering health benefits of quitting and baseline risk. Upper CI suggests large benefit based on same rationale. Lower CI suggests small but important benefit. Data on harms in this population suggests little to no difference in harms, but is hard to interpret for certain outcomes. | ⨁⨁◯◯ LOW |
| 2 | Smoking reduction  (change in mean number of daily cigarettes smoked since baseline; self-reported)  (fu: 6 months)  625 (1 RCT) | 7/10  (critical) | 5.0/10  (important) | - | The mean number of cigarettes smoked per day was 8.6 (SD 1.0) | **MD 0.3 lower**  (0.48 lower to 0.12 lower) | Little to no difference | Little to no difference | Little to no difference | Point estimate, lower CI, and upper CI suggest little to no difference. | ⨁⨁⨁◯  MODERATE |
|  | Smoking reduction  (≥50% reduction in the number of cigarettes/day since baseline; self-reported)  (fu: 6 months)  625 (1 RCT) | 7/10  (critical) | 5.0/10  (important) | **RR 1.70**  (1.24 to 2.33) | 256 per 1,000 | **179 more per 1,000**  (from 61 more to 340 more) | Moderate benefit | Small but important benefit | Moderate benefit | Point estimate and upper CI suggest moderate benefit given that those reducing by 50% are still continuing to smoke (reduction in smoking [still causes significantly increased morbidity and mortality](https://www.ncbi.nlm.nih.gov/pmc/articles/PMC2865193/), as opposed to quitting), but considering little to no difference in harms. Lower CI is judged as a small benefit for the same reasons. | ⨁⨁◯◯ LOW |
| 5 | Adverse events: Participants with a serious adverse event (follow up: 6 months)  625 (1 RCT) | 7/10 (important) | 6.5/10 (important) | **RR 1.33**  (0.39 to 4.50) | 24 per 1,000 | **8 fewer per 1,000**  (from 15 fewer to 84 more) | Little to no difference | Small but important benefit | Large harm | Considering the reduction in a serious outcome and smoking cessation benefit, point estimate and lower CI suggest small but important benefit. Upper CI suggests large harm given the severity of the outcome and baseline risk. However, study authors note that none of the serious adverse events in any group were related to product use. As such we judge the results to represent little to no difference in serious adverse effects over the follow-up period. | ⨁⨁◯◯ LOW |
|  | Adverse events: Total serious adverse events (follow up: 6 months)  625 (1 RCT) | 7/10 (important) | 6.5/10 (important) | At 6 months, there was a total of 18 SAEs in the intervention group (11 hospitalizations, 4 otherwise medically important, 2 life-threatening, 1 persistent, significant disability or incapacity) and 4 in the control group (3 hospitalizations, 1 otherwise medically important). | | | Unable to assess | Unable to assess | Unable to assess | Insufficient data to determine imprecision. There was a higher absolute number of SAEs in the intervention group compared to the control group, but the rate of SAEs in each group is unclear. | ⨁⨁⨁◯  MODERATE |
|  | Other adverse events (follow up: 6 months)  625 (1 RCT) | 7/10 (important) | 6.5/10 (important) | At 6 months, various possible adverse events were reported for the intervention and control groups:  vivid dreams 12 (4%) vs 6 (10%);  itchiness 12 (4%) vs 2 (3%);  redness, swollen at patch site 10 (3%) vs 5 (8%);  dry mouth or throat 10 (3%) vs 0 (0%);  cough 15 (4%) vs 0 (0%);  nausea 6 (2%) vs 2 (3%);  headache 6 (2%) vs 1 (2%); | | | Little to no difference | Unable to assess | Unable to assess | Increase in adverse events identified considered trivial (little to no difference) when taking into account relative severity (not severe enough to prompt discontinuation) and potential benefits of quitting smoking. Possible increase in cough could be important to patients. However, difficult to interpret due to lack of baseline risk to compare for absolute differences. Insufficient data to determine imprecision. | ⨁⨁⨁◯  MODERATE |
| 6 | Possible adverse outcome: Change in BMI from baseline (follow up: 6 months)  625 (1 RCT) | 5/10 (important) | 6.0/10 (important) | - | The mean change in BMI from baseline was 0.1 (SD 0.4) | **MD 0.5 lower**  (0.57 lower to 0.43 lower) | Little to no difference | Little to no difference | Little to no difference | Point estimate, lower CI, and upper CI are considered trivial (little to no difference) BMI change and could be due to daily fluctuations. | ⨁⨁⨁◯  MODERATE |
|  | Possible adverse outcome: Change in weight from baseline (follow up: 6 months)  625 (1 RCT) | 5/10 (important) | 6.0/10 (important) | - | The mean change in weight from baseline was -0.4 (SD 1.0) | **MD 0.7 lower**  (0.88 lower to 0.52 lower) | Little to no difference | Little to no difference | Little to no difference | Point estimate, lower CI, and upper CI are considered trivial (little to no difference) weight change and could be due to daily fluctuations. | ⨁⨁⨁◯  MODERATE |

| E-cigarette with no nicotine + other smoking cessation treatment (behavioural support) + standard care (nicotine patch) vs. Other smoking cessation treatment (behavioural support) + standard care (nicotine patch) in smokers willing to quit within the next 2 weeks | | | | | | | | | | | |
| --- | --- | --- | --- | --- | --- | --- | --- | --- | --- | --- | --- |
| **Outcome(s)**  **(Follow up (fu): time)**  **# of participants (# studies)** | | **Median Patient rating** | **Mean WG rating** | **Relative effect (95%CI)** | **Baseline (control) risk** | **Absolute difference with intervention** | **Assessment of absolute difference** | | | | **Certainty** |
|  |  |  |  |  |  |  | **Point estimate** | **Lower 95% CI** | **Upper 95%CI** | **Comments on judgment** |  |
| 1 | Smoking cessation  (continuous; self-reported; allowing ≤5 cigarettes in total, eCO verified ≤9ppm)  (fu: 6 months)  624 (1 RCT) | 8/10 (critical) | 8.8/10 (critical) | **RR 1.67**  (0.50 to 5.53) | 24 per 1,000 | **16 more per 1,000**  (from 12 fewer to 109 more) | Small but important benefit | Small but important harm | Large benefit | Increase in quit rate judged as small benefit when considering health benefits of quitting and baseline risk. Upper CI suggests large benefit based on same rationale. However, lower CI suggests small but important harm. Data on harms in this population suggests little to no difference in harms, but is hard to interpret for certain outcomes. | ⨁⨁◯◯ LOW |
|  | Smoking cessation  (continuous; self-reported)  (fu: 6 months)  624 (1 RCT) | 8/10 (critical) | 8.8/10 (critical) | **RR 1.33**  (0.70 to 2.53) | 80 per 1,000 | **26 more per 1,000**  (from 24 fewer to 122 more) | Small but important benefit | Small but important harm | Large benefit | Increase in quit rate judged as small benefit when considering health benefits of quitting and baseline risk. Upper CI suggests large benefit based on same rationale. However, lower CI suggests small but important harm. Data on harms in this population suggests little to no difference in harms, but is hard to interpret for certain outcomes. | ⨁⨁◯◯ LOW |
|  | Smoking cessation  (7-day point prevalence abstinence; self-reported)  (fu: 6 months)  624 (1 RCT) | 8/10 (critical) | 8.8/10 (critical) | **RR 1.49**  (0.87 to 2.53) | 112 per 1,000 | **55 more per 1,000**  (from 15 fewer to 171 more) | Moderate benefit | Small but important harm | Large benefit | Increase in quit rate moderate when considering health benefits of quitting and baseline risk. Upper CI suggests large benefit based on same rationale. Lower CI suggests small but important benefit. Data on harms in this population suggests little to no difference in harms, but is hard to interpret for certain outcomes. | ⨁⨁◯◯ LOW |
| 2 | Smoking reduction  (change in mean number of daily cigarettes smoked since baseline; self-reported)  (fu: 6 months)  624 (1 RCT) | 7/10  (critical) | 5.0/10  (important) | - | The mean number of cigarettes smoked per day was 8.6 (SD 1.0) | **MD 0.3 lower**  (0.48 lower to 0.12 lower) | Little to no difference | Little to no difference | Little to no difference | Point estimate, lower CI, and upper CI suggest little to no difference. | ⨁⨁⨁◯  MODERATE |
|  | Smoking reduction  (≥50% reduction in the number of cigarettes/day since baseline; self-reported)  (fu: 6 months)  624 (1 RCT) | 7/10  (critical) | 5.0/10  (important) | **RR 1.49**  (1.08 to 2.05) | 256 per 1,000 | **125 more per 1,000**  (from 20 more to 269 more) | Moderate benefit | Small but important benefit | Moderate benefit | Point estimate and upper CI suggest moderate benefit given that those reducing by 50% are still continuing to smoke (reduction in smoking [still causes significantly increased morbidity and mortality](https://www.ncbi.nlm.nih.gov/pmc/articles/PMC2865193/), as opposed to quitting), but considering little to no difference in harms. Lower CI is judged as a small benefit for the same reasons. | ⨁⨁◯◯ LOW |
| 5 | Adverse events: Participants with a serious adverse event (follow up: 6 months)  624 (1 RCT) | 7/10 (important) | 6.5/10 (important) | **RR 1.84**  (0.56 to 6.04) | 24 per 1,000 | **20 more per 1,000**  (from 11 fewer to 121 more) | Little to no difference | Small but important benefit | Large harm | Considering severity of the outcome and baseline risk, point estimate could suggest moderate benefit. CI includes small benefit and large harm based on same rationale. However, study authors note that none of the serious adverse events in any group were related to product use. As such we judge the results to represent little to no difference in serious adverse effects over the follow-up period. | ⨁⨁◯◯ LOW |
|  | Adverse events: Total serious adverse events (follow up: 6 months)  624 (1 RCT) | 7/10 (important) | 6.5/10 (important) | At 6 months, there was a total of 27 SAEs in the intervention group (19 hospitalizations, 6 otherwise medically important, 1 life-threatening, 1 death) and 4 in the control group (3 hospitalizations, 1 otherwise medically important). | | | Unable to assess | Unable to assess | Unable to assess | Insufficient data to determine imprecision. There was a higher absolute number of SAEs in the intervention group compared to the control group, but the rate of SAEs in each group is unclear. | ⨁⨁⨁◯  MODERATE |
|  | Other adverse events (follow up: 6 months)  624 (1 RCT) | 7/10 (important) | 6.5/10 (important) | At 6 months, various possible adverse events were reported for the intervention and control groups:  vivid dreams 11 (3%) vs 6 (10%);  itchiness 10 (3%) vs 2 (3%);  redness, swollen at patch site 11 (3%) vs 5 (8%);  dry mouth or throat 5 (2%) vs 0 (0%);  cough 4 (1%) vs 0 (0%);  nausea 4 (1%) vs 2 (3%);  headache 7 (2%) vs 1 (2%); | | | Little to no difference | Unable to assess | Unable to assess | Similar rates of other adverse events between groups. Differences identified are judged as trivial (little to no difference) when taking into account relative severity (not severe enough to prompt discontinuation) and potential benefits of quitting smoking. Possible increase in dry mouth and cough in intervention group could be important to patients. However, difficult to interpret due to lack of baseline risk to compare for absolute differences. Insufficient data to determine imprecision. | ⨁⨁⨁◯  MODERATE |
| 6 | Possible adverse outcome: Change in BMI from baseline (follow up: 6 months)  624 (1 RCT) | 5/10 (important) | 6.0/10 (important) | - | The mean change in BMI from baseline was 0.1 (SD 0.4) | **MD 0.2 lower**  (0.27 lower to 0.13 lower) | Little to no difference | Little to no difference | Little to no difference | Point estimate, lower CI, and upper CI are considered trivial (little to no difference) BMI change and could be due to daily fluctuations. | ⨁⨁⨁◯  MODERATE |
|  | Possible adverse outcome: Change in weight from baseline (follow up: 6 months)  624 (1 RCT) | 5/10 (important) | 6.0/10 (important) | - | The mean change in weight from baseline was -0.4 (SD 1.0) | **MD 0 lower**  (0.18 lower to 0.18 higher) | Little to no difference | Little to no difference | Little to no difference | Point estimate, lower CI, and upper CI are considered trivial (little to no difference) weight change and could be due to daily fluctuations. | ⨁⨁◯◯ LOW |

| E-cigarette with no nicotine + other smoking cessation treatment (behavioural support) vs. Other smoking cessation treatment (behavioural support) in smokers willing to quit | | | | | | | | | | | |
| --- | --- | --- | --- | --- | --- | --- | --- | --- | --- | --- | --- |
| **Outcome(s)**  **(Follow up (fu): time)**  **# of participants (# studies)** | | **Median Patient rating** | **Mean WG rating** | **Relative effect (95%CI)** | **Baseline (control) risk** | **Absolute difference with intervention** | **Assessment of absolute difference** | | | | **Certainty** |
|  |  |  |  |  |  |  | **Point estimate** | **Lower 95% CI** | **Upper 95%CI** | **Comments on judgment** |  |
| 1 | Smoking cessation  (continuous; self-reported; eCO verified ≤7ppm)  (fu: 6 months)  140 (1 RCT) | 8/10 (critical) | 8.8/10 (critical) | **RR 1.57**  **(0.65 to 3.82)** | 100 per 1,000 | **57 more per 1,000**  (from 35 fewer to 282 more) | Moderate benefit | Moderate harm | Large benefit | Increase in quit rate judged as moderate benefit when considering health benefits of quitting and baseline risk. However, CI includes moderate harm and large benefit. | ⨁◯◯◯  VERY LOW |
| 2 | Smoking reduction  (change in mean number of daily cigarettes smoked since baseline; self-reported)  (fu: 24 weeks)  249 (1 RCT) | 7/10  (critical) | 5.0/10  (important) | At 24 weeks, the intervention group had a -9.1 change in mean number of daily cigarettes smoked since baseline and the control group had -5.5. SD were not reported. | | | Little to no difference | Unable to assess | Unable to assess | A difference in reduction by 3.6 cigarettes per day judged as little to no difference given harms of continuing to smoke. Reduction in smoking [still causes significantly increased morbidity and mortality](https://www.ncbi.nlm.nih.gov/pmc/articles/PMC2865193/). Insufficient data to determine imprecision. | ⨁◯◯◯  VERY LOW |
|  | Smoking reduction  (number of daily cigarettes smoked; self-reported)  (fu: 6 months)  140 (1 RCT) | 7/10  (critical) | 5.0/10  (important) | - | The mean number of daily cigarettes smoked was 13.45 (SD 6.49) | **MD 0.58 higher**  (1.82 lower to 2.98 higher) | Little to no difference | Little to no difference | Little to no difference | Point estimate, lower CI, and upper CI suggest little to no difference. | ⨁◯◯◯  VERY LOW |
|  | Smoking reduction  (eCO levels)  (fu: 6 months)  140 (1 RCT) | 7/10  (critical) | 5.0/10  (important) | - | The mean change in eCO levels was 6.52 (SD 10.24) | **MD 8.76 higher**  (5.17 higher to 12.35 higher) | Small but important harm | Small but important harm | Small but important harm | Point estimate, lower CI, and upper CI are judged as small but important harms given that the difference could correspond to an increase in smoking. | ⨁◯◯◯  VERY LOW |
| 5 | Serious adverse events  (fu: 12 weeks)  249 (1 RCT) | 7/10 (important) | 6.5/10 (important) | Serious adverse events were adjudicated by an end points evaluation committee and include death, respiratory, cardiovascular, neuropsychiatric or other events. At 12 weeks, the intervention group had experienced 4 (3.1%) and the control group had experienced 2 (1.7%). | | | Little to no difference | Unable to assess | Unable to assess | Insufficient data to determine imprecision. Data suggest little to no difference between groups but is difficult to interpret. | ⨁◯◯◯  VERY LOW |
|  | Serious adverse events  (fu: 12 to 24 weeks)  249 (1 RCT) | 7/10 (important) | 6.5/10 (important) | Serious adverse events were adjudicated by an end points evaluation committee and include death, respiratory, cardiovascular, neuropsychiatric or other events. At 12 to 24 weeks, the intervention group had experienced 2 (1.7%) and the control group had experienced 2 (1.7%). | | | Little to no difference | Unable to assess | Unable to assess | Insufficient data to determine imprecision. Data suggest little to no difference between groups. | ⨁◯◯◯  VERY LOW |
|  | Mild adverse events  (fu: 12 weeks)  249 (1 RCT) | 7/10 (important) | 6.5/10 (important) | Mild adverse events included cough, dry mouth, headache, rhinitis, throat irritation, dyspnea, sore throat, light headedness, dizziness, mouth irritation, nausea, indigestion, mouth ulcers, or vertigo. Only the first event for each participant in each category was counted. At 12 weeks, the intervention group had experienced 118 (93%) and the control group had experienced 88 (73%). | | | Small but important harm | Unable to assess | Unable to assess | Increase in mild adverse events judged as small but important harm when taking into account relative severity (may not be severe enough to prompt discontinuation, depending on the event and individual) and potential benefits of quitting smoking. However, difficult to interpret due to lack of baseline risk to compare for absolute differences. Insufficient data to determine imprecision. | ⨁◯◯◯  VERY LOW |
