# Supplementary material for: Effectiveness of e-cigarettes as a stop smoking intervention in adults: a systematic review
Source: Syst Rev. 2024 Jun 29;13:168. doi: 10.1186/s13643-024-02572-7 (PMC11218295; doi:10.1186/s13643-024-02572-7)
Supplement: Supplementary file 6 — Additional file 6: Appendix 6. Characteristics of included studies. [file 13643_2024_2572_MOESM6_ESM.docx]

**Appendix F. Characteristics of included studies**

| **Author Year, Country**  **Funding** | **Study details**  (*n; Setting; Follow-up*) | **Population Characteristics**  (*Age^A^; % male, % pregnant, Ethnicity, Quit details*) | **Intervention and Comparator**  (*n, type, nicotine dose, duration of intervention*) | **Outcomes** |
| --- | --- | --- | --- | --- |
| **Adriaens 2014,** Belgium [1] (RCT)  **Funding:** No external funding | n=48; one academic research setting, 8 months follow-up (8 weeks of lab sessions with 6 months further follow-up) | **Age**: 43.71 yrs (13.13)  **% male:** 43.75%  **% pregnant**: 0%  **Ethnicity:** NR  4.17% with concrete plans to quit. Participants undertook 1.60 (2.03) quit attempts. *[Mean (SD)]* | **Group 1 (n=16)**: Joyetech eGo-C, 18 mg/mL  **Group 2 (n=16)**: Kanger T2-CC, 18 mg/mL  E-cigarettes used ad libitum with or without cigarettes  **Group 3 (n=16)**: Wait-list controls. Instructed to continue smoking. At week 8, given e-cigarettes to smoke ad libitum  **Duration of intervention**: 8 weeks | **Adverse events**: Complaints at weeks 1, 2, 3-4, 5-6, and 7-8 |
| **Baldassarri 2018,** USA [2] (RCT)  **Funding:** Yale School of Medicine, Section of Pulmonary, Critical Care, and Sleep Medicine and the National Heart, Lung and Blood Institute grant | n=40; recruited from outpatient pulmonary and primary care clinics, Tobacco Treatment Service, and through referrals from medical providers; 24 weeks follow-up | **Age:** 53 yrs (10.1)  **% male:** 47.5%  **% pregnant**: 0%  **Ethnicity**: Non-white: 35%  100% of participants were willing to quit. No information on quit attempts. | **Group 1 (n=20)**: eGo style e-cig (2^nd^ generation), 24 mg/mL + standard care  **Group 2 (n=20)**: eGo style e-cig (2^nd^ generation), no nicotine + standard care  *Standard care consisted of nicotine patch and counselling sessions.*  **Duration of intervention**: 8 weeks, remaining 16 weeks allowed to use any available therapies for tobacco treatment | **Tobacco use abstinence at 24 weeks**: 7-day point prevalence abstinence and confirmed by exCO ≤6 ppm  **Reduction at 24 weeks**: Change in mean number of cigarettes smoked/day  **Adverse events**: (i) Total number of side effects; (ii) Total number of side effects experienced (abnormal dreams, anxiety, fatigue, headache, insomnia, nausea, palpitations, pruritus, cough, shortness of breath, sore throat, increased appetite) |
| **Bullen 2013,** New Zealand [3] (RCT)  **Funding:** Health Research Council of New Zealand | n=657; one study centre in Auckland with participants recruited from community; 6 months follow-up | **Age (by group)**: 43.6 yrs (12.7), 43.2 yrs (12.4), 40.4 yrs (13.0)  **% male:** 38%  **% pregnant**: 0%  **Ethnicity (by group): %** NZ Maori: Group 1: 33%; Group 2: 32%; Group 3: 32%  Participants described as wanting to quit. 55%, 53% and 57% of participants in each group reported at least 1 quit attempt in the last year. | **Group 1 (n=289):** Elusion e-cig, 16 mg/mL + referral to Quitline (low intensity telephone-based behavioural support)  **Group 2 (n=73):** No nicotine Elusion e-cig, 0 mg/mL + referral to Quitline  ***Group 3 (n=295):*** *Patches (NRT), 21 mg + referral to Quitline (Not included ^B^)*  **Duration of intervention:** 1 week before until 12 weeks after chosen quit day | **Tobacco use abstinence at 6 months**: (i) 7-day point prevalence abstinence; (ii) continuous abstinence confirmed by exCO ≤10 ppm  **Reduction at 6 months**: (i) Proportion of participants who reduced daily cigarettes by at least half  **Adverse events**: (i) Total AEs; (ii) Serious AEs (death, life threatening illness, admission to hospital or prolongation of hospital stay,  persistent or significant disability or incapacity, congenital abnormality, medically important) (iii) Any non-serious AE, (iv) AEs by relation to study treatment |
| **Caponnetto 2013** [4] ^C^ **& Russo 2016** [5], Italy (RCT)  **Funding**: Lega Italiana AntiFumo | n=300; one academic research setting; 12-months follow-up | **Age:** 44.0 yrs (12.5)  **% male:** 63%  **% pregnant**: 0%  **Ethnicity**: NR  Smokers were not intending to quit or wishing to do so in the next 30 days. 51% reported past attempts to quit with 0.6 (0.7) attempts. *[Mean (SD)]* | **Group 1 (n=100)**: Categoria e-cig (model 401), 7.2mg/mL  **Group 2 (n=100)**: Categoria e-cig (model 401), 7.2mg/mL (6 weeks), then 5.4mg/mL (6 weeks)  **Group 3 (n=100)**: Categoria e-cig (model 401), no nicotine  **Duration of intervention:** 12 weeks  **Comparison:** Group 1 vs Group 3; Group 2 vs Group 3 | **Tobacco use abstinence at 24 and 52 weeks**: Quit rates  **Reduction at 24 and 52 weeks**: (i) ≥50% reduction in the number of cigarettes/day since baseline; (ii) Median number of cigarettes/day; (iii) measured by eCO levels  **Adverse events**: (i) Serious AEs; (ii) Side effects; (iii) Total AEs at week 12 and 52 (among top five most commonly reported at baseline)  **Adverse outcome**: Weight gain from baseline at 12, 24 and 52 weeks (Russo 2016) |
| **Carpenter 2017**, USA [6] (RCT)  **Funding**: National Institutes of Health | n=68; one academic research setting; 16 weeks follow-up | **Age (by group)**: 43.3 yrs (14.4), 40.9 yrs (12.3), 42.3 yrs (14.2)  **% male:** 39.7%  **% pregnant**: 0%  **Ethnicity**: White: 51.5%; Black or African American: 48.5%  Motivation to quit in the next month was 5.0 (3.8) in group 1, 4.4 (3.1) in group 2 and 4.0 (3.9) in group 3 (as measured by the VAS scale, 0-10). 33.8% reported a quit attempt in the past year. | **Group 1 (n=25)**: BluCig (1^st^ generation), 16mg/mL  **Group 2 (n=21)**: BluPlus+, 24 mg/mL (patient directed use to maintain naturalistic environment)  **Group 3 (n=22)**: No Intervention  **Duration of intervention**: 3 weeks  BluCig 16 mg/mL was discontinued during the study and BluPlus+ 24 mg/mL was then offered. | **Adverse events during 16 weeks**: (i) Serious AEs; (ii) Total number of AEs; (iii) Number of participants experiencing AEs; (iv) All-cause mortality; (v) Total number of side effects experienced (cough, dizziness, nausea, throat/mouth irritation, heartburn, trouble sleeping, headache, general disorders: other) |
| **Cravo 2016**, UK [7] (RCT)  **Funding**: Fontem Ventures B.V. | n=408 (40 participants in cohort 2 spent one week in confinement); two research centres; 12 weeks follow-up | **Age (by group)**: 34.1 (10.6), 35.1 (10.6);  **% male:** 55.4%  **% pregnant**: 0%  **Ethnicity**: NR  Participants were excluded if they were trying to stop smoking or were considering quitting. No information on quit attempts. | **Group 1 (n=306)**: E-vapour product, 2.0% nicotine (2.7 mg/capsule) (choice between tobacco or menthol flavour)  **Group 2 (n=102)**: No Intervention (cigarette smokers who used their own usual cigarette brand)  **Duration of intervention**: 12 weeks | **Adverse events during 12 weeks**: (i) Total number of AEs; (ii) Number of participants experiencing AEs; (iii) Incidence least square mean of total AEs; (iv) Incidence least square mean of total AEs (excluding AEs related to nicotine withdrawal); (v) Serious AEs; (vi) AEs by severity; (vii) AEs by relationship to the product; (viii) AEs leading to study withdrawal; (ix) Frequency of each AE (59 AE types reported)  **Possible adverse outcomes**: Body weight at 12 weeks |
| **Eisenberg 2020,** Canada [8] (RCT)  **Funding**: Canadian Institutes of Health Research | n=376; 17 research sites/centres; 52 weeks follow-up with outcomes through 24 weeks reported | **Age (by group)**: 34.1 (10.6), 35.1 (10.6);  **% male:** 52.7%  **% pregnant**: 0%  **Ethnicity (by group)**:  Group 1: White: 94%; Black: 1%; Other: 6%;  Group 2: White: 87%; Black: 6%; Other: 7%;  Group 3: White: 86%; Black: 2%; Other: 12%  Participants had a moderate or strong desire and intention to attempt to quit (Motivation to Stop Scale ≥ 5). 91%, 93% and 89% in each group reported a previous quit attempt. | **Group 1 (n=128)**: NJOY e-cig, 15 mg/mL (tobacco flavour) + individual counseling (smoking cessation and relapse prevention counseling at baseline/follow-up)  **Group 2 (n=127)**: NJOY non-nicotine e-cig, 0 mg/mL + individual counseling  **Group 3 (n=121)**: Counseling alone  *Participants were instructed to use their e-cigarettes as desired*.  **Duration of intervention**: 12 weeks | **Reduction at 24 weeks**: (i) Change in Self-Reported Daily Cigarette Consumption from Baseline  **Adverse events :** (i) Serious AEs at 12 and 24 weeks; (ii) Mild AEs at 12 weeks |
| **Flacco 2019,** Italy [9] (Cohort)  **Funding**: University of Catania, crowdfunding (Kickstarter); First two years unfunded | N=1355; multiple health centers; 4 fp | **Age (by group)**: 48.4 yrs  **% male:** 56.3%  **% pregnant**: NR  **Ethnicity**: NR  No information on willingness to quit or past quit attempts. | **Group 1 (n=343)**: Users of any type of e-cig for ≥ 6 months at baseline, nicotine doses NR  **Group 2 (n=693)**: Smokers of ≥ 1 tobacco cigarettes daily for ≥ 6 months at baseline  ***Group 3 (n=319)****: Users of both tobacco and e-cig for ≥ 6 months at baseline (Not included ^D^)*  **Duration of intervention**: 4 years | **Quality of life:** (i) Change in self-reported health (assessed through the final item of the Italian version of the EuroQol EQ-D5L)  **Adverse events:** Serious AEs |
| **Holliday 2019**, UK [10] (RCT)  **Funding:** National Institute for Health Research Doctoral Research Fellowship | n=80 dental patients with periodontitis; one dental office; 6 months follow-up | **Age**: 44.3 (10.7) (Range 19-71)  **% male**: 47.5%  **% pregnant**: 0%  **Ethnicity**: White: 93.8%; Asian or Asian British: 6.3%  No information on willingness to quit or past quit attempts. | **Group 1 (n=40)**: E-cigarette starter kit (Vype 2^nd^ generation), choice of nicotine strength (0, 6, 12, 18 mg/mL) and flavour (2 week supply of e-liquid) + Usual care  **Group 2 (n=40)**: Usual care  *Usual care consisted of smoking cessation advice, a referral to stop smoking services was available, and standard non-surgical periodontal therapy*  **Duration of intervention**: 2 weeks | **Tobacco use abstinence at 6 months**: Continuous smoking abstinence, eCO verified  **Reduction at 6 months**: (i) Exhaled carbon monoxide; (ii) Salivary cotinine; (iii) Salivary anabasine  **Quality of life at 6 months**: Measured with the UK Oral Health-related Quality of Life measure  **Adverse events**: (i) Serious AEs; (ii) Total number of AEs; (iii) Total number of participants experiencing each AE and number of events  **Possible adverse outcomes**: Changes in emotional state measured with Mood and Physical Symptoms Scale |
| **Lucchiari 2020,** Italy [11] (RCT)  **Funding:** Umberto Veronesi (FUV) | n=210; European Institute of Oncology within the COSMOS II screening program; 12 months follow-up, but only 6 months follow-up reported | **Age**: 62.8 (4.58)  **% male**: 62.9%  **% pregnant**: 0%  **Ethnicity**: NR  Participants motivated to quit  (measured with 4-item questionnaire, scores ranging from 4 (low) to 19 (high); Group 1: 12.66, Group 2: 13.35, Group 3: 13.10) | **Group 1 (n=70)**: E-cig (VP5 electronic cigarettes kit, 8 mg/mL) + support (low-intensity counseling)  **Group 2 (n=70)**: Non-nicotine e-cig (VP5 electronic cigarettes kit, 0 mg/mL) + support  **Group 3 (n=70)**: Support only  **Duration of intervention**: 12 weeks | **Tobacco use abstinence at 6 months**: Continuous smoking abstinence, eCO verified ≤7ppm  **Reduction at 6 months**: (i) Number of daily cigarettes smoked; (ii) Exhaled carbon monoxide  **Possible adverse outcomes**: (i) Side effects likely to be related to e-cig use (self-reported) at 3 and 6 months; |
| **Walker 2020,** New Zealand [12] (RCT)  **Funding:** Funding  Health Research Council of New Zealand | n=1124; community-based trial with participants recruited via national media  advertising; 12 months | **Age (by group)**: Group 1: 41.4 (12.3); Group 2: 41.2 (12.6); Group 3: 42.3 (13.1)  **% male (by group)**: Group 1: 34%; Group 2: 30%; Group 3: 29%  **% pregnant**: 0%  **Ethnicity**: NZ Maori: Group 1: 40%; Group 2: 40%; Group 3: 40%  Participants motivated to quit in the next 2 weeks. 37%, 43% and 49% in each group, respectively, reported at least one previous quit attempt in the past 12 months. | **Group 1 (n=500)**: 2^nd^-generation eVOD E-cig (e-liquid of choice, 18 mg/mL) + standard care (24h Habitrol patch, 21 mg) + support (moderate-intensity behavioural support)  **Group 2 (n=499)**: Non-nicotine 2^nd^-generation eVOD E-cig (e-liquid of choice, 0 mg/mL) + standard care (24h Habitrol patch, 21 mg) + support  **Group 3 (n=125)**: standard care (24h Habitrol patch, 21 mg) + support  **Duration of intervention**: 14 weeks (2 weeks prior to quit date, and 12 weeks after quit date). Standard care consisted of 14 weeks of NRT treatment. | **Tobacco use abstinence at 6 months**: (i) Continuous smoking abstinence, eCO verified ≤9ppm; (ii) Self-reported quit rate; (iii) 7-day point-prevalence abstinence  **Reduction at 6 months**: (i) Change from baseline in the mean number of cigarettes smoked per day; (ii) ≥50% reduction in the number of cigarettes/day since baseline  **Adverse events**: (i) Serious AEs; (ii) Total number of participants with a serious AE  **Possible adverse outcomes**: (i) Self-reported change in BMI; (ii) Self-reported change in weight; (iii) Self-reported side effects in participants |
| **Myers Smith 2022**,  United Kingdom  (RCT)  **Funding:** Tobacco Advisory Group project grant, Cancer Research UK | N=135; participants recruited from the Academic research setting (Queen Mary University of London), which provided community stop-smoking service; 6 months | **Age (Median):** 40 years  **% male:** 51%  **% pregnant:** 0%  **Ethnicity:** White British: Group 1: 50%; Group 2: 52.2%  Participants were included if they had a history of unsuccessful quit attempts with stop smoking medications. No other information on quit attempts. | **Group 1 (n=68**): E-cigarette (Innokin T18E, Smok, and TECC mini with variable voltage). Participants were instructed to use one of these or any other product of their choice, along with the strength and flavor of their choice.  **Group 2 (n=67)**: NRT (choices included nicotine patch, chewing gum, nasal spray, microtab, inhalator and mouth spray)  **Duration of intervention**: 12 weeks | **Reduction of at least 50% at 6 months:**  self-reported reduction of ≥ 50% in the number of cigarettes smoked per day, confirmed by a reduction in end-expired CO levels of ≥ 50% compared to baseline  **Sustained abstinence at 6 months:** (i) no more than 5 cigarettes smoked since the contact at 4 weeks (ii) validated by CO reading of <8 p.p.m  **Other reduction outcomes**: Reduction in cigarette consumption at 4 weeks and self-reported abstinence at 4 weeks  **Adverse events:** Throat irritation, nausea, cough, itchiness, vivid dreams, hiccups, indigestion |
| **Xu 2023,** United States (RCT)  **Funding:**  Juul Labs | N=837;  Participants recruited from Research center (Center for Substance Use Research) via random sampling; 12 months | **Age:** 45.99 (11.48)  **% male:** 49.8%  **% pregnant:** 0%  **Ethnicity:** White: 82.4%; African American: 7.3%; Hispanic: 4.4%; Other: 5.9%  Individuals recruited did not necessarily plan to quit smoking. 2% plan to quit smoking in the next 30 days. | **Group 1 (n=566):** JUUL (Electronic nicotine delivery system [ENDS]); received two JUUL devices, charging dock, two to five packs of pods (four pods per pack, 5.0% nicotine by weight [59 mg/mL].  **Group 2 (n=271)**: Quit advice (printed materials explaining why quitting smoking is important and behavioural advice about how to quit smoking)  **Duration of intervention**: 6 months | **Daily cigarette consumption:** number of cigarettes consumed per day |
| **Carpenter 2023,** United States (RCT)  **Funding**: National Cancer Institute | N=638;  Participants recruited from the general community through online methods, which provided a direct link to a secure online platform for initial screening. Upon initial eligibility, individuals were subsequently consented through one of two channels (i.e., mail or synchronous tele-consent); 6 months | **Age:** Group 1: 42.4 (11.2)  Group 2: 42.0 (11.9)  **% male:** Group 1: 205%; Group 2: 91%  **% pregnant:** 0%  **Ethnicity:** Non-white: 31%; Hispanic or Latino: 14%  **Group 1:** Motivation to quit [Mean (SD)]: 4.3(3.3); Quit attempts in past year (%): 96 (22.5%)  **Group 2:** Motivation to quit [Mean (SD)]: 4.5(3.1); Quit attempts in past year N (%): 58 (27.5%) | **Group 1 (n=427):** E-cigarette (Njoy's pre-filled tank (3 ml nicotine [15 mg/ml] with sufficiently powered battery (1000 mAh) and participants could choose up to 2 flavors among 5 offered. Product was provided in two ∼2-week shipment.  **Group 2 (n=211)**: No E-cigarettes  **Duration of intervention:** 4 weeks | **7-day abstinence at each weekly visit;** Abstinence from cigarettes  **Floating abstinence:** having ever achieved 7-days of non-smoking throughout follow-up  **Reduction in tobacco smoking frequency**: Smoking reduction (≥50% reduction in cigarettes per day (CPD))  **Adverse events**: Total no. of adverse events (AEs), cough, headaches, increased phlegm |
| **Dawkins 2020,** United Kingdom (RCT)  **Funding**: National Institute for Health Research Public Health | N=80; Participants were recruited from four homeless centers across the UK; two in London (both residential centers), one in Northampton, and one in Edinburgh; 24 weeks | **Age:** Group 1: 42.75 (10.90); Group 2: 42.53 (10.78)  **% male**: 65%  **% pregnant:** 0%  **Ethnicity:** White: 76.3%; Asian/Asian British: 2.6%; Black/Black British: 11.4%; Mixed race: 10.2%  Motivation to stop smoking varied considerably; although, 6.3% reported not wanting to stop smoking | **Group 1 (n=48):** E-cigarette starter kit (a tank-style refillable EC with a choice of nicotine strength e-liquid [12 and 18 mg/ml] and flavors) and a guide to e-cigarettes fact sheet  **Group 2 (n=32)**: Usual care (study information sheet, brief advice to quit and help-quit leaflet)  **Duration of intervention**: 4 weeks | **Tobacco use abstinence:** a) CO-validated sustained smoking abstinence (per-protocol [PP] analysis); b) CO-validated sustained smoking abstinence (ITT analysis); c) 7-day point prevalence abstinence (per-protocol analysis)  **Reduction in tobacco smoking frequency:** a) 50% reduction in cigarettes smoked per day (PP analysis) b) 50% reduction in expired CO levels (PP analysis)  **Quality of Life:** a) HRQoL (EQ-5D-3L) per protocol analysis b) HRQoL EQ VAS (perceived health on the day of administration, ranging from 0 (death) to 100 (perfect health)  **Adverse events:** Mental Health Status |
| **Lucchiari 2022,** Italy (RCT)  **Funding:** Fondazione Umberto Veronesi & Italian Ministry of Health | N=210; Participants recruited at the Academic research setting (University of Milan & European Institute of Oncology) as a part of lung cancer screening program; 12 months | **Age:** 62.8 years (4.58)  **% male**: 64.6%  **% pregnant:** 0%  **Ethnicity:** NR  Participants who had motivational score > 10 and not be treated at a smoking center | **Group 1 (n=70):** E-cigarette kit and 12 10-mL nicotine liquid cartridges (8 mg/mL) + psychological counselling  **Group 2 (n=70)**: Placebo-control (non-nicotine) e-cigarettes + psychological counselling  **Group 3 (n=70)**: Psychological counselling  **Duration of intervention**: 3 months | **Reduction in smoking-related respiratory symptoms** (Cough, Breathlessness, Bronchitis, Catarrh)  **Success rate** of smoking cessation attempts and daily smoking reduction  **Safety and toxicity** for E-cigs and placebo arm |
| **Foulds 2022,**  United States (RCT)  **Funding**: National Institute on Drug Abuse (NIDA) & Centre for Tobacco Products of the US Food and Drug Administration | N=520;  Participants recruited at the Academic medical centers (Penn State Hershey and Virginia Commonwealth University); 6 months | **Age:** 46.2 years (11.6)  **% male**: 41.2%  **% pregnant**: 0%  **Ethnicity:** 67.3% Caucasian/White Non-Hispanic; 27.9% African American/Black Non-Hispanic;  4.8% Other  Participants interested in reducing their cigarette consumption by 50% had no plans to quit within 6 months | **Group 1 (n=130):** non-nicotine cigarette substitute (plastic tube with no electronics or aerosol)  **Group 2 (n=130):** an eGO style ENDS with no nicotine (0 mg/mL)  **Group 3 (n=130):** an eGO style ENDS with nicotine (8 mg/mL)  **Group 4 (n=130):** an eGO style ENDS with nicotine (36 mg/mL)  **Duration of intervention: 24 weeks** | **Tobacco use abstinence** 7-day point prevalence abstinence biochemically confirmed by exhaled CO (<10ppm) at week 24, 28+ days abstinent with CO < 10 ppm at weeks 20 and 24, Mean days on days of no cigarette smoking from week 1 to 24. |

NR: not reported; NRT: nicotine replacement therapy; SD: standard deviation

A Age in years presented with Mean (SD) unless otherwise stated.

B This group was not included in this systematic review because it did not meet the comparator group inclusion criteria.

C This study also has two companion studies that present the results by smoking phenotype (i.e., quitters, reducers, failures) regardless of intervention group.

D This group was not included in this systematic review because dual users are not an acceptable comparator group.
