# Supplementary material for: Effectiveness of e-cigarettes as a stop smoking intervention in adults: a systematic review
Source: Syst Rev. 2024 Jun 29;13:168. doi: 10.1186/s13643-024-02572-7 (PMC11218295; doi:10.1186/s13643-024-02572-7)
Supplement: Supplementary file 7 — Additional file 7: Appendix 7. Results tables and RoB assessments. [file 13643_2024_2572_MOESM7_ESM.docx]

# Appendix G. Comparison results

F.1. E-cigarette with nicotine vs no intervention

F.2 E-cigarette with nicotine vs waitlist

F.3 E-cigarette with nicotine + usual care vs Usual care

F.4 E-cigarette with nicotine + standard care vs Standard care

F.5 E-cigarette with nicotine vs E-cigarette with no nicotine (NNEC)

F.6 E-cigarette with nicotine + other smoking cessation treatment vs E-cigarette with no nicotine + other smoking cessation treatment

F.7 E-cigarette with nicotine + other smoking cessation treatment vs no intervention + other smoking cessation treatment

F.8 E-cigarette with nicotine + other smoking cessation treatment + standard care vs. E-cigarette with no nicotine + other smoking cessation treatment + standard care

F.9 E-cigarette with nicotine + other smoking cessation treatment + standard care vs. other smoking cessation treatment + standard care

F.10 E-cigarette with no nicotine combined with other smoking cessation treatment + standard care vs. other smoking cessation treatment + standard care

F.11 E-cigarette with no nicotine combined with other smoking cessation treatment vs no intervention + other smoking cessation treatment

F. 12 E-cigarette with nicotine vs. Other smoking cessation treatment (NRT choices included nicotine patch, chewing gum, nasal spray, microtab, inhalator and mouth spray)

F. 13 E-cigarette with nicotine vs. Other smoking cessation treatment (Quit advice)

F. 14 E-cigarette with nicotine vs. No E-cigarette

F. 15 E-cigarette with nicotine vs. other smoking cessation intervention (Usual Care)

F. 16 E-cigarette with nicotine + Support (psychological counselling) vs. E-cigarette without nicotine + Support (psychological counselling)

F. 17 E-cigarette with nicotine + Support (psychological counselling) vs. Support (psychological counselling)

F. 18 E-cigarette with nicotine + vs. Non-nicotine cigarette substitute

F. 19 E-cigarette with nicotine + vs. E-cigarette without nicotine

## Appendix F.1. E-cigarette with nicotine vs No intervention

### Results table

| ***Study details*** | ***Outcome details*** | ***E-cigarette^A^*** | ***No intervention^B^*** | ***Notes*** |
| --- | --- | --- | --- | --- |
| **Adverse events (duration of the trial)** | | **n/N (%); number of events** | |  |
| Carpenter 2017 [66], USA [RCT] ^C^ | All-cause mortality | 0/46 | 0/22 | Duration of trial: 16 weeks    Patient directed use of e-cig to maintain naturalistic environment.    AEs were collected by systematic assessment (no further details) |
|  | Serious AEs | 0/46 | 0/22 |  |
|  | # of participants experiencing AEs; # events  Cough  Dizziness  Nausea  Throat/mouth irritation  Heartburn  Trouble sleeping  Headache  General disorders: other | 20/46 (43.5%); 38  11/46 (23.9%); 12  2/46 (4.3%); 2  8/46 (17.4%); 9  6/46 (13%); 6  1/46 (2.2%); 1  2/46 (4.3%); 2  4/46 (8.7%); 4  2/46 (4.3%); 2 | 8/22 (36%); 29  4/22 (18.2%); 6  1/22 (4.6%); 1  4/22 (18.2%); 4  5/22 (22.7%); 5  2/22 (9.1%); 2  3/22 (13.6%); 4  4/22 (18.2%); 7  0/22 (0%); 0 |  |
| Cravo 2016 [65], UK [RCT] | # of participants experiencing AEs; # events | 271/306 (88.6%); 1515^D^ | 80/102 (78.4%); 225 | Duration of trial: 12 weeks    AEs were monitored at every study visit.    59 different AE events were reported^E^ |
|  | Incidence of:  Total AEs  Total AEs excluding AEs related to nicotine withdrawal^D^ | Least square mean (95%CI): 1.60 (1.55 to 1.65)  1.20 (1.14 to 1.27) | Least square mean (95%CI): 0.79 (0.66 to 0.92)  0.79 (0.66 to 0.92) |  |
|  | Serious AEs | 5/1515 (0.3%) | 0/225 (0%) |  |
|  | Mild severity  Moderate severity  Severe severity | 449/1515 (29.6%)  827/1515 (54.6%)  239/1515 (15.8%) | 64/225 (28.4%)  129/225 (57.3%)  32/225 (14.2%) |  |
|  | Almost definitely related  Possibly related  Probably related  Unlikely to be related  Unrelated | 19/1515 (1.3%)  752/1515 (49.6%)  71/1515 (4.7%)  246/1515 (16.2%)  427/1515 (28.2%) | 0/225 (0%)  0/225 (0%)  0/225 (0%)  0/225 (0%)  225/225 (100%) |  |
|  | AEs leading to study withdrawal | 2/1515 (0.1%) | 0/225 (0%) |  |
| Flacco 2019 [74], Italy [Cohort] | Serious AEs | 0/228 | 0/471 | Based on text stating that “no serious adverse events and no higher risk were reported for e-cig when compared to traditional tobacco smoking”. |
| **Quality of Life** | | **Mean (SD)** | |  |
| Flacco 2019 [74], Italy [Cohort] | Change in self-reported health (baseline-4 years; assessed through the final item of the Italian version of the EuroQol EQ-D5L) | -0.3 (1.5); n=123 | -0.1 (1.3); n=409 | Timepoint: 4 years  Only including participants with 4-year follow-up data.    Includes only participants who did not change their exposure status during the follow-up. |
| **Possible adverse outcomes** | | **Mean (SD); n** | |  |
| Cravo 2016 [65], UK [RCT] | Body weight at baseline and at the end of study (12 weeks) | BL: 75.8 kg (14.0); 12 w: 76.1 kg (13.81); n=306  *Mean difference (SD):*  *0.3 kg (17.03)†* | BL: 74.0 kg (13.5); 12 w: 74.1 kg (13.5); n=102  *Mean difference (SD):*  *0.1 kg (16.53)†* |  |

A Carpenter 2017: BluCig 16mg/mL and BluPlus+ 24 mg/mL results combined. BluCig 16 mg/mL was discontinued during the study and BluPlus+ 24 mg/mL was then offered. Data available for both groups separately; Cravo 2016: E-vapour product 2.0% nicotine (2.7 mg/capsule); Flacco 2019: Users of any type of e-cig for ≥ 6 months at baseline (nicotine dose not measured)

B Flacco 2019: Smokers of ≥ 1 tobacco (only) cigarette daily for ≥ 6 months at baseline

C AEs retrieved from the clinical registry (https://clinicaltrials.gov/ct2/show/results/NCT02357173?term=carpenter&cond=Smoking&draw=2&rank=7)

D 495 AEs related to nicotine withdrawal (all in EVP group)

E  Cough, headache, nasopharyngitis, irritability, Increased appetite, dry throat, rhinorrhea, dyspnea, disturbance in attention, dizziness, nausea, toothache, abdominal pain upper, dyspepsia, diarrhea, dry mouth, constipation, abdominal discomfort, abdominal pain, oral pain, upper respiratory tract infection, gastroenteritis, influenza, rhinitis, tonsillitis, oral herpes, anger, depressed mood, frustration, impatience, insomnia, middle insomnia, restlessness, anxiety, sleep disorder, abnormal dreams, nightmare, nervousness, hunger, fatigue, chest  discomfort, pain, back pain, arthralgia, musculoskeletal chest pain, neck pain, ligament sprain, dysmenorrhea, rash, seasonal allergy, palpitations, lymphadenopathy, ear pain, desire to smoke, mouth ulceration, sore throat, vomiting, weight increased, nasal congestion

† Values in Italics were calculated by the review team in Excel using imputation assuming a correlation of 0.25.

BL: Baseline

12 w: 12 weeks

Abbreviations: AE= Adverse event; CI= Confidence interval; SD= Standard deviation

### Forest Plot

#### P.1.1 Outcome: Body weight


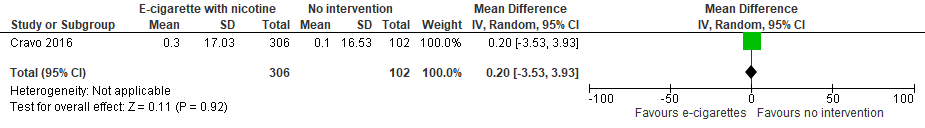


### RoB results

**Summary**

| **Author/Year** | **Sequence generation** | **Allocation Concealment** | **Blinding of Participants/ Personnel** | **Blinding of Outcome Assessors** | **Incomplete Outcome Data** | **Selective Outcome Reporting** | **Other** | **Overall ROB** |
| --- | --- | --- | --- | --- | --- | --- | --- | --- |
| **Adverse events** | | | | | | | | |
| ***Complaints*** | | | | | | | | |
| Adriaens 2014 (71) | Low | Unclear | High | High | Low | Unclear | Low | High risk |

**Ratings with judgement support**

| **Adriaens 2014**  **Outcome:** Complaints | | |
| --- | --- | --- |
| **Risk of bias domain** | **Rating** | **Judgement support** |
| Sequence generation | Low risk | Block randomization using a randomization tool available on website: www.randomizer.org |
| Allocation Concealment | Unclear risk | Insufficient information reported. |
| Blinding of Participants/ Personnel | High risk | The participants and personnel were not blinded and complaints were collected through online questionnaires from the participants. Knowledge of assignment group could influence reporting. |
| Blinding of Outcome Assessors | High risk | Self-reported outcome. |
| Incomplete Outcome Data | Low risk | Little loss to follow-up during the 8-weeks of lab sessions (one in each group). |
| Selective Outcome Reporting | Unclear risk | No protocol, so it is unclear if all outcomes were reported. AEs reported as "complaints", but actual complaints not reported. |
| Other | Low risk | No other concerns. |
| **Overall ROB** | **High risk** | **Lack of blinding a major concern complaints outcome, as it is a subjective outcome reported by the participants.** |

### RoB results

**Summary**

| **Author/Year** | **Sequence generation** | **Allocation Concealment** | **Blinding of Participants/ Personnel** | **Blinding of Outcome Assessors** | **Incomplete Outcome Data** | **Selective Outcome Reporting** | **Other** | **Overall ROB** |
| --- | --- | --- | --- | --- | --- | --- | --- | --- |
| **Adverse events** | | | | | | | | |
| ***All-cause mortality*** | | | | | | | | |
| Carpenter 2017 (66) | Unclear | Unclear | Low | Low | Unclear | Low | Low | **Unclear risk** |
| ***All serious and non-serious AEs*** | | | | | | | | |
| Carpenter 2017 (66) | Unclear | Unclear | High | High | Unclear | Low | Low | **High risk** |
| Cravo 2016 (65) | Unclear | Unclear | High | High | Low | High | High | **High risk** |
| Flacco 2019 (4) |  |  |  |  |  |  |  | **Acceptable (+)** |
| **QUALITY OF LIFE** | | | | | | | | |
| ***Change in self-reported health*** | | | | | | | | |
| Flacco 2019 (74) |  |  |  |  |  |  |  | **Acceptable (+)** |
| **Possible adverse outcomes** | | | | | | | | |
| ***Body weight*** | | | | | | | | |
| Cravo 2016 (65) | Unclear | Unclear | Unclear | Unclear | Low | High | High | **High risk** |
| ***Any possibly smoking related disease, cancer, mouth irritation*** | | | | | | | | |
| Flacco 2019 (74) |  |  |  |  |  |  |  | **Acceptable (+)** |

**Ratings with judgement support**

| **Carpenter 2017**  **Outcomes:** All-cause mortality and All serious and non-serious AEs | | |
| --- | --- | --- |
| **Risk of bias** | **Rating** | **Judgement support** |
| Sequence generation | Unclear risk | Method of allocation sequence not reported. |
| Allocation Concealment | Unclear risk | No details about allocation concealment. |
| Blinding of Participants/ Personnel | Outcome: AE – all-cause mortality: Low risk | Participants would not have been blind to group allocation (particularly in the e-cig vs control groups), however, as all-cause mortality is an objective outcome, knowledge of group allocation is not likely to influence the outcome result. |
|  | Outcome: AE – all serious and non-serious AEs: High risk | Participants would not have been blind to group allocation (particularly in the e-cig vs control groups), which could influence the reporting of adverse events. |
| Blinding of Outcome Assessors | Outcome: All-cause mortality: Low risk | Outcome assessors not blinded, however, as all-cause mortality is an objective outcome, knowledge of group allocation is not likely to influence the outcome result. |
|  | Outcome: All serious and non-serious AEs: High risk | Lack of blinding of outcome assessors could influence enquiry about adverse events. |
| Incomplete Outcome Data | All outcomes: Unclear risk | 73.5% of the participants completed study participation (at 16-weeks), with little difference between groups. Side effects were collected throughout the duration of the study. It is unclear how many participants contributed to this data, although the %'s given in the text point to all participants, but it is unclear how this would be possible as not everyone completed the 4-week sampling period. |
| Selective Outcome Reporting | Low risk | Although the methods section and trial registry do not report that adverse events will be explored, you would expect this to be an outcome in any trial in this area of research. |
| Other | Low risk | No other concerns. |
| **Overall ROB** | **Outcome: AE – all-cause mortality: Unclear risk** | **Several domains rated as unclear, mainly due to insufficient details in reporting.** |
|  | **Outcome: AE – all serious and non-serious AEs: High risk** | **Lack of blinding a major concern for adverse events/side effects outcome, as it is a subjective outcome reported by the participants.** |

Abbreviations: AE= Adverse event

| **Cravo 2016**  **Outcomes:** All serious and non-serious AEs and Body weight | | |
| --- | --- | --- |
| **Risk of bias** | **Rating** | **Judgement support** |
| Sequence generation | Unclear risk | Only states that randomization was performed using an interactive Web Response system. There are no further details. |
| Allocation Concealment | Unclear risk | It is unclear if allocation was concealed or how the information was shared with study personnel or participants. |
| Blinding of Participants/ Personnel | Outcome: AE – all serious and non-serious AEs: High risk | Open-label, which could impact reporting of adverse outcomes/events. |
|  | Outcome: Possible Adverse Outcomes – Body weight: Unclear risk | This was an open-label study, and usually weight is an objective variable, but the study authors do not provide any information on how body weight was collected. |
| Blinding of Outcome Assessors | Outcome: AE – all serious and non-serious AEs: High risk | Open-label. Outcome assessors were not blinded and could have influenced results. How outcomes were measured (self report, etc), not explicitly stated. |
|  | Outcome: Possible Adverse Outcomes – Body weight: Unclear risk | This was an open-label study, and usually weight is an objective variable, but the study authors do not provide any information on how body weight was collected. |
| Incomplete Outcome Data | Outcome: AE – all serious and non-serious AEs: Low risk | Significantly more drop outs in the e-cigs arm (7% (n=20) vs. 0.009% (n=1)), but still a fairly low number. Those who contributed to the AEs outcomes covered almost all participants. Although 2 withdrew because of AEs and 1 due to death, these were considered in the AE results. |
|  | Outcome: Possible Adverse Outcomes – Body weight: Low risk | Significantly more drop outs in the e-cigs arm (7% (n=20) vs. 0.009% (n=1)), but still a fairly low number. It is unclear how many participants contributed to the end of study weight (assuming only those who completed the study). |
| Selective Outcome Reporting | High risk | The only two outcomes reported in clinical trials registry (NCT02029196) are adverse events and exhaled carbon monoxide. This study also provided several other outcomes (e.g., vital signs, lung function tests, haematology). |
| Other | High risk | The funders of this project was the company who made the e-cigarette prototype. |
| **Overall ROB** | **Outcome: AE – all serious and non-serious AEs: High risk** | **Several domains at high risk of bias.** |
|  | **Outcome: Possible Adverse Outcomes – Body weight: High risk** | **Several domains at high risk of bias.** |

Abbreviations: AE= Adverse event

| **Flacco 2019**  **Outcomes:** Serious adverse events, Quality of life (Change in self-reported health) | | |
| --- | --- | --- |
| **Question** | **Rating** | **Judgement support** |
| **Internal Validity: Selection of Subjects** | | |
| The study addresses an appropriate and clearly focused question. | Yes |  |
| The two groups being studied are selected from source populations that are comparable in all respects other than the factor under investigation. | Can’t say | The two groups are selected from the same source. However, it is difficult to determine comparability, as the authors did not provide a table of baseline characteristics. |
| The study indicates how many of the people asked to take part did so, in each of the groups being studied. | No | No indication of how many were asked to participate. |
| The likelihood that some eligible subjects might have the outcome at the time of enrolment is assessed and taken into account in the analysis. | Can’t say | Unclear if participants were assessed for previous adverse events or adverse outcomes prior to study start. |
| What percentage of individuals or clusters recruited into each arm of the study dropped out before the study was completed. | E-cig – 33.5%  Dual – 32.3%  Tobacco 32% |  |
| Comparison is made between full participants and those lost to follow up, by exposure status | No | No comparison made by exposure status. |
| **Internal Validity: Assessment** | | |
| The outcomes are clearly defined. | All outcomes: Yes |  |
| The assessment of outcome is made blind to exposure status. | All outcomes: Can’t say | Health data was collected by both self-report and direct visits or hospital discharge abstracts. Unclear if personnel who collected outcome information were blinded. |
| Where blinding was not possible, there is some recognition that knowledge of exposure status could have influenced the assessment of outcome. | All outcomes: No |  |
| The method of assessment of exposure is reliable. | Yes | While different e-cigarette models with various nicotine doses might introduce heterogeneity into study, authors intended study to be pragmatic and represent real-world experiences. |
| Evidence from other sources is used to demonstrate that the method of outcome assessment is valid and reliable. | Quality of Life: Yes | References to validated EuroQol EQ-D5L questionnaire provided. |
| Exposure level or prognostic factor is assessed more than once. | Yes | Assessed if participants switched exposure groups and performed sensitivity analyses to determine effect. |
| **Internal Validity: Confounding** | | |
| The main potential confounders are identified and taken into account in the design and analysis. | No | Authors controlled for a number of baseline characteristics, but did not include information on previous quit attempts or motivation to quit. |
| **Internal Validity: Statistical Analysis** | | |
| Have confidence intervals been provided? | Yes |  |
| **Overall Assessment of the Study** |  |  |
| How well was the study done to minimise the risk of bias or confounding? | **Acceptable (+)** | Most criteria are met. Some concern of attrition bias (unable to evaluate reasons for loss to follow-up and loss to follow-up was greater than 20%). Concerns with outcome assessment, as it was unclear who performed the assessment. |

## Appendix F.2. E-cigarette with nicotine vs Waitlist

### Results table

| ***Study details*** | ***Outcome details*** | ***Timepoint*** | ***Joyetech eGo***  ***(18 mg/mL)*** | ***Kanger T2-CC***  ***(18 mg/mL)*** | ***Waitlist*** | ***Notes*** |
| --- | --- | --- | --- | --- | --- | --- |
| **Adverse events** | | | **Mean (SE) (n=16 in each group)** | | |  |
| Adriaens 2014 (71), Belgium [RCT] | Complaints^A^ | Week 1 | 2.07 (0.39) | 2.92 (0.38) | 3.46 (0.35) | E-cigarettes used ad libitum with or without cigarettes. AEs were self-reported among participants. Data was provided by the study author upon request. |
|  |  | Week 2 | 1.80 (0.35) | 2.23 (0.34) | 3.11 (0.31) |  |
|  |  | Weeks 3-4 | 1.82 (0.32) | 2.09 (0.31) | 2.95 (0.29) |  |
|  |  | Weeks 5-6 | 1.89 (0.35) | 2.11 (0.34) | 2.99 (0.32) |  |
|  |  | Weeks 7-8 | 2.16 (0.39) | 2.23 (0.37) | 3.10 (0.35) |  |

A Complaints questioned in online diaries included bad taste, dry mouth/throat, irritated mouth/throat, dizziness, headache, nausea, increased heart rate/palpitations, increased weight, or concerns about health risks.

Abbreviations: AE= Adverse event; SE= Standard error

## Appendix F.3. E-cigarette with nicotine + Usual care vs Usual care

### Results table

| ***Study details*** | ***Outcome details*** | ***E-cigarette ^A^***  ***+ Usual care ^B^*** | ***Usual care ^B^*** | ***Notes*** |
| --- | --- | --- | --- | --- |
| **Tobacco use abstinence** | | **Rate (95% CI); n** | |  |
| Holliday 2019 (69), UK [RCT] | Continuous eCO-verified smoking abstinence (Russell Standard eCO) at 6 months | 15% (7 to 29%); n=40  *6/40* | 5% (1 to 17%); n=40  *2/40* | Rate converted to n/N to produce RR (95%CI). |
| **Reduction in tobacco smoking frequency/quantity** | | **Mean change (SD) from baseline to 6 months [95%CI]; n** | |  |
| Holliday 2019 (69), UK [RCT] | eCO levels | -12.0 ppm (11.0)  [-16.2 to -7.9]; n=29 | -5.8 ppm (12.3)  [-10.5 to -1.1]; n=29 |  |
|  | Salivary cotinine | -62.2 ng/ml (132.3)  [-112.5 to -11.8]; n=29 | -37.1 ng/ml (133.4)  [-90.0 to -15.7]; n=29 |  |
|  | Salivary anabasine | -0.4 ng/ml (1.2)  [-0.9 to 0.0]; n=29 | 0.5 ng/ml (2.3)  [-0.5 to 1.4]; n=29 |  |
| **Quality of Life** | | **Mean change (SD) from baseline to 6 months [95%CI]; n** | |  |
| Holliday 2019 (69), UK [RCT] | UK Oral Health-Related Quality of Life measure | 9.6 (13.2) [4.6 to 14.6]; n=29 [Baseline Mean (SD): 43.4 (7.7)] | 8.2 (15.1) [2.4 to 14.0]; n=29  [Baseline Mean (SD): 43.1 (6.7)] | Self-reported questionnaire. |
| **Adverse events** | | **n/N; # of events** | |  |
| Holliday 2019 (69), UK [RCT] | Serious AEs | 0/40 | 0/40 | Patients all had periodontitis. AEs were monitored at each study visit for the duration of the trial (6 months). A patient may have reported more than one AE.  The text says there were 56 AEs, but Table 3 reports 55 events. |
|  | Total number of AEs  Toothache  Dentine hypersensitivity  Dental/periodontal abscess  Tooth/teeth loss  Mouth ulceration  Soreness of intra-oral soft tissues  Fractured/carious filling or tooth  Other | 20 events  9/40; 11 events  3/40; 3 events  3/40; 3 events  3/40; 5 events (9 teeth)  2/40; 2 events  3/40; 3 events  2/40; 2 events  5/40; 6 events | 35 events  4/40; 4 events  3/40; 3 events  2/40; 2 events  4/40; 5 events (6 teeth)  0/40  0/40  3/40; 3 events  2/40; 3 events |  |
| **Possible adverse outcomes: Change in emotional state** | | **Mean change (SD) from baseline to 6 months [95%CI]; n** | |  |
| Holliday 2019 (69), UK [RCT] | measured with Mood and Physical Symptoms Scale at 6 months | -2.8 (8.8) [-6.1 to 0.6]; n=29  [Baseline Mean (SD): 21.8 (4.9)] | -2.8 (8.3) [-6.0 to 0.3]; n=29  [Baseline Mean (SD): 22.8 (7.5)] | Self-reported questionnaire |

A Vype e-cigarette starter kits nicotine levels were available in 0, 6, 12, and 18 mg/mL and the participants were able to select which dosage

B Usual care: smoking cessation advice, a referral to stop smoking services was available, and standard non-surgical periodontal therapy

Abbreviations: AE= Adverse event; CI= Confidence interval; eCO= Exhaled carbon monoxide; SD= Standard deviation; RR= Risk ratio

### Forest Plots

#### P.3.1 Outcome: Tobacco use abstinence


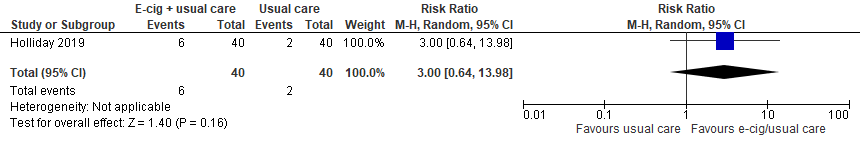


#### P.3.2 Outcome: Reduction: eCO levels, salivary cotinine, salivary anabasine


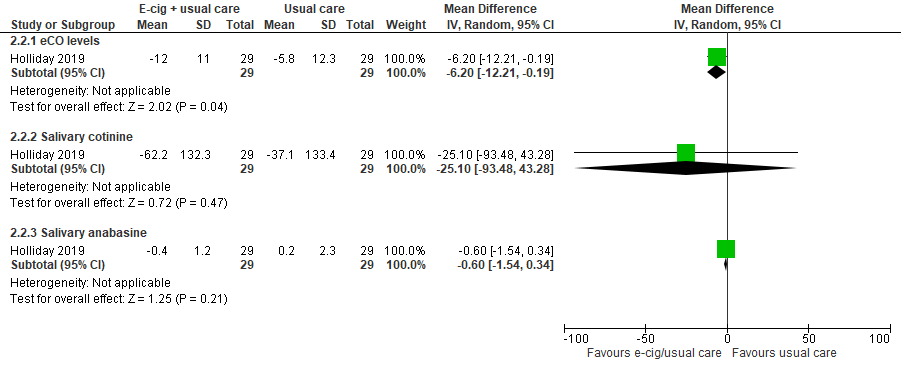


#### P.3.3 Outcome: Quality of life


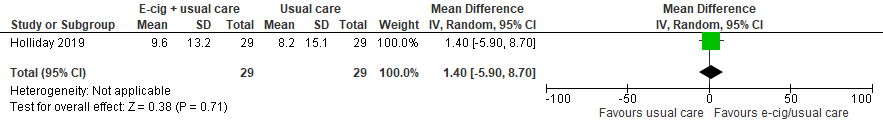


#### P.3.4 Outcome: Possible adverse outcomes: Change in emotional state


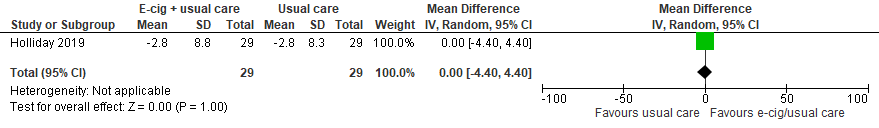


### RoB results

**Summary**

| **Author/Year** | **Sequence generation** | **Allocation Concealment** | **Blinding of Participants/ Personnel** | **Blinding of Outcome Assessors** | **Incomplete Outcome Data** | **Selective Outcome Reporting** | **Other** | **Overall ROB** |
| --- | --- | --- | --- | --- | --- | --- | --- | --- |
| **Tobacco use abstinence** | | | | | | | | |
| ***Continuous eCO-verified smoking abstinence*** | | | | | | | | |
| Holliday 2019 (69) | Low | Low | Low | Low | High | High | High | **High risk** |
| **Reduction in tobacco smoking frequency/quantity** | | | | | | | | |
| ***Exhaled carbon monoxide (eCO) levels, Salivary cotinine, Salivary anabasine*** | | | | | | | | |
| Holliday 2019 (69) | Low | Low | Low | Low | High | High | High | **High risk** |
| **Quality of life** | | | | | | | | |
| ***Oral Health-Related Quality of Life*** | | | | | | | | |
| Holliday 2019 (69) | Low | Low | High | High | High | High | High | **High risk** |
| **Adverse events** | | | | | | | | |
| ***All serious and non-serious AEs*** | | | | | | | | |
| Holliday 2019 (69) | Low | Low | High | High | High | High | High | **High risk** |
| **Possible adverse outcomes** | | | | | | | | |
| ***Change in emotional state*** | | | | | | | | |
| Holliday 2019 (69) | Low | Low | High | High | High | High | High | **High risk** |

**Ratings with judgement support**

| **Holliday 2019**  **Outcomes:** Abstinence, Reduction, Quality of life, All serious and non-serious AEs, Change in emotional state | | |
| --- | --- | --- |
| **Risk of bias** | **Rating** | **Judgement support** |
| Sequence generation | Low risk | Random permuted blocks of variable size (2, 4 or 6). |
| Allocation Concealment | Low risk | Allocation schedule was generated by a statistician with no other involvement in the study. Secure- password protected web based system. |
| Blinding of Participants/ Personnel | Outcome: continuous eCO-verified smoking abstinence, eCO level, salivary cotinine, salivary anabasine: Low risk | Participants and personnel were not blinded, however, as quit status was confirmed with biomarker levels which could not be impacted by the participant, there was low risk. |
|  | Outcomes: oral health-related quality of life, All serious and non-serious AEs, Change in emotional state: High risk | Participants and personnel were not blinded to group allocation. |
| Blinding of Outcome Assessors | Outcome: continuous eCO-verified smoking abstinence, eCO level, salivary cotinine, salivary anabasine: Low risk | Quit status at 6 months was measured with the Russell Standard which includes continuous eCO-verified smoking abstinence. To measure reduction, there were three biomarkers: 1. eCO level, which was assessed by an non-blinded member of the team; 2. salivary cotinine; and 3. salivary anabasine which were both assessed at an external lab, where it is unlikely they were aware of group assignment. |
|  | Outcomes: oral health-related quality of life, All serious and non-serious AEs, Change in emotional state: High risk | Outcome assessors, in this case the participants, were not blinded. Adverse events, responses to the Mood and Physical symptom scale and QoL were self-reported. |
| Incomplete Outcome Data | All outcomes: High risk | Number lost in each arm was the same with reasons balanced, however the loss to follow-up at 6 months was high (11/40 (27.5%) in each group). |
| Selective Outcome Reporting | High risk | There are some primary outcomes listed in the trial registry that were not reported in this study (e.g., Periodontal Inflammed Surface Area [PISA]). Additionally, there are some outcomes reported in this study (e.g., QoL) that were not listed in the trial registry or mentioned in the amendments to the protocol additional file 2. |
| Other | High risk | Contamination of control group was noted. 8 participants in the control group (20%, 95% CI 11-35%) reported using an e-cigarette at some point during the study (against instructions), with one reporting usage at all post-randomisation visits. |
| **Overall ROB** | **All outcomes: High risk** | **Due to reporting some outcomes that were not listed in the registry and not reporting others. Additionally, several participants in control group used intervention. Lack of blinding was a concern for the subjective outcomes, as they were reported by the participants.** |

Abbreviations: AE= Adverse event; eCO= Exhaled carbon monoxide

## Appendix F.4. E-cigarette with nicotine + Standard care vs E-cigarette with no nicotine + Standard care

### Results table

| ***Study details*** | ***Outcome details*** | ***E-cigarette ^A^ (*24 mg/mL*) + Standard care^B^*** | ***E-cigarette ^A^ (0 mg/mL) + Standard care^B^*** | ***Notes*** | |
| --- | --- | --- | --- | --- | --- |
| **Tobacco use abstinence** | | **n/N (%)** | | |  |
| Baldassarri 2019 (70), USA [RCT] | 7 day point prevalence confirmed by exCO ≤6ppm measured at 24 weeks | 4/20 (20%) | 2/20 (10%) | E-cig (24 mg/mL and 0 mg/mL) and patch for 8 weeks, allowed any intervention for remaining 16 weeks. | |
|  |  | 95%CI: 0.36 to 14.0, p=0.66  *Odds Ratio 2.25 (95% CI 0.36 to 13.97); p=0.38†‡* | |  |  |
| **Reduction in tobacco smoking frequency/quantity** | | **Mean (SD); n** | |  | |
| Baldassarri 2019 (70), USA [RCT] | Change in mean number of cigarettes smoked/day measured at 24 weeks | -5.5 (11.5); n=20 | -8.04 (11.6); n=20 | 95%CI is as reported in the study, although it is unclear which data this corresponds to. | |
|  |  | 95%CI: -9.9 to 4.9; p=0.49 | |  |  |
| **Adverse events (duration of trial)** | | **Number of events** | |  | |
| Baldassarri 2019 (70), USA [RCT] | Total number of side effects  Abnormal dreams  Anxiety  Fatigue  Headache  Insomnia  Nausea  Palpitations  Pruritus  Cough  Shortness of breath  Sore throat  Increased appetite | 28  4  0  1  1  1  2  2  1  7  2  4  3 | 21  3  1  1  0  2  0  0  0  5  0  5  4 | A participant may have reported more than one AE. | |

A eGo cig style e-cig (2^nd^ generation). Use of the e-cig as a substitute for cigarette smoking was encouraged but not considered mandatory and was at the discretion of study subjects.

B Standard care consisted of nicotine patch (those who smoked <10 cigarettes/day (14 mg patch), those who smoke >10 cigarettes/day (21 mg patch)) and intensive counselling sessions with an Advance Practice Registered Nurse behavioural tobacco treatment specialist or a clinical psychologist trained in motivational interviewing techniques and tobacco dependence pharmacotherapy.

† Values in Italics were calculated in RevMan.

‡ P-value differs in RevMan then what was reported in the study.

Abbreviations: AE= Adverse event; CI= Confidence interval; exCO= Exhaled carbon monoxide; SD= Standard deviation

### Forest plots

#### P.4.1 Outcome: Tobacco use abstinence


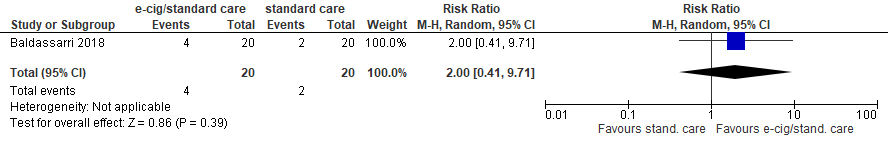


#### P.4.2 Outcome: Reduction in tobacco smoking frequency/quantity


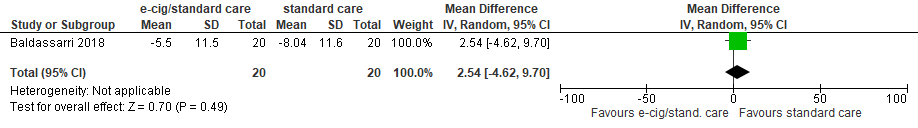


### ROB results

**Summary**

| **Author/Year** | **Sequence generation** | **Allocation Concealment** | **Blinding of Participants/ Personnel** | **Blinding of Outcome Assessors** | **Incomplete Outcome Data** | **Selective Outcome Reporting** | **Other** | **Overall ROB** |  |
| --- | --- | --- | --- | --- | --- | --- | --- | --- | --- |
| **tobacco use abstinence** | | | | | | | | | |
| ***7-day point prevalence confirmed by exCO*** | | | | | | | | | |
| Baldassarri 2019 (70) | Low | Unclear | Low | Low | Unclear | Unclear | Low | **Unclear risk** |  |
| **reduction in tobacco smoking frequency/quantity** | | | | | | | | | |
| ***Change in mean number of cigarettes smoked/day*** | | | | | | | | | |
| Baldassarri 2019 (70) | Low | Unclear | Low | Low | Unclear | Unclear | Low | **Unclear risk** |  |
| **adverse events** | | | | | | | | | |
| ***All adverse events*** | | | | | | | | | |
| Baldassarri 2019 (70) | Low | Unclear | Low | Low | Unclear | Unclear | Low | **Unclear risk** |  |

Abbreviations: exCO= Exhaled carbon monoxide

| **Baldassarri 2019**  **Outcomes:** Abstinence, Reduction, Adverse events | | |
| --- | --- | --- |
| **Risk of bias** | **Rating** | **Judgement support** |
| Sequence generation | Low risk | Random number generator with 1:1 blocked randomization (block size n=8) |
| Allocation Concealment | Unclear risk | No details about allocation concealment. |
| Blinding of Participants/ Personnel | All outcomes: Low risk | Participants and investigators were blinded. |
| Blinding of Outcome Assessors | All outcomes: Low risk | Abstinence confirmed by exCO <=6ppm (objective measure). Assuming that the investigators were the outcome assessors, who were blinded. |
| Incomplete Outcome Data | All outcomes: Unclear risk | 20% lost to follow-up (not provided per group). All participants lost to follow up were treated as smoking. |
| Selective Outcome Reporting | Unclear risk | No protocol noted; however when checking methods with what is reported, the outcomes of relapse and adverse events were not mentioned in the methods section. Although they were provided overall, but not by group. |
| Other | Low risk | No other concerns. |
| **Overall ROB** | **All outcomes: Unclear risk** | **Several domains unclear due to insufficient reporting.** |

Abbreviations: exCO= Exhaled carbon monoxide

## Appendix F.5. E-cigarette with nicotine vs E-cigarette with no nicotine

### Results table

| ***Study details*** | ***Outcome details*** | ***Timepoint*** | ***Categoria***  ***(7.2 mg)^A^*** | ***Categoria***  ***(7.2/5.4 mg)^B^*** | ***Categoria***  ***(0 mg/mL)^C^*** | ***Notes*** |
| --- | --- | --- | --- | --- | --- | --- |
| **Tobacco use abstinence** | | | **n/N (%)** | | |  |
| Caponnetto 2013 (67), Italy [RCT] | Quit rates (self-reported (not even a puff) together with an eCO of ≤7 ppm since the previous study visit) | 24 weeks | 12/100 (12%) | 10/100 (10%) | 5% (5/100) | ITT analysis. n/N calculated from % given.  e-cigs with nicotine vs e-cig with no nicotine p=0.04 at 52 weeks |
|  |  | 52 weeks | 13/100 (13%) | 9/100 (9%) | 4% (4/100) |  |
| **Reduction in tobacco smoking frequency/quantity** | | | **n/N (%)** | | |  |
| Caponnetto 2013 (67), Italy [RCT] | 50% or greater reduction in the number of cig/day since baseline (self-reported reduction) | 24 weeks | 17% (17/100) | 19% (19/100) | 15% (15/100) | ITT analysis. n/N calculated from % given. |
|  |  | 52 weeks | 10% (10/100) | 9% (9/100) | 12% (12/100) |  |
|  | 50% or greater reduction in the number of cig/day since baseline (self-reported reduction) | 52 weeks | 19*/200 (9.5%) | | 12/100 (12%); p=0.55 | Excluding quitters.  ITT analysis. *Typo in text, but received confirmation from author that table was correct. |
|  | 50% or greater reduction in the number of cig/day since baseline (self-reported reduction) | 52 weeks | 2/200 (1.0%) | | 2/100 (2%);  p=0.48 | Excluding quitters among heavy reducers (at least 80% reduction).  ITT analysis. |
| **Reduction in tobacco smoking frequency/quantity** | | | **Median (IQR)** | | |  |
| Caponnetto 2013(67), Italy [RCT] | Median (IQR) number of cig/day (self-reported reduction) | Baseline and 52 weeks | Baseline: 19.0 (14.0 to 25.0), 52-weeks: 12.0 (5.8 to 20.0); n=65 | Baseline: 21.0 (15.0 to 26.0), 52-weeks: 14.0 (6.0 to 20.0); n=63 | Baseline: 22.0 (15.0 to 27.0), 52-weeks: 12.0 (9.0 to 20.0); n=55 | Per-protocol evaluation. |
|  |  |  | *Pooled Mean (SD): 12.96 (10.69)^D^* | | *13.67 (8.37)^D^* |  |
|  | Median (IQR) number of cig/day (self-reported reduction) | Baseline and 52 weeks | Baseline: 19.0 (14.0 to 25.0), 52-weeks: 15.0 (10.0 to 20.0); n=52 | Baseline: 21.0 (15.0 to 26.0), 52-weeks: 15.0 (10.0 to 20.0); n=54 | Baseline: 22.0 (15.0 to 27.0), 52-weeks: 13.0 (10.0 to 20.0); n=51 | Per-protocol evaluation. Excluding quitters. |
|  |  |  | *Pooled Mean (SD): 15 (7.62)^D^* | | *14.33 (7.63)^D^* |  |
|  | measured by eCO levels through a portable device^E^ | Baseline and 52 weeks | Baseline: 19.0 ppm, 52-weeks: 15.0 ppm (8.8 to 29.0); n=65 | Baseline: 22.0 ppm,  52-weeks: 16.0 ppm (10.0 to 26.5); n=63 | Baseline: 19.5 ppm,  52-weeks: 17.0 ppm (11.3 to 25.0); n=55 | Per-protocol evaluation. Baseline results extracted from Figure 5. |
|  |  |  | *Pooled Mean (SD): 17.55 (13.94) ^D^* | | *17.77 (10.43) ^D^* |  |
|  | measured by eCO levels through a portable device^E^ | Baseline and 52 weeks | Baseline: 19.0 ppm, 52-weeks: 15.0 ppm (8.8 to 29.0); n=52 | Baseline: 22.0 ppm, 52-weeks: 18.0 ppm (10.0 to 26.0); n=54 | Baseline: 19.5 ppm,  52-weeks: 19.0 ppm (13.0 to 28.0); n=51 | Excluding quitters. Baseline results extracted from Figure 5. Per-protocol evaluation. |
|  |  |  | *Pooled Mean (SD): 17.80 (13.76) ^D^* | | *20 (11.44) ^D^* |  |
| **Adverse events** | | | **n/N or %** | | |  |
| Caponnetto 2013(67), Italy [RCT] | Serious AEs^F^ | 52 weeks | 0/100 | 0/100 | 0/100 |  |
|  | Hunger  Insomnia  Irritability  Anxiety  Depression | 52 weeks | 6%  3%  3%  3%  2% | 6%  4%  3%  3%  2% | 7%  5%  4%  3%  2% | Labeled as side effects |
|  | Dry cough  Mouth irritation  Shortness of breath  Throat irritation  Headache | 12 weeks; 52 weeks^G^ | 13%; 13%  8%; 8%  2%; 8%  17%; 12%  5%; 3% | 7%; 13%  7%; 13%  3%; 5%  15%; 16%  3%; 2% | 9%; 11%  10%; 13%  1%; 5%  9%; 9%  2%; 2% | The most frequently reported AEs before using e-cigarettes. Reporting from AE page of study diary. |
| **Possible adverse outcomes** | | | **Percent (95% CI)** | | |  |
| Russo 2016 (68), Italy [RCT] | Change in weight from baseline | 12 weeks | 0.93  (0.09 to 1.87) | 1.54  (0.41 to 2.87) | 0.76  (-0.24 to 1.77) | Authors presented results as a percent of baseline, however these have been converted for presentation as percent increase or decrease from baseline weight. |
|  |  | 24 weeks | 0.94  (-0.10 to 2.01) | 1.64  (0.29 to 2.96) | 0.28  (-1.05 to 1.47) |  |
|  |  | 52 weeks | 0.99  (-0.25 to 2.22) | 0.62  (-0.71 to 1.84) | 0.30  (-1.37 to 1.85) |  |

A Categoria Original 7.2 mg (2.27 +/- 0.13% nicotine) for 12 weeks

B Categoria 7.2 mg for six weeks and 5.4 mg for six weeks

C Categoria 0 mg/mL for 12 weeks

D We have ignored the baseline data (because of randomization) and calculated the mean (SD) based on the data at 52 weeks. We then merged the two intervention groups and calculated a pooled mean and pooled SD. Numbers converted from Median (IQR) to Mean (SD) using Want et al. 2014 [1] and pooled mean and pooled SD calculated at: <https://home.ubalt.edu/ntsbarsh/Business-stat/otherapplets/Pooled.htm> (accessed November 18th, 2019)

E Micro CO, Micro Medical Ltd, UK

F Including major depression, abnormal behaviour or any event requiring unscheduled visit to the family practitioner or hospitalization

G Data at week 12 was provided by study authors. Data at week 52 was extracted manually from graphs. Results also available at week 2, 4, 6, 8, 10 and 24.

[1] Wan X, Wang W, Liu J, Tong T. Estimating the sample mean and standard deviation from the sample size, median, range and/or interquartile range. *BMC medical research methodology* 2014; 14(1):135.

Abbreviations: AE= Adverse event; CI= Confidence interval; eCO= Exhaled carbon monoxide; IQR= interquartile range; ITT= Intention-to-treat; SD= Standard deviation

### Forest plots

#### P.5.1 Outcome: Tobacco use abstinence


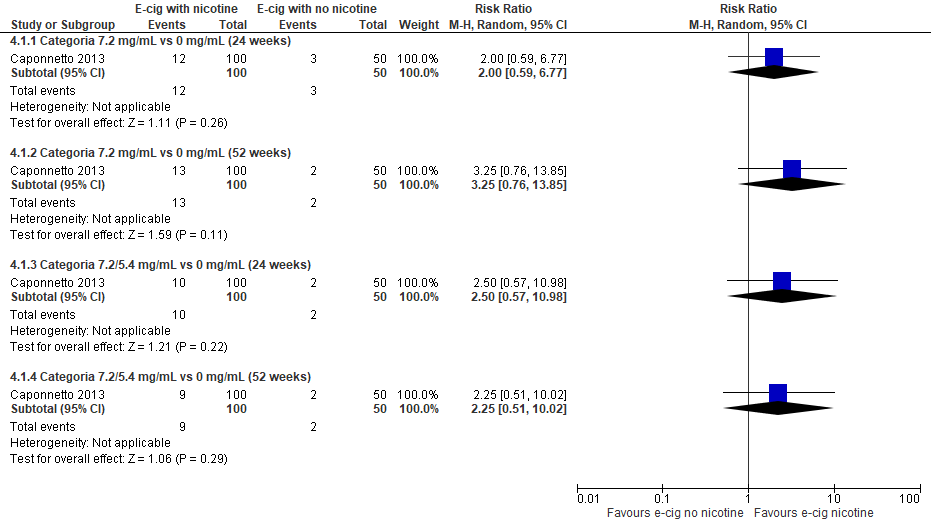


#### P.5.2 Outcome: Reduction: ≥50% reduction in the number of cigarettes/day


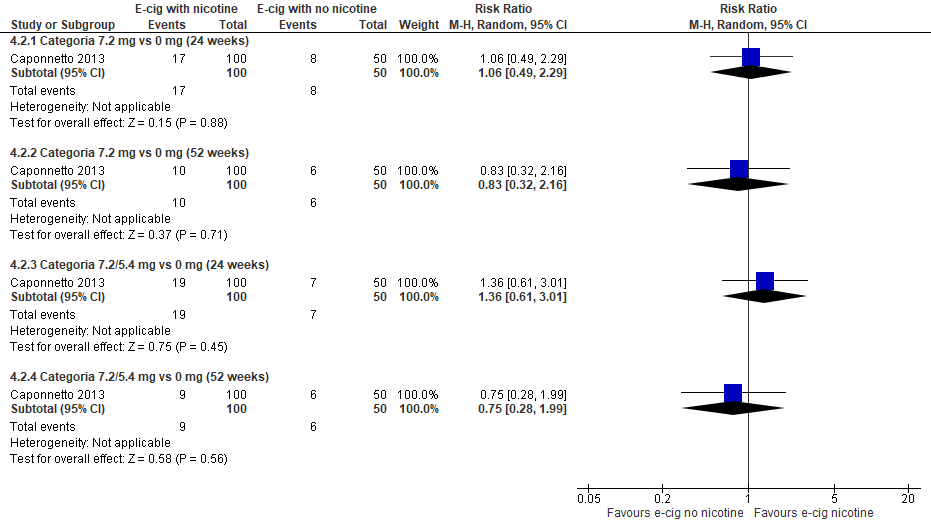


#### P.5.3 Outcome: Reduction: ≥50% reduction in the number of cigarettes/day [per-protocol analysis]


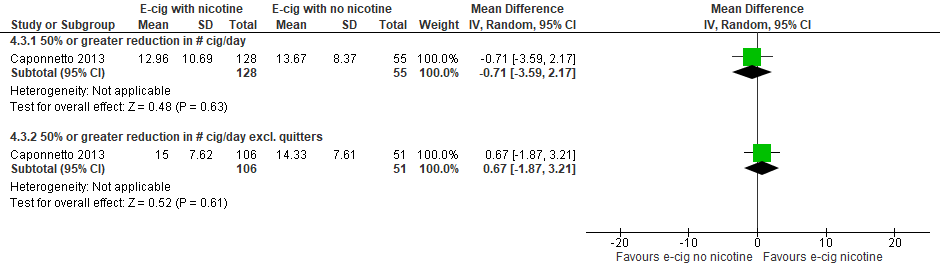


#### P.5.4 Outcome: eCO levels [per-protocol analysis]


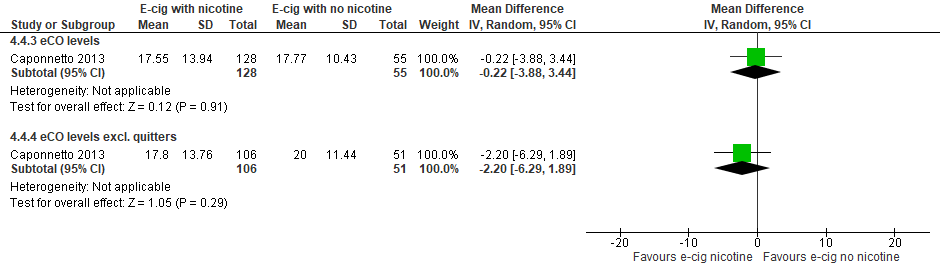


### ROB results

**Summary**

| **Author/Year** | **Sequence generation** | **Allocation Concealment** | **Blinding of Participants/ Personnel** | **Blinding of Outcome Assessors** | **Incomplete Outcome Data** | **Selective Outcome Reporting** | **Other** | **Overall ROB** |
| --- | --- | --- | --- | --- | --- | --- | --- | --- |
| **tobacco use abstinence** | | | | | | | | |
| ***Quit rates*** | | | | | | | | |
| Caponnetto 2013 (67) | Low | Unclear | Low | Low | Low | Low | Low | **Unclear risk** |
| **reduction in tobacco smoking frequency/quantity** | | | | | | | | |
| ***50% or greater reduction in the number of cig/day since baseline, eCO levels (ITT analysis)*** | | | | | | | | |
| Caponnetto 2013 (67) | Low | Unclear | Low | Low | Low | Low | Low | **Unclear risk** |
| ***50% or greater reduction in the number of cig/day since baseline, eCO levels (per protocol analysis)*** | | | | | | | | |
| Caponnetto 2013 (67) | Low | Unclear | Low | Low | High | Low | Low | **High risk** |
| **adverse events** | | | | | | | | |
| ***All adverse events*** | | | | | | | | |
| Caponnetto 2013 (67) | Low | Unclear | Low | Low | Low | Low | Low | **Unclear risk** |
| **possible adverse outcomes** | | | | | | | | |
| ***Change in weight from baseline*** | | | | | | | | |
| Russo 2016 (68) | Low | Unclear | Low | Low | Unclear | Low | Low | **Unclear risk** |

Abbreviations: eCO= Exhaled carbon monoxide; ITT= Intention-to-treat

| **Caponnetto 2013**  **Outcomes:** Abstinence, Reduction, Adverse events | | |
| --- | --- | --- |
| **Risk of bias** | **Rating** | **Judgement support** |
| Sequence generation | Low risk | The randomization sequence was computer generated by using block size of 15 with an allocation ratio of 5:5:5 for each of the three study conditions. |
| Allocation Concealment | Unclear risk | No details about allocation concealment. |
| Blinding of Participants/ Personnel | All outcomes: Low risk | Blinding was ensured by the identical external appearance of the cartridges. The hospital pharmacy was in charge of randomization and packaging of the cartridges. |
| Blinding of Outcome Assessors | All outcomes: Low risk | Blinding was ensured by the identical external appearance of the cartridges. |
| Incomplete Outcome Data | All outcomes with ITT analysis: Low risk | 61% of participants returned for the final follow-up, however, all participants were included in the analysis (ITT). |
|  | All outcomes with per protocol analysis: High risk | Large loss to follow-up (39%) in per protocol analysis. |
| Selective Outcome Reporting | Low risk | Seems to report all outcomes detailed in the clinical trial registry (NCT01164072) |
| Other | Low risk | RP has served as a consultant for Pfizer and Arbi Group Srl, the distributor of the Categoria e-cigarette. Although there may be a conflict of interest, since both groups were given the same e-cigarette brand and the only difference was the dose of nicotine, this was thought to be of little concern. |
| **Overall ROB** | **Outcomes: all outcomes with ITT analysis: Unclear risk** | **No details about allocation concealment.** |
|  | **Outcomes: all outcomes with per protocol analysis: High risk** | **No details about allocation concealment, and high loss to follow-up in per protocol analysis.** |

Abbreviations: ITT= Intention-to-treat

| **Russo 2016**  **Outcomes:** Change in weight from baseline | | |
| --- | --- | --- |
| **Risk of bias** | **Rating** | **Judgement support** |
| Sequence generation | Low risk | The randomization sequence was computer generated by using block size of 15 with an allocation ratio of 5:5:5 for each of the three study conditions. |
| Allocation Concealment | Unclear risk | No information on allocation concealment. |
| Blinding of Participants/ Personnel | Low risk | Blinding was ensured by the identical external appearance of the cartridges. The hospital pharmacy was in charge of randomization and packaging of the cartridges. Additionally, weight was measured at the visit using a mechanical column scale. |
| Blinding of Outcome Assessors | Low risk | Blinding was ensured by the identical external appearance of the cartridges. Additionally, weight was measured at the visit using a mechanical column scale. |
| Incomplete Outcome Data | Unclear risk | 61% of participants returned for the final follow-up, and it is unclear how many participants contributed to the weight outcome at each follow-up visit. |
| Selective Outcome Reporting | Low risk | Seems to report all outcomes detailed in the clinical trial registry (NCT01164072). |
| Other | Low risk | RP has served as a consultant for Pfizer and Arbi Group Srl, the distributor of the Categoria e-cigarette. Although there may be a conflict of interest, since both groups were given the same e-cigarette brand and the only difference was the dose of nicotine, this was thought to be of little concern. |
| **Overall ROB** | **Unclear risk** | **No details about allocation concealment, and it is unclear how many participants contributed to the weight change outcome at each time-point.** |

## Appendix F.6. E-cigarette with nicotine + other smoking cessation treatment (behavioural support) vs E-cigarette with no nicotine + other smoking cessation treatment (behavioural support)

### Results table

| ***Study details*** | ***Outcome details*** | ***Timepoint*** | ***E-cigarette^A^ + Behavioural Support^B^*** | ***E-cigarette^C^ (0 mg/mL) + Behavioural Support^B^*** | ***Notes*** |
| --- | --- | --- | --- | --- | --- |
| **Tobacco use abstinence** | | | **n/N (%)** | |  |
| Bullen 2013 (72), New Zealand [RCT] | Continuous smoking abstinence (self-reported, allowing ≤5 cigarettes in total, eCO^D^ of <10ppm) | 6 months | 21/289 (7.3%) | 3/73 (4.1%) | ITT analysis. |
|  | Continuous smoking abstinence (self-reported, allowing ≤5 cigarettes in total, eCO^D^ of <10ppm) | 6 months | 21/241 (8.7%) | 3/57 (5.3%) | Complete case analysis. |
|  | Continuous smoking abstinence (self-reported, allowing ≤5 cigarettes in total, eCO^D^ of <10ppm) | 6 months | 21/231 (9.1%) | 3/54 (5.6%) | Per protocol analysis excluding pregnancy, death, quitters who did not have biochemical verification at 6  months, undisclosed medication ineligibility, withdrew, and lost to follow-up at 6 months. |
|  | Continuous smoking abstinence (self-reported, allowing ≤5 cigarettes in total, eCO^D^ of <10ppm) | 6 months | 20/211 (9.5%) | 2/46 (4.3%) | Per protocol analysis excluding pregnancy, death, quitters who did not have biochemical verification at 6  months, undisclosed medication ineligibility, withdrew, lost to follow-up at 6 months. cross-overs, use of other or combined NRT products, and use of non-NRT |
|  | Continuous smoking abstinence (self-reported, allowing ≤5 cigarettes in total, eCO^D^ of <10ppm) | 6 months | 12/147 (8.2%) | 1/30 (3.3%) | Per protocol analysis excluding pregnancy, death, quitters who did not have biochemical verification at 6  months, undisclosed medication ineligibility, withdrew, lost to follow-up at 6 months. cross-overs, and use of other or combined NRT products. Participants must also still be using product to which they were randomized at 6 months. |
|  | 7-day point prevalence abstinence (self-reported no smoking of tobacco cigarettes in the past 7 days) | 6 months | 61/289 (21.1%) | 16/73 (21.9%) | ITT analysis. |
| Lucchiari 2020 (75)**,**Italy [RCT] | Continuous smoking abstinence (self-reported, eCO^D^ verified ≤7ppm) | 6 months | 13/70 (18.6%) | 11/70 (15.7%) | ITT analysis. |
| **Reduction in tobacco smoking frequency/quantity** | | | **n/N (%)** | |  |
| Bullen 2013 (72), New Zealand [RCT] | Proportion of participants who reduced daily cigarettes by 50% or greater | 6 months | 165/289 (57%) | 33/73 (45%) | n/N calculated from % given. |
| **Reduction in tobacco smoking frequency/quantity** | | | **Mean (SD or SE)** | |  |
| Eisenberg 2020 (73), Canada [RCT] | Change in mean number of daily cigarettes smoked since baseline (self-reported) | 24 weeks | -10.7 (NR) | -9.1 (NR) |  |
| Lucchiari 2020 (75)**,**Italy [RCT] | Number of daily cigarettes smoked | 6 months | 11.01 (SD=6.51) | 14.03 (SD=7.92) |  |
|  | eCO^D^ | 6 months | 12.01 (SD=8.13) | 15.28 (SD=11.43) |  |
| **Adverse events** | | | **n/N (%); number of events** | |  |
| Bullen 2013 (72), New Zealand [RCT] | Participants with at least one event | 6 months | 107/289 (37.0%) | 26/73 (35.6%) | Adverse events  were defined according to international guidelines and categorized by a masked researcher as related or unrelated to the intervention. |
|  | Total events | 6 months | 137 | 36 |  |
|  | Serious AEs^E^ | 6 months | 27/137 (19.7%) | 5/36 (13.9%) |  |
|  | Any non-serious event | 6 months | 110/137 (80.3%) | 31/36 (86.1%) |  |
|  | Events definitely related to study treatment  Events probably related to study treatment  Events possibly related to study treatment  Events unrelated to study treatment | 6 months | 0  1/137 (0.7%)  5/137 (3.6%)  131/137 (95.6%) | 0  1/36 (2.8%)  1/36 (2.8%)  34/36 (94.4%) |  |
| Eisenberg 2020 (73), Canada [RCT] | Serious AEs^F^ (self-reported) | 12 weeks | 1/128 (0.8%) | 4/127 (3.1%) | Only the first event for each participant in each category was counted. |
|  |  | 12 weeks to 24 weeks | 2/128 (1.6%) | 2/127 (1.6%) |  |
|  | Mild AEs^G^ (self-reported) | 12 weeks | 120/128 (93.8%) | 118/127 (92.9%) |  |
| Lucchiari 2020 (75)**,**Italy [RCT] | Side effects likely to be related to e-cig use:  Burning throat  Cough  Nausea  Headache  Insomnia  Stomachache  Confusion | 3 months | 5.7%  10%  1.4%  NR  1.4%  NR  1.4% | 2.9%  2.9%  NR  NR  NR  NR  NR | Self-reported through checklist of symptoms likely to be related to e-cigarettes. |
|  | Side effects likely to be related to e-cig use:  Burning throat  Cough  Nausea  Headache  Insomnia  Stomachache  Confusion | 6 months | 15.9%  5.8%  5.8%  NR  1.4%  4.3%  1.4% | 5.6%  2.8%  7%  1.4%  NR  4.2%  NR |  |

A Bullen 2013: Elusion e-cigarettes labelled 16 mg (tested 10-16 mg nicotine per mL) for 1 week before participants chosen quit day until 12 weeks after their chosen quit day; Eisenberg 2020: e-Cigarettes were purchased from purchased from NJOY Inc, Scottsdale, Arizona (tobacco-flavored liquid cartridges, 15 mg/mL); Lucchiari 2020: VP5 e-cigarette kit with 10 mL liquid cartridges (8 mg/mL)

B Bullen 2013: All participants referred to Quitline (low intensity behavioural support via voluntary telephone counselling); Eisenberg 2020: Participants received individual smoking cessation and relapse prevention counseling from trained research personnel at baseline, telephone follow-ups and clinic visits; Lucchiari 2020: All participants received low-intensity counseling delivered by phone (cognitive/behavioural intervention supporting smoking awareness and motivation to quit)

C Bullen 2013: Elusion e-cigarettes with 0 mg for 1 week before participants chosen quit day until 12 weeks after their chosen quit day; Eisenberg 2020: e-Cigarettes were purchased from purchased from NJOY Inc, Scottsdale, Arizona (tobacco-flavored liquid cartridges, 0 mg/mL); Lucchiari 2020: VP5 e-cigarette kit with 10 mL liquid cartridges (0 mg/mL)

D Measured with Bedfont Micro Smokerlyzers (Bedfont Scientific, Maidstone, UK)

E Including death, life threatening illness, admission to hospital or prolongation of hospital stay persistent or significant disability or incapacity, congenital abnormality, or other medically important events

F Serious AEs were adjudicated by an end points evaluation committee and include death, respiratory, cardiovascular, neuropsychiatric or other events

G Including cough, dry mouth, headache, rhinitis, throat irritation, dyspnea, sore throat, light headedness, dizziness, mouth irritation, nausea, indigestion, mouth ulcers, or vertigo

H Beck Depression Inventory-II: Possible scores range between 0 and 63, with higher scores indicating greater depressive symptoms

Abbreviations: AE= Adverse event; BDI-II=Beck Depression Inventory-II; eCO= Exhaled carbon monoxide; ITT= Intention-to-treat; NRT= Nicotine replacement therapy; SE= Standard error

### ROB results

**Summary**

| **Author/Year** | **Sequence generation** | **Allocation Concealment** | **Blinding of Participants/ Personnel** | **Blinding of Outcome Assessors** | **Incomplete Outcome Data** | **Selective Outcome Reporting** | **Other** | **Overall ROB** |
| --- | --- | --- | --- | --- | --- | --- | --- | --- |
| **tobacco use abstinence** | | | | | | | | |
| ***Continuous smoking abstinence (ITT analysis)*** | | | | | | | | |
| Bullen 2013 (72) | Low | Low | Low | Low | Low | Low | Low | **Low risk** |
| Lucchiari 2020 (75) | Low | Unclear | Low | Unclear | High | High | Low | **High risk** |
| ***Continuous smoking abstinence (per protocol analysis)*** | | | | | | | | |
| Bullen 2013 (72) | Low | Low | Low | Low | Low | Low | Low | **Low risk** |
| ***7-day point prevalence abstinence*** | | | | | | | | |
| Bullen 2013 (72) | Low | Low | Low | Low | Low | Low | Low | **Low risk** |
| **reduction in tobacco smoking frequency/quantity** | | | | | | | | |
| ***50% or greater reduction in the number of cig/day since baseline*** | | | | | | | | |
| Bullen 2013 (72) | Low | Low | Low | Low | Low | Low | Low | **Low risk** |
| ***Change in the mean number of cig/day since baseline*** | | | | | | | | |
| Eisenberg 2020 (73) | Low | Low | Low | Unclear | Low | Low | Low | **Unclear** |
| ***Number of daily cigarettes smoked, eCO*** | | | | | | | | |
| Lucchiari 2020 (75) | Low | Unclear | Low | Unclear | High | High | Low | **High risk** |
| **adverse events** | | | | | | | | |
| ***All serious and non-serious AEs*** | | | | | | | | |
| Bullen 2013 (72) | Low | Low | Low | Low | Low | Low | Low | **Low risk** |
| Eisenberg 2020 (73) | Low | Low | Low | Unclear | Low | Low | Low | **Low risk** |
| Lucchiari 2020 (75) | Low | Unclear | Low | Unclear | High | High | Low | **High risk** |

Abbreviations: AE= Adverse event; eCO= Exhaled carbon monoxide; ITT= Intention-to-treat

| **Bullen 2013**  **Outcomes:** Abstinence, Reduction, All serious and non-serious AEs | | |
| --- | --- | --- |
| **Risk of bias** | **Rating** | **Judgement support** |
| Sequence generation | Low risk | The randomization sequence was computer generated using a block size of 9 with an allocation ratio of 4:4:1 for each of the three study conditions, with participants stratified by ethnicity, sex and level of nicotine dependence. |
| Allocation Concealment | Low risk | Allocation sequence was generated in advance by a statistician. The trial protocol stated that the sequence was concealed from research assistants enrolling participants. |
| Blinding of Participants/ Personnel | All outcomes: Low risk | Blinding was ensured by the identical appearance of the nicotine e-cigarettes and nicotine-free e-cigarettes. Cartridge labels were also masked to nicotine content. |
| Blinding of Outcome Assessors | All outcomes: Low risk | Researchers assessing outcomes used a list generated by the trial database that concealed treatment allocation. |
| Incomplete Outcome Data | All outcomes: Low risk | Overall loss to follow-up was high (22%), with 17% in the nicotine e-cigarette group and 22% in the non-nicotine e-cigarette group. Authors conducted both ITT (with all missing assumed smoking) and per protocol analyses with similar results. |
| Selective Outcome Reporting | Low risk | Outcomes reported appear to be consistent with all outcomes detailed in the clinical trial registry (ACTRN12610000866000) and study protocol. |
| Other | Low risk | No other concerns. |
| **Overall ROB** | **All outcomes: Low risk** | **All domains rated as having few concerns with risk of bias.** |

Abbreviations: ITT= Intention-to-treat

| **Eisenberg 2020**  **Outcomes:** Reduction, All serious and non-serious AEs | | |
| --- | --- | --- |
| **Risk of bias** | **Rating** | **Judgement support** |
| Sequence generation | Low risk | The randomization sequence was computer generated using permuted blocks of 6 and 9, stratified by centre. |
| Allocation Concealment | Low risk | Study did not report on allocation concealment method, but risk of bias judged to be low due to randomization with an online centralized system. |
| Blinding of Participants/ Personnel | All outcomes: Low risk | For the e-cigarette groups, blinding was ensured by the identical appearance of the nicotine e-cigarettes and nicotine-free e-cigarettes. |
| Blinding of Outcome Assessors | All outcomes: Unclear risk | No details about blinding of the outcome assessors. |
| Incomplete Outcome Data | All outcomes: Low risk | 70 participants lost to follow-up (18.6%), with higher rates of loss in the counseling-alone group (29.8%). Sensitivity analyses were performed to examine the effect of missing data (complete case analysis and multiple imputation to impute missing reduction data) and results found to be similar. |
| Selective Outcome Reporting | Low risk | Outcomes reported appear to be consistent with all outcomes detailed in the study protocol. |
| Other | Low risk | No other concerns. |
| **Overall ROB** | **Outcomes: Unclear risk** |  |

| **Lucchiari 2020**  **Outcomes:** Abstinence, Reduction, Side effects, | | |
| --- | --- | --- |
| **Risk of bias** | **Rating** | **Judgement support** |
| Sequence generation | Low risk | The randomization sequence was prepared by independent personnel using a permuted block design. |
| Allocation Concealment | Unclear risk | No details provided on how allocation was concealed. However, a central pharmacy was responsible for assigning, allocating, and delivering e-cigarette liquids. |
| Blinding of Participants/ Personnel | All outcomes: Low risk | Participants and researchers were blinded to allocation status, ensured through masked nicotine content in the liquid for the e-cigarettes kit. |
| Blinding of Outcome Assessors | All outcomes: Unclear risk | No details provided on blinding of outcome assessors. |
| Incomplete Outcome Data | All outcomes: High risk | High amount of loss to follow up (26%). ITT analysis performed, but strategy for handling missing data was not reported. |
|  | Outcome: Severe AEs: High risk | Unclear how data on severe AEs were collected. |
| Selective Outcome Reporting | High risk | Primary pulmonary and smoking cessation outcomes consistent with the trial registry (NCT02422914). Activity and lifestyle outcomes registered were not reported. Outcomes at 12 months also not reported. |
| Other | Low risk | No other concerns. |
| **Overall ROB** | **All outcomes: High risk** | **High risk of bias due to not reporting some outcomes that were listed in the registry and high loss to follow-up. Additionally, unclear reporting of allocation concealment and blinding of outcome assessment.** |

Abbreviations: AE = Adverse event

## Appendix F.7. E-cigarette with nicotine + other smoking cessation treatment (behavioural support) vs no intervention + other smoking cessation treatment (behavioural support)

### Results table

| ***Study details*** | ***Outcome details*** | ***Timepoint*** | ***E-cigarette ^A^ + Behavioural Support^B^*** | ***No intervention + Behavioural Support^B^*** | ***Notes*** |
| --- | --- | --- | --- | --- | --- |
| **Tobacco use abstinence** | | | **n/N (%)** | |  |
| Lucchiari 2020 (75)**,**Italy [RCT] | Continuous smoking abstinence (self-reported, eCO^C^ verified ≤7ppm) | 6 months | 13/70 (18.6%) | 7/70 (10.0%) | ITT analysis. |
| **Reduction in tobacco smoking frequency/quantity** | | | **Mean (SD)** | |  |
| Eisenberg 2020 (73), Canada [RCT] | Change in mean number of daily cigarettes smoked since baseline (self-reported) | 24 weeks | -10.7 (NR) | -5.5 (NR) |  |
| Lucchiari 2020 (75)**,**Italy [RCT] | Number of daily cigarettes smoked | 6 months | 11.01 (SD=6.51) | 13.45 (SD=6.49) |  |
|  | eCO^C^ | 6 months | 12.01 (SD=8.13) | 16.52 (SD=10.24) |  |
| **Adverse events** | | | **n/N (%)** | |  |
| Eisenberg 2020 (73), Canada [RCT] | Serious AEs^D^ (self-reported) | 12 weeks | 1/128 (0.8%) | 2/121 (1.7%) | Only the first event for each participant in each category was counted. |
|  |  | 12 weeks to 24 weeks | 2/128 (1.6%) | 2/121 (1.7%) |  |
|  | Mild AEs^E^ (self-reported) | 12 weeks | 120/128 (94%) | 88/121 (73%) |  |

A Eisenberg 2020: e-Cigarettes were purchased from purchased from NJOY Inc, Scottsdale, Arizona (tobacco-flavored liquid cartridges, 15 mg/mL); Lucchiari 2020: VP5 e-cigarette kit with 10 mL liquid cartridges (8 mg/mL)

B Eisenberg 2020: Participants received individual smoking cessation and relapse prevention counseling from trained research personnel at baseline, telephone follow-ups and clinic visits; Lucchiari 2020: All participants received low-intensity counseling delivered by phone (cognitive/behavioural intervention supporting smoking awareness and motivation to quit)

C Measured with Bedfont Micro Smokerlyzers (Bedfont Scientific, Maidstone, UK)

D Serious AEs were adjudicated by an end points evaluation committee and include death, respiratory, cardiovascular, neuropsychiatric or other events

E Including cough, dry mouth, headache, rhinitis, throat irritation, dyspnea, sore throat, light headedness, dizziness, mouth irritation, nausea, indigestion, mouth ulcers, or vertigo

Abbreviations: AE= Adverse event; eCO= Exhaled carbon monoxide; ITT= Intention-to-treat; SE= Standard error

### ROB results

**Summary**

| **Author/Year** | **Sequence generation** | **Allocation Concealment** | **Blinding of Participants/ Personnel** | **Blinding of Outcome Assessors** | **Incomplete Outcome Data** | **Selective Outcome Reporting** | **Other** | **Overall ROB** |
| --- | --- | --- | --- | --- | --- | --- | --- | --- |
| **tobacco use abstinence** | | | | | | | | |
| ***Continuous smoking abstinence*** | | | | | | | | |
| Lucchiari 2020 (75) | Low | Unclear | Low | Unclear | High | High | Low | **High risk** |
| **reduction in tobacco smoking frequency/quantity** | | | | | | | | |
| ***Change in the mean number of cig/day since baseline*** | | | | | | | | |
| Eisenberg 2020 (73) | Low | Low | High | Unclear | Low | Low | Low | **High risk** |
| ***Number of daily cigarettes smoked, eCO*** | | | | | | | | |
| Lucchiari 2020 (75) | Low | Unclear | Low | Unclear | High | High | Low | **High risk** |
| **adverse events** | | | | | | | | |
| ***All serious and non-serious AEs*** | | | | | | | | |
| Eisenberg 2020 (73) | Low | Low | High | Unclear | Low | Low | Low | **High risk** |

Abbreviations: AE= Adverse event; eCO= Exhaled carbon monoxide; ITT= Intention-to-treat

| **Eisenberg 2020**  **Outcomes:** Reduction, All serious and non-serious AEs | | |
| --- | --- | --- |
| **Risk of bias** | **Rating** | **Judgement support** |
| Sequence generation | Low risk | The randomization sequence was computer generated using permuted blocks of 6 and 9, stratified by centre. |
| Allocation Concealment | Low risk | Study did not report on allocation concealment method, but risk of bias judged to be low due to randomization with an online centralized system. |
| Blinding of Participants/ Personnel | All outcomes: High risk | For the e-cigarette groups, blinding was ensured by the identical appearance of the nicotine e-cigarettes and nicotine-free e-cigarettes. Those in the counselling-alone group were not blinded. Knowledge of assignment group could influence reporting. |
| Blinding of Outcome Assessors | All outcomes: Unclear risk | No details about blinding of the outcome assessors. |
| Incomplete Outcome Data | All outcomes: Low risk | 70 participants lost to follow-up (18.6%), with higher rates of loss in the counseling-alone group (29.8%). Sensitivity analyses were performed to examine the effect of missing data (complete case analysis and multiple imputation to impute missing reduction data) and results found to be similar. |
| Selective Outcome Reporting | Low risk | Outcomes reported appear to be consistent with all outcomes detailed in the study protocol. |
| Other | Low risk | No other concerns. |
| **Overall ROB** | **Outcomes: High risk** |  |

| **Lucchiari 2020**  **Outcomes:** Abstinence, Reduction, Side effects | | |
| --- | --- | --- |
| **Risk of bias** | **Rating** | **Judgement support** |
| Sequence generation | Low risk | The randomization sequence was prepared by independent personnel using a permuted block design. |
| Allocation Concealment | Unclear risk | No details provided on how allocation was concealed. However, a central pharmacy was responsible for assigning, allocating, and delivering e-cigarette liquids. |
| Blinding of Participants/ Personnel | All outcomes: Low risk | Participants and researchers were blinded to allocation status, ensured through masked nicotine content in the liquid for the e-cigarettes kit. |
| Blinding of Outcome Assessors | All outcomes: Unclear risk | No details provided on blinding of outcome assessors. |
| Incomplete Outcome Data | All outcomes: High risk | High amount of loss to follow up (26%). ITT analysis performed, but strategy for handling missing data was not reported. |
| Selective Outcome Reporting | High risk | Primary pulmonary and smoking cessation outcomes consistent with the trial registry (NCT02422914). Activity and lifestyle outcomes registered were not reported. Outcomes at 12 months also not reported. |
| Other | Low risk | No other concerns. |
| **Overall ROB** | **All outcomes: High risk** | **High risk of bias due to not reporting some outcomes that were listed in the registry and high loss to follow-up. Additionally, unclear reporting of allocation concealment and blinding of outcome assessment.** |

Abbreviations: AE = Adverse event

## Appendix F.8. E-cigarette with nicotine + other smoking cessation treatment (behavioural support) + standard care (nicotine patch) vs. E-cigarette with no nicotine + other smoking cessation treatment (behavioural support) + standard care (nicotine patch)

### Results table

| ***Study details*** | ***Outcome details*** | ***Timepoint*** | ***E-cigarette (18 mg/mL) ^A^ + Behavioural Support^B^ + Standard Care^C^*** | ***E-cigarette (0 mg/mL)^D^ + Behavioural Support^B^+ Standard Care^C^*** | ***Notes*** |
| --- | --- | --- | --- | --- | --- |
| **Tobacco use abstinence** | | | **n/N (%)** | |  |
| Walker 202o (76), New Zealand [RCT] | Continuous smoking abstinence (self-reported, allowing ≤5 cigarettes in total, eCO^E^ verified ≤9ppm) | 6 months | 35/500 (7.0%) | 20/499 (4.0%) | ITT analysis. |
|  | Continuous smoking abstinence (self-reported) | 6 months | 89/500 (17.8%) | 53/499 (10.6%) |  |
|  | 7-day point prevalence abstinence (self-reported) | 6 months | 119/500 (23.8%) | 83/499 (16.6%) |  |
| **Reduction in tobacco smoking frequency/quantity** | | | **Mean (SE) or n/N (%)** | |  |
| Walker 2020 (76), New Zealand [RCT] | Change from baseline in the mean number of cigarettes smoked per day | 6 months | 8.3 (0.5) | 8.3 (0.4) | Assessed in participants still smoking. |
|  | ≥50% reduction in the number of cigarettes/day since baseline | 6 months | 218/500 (44%) | 190/499 (38%) |  |
| **Adverse events** | | | **n/N (%); number of events** | |  |
| Walker 2020 (76), New Zealand [RCT] | Participants with a serious adverse event | 6 months | 16/500 (3.2%) | 22/499 (4.4%) | Serious adverse events were allocated ICD 10th edition Australian Modification codes  and were classified as non-serious or serious by a physician. |
|  | Total serious adverse events | 6 months | 18 | 27 |  |
|  | Death  Life threatening  Hospitalization  Persistent, significant disability or incapacity  Otherwise medically important | 6 months | 0  2  11  1  4 | 1  1  19  0  6 |  |
| **Possible adverse outcomes** | | | **Mean (SE) or n/N (%)** | | |
| Walker 2020 (76), New Zealand [RCT] | Change in BMI from baseline (self-reported) | 3 months  6 months | -0.4 (0.2)  -0.4 (0.2) | 0.1 (0.2)  -0.1 (0.2) |  |
|  | Change in weight from baseline (self-reported) | 3 months  6 months | -1.3 (0.6)  -1.1 (0.5) | 0.1 (0.6)  -0.4 (0.5) |  |
|  | Vivid dreams | 1 month  3 months  6 months | 34/500 (11%)  21/500 (7%)  12/500 (4%) | 29/499 (10%)  13/499 (5%)  11/499 (3%) |  |
|  | Itchiness | 1 month  3 months  6 months | 41/500 (13%)  25/500 (8%)  12/500 (4%) | 33/499 (11%)  20/499 (7%)  10/499 (3%) |  |
|  | Redness, swollen at patch site | 1 month  3 months  6 months | 36/500 (11%)  20/500 (6%)  10/500 (3%) | 30/499 (10%)  25/499 (9%)  11/499 (3%) |  |
|  | Dry mouth or throat | 1 month  3 months  6 months | 21/500 (7%)  14/500 (4%)  10/500 (3%) | 15/499 (5%)  13/499 (5%)  5/499 (2%) |  |
|  | Cough | 1 month  3 months  6 months | 19/500 (6%)  14/500 (4%)  15/500 (4%) | 9/499 (3%)  3/499 (1%)  4/499 (1%) |  |
|  | Nausea | 1 month  3 months  6 months | 15/500 (5%)  6/500 (2%)  6/500 (2%) | 11/499 (4%)  3/499 (1%)  4/499 (1%) |  |
|  | Headache | 1 month  3 months  6 months | 12/500 (4%)  5/500 (2%)  6/500 (2%) | 11/499 (4%)  8/499 (3%)  7/499 (2%) |  |

A e-Cigarettes were 2nd-generation eVOD purchased from Kangertech, Shenzhen GuangDong, China (e-liquid of choice, 18 mg/mL; nicotine content was laboratory tested with +/- 10% nicotine content considered acceptable)

B All participants were offered moderate-intensity withdrawal-oriented behavioural support, delivered weekly over the phone by researchers with standardized training

C Standard care consisted of 14 weeks of NRT treatment, delivered through a 24h Habitrol nicotine patch (21 mg)

D e-Cigarettes were 2nd-generation eVOD purchased from Kangertech, Shenzhen GuangDong, China (e-liquid of choice, 0 mg/mL)

E Measured with Bedfont Smokerlyzer (Bedfont Scientific Ltd, Kent, UK)

Abbreviations: AE= Adverse event; BMI=Body mass index; eCO= Exhaled carbon monoxide; ITT= Intention-to-treat; SE= Standard error

### ROB results

**Summary**

| **Author/Year** | **Sequence generation** | **Allocation Concealment** | **Blinding of Participants/ Personnel** | **Blinding of Outcome Assessors** | **Incomplete Outcome Data** | **Selective Outcome Reporting** | **Other** | **Overall ROB** |
| --- | --- | --- | --- | --- | --- | --- | --- | --- |
| **tobacco use abstinence** | | | | | | | | |
| ***Continuous smoking abstinence (eCO verified and self-reported), 7-day point prevalence abstinence*** | | | | | | | | |
| Walker 2020 (76) | Low | Low | Low | Low | Low | Low | Low | **Low risk** |
| **reduction in tobacco smoking frequency/quantity** | | | | | | | | |
| ***Change in the mean number of cig/day since baseline, ≥50% reduction in the number of cigarettes/day since baseline*** | | | | | | | | |
| Walker 2020 (76) | Low | Low | Low | Low | Low | Low | Low | **Low risk** |
| ***Number of daily cigarettes smoked, eCO*** | | | | | | | | |
| Walker 2020 (76) | Low | Low | Low | Low | Low | Low | Low | **Low risk** |
| **adverse events** | | | | | | | | |
| ***Serious AEs*** | | | | | | | | |
| Walker 2020 (76) | Low | Low | Low | Low | Low | Low | Low | **Low risk** |
| **possible adverse outcomes** | | | | | | | | |
| ***Change in BMI/weight, Side effects*** | | | | | | | | |
| Walker 2020 (76) | Low | Low | Low | Low | Low | Low | Low | **Low risk** |

Abbreviations: AE= Adverse event; eCO= Exhaled carbon monoxide

| **Walker 2020**  **Outcomes:** Abstinence, Reduction, Serious AEs, Change in BMI/weight, Side effects | | |
| --- | --- | --- |
| **Risk of bias** | **Rating** | **Judgement support** |
| Sequence generation | Low risk | The randomization sequence was computer generated using permuted blocks of 9. |
| Allocation Concealment | Low risk | Allocation concealment was ensured through a study statistician generating the sequence and randomly allocating participants by computer. |
| Blinding of Participants/ Personnel | All outcomes: Low risk | Participants and personnel were blinded to the contents of the e-liquid that they received. |
| Blinding of Outcome Assessors | All outcomes: Low risk | Outcome assessors were blinded to allocation status. |
| Incomplete Outcome Data | All outcomes: Low risk | Loss-to-follow-up was high (34% in the patches and support alone group and 30% in the e-cigarette groups). Authors report reasons for participant withdrawal from the study and examine differences in baseline characteristics between participants lost to follow-up and those retained. |
| Selective Outcome Reporting | Low risk | Reported secondary outcomes are consistent with trial registry (NCT02521662), with the exception of cost, to be presented in a separate publication. |
| Other | Low risk | No other concerns. |
| **Overall ROB** | **Low risk** | **All domains rated as having few concerns with risk of bias.** |

Abbreviations: BMI=Body mass index

For P.10 and P.11:

| **Walker 2020**  **Outcomes:** Abstinence, Reduction, Serious AEs, Change in BMI/weight, Side effects | | |
| --- | --- | --- |
| **Risk of bias** | **Rating** | **Judgement support** |
| Sequence generation | Low risk | The randomization sequence was computer generated using permuted blocks of 9. |
| Allocation Concealment | Low risk | Allocation concealment was ensured through a study statistician generating the sequence and randomly allocating participants by computer. |
| Blinding of Participants/ Personnel | All outcomes: High risk | Participants and personnel were blinded to the contents of the e-liquid that they received. Those in the patch and behavioural support intervention group were not blinded. Knowledge of assignment group could influence reporting. |
| Blinding of Outcome Assessors | All outcomes: Low risk | Outcome assessors were blinded to allocation status. |
| Incomplete Outcome Data | All outcomes: Low risk | Loss-to-follow-up was high (34% in the patches and support alone group and 30% in the e-cigarette groups). Authors report reasons for participant withdrawal from the study and examine differences in baseline characteristics between participants lost to follow-up and those retained. |
| Selective Outcome Reporting | Low risk | Reported secondary outcomes are consistent with trial registry (NCT02521662), with the exception of cost, to be presented in a separate publication. |
| Other | Low risk | No other concerns. |
| **Overall ROB** | **High risk** |  |

Abbreviations: BMI=Body mass index

## Appendix F.9. E-cigarette with nicotine + other smoking cessation treatment (behavioural support) + standard care (nicotine patch) vs. other smoking cessation treatment (behavioural support) + standard care (nicotine patch)

### Results table

| ***Study details*** | ***Outcome details*** | ***Timepoint*** | ***E-cigarette (18 mg/mL) ^A^ + Behavioural Support^B^ + Standard Care^C^*** | ***Behavioural Support^B^+ Standard Care^C^*** | ***Notes*** |
| --- | --- | --- | --- | --- | --- |
| **Tobacco use abstinence** | | | **n/N (%)** | |  |
| Walker 2020 (76), New Zealand [RCT] | Continuous smoking abstinence (self-reported, allowing ≤5 cigarettes in total, eCO^D^ verified ≤9ppm) | 6 months | 35/500 (7.0%) | 3/125 (2.4%) | ITT analysis. |
|  | Continuous smoking abstinence (self-reported) | 6 months | 89/500 (17.8%) | 10/125 (8%) |  |
|  | 7-day point prevalence abstinence (self-reported) | 6 months | 119/500 (23.8%) | 14/125 (11.2%) |  |
| **Reduction in tobacco smoking frequency/quantity** | | | **Mean (SE) or n/N (%)** | |  |
| Walker 2020 (76), New Zealand [RCT] | Change from baseline in the mean number of cigarettes smoked per day | 6 months | 8.3 (0.5) | 8.6 (1.0) | Assessed in participants still smoking. |
|  | Proportion of participants who reduced daily cigarettes by at least half | 6 months | 218/500 (44%) | 32/125 (26%) |  |
| **Adverse events** | | | **n/N (%); number of events** | |  |
| Walker 2020 (76), New Zealand [RCT] | Participants with a serious adverse event | 6 months | 16/500 (3.2%) | 3/125 (2.4%) | Serious adverse events were allocated ICD 10th edition Australian Modification codes  and were classified as non-serious or serious by a physician. |
|  | Total serious adverse events | 6 months | 18 | 4 |  |
|  | Death  Life threatening  Hospitalization  Persistent, significant disability or incapacity  Otherwise medically important | 6 months | 0  2  11  1  4 | 0  0  3  0  1 |  |
| **Possible adverse outcomes** | | | **Mean (SE) or n/N (%)** | | |
| Walker 2020 (76), New Zealand [RCT] | Change in BMI from baseline (self-reported) | 3 months  6 months | -0.4 (0.2)  -0.4 (0.2) | -0.2 (0.4)  0.1 (0.4) |  |
|  | Change in weight from baseline (self-reported) | 3 months  6 months | -1.3 (0.6)  -1.1 (0.5) | 0.1 (1.1)  -0.4 (1.0) |  |
|  | Vivid dreams | 1 month  3 months  6 months | 34/500 (11%)  21/500 (7%)  12/500 (4%) | 8/125 (15%)  7/125 (13%)  6/125 (10%) |  |
|  | Itchiness | 1 month  3 months  6 months | 41/500 (13%)  25/500 (8%)  12/500 (4%) | 6/125 (11%)  3/125 (6%)  2/125 (3%) |  |
|  | Redness, swollen at patch site | 1 month  3 months  6 months | 36/500 (11%)  20/500 (6%)  10/500 (3%) | 11/125 (21%)  6/125 (11%)  5/125 (8%) |  |
|  | Dry mouth or throat | 1 month  3 months  6 months | 21/500 (7%)  14/500 (4%)  10/500 (3%) | 0/125 (0%)  0/125 (0%)  0/125 (0%) |  |
|  | Cough | 1 month  3 months  6 months | 19/500 (6%)  14/500 (4%)  15/500 (4%) | 1/125 (2%)  0/125 (0%)  0/125 (0%) |  |
|  | Nausea | 1 month  3 months  6 months | 15/500 (5%)  6/500 (2%)  6/500 (2%) | 3/125 (6%)  1/125 (2%)  2/125 (3%) |  |
|  | Headache | 1 month  3 months  6 months | 12/500 (4%)  5/500 (2%)  6/500 (2%) | 2/125 (4%)  2/125 (4%)  1/125 (2%) |  |

A e-Cigarettes were 2nd-generation eVOD purchased from Kangertech, Shenzhen GuangDong, China (e-liquid of choice, 18 mg/mL; nicotine content was laboratory tested with +/- 10% nicotine content considered acceptable)

B All participants were offered moderate-intensity withdrawal-oriented behavioural support, delivered weekly over the phone by researchers with standardized training

C Standard care consisted of 14 weeks of NRT treatment, delivered through a 24h Habitrol nicotine patch (21 mg)

D Measured with Bedfont Smokerlyzer (Bedfont Scientific Ltd, Kent, UK)

Abbreviations: AE= Adverse event; BMI=Body mass index; eCO= Exhaled carbon monoxide; ITT= Intention-to-treat; SE= Standard error

## Appendix F.10. E-cigarette with no nicotine combined with other smoking cessation treatment (behavioural support) + standard care (nicotine patch) vs. other smoking cessation treatment (behavioural support) + standard care (nicotine patch)

### Results table

| ***Study details*** | ***Outcome details*** | ***Timepoint*** | ***E-cigarette (0 mg/mL)^A^ + Behavioural Support^B^ + Standard Care^C^*** | ***Behavioural Support^B^+ Standard Care^C^*** | ***Notes*** |
| --- | --- | --- | --- | --- | --- |
| **Tobacco use abstinence** | | | **n/N (%)** | |  |
| Walker 2020 (76), New Zealand [RCT] | Continuous smoking abstinence (self-reported, allowing ≤5 cigarettes in total, eCO^D^ verified ≤9ppm) | 6 months | 20/499 (4.0%) | 3/125 (2.4%) | ITT analysis. |
|  | Continuous smoking abstinence (self-reported) | 6 months | 53/499 (10.6%) | 10/125 (8%) |  |
|  | 7-day point prevalence abstinence (self-reported) | 6 months | 83/499 (16.6%) | 14/125 (11.2%) |  |
| **Reduction in tobacco smoking frequency/quantity** | | | **Mean (SE) or n/N (%)** | |  |
| Walker 2020 (76), New Zealand [RCT] | Change from baseline in the mean number of cigarettes smoked per day | 6 months | 8.3 (0.4) | 8.6 (1.0) | Assessed in participants still smoking. |
|  | Proportion of participants who reduced daily cigarettes by at least half | 6 months | 190/499 (38%) | 32/125 (26%) |  |
| **Adverse events** | | | **n/N (%); number of events** | |  |
| Walker 2020 (76), New Zealand [RCT] | Participants with a serious adverse event | 6 months | 22/499 (4.4%) | 3/125 (2.4%) | Serious adverse events were allocated ICD 10th edition Australian Modification codes  and were classified as non-serious or serious by a physician. |
|  | Total serious adverse events | 6 months | 27 | 4 |  |
|  | Death  Life threatening  Hospitalization  Persistent, significant disability or incapacity  Otherwise medically important | 6 months | 1  1  19  0  6 | 0  0  3  0  1 |  |
| **Possible adverse outcomes** | | | **Mean (SE) or n/N (%)** | | |
| Walker 2020 (76), New Zealand [RCT] | Change in BMI from baseline (self-reported) | 3 months  6 months | 0.1 (0.2)  -0.1 (0.2) | -0.2 (0.4)  0.1 (0.4) |  |
|  | Change in weight from baseline (self-reported) | 3 months  6 months | 0.1 (0.6)  -0.4 (0.5) | 0.1 (1.1)  -0.4 (1.0) |  |
|  | Vivid dreams | 1 month  3 months  6 months | 29/499 (10%)  13/499 (5%)  11/499 (3%) | 8/125 (15%)  7/125 (13%)  6/125 (10%) |  |
|  | Itchiness | 1 month  3 months  6 months | 33/499 (11%)  20/499 (7%)  10/499 (3%) | 6/125 (11%)  3/125 (6%)  2/125 (3%) |  |
|  | Redness, swollen at patch site | 1 month  3 months  6 months | 30/499 (10%)  25/499 (9%)  11/499 (3%) | 11/125 (21%)  6/125 (11%)  5/125 (8%) |  |
|  | Dry mouth or throat | 1 month  3 months  6 months | 15/499 (5%)  13/499 (5%)  5/499 (2%) | 0/125 (0%)  0/125 (0%)  0/125 (0%) |  |
|  | Cough | 1 month  3 months  6 months | 9/499 (3%)  3/499 (1%)  4/499 (1%) | 1/125 (2%)  0/125 (0%)  0/125 (0%) |  |
|  | Nausea | 1 month  3 months  6 months | 11/499 (4%)  3/499 (1%)  4/499 (1%) | 3/125 (6%)  1/125 (2%)  2/125 (3%) |  |
|  | Headache | 1 month  3 months  6 months | 11/499 (4%)  8/499 (3%)  7/499 (2%) | 2/125 (4%)  2/125 (4%)  1/125 (2%) |  |

A e-Cigarettes were 2nd-generation eVOD purchased from Kangertech, Shenzhen GuangDong, China (e-liquid of choice, 0 mg/mL)

B All participants were offered moderate-intensity withdrawal-oriented behavioural support, delivered weekly over the phone by researchers with standardized training

C Standard care consisted of 14 weeks of NRT treatment, delivered through a 24h Habitrol nicotine patch (21 mg)

D Measured with Bedfont Smokerlyzer (Bedfont Scientific Ltd, Kent, UK)

Abbreviations: AE= Adverse event; BMI=Body mass index; eCO= Exhaled carbon monoxide; ITT= Intention-to-treat; SE= Standard error

## Appendix F.11. E-cigarette with no nicotine combined with other smoking cessation treatment (behavioural support) vs no intervention + other smoking cessation treatment (behavioural support)

### Results table

| ***Study details*** | ***Outcome details*** | ***Timepoint*** | ***E-cigarette ^A^ (0 mg/mL) + Behavioural Support^B^*** | ***No intervention + Behavioural Support^B^*** | ***Notes*** |
| --- | --- | --- | --- | --- | --- |
| **Tobacco use abstinence** | | | **n/N (%)** | |  |
| Lucchiari 2020 (75)**,**Italy [RCT] | Continuous smoking abstinence (self-reported, eCO^C^ verified ≤7ppm) | 6 months | 11/70 (15.7%) | 7/70 (10.0%) | ITT analysis. |
| **Reduction in tobacco smoking frequency/quantity** | | | **Mean (SD)** | |  |
| Eisenberg 2020 (73), Canada [RCT] | Change in mean number of daily cigarettes smoked since baseline (self-reported) | 24 weeks | -9.1 (NR) | -5.5 (NR) |  |
| Lucchiari 2020 (75)**,**Italy [RCT] | Number of daily cigarettes smoked | 6 months | 14.03 (SD=7.92) | 13.45 (SD=6.49) |  |
|  | eCO^C^ | 6 months | 15.28 (SD=11.43) | 16.52 (SD=10.24) |  |
| **Adverse events** | | | **n/N (%)** | |  |
| Eisenberg 2020 (73), Canada [RCT] | Serious AEs^D^ (self-reported) | 12 weeks | 4/127 (3.1%) | 2/121 (1.7%) | Only the first event for each participant in each category was counted. |
|  |  | 12 weeks to 24 weeks | 2/127 (1.6%) | 2/121 (1.7%) |  |
|  | Mild AEs^E^ (self-reported) | 12 weeks | 118/127 (93%) | 88/121 (73%) |  |

A Eisenberg 2020: e-Cigarettes were purchased from purchased from NJOY Inc, Scottsdale, Arizona (tobacco-flavored liquid cartridges, 15 mg/mL); Lucchiari 2020: VP5 e-cigarette kit with 10 mL liquid cartridges (8 mg/mL)

B Eisenberg 2020: Participants received individual smoking cessation and relapse prevention counseling from trained research personnel at baseline, telephone follow-ups and clinic visits; Lucchiari 2020: All participants received low-intensity counseling delivered by phone (cognitive/behavioural intervention supporting smoking awareness and motivation to quit)

C Measured with Bedfont Micro Smokerlyzers (Bedfont Scientific, Maidstone, UK)

D Serious AEs were adjudicated by an end points evaluation committee and include death, respiratory, cardiovascular, neuropsychiatric or other events

E Including cough, dry mouth, headache, rhinitis, throat irritation, dyspnea, sore throat, light headedness, dizziness, mouth irritation, nausea, indigestion, mouth ulcers, or vertigo

Abbreviations: AE= Adverse event; eCO= Exhaled carbon monoxide; ITT= Intention-to-treat; SE= Standard error

### ROB results

**Summary**

| **Author/Year** | **Sequence generation** | **Allocation Concealment** | **Blinding of Participants/ Personnel** | **Blinding of Outcome Assessors** | **Incomplete Outcome Data** | **Selective Outcome Reporting** | **Other** | **Overall ROB** |
| --- | --- | --- | --- | --- | --- | --- | --- | --- |
| **tobacco use abstinence** | | | | | | | | |
| ***Continuous smoking abstinence*** | | | | | | | | |
| Lucchiari 2020 (75) | Low | Unclear | Low | Unclear | High | High | Low | **High risk** |
| **reduction in tobacco smoking frequency/quantity** | | | | | | | | |
| ***Change in the mean number of cig/day since baseline*** | | | | | | | | |
| Eisenberg 2020 (73) | Low | Low | High | Unclear | Low | Low | Low | **High risk** |
| ***Number of daily cigarettes smoked, eCO*** | | | | | | | | |
| Lucchiari 2020 (75) | Low | Unclear | Low | Unclear | High | High | Low | **High risk** |
| **adverse events** | | | | | | | | |
| ***All serious and non-serious AEs*** | | | | | | | | |
| Eisenberg 2020 (73) | Low | Low | High | Unclear | Low | Low | Low | **High risk** |

Abbreviations: AE= Adverse event; eCO= Exhaled carbon monoxide; ITT= Intention-to-treat

| **Eisenberg 2020**  **Outcomes:** Reduction, All serious and non-serious AEs | | |
| --- | --- | --- |
| **Risk of bias** | **Rating** | **Judgement support** |
| Sequence generation | Low risk | The randomization sequence was computer generated using permuted blocks of 6 and 9, stratified by centre. |
| Allocation Concealment | Low risk | Study did not report on allocation concealment method, but risk of bias judged to be low due to randomization with an online centralized system. |
| Blinding of Participants/ Personnel | All outcomes: High risk | For the e-cigarette groups, blinding was ensured by the identical appearance of the nicotine e-cigarettes and nicotine-free e-cigarettes. Those in the counselling-alone group were not blinded. Knowledge of assignment group could influence reporting. |
| Blinding of Outcome Assessors | All outcomes: Unclear risk | No details about blinding of the outcome assessors. |
| Incomplete Outcome Data | All outcomes: Low risk | 70 participants lost to follow-up (18.6%), with higher rates of loss in the counseling-alone group (29.8%). Sensitivity analyses were performed to examine the effect of missing data (complete case analysis and multiple imputation to impute missing reduction data) and results found to be similar. |
| Selective Outcome Reporting | Low risk | Outcomes reported appear to be consistent with all outcomes detailed in the study protocol. |
| Other | Low risk | No other concerns. |
| **Overall ROB** | **Outcomes: High risk** |  |

| **Lucchiari 2020**  **Outcomes:** Abstinence, Reduction, Side effects | | |
| --- | --- | --- |
| **Risk of bias** | **Rating** | **Judgement support** |
| Sequence generation | Low risk | The randomization sequence was prepared by independent personnel using a permuted block design. |
| Allocation Concealment | Unclear risk | No details provided on how allocation was concealed. However, a central pharmacy was responsible for assigning, allocating, and delivering e-cigarette liquids. |
| Blinding of Participants/ Personnel | All outcomes: Low risk | Participants and researchers were blinded to allocation status, ensured through masked nicotine content in the liquid for the e-cigarettes kit. |
| Blinding of Outcome Assessors | All outcomes: Unclear risk | No details provided on blinding of outcome assessors. |
| Incomplete Outcome Data | All outcomes: High risk | High amount of loss to follow up (26%). ITT analysis performed, but strategy for handling missing data was not reported. |
|  | Outcome: Severe AEs: High risk | Unclear how data on severe AEs were collected. |
| Selective Outcome Reporting | High risk | Primary pulmonary and smoking cessation outcomes consistent with the trial registry (NCT02422914). Activity and lifestyle outcomes registered were not reported. Outcomes at 12 months also not reported. |
| Other | Low risk | No other concerns. |
| **Overall ROB** | **All outcomes: High risk** | **High risk of bias due to not reporting some outcomes that were listed in the registry and high loss to follow-up. Additionally, unclear reporting of allocation concealment and blinding of outcome assessment.** |

Abbreviations: AE = Adverse event

## Appendix F. 12 E-cigarette with nicotine vs. Other smoking cessation treatment (NRT choices included nicotine patch, chewing gum, nasal spray, microtab, inhalator and mouth spray)

### Results table

| ***Study details*** | ***Outcome details*** | ***Timepoint*** | ***E-cigarette with nicotine ^A^*** | ***Other smoking cessation treatment (NRT choices included nicotine patch, chewing gum, nasal spray, microtab, inhalator and mouth spray) ^B^*** | ***Notes*** |
| --- | --- | --- | --- | --- | --- |
| **Tobacco use abstinence** | | | **n/N (%)** | |  |
| Myers Smith 2022 (77) UK  [RCT] | eCO***^C^*** | 6 months | 2/67 (3%) | 13/68 (10.0%) |  |
|  | Self-reported***^D^*** | 6 months | 6/67 (9) | 20/68 (29.4) |  |
| **Reduction in tobacco smoking frequency/quantity** | | | **n/N (%)** | |  |
| Myers Smith 2022 (77)**,**UK  [RCT] | CO-validated, reduction of at least 50% at 6 months ^E^ | 6 months | 4/67 (6) | 18/68 (26.5) |  |
|  | Self-reported, reduction of at least 50% at 6 months ^F^ | 6 months | 25/67 (37.3) | 45/68 (66.2) |  |
| **Adverse events** | | | **n/N (%)** | |  |
| Myers Smith 2022 (77)**,**UK [RCT] | At week 24, in the EC arm there was a report of dry mouth (n = 1) and cough/throat/chest irritation (n = 3) while in the NRT arm there was a report of itchiness (n = 1) and nausea (n = 1). | 6 months | 4/67 (6) | 2/68 (3) | Only the first event for each participant in each category was counted. |

A: Participants were shown three different refillable EC products (Innokin T18E, Smok and TECC mini with variable voltage and were instructed to obtain one of these or another product of their choice, together with initial samples of e-liquid with the strength and flavour of their choice.

B: Participants shown with different choice of Nicotine replacement treatment (NRT) products which included nicotine patch, chewing gum, nasal spray, microtab, inhalator and mouth spray and could switch to different NRT product/s if required.

C. Confirmed by reduction in end-expired CO levels of ≥ 50% from baseline

D. Self-reported abstinence with ≥ 50% reduction in the number of cigarettes smoked per day

E. CO validated confirmed by a reduction in end-expired CO levels of ≥ 50% compared to baseline (i.e., < 8 ppm)

F. confirmed by a reduction in number of cigarettes smoked of no more than 5 cigarettes smoked since 4 weeks and confirmed end-expired CO levels of < 8ppm at six months

Abbreviations: AE= Adverse event; eCO= Exhaled carbon monoxide; NRT=Nicotine replacement treatment

### RoB results

**Summary**

| **Author/Year** | **Sequence generation** | **Allocation Concealment** | **Blinding of Participants/ Personnel** | **Blinding of Outcome Assessors** | **Incomplete Outcome Data** | **Selective Outcome Reporting** | **Other** | **Overall ROB** |  |
| --- | --- | --- | --- | --- | --- | --- | --- | --- | --- |
| **Reduction in tobacco smoking frequency** | | | | | | | | | |
| ***CO-validated, reduction of at least 50% at 6 months (confirmed by a reduction in end-expired CO levels of ≥ 50% compared to baseline)*** | | | | | | | | | |
| Myers Smith (77) | Low | Low | Low | Low | Low | Low | Low | Low |  |
| ***Self-reported, reduction of at least 50% at 6 months (defined as self-reported reduction of ≥ 50% in the number of cigarettes smoked per day)*** | | | | | | | | | |
| Myers Smith (77) | Low | Low | Low | Low | Low | Low | Low | Low |  |
| **Tobacco use abstinence** | | | | | | | | | |
| ***CO-validated, confirmed by a reduction in end-expired CO levels*** | | | | | | | | | |
| Myers Smith (77) | Low | Low | Low | Low | Low | Low | Low | Low |  |
| ***Self-reported*** | | | | | | | | | |
| Myers Smith (77) | Low | Low | Low | Low | Low | Low | Low | Low |  |
| **Adverse events** | | | | | | | | | |
| ***Self-reported*** | | | | | | | | | |
| Myers Smith (77) | Low | Low | Low | Low | Low | Low | Low | Low |  |

Abbreviations: CO= Carbon monoxide

**Ratings with judgement support**

| **Myers Smith (2022)**  **Outcomes: Reduction in tobacco use, Abstinence, AEs** | | |
| --- | --- | --- |
| **Risk of bias** | **Rating** | **Judgement support** |
| Sequence generation | Low risk | Randomization was a 1:1 ratio in permuted blocks of 20 and the sequence was computer-generated done by an independent statistician. |
| Allocation Concealment | Low risk | Codes were sealed in opaque envelopes and marked with a unique randomization number. Study staff allocated randomization numbers sequentially. |
| Blinding of Participants/ Personnel | Low risk | Participants and personnel were unaware of allocation assignment and sequence generation, as was done by an independent statistician. |
| Blinding of Outcome Assessors | Low risk | Outcome assessors were unaware of participant assignment and data analysis was conducted by an independent statistician. |
| Incomplete Outcome Data | All outcomes: Low risk | Used appropriate method (i.e., multiple imputation by chained equation) to assess missing data. Plus, treated participants lost to follow-up as non-reducers. |
| Selective Outcome Reporting | Low risk | Outcomes reported do not appear to showcase any selective reporting. |
| Other | Low risk | Two authors (including the principal investigator) received previous funding and provided consultancy to Pfizer, a manufacturer of stop-smoking medication. However, the primary exposure is unrelated to stop-smoking medications. |
| **Overall ROB** | **Low risk** | **Low risk of bias due to clear randomization process and allocation concealment, blinding of participants/personnel and outcome assessors. In addition, appropriate methodology to handle missing data and no evidence of selective reporting.** |

Abbreviations: AE= Adverse event

## Appendix F. 13 E-cigarette with nicotine vs. Other smoking cessation treatment (Quit advice)

### Results table

| ***Study details*** | ***Outcome details*** | ***Timepoint*** | ***E-cigarette with nicotine^A^*** | ***Other smoking cessation treatment (Quit advice) ^B^*** | ***Notes*** |
| --- | --- | --- | --- | --- | --- |
| **Tobacco use abstinence** | | | **n/N (%)** | |  |
| Xu 2023 (78)**,**USA [RCT] | Past 30-day switching, defined as a "no" response to the question, "In the past 30 days, have you smoked a cigarette, even one or two puffs?" | 6 months | 10/271 (3.7) | 98/566 (17.3) | ITT analysis. Odds ratio was based on repeated-measure regression model that included all three follow-ups during 6-month intervention |
|  |  | 12 months | 14/271 (5.2) | 92/566 (16.1) |  |
| **Reduction in tobacco smoking frequency/quantity** | | | **n/N (%)** | |  |
| Xu 2023 (78)**,**USA [RCT] | Cigarette consumption: number of cigarettes smokes per day | 6 months | 40/271 (14.62) | 63/566 (11.16) |  |
|  |  | 12 months | 45/271 (16.4) | 199/566 (35.2) |  |

A. Participants in the JUUL groups initially received two JUUL devices with two to five packs of pods (four pods per pack, 5.0% nicotine by weight [59 mg/ml]) and they could order up to 15 four-packs of pods and additional devices each month for the first 6 months

B. Participants were provide with quit advice (QA), consisting of printed materials explaining why quitting smoking is important and behavioural advice about how to quit smoking.

### RoB results

**Summary**

| **Reduction in tobacco smoking frequency** | | | | | | | | | |
| --- | --- | --- | --- | --- | --- | --- | --- | --- | --- |
| ***Cigarette consumption: number of cigarettes smokes per day*** | | | | | | | | | |
| Xu (78) | Low | Low | High | Unclear | Unclear | Low | High | **High** |  |
| **Tobacco use abstinence** | | | | | | | | | |
| ***Past 30-day switching, defined as a "no" response to the question, "In the past 30 days, have you smoked a cigarette, even one or two puffs?"*** | | | | | | | | | |
| Xu (78) | Low | Low | High | Unclear | Unclear | Low | High | **High** |  |

**Ratings with judgement support**

| **Xu (2023)**  **Outcomes:** Reduction in tobacco smoking frequency, Tobacco use abstinence | | |
| --- | --- | --- |
| **Risk of bias** | **Rating** | **Judgement support** |
| Sequence generation | Low risk | Randomization done via a blocked randomization scheme using a random number generator in Statistical Package for the Social Sciences. |
| Allocation Concealment | Low risk | Neither participants nor study staff knew the allocation in advance. But method of concealment was not disclosed. |
| Blinding of Participants/ Personnel | High risk | Open-label. Given nature of intervention, participants knew whether they were given the JUUL treatment, same goes for the Virginia Tobacco group. |
| Blinding of Outcome Assessors | Unclear risk | Open-label. Outcome assessors were not blinded, but the outcomes were all self-reported via survey’s conducted online.  It cannot be assumed that the staff was blinded during analysis, this was not explicitly stated. |
| Incomplete Outcome Data | Unclear risk | No detail given regarding the number of drop-outs specifically from each arm.  Only 2.3% completed no follow-ups, missed follow-ups did not reflect dropping out. 91.4% completed greater than or equal to 3 follow ups. |
| Selective Outcome Reporting | Low risk | All of the outcomes outlined in the study were reported on. |
| Other | High risk | Conflict of interest bias as 3 of 4 authors have affiliations with JUUL labs. All involved in the writing and analysis of the paper. |
| **Overall ROB** | **High risk** | **Several domains of high and unclear risks** |

## Appendix F. 14 E-cigarette with nicotine vs. No E-cigarette

### Results table

| ***Study details*** | | ***Outcome details*** | ***Timepoint*** | ***Subgroup*** | ***E-cigarette with nicotine ^A^*** | | ***No E-cigarette ^B^*** | ***Notes*** |
| --- | --- | --- | --- | --- | --- | --- | --- | --- |
| **Tobacco use abstinence** | | | |  | **n/N (%)** | | |  |
| Carpenter 2023 (79)**,**USA [RCT] | | Abstinence from cigarettes (point prevalence abstinence) ^C^ | 6 months | Overall | 17/211 (8) | | 58/427 (14) | OR - estimated from models were imputed where missing = smoking (M=S), did not extract generalized linear mixed model results. |
|  |  |  |  | High motivation to quit | 10/58 (17) | | 24/116 (21) |  |
|  |  |  |  | Low Motivation to quit | 7/153 (5) | | 24/311 (11) |  |
|  |  | Floating abstinence - having ever achieved 7-days of non-smoking throughout follow-up |  | Overall | 26/211 (12) | | 72/427 (17) |  |
|  |  |  |  | High motivation to quit | 10/58 (17) | | 34/116 (29) |  |
|  |  |  |  | Low motivation to quit | 16/153 (11) | | 38/311 (12) |  |
| **Reduction in tobacco smoking frequency/quantity** | | | |  | **n/N (%)** | | |  |
| Carpenter 2023 (79)**,**USA [RCT] | | Smoking reduction (≥50% reduction in cigarettes per day (CPD)) | 6 months | Overall | 38/211 (18) | | 119/427 (28) | OR - estimated from models were imputed where missing = smoking (M=S), did not extract generalized linear mixed model results. |
|  |  |  |  | High motivation to quit | 15/58 (26) | | 44/116 (38) |  |
|  |  |  |  | Low motivation to quit | 23/153 (15) | | 75/311 (24) |  |
| **Adverse events^D^** | | | |  | **n/N (%)** | | |  |
| Carpenter 2023 (79)**,**USA [RCT] | Within the e-cigarette group, 180 people (42%) reported a total of 360 adverse events (AEs), of which 7 (2%) were severe, 113 (31%) were moderate, and 232 (64%) were mild (8 additional uncoded). The most common adverse events were cough (17%), headaches (12%), and increased phlegm (12%). Within the no-product control group, 86 people (41%) reported a total of 197 AEs, of which 7 (4%) were severe, 60 (30%) were moderate, and 124 (63%) were mild (6 additional uncoded). The most commonly reported AEs in the control group were cough (20%), increased phlegm (18%), and headaches (8.1%). | | 6 months | Overall | 86/211 (41) | 180/427 (42) | |  |

A: Participants received the NJOY device with a closed tank system, sufficiently powered with 3 ml pre-filled nicotine (15 mg/ml) and could choose up to 2 flavors among 5 offered

B. Participants did not receive e-cigs and were categorized as no-product control group.

C. Self-reported abstinence at six months

D. Self-reported adverse events

Abbreviations: AE= Adverse event; OR=Odds ratio

### RoB results

**Summary**

| **Tobacco use abstinence** | | | | | | | | |
| --- | --- | --- | --- | --- | --- | --- | --- | --- |
| ***Abstinence from cigarettes (point prevalence abstinence and Floating abstinence)*** | | | | | | | | |
| Carpenter (79) | Low | Low | High | Low | High | Low | Low | **High** |
| **Reduction in tobacco smoking frequency** | | | | | | | | |
| ***Smoking reduction (≥50% reduction in cigarettes per day (CPD))*** | | | | | | | | |
| Carpenter (79) | Low | Low | High | Low | High | Low | Low | High |
| **Adverse events** | | | | | | | | |
| ***^A^ Footnote*** | | | | | | | | |
| Carpenter (79) | Low | Low | High | Low | High | Low | Low | High |

***^A^ Within the e-cigarette group, 180 people (42%) reported a total of 360 adverse events (AEs), of which 7 (2%) were severe, 113 (31%) were moderate, and 232 (64%) were mild (8 additional uncoded). The most common adverse events were cough (17%), headaches (12%), and increased phlegm (12%). Within the no-product control group, 86 people (41%) reported a total of 197 AEs, of which 7 (4%) were severe, 60 (30%) were moderate, and 124 (63%) were mild (6 additional uncoded). The most commonly reported AEs in the control group were cough (20%), increased phlegm (18%), and headaches (8.1%).***

Abbreviations: AE= Adverse event; OR=Odds ratio; CPD=Cigarettes per day

**Ratings with judgement support**

| **Carpenter (2023)**  **Outcomes:** Tobacco use abstinence, Reduction in tobacco smoking frequency, AE | | |
| --- | --- | --- |
| **Risk of bias** | **Rating** | **Judgement support** |
| Sequence generation | Low risk | Detailed account of randomization process. |
| Allocation Concealment | Low risk | The randomization allocation was created by the study statistician and uploaded into REDCap so that the research staff team was blinded to the sequence and could randomize individuals as they were eligible for participation. |
| Blinding of Participants/ Personnel | Outcome: Tobacco use abstinence: High risk | Participants were likely to not have been blinded to group allocation (provided e-cigs vs not). Subjective reporting on tobacco use abstinence. |
|  | Outcome: Reduction in tobacco smoking frequency: High risk | Participants were likely to not have been blinded to group allocation (provided e-cigs vs not). Subjective reporting on smoking frequency. |
|  | Outcome: Adverse events: High risk | For adverse effects outcomes, participants are not blinded to group allocation, could bias/influence adverse effects reporting. |
| Blinding of Outcome Assessors | Low risk | It was stated that the research staff team was blinded to sequence. |
| Incomplete Outcome Data | All outcomes: High risk | The study outlines in general there was a high attrition rate (not specified) and in general a lack of retention, especially within the e-cigarette group. Replaced as smoking or no e-cigarette. |
| Selective Outcome Reporting | Low risk | Despite the methods not mentioning the reporting on adverse events, it is typical to include such measurements/outcomes in self-reporting efforts for tobacco related trials. |
| Other | Low risk | No other concerns. |
| **Overall ROB** | **High** | **Several areas of high risk** |

Abbreviations: AE= Adverse event

## Appendix F. 15 E-cigarette with nicotine vs. other smoking cessation intervention (Usual Care)

### Results table

| ***Study details*** | | ***Outcome details*** | ***Timepoint*** | ***E-cigarette with nicotine^A^*** | | ***Other smoking cessation intervention (Usual Care)*** *^B^* | ***Notes*** |
| --- | --- | --- | --- | --- | --- | --- | --- |
| **Tobacco use abstinence** | | | | **n/N (%)** | | |  |
| Dawkins 2020 (80)**,**UK  [RCT] | | CO validated sustained smoking abstinence (<8 ppm) | 24 weeks | 0/12 (0) | | 3/35 (9) | Per protocol analysis  Intention to treat (ITT) analysis  Per protocol analysis |
|  |  | CO validated sustained smoking abstinence (<8 ppm) |  | 0/32 (0) | | 3/48 (6.25) |  |
|  |  | 7-day point prevalence rates |  | 0/12 (0) | | 3/35 (9) |  |
| **Reduction in tobacco smoking frequency/quantity** | | | | **n/N (%)** | | |  |
| Dawkins 2020 (80)**,**UK  [RCT] | | 50% reduction in cigarettes smoked per day | 24 weeks | 3/12 (25) | | 15/35 (43) | Per protocol analysis |
|  | | 50% reduction in expired CO |  | 3/12 (25) | | 7/35 (20) | Per protocol analysis |
| **Adverse events^C^** | | | | **Mean (SD)** | | |  |
| Dawkins 2020 (80)**,**UK  [RCT] | Mental health status | | 24 weeks | 12.70 (4.42) | 5.63 (6.34) | | GAD questionnaire - 7 |
|  | Mental health status | |  | 10.82 (7.23) | 7.12 (7.22) | | PHQ- 9 [Depression score] |
| **Quality of life** | | | | **QoL. Score (SD)** | | |  |
| Dawkins 2020 (80)**,**UK  [RCT] | HRQoL (EQ-5D-3L) | | 24 weeks | 0.619 (0.238) | 0.653 (0.363) | | Per protocol analysis [descriptive system converted to a utility value based on UK population tariff, ranging from 0 (death) to 1 (perfect health). |
|  | HRQoL EQ VAS | |  | 61 (22.5) | 61.8 (21.6) | | Perceived health on the day of administration, ranging from 0 (death) to 100 (perfect health). |

A: Participants received a starter kit comprising a tank-style refillable EC with a choice of: a) nicotine strength e-liquid (12 & 18 mg/mL) and b) flavors (3 options: tobacco, fruit, menthol) with explanation on how to use the product along with a guide to e-cigarettes fact sheet.

B: Participants in the control arm received the same study information sheet and received a brief advice to quit along with a help-quit leaflet

C: Measured using two scale (i.e., GAD-7 and PHQ-9 questionnaires)

D: Measures using two scales (i.e., HRQoL[EQ-5D-3L] & HRQoL [EQ VAS])

Abbreviations: GAD=Generalized anxiety disorder; PHQ=Patient health questionnaire; CO=Carbon monoxide; HRQoL=Health related quality of life; VAS=Visual Analogue Scales

### RoB results

**Summary**

| **Tobacco use abstinence** | | | | | | | | |
| --- | --- | --- | --- | --- | --- | --- | --- | --- |
| ***Abstinent participants at month 12 - CO validated sustained smoking abstinence (ITT)*** | | | | | | | | |
| Dawkins (80) | High | High | High | High | Low | Low | Low | **High** |
| ***7-day point prevalence rates*** | | | | | | | | |
| Dawkins (80) | High | High | High | High | Low | Low | Low | **High** |
| **Reduction in tobacco smoking frequency** | | | | | | | | |
| ***50% reduction in expired CO*** | | | | | | | | |
| Dawkins (80) | High | High | High | High | Low | Low | Low | **High** |
| ***Smoking reduction (≥50% reduction in cigarettes per day (CPD))*** | | | | | | | | |
| Dawkins (80) | High | High | High | High | Low | Low | Low | **High** |
| **Quality of Life** | | | | | | | | |
| ***HRQoL (EQ-5D-3L) per protocol analysis*** | | | | | | | | |
| Dawkins (80) | High | High | High | High | Low | Low | Low | **High** |
| ***HRQoL EQ VAS (perceived health on the day of administration, ranging from 0 (death) to 100 (perfect health).*** | | | | | | | | |
| Dawkins (80) | High | High | High | High | Low | Low | Low | **High** |
| **Adverse Events** | | | | | | | | |
| ***Mental health status*** | | | | | | | | |
| Dawkins (80) | High | High | High | High | Low | Low | Low | **High** |

**Ratings with judgement support**

| **Dawkins (2020)**  **Outcomes: Tobacco abstinence; reduction in smoking frequency; QoL and Mental health status** | | |
| --- | --- | --- |
| **Risk of bias** | **Rating** | **Judgement support** |
| Sequence generation | High risk | No sequence generation. Authors had intended to randomize the centre to each condition; however, due to one centre’s readiness, authors opted not to randomize to balance potential confounders. |
| Allocation Concealment | High risk | No allocation concealment took place as participants were non-randomly allocated to either to usual care or e-cigarette arm. |
| Blinding of Participants/ Personnel | All outcomes: High Risk | Participants/personnel were not blinded; hence, knowledge of assignment is known and can influence reporting of outcome. |
| Blinding of Outcome Assessors | All outcomes:  High risk | Researchers assessing outcome could not be blinded considering the nature of the intervention. |
| Incomplete Outcome Data | All outcomes:  Low risk | Few losses to follow up and authors utilized appropriate method (ITT) to account for those lost to follow up. |
| Selective Outcome Reporting | Low risk | Outcomes reported appear to be consistent with all outcomes detailed in the study protocol. |
| Other | Low risk | No other concern. |
| **Overall ROB** | **High risk** | **High risk of bias due to lack of sequence generation, allocation concealment and blinding of participants/personnel and unclear risk of blinding of outcome assessors.** |

Abbreviations: RoB=Risk of bias

## Appendix F. 16 E-cigarette with nicotine + Support (psychological counselling) vs. E-cigarette without nicotine + Support (psychological counselling)

### Results table

| ***Study details*** | | ***Outcome details*** | ***Timepoint*** | ***"Comparison EC: E-cigarette with nicotine, S: support (psychological counselling), ECP: E-cigarette placebo*** | ***E-cigarette with nicotine^A^*** | | ***Other intervention^B^*** | ***Notes*** |
| --- | --- | --- | --- | --- | --- | --- | --- | --- |
| **Tobacco use abstinence** | | | |  | **n/N (%)** | | |  |
| Lucchiari 2022 (81)**,**Italy [RCT] | | Abstinent participants at month 12 | 12 months | EC + S vs ECP + S | 15/58 (25.9) | | 15/60 (25) |  |
| **Reduction in tobacco smoking frequency/quantity** | | | |  | **Mean (SD)** | | |  |
| Lucchiari 2022 (81)**,**Italy [RCT] | | Mean number of daily cigarettes smoked | 12 months | EC + S vs ECP + S | 13.71 (7.22) | | 16.18 (7.23) | OR - estimated from models were imputed where missing = smoking (M=S), did not extract generalized linear mixed model results. |
| **Adverse events^C^** | | | |  | **n/N (%)** | | |  |
| Lucchiari 2022 (81)**,**Italy [RCT] | Anxiety and depression levels | | 12 months | EC + S vs ECP + S | Mean (SD) score=12.17 (2.20) – [Anxiety score]  Mean (SD) score=9.13 (1.57) – [Depression score] | Mean (SD) score=12.45(2.37) – [Anxiety score]  Mean (SD) score=8.90 (1.81) – [Depression score] | | Mixed-design ANCOVAs were run to evaluate changes in cough-related quality of life (LCQ), anxiety, and depression levels (HADS). Abstinent participants were found to report a significant increase in anxiety symptoms (F = 7.072, p = 0.001), while LCQ changes signaled an improvement in their cough-related quality of life (F = 3.373, p = 0.039) |

A: Participants received a treatment based on the use of nicotine e-cigarettes (i.e., an e-cigarette kit plus 12 10-mL liquid cartridges (8 mg/mL)

B: Participants received nicotine free e-cigarettes (i.e., E-cigarette placebo)

C: The quality of anxiety and depression measured using the Hospital Anxiety and Depression Scale (HADS) which had 2 subscales (7 items each) to assess depression and anxiety.

Abbreviations: EC=Electronic cigarettes; ECP=Electronic cigarette placebo; S=Support; HADS=Hospital Anxiety and Depression Scale

## Appendix F. 17 E-cigarette with nicotine + Support (psychological counselling) vs. Support (psychological counselling)

### Results table

| ***Study details*** | ***Outcome details*** | ***Timepoint*** | ***"Comparison EC: E-cigarette with nicotine, S: support (psychological counselling)*** | ***E-cigarette with nicotine^A^*** | ***Other intervention^B^*** | ***Notes*** |
| --- | --- | --- | --- | --- | --- | --- |
| **Tobacco use abstinence** | | |  | **n/N (%)** | |  |
| Lucchiari 2022 (81)**,**Italy [RCT] | Abstinent participants at month 12 | 12 months | EC + S vs S | 10/60 (16.7) | 15/60 (25) |  |
| **Reduction in tobacco smoking frequency/quantity** | | |  | **Mean (SD)** | |  |
| Lucchiari 2022 (81)**,**Italy [RCT] | Mean number of daily cigarettes smoked | 12 months | EC + S vs S | 13.93 (7.20) | 16.18 (7.23) | OR - estimated from models were imputed where missing = smoking (M=S), did not extract generalized linear mixed model results. |

A: Participants received a treatment based on the use of nicotine e-cigarettes (i.e., an e-cigarette kit plus 12 10-mL liquid cartridges (8 mg/mL)

B: Participants received support (psychological counselling)

### RoB results

**Summary**

| **Tobacco use abstinence** | | | | | | | | | |
| --- | --- | --- | --- | --- | --- | --- | --- | --- | --- |
| ***Abstinent participants at month 12 (EC + S vs ECP + S)*** | | | | | | | | | |
| Lucchiari (81) | Low | Low | High | Unclear | Unclear | Low | Low | High |  |
| ***Abstinent participants at month 12 (EC + S vs S)*** | | | | | | | | | |
| Lucchiari (81) | Low | Low | High | Unclear | Unclear | Low | Low | High |  |
| **Reduction in tobacco smoking frequency** | | | | | | | | | |
| ***Mean number of daily cigarettes smoked (EC + S vs ECP + S)*** | | | | | | | | | |
| Lucchiari (81) | Low | Low | High | Unclear | Unclear | Low | Low | High |  |
| ***Mean number of daily cigarettes smoked (EC + S vs S)*** | | | | | | | | | |
| Lucchiari (81) | Low | Low | High | Unclear | Unclear | Low | Low | High |  |
| **Adverse Events** | | | | | | | | | |
| ***Anxiety and depression levels (using HADS) (EC+S vs ECP+S)*** | | | | | | | | | |
| Lucchiari (81) | Low | Low | High | Unclear | Unclear | Low | Low | High |  |

Abbreviations: EC=Electronic cigarettes; ECP=Electronic cigarette placebo; S=Support; HADS=Hospital Anxiety and Depression Scale

**Ratings with judgement support**

| **Lucchiari (2022)**  **Outcomes: Tobacco abstinence use, reduction in tobacco smoking frequency, and Adverse events** | | |
| --- | --- | --- |
| **Risk of bias** | **Rating** | **Judgement support** |
| Sequence generation | Low risk | Randomization sequence generated using block randomization. |
| Allocation Concealment | Low risk | Labelled with the progressive number applied to the packaging containing e-cigs and liquid cartridges with or without nicotine. Neither the participants nor the researcher in charge knows whether the liquid in the e-cigarettes kit contains nicotine. |
| Blinding of Participants/ Personnel | All outcomes: High risk | For e-cigarette groups (Arm 1 and 2), blinding was ensured by identical appearance of the nicotine e-cigarettes and nicotine-free e-cigarettes. Those in counselling group (Arm 3) were not blinded. Knowledge of assignment group could influence results. |
| Blinding of Outcome Assessors | All outcomes: Unclear risk | No details about blinding of the outcome assessors. |
| Incomplete Outcome Data | Unclear risk | Due to drop-outs authors utilize ITT analysis. However, authors do not describe reasons for dropouts or any missing data and do not describe how missing data was handled. |
| Selective Outcome Reporting | Low risk | Outcomes reported appear to be consistent with all outcomes detailed in protocol. |
| Other | Low risk | No other concern |
| **Overall ROB** | **High risk** | **Knowledge of participants assignment to treatment arms may influence reporting. In addition, lack of information of on randomization sequence and allocation concealment plus unclear methodology on handling missing data yield a ‘high’ risk of : bias.** |

Abbreviations: RoB=Risk of bias

## Appendix F. 18 E-cigarette with nicotine + vs. Non-nicotine cigarette substitute (CS)

### Results table

| ***Study details*** | ***Outcome details*** | ***Timepoint*** | ***E-cigarette with nicotine^A^*** | ***Non-nicotine cigarette substitute (CS)^B^*** | ***Notes*** |
| --- | --- | --- | --- | --- | --- |
| **Tobacco use abstinence** | | | **n/N (%)** | |  |
| Foulds 2022 (82)**,**USA [RCT] | 7-day point prevalence (CO <10 ppm) abstinence | 24 weeks | 4/130 (3.1) | 14/130 (10.8) |  |
|  | 28+ days abstinent with CO <10 at weeks 20 and 24 |  | 2/130 (1.5) | 10/130 (7.7) |  |
|  | Mean days on days of no cigarette smoking from week 1 to 24 |  | **Mean (SD)** | |  |
|  |  |  | 5.3 (18.5) | 15.6 (36.4) |  |

A: Participants received an eGo-style ENDS paired with 8 mg/mL nicotine liquid with tobacco or menthol flavor

B: Participants received a cigarette shaped plastic tube with no electronics or aerosol for use as a cigarette substitute (CS)

Abbreviations: CS=Cigarette substitute; CO=Carbon monoxide

## Appendix F. 19 E-cigarette with nicotine + vs. E-cigarette without nicotine (ECP)

### Results table

| ***Study details*** | ***Outcome details*** | ***Timepoint*** | ***E-cigarette with nicotine^A^*** | ***E-cigarette without nicotine (ECP)^B^*** | ***Notes*** |
| --- | --- | --- | --- | --- | --- |
| **Tobacco use abstinence** | | | **n/N (%)** | |  |
| Foulds 2022 (82)**,**USA [RCT] | 7-day point prevalence (CO <10) abstinence | 24 weeks | 1/130 (0.8) | 14/130 (10.8) |  |
|  | 28+ days abstinent with CO <10 at weeks 20 and 24 |  | 1/130 (0.8) | 10/130 (7.7) |  |
|  | Mean days on days of no cigarette smoking from week 1 to 24 |  | **Mean (SD)** | |  |
|  |  |  | 4.7 (17) | 15.6 (36.4) |  |

A: Participants received an eGo-style ENDS paired with 8 mg/mL nicotine liquid with tobacco or menthol flavor

B: Participants received an eGo-style ENDS paired with 0 mg/mL nicotine liquid as E-cigarette placebo (ECP)

Abbreviations: ECP=E-Cigarette placebo; CO=Carbon monoxide

### RoB results

| **Tobacco use abstinence** | | | | | | | | |
| --- | --- | --- | --- | --- | --- | --- | --- | --- |
| ***7-day point prevalance (CO <10) abstinence (EC vs CS)*** | | | | | | | | |
| Foulds (82) | Low | Low | Low | High | Low | Low | Low | **High** |
| ***7-day point prevalance (CO <10) abstinence (EC vs ECP)*** | | | | | | | | |
| Foulds (82) | Low | Low | Low | High | Low | Low | Low | **High** |
| ***28+ days abstinent with CO <10 at weeks 20 and 24 (EC vs CS)*** | | | | | | | | |
| Foulds (82) | Low | Low | Low | High | Low | Low | Low | **High** |
| ***28+ days abstinent with CO <10 at weeks 20 and 24 (EC vs ECP)*** | | | | | | | | |
| Foulds (82) | Low | Low | Low | High | Low | Low | Low | **High** |
| ***Mean days on days of no cigarette smoking from week 1 to 24 (EC vs CS)*** | | | | | | | | |
| Foulds (82) | Low | Low | Low | High | Low | Low | Low | **High** |
| ***Mean days on days of no cigarette smoking from week 1 to 24 (EC vs CP)*** | | | | | | | | |
| Foulds (82) | Low | Low | Low | High | Low | Low | Low | **High** |

**Ratings with judgement support**

| **Foulds (2021)**  **Outcomes: Tobacco use abstinence** | | |
| --- | --- | --- |
| **Risk of bias** | **Rating** | **Judgement support** |
| Sequence generation | Low risk | Randomization performed using sample function in R software (Blocks of 8). |
| Allocation Concealment | Low risk | Randomized in-person and in real-time using an electronic function within REDCap (i.e., data collection/management system) that revealed allocation to either an ENDS or CS at the time of randomization. |
| Blinding of Participants/ Personnel | All outcomes:  Low risk | Participants were blinded through receiving identical eGo-style e-cigarette with varying levels of nicotine liquid depending. Cigarette substitute group received cigarette like tube without no electronic or aerosol in it. |
| Blinding of Outcome Assessors | All outcomes:  High risk | Those analyzing data were not masked to condition assignment. |
| Incomplete Outcome Data | Low risk | Considerable participants lost to follow up; however, authors provide explanation for loss to follow up and those who withdrew for other reasons. Authors utilized imputation method and conducted ITT analysis appropriately. |
| Selective Outcome Reporting | Low risk | Outcomes reported appear to be consistent with all outcomes detailed in protocol. |
| Other | Low risk | No other concern. |
| **Overall ROB** | **High risk** | **Since the blinding of outcome assessor’s domain is at high risk, the overall RoB is judged having ‘high’ risk of bias.** |
