# Supplementary material for: Effectiveness of e-cigarettes as a stop smoking intervention in adults: a systematic review
Source: Syst Rev. 2024 Jun 29;13:168. doi: 10.1186/s13643-024-02572-7 (PMC11218295; doi:10.1186/s13643-024-02572-7)
Supplement: Supplementary file 8 — Additional file 8: Appendix 8. GRADE Evidence Profile and Summary of Findings (SoF) tables. [file 13643_2024_2572_MOESM8_ESM.docx]

**Appendix G. GRADE Evidence Profile & Summary of Findings Tables**

Table of Contents

[Appendix H Table 1. E-cigs with nicotine versus No intervention: Adverse events, weight gain, and quality of life in general/mixed population of smokers 3](#_Toc164682289)

[Summary of findings: 5](#_Toc164682290)

[Appendix H Table 2. E-cigs with nicotine versus Waitlist: Adverse events in general/mixed population of smokers 8](#_Toc164682291)

[Summary of findings: 9](#_Toc164682292)

[Appendix H Table 3. E-cigs with nicotine plus usual care versus Usual care: Smoking cessation, reduction, quality of life, adverse events, and change in emotional state in patients with periodontitis 10](#_Toc164682293)

[Summary of findings: 13](#_Toc164682294)

[Appendix H Table 4. E-cigs with nicotine plus standard care versus E-cigs without nicotine plus standard care: Smoking cessation, reduction, and adverse events in smokers willing to quit 16](#_Toc164682295)

[Summary of findings: 18](#_Toc164682296)

[Appendix H Table 5. E-cigs with nicotine versus E-cigs without nicotine: Smoking cessation, reduction, adverse events, and weight gain in smokers not intending to quit 20](#_Toc164682297)

[Summary of findings: 23](#_Toc164682298)

[Appendix H Table 6. E-cigarette with nicotine + other smoking cessation treatment vs E-cigarette with no nicotine + other smoking cessation treatment: Smoking cessation, reduction, and adverse events 27](#_Toc164682299)

[Summary of findings: 31](#_Toc164682300)

[Appendix H Table 7. E-cigarette with nicotine + other smoking cessation treatment vs no intervention + other smoking cessation treatment: Smoking cessation, reduction, and adverse events 38](#_Toc164682301)

[Summary of findings: 40](#_Toc164682302)

[Appendix H Table 8. E-cigarette with nicotine + other smoking cessation treatment + standard care vs. E-cigarette with no nicotine + other smoking cessation treatment + standard care: Smoking cessation, reduction, adverse events, possible adverse outcomes 44](#_Toc164682303)

[Summary of findings: 47](#_Toc164682304)

[Appendix H Table 9. E-cigarette with nicotine + other smoking cessation treatment + standard care vs. Other smoking cessation treatment + standard care: Smoking cessation, reduction, adverse events, possible adverse outcomes 53](#_Toc164682305)

[Summary of findings: 56](#_Toc164682306)

[Appendix H Table 10. E-cigarette with no nicotine + other smoking cessation treatment + standard care vs. Other smoking cessation treatment + standard care: Smoking cessation, reduction, adverse events, possible adverse outcomes 61](#_Toc164682307)

[Summary of findings: 64](#_Toc164682308)

[Appendix H Table 11. E-cigarette with no nicotine + other smoking cessation treatment vs. Other smoking cessation treatment: Smoking cessation, reduction, adverse events 69](#_Toc164682309)

[Summary of findings: 71](#_Toc164682310)

[Appendix H Table 12. E-cigarette with nicotine vs. Other smoking cessation treatment (NRT choices included nicotine patch, chewing gum, nasal spray, microtab, inhalator and mouth spray): Smoking cessation, reduction, adverse events 75](#_Toc164682311)

[Summary of findings: 77](#_Toc164682312)

[Appendix H Table 13. E-cigarette with nicotine vs. Other smoking cessation treatment (Quit advice): Smoking cessation, reduction 79](#_Toc164682313)

[Summary of findings: 81](#_Toc164682314)

[Appendix H Table 14. E-cigarette with nicotine vs. No E-cigarette: Smoking cessation, reduction 83](#_Toc164682315)

[Summary of findings: 86](#_Toc164682316)

[Appendix H Table 15. E-cigarette with nicotine vs. other smoking cessation intervention (Usual Care) Smoking cessation, reduction, Quality of Life, adverse events 91](#_Toc164682317)

[Summary of findings: 94](#_Toc164682318)

[Appendix H Table 16. E-cigarette with nicotine + Support (psychological counselling) vs. E-cigarette without nicotine + Support (psychological counselling) Smoking cessation, reduction, adverse events 97](#_Toc164682319)

[Summary of findings: 99](#_Toc164682320)

[Appendix H Table 17. E-cigarette with nicotine + Support (psychological counselling) vs. Support (psychological counselling) Smoking cessation, reduction 101](#_Toc164682321)

[Summary of findings: 102](#_Toc164682322)

[Appendix H Table 18. E-cigarette with nicotine + vs. Non-nicotine cigarette substitute Smoking cessation, reduction 104](#_Toc164682323)

[Summary of findings: 106](#_Toc164682324)

[Appendix H Table 19. E-cigarette with nicotine + vs. E-cigarette without nicotine Smoking cessation, reduction 108](#_Toc164682325)

[Summary of findings: 110](#_Toc164682326)

## Appendix H Table 1. E-cigs with nicotine versus No intervention: Adverse events, weight gain, and quality of life in general/mixed population of smokers

Electronic cigarettes with nicotine versus no intervention in general/mixed population of smokers

**Bibliography:** Carpenter 2017; Cravo 2016; Flacco 2019; Date of last search September 2020

| **Certainty assessment** | | | | | | | **Summary of findings** | | | | |
| --- | --- | --- | --- | --- | --- | --- | --- | --- | --- | --- | --- |
| **№ of participants (studies) Follow-up** | **Risk of bias** | **Inconsistency** | **Indirectness** | **Imprecision** | **Publication bias** | **Overall certainty of evidence** | **Study event rates (%)** | | **Relative effect (95% CI)** | **Anticipated absolute effects** | |
|  |  |  |  |  |  |  | **no intervention** | **e-cigarettes with nicotine** |  | **Risk with no intervention** | **Risk difference with e-cigarettes with nicotine** |
| **Adverse events (follow up: range 12 weeks to 16 weeks)^a,b,c^**  assessed with: AEs collected by systematic assessment (no further details)) | | | | | | | | | | | |
| up to 1808  (2 RCTs) | very serious ^d^ | not serious ^e^ | not serious ^f^ | serious to very serious ^g^ | none ^h^ | ⨁◯◯◯  VERY LOW | As some adverse events may be device-specific and type of vaping liquid may affect type of adverse events, a quantitative analysis between studies has not been performed.  Both studies. Five serious adverse events in e-cigarette group of one study (n=1740); no events occurred in other study (n=68). A higher frequency of adverse events reported with e-cigarettes in both studies.  One study (n=1740). AEs were analyzed in different ways with a potential for double counting participants experiencing multiple events. The proportion of mild (29.6% vs 28.4%), moderate (54.6% vs 57.3%), and severe (15.8% vs 14.2%) adverse events were similar between groups. Authors also report varying degrees of attribution of adverse events to the e-cigarette intervention, with 50% accounting for events that are possibly related to the intervention, while 28% were unrelated; all events experienced in the no intervention group were considered not related to allocation. Only two events in the e-cigarette group led to withdrawal (none in no intervention group). | | | | |
| 68  (1 RCT) | serious ^d^ | not serious ^e^ | not serious^f^ | serious to very serious ^i^ | none ^h^ | unable to assess | No mortality events occurred in either group. | | | | |
| 1355  (1 cohort) | not serious | not serious | not serious | serious to very serious ^i^ | none ^h^ | unable to assess | Study authors reported that “no serious adverse events and no higher risk were reported for e-cig when compared to traditional tobacco smoking.” | | | | |
| **Possible adverse outcomes: Body weight (follow up: 12 weeks)** ^j,k,l^  assessed with: not reported | | | | | | | | | | | |
| 408  (1 RCT) | very serious ^m^ | serious ^n^ | not serious ^f^ | not serious ^o^ | none ^h^ | ⨁◯◯◯  VERY LOW | 102 | 306 | - | The mean change in body weight was **0.1** kg | MD **0.2 kg higher** (3.53 lower to 3.93 higher) |
| **Quality of life: Change in self-reported health (follow up: baseline-4 years)**  assessed with: final item of the Italian version of the EuroQol EQ-D5L | | | | | | | | | | | |
| 532  (1 cohort) | not serious | not serious | not serious | serious to very serious ^i^ | none ^h^ | unable to assess | 123 | 409 | - | The mean change was **0.1** | MD **0.2 lower** (0.49 lower to 0.09 higher) |

**CI:** Confidence interval; **MD:** Mean difference

#### Explanations

1. One study where some participants were motivated to quit (4 to 5 out of 10 on a VAS scale); second study excluded those motivated to quit.
2. No co-interventions mentioned in either study.
3. E-cigarette nicotine concentrations varied from 2.7 mg/capsule to 16-24 mg/mL for study duration.
4. Adverse events: High risk for performance, detection, and selective reporting biases. Unclear risk for selection bias; we downrate by -2.0. All-cause mortality: Unclear risk for selection and attrition biases; we downrate by -1.0.
5. Given reporting, difficult to ascertain inconsistency, but not likely for adverse events. One study for mortality. We do not downrate.
6. Meets criteria for review update. Unknown whether e-cigarette type(s) not available in Canada.
7. Unable to assess confidence intervals. Sample sizes vary, but up to 1808 participants and the optimal information size cannot be met. We cannot rate this domain.
8. Although few studies, comprehensive literature search undertaken and inclusive of negative findings. We do not downrate this domain.
9. Unable to assess confidence intervals. The optimal information size cannot be met with small sample size. We cannot rate this domain.
10. E-cigarette nicotine concentration 2.7 mg/capsule.
11. No co-interventions.
12. Excluded participants who were motivated to quit.
13. High risk of bias for selective outcome reporting. Unclear risk for selection, performance, and detection biases. We downrate by -2.0.
14. One study only. We do not downrate this domain.
15. Confidence interval encompasses little to no difference. The optimal information size is met and there is an adequate sample size (>400 participants for continuous outcome). We did not downrate this domain.

| Summary of findings: | | | | | | |
| --- | --- | --- | --- | --- | --- | --- |
| **E-cigarettes with nicotine compared to no intervention in the general/mixed population of smokers** | | | | | | |
| **Patient or population**: General/mixed population of smokers  **Setting**: Varied  **Intervention**: Electronic cigarettes with nicotine  **Comparison**: No intervention | | | | | | |
| Outcomes | **Anticipated absolute effects^*^** (95% CI) | | Relative effect (95% CI) | № of participants  (studies) | Certainty of the evidence (GRADE) | Comments |
|  | **Risk with no intervention** | **Risk with e-cigarettes with nicotine** |  |  |  |  |
| Adverse events^a,b,c^  Assessed with: AEs collected by systematic assessment (no further details)  Follow up: range 12 weeks to 16 weeks | As some adverse events may be device-specific and type of vaping liquid may affect type of adverse events, a quantitative analysis between studies has not been performed.  Both studies. Five serious adverse events in e-cigarette group of one study (n=1740); no events occurred in other study (n=68). A higher frequency of adverse events reported with e-cigarettes in both studies.    One study (n=1740). AEs were analyzed in different ways with a potential for double counting participants experiencing multiple events. The proportion of mild (29.6% vs 28.4%), moderate (54.6% vs 57.3%), and severe (15.8% vs 14.2%) adverse events were similar between groups. Authors also report varying degrees of attribution of adverse events to the e-cigarette intervention, with 50% accounting for events that are possibly related to the intervention, while 28% were unrelated; all events experienced in the no intervention group were considered not related to allocation. Only two events in the e-cigarette group led to withdrawal (none in no intervention group). | |  | Up to 1808  (2 RCTs) | ⨁◯◯◯  VERY LOW ^d,e,f,g,h^ | Date of last search: September 2020. |
| Follow up: baseline to 4 years | Study authors reported that “no serious adverse events and no higher risk were reported for e-cig when compared to traditional tobacco smoking.” | |  | 1355  (1 cohort) | unable to assess^i,h^ | Date of last search: September 2020. |
| All-cause mortality^a,b,c^  Assessed with: Not reported  Follow-up: range 12 weeks to 16 weeks | No mortality events occurred in either group. | |  | 68  (1 RCT) | unable to assess^d,e,f,h,i^ | Date of last search: September 2020. |
| Possible adverse outcomes: Body weight ^j,k,l^  Assessed with: Not reported  Follow up: 12 weeks | The mean change in body weight was 0.1 kg | MD **0.2 kg higher** (3.53 lower to 3.93 higher) | - | 408 (1 RCT) | ⨁◯◯◯  VERY LOW ^f,h,m,n,o^ | Date of last search: September 2020. |
| Quality of life: Change in self-reported health  Assessed with: final item of the Italian version of the EuroQol EQ-D5L  Follow up: baseline-4 years | The mean change was **0.1** | MD **0.2 lower** (0.49 lower to 0.09 higher) | - | 532  (1 cohort) | unable to assess ^i,h^ | Date of last search: September 2020. |
| ***The risk in the intervention group** (and its 95% confidence interval) is based on the assumed risk in the comparison group and the **relative effect** of the intervention (and its 95% CI).  **CI:** Confidence interval; **MD:** Mean difference | | | | | | |
| **GRADE Working Group grades of evidence** **High certainty:** We are very confident that the true effect lies close to that of the estimate of the effect **Moderate certainty:** We are moderately confident in the effect estimate: The true effect is likely to be close to the estimate of the effect, but there is a possibility that it is substantially different **Low certainty:** Our confidence in the effect estimate is limited: The true effect may be substantially different from the estimate of the effect **Very low certainty:** We have very little confidence in the effect estimate: The true effect is likely to be substantially different from the estimate of effect | | | | | | |

#### Explanations

1. One study where some participants were motivated to quit (4 to 5 out of 10 on a VAS scale); second study excluded those motivated to quit.
2. No co-interventions mentioned in either study.
3. E-cigarette nicotine concentrations varied from 2.7 mg/capsule to 16-24 mg/mL for study duration.
4. Adverse events: High risk for performance, detection, and selective reporting biases. Unclear risk for selection bias; we downrate by -2.0. All-cause mortality: Unclear risk for selection and attrition biases; we downrate by -1.0.
5. Given reporting, difficult to ascertain inconsistency, but not likely for adverse events. One study for mortality. We do not downrate.
6. Meets criteria for review update. Unknown whether e-cigarette type(s) not available in Canada.
7. Unable to assess confidence intervals. Sample sizes vary, but up to 1808 participants and the optimal information size cannot be met. We cannot rate this domain.
8. Although few studies, comprehensive literature search undertaken and inclusive of negative findings. We do not downrate this domain.
9. Unable to assess confidence intervals. The optimal information size cannot be met with 68 participants. We cannot rate this domain.
10. E-cigarette nicotine concentration 2.7 mg/capsule.
11. No co-interventions.
12. Excluded participants who were motivated to quit.
13. High risk of bias for selective outcome reporting. Unclear risk for selection, performance, and detection biases. We downrate by -2.0.
14. One study only. We do not downrate this domain.
15. Confidence interval encompasses little to no difference. The optimal information size is met and there is an adequate sample size (>400 participants for continuous outcome). We did not downrate this domain.

## Appendix H Table 2. E-cigs with nicotine versus Waitlist: Adverse events in general/mixed population of smokers

| E-cigarettes with nicotine versus Waitlist in the general/mixed population of smokers  **Bibliography**: Adriaens 2014; Date of last search September 2020 | | | | | | | | | | | |
| --- | --- | --- | --- | --- | --- | --- | --- | --- | --- | --- | --- |
| **Certainty assessment** | | | | | | | **Summary of findings** | | | | |
| **№ of participants (studies) Follow-up** | **Risk of bias** | **Inconsistency** | **Indirectness** | **Imprecision** | **Publication bias** | **Overall certainty of evidence** | **Study event rates (%)** | | **Relative effect (95% CI)** | **Anticipated absolute effects** | |
|  |  |  |  |  |  |  | **waitlist** | **e-cigarettes with nicotine** |  | **Risk with waitlist** | **Risk difference with e-cigarettes with nicotine** |
| **Adverse events: Complaints (follow up: range 1 weeks to 8 weeks)**^a,b^  Assessed with: Self-reported | | | | | | | | | | | |
| 48  (1 RCT) | very serious ^c^ | not serious ^d^ | serious ^e^ | serious to very serious ^f^ | none ^g^ | ⨁◯◯◯  VERY LOW | Approximately 2 mean complaints in either e-cigarette group and about 3 mean complaints in the waitlist group, as measured in weekly or biweekly interval across the 8-week period.  Small sample size in each group (n=16 in each), and no clear details on which types of complaints were reported for the e-cigarette and waitlist groups. | | | | |

**CI:** Confidence interval

#### Explanations

1. The two e-cigarette groups evaluated different brand names but at 18 mg/mL nicotine dosing each and were used ad libitum with or without cigarettes. The waitlist control was instructed to continue smoking.
2. No co-interventions reported.
3. High risk for performance and detection biases, unclear for selection and selective reporting biases. We downrate by -2.0.
4. One study only. We do not downrate this domain.
5. E-cigarettes were used ad libitum with or without cigarettes. It would not be possible to attribute a complaint to a particular device or cigarettes for those who were dual users. We downrate by -1.0.
6. Unable to assess confidence intervals. The optimal information size cannot be met with 16 participants in each group. We cannot rate this domain.
7. Although few studies, literature search was comprehensive. No suspicion of suppression of results of other studies. We do not downrate this domain.

| Summary of findings: | | | | |  |
| --- | --- | --- | --- | --- | --- |
| **E-cigarettes with nicotine compared to Waitlist in the general population of smokers** | | | | | |
| **Patient or population**: The general population of smokers (4.2% with concrete plans to quit)  **Setting**: Academic research setting  **Intervention**: Electronic cigarettes with nicotine  **Comparison**: Waitlist | | | | | |
| Outcomes | Impact | № of participants  (studies) | Certainty of the evidence (GRADE) | Comments | |
|  |  |  |  |  |  |
| Adverse events: Complaints ^a,b^  Assessed with: Self-reported  Follow up: range 1 weeks to 8 weeks | Approximately 2 mean complaints in either e-cigarette group and about 3 mean complaints in the waitlist group, as measured in weekly or biweekly interval across the 8-week period.  Small sample size (n=16) in each of the three groups, with no clear details on which types of complaints were reported for the e-cigarette and waitlist groups. | 48  (1 RCT) | ⨁◯◯◯  VERY LOW ^c,d,e,f,g^ | Date of last search: September 2020.  Vaping liquids used in these devices may be different between devices, which could impact occurrence and frequency of adverse events. | |
| ***The risk in the intervention group** (and its 95% confidence interval) is based on the assumed risk in the comparison group and the **relative effect** of the intervention (and its 95% CI).   **CI:** Confidence interval | | | | | |
| **GRADE Working Group grades of evidence** **High certainty:** We are very confident that the true effect lies close to that of the estimate of the effect **Moderate certainty:** We are moderately confident in the effect estimate: The true effect is likely to be close to the estimate of the effect, but there is a possibility that it is substantially different **Low certainty:** Our confidence in the effect estimate is limited: The true effect may be substantially different from the estimate of the effect **Very low certainty:** We have very little confidence in the effect estimate: The true effect is likely to be substantially different from the estimate of effect | | | | | |

#### Explanations

1. The two e-cigarette groups evaluated different brand names but at 18 mg/mL nicotine dosing each and were used ad libitum with or without cigarettes. The waitlist control was instructed to continue smoking.
2. No co-interventions reported.
3. High risk for performance and detection biases, unclear for selection and selective reporting biases. We downrate by -2.0.
4. One study only. We do not downrate this domain.
5. E-cigarettes were used ad libitum with or without cigarettes. It would not be possible to attribute a complaint to a particular device or cigarettes for those who were dual users. We downrate by -1.0.
6. Unable to assess confidence intervals. The optimal information size cannot be met with 16 participants in each group. We cannot rate this domain.
7. Although few studies, literature search was comprehensive. No suspicion of suppression of results of other studies. We do not downrate this domain.

## Appendix H Table 3. E-cigs with nicotine plus usual care versus Usual care: Smoking cessation, reduction, quality of life, adverse events, and change in emotional state in patients with periodontitis

E-cigarettes with nicotine plus usual care versus Usual care in patients with periodontitis

**Bibliography:** Holliday 2019; Date of last search September 2020

| **Certainty assessment** | | | | | | | | **Summary of findings** | | | | |
| --- | --- | --- | --- | --- | --- | --- | --- | --- | --- | --- | --- | --- |
| **№ of participants (studies) Follow-up** | **Risk of bias** | **Inconsistency** | **Indirectness** | **Imprecision** | **Publication bias** | **Overall certainty of evidence** | **Study event rates (%)** | | | **Relative effect (95% CI)** | **Anticipated absolute effects** | |
|  |  |  |  |  |  |  | **Usual care** | | **e-cigarettes with nicotine plus usual care** |  | **Usual care** | **e-cigarettes with nicotine plus usual care** |
| **Continuous eCO-verified smoking abstinence (follow up: 6 months)** ^a,b,c^ | | | | | | | | | | | | |
| 80  (1 RCT) | very serious ^d^ | not serious ^e^ | serious ^f^ | very serious ^g^ | none ^h^ | ⨁◯◯◯  VERY LOW | 2/40 (5.0%) | | 6/40 (15.0%) | **RR 3.00** (0.64 to 13.98) | 50 per 1,000 | **100 more per 1,000** (from 18 fewer to 649 more) |
| **Reduction in tobacco smoking frequency/quantity - eCO levels (follow up: 6 months)** ^a,b,c^ | | | | | | | | | | | | |
| 58  (1 RCT) | very serious ^d^ | not serious ^e^ | serious ^f^ | serious ^i^ | none ^h^ | ⨁◯◯◯  VERY LOW | 29 | | 29 | **-** | The mean reduction from baseline in tobacco smoking frequency/quantity - eCO levels was 5.8 ppm | MD **6.2 ppm lower** (12.21 lower to 0.19 lower) |
| **Reduction in tobacco smoking frequency/quantity - Salivary cotinine (follow up: 6 months)** ^a,b,c^ | | | | | | | | | | | | |
| 58  (1 RCT) | very serious ^d^ | not serious ^e^ | serious ^f^ | very serious ^j^ | none ^h^ | ⨁◯◯◯  VERY LOW | 29 | | 29 | **-** | The mean reduction from baseline in tobacco smoking frequency/quantity - Salivary cotinine was 37.1 ng/mL | MD **25.1 ng/mL lower**  (93.48 lower to 43.28 higher) |
| **Reduction in tobacco smoking frequency/quantity - Salivary anabasine (follow up: 6 months)** ^a,b,c^ | | | | | | | | | | | | |
| 58  (1 RCT) | very serious ^d^ | not serious ^e^ | serious ^f^ | serious ^k^ | none ^h^ | ⨁◯◯◯  VERY LOW | 29 | | 29 | **-** | The mean increase from baseline in tobacco smoking frequency/ quantity - Salivary anabasine was 0.5 ng/mL | MD **0.9 ng/mL lower** (1.54 lower to 0.34 higher) |
| **Quality of Life (follow up: 6 months)** ^a,b,c^  Assessed with: UK Oral Health-Related QoL (higher score represents better quality of life); Scale from: 12 to 80 | | | | | | | | | | | | |
| 58  (1 RCT) | very serious ^l^ | not serious ^e^ | serious ^f^ | serious ^m^ | none ^h^ | ⨁◯◯◯  VERY LOW | 29 | | 29 | **-** | The mean change from baseline quality of life was 8.2 points higher | MD **1.4 points higher** (5.9 lower to 8.7 higher) |
| **Adverse events (follow up: 6 months)** ^a,b,c^ | | | | | | | | | | | | |
| -  (1 RCT) | very serious ^l^ | not serious ^e^ | serious ^f^ | serious to very serious ^n^ | none ^h^ | ⨁◯◯◯  VERY LOW | No serious adverse events in groups.  E-cigarettes vs usual care: Various dental adverse events reported, 20 (range 2 to 11) vs 35 total events (range 0 to 5) for: toothache, dentine hypersensitivity, dental/periodontal abscess, tooth loss, mouth ulceration, soreness of soft tissues, fractured or caries filling or tooth, and other miscellaneous events (not reported). A patient may have reported more than one AE. | | | | | |
| **Possible adverse outcomes: Change in emotional state (follow up: 6 months)** ^a,b,c^  Assessed with: Mood and Physical Symptoms Scale (higher score represents worse symptoms); Scale from: 5 to 35 | | | | | | | | | | | | |
| 58  (1 RCT) | very serious ^l^ | not serious ^e^ | serious ^f^ | serious ^m^ | none ^h^ | ⨁◯◯◯  VERY LOW | 29 | | 29 | **-** | The mean change from baseline in emotional state was 2.8 points lower | MD **0 points**  (4.4 lower to 4.4 higher) |

**CI:** Confidence interval; **RR:** Risk ratio; **MD:** Mean difference

#### Explanations

1. One study in participants with no information relating to willingness or motivation to quit.
2. No co-interventions.
3. E-cigarette starter kit (2 weeks supply of liquid) with the choice of nicotine dosage (0, 6, 12, or 18 mg/mL) and flavour. No participants selected the 0 mg/mL dose.
4. High risk of bias for attrition, selective reporting and contamination of control group. We downrate by -2.0.
5. One study only. We do not downrate this domain.
6. The starter kit included a 2-week supply of liquid. Follow-up was at six months at which point participants may have changed dosages of the cartridge or changed use of e-cigarettes. We downrate by -1.0.
7. Confidence interval encompasses harm (small but important) to benefit (large). The optimal information size not met (total of 8 events) and inadequate sample size (<2000 participants). We downrate this domain by -2.0.
8. Although few studies, literature search was comprehensive. No suspicion of suppression of results of other studies. We do not downrate this domain.
9. Confidence interval encompasses one range of effect (small but important benefit to little to no difference). The optimal information size not met and inadequate sample size (<400 participants for a continuous outcome). We downrate this domain by -1.0.
10. Confidence interval encompasses both harm (small but important) to benefit (small but important). The optimal information size not met and inadequate sample size (<400 participants for a continuous outcome). We downrate this domain by -2.0.
11. Confidence interval encompasses one range of effect (small but important benefit to little to no difference). The optimal information size not met and inadequate sample size (<400 participants for a continuous outcome). We downrate this domain by -1.0.
12. High risk for performance, detection, attrition, and selective reporting biases and contamination of control group. We downrate by -2.0.
13. Confidence interval encompasses little to no difference. The optimal information size not met and inadequate sample size (<400 participants for a continuous outcome). We downrate this domain by -1.0.
14. Unable to assess confidence intervals. Unclear sample size analyzed but <50 events reported. We cannot rate this domain.

| Summary of findings: | | | | | | |
| --- | --- | --- | --- | --- | --- | --- |
| **E-cigarettes with nicotine plus usual care compared to Usual care in patients with periodontitis** | | | | | | |
| **Patient or population**: Patients with periodontitis  **Setting**: Dental office  **Intervention**: Electronic cigarettes with nicotine plus usual care  **Comparison**: Usual care | | | | | | |
| Outcomes | **Anticipated absolute effects^*^** (95% CI) | | Relative effect (95% CI) | № of participants  (studies) | Certainty of the evidence (GRADE) | Comments |
|  | **Risk with Usual care** | **Risk with e-cigarettes with nicotine plus usual care** |  |  |  |  |
| Continuous eCO-verified smoking abstinence ^a,b,c^  Follow up: 6 months | 50 per 1,000 | **150 per 1,000** (32 to 699) | **RR 3.00** (0.64 to 13.98) | 80 (1 RCT) | ⨁◯◯◯  VERY LOW - ^d,e,f,g,h^ | Date of last search: September 2020. |
| Reduction in tobacco smoking frequency/quantity - eCO levels ^a,b,c^  Follow up: 6 months | The mean reduction from baseline in tobacco smoking frequency/quantity - eCO levels was 5.8 ppm | MD **6.2 ppm lower** (12.21 lower to 0.19 lower) | - | 58 (1 RCT) | ⨁◯◯◯  VERY LOW ^d,e,f,h,i^ | Date of last search: September 2020. |
| Reduction in tobacco smoking frequency/quantity - Salivary cotinine ^a,b,c^  Follow up: 6 months | The mean reduction from baseline in tobacco smoking frequency/quantity - Salivary cotinine was 37.1 ng/mL | MD **25.1 ng/mL lower** (93.48 lower to 43.28 higher) | - | 58 (1 RCT) | ⨁◯◯◯  VERY LOW ^d,e,f,h,j^ | Date of last search: September 2020. |
| Reduction in tobacco smoking frequency/quantity - Salivary anabasine ^a,b,c^  Follow up: 6 months | The mean increase from baseline in tobacco smoking frequency/quantity - Salivary anabasine was 0.5 ng/mL | MD **0.9 ng/mL lower** (1.54 lower to 0.34 higher) | - | 58 (1 RCT) | ⨁◯◯◯  VERY LOW ^d,e,f,h,k^ | Date of last search: September 2020. |
| Quality of Life ^a,b,c^  Assessed with: UK Oral Health-Related QoL  Follow up: 6 months | The mean change from baseline quality of life score was 8.2 points higher | MD **1.4 points higher** (5.9 lower to 8.7 higher) | - | 58 (1 RCT) | ⨁◯◯◯  VERY LOW ^e,f,h,l,m^ | Date of last search: September 2020. |
| Adverse events ^a,b,c^  Follow up: 6 months | No serious adverse events in groups.  E-cigarettes vs usual care: Various dental adverse events reported, 20 (range 2 to 11) vs 35 total events (range 0 to 5) for: toothache, dentine hypersensitivity, dental/periodontal abscess, tooth loss, mouth ulceration, soreness of soft tissues, fractured or caries filling or tooth, and other miscellaneous events (not reported). A patient may have reported more than one AE. | |  | (1 RCT) | ⨁◯◯◯  VERY LOW ^e,f,h,l,n^ | Date of last search: September 2020.  Difficulty in the interpretation of small numbers and unit of analysis. |
| Possible adverse outcomes: Change in emotional state ^a,b,c^  Assessed with: Mood and Physical Symptoms Scale (higher score represents worse symptoms) Scale from: 5 to 35  Follow up: 6 months | The mean change from baseline in emotional state was 2.8 points lower | MD **0 points**  (4.4 lower to 4.4 higher) | - | 58 (1 RCT) | ⨁◯◯◯  VERY LOW ^e,f,h,l,m^ | Date of last search: September 2020. |
| ***The risk in the intervention group** (and its 95% confidence interval) is based on the assumed risk in the comparison group and the **relative effect** of the intervention (and its 95% CI).   **CI:** Confidence interval; **RR:** Risk ratio; **MD:** Mean difference | | | | | | |
| **GRADE Working Group grades of evidence** **High certainty:** We are very confident that the true effect lies close to that of the estimate of the effect **Moderate certainty:** We are moderately confident in the effect estimate: The true effect is likely to be close to the estimate of the effect, but there is a possibility that it is substantially different **Low certainty:** Our confidence in the effect estimate is limited: The true effect may be substantially different from the estimate of the effect **Very low certainty:** We have very little confidence in the effect estimate: The true effect is likely to be substantially different from the estimate of effect | | | | | | |

#### Explanations

1. One study in participants with no information relating to willingness or motivation to quit.
2. No co-interventions.
3. E-cigarette starter kit (2 weeks supply of liquid) with the choice of nicotine dosage (0, 6, 12, or 18 mg/mL) and flavour. No participants selected the 0 mg/mL dose.
4. High risk of bias for attrition, selective reporting and contamination of control group. We downrate by -2.0.
5. One study only. We do not downrate this domain.
6. The starter kit included a 2-week supply of liquid. Follow-up was at six months at which point participants may have changed dosages of the cartridge or changed use of e-cigarettes. We downrate by -1.0.
7. Confidence interval encompasses harm (small but important) to benefit (large). The optimal information size not met (total of 8 events) and inadequate sample size (<2000 participants). We downrate this domain by -2.0.
8. Although few studies, literature search was comprehensive. No suspicion of suppression of results of other studies. We do not downrate this domain.
9. Confidence interval encompasses one range of effect (small but important benefit to little to no difference). The optimal information size not met and inadequate sample size (<400 participants for a continuous outcome). We downrate this domain by -1.0.
10. Confidence interval encompasses both harm (small but important) to benefit (small but important). The optimal information size not met and inadequate sample size (<400 participants for a continuous outcome). We downrate this domain by -2.0.
11. Confidence interval encompasses one range of effect (small but important benefit to little to no difference). The optimal information size not met and inadequate sample size (<400 participants for a continuous outcome). We downrate this domain by -1.0.
12. High risk for performance, detection, attrition, and selective reporting biases and contamination of control group. We downrate by -2.0.
13. Confidence interval encompasses little to no difference. The optimal information size not met and inadequate sample size (<400 participants for a continuous outcome). We downrate this domain by -1.0.
14. Unable to assess confidence intervals. Unclear sample size analyzed but <50 events reported. We cannot rate this domain.

## Appendix H Table 4. E-cigs with nicotine plus standard care versus E-cigs without nicotine plus standard care: Smoking cessation, reduction, and adverse events in smokers willing to quit

E-cigarettes with nicotine plus standard care versus e-cigarettes with no nicotine plus standard care in smokers willing to quit

**Bibliography:** Baldassarri 2019; Date of last search September 2020

| **Certainty assessment** | | | | | | | | **Summary of findings** | | | | |
| --- | --- | --- | --- | --- | --- | --- | --- | --- | --- | --- | --- | --- |
| **№ of participants (studies) Follow-up** | **Risk of bias** | **Inconsistency** | **Indirectness** | **Imprecision** | **Publication bias** | **Overall certainty of evidence** | **Study event rates (%)** | | | **Relative effect (95% CI)** | **Anticipated absolute effects** | |
|  |  |  |  |  |  |  | **e-cigarettes with no nicotine plus standard care** | | **e-cigarettes with nicotine plus standard care** |  | **Risk with e-cigarettes with no nicotine plus standard care** | **Risk difference with e-cigarettes with nicotine plus standard care** |
| **Abstinence: 7-day point prevalence confirmed by exCO ≤6ppm measured (follow up: 24 weeks)** ^a,b,c^ | | | | | | | | | | | | |
| 40  (1 RCT) | serious ^d^ | not serious ^e^ | serious ^f^ | very serious ^g^ | none ^h^ | ⨁◯◯◯  VERY LOW | 2/20 (10.0%) | | 4/20 (20.0%) | **RR 2.00** (0.41 to 9.71) | 100 per 1,000 | **100 more per 1,000** (from 59 fewer to 871 more) |
| **Reduction in tobacco smoking frequency/quantity (follow up: 24 weeks)** ^a,b,c^  Assessed with: change in mean number of cigarettes smoked/day | | | | | | | | | | | | |
| 40  (1 RCT) | serious ^d^ | not serious ^e^ | serious ^f^ | serious ^i^ | none ^h^ | ⨁◯◯◯  VERY LOW | 20 | | 20 | **-** | The mean reduction in tobacco smoking frequency/quantity was **8.04** | Smaller reduction by MD **2.54** (4.62 greater reduction to 9.7 smaller reduction) |
| **Adverse events (follow up: 24 weeks)** ^a,b,c^ | | | | | | | | | | | | |
| 40  (1 RCT) | serious ^d^ | not serious ^e^ | serious ^f^ | serious to very serious ^j^ | none ^h^ | ⨁◯◯◯  VERY LOW | E-cigarette with nicotine vs e-cigarette with no nicotine: Various side effects reported, 28 (range 0 to 7) vs 21 total events (range 0 to 5) for: abnormal dreams, anxiety, fatigue, headache, insomnia, nausea, palpitations, pruritus, cough, shortness of breath, sore throat, and increased appetite. A participant may have reported more than one AE. Small sample sizes (n=20) in each group. | | | | | |

**CI:** Confidence interval; **OR:** Odds ratio; **MD:** Mean difference

#### Explanations

1. One study in participants who were willing to quit.
2. No co-interventions.
3. Both groups received the same brand of e-cigarette, with one group receiving cartridge with 24 mg/mL of nicotine and the other group receiving cartridge with 0 mg/mL of nicotine. Both groups also received standard care which consisted of nicotine patch and counselling sessions.
4. 7-day point prevalence: unclear for selection and selective reporting biases. Other outcomes: unclear for selection, attrition, and selective reporting biases. For all outcomes we downrate by -1.0.
5. One study only. We do not downrate this domain.
6. E-cigarette and patch were used for 8 weeks and then participants were allowed any intervention for the remaining 16 weeks and use of the e-cig as a substitute for cigarette smoking was encouraged, but not considered mandatory and was at the discretion of the participants. We downrate by -1.0.
7. Confidence interval encompasses harm (moderate) to benefit (large). The optimal information size not met (total of 6 events) and inadequate sample size (<2000 participants). We downrate this domain by -2.0.
8. Although few studies, literature search was comprehensive. No suspicion of suppression of results of other studies. We do not downrate this domain.
9. Confidence interval encompasses little to no difference. The optimal information size not met and inadequate sample size (<400 participants for a continuous outcome). We downrate this domain by -1.0.
10. Unable to assess confidence intervals. Approximately 20 participants in each group and the optimal information size cannot be met. We cannot rate this domain.

| Summary of findings: | | | | | | |
| --- | --- | --- | --- | --- | --- | --- |
| **E-cigarettes with nicotine plus standard care compared to e-cigarettes with no nicotine plus standard care in smokers willing to quit** | | | | | | |
| **Patient or population**: Smokers willing to quit  **Setting**: Not reported. Participants recruited from outpatient pulmonary and primary care clinics, tobacco treatment service, and through referrals from medical providers.  **Intervention**: Electronic cigarettes with nicotine plus standard care  **Comparison**: Electronic cigarettes with no nicotine plus standard care | | | | | | |
| Outcomes | **Anticipated absolute effects^*^** (95% CI) | | Relative effect (95% CI) | № of participants  (studies) | Certainty of the evidence (GRADE) | Comments |
|  | **Risk with e-cigarettes with no nicotine plus standard care** | **Risk with e-cigarettes with nicotine plus standard care** |  |  |  |  |
| Abstinence: 7-day point prevalence confirmed by exCO ≤6ppm measured ^a,b,c^  Follow up: 24 weeks | 100 per 1,000 | **200 per 1,000** (41 to 971) | **RR 2.00** (0.41 to 9.71) | 40 (1 RCT) | ⨁◯◯◯  VERY LOW ^d,e,f,g,h^ | Date of last search: September 2020. |
| Reduction in tobacco smoking frequency/quantity assessed with: change in mean number of cigarettes smoked/day ^a,b,c^  Follow up: 24 weeks | The mean reduction in tobacco smoking frequency/quantity was 8.04 | Smaller reduction by MD **2.54** (4.62 greater reduction to 9.7 smaller reduction) | - | 40 (1 RCT) | ⨁◯◯◯  VERY LOW ^d,e,f,h,i^ | Date of last search: September 2020.  The absolute difference was 2.5 cigarettes per day higher in the group given nicotine, ranging from approximately five fewer cigarettes per day to almost 10 more cigarettes per day, a range of 15 cigarettes per day. |
| Adverse events ^a,b,c^  Follow up: 24 weeks | E-cigarette with nicotine vs e-cigarette with no nicotine: Various side effects reported, 28 (range 0 to 7) vs 21 total events (range 0 to 5) for: abnormal dreams, anxiety, fatigue, headache, insomnia, nausea, palpitations, pruritus, cough, shortness of breath, sore throat, and increased appetite. A participant may have reported more than one AE. Small sample sizes (n=20) in each group. | |  | 40  (1 RCT) | ⨁◯◯◯  VERY LOW ^d,e,f,h,j^ | Date of last search: September 2020. |
| ***The risk in the intervention group** (and its 95% confidence interval) is based on the assumed risk in the comparison group and the **relative effect** of the intervention (and its 95% CI).   **CI:** Confidence interval; **OR:** Odds ratio; **MD:** Mean difference | | | | | | |
| **GRADE Working Group grades of evidence** **High certainty:** We are very confident that the true effect lies close to that of the estimate of the effect **Moderate certainty:** We are moderately confident in the effect estimate: The true effect is likely to be close to the estimate of the effect, but there is a possibility that it is substantially different **Low certainty:** Our confidence in the effect estimate is limited: The true effect may be substantially different from the estimate of the effect **Very low certainty:** We have very little confidence in the effect estimate: The true effect is likely to be substantially different from the estimate of effect | | | | | | |

#### Explanations

1. One study in participants who were willing to quit.
2. No co-interventions.
3. Both groups received the same brand of e-cigarette, with one group receiving cartridge with 24 mg/mL of nicotine and the other group receiving cartridge with 0 mg/mL of nicotine. Both groups also received standard care which consisted of nicotine patch and counselling sessions.
4. 7-day point prevalence: unclear for selection and selective reporting biases. Other outcomes: unclear for selection, attrition, and selective reporting biases. For all outcomes we downrate by -1.0.
5. One study only. We do not downrate this domain.
6. E-cigarette and patch were used for 8 weeks and then participants were allowed any intervention for the remaining 16 weeks and use of the e-cig as a substitute for cigarette smoking was encouraged, but not considered mandatory and was at the discretion of the participants. We downrate by -1.0.
7. Confidence interval encompasses harm (moderate) to benefit (large). The optimal information size not met (total of 6 events) and inadequate sample size (<2000 participants). We downrate this domain by -2.0.
8. Although few studies, literature search was comprehensive. No suspicion of suppression of results of other studies. We do not downrate this domain.
9. Confidence interval encompasses little to no difference. The optimal information size not met and inadequate sample size (<400 participants for a continuous outcome). We downrate this domain by -1.0.
10. Unable to assess confidence intervals. Approximately 20 participants in each group and the optimal information size cannot be met. We cannot rate this domain.

## Appendix H Table 5. E-cigs with nicotine versus E-cigs without nicotine: Smoking cessation, reduction, adverse events, and weight gain in smokers not intending to quit

E-cigarettes with nicotine versus e-cigarettes with no nicotine in smokers not intending to quit in the next 30 days

**Bibliography:** Caponnetto 2013; Russo 2016; Date of last search September 2020

| **Certainty assessment** | | | | | | | | **Summary of findings** | | | | |
| --- | --- | --- | --- | --- | --- | --- | --- | --- | --- | --- | --- | --- |
| **№ of participants (studies) Follow-up** | **Risk of bias** | **Inconsistency** | **Indirectness** | **Imprecision** | **Publication bias** | **Overall certainty of evidence** | **Study event rates (%)** | | | **Relative effect (95% CI)** | **Anticipated absolute effects** | |
|  |  |  |  |  |  |  | **e-cigarettes with no nicotine** | | **e-cigarettes with nicotine** |  | **Risk with e-cigarettes with no nicotine** | **Risk difference with e-cigarettes with nicotine** |
| **Tobacco use abstinence: Quit rates (follow up: 24 weeks)^a,b,c,d^**  Assessed with: self-reported (not even a puff) with an eCO of <=7ppm since the previous study visit | | | | | | | | | | | | |
| 300  (1 RCT) | not serious ^e^ | not serious ^f^ | serious ^g^ | very serious ^h^ | none ^i^ | ⨁◯◯◯  VERY LOW | 5/100 (5.0%) | | 22/200 (11.0%) | **RR 2.20** (0.86 to 5.64) | 50 per 1,000 | **60 more per 1,000** (from 7 fewer to 232 more) |
| **Tobacco use abstinence: Quit rates (follow up: 52 weeks)^a,b,c,d^**  Assessed with: self-reported (not even a puff) with an eCO of <=7ppm since the previous study visit | | | | | | | | | | | | |
| 300  (1 RCT) | not serious ^e^ | not serious ^f^ | serious ^g^ | very serious ^j^ | none ^i^ | ⨁◯◯◯  VERY LOW | 4/100 (4.0%) | | 22/200 (11.0%) | **RR 2.76** (0.97 to 7.76) | 40 per 1,000 | **70 more per 1,000** (from 1 fewer to 270 more) |
| **Reduction in tobacco smoking frequency/quantity: 50% or greater reduction in the number of cig/day since baseline (follow up: 24 weeks)^a,b,c,d^**  Assessed with: self-reported reduction | | | | | | | | | | | | |
| 300  (1 RCT) | serious ^k^ | not serious ^f^ | serious ^g^ | very serious ^l^ | none ^i^ | ⨁◯◯◯  VERY LOW | 15/100 (15.0%) | | 36/200 (18.0%) | **RR 1.20** (0.69 to 2.08) | 150 per 1,000 | **30 more per 1,000** (from 47 fewer to 162 more) |
| **Reduction in tobacco smoking frequency/quantity: 50% or greater reduction in the number of cig/day since baseline (follow up: 52 weeks)^a,b,c,d^**  Assessed with: self-reported reduction | | | | | | | | | | | | |
| 300  (1 RCT) | not serious ^e^ | not serious ^f^ | serious ^g^ | very serious ^m^ | none ^i^ | ⨁◯◯◯  VERY LOW | 12/100 (12.0%) | | 19/200 (9.5%) | **RR 0.79** (0.40 to 1.57) | 120 per 1,000 | **25 fewer per 1,000** (from 72 fewer to 68 more) |
| **Reduction in tobacco smoking frequency/quantity (follow up: 52 weeks)^a,b,c^**  Assessed with: eCO levels through a portable device | | | | | | | | | | | | |
| -  (1 RCT) | not serious ^e^ | not serious ^f^ | serious ^g^ | serious to very serious ^n^ | none ^i^ | unable to assess | Among all participants in the per protocol analysis there was no statistically significant difference between groups in eCO levels at 52 weeks (Group A&B: pooled mean 17.55 ppm, Group C: 17.77 ppm). There was a high number of missing participants (35/100 in group A, 37/100 in group B, and 45/100 in group C). After excluding quitters, there was no statistically significant difference between groups (Group A&B: 17.8 ppm, Group C: 20 ppm). | | | | | |
| **Adverse events (all AEs and side effects) (follow up: 52 weeks) ^a,b,c^** | | | | | | | | | | | | |
| -  (1 RCT) | not serious ^e^ | not serious ^f^ | serious ^g^ | serious to very serious ^n^ | none ^i^ | unable to assess | There were no serious AEs reported. Various side effects reported, ranging from 2 to 6% in the groups who were given nicotine and 2 to 7% in the no nicotine group for: hunger, insomnia, irritability, anxiety, and depression. AEs were reported as the most frequently reported before using e-cigarettes and noted in the AE page of the study diary. AEs reported between weeks 12 and 52 remained fairly stable within groups with some exceptions (e.g., shortness of breath increased in all groups). There were small sample sizes in each group (n=100). | | | | | |
| **Possible adverse outcomes: Change in weight from baseline (follow up: 52 weeks) ^a,b,c^** | | | | | | | | | | | | |
| -  (1 RCT) | serious ^o^ | not serious ^f^ | serious ^g^ | serious to very serious ^n^ | none ^i^ | ⨁◯◯◯  VERY LOW | The study authors present the data as a percent of baseline weight, and have made the data unitless as they set have the baseline as 100%. As we do not have individual patient data, and a percent change would depend on starting weight, no formal analysis has been done on this outcome. There was some fluctuation in weight gain from baseline to weeks 12, 24 and 52, ranging from 0.93 to 0.99% from baseline weight in Group A, 0.62 to 1.64% of baseline weight in Group B, and 0.28 to 0.76% of baseline weight in Group C. However, depending on the actual baseline weight in pounds/kg, these percentages may vary in actual weight gain. | | | | | |

**CI:** Confidence interval; **RR:** Risk ratio

#### Explanations

1. One study in participants who were not intending to quit or wishing to do so in the next 30 days.
2. No co-interventions.
3. All groups received the same Categoria e-cig (model 401), with difference in dosage of nicotine (i.e., 7.2 mg/mL for 12 weeks, 7.2 mg/mL for six weeks followed by 5.4 mg/mL for six weeks, no nicotine for 12 weeks).
4. As there was no difference between the two groups who received nicotine, these results were combined in the GRADE tables, but are presented by group in the results tables.
5. Unclear for selection bias. We downrate by -0.5.
6. One study only. We do not downrate this domain.
7. After the 12-week intervention period, no more cartridges were provided and participants were advised to continue using their e-cigarette if they wished to do so. It was also possible for the 0 mg/mL group to use liquid with nicotine. We downrate by -1.0.
8. Confidence interval encompasses harm (small but important) to benefit (large). The optimal information size not met (total of 27 events) and inadequate sample size (<2000 participants). We downrate this domain by -2.0.
9. Although few studies, literature search was comprehensive. No suspicion of suppression of results of other studies. We do not downrate this domain.
10. Confidence interval encompasses three ranges of effect (little to no difference to large benefit). The optimal information size not met (total of 26 events) and inadequate sample size (<2000 participants). We downrate this domain by -2.0.
11. High risk for attrition bias and unclear risk for selection bias; we downrate by -1.5.
12. Confidence interval encompasses harm (small but important) to benefit (moderate). The optimal information size not met (total of 51 events) and inadequate sample size (<2000 participants). We downrate this domain by -2.0.
13. Confidence interval encompasses harm (moderate) to benefit (small but important). The optimal information size not met (total of 31 events) and inadequate sample size (<2000 participants). We downrate this domain by -2.0.
14. Unable to assess confidence intervals. Unclear sample size analyzed but a maximum of 300 total participants (with several missing participants) could be included. Thus, the optimal information size cannot be met. We cannot rate this domain.
15. Unclear risk for selection and attrition biases; we downrate by -1.0.

| Summary of findings: | | | | | | |  |
| --- | --- | --- | --- | --- | --- | --- | --- |
| **E-cigarettes with nicotine compared to e-cigarettes with no nicotine in smokers not intending to quit in the next 30 days** | | | | | | | |
| **Patient or population**: Smokers not intending to quit or wishing to do so in the next 30 days  **Setting**: Academic research setting  **Intervention**: Electronic cigarettes with nicotine  **Comparison**: Electronic cigarettes with no nicotine | | | | | | | |
| Outcomes | **Anticipated absolute effects^*^** (95% CI) | | Relative effect (95% CI) | № of participants  (studies) | Certainty of the evidence (GRADE) | Comments | |
|  | **Risk with e-cigarettes with no nicotine** | **Risk with e-cigarettes with nicotine** |  |  |  |  |  |
| Tobacco use abstinence: Quit rates ^a,b,c,d^  Assessed with: self-reported (not even a puff) with an eCO of <=7ppm since the previous study visit  Follow up: 24 weeks | 50 per 1,000 | 110 per 1,000 (43 to 282) | **RR 2.20** (0.86 to 5.64) | 300 (1 RCT) | ⨁◯◯◯  VERY LOW ^e,f,g,h,i^ | Date of last search: September 2020.  The results are presented for all three groups. As there was no difference between groups who received nicotine, the GRADE results are presented by combining the nicotine groups together. | |
| Tobacco use abstinence: Quit rates ^a,b,c,d^  Assessed with: self-reported (not even a puff) with an eCO of <=7ppm since the previous study visit  Follow up: 52 weeks | 40 per 1,000 | 110 per 1,000 (39 to 310) | **RR 2.75** (0.97 to 7.76) | 300 (1 RCT) | ⨁◯◯◯  VERY LOW ^e,f,g,i,j^ | Date of last search: September 2020.  The results are presented for all three groups. As there was no difference between groups who received nicotine, the GRADE results are presented by combining the nicotine groups together. | |
| Reduction in tobacco smoking frequency/quantity: 50% or greater reduction in the number of cig/day since baseline ^a,b,c,d^  Assessed with: self-reported reduction  Follow up: 24 weeks | 150 per 1,000 | 180 per 1,000 (104 to 312) | **RR 1.20** (0.69 to 2.08) | 300 (1 RCT) | ⨁◯◯◯  VERY LOW ^f,g,i,k,l^ | Date of last search: September 2020.  The results are presented for all three groups. As there was no difference between groups who received nicotine, the GRADE results are presented by combining the nicotine groups together. | |
| Reduction in tobacco smoking frequency/quantity: 50% or greater reduction in the number of cig/day since baseline ^a,b,c,d^  Assessed with: self-reported reduction  Follow up: 52 weeks | 120 per 1,000 | 95 per 1,000 (48 to 188) | **RR 0.79** (0.40 to 1.57) | 300 (1 RCT) | ⨁◯◯◯  VERY LOW ^e,f,g,i,m^ | Date of last search: September 2020.  Results were also provided excluding quitters and by including only those with ≥80% reduction, showing no difference between groups. These results were also presented as a combined results for those who were provided an e-cigarette with nicotine. Results were also reported per-protocol analysis, and results report that those that were lost to follow-up were not significantly different from participants who completed the study, with the exception of gender. | |
| Reduction in tobacco smoking frequency/quantity ^a,b,c^  Assessed with: eCO levels through a portable device  Follow up: 52 weeks | Among all participants in the per protocol analysis there was no statistically significant difference between groups in eCO levels at 52 weeks (Group A&B: pooled mean 17.55 ppm, Group C: 17.77 ppm). There was a high number of missing participants (35/100 in group A, 37/100 in group B, and 45/100 in group C). After excluding quitters, there was no statistically significant difference between groups (Group A&B: 17.8 ppm, Group C: 20 ppm). | |  | (1 RCT) | unable to assess ^e,f,g,i,n^ | Date of last search: September 2020.  Results were reported per-protocol analysis, and results report that those that were lost to follow-up were not significantly different from participants who completed the study, with the exception of gender. | |
| Adverse events (all AEs and side effects) ^a,b,c^  Follow up: 52 weeks | There were no serious AEs reported. Various side effects reported, ranging from 2 to 6% in the groups who were given nicotine and 2 to 7% in the no nicotine group for: hunger, insomnia, irritability, anxiety, and depression. AEs were reported as the most frequently reported before using e-cigarettes and noted in the AE page of the study diary. AEs reported between weeks 12 and 52 remained fairly stable within groups with some exceptions (e.g., shortness of breath increased in all groups). There were small sample sizes in each group (n=100). | |  | (1 RCT) | unable to assess ^e,f,g,i,n^ | Date of last search: September 2020. | |
| Possible adverse outcomes: Change in weight from baseline ^a,b,c^  Follow up: 52 weeks | The study authors present the data as a percent of baseline weight, and have made the data unitless as they set have the baseline as 100%. As we do not have individual patient data, and a percent change would depend on starting weight, no formal analysis has been done on this outcome. There was some fluctuation in weight gain from baseline to weeks 12, 24 and 52, ranging from 0.93 to 0.99% from baseline weight in Group A, 0.62 to 1.64% of baseline weight in Group B, and 0.28 to 0.76% of baseline weight in Group C. However, depending on the actual baseline weight in pounds/kg, these percentages may vary in actual weight gain. | |  | (1 RCT) | ⨁◯◯◯  VERY LOW ^f,g,i,n,o^ | Date of last search: September 2020. | |
| ***The risk in the intervention group** (and its 95% confidence interval) is based on the assumed risk in the comparison group and the **relative effect** of the intervention (and its 95% CI).  **CI:** Confidence interval; **RR:** Risk ratio | | | | | | | |
| **GRADE Working Group grades of evidence** **High certainty:** We are very confident that the true effect lies close to that of the estimate of the effect **Moderate certainty:** We are moderately confident in the effect estimate: The true effect is likely to be close to the estimate of the effect, but there is a possibility that it is substantially different **Low certainty:** Our confidence in the effect estimate is limited: The true effect may be substantially different from the estimate of the effect **Very low certainty:** We have very little confidence in the effect estimate: The true effect is likely to be substantially different from the estimate of effect | | | | | | | |

#### Explanations

1. One study in participants who were not intending to quit or wishing to do so in the next 30 days.
2. No co-interventions.
3. All groups received the same Categoria e-cig (model 401), with difference in dosage of nicotine (i.e., 7.2 mg/mL for 12 weeks, 7.2 mg/mL for six weeks followed by 5.4 mg/mL for six weeks, no nicotine for 12 weeks).
4. As there was no difference between the two groups who received nicotine, these results were combined in the GRADE tables, but are presented by group in the results tables.
5. Unclear for selection bias. We downrate by -0.5.
6. One study only. We do not downrate this domain.
7. After the 12-week intervention period, no more cartridges were provided and participants were advised to continue using their e-cigarette if they wished to do so. It was also possible for the 0 mg/mL group to use liquid with nicotine. We downrate by -1.0.
8. Confidence interval encompasses harm (small but important) to benefit (large). The optimal information size not met (total of 27 events) and inadequate sample size (<2000 participants). We downrate this domain by -2.0.
9. Although few studies, literature search was comprehensive. No suspicion of suppression of results of other studies. We do not downrate this domain.
10. Confidence interval encompasses three ranges of effect (little to no difference to large benefit). The optimal information size not met (total of 26 events) and inadequate sample size (<2000 participants). We downrate this domain by -2.0.
11. High risk for attrition bias and unclear risk for selection bias; we downrate by -1.5.
12. Confidence interval encompasses harm (small but important) to benefit (moderate). The optimal information size not met (total of 51 events) and inadequate sample size (<2000 participants). We downrate this domain by -2.0.
13. Confidence interval encompasses harm (moderate) to benefit (small but important). The optimal information size not met (total of 31 events) and inadequate sample size (<2000 participants). We downrate this domain by -2.0.
14. Unable to assess confidence intervals. Unclear sample size analyzed but a maximum of 300 total participants (with several missing participants) could be included. Thus, the optimal information size cannot be met. We cannot rate this domain.
15. Unclear risk for selection and attrition biases; we downrate by -1.0.

## Appendix H Table 6. E-cigarette with nicotine + other smoking cessation treatment vs E-cigarette with no nicotine + other smoking cessation treatment: Smoking cessation, reduction, and adverse events

E-cigarette with nicotine + other smoking cessation treatment (behavioural support) vs E-cigarette with no nicotine + other smoking cessation treatment (behavioural support)

**Bibliography:** Bullen 2013; Lucchiari 2020; Eisenberg 2020; Date of last search September 2020

| **Certainty assessment** | | | | | | | | **Summary of findings** | | | | | | | | |
| --- | --- | --- | --- | --- | --- | --- | --- | --- | --- | --- | --- | --- | --- | --- | --- | --- |
| **№ of participants (studies) Follow-up** | **Risk of bias** | **Inconsistency** | **Indirectness** | **Imprecision** | **Publication bias** | **Overall certainty of evidence** | **Study event rates (%)** | | | | **Relative effect (95% CI)** | | **Anticipated absolute effects** | | | |
|  |  |  |  |  |  |  | **e-cigarettes with nicotine + other treatment** | | **e-cigarettes with no nicotine + other treatment** | |  |  | **Risk with e-cigarettes with no nicotine + other treatment** | | | **Risk difference with e-cigarettes with nicotine + other treatment** |
| **Tobacco use abstinence: Continuous smoking abstinence (ITT analysis) (follow up: 6 months)^a,b,c^**  Assessed with: self-reported, allowing ≤5 cigarettes in total, eCOD of <10ppm | | | | | | | | | | | | | | | | |
| 657  (1 RCT) | not serious ^d^ | not serious ^e^ | not serious ^f^ | very serious ^g^ | none ^h^ | ⨁⨁◯◯ LOW | 21/289 (7.3%) | | 3/73  (4.1%) | | **RR 1.77** (0.54 to 5.77) | | 41 per 1,000 | | | **32 more per 1,000** (from 19 fewer to 196 more) |
| **Tobacco use abstinence: 7-day point prevalence abstinence (follow up: 6 months)^a,b,c^**  Assessed with: self-reported no smoking of tobacco cigarettes in the past 7 days) | | | | | | | | | | | | | | | | |
| 657  (1 RCT) | not serious ^d^ | not serious ^e^ | not serious ^f^ | very serious ^g^ | none ^h^ | ⨁⨁◯◯ LOW | 61/289 (21.1%) | | 16/73  (21.9%) | | **RR 0.96** (0.59 to 1.57) | | 219 per 1,000 | | | **9 fewer per 1,000** (from 90 fewer to 125 more) |
| **Tobacco use abstinence: Continuous smoking abstinence (follow up: 6 months)^i,j,k^**  Assessed with: self-reported, eCO^C^ verified ≤7ppm | | | | | | | | | | | | | | | | |
| 140  (1 RCT) | very serious ^l^ | not serious ^e^ | not serious ^f^ | very serious ^m^ | none ^h^ | ⨁◯◯◯  VERY LOW | 13/70 (18.6%) | | 11/70  (15.7%) | | **RR 1.18** (0.57 to 2.46) | | 157 per 1,000 | | | **2**8 **more per 1,000** (from 68 fewer to 229 more) |
| **Reduction in tobacco smoking frequency/quantity: Proportion of participants who reduced daily cigarettes by 50% or greater (follow up: 6 months)^a,b,c^**  Assessed with: self-reported | | | | | | | | | | | | | | | | |
| 657  (1 RCT) | not serious ^d^ | not serious ^e^ | not serious ^f^ | serious ^n^ | none ^h^ | ⨁⨁⨁◯  MODERATE | 165/289 (57.1%) | | 33/73  (45.2%) | | **RR 1.26** (0.96 to 1.66) | | 452 per 1,000 | | | **118 more per 1,000** (from 18 fewer to 298 more) |
| **Reduction in tobacco smoking frequency/quantity: Change in mean number of daily cigarettes smoked since baseline (follow up: 24 weeks)^o,p,q^**  Assessed with: self-reported | | | | | | | | | | | | | | | | |
| 249  (1 RCT) | very serious ^r^ | not serious ^e^ | not serious ^s^ | very serious ^t^ | none ^h^ | ⨁◯◯◯  VERY LOW | At 24 weeks, the intervention group had a -10.7 change in mean number of daily cigarettes smoked since baseline and the control group had -9.1. SD were not reported. | | | | | | | | | |
| **Reduction in tobacco smoking frequency/quantity: Number of daily cigarettes smoked (follow up: 6 months)^i,j,k^**  Assessed with: self-reported | | | | | | | | | | | | | | | | |
| 140  (1 RCT) | very serious ^l^ | not serious ^e^ | not serious ^f^ | serious ^u^ | none ^h^ | ⨁◯◯◯  VERY LOW | 70 | | 70 | | **-** | | The mean number of daily cigarettes smoked was 14.03 (SD 7.92) | | | **MD 3.02 lower** (5.42 lower to 0.62 lower) |
| **Reduction in tobacco smoking frequency/quantity: eCO levels (follow up: 6 months)^i,j,k^**  Assessed with: measured with Bedfont Micro Smokerlyzers (Bedfont Scientific, Maidstone, UK) | | | | | | | | | | | | | | | | |
| 140  (1 RCT) | very serious ^l^ | not serious ^e^ | not serious ^f^ | serious ^u^ | none ^h^ | ⨁◯◯◯  VERY LOW | 70 | | | 70 | | - | | The mean change in eCO levels was 15.28 (SD 11.43) | **MD 3.27 higher** (6.56 lower to 0.02 higher) | |
| **Adverse events: Serious adverse events (follow up: 12 weeks)^o,p,q^**  Assessed with: self-reported | | | | | | | | | | | | | | | | |
| 249  (1 RCT) | very serious ^r^ | not serious ^e^ | not serious ^s^ | very serious ^t^ | none ^v^ | ⨁◯◯◯  VERY LOW | Serious adverse events were adjudicated by an end points evaluation committee and include death, respiratory, cardiovascular, neuropsychiatric or other events. At 12 weeks, the intervention group had experienced 1 (0.8%) and the control group had experienced 4 (3.1%). | | | | | | | | | |
| **Adverse events: Serious adverse events (follow up: 12 to 24 weeks) ^o,p,q^**  Assessed with: self-reported | | | | | | | | | | | | | | | | |
| 249  (1 RCT) | very serious ^r^ | not serious ^e^ | not serious ^s^ | very serious ^t^ | none ^v^ | ⨁◯◯◯  VERY LOW | Serious adverse events were adjudicated by an end points evaluation committee and include death, respiratory, cardiovascular, neuropsychiatric or other events. At 12 to 24 weeks, the intervention group had experienced 2 (1.6%) and the control group had experienced 2 (1.6%). | | | | | | | | | |
| **Adverse events: Mild adverse events (follow up: 12 weeks) ^o,p,q^**  Assessed with: self-reported | | | | | | | | | | | | | | | | |
| 249  (1 RCT) | very serious ^r^ | not serious ^e^ | not serious ^s^ | very serious ^t^ | none ^v^ | ⨁◯◯◯  VERY LOW | Mild adverse events included cough, dry mouth, headache, rhinitis, throat irritation, dyspnea, sore throat, light headedness, dizziness, mouth irritation, nausea, indigestion, mouth ulcers, or vertigo. Only the first event for each participant in each category was counted. At 12 weeks, the intervention group had experienced 120 (94%) and the control group had experienced 118 (93%). | | | | | | | | | |
| **Adverse events: Serious adverse events (follow up: 6 months)^a,b,c^**  Assessed with: self-reported | | | | | | | | | | | | | | | | |
| 657  (1 RCT) | not serious ^d^ | not serious ^e^ | not serious ^f^ | serious ^n^ | none ^h^ | ⨁⨁⨁◯  MODERATE | Serious adverse events included death, life threatening illness, admission to hospital or prolongation of hospital stay persistent or significant disability or incapacity, congenital abnormality, or other medically important events. At 6 months, the intervention group had experienced 27 (20%) and the control group had experienced 5 (14%). | | | | | | | | | |
| **Adverse events: Any non-serious event (follow up: 6 months)^a,b,c^**  Assessed with: self-reported | | | | | | | | | | | | | | | | |
| 657  (1 RCT) | not serious ^d^ | not serious ^e^ | not serious ^f^ | serious ^n^ | none ^h^ | ⨁⨁⨁◯  MODERATE | At 6 months, the intervention group had experienced 110 (80%) and the control group had experienced 31 (86%). | | | | | | | | | |
| **Adverse events: Side effects likely to be related to e-cig use (follow up: 3 & 6 months)^i,j,k^**  Assessed with: self-reported | | | | | | | | | | | | | | | | |
| 140  (1 RCT) | very serious ^l^ | not serious ^e^ | not serious ^f^ | very serious ^m^ | none ^h^ | ⨁◯◯◯  VERY LOW | At 3 months, 5.7% of the intervention group had experienced side effects (10% burning throat, 1.4% cough, 1.4% headache, 1.4% stomach-ache) and 2.9% in the control group (2.9% burning throat).  At 6 months, 15.9% of the intervention group had experienced side effects (5.8% burning throat, 5.8% cough, 1.4% headache, 4.3% insomnia, 1.4% stomach-ache) and 5.6% in the control group (2.8% burning throat, 7% cough, 1.4% headache, 4.2% insomnia). | | | | | | | | | |

#### Explanations

1. Study participants included smokers motivated to quit.
2. All participants were referred to Quitline, a low intensity behavioural support via voluntary telephone counselling.
3. Elusion e-cigarettes labelled 16 mg (tested 10-16 mg nicotine per mL) for 1 week before participants chosen quit day until 12 weeks after their chosen quit day (intervention), Elusion e-cigarettes with 0 mg for 1 week before participants chosen quit day until 12 weeks after their chosen quit day (control).
4. Issues of bias not substantive enough to warrant downrating.
5. One study only, we do not downrate this domain.
6. No indirectness, we do not downrate this domain.
7. The optimal information size was not met and inadequate sample size (<2000 participants). Confidence interval encompasses both harm and benefit. We downrate this domain by -2.0.
8. Although few studies, literature search was comprehensive. No suspicion of suppression of results of other studies. We do not downrate this domain.
9. Population included individuals who decided to participate in a screening program with a high motivation to stop smoking.
10. All participants received a 3-month cessation program that included a cognitive-behavioural intervention that aimed to support participants in changing their behaviour and improving motivation to quit.
11. Each participant received an e-cigarette kit and 12 10-mL liquid cartridges (8 mg/mL nicotine concentration). During the first week, participants could use the e-cigarettes
12. High risk for incomplete outcome data and selective outcome reporting, unclear for blinding of outcome assessors and allocation concealment. We downrate by -2.0.
13. Unable to assess confidence intervals and the optimal information size. Inadequate sample size (<400 participants for a continuous outcome). We downrate this domain by -2.0.
14. The optimal information size was not met and inadequate sample size (<2000 participants). Confidence interval encompasses both harm and benefit. We downrate this domain by -1.0.
15. Study enrolled adults motivated to quit smoking.
16. All treatment groups received relapse prevention counseling (minimum 30 minutes at baseline, 10 minutes during telephone follow-ups, and 15-20 minutes at clinic visits).
17. Participants randomized to e-cigarettes were supplied with 12 weeks of e-cigarettes (15 or 0 mg nicotine/mL).
18. High risk for blinding of participants/personnel, unclear for blinding of outcome assessors. We downrate by -2.0.
19. No indirectness, we do not downrate this domain
20. Unable to assess confidence intervals and the optimal information size. Inadequate sample size (<400 participants for a continuous outcome). We downrate this domain by -2.0.
21. Confidence interval encompasses benefit. Unable to access optimal information size. Inadequate sample size (<400 participants for a continuous outcome). We downrate this domain by -1.0.
22. One study only, we do not downrate this domain.

| Summary of findings: | | | | | | |  |
| --- | --- | --- | --- | --- | --- | --- | --- |
| **E-cigarette with nicotine and behavioural support compared to e-cigarette with no nicotine and behavioural support in smokers willing to quit** | | | | | | | |
| **Patient or population**: Smokers willing to quit  **Setting**: Mixed (lung cancer screening program, academic research setting, community)  **Intervention**: Electronic cigarettes with nicotine and behavioural support  **Comparison**: Electronic cigarettes with no nicotine and behavioural support | | | | | | | |
| Outcomes | **Anticipated absolute effects^*^** (95% CI) | | Relative effect (95% CI) | № of participants  (studies) | Certainty of the evidence (GRADE) | Comments | |
|  | **Risk with e-cigarettes with no nicotine and behavioural support** | **Risk with e-cigarettes with nicotine and behavioural support** |  |  |  |  |  |
| Tobacco use abstinence: Continuous smoking abstinence (ITT analysis)^a,b,c^  Assessed with: self-reported, allowing ≤5 cigarettes in total, eCO of <10ppm  Follow up: 6 months | 41 per 1,000 | 32 more per 1,000  (from 19 fewer to 196 more) | **RR 1.77**  (0.54 to 5.77) | 657  (1 RCT) | ⨁⨁⨁◯  MODERATE^d,e,f,g,h^ | Date of last search: September 2020. | |
| Tobacco use abstinence: 7-day point prevalence abstinence^a,b,c^  Assessed with: self-reported no smoking of tobacco cigarettes in the past 7 days)  Follow up: 6 months | 219 per 1,000 | 9 fewer per 1,000  (from 90 fewer to 125 more) | **RR 0.96**  (0.59 to 1.57 | 657  (1 RCT) | ⨁⨁⨁◯  MODERATE^d,e,f,g,h^ | Date of last search: September 2020. | |
| Tobacco use abstinence: Continuous smoking abstinence^i,j,k^  Assessed with: self-reported, eCOC verified ≤7ppm  Follow up: 6 months | 157 per 1,000 | 28 more per 1,000  (from 68 fewer to 229 more) | **RR 1.18**  (0.57 to 2.46) | 140  (1 RCT) | ⨁◯◯◯  VERY LOW^l,e,f,m,h^ | Date of last search: September 2020. | |
| Reduction in tobacco smoking frequency/quantity: Proportion of participants who reduced daily cigarettes by 50% or greater^a,b,c^  Assessed with: self-reported  Follow up: 6 months | 452 per 1,000 | 118 more per 1,000  (from 18 fewer to 298 more | **RR 1.26**  (0.96 to 1.66) | 657  (1 RCT) | ⨁⨁⨁◯  MODERATE^d,e,f,n,h^ | Date of last search: September 2020. | |
| Reduction in tobacco smoking frequency/quantity: Change in mean number of daily cigarettes smoked since baseline^o,p,q^  Assessed with: self-reported  Follow up: 24 weeks | At 24 weeks, the intervention group had a -10.7 change in mean number of daily cigarettes smoked since baseline and the control group had -9.1. SD were not reported. | | **-** | 249  (1 RCT) | ⨁◯◯◯  VERY LOW^r,e,s,t,h^ | Date of last search: September 2020. | |
| Reduction in tobacco smoking frequency/quantity: Number of daily cigarettes smoked^i,j,k^  Assessed with: self-reported  Follow up: 6 months | The mean number of daily cigarettes smoked was 14.03 (SD 7.92) | MD 3.02 lower  (5.42 lower to 0.62 lower) | **-** | 140  (1 RCT) | ⨁◯◯◯  VERY LOW^l,e,f,u,h^ | Date of last search: September 2020. | |
| Reduction in tobacco smoking frequency/quantity: eCO levels^i,j,k^  Assessed with: measured with Bedfont Micro Smokerlyzers (Bedfont Scientific, Maidstone, UK)  Follow up: 6 months | The mean change in eCO levels was 15.28 (SD 11.43) | MD 3.27 higher  (6.56 lower to 0.02 higher) | **-** | 140  (1 RCT) | ⨁◯◯◯  VERY LOW^l,e,f,u,h^ | Date of last search: September 2020. | |
| Adverse events: Serious adverse events^o,p,q^  Assessed with: self-reported  Follow up: 12 weeks | Serious adverse events were adjudicated by an end points evaluation committee and include death, respiratory, cardiovascular, neuropsychiatric or other events. At 12 weeks, the intervention group had experienced 1 (0.8%) and the control group had experienced 4 (3.1%). | | **-** | 249  (1 RCT) | ⨁◯◯◯  VERY LOW^r,e,s,t,v^ | Date of last search: September 2020. | |
| Adverse events: Serious adverse events^o,p,q^  Assessed with: self-reported  Follow up: 12 to 24 weeks | Serious adverse events were adjudicated by an end points evaluation committee and include death, respiratory, cardiovascular, neuropsychiatric or other events. At 12 to 24 weeks, the intervention group had experienced 2 (1.6%) and the control group had experienced 2 (1.6%). | | **-** | 249  (1 RCT) | ⨁◯◯◯  VERY LOW^r,e,s,t,v^ | Date of last search: September 2020. | |
| Adverse events: Mild adverse events^o,p,q^  Assessed with: self-reported  Follow up: 12 weeks | Mild adverse events included cough, dry mouth, headache, rhinitis, throat irritation, dyspnea, sore throat, light headedness, dizziness, mouth irritation, nausea, indigestion, mouth ulcers, or vertigo. Only the first event for each participant in each category was counted. At 12 weeks, the intervention group had experienced 120 (94%) and the control group had experienced 118 (93%). | | **-** | 249  (1 RCT) | ⨁◯◯◯  VERY LOW^r,e,s,t,v^ | Date of last search: September 2020. | |
| Adverse events: Any non-serious event^a,b,c^  Assessed with: self-reported  Follow up: 6 months | Serious adverse events included death, life threatening illness, admission to hospital or prolongation of hospital stay persistent or significant disability or incapacity, congenital abnormality, or other medically important events. At 6 months, the intervention group had experienced 27 (20%) and the control group had experienced 5 (14%).  At 6 months, the intervention group had experienced 110 (80%) and the control group had experienced 31 (86%). | | **-** | 657  (1 RCT) | ⨁⨁⨁◯  MODERATE^d,e,f,n,h^ | Date of last search: September 2020. | |
| Adverse events: Side effects likely to be related to e-cig use^i,j,k^  Assessed with: self-reported  Follow up: 3 & 6 months | At 3 months, 5.7% of the intervention group had experienced side effects (10% burning throat, 1.4% cough, 1.4% headache, 1.4% stomach-ache) and 2.9% in the control group (2.9% burning throat).  At 6 months, 15.9% of the intervention group had experienced side effects (5.8% burning throat, 5.8% cough, 1.4% headache, 4.3% insomnia, 1.4% stomach-ache) and 5.6% in the control group (2.8% burning throat, 7% cough, 1.4% headache, 4.2% insomnia). | | **-** | 140  (1 RCT) | ⨁◯◯◯  VERY LOW^l,e,f,m,h^ | Date of last search: September 2020. | |
| ***The risk in the intervention group** (and its 95% confidence interval) is based on the assumed risk in the comparison group and the **relative effect** of the intervention (and its 95% CI).  **CI:** Confidence interval; **RR:** Risk ratio | | | | | | | |
| **GRADE Working Group grades of evidence** **High certainty:** We are very confident that the true effect lies close to that of the estimate of the effect **Moderate certainty:** We are moderately confident in the effect estimate: The true effect is likely to be close to the estimate of the effect, but there is a possibility that it is substantially different **Low certainty:** Our confidence in the effect estimate is limited: The true effect may be substantially different from the estimate of the effect **Very low certainty:** We have very little confidence in the effect estimate: The true effect is likely to be substantially different from the estimate of effect | | | | | | | |

#### Explanations

1. Study participants included smokers motivated to quit.
2. All participants were referred to Quitline, a low intensity behavioural support via voluntary telephone counselling.
3. Elusion e-cigarettes labelled 16 mg (tested 10-16 mg nicotine per mL) for 1 week before participants chosen quit day until 12 weeks after their chosen quit day (intervention), Elusion e-cigarettes with 0 mg for 1 week before participants chosen quit day until 12 weeks after their chosen quit day (control).
4. Issues of bias not substantive enough to warrant downrating.
5. One study only, we do not downrate this domain.
6. No indirectness, we do not downrate this domain.
7. The optimal information size was not met and inadequate sample size (<2000 participants). Confidence interval encompasses both harm and benefit. We downrate this domain by -2.0.
8. Although few studies, literature search was comprehensive. No suspicion of suppression of results of other studies. We do not downrate this domain.
9. Population included individuals who decided to participate in a screening program with a high motivation to stop smoking.
10. All participants received a 3-month cessation program that included a cognitive-behavioural intervention that aimed to support participants in changing their behaviour and improving motivation to quit.
11. Each participant received an e-cigarette kit and 12 10-mL liquid cartridges (8 mg/mL nicotine concentration). During the first week, participants could use the e-cigarettes
12. High risk for incomplete outcome data and selective outcome reporting, unclear for blinding of outcome assessors and allocation concealment. We downrate by -2.0.
13. Unable to assess confidence intervals and the optimal information size. Inadequate sample size (<400 participants for a continuous outcome). We downrate this domain by -2.0.
14. The optimal information size was not met and inadequate sample size (<2000 participants). Confidence interval encompasses both harm and benefit. We downrate this domain by -1.0.
15. Study enrolled adults motivated to quit smoking.
16. All treatment groups received relapse prevention counseling (minimum 30 minutes at baseline, 10 minutes during telephone follow-ups, and 15-20 minutes at clinic visits).
17. Participants randomized to e-cigarettes were supplied with 12 weeks of e-cigarettes (15 or 0 mg nicotine/mL).
18. High risk for blinding of participants/personnel, unclear for blinding of outcome assessors. We downrate by -2.0.
19. No indirectness, we do not downrate this domain
20. Unable to assess confidence intervals and the optimal information size. Inadequate sample size (<400 participants for a continuous outcome). We downrate this domain by -2.0.
21. Confidence interval encompasses benefit. Unable to access optimal information size. Inadequate sample size (<400 participants for a continuous outcome). We downrate this domain by -1.0.
22. One study only, we do not downrate this domain.

## Appendix H Table 7. E-cigarette with nicotine + other smoking cessation treatment vs no intervention + other smoking cessation treatment: Smoking cessation, reduction, and adverse events

E-cigarette with nicotine + other smoking cessation treatment (behavioural support) vs No intervention + other smoking cessation treatment (behavioural support)

**Bibliography:** Lucchiari 2020; Eisenberg 2020; Date of last search September 2020

| **Certainty assessment** | | | | | | | | **Summary of findings** | | | | | | | | |
| --- | --- | --- | --- | --- | --- | --- | --- | --- | --- | --- | --- | --- | --- | --- | --- | --- |
| **№ of participants (studies) Follow-up** | **Risk of bias** | **Inconsistency** | **Indirectness** | **Imprecision** | **Publication bias** | **Overall certainty of evidence** | **Study event rates (%)** | | | | **Relative effect (95% CI)** | | **Anticipated absolute effects** | | | |
|  |  |  |  |  |  |  | **e-cigarettes with nicotine + other treatment** | | **other treatment** | |  |  | **Risk with other treatment** | | | **Risk difference with e-cigarettes with nicotine + other treatment** |
| **Tobacco use abstinence: Continuous smoking abstinence (follow up: 6 months)^a,b,c^**  Assessed with: self-reported, eCO^C^ verified ≤7ppm | | | | | | | | | | | | | | | | |
| 140  (1 RCT) | very serious ^d^ | not serious ^e^ | not serious ^f^ | very serious ^g^ | none ^h^ | ⨁◯◯◯  VERY LOW | 13/70 (18.6%) | | 7/70  (10.0%) | | **RR 1.86** (0.79 to 4.38) | | 100 per 1,000 | | | **86 more per 1,000** (from 21 fewer to 338 more) |
| **Reduction in tobacco smoking frequency/quantity: Change in mean number of daily cigarettes smoked since baseline (follow up: 24 weeks)^i,j,k^**  Assessed with: self-reported | | | | | | | | | | | | | | | | |
| 249  (1 RCT) | very serious ^l^ | not serious ^e^ | not serious ^f^ | very serious ^m^ | none ^h^ | ⨁◯◯◯  VERY LOW | At 24 weeks, the intervention group had a -10.7 change in mean number of daily cigarettes smoked since baseline and the control group had -5.5. SD were not reported. | | | | | | | | | |
| **Reduction in tobacco smoking frequency/quantity: Number of daily cigarettes smoked (follow up: 6 months)^a,b,c^**  Assessed with: self-reported | | | | | | | | | | | | | | | | |
| 140  (1 RCT) | very serious ^d^ | not serious ^e^ | not serious ^f^ | serious ^n^ | none ^h^ | ⨁◯◯◯  VERY LOW | 70 | | 70 | | **-** | | The mean number of daily cigarettes smoked was 13.45 (SD 6.49) | | | **MD 2.44 lower** (4.59 lower to 0.29 lower) |
| **Reduction in tobacco smoking frequency/quantity: eCO levels (follow up: 6 months)^a,b,c^**  Assessed with: measured with Bedfont Micro Smokerlyzers (Bedfont Scientific, Maidstone, UK) | | | | | | | | | | | | | | | | |
| 140  (1 RCT) | very serious ^d^ | not serious ^e^ | not serious ^f^ | serious ^n^ | none ^h^ | ⨁◯◯◯  VERY LOW | 70 | | | 70 | | - | | The mean change in eCO levels was 16.52 (SD 10.24) | **MD 4.51 higher** (1.42 higher to 7.60 higher) | |
| **Adverse events: Serious adverse events (follow up: 12 weeks)^i,j,k^**  Assessed with: self-reported | | | | | | | | | | | | | | | | |
| 249  (1 RCT) | very serious ^l^ | not serious ^e^ | not serious ^f^ | very serious ^m^ | none ^h^ | ⨁◯◯◯  VERY LOW | Serious adverse events were adjudicated by an end points evaluation committee and include death, respiratory, cardiovascular, neuropsychiatric, or other events. At 12 weeks, the intervention group had experienced 1 (0.8%) and the control group had experienced 2 (1.7%). | | | | | | | | | |
| **Adverse events: Serious adverse events (follow up: 12 to 24 weeks)^i,j,k^**  Assessed with: self-reported | | | | | | | | | | | | | | | | |
| 249  (1 RCT) | very serious ^l^ | not serious ^e^ | not serious ^f^ | very serious ^m^ | none ^h^ | ⨁◯◯◯  VERY LOW | Serious adverse events were adjudicated by an end points evaluation committee and include death, respiratory, cardiovascular, neuropsychiatric, or other events. At 12 to 24 weeks, the intervention group had experienced 2 (1.6%) and the control group had experienced 2 (1.7%). | | | | | | | | | |
| **Adverse events: Mild adverse events (follow up: 12 weeks)^i,j,k^**  Assessed with: self-reported | | | | | | | | | | | | | | | | |
| 249  (1 RCT) | very serious ^l^ | not serious ^e^ | not serious ^f^ | very serious ^m^ | none ^h^ | ⨁◯◯◯  VERY LOW | Mild adverse events included cough, dry mouth, headache, rhinitis, throat irritation, dyspnea, sore throat, light headedness, dizziness, mouth irritation, nausea, indigestion, mouth ulcers, or vertigo. Only the first event for each participant in each category was counted. At 12 weeks, the intervention group had experienced 120 (94%) and the control group had experienced 88 (73%). | | | | | | | | | |

#### Explanations

1. Population included individuals who decided to participate in a screening program with a high motivation to stop smoking.
2. All participants received a 3-month cessation program that included a cognitive-behavioural intervention that aimed to support participants in changing their behaviour and improving motivation to quit.
3. Each participant received an e-cigarette kit and 12 10-mL liquid cartridges (8 mg/mL nicotine concentration). During the first week, participants could use the e-cigarettes ad libitum. After the first week, they were asked to use the e-cigarette for the next 11 weeks.
4. High risk for incomplete outcome data and selective outcome reporting, unclear for blinding of outcome assessors and allocation concealment. We downrate by -2.0.
5. One study only, we do not downrate this domain.
6. No indirectness, we do not downrate this domain.
7. The optimal information size was not met (total of 20 events) and inadequate sample size (<2000 participants). Confidence interval encompasses both harm and benefit. We downrate this domain by -2.0.
8. Although few studies, literature search was comprehensive. No suspicion of suppression of results of other studies. We do not downrate this domain.
9. Study enrolled adults motivated to quit smoking.
10. All treatment groups received relapse prevention counseling (minimum 30 minutes at baseline, 10 minutes during telephone follow-ups, and 15-20 minutes at clinic visits).
11. Participants randomized to e-cigarettes were supplied with 12 weeks of e-cigarettes (15 or 0 mg nicotine/mL).
12. High risk for blinding of participants/personnel, unclear for blinding of outcome assessors. We downrate by -2.0.
13. Unable to assess confidence intervals and the optimal information size. Inadequate sample size (<400 participants for a continuous outcome). We downrate this domain by -2.0.
14. Confidence interval encompasses benefit. Unable to access optimal information size. Inadequate sample size (<400 participants for a continuous outcome). We downrate this domain by -1.0.

| Summary of findings: | | | | | | |  |
| --- | --- | --- | --- | --- | --- | --- | --- |
| **E-cigarette with nicotine and behavioural support compared to no intervention and behavioural support in smokers willing to quit** | | | | | | | |
| **Patient or population**: Smokers willing to quit  **Setting**: Mixed (lung cancer screening program, academic research setting)  **Intervention**: Electronic cigarettes with nicotine and behavioural support  **Comparison**: No intervention and behavioural support | | | | | | | |
| Outcomes | **Anticipated absolute effects^*^** (95% CI) | | Relative effect (95% CI) | № of participants  (studies) | Certainty of the evidence (GRADE) | Comments | |
|  | **Risk with no intervention and behavioural support** | **Risk with e-cigarettes with nicotine and behavioural support** |  |  |  |  |  |
| Tobacco use abstinence: Continuous smoking abstinence^a,b,c^  Assessed with: self-reported, eCO verified ≤7ppm  Follow up: 6 months | 100 per 1,000 | 86 more per 1,000  (from 21 fewer to 338 more) | **RR 1.86**  (0.79 to 4.38) | 140  (1 RCT) | ⨁◯◯◯  VERY LOW^d,e,f,g,h^ | Date of last search: September 2020. | |
| Reduction in tobacco smoking frequency/quantity: Change in mean number of daily cigarettes smoked since baseline^i,j,k^  Assessed with: self-reported  Follow up: 24 weeks | At 24 weeks, the intervention group had a -10.7 change in mean number of daily cigarettes smoked since baseline and the control group had -5.5. SD were not reported. | | **-** | 249  (1 RCT) | ⨁◯◯◯  VERY LOW^e,f,h,l,m^ | Date of last search: September 2020. | |
| Reduction in tobacco smoking frequency/quantity: Number of daily cigarettes smoked^a,b,c^  Assessed with: self-reported  Follow up: 6 months | The mean number of daily cigarettes smoked was 13.45 (SD 6.49) | MD 2.44 lower  (4.59 lower to 0.29 lower) | **-** | 140  (1 RCT) | ⨁◯◯◯  VERY LOW^d,e,f,h,n^ | Date of last search: September 2020. | |
| Reduction in tobacco smoking frequency/quantity: eCO levels^a,b,c^  Assessed with: measured with Bedfont Micro Smokerlyzers (Bedfont Scientific, Maidstone, UK)  Follow up: 6 months | The mean change in eCO levels was 6.52 (SD 10.24) | MD 5.49 higher  (2.43 higher to 8.55 higher) | **-** | 140  (1 RCT) | ⨁◯◯◯  VERY LOW^d,e,f,h,n^ | Date of last search: September 2020. | |
| Adverse events: Serious adverse events^i,j,k^  Assessed with: self-reported  Follow up: 12 weeks | Serious adverse events were adjudicated by an end points evaluation committee and include death, respiratory, cardiovascular, neuropsychiatric or other events. At 12 weeks, the intervention group had experienced 1 (0.8%) and the control group had experienced 2 (1.7%). | | **-** | 249  (1 RCT) | ⨁◯◯◯  VERY LOW^e,f,h,l,m^ | Date of last search: September 2020. | |
| Adverse events: Serious adverse events^i,j,k^  Assessed with: self-reported  Follow up: 12 to 24 weeks | Serious adverse events were adjudicated by an end points evaluation committee and include death, respiratory, cardiovascular, neuropsychiatric or other events. At 12 to 24 weeks, the intervention group had experienced 2 (1.6%) and the control group had experienced 2 (1.7%). | | **-** | 249  (1 RCT) | ⨁◯◯◯  VERY LOW^e,f,h,l,m^ | Date of last search: September 2020. | |
| Adverse events: Mild adverse events^i,j,k^  Assessed with: self-reported  Follow up: 12 weeks | Mild adverse events included cough, dry mouth, headache, rhinitis, throat irritation, dyspnea, sore throat, light headedness, dizziness, mouth irritation, nausea, indigestion, mouth ulcers, or vertigo. Only the first event for each participant in each category was counted. At 12 weeks, the intervention group had experienced 120 (94%) and the control group had experienced 88 (73%). | | **-** | 249  (1 RCT) | ⨁◯◯◯  VERY LOW^e,f,h,l,m^ | Date of last search: September 2020. | |
| ***The risk in the intervention group** (and its 95% confidence interval) is based on the assumed risk in the comparison group and the **relative effect** of the intervention (and its 95% CI).  **CI:** Confidence interval; **RR:** Risk ratio | | | | | | | |
| **GRADE Working Group grades of evidence** **High certainty:** We are very confident that the true effect lies close to that of the estimate of the effect **Moderate certainty:** We are moderately confident in the effect estimate: The true effect is likely to be close to the estimate of the effect, but there is a possibility that it is substantially different **Low certainty:** Our confidence in the effect estimate is limited: The true effect may be substantially different from the estimate of the effect **Very low certainty:** We have very little confidence in the effect estimate: The true effect is likely to be substantially different from the estimate of effect | | | | | | | |

#### Explanations

1. Population included individuals who decided to participate in a screening program with a high motivation to stop smoking.
2. All participants received a 3-month cessation program that included a cognitive-behavioural intervention that aimed to support participants in changing their behaviour and improving motivation to quit.
3. Each participant received an e-cigarette kit and 12 10-mL liquid cartridges (8 mg/mL nicotine concentration). During the first week, participants could use the e-cigarettes ad libitum. After the first week, they were asked to use the e-cigarette for the next 11 weeks.
4. High risk for incomplete outcome data and selective outcome reporting, unclear for blinding of outcome assessors and allocation concealment. We downrate by -2.0.
5. One study only, we do not downrate this domain.
6. No indirectness, we do not downrate this domain.
7. The optimal information size was not met (total of 20 events) and inadequate sample size (<2000 participants). Confidence interval encompasses both harm and benefit. We downrate this domain by -2.0.
8. Although few studies, literature search was comprehensive. No suspicion of suppression of results of other studies. We do not downrate this domain.
9. Study enrolled adults motivated to quit smoking.
10. All treatment groups received relapse prevention counseling (minimum 30 minutes at baseline, 10 minutes during telephone follow-ups, and 15-20 minutes at clinic visits).
11. Participants randomized to e-cigarettes were supplied with 12 weeks of e-cigarettes (15 or 0 mg nicotine/mL).
12. High risk for blinding of participants/personnel, unclear for blinding of outcome assessors. We downrate by -2.0.
13. Unable to assess confidence intervals and the optimal information size. Inadequate sample size (<400 participants for a continuous outcome). We downrate this domain by -2.0.
14. Confidence interval encompasses benefit. Unable to access optimal information size. Inadequate sample size (<400 participants for a continuous outcome). We downrate this domain by -1.0.

## Appendix H Table 8. E-cigarette with nicotine + other smoking cessation treatment + standard care vs. E-cigarette with no nicotine + other smoking cessation treatment + standard care: Smoking cessation, reduction, adverse events, possible adverse outcomes

E-cigarette with nicotine + other smoking cessation treatment (behavioural support) + standard care (nicotine patch) vs. E-cigarette with no nicotine + other smoking cessation treatment (behavioural support) + standard care (nicotine patch)

**Bibliography:** Walker 2020; Date of last search September 2020

| **Certainty assessment** | | | | | | | | **Summary of findings** | | | | | | | |
| --- | --- | --- | --- | --- | --- | --- | --- | --- | --- | --- | --- | --- | --- | --- | --- |
| **№ of participants (studies) Follow-up** | **Risk of bias** | **Inconsistency** | **Indirectness** | **Imprecision** | **Publication bias** | **Overall certainty of evidence** | **Study event rates (%)** | | | | **Relative effect (95% CI)** | | **Anticipated absolute effects** | | |
|  |  |  |  |  |  |  | **e-cigarettes with nicotine + other treatment + standard care** | | **e-cigarettes with no nicotine + other treatment + standard care** | |  |  | **Risk with e-cigarettes with no nicotine + other treatment + standard care** | | **Risk difference with e-cigarettes with nicotine + other treatment + standard care** |
| **Tobacco use abstinence: Continuous smoking abstinence (follow up: 6 months)^a,b,c^**  Assessed with: self-reported, allowing ≤5 cigarettes in total, eCOE verified ≤9ppm | | | | | | | | | | | | | | | |
| 999  (1 RCT) | not serious ^d^ | not serious ^e^ | not serious ^f^ | serious ^g^ | none ^h^ | ⨁⨁⨁◯  MODERATE | 35/500 (7.0%) | | 20/499  (4.0%) | | **RR 1.75** (1.02 to 2.98) | | 40 per 1,000 | | **30 more per 1,000** (from 1 more to 79 more) |
| **Tobacco use abstinence: Continuous smoking abstinence (follow up: 6 months) ^a,b,c^**  Assessed with: self-reported | | | | | | | | | | | | | | | |
| 999  (1 RCT) | not serious ^d^ | not serious ^e^ | not serious ^f^ | serious ^g^ | none ^h^ | ⨁⨁⨁◯  MODERATE | 89/500 (17.8%) | | 53/499  (10.6%) | | **RR 1.68** (1.22 to 2.30) | | 106 per 1,000 | | **72 more per 1,000** (from 23 more to 138 more) |
| **Tobacco use abstinence: 7-day point prevalence abstinence (follow up: 6 months)^a,b,c^**  Assessed with: self-reported | | | | | | | | | | | | | | | |
| 999  (1 RCT) | not serious ^d^ | not serious ^e^ | not serious ^f^ | serious ^g^ | none ^h^ | ⨁⨁⨁◯  MODERATE | 119/500 (23.8%) | | 83/499  (16.6%) | | **RR 1.43** (1.11 to 1.84) | | 166 per 1,000 | | **72 more per 1,000** (from 18 more to 140 more) |
| **Reduction in tobacco smoking frequency/quantity: Change from baseline in the mean number of cigarettes smoked per day (follow up: 6 months) ^a,b,c^**  Assessed with: self-reported | | | | | | | | | | | | | | | |
| 999  (1 RCT) | not serious ^d^ | not serious ^e^ | not serious ^f^ | serious ^g^ | none ^h^ | ⨁⨁⨁◯  MODERATE | 500 | | 499 | | - | | The mean number of cigarettes smoked per day was 8.3 (SD 0.4) | | **MD 0 lower** (0.06 lower to 0.06 higher) |
| **Reduction in tobacco smoking frequency/quantity: ≥50% reduction in the number of cigarettes/day since baseline (follow up: 6 months) ^a,b,c^**  Assessed with: self-reported | | | | | | | | | | | | | | | |
| 999  (1 RCT) | not serious ^d^ | not serious ^e^ | not serious ^f^ | serious ^g^ | none ^h^ | ⨁⨁⨁◯  MODERATE | 218/500 (43.6%) | | 199/499  (39.9%) | | **RR 1.15** (0.99 to 1.33) | | 399 per 1,000 | | **60 more per 1,000** (from 4 fewer to 132 more) |
| **Adverse events: Participants with a serious adverse event (follow up: 6 months) ^a,b,c^**  Assessed with: self-reported | | | | | | | | | | | | | | | |
| 999  (1 RCT) | not serious ^d^ | not serious ^e^ | not serious ^f^ | very serious ^i^ | none ^h^ | ⨁⨁◯◯ LOW | 16/500 (3.2%) | | | 22/499  (4.4%) | | **RR 0.73** (0.39 to 1.37) | | 44 per 1,000 | **12 fewer per 1,000** (from 27 fewer to 16 more) |
| **Adverse events: Total serious adverse events (follow up: 6 months) ^a,b,c^**  Assessed with: self-reported | | | | | | | | | | | | | | | |
| 999  (1 RCT) | not serious ^d^ | not serious ^e^ | not serious ^f^ | serious ^g^ | none ^h^ | ⨁⨁⨁◯  MODERATE | At 6 months, there was a total of 18 SAEs in the intervention group (11 hospitalizations, 4 otherwise medically important, 2 life-threatening, 1 persistent, significant disability or incapacity) and 27 in the control group (19 hospitalizations, 6 otherwise medically important, 1 life-threatening, 1 death). | | | | | | | | |
| **Possible adverse outcome: Change in BMI from baseline (follow up: 6 months) ^a,b,c^**  Assessed with: self-reported | | | | | | | | | | | | | | | |
| 999  (1 RCT) | not serious ^d^ | not serious ^e^ | not serious ^f^ | serious ^g^ | none ^h^ | ⨁⨁⨁◯  MODERATE | 500 | | | 499 | | - | | The mean change in BMI from baseline was -0.1 (SD 0.2) | **MD 0.3 lower** (0.32 lower to 0.28 lower) |
| **Possible adverse outcome: Change in weight from baseline (follow up: 6 months) ^a,b,c^**  Assessed with: self-reported | | | | | | | | | | | | | | | |
| 999  (1 RCT) | not serious ^d^ | not serious ^e^ | not serious ^f^ | serious ^g^ | none ^h^ | ⨁⨁⨁◯  MODERATE | 500 | | | 499 | | - | | The mean change in weight from baseline was -0.4 (SD 0.5) | **MD 0.7 lower** (0.76 lower to 0.64 lower) |
| **Possible adverse outcomes (follow up: 6 months) ^a,b,c^**  Assessed with: self-reported | | | | | | | | | | | | | | | |
| 999  (1 RCT) | not serious ^d^ | not serious ^e^ | not serious ^f^ | serious ^g^ | none ^h^ | ⨁⨁⨁◯  MODERATE | At 6 months, various possible adverse events were reported for the intervention and control groups:  vivid dreams 12 (4%) vs 11 (3%);  itchiness 12 (4%) vs 10 (3%);  redness, swollen at patch site 10 (3%) vs 11 (3%);  dry mouth or throat 10 (3%) vs 5 (2%);  cough 15 (4%) vs 4 (1%);  nausea 6 (2%) vs 4 (1%);  headache 6 (2%) vs 7 (2%); | | | | | | | | |

#### Explanations

1. Population included individuals who decided to participate in the trial with a high motivation to quit smoking in the next 2 weeks.
2. Along with Nicotine patches and/or nicotine-E-cig, all participants received a moderate-intensity behavioral support once a week for 6 weeks that aimed to provide 10-15 min of withdrawal-oriented behavioral support with some advice to the participant on using their allocated treatment.
3. Each participant received 14 weeks of 21 mg, 24h nicotine patches, plus an 18 mg/L nicotine e-cig, or patches plus a nicotine-free e-cig.
4. Issues of bias not substantive enough to warrant downrating
5. One study only, we do not downrate this domain.
6. No indirectness, we do not downrate this domain.
7. Unable to assess confidence intervals and the optimal information size. We downrate this domain by -1.0.
8. Search not completely comprehensive but study reports negative findings. We do not downrate this domain.
9. The optimal information size was not met and inadequate sample size (<2000 participants). Confidence interval encompasses both harm and benefit. We downrate this domain by -2.0.

| Summary of findings: | | | | | | |  |
| --- | --- | --- | --- | --- | --- | --- | --- |
| **E-cigarette with nicotine, behavioural support, and standard care (nicotine patch) compared to e-cigarette with no nicotine, behavioural support, and standard care (nicotine patch) in smokers willing to quit within the next 2 weeks** | | | | | | | |
| **Patient or population**: Smokers willing to quit within the next 2 weeks  **Setting**: Community  **Intervention**: Electronic cigarettes with nicotine, behavioural support, and standard care (nicotine patch)  **Comparison**: Electronic cigarettes with no nicotine, behavioural support, and standard care (nicotine patch) | | | | | | | |
| Outcomes | **Anticipated absolute effects^*^** (95% CI) | | Relative effect (95% CI) | № of participants  (studies) | Certainty of the evidence (GRADE) | Comments | |
|  | **Risk with e-cigarettes with no nicotine, behavioural support, and standard care (nicotine patch)** | **Risk with e-cigarettes with nicotine, behavioural support, and standard care (nicotine patch)** |  |  |  |  |  |
| Tobacco use abstinence: Continuous smoking abstinence^a,b,c^  Assessed with: self-reported, allowing ≤5 cigarettes in total, eCO verified ≤9ppm  Follow up: 6 months | 40 per 1,000 | 30 more per 1,000  (from 1 more to 79 more) | **RR 1.75**  (1.02 to 2.98) | 999  (1 RCT) | ⨁⨁⨁◯^d,e,f,g,h^  MODERATE | Date of last search: September 2020. | |
| Tobacco use abstinence: Continuous smoking abstinence^a,b,c^  Assessed with: self-reported  Follow up: 6 months | 106 per 1,000 | 72 more per 1,000  (from 23 more to 138 more) | **RR 1.68**  (1.22 to 2.30) | 999  (1 RCT) | ⨁⨁⨁◯ ^d,e,f,g,h^  MODERATE | Date of last search: September 2020. | |
| Tobacco use abstinence: 7-day point prevalence abstinence^a,b,c^  Assessed with: self-reported  Follow up: 6 months | 166 per 1,000 | 72 more per 1,000  (from 18 more to 140 more) | **RR 1.43**  (1.11 to 1.84) | 999  (1 RCT) | ⨁⨁⨁◯^d,e,f,g,h^  MODERATE | Date of last search: September 2020. | |
| Reduction in tobacco smoking frequency/quantity: Change from baseline in the mean number of cigarettes smoked per day^a,b,c^  Assessed with: self-reported  Follow up: 6 months | The mean number of cigarettes smoked per day was 8.3 (SD 0.4) | MD 0 lower  (0.06 lower to 0.06 higher) | - | 999  (1 RCT) | ⨁⨁⨁◯^d,e,f,g,h^  MODERATE | Date of last search: September 2020. | |
| Reduction in tobacco smoking frequency/quantity: ≥50% reduction in the number of cigarettes/day since baseline^a,b,c^  Assessed with: self-reported  Follow up: 6 months | 399 per 1,000 | 60 more per 1,000  (from 4 fewer to 132 more) | **RR 1.15**  (0.99 to 1.33) | 999  (1 RCT) | ⨁⨁⨁◯^d,e,f,g,h^  MODERATE | Date of last search: September 2020. | |
| Adverse events: Participants with a serious adverse event^a,b,c^  Assessed with: self-reported  Follow up: 6 months | 44 per 1,000 | 12 fewer per 1,000  (from 27 fewer to 16 more) | **RR 0.73**  (0.39 to 1.37) | 999  (1 RCT) | ⨁⨁◯◯^d,e,f,I,h^  LOW | Date of last search: September 2020. | |
| Adverse events: Total serious adverse events^a,b,c^  Assessed with: self-reported  Follow up: 6 months | At 6 months, there was a total of 18 SAEs in the intervention group (11 hospitalizations, 4 otherwise medically important, 2 life-threatening, 1 persistent, significant disability or incapacity) and 27 in the control group (19 hospitalizations, 6 otherwise medically important, 1 life-threatening, 1 death). | | **-** | 999  (1 RCT) | ⨁⨁⨁◯^d,e,f,g,h^  MODERATE | Date of last search: September 2020. | |
| Possible adverse outcome: Change in BMI from baseline^a,b,c^  Assessed with: self-reported  Follow up: 6 months | The mean change in BMI from baseline was -0.1 (SD 0.2) | MD 0.3 lower  (0.32 lower to 0.28 lower) | **-** | 999  (1 RCT) | ⨁⨁⨁◯^d,e,f,g,h^  MODERATE | Date of last search: September 2020. | |
| Possible adverse outcome: Change in weight from baseline^a,b,c^  Assessed with: self-reported  Follow up: 6 months | The mean change in weight from baseline was -0.4 (SD 0.5) | MD 0.7 lower  (0.76 lower to 0.64 lower) | **-** | 999  (1 RCT) | ⨁⨁⨁◯^d,e,f,g,h^  MODERATE | Date of last search: September 2020. | |
| Possible adverse outcomes^a,b,c^  Assessed with: self-reported  Follow up: 6 months | At 6 months, various possible adverse events were reported for the intervention and control groups:  vivid dreams 12 (4%) vs 11 (3%);  itchiness 12 (4%) vs 10 (3%);  redness, swollen at patch site 10 (3%) vs 11 (3%);  dry mouth or throat 10 (3%) vs 5 (2%);  cough 15 (4%) vs 4 (1%);  nausea 6 (2%) vs 4 (1%);  headache 6 (2%) vs 7 (2%). | | **-** | 999  (1 RCT) | ⨁⨁⨁◯^d,e,f,g,h^  MODERATE | Date of last search: September 2020. | |
| ***The risk in the intervention group** (and its 95% confidence interval) is based on the assumed risk in the comparison group and the **relative effect** of the intervention (and its 95% CI).  **CI:** Confidence interval; **RR:** Risk ratio | | | | | | | |
| **GRADE Working Group grades of evidence** **High certainty:** We are very confident that the true effect lies close to that of the estimate of the effect **Moderate certainty:** We are moderately confident in the effect estimate: The true effect is likely to be close to the estimate of the effect, but there is a possibility that it is substantially different **Low certainty:** Our confidence in the effect estimate is limited: The true effect may be substantially different from the estimate of the effect **Very low certainty:** We have very little confidence in the effect estimate: The true effect is likely to be substantially different from the estimate of effect | | | | | | | |

#### Explanations

1. Population included individuals who decided to participate in the trial with a high motivation to quit smoking in the next 2 weeks.
2. Along with Nicotine patches and/or nicotine-E-cig, all participants received a moderate-intensity behavioral support once a week for 6 weeks that aimed to provide 10-15 min of withdrawal-oriented behavioral support with some advice to the participant on using their allocated treatment.
3. Each participant received 14 weeks of 21 mg, 24h nicotine patches, plus an 18 mg/L nicotine e-cig, or patches plus a nicotine-free e-cig.
4. Issues of bias not substantive enough to warrant downrating
5. One study only, we do not downrate this domain.
6. No indirectness, we do not downrate this domain.
7. Unable to assess confidence intervals and the optimal information size. We downrate this domain by -1.0.
8. Search not completely comprehensive but study reports negative findings. We do not downrate this domain.
9. The optimal information size was not met and inadequate sample size (<2000 participants). Confidence interval encompasses both harm and benefit. We downrate this domain by -2.0.

## Appendix H Table 9. E-cigarette with nicotine + other smoking cessation treatment + standard care vs. Other smoking cessation treatment + standard care: Smoking cessation, reduction, adverse events, possible adverse outcomes

E-cigarette with nicotine + other smoking cessation treatment (behavioural support) + standard care (nicotine patch) vs. Other smoking cessation treatment (behavioural support) + standard care (nicotine patch)

**Bibliography:** Walker 2020; Date of last search September 2020

| **Certainty assessment** | | | | | | | | **Summary of findings** | | | | | | | | |
| --- | --- | --- | --- | --- | --- | --- | --- | --- | --- | --- | --- | --- | --- | --- | --- | --- |
| **№ of participants (studies) Follow-up** | **Risk of bias** | **Inconsistency** | **Indirectness** | **Imprecision** | **Publication bias** | **Overall certainty of evidence** | **Study event rates (%)** | | | | **Relative effect (95% CI)** | | **Anticipated absolute effects** | | | |
|  |  |  |  |  |  |  | **e-cigarettes with nicotine + other treatment + standard care** | | **other treatment + standard care** | |  |  | **Risk with other treatment + standard care** | | | **Risk difference with e-cigarettes with nicotine + other treatment + standard care** |
| **Tobacco use abstinence: Continuous smoking abstinence (follow up: 6 months)^a,b,c^**  Assessed with: self-reported, allowing ≤5 cigarettes in total, eCOE verified ≤9ppm | | | | | | | | | | | | | | | | |
| 625  (1 RCT) | not serious ^d^ | not serious ^e^ | not serious ^f^ | very serious ^g^ | none ^h^ | ⨁⨁◯◯ LOW | 35/500 (7.0%) | | 3/125  (2.4%) | | **RR 2.92** (0.91 to 9.33) | | 24 per 1,000 | | | **46 more per 1,000** (from 2 fewer to 200 more) |
| **Tobacco use abstinence: Continuous smoking abstinence (follow up: 6 months)^a,b,c^**  Assessed with: self-reported | | | | | | | | | | | | | | | | |
| 625  (1 RCT) | not serious ^d^ | not serious ^e^ | not serious ^f^ | very serious ^g^ | none ^h^ | ⨁⨁◯◯ LOW | 89/500 (17.8%) | | 10/125  (8.0%) | | **RR 2.23** (1.19 to 4.15) | | 80 per 1,000 | | | **98 more per 1,000** (from 15 more to 252 more) |
| **Tobacco use abstinence: 7-day point prevalence abstinence (follow up: 6 months)^a,b,c^**  Assessed with: self-reported | | | | | | | | | | | | | | | | |
| 625  (1 RCT) | not serious ^d^ | not serious ^e^ | not serious ^f^ | very serious ^g^ | none ^h^ | ⨁⨁◯◯ LOW | 119/500 (23.8%) | | 14/125  (11.2%) | | **RR 2.13** (1.27 to 3.57) | | 112 per 1,000 | | | **127 more per 1,000** (from 30 more to 288 more) |
| **Reduction in tobacco smoking frequency/quantity: Change from baseline in the mean number of cigarettes smoked per day (follow up: 6 months)^a,b,c^**  Assessed with: self-reported | | | | | | | | | | | | | | | | |
| 625  (1 RCT) | not serious ^d^ | not serious ^e^ | not serious ^f^ | serious ^i^ | none ^h^ | ⨁⨁⨁◯  MODERATE | 500 | | 125 | | - | | The mean number of cigarettes smoked per day was 8.6 (SD 1.0) | | | **MD 0 .3lower** (0.48 lower to 0.12 lower) |
| **Reduction in tobacco smoking frequency/quantity: ≥50% reduction in the number of cigarettes/day since baseline (follow up: 6 months)^a,b,c^**  Assessed with: self-reported | | | | | | | | | | | | | | | | |
| 625  (1 RCT) | not serious ^d^ | not serious ^e^ | not serious ^f^ | very serious ^g^ | none ^h^ | ⨁⨁◯◯ LOW | 218/500 (43.6%) | | 32/125  (25.6%) | | **RR 1.70** (1.24 to 2.33) | | 256 per 1,000 | | | **179 more per 1,000** (from 61 more to 340 more) |
| **Adverse events: Participants with a serious adverse event (follow up: 6 months)^a,b,c^**  Assessed with: self-reported | | | | | | | | | | | | | | | | |
| 625  (1 RCT) | not serious ^d^ | not serious ^e^ | not serious ^f^ | very serious ^g^ | none ^h^ | ⨁⨁◯◯ LOW | 16/500 (3.2%) | | | 3/125  (4.4%) | | **RR 1.33** (0.39 to 4.50) | | 24 per 1,000 | **8 fewer per 1,000** (from 15 fewer to 84 more) | |
| **Adverse events: Total serious adverse events (follow up: 6 months)^a,b,c^**  Assessed with: self-reported | | | | | | | | | | | | | | | | |
| 625  (1 RCT) | not serious ^d^ | not serious ^e^ | not serious ^f^ | serious ^i^ | none ^h^ | ⨁⨁⨁◯  MODERATE | At 6 months, there was a total of 18 SAEs in the intervention group (11 hospitalizations, 4 otherwise medically important, 2 life-threatening, 1 persistent, significant disability or incapacity) and 4 in the control group (3 hospitalizations, 1 otherwise medically important). | | | | | | | | | |
| **Possible adverse outcome: Change in BMI from baseline (follow up: 6 months)^a,b,c^**  Assessed with: self-reported | | | | | | | | | | | | | | | | |
| 625  (1 RCT) | not serious ^d^ | not serious ^e^ | not serious ^f^ | serious ^i^ | none ^h^ | ⨁⨁⨁◯  MODERATE | 500 | | | 499 | | - | | The mean change in BMI from baseline was 0.1 (SD 0.4) | **MD 0.5 lower** (0.57 lower to 0.43 lower) | |
| **Possible adverse outcome: Change in weight from baseline (follow up: 6 months)^a,b,c^**  Assessed with: self-reported | | | | | | | | | | | | | | | | |
| 625  (1 RCT) | not serious ^d^ | not serious ^e^ | not serious ^f^ | serious ^i^ | none ^h^ | ⨁⨁⨁◯  MODERATE | 500 | | | 499 | | - | | The mean change in weight from baseline was -0.4 (SD 1.0) | **MD 0.7 lower** (0.88 lower to 0.52 lower) | |
| **Possible adverse outcomes (follow up: 6 months)^a,b,c^**  Assessed with: self-reported | | | | | | | | | | | | | | | | |
| 625  (1 RCT) | not serious ^d^ | not serious ^e^ | not serious ^f^ | serious ^i^ | none ^h^ | ⨁⨁⨁◯  MODERATE | At 6 months, various possible adverse events were reported for the intervention and control groups:  vivid dreams 12 (4%) vs 6 (10%);  itchiness 12 (4%) vs 2 (3%);  redness, swollen at patch site 10 (3%) vs 5 (8%);  dry mouth or throat 10 (3%) vs 0 (0%);  cough 15 (4%) vs 0 (0%);  nausea 6 (2%) vs 2 (3%);  headache 6 (2%) vs 1 (2%); | | | | | | | | | |

#### Explanations

1. Population included individuals who decided to participate in the trial with a high motivation to quit smoking in the next 2 weeks.
2. Along with Nicotine patches and/or nicotine-E-cig, all participants received a moderate-intensity behavioral support once a week for 6 weeks that aimed to provide 10-15 min of withdrawal-oriented behavioral support with some advice to the participant on using their allocated treatment.
3. Each participant received 14 weeks of 21 mg, 24h nicotine patches, plus an 18 mg/L nicotine e-cig, or patches plus a nicotine-free e-cig.
4. Issues of bias not substantive enough to warrant downrating
5. One study only, we do not downrate this domain.
6. No indirectness, we do not downrate this domain.
7. The optimal information size was not met and inadequate sample size (<2000 participants). Confidence interval encompasses both harm and benefit. We downrate this domain by -2.0.
8. Search not completely comprehensive but study reports negative findings. We do not downrate this domain.
9. Unable to assess confidence intervals and the optimal information size. We downrate this domain by -1.0.

| Summary of findings: | | | | | | |  |
| --- | --- | --- | --- | --- | --- | --- | --- |
| **E-cigarette with nicotine, behavioural support, and standard care (nicotine patch) compared to behavioural support and standard care (nicotine patch) in smokers willing to quit within the next 2 weeks** | | | | | | | |
| **Patient or population**: Smokers willing to quit within the next 2 weeks  **Setting**: Community  **Intervention**: Electronic cigarettes with nicotine, behavioural support, and standard care (nicotine patch)  **Comparison**: Behavioural support and standard care (nicotine patch) | | | | | | | |
| Outcomes | **Anticipated absolute effects^*^** (95% CI) | | Relative effect (95% CI) | № of participants  (studies) | Certainty of the evidence (GRADE) | Comments | |
|  | **Risk with behavioural support and standard care (nicotine patch)** | **Risk with e-cigarettes with nicotine, behavioural support, and standard care (nicotine patch)** |  |  |  |  |  |
| Tobacco use abstinence: Continuous smoking abstinence^a,b,c^  Assessed with: self-reported, allowing ≤5 cigarettes in total, eCO verified ≤9ppm  Follow up: 6 months | 24 per 1,000 | 46 more per 1,000  (from 2 fewer to 200 more) | **RR 2.92**  (0.91 to 9.33) | 625  (1 RCT) | ⨁⨁◯◯^d,e,f,g,h^  LOW | Date of last search: September 2020. | |
| Tobacco use abstinence: Continuous smoking abstinence^a,b,c^  Assessed with: self-reported  Follow up: 6 months | 80 per 1,000 | 98 more per 1,000  (from 15 more to 252 more) | **RR 2.23**  (1.19 to 4.15) | 625  (1 RCT) | ⨁⨁◯◯ ^d,e,f,g,h^  LOW | Date of last search: September 2020. | |
| Tobacco use abstinence: 7-day point prevalence abstinence^a,b,c^  Assessed with: self-reported  Follow up: 6 months | 112 per 1,000 | 127 more per 1,000  (from 30 more to 288 more) | **RR 2.13**  (1.27 to 3.57) | 625  (1 RCT) | ⨁⨁◯◯ ^d,e,f,g,h^  LOW | Date of last search: September 2020. | |
| Reduction in tobacco smoking frequency/quantity: Change from baseline in the mean number of cigarettes smoked per day^a,b,c^  Assessed with: self-reported  Follow up: 6 months | The mean number of cigarettes smoked per day was 8.6 (SD 1.0) | MD 0.3 lower  (0.48 lower to 0.12 lower | - | 625  (1 RCT) | ⨁⨁◯◯^d,e,f,I,h^  LOW | Date of last search: September 2020. | |
| Reduction in tobacco smoking frequency/quantity: ≥50% reduction in the number of cigarettes/day since baseline^a,b,c^  Assessed with: self-reported  Follow up: 6 months | 256 per 1,000 | 179 more per 1,000  (from 61 more to 340 more) | **RR 1.70**  (1.24 to 2.33) | 625  (1 RCT) | ⨁⨁◯◯^d,e,f,g,h^  LOW | Date of last search: September 2020. | |
| Adverse events: Participants with a serious adverse event^a,b,c^  Assessed with: self-reported  Follow up: 6 months | 24 per 1,000 | 8 fewer per 1,000  (from 15 fewer to 84 more) | **RR 1.33**  (0.39 to 4.50) | 625  (1 RCT) | ⨁⨁◯◯ ^d,e,f,g,h^  LOW | Date of last search: September 2020. | |
| Adverse events: Total serious adverse events^a,b,c^  Assessed with: self-reported  Follow up: 6 months | At 6 months, there was a total of 18 SAEs in the intervention group (11 hospitalizations, 4 otherwise medically important, 2 life-threatening, 1 persistent, significant disability or incapacity) and 4 in the control group (3 hospitalizations, 1 otherwise medically important). | | **-** | 625  (1 RCT) | ⨁⨁⨁◯^d,e,f,I,h^  MODERATE | Date of last search: September 2020. | |
| Possible adverse outcome: Change in BMI from baseline^a,b,c^  Assessed with: self-reported  Follow up: 6 months | The mean change in BMI from baseline was 0.1 (SD 0.4) | MD 0.5 lower  (0.57 lower to 0.43 lower) | **-** | 625  (1 RCT) | ⨁⨁⨁◯ ^d,e,f,I,h^  MODERATE | Date of last search: September 2020. | |
| Possible adverse outcome: Change in weight from baseline^a,b,c^  Assessed with: self-reported  Follow up: 6 months | The mean change in weight from baseline was -0.4 (SD 1.0) | MD 0.7 lower  (0.88 lower to 0.52 lower) | **-** | 625  (1 RCT) | ⨁⨁⨁◯ ^d,e,f,I,h^  MODERATE | Date of last search: September 2020. | |
| Possible adverse outcomes^a,b,c^  Assessed with: self-reported  Follow up: 6 months | At 6 months, various possible adverse events were reported for the intervention and control groups:  vivid dreams 12 (4%) vs 6 (10%);  itchiness 12 (4%) vs 2 (3%);  redness, swollen at patch site 10 (3%) vs 5 (8%);  dry mouth or throat 10 (3%) vs 0 (0%);  cough 15 (4%) vs 0 (0%);  nausea 6 (2%) vs 2 (3%);  headache 6 (2%) vs 1 (2%); | | **-** | 625  (1 RCT) | ⨁⨁⨁◯ ^d,e,f,I,h^  MODERATE | Date of last search: September 2020. | |
| ***The risk in the intervention group** (and its 95% confidence interval) is based on the assumed risk in the comparison group and the **relative effect** of the intervention (and its 95% CI).  **CI:** Confidence interval; **RR:** Risk ratio | | | | | | | |
| **GRADE Working Group grades of evidence** **High certainty:** We are very confident that the true effect lies close to that of the estimate of the effect **Moderate certainty:** We are moderately confident in the effect estimate: The true effect is likely to be close to the estimate of the effect, but there is a possibility that it is substantially different **Low certainty:** Our confidence in the effect estimate is limited: The true effect may be substantially different from the estimate of the effect **Very low certainty:** We have very little confidence in the effect estimate: The true effect is likely to be substantially different from the estimate of effect | | | | | | | |

#### Explanations

1. Population included individuals who decided to participate in the trial with a high motivation to quit smoking in the next 2 weeks.
2. Along with Nicotine patches and/or nicotine-E-cig, all participants received a moderate-intensity behavioral support once a week for 6 weeks that aimed to provide 10-15 min of withdrawal-oriented behavioral support with some advice to the participant on using their allocated treatment.
3. Each participant received 14 weeks of 21 mg, 24h nicotine patches, plus an 18 mg/L nicotine e-cig, or patches plus a nicotine-free e-cig.
4. Issues of bias not substantive enough to warrant downrating
5. One study only, we do not downrate this domain.
6. No indirectness, we do not downrate this domain.
7. The optimal information size was not met and inadequate sample size (<2000 participants). Confidence interval encompasses both harm and benefit. We downrate this domain by -2.0.
8. Search not completely comprehensive but study reports negative findings. We do not downrate this domain.
9. Unable to assess confidence intervals and the optimal information size. We downrate this domain by -1.0.

## Appendix H Table 10. E-cigarette with no nicotine + other smoking cessation treatment + standard care vs. Other smoking cessation treatment + standard care: Smoking cessation, reduction, adverse events, possible adverse outcomes

E-cigarette with no nicotine + other smoking cessation treatment (behavioural support) + standard care (nicotine patch) vs. Other smoking cessation treatment (behavioural support) + standard care (nicotine patch)

**Bibliography:** Walker 2020; Date of last search September 2020

| **Certainty assessment** | | | | | | | | **Summary of findings** | | | | | | | | |
| --- | --- | --- | --- | --- | --- | --- | --- | --- | --- | --- | --- | --- | --- | --- | --- | --- |
| **№ of participants (studies) Follow-up** | **Risk of bias** | **Inconsistency** | **Indirectness** | **Imprecision** | **Publication bias** | **Overall certainty of evidence** | **Study event rates (%)** | | | | **Relative effect (95% CI)** | | **Anticipated absolute effects** | | | |
|  |  |  |  |  |  |  | **e-cigarettes with no nicotine + other treatment + standard care** | | **other treatment + standard care** | |  |  | **Risk with other treatment + standard care** | | | **Risk difference with e-cigarettes with no nicotine + other treatment + standard care** |
| **Tobacco use abstinence: Continuous smoking abstinence (follow up: 6 months)^a,b,c^**  Assessed with: self-reported, allowing ≤5 cigarettes in total, eCOE verified ≤9ppm | | | | | | | | | | | | | | | | |
| 624  (1 RCT) | not serious ^d^ | not serious ^e^ | not serious ^f^ | very serious ^g^ | none ^h^ | ⨁⨁◯◯ LOW | 20/499 (4.0%) | | 3/125  (2.4%) | | **RR 1.67** (0.50 to 5.53) | | 24 per 1,000 | | | **16 more per 1,000** (from 12 fewer to 109 more) |
| **Tobacco use abstinence: Continuous smoking abstinence (follow up: 6 months) ^a,b,c^**  Assessed with: self-reported | | | | | | | | | | | | | | | | |
| 624  (1 RCT) | not serious ^d^ | not serious ^e^ | not serious ^f^ | very serious ^g^ | none ^h^ | ⨁⨁◯◯ LOW | 53/499 (10.6%) | | 10/125  (8.0%) | | **RR 1.33** (0.70 to 2.53) | | 80 per 1,000 | | | **26 more per 1,000** (from 24 fewer to 122 more) |
| **Tobacco use abstinence: 7-day point prevalence abstinence (follow up: 6 months) ^a,b,c^**  Assessed with: self-reported | | | | | | | | | | | | | | | | |
| 624  (1 RCT) | not serious ^d^ | not serious ^e^ | not serious ^f^ | very serious ^g^ | none ^h^ | ⨁⨁◯◯ LOW | 83/499 (16.6%) | | 14/125  (11.2%) | | **RR 1.49** (0.87 to 2.53) | | 112 per 1,000 | | | **55 more per 1,000** (from 15 fewer to 171 more) |
| **Reduction in tobacco smoking frequency/quantity: Change from baseline in the mean number of cigarettes smoked per day (follow up: 6 months) ^a,b,c^**  Assessed with: self-reported | | | | | | | | | | | | | | | | |
| 624  (1 RCT) | not serious ^d^ | not serious ^e^ | not serious ^f^ | serious ^i^ | none ^h^ | ⨁⨁⨁◯  MODERATE | 499 | | 125 | | - | | The mean number of cigarettes smoked per day was 8.6 (SD 1.0) | | | **MD 0 .3 lower** (0.48 lower to 0.12 lower) |
| **Reduction in tobacco smoking frequency/quantity: ≥50% reduction in the number of cigarettes/day since baseline (follow up: 6 months) ^a,b,c^**  Assessed with: self-reported | | | | | | | | | | | | | | | | |
| 624  (1 RCT) | not serious ^d^ | not serious ^e^ | not serious ^f^ | very serious ^g^ | none ^h^ | ⨁⨁◯◯ LOW | 190/499 (38.1%) | | 32/125  (25.6%) | | **RR 1.49** (1.08 to 2.05) | | 256 per 1,000 | | | **125 more per 1,000** (from 20 more to 269 more) |
| **Adverse events: Participants with a serious adverse event (follow up: 6 months) ^a,b,c^**  Assessed with: self-reported | | | | | | | | | | | | | | | | |
| 624  (1 RCT) | not serious ^d^ | not serious ^e^ | not serious ^f^ | very serious ^g^ | none ^h^ | ⨁⨁◯◯ LOW | 22/499 (4.4%) | | | 3/125  (4.4%) | | **RR 1.84** (0.56 to 6.04) | | 24 per 1,000 | **20 more per 1,000** (from 11 fewer to 121 more) | |
| **Adverse events: Total serious adverse events (follow up: 6 months) ^a,b,c^**  Assessed with: self-reported | | | | | | | | | | | | | | | | |
| 624  (1 RCT) | not serious ^d^ | not serious ^e^ | not serious ^f^ | serious ^i^ | none ^h^ | ⨁⨁⨁◯  MODERATE | At 6 months, there was a total of 27 SAEs in the intervention group (19 hospitalizations, 6 otherwise medically important, 1 life-threatening, 1 death) and 4 in the control group (3 hospitalizations, 1 otherwise medically important). | | | | | | | | | |
| **Possible adverse outcome: Change in BMI from baseline (follow up: 6 months) ^a,b,c^**  Assessed with: self-reported | | | | | | | | | | | | | | | | |
| 624  (1 RCT) | not serious ^d^ | not serious ^e^ | not serious ^f^ | serious ^i^ | none ^h^ | ⨁⨁⨁◯  MODERATE | 499 | | | 125 | | - | | The mean change in BMI from baseline was 0.1 (SD 0.4) | **MD 0.2 lower** (0.27 lower to 0.13 lower) | |
| **Possible adverse outcome: Change in weight from baseline (follow up: 6 months) ^a,b,c^**  Assessed with: self-reported | | | | | | | | | | | | | | | | |
| 624  (1 RCT) | not serious ^d^ | not serious ^e^ | not serious ^f^ | very serious ^g^ | none ^h^ | ⨁⨁◯◯ LOW | 499 | | | 125 | | - | | The mean change in weight from baseline was -0.4 (SD 1.0) | **MD 0 lower** (0.18 lower to 0.18 higher) | |
| **Possible adverse outcomes (follow up: 6 months) ^a,b,c^**  Assessed with: self-reported | | | | | | | | | | | | | | | | |
| 624  (1 RCT) | not serious ^d^ | not serious ^e^ | not serious ^f^ | serious ^i^ | none ^h^ | ⨁⨁⨁◯  MODERATE | At 6 months, various possible adverse events were reported for the intervention and control groups:  vivid dreams 11 (3%) vs 6 (10%);  itchiness 10 (3%) vs 2 (3%);  redness, swollen at patch site 11 (3%) vs 5 (8%);  dry mouth or throat 5 (2%) vs 0 (0%);  cough 4 (1%) vs 0 (0%);  nausea 4 (1%) vs 2 (3%);  headache 7 (2%) vs 1 (2%); | | | | | | | | | |

#### Explanations

1. Population included individuals who decided to participate in the trial with a high motivation to quit smoking in the next 2 weeks.
2. Along with Nicotine patches and/or nicotine-E-cig, all participants received a moderate-intensity behavioral support once a week for 6 weeks that aimed to provide 10-15 min of withdrawal-oriented behavioral support with some advice to the participant on using their allocated treatment.
3. Each participant received 14 weeks of 21 mg, 24h nicotine patches, plus an 18 mg/L nicotine e-cig, or patches plus a nicotine-free e-cig.
4. Issues of bias not substantive enough to warrant downrating
5. One study only, we do not downrate this domain.
6. No indirectness, we do not downrate this domain.
7. The optimal information size was not met and inadequate sample size (<2000 participants). Confidence interval encompasses both harm and benefit. We downrate this domain by -2.0.
8. Search not completely comprehensive but study reports negative findings. We do not downrate this domain.
9. Unable to assess confidence intervals and the optimal information size. We downrate this domain by -1.0.

| Summary of findings: | | | | | | |  |
| --- | --- | --- | --- | --- | --- | --- | --- |
| **E-cigarette with no nicotine, behavioural support, and standard care (nicotine patch) compared to behavioural support and standard care (nicotine patch) in smokers willing to quit within the next 2 weeks** | | | | | | | |
| **Patient or population**: Smokers willing to quit within the next 2 weeks  **Setting**: Community  **Intervention**: Electronic cigarettes with no nicotine, behavioural support, and standard care (nicotine patch)  **Comparison**: Behavioural support and standard care (nicotine patch) | | | | | | | |
| Outcomes | **Anticipated absolute effects^*^** (95% CI) | | Relative effect (95% CI) | № of participants  (studies) | Certainty of the evidence (GRADE) | Comments | |
|  | **Risk with behavioural support and standard care (nicotine patch)** | **Risk with e-cigarettes with no nicotine, behavioural support, and standard care (nicotine patch)** |  |  |  |  |  |
| Tobacco use abstinence: Continuous smoking abstinence^a,b,c^  Assessed with: self-reported, allowing ≤5 cigarettes in total, eCO verified ≤9ppm  Follow up: 6 months | 24 per 1,000 | 16 more per 1,000  (from 12 fewer to 109 more) | **RR 1.67**  (0.50 to 5.53) | 624  (1 RCT) | ⨁⨁◯◯^d,e,f,g,h^  LOW | Date of last search: September 2020. | |
| Tobacco use abstinence: Continuous smoking abstinence^a,b,c^  Assessed with: self-reported  Follow up: 6 months | 80 per 1,000 | 26 more per 1,000  (from 24 fewer to 122 more) | **RR 1.33**  (0.70 to 2.53) | 624  (1 RCT) | ⨁⨁◯◯ ^d,e,f,g,h^  LOW | Date of last search: September 2020. | |
| Tobacco use abstinence: 7-day point prevalence abstinence^a,b,c^  Assessed with: self-reported  Follow up: 6 months | 112 per 1,000 | 55 more per 1,000  (from 15 fewer to 171 more) | **RR 1.49**  (0.87 to 2.53) | 624  (1 RCT) | ⨁⨁◯◯ ^d,e,f,g,h^  LOW | Date of last search: September 2020. | |
| Reduction in tobacco smoking frequency/quantity: Change from baseline in the mean number of cigarettes smoked per day^a,b,c^  Assessed with: self-reported  Follow up: 6 months | The mean number of cigarettes smoked per day was 8.6 (SD 1.0) | MD 0.3 lower  (0.48 lower to 0.12 lower) | - | 624  (1 RCT) | ⨁⨁⨁◯^d,e,f,I,h^  MODERATE | Date of last search: September 2020. | |
| Reduction in tobacco smoking frequency/quantity: ≥50% reduction in the number of cigarettes/day since baseline^a,b,c^  Assessed with: self-reported  Follow up: 6 months | 256 per 1,000 | 125 more per 1,000  (from 20 more to 269 more) | **RR 1.49**  (1.08 to 2.05) | 624  (1 RCT) | ⨁⨁◯◯ ^d,e,f,g,h^  LOW | Date of last search: September 2020. | |
| Adverse events: Participants with a serious adverse event^a,b,c^  Assessed with: self-reported  Follow up: 6 months | 24 per 1,000 | 20 more per 1,000  (from 11 fewer to 121 more) | **RR 1.84**  (0.56 to 6.04) | 624  (1 RCT) | ⨁⨁◯◯ ^d,e,f,g,h^  LOW | Date of last search: September 2020. | |
| Adverse events: Total serious adverse events^a,b,c^  Assessed with: self-reported  Follow up: 6 months | At 6 months, there was a total of 27 SAEs in the intervention group (19 hospitalizations, 6 otherwise medically important, 1 life-threatening, 1 death) and 4 in the control group (3 hospitalizations, 1 otherwise medically important). | | **-** | 624  (1 RCT) | ⨁⨁⨁◯ ^d,e,f,I,h^  MODERATE | Date of last search: September 2020. | |
| Possible adverse outcome: Change in BMI from baseline^a,b,c^  Assessed with: self-reported  Follow up: 6 months | The mean change in BMI from baseline was 0.1 (SD 0.4) | MD 0.2 lower  (0.27 lower to 0.13 lower) | **-** | 624  (1 RCT) | ⨁⨁⨁◯ ^d,e,f,I,h^  MODERATE | Date of last search: September 2020. | |
| Possible adverse outcome: Change in weight from baseline^a,b,c^  Assessed with: self-reported  Follow up: 6 months | The mean change in weight from baseline was -0.4 (SD 1.0) | MD 0 lower  (0.18 lower to 0.18 higher) | **-** | 624  (1 RCT) | ⨁⨁◯◯ ^d,e,f,g,h^  LOW | Date of last search: September 2020. | |
| Possible adverse outcomes^a,b,c^  Assessed with: self-reported  Follow up: 6 months | At 6 months, various possible adverse events were reported for the intervention and control groups:  vivid dreams 11 (3%) vs 6 (10%);  itchiness 10 (3%) vs 2 (3%);  redness, swollen at patch site 11 (3%) vs 5 (8%);  dry mouth or throat 5 (2%) vs 0 (0%);  cough 4 (1%) vs 0 (0%);  nausea 4 (1%) vs 2 (3%);  headache 7 (2%) vs 1 (2%); | | **-** | 624  (1 RCT) | ⨁⨁⨁◯ ^d,e,f,I,h^  MODERATE | Date of last search: September 2020. | |
| ***The risk in the intervention group** (and its 95% confidence interval) is based on the assumed risk in the comparison group and the **relative effect** of the intervention (and its 95% CI).  **CI:** Confidence interval; **RR:** Risk ratio | | | | | | | |
| **GRADE Working Group grades of evidence** **High certainty:** We are very confident that the true effect lies close to that of the estimate of the effect **Moderate certainty:** We are moderately confident in the effect estimate: The true effect is likely to be close to the estimate of the effect, but there is a possibility that it is substantially different **Low certainty:** Our confidence in the effect estimate is limited: The true effect may be substantially different from the estimate of the effect **Very low certainty:** We have very little confidence in the effect estimate: The true effect is likely to be substantially different from the estimate of effect | | | | | | | |

#### Explanations

1. Population included individuals who decided to participate in the trial with a high motivation to quit smoking in the next 2 weeks.
2. Along with Nicotine patches and/or nicotine-E-cig, all participants received a moderate-intensity behavioral support once a week for 6 weeks that aimed to provide 10-15 min of withdrawal-oriented behavioral support with some advice to the participant on using their allocated treatment.
3. Each participant received 14 weeks of 21 mg, 24h nicotine patches, plus an 18 mg/L nicotine e-cig, or patches plus a nicotine-free e-cig.
4. Issues of bias not substantive enough to warrant downrating
5. One study only, we do not downrate this domain.
6. No indirectness, we do not downrate this domain.
7. The optimal information size was not met and inadequate sample size (<2000 participants). Confidence interval encompasses both harm and benefit. We downrate this domain by -2.0.
8. Search not completely comprehensive but study reports negative findings. We do not downrate this domain.
9. Unable to assess confidence intervals and the optimal information size. We downrate this domain by -1.0.

## Appendix H Table 11. E-cigarette with no nicotine + other smoking cessation treatment vs. Other smoking cessation treatment: Smoking cessation, reduction, adverse events

E-cigarette with no nicotine + other smoking cessation treatment (behavioural support) vs. Other smoking cessation treatment (behavioural support)

**Bibliography:** Lucchiari 2020; Eisenberg 2020; Date of last search September 2020

| **Certainty assessment** | | | | | | | | **Summary of findings** | | | | | | | | |
| --- | --- | --- | --- | --- | --- | --- | --- | --- | --- | --- | --- | --- | --- | --- | --- | --- |
| **№ of participants (studies) Follow-up** | **Risk of bias** | **Inconsistency** | **Indirectness** | **Imprecision** | **Publication bias** | **Overall certainty of evidence** | **Study event rates (%)** | | | | **Relative effect (95% CI)** | | **Anticipated absolute effects** | | | |
|  |  |  |  |  |  |  | **e-cigarettes with no nicotine + other treatment** | | **other treatment** | |  |  | **Risk with other treatment** | | | **Risk difference with e-cigarettes with no nicotine + other treatment** |
| **Tobacco use abstinence: Continuous smoking abstinence (follow up: 6 months)^a,b,c^**  Assessed with: self-reported, eCO^C^ verified ≤7ppm | | | | | | | | | | | | | | | | |
| 140  (1 RCT) | very serious ^d^ | not serious ^e^ | not serious ^f^ | very serious ^g^ | none ^h^ | ⨁◯◯◯  VERY LOW | 11/70 (15.7%) | | 7/70  (10.0%) | | **RR 1.57** (0.65 to 3.82) | | 100 per 1,000 | | | **57 more per 1,000** (from 35 fewer to 282 more) |
| **Reduction in tobacco smoking frequency/quantity: Change in mean number of daily cigarettes smoked since baseline (follow up: 24 weeks)^i,j,k^**  Assessed with: self-reported | | | | | | | | | | | | | | | | |
| 249  (1 RCT) | very serious ^l^ | not serious ^e^ | not serious ^f^ | very serious ^m^ | none ^h^ | ⨁◯◯◯  VERY LOW | At 24 weeks, the intervention group had a -9.1 change in mean number of daily cigarettes smoked since baseline and the control group had -5.5. SD were not reported. | | | | | | | | | |
| **Reduction in tobacco smoking frequency/quantity: Number of daily cigarettes smoked (follow up: 6 months)^a,b,c^**  Assessed with: self-reported | | | | | | | | | | | | | | | | |
| 140  (1 RCT) | very serious ^d^ | not serious ^e^ | not serious ^f^ | very serious ^n^ | none ^h^ | ⨁◯◯◯  VERY LOW | 70 | | 70 | | **-** | | The mean number of daily cigarettes smoked was 13.45 (SD 6.49) | | | **MD 0.58 higher** (1.82 lower to 2.98 higher) |
| **Reduction in tobacco smoking frequency/quantity: eCO levels (follow up: 6 months)^a,b,c^**  Assessed with: measured with Bedfont Micro Smokerlyzers (Bedfont Scientific, Maidstone, UK) | | | | | | | | | | | | | | | | |
| 140  (1 RCT) | very serious ^d^ | not serious ^e^ | not serious ^f^ | serious ^o^ | none ^h^ | ⨁◯◯◯  VERY LOW | 70 | | | 70 | | - | | The mean change in eCO levels was 16.52 (SD 10.24) | **MD 1.24 lower** (2.38 lower to 4.86 higher) | |
| **Adverse events: Serious adverse events (follow up: 12 weeks)^i,j,k^**  Assessed with: self-reported | | | | | | | | | | | | | | | | |
| 249  (1 RCT) | very serious ^l^ | not serious ^e^ | not serious ^f^ | very serious ^m^ | none ^h^ | ⨁◯◯◯  VERY LOW | Serious adverse events were adjudicated by an end points evaluation committee and include death, respiratory, cardiovascular, neuropsychiatric or other events. At 12 weeks, the intervention group had experienced 4 (3.1%) and the control group had experienced 2 (1.7%). | | | | | | | | | |
| **Adverse events: Serious adverse events (follow up: 12 to 24 weeks)^i,j,k^**  Assessed with: self-reported | | | | | | | | | | | | | | | | |
| 249  (1 RCT) | very serious ^l^ | not serious ^e^ | not serious ^f^ | very serious ^m^ | none ^h^ | ⨁◯◯◯  VERY LOW | Serious adverse events were adjudicated by an end points evaluation committee and include death, respiratory, cardiovascular, neuropsychiatric or other events. At 12 to 24 weeks, the intervention group had experienced 2 (1.7%) and the control group had experienced 2 (1.7%). | | | | | | | | | |
| **Adverse events: Mild adverse events (follow up: 12 weeks)^i,j,k^**  Assessed with: self-reported | | | | | | | | | | | | | | | | |
| 249  (1 RCT) | very serious ^l^ | not serious ^e^ | not serious ^f^ | very serious ^m^ | none ^h^ | ⨁◯◯◯  VERY LOW | Mild adverse events included cough, dry mouth, headache, rhinitis, throat irritation, dyspnea, sore throat, light headedness, dizziness, mouth irritation, nausea, indigestion, mouth ulcers, or vertigo. Only the first event for each participant in each category was counted. At 12 weeks, the intervention group had experienced 118 (93%) and the control group had experienced 88 (73%). | | | | | | | | | |

#### Explanations

1. Population included individuals who decided to participate in a screening program with a high motivation to stop smoking.
2. All participants received a 3-month cessation program that included a cognitive-behavioural intervention that aimed to support participants in changing their behaviour and improving motivation to quit.
3. Each participant received an e-cigarette kit and 12 10-mL liquid cartridges (8 mg/mL nicotine concentration). During the first week, participants could use the e-cigarettes ad libitum. After the first week, they were asked to use the e-cigarette for the next 11 weeks.
4. High risk for incomplete outcome data and selective outcome reporting, unclear for blinding of outcome assessors and allocation concealment. We downrate by -2.0.
5. One study only, we do not downrate this domain.
6. No indirectness, we do not downrate this domain.
7. The optimal information size was not met (total of 18 events) and inadequate sample size (<2000 participants). Confidence interval encompasses both harm and benefit. We downrate this domain by -2.0.
8. Although few studies, literature search was comprehensive. No suspicion of suppression of results of other studies. We do not downrate this domain.
9. Study enrolled adults motivated to quit smoking.
10. All treatment groups received relapse prevention counseling (minimum 30 minutes at baseline, 10 minutes during telephone follow-ups, and 15-20 minutes at clinic visits).
11. Participants randomized to e-cigarettes were supplied with 12 weeks of e-cigarettes (15 or 0 mg nicotine/mL).
12. High risk for blinding of participants/personnel, unclear for blinding of outcome assessors. We downrate by -2.0.
13. Unable to assess confidence intervals and the optimal information size. Inadequate sample size (<400 participants for a continuous outcome). We downrate this domain by -2.0.
14. Confidence interval encompasses both harm and benefit. Inadequate sample size (<400 participants for a continuous outcome). We downrate this domain by -2.0
15. Confidence interval encompasses benefit. The optimal information size not met (total of 140 events) and inadequate sample size (<400 participants for a continuous outcome). We downrate this domain by -1.0.

| Summary of findings: | | | | | | |  |
| --- | --- | --- | --- | --- | --- | --- | --- |
| **E-cigarette with no nicotine and behavioural support compared to behavioural support in smokers willing to quit** | | | | | | | |
| **Patient or population**: Smokers willing to quit  **Setting**: Mixed (lung cancer screening program, academic research setting)  **Intervention**: Electronic cigarettes with no nicotine and behavioural support  **Comparison**: Behavioural support | | | | | | | |
| Outcomes | **Anticipated absolute effects^*^** (95% CI) | | Relative effect (95% CI) | № of participants  (studies) | Certainty of the evidence (GRADE) | Comments | |
|  | **Risk with behavioural support** | **Risk with e-cigarettes with no nicotine and behavioural support** |  |  |  |  |  |
| Tobacco use abstinence: Continuous smoking abstinence^a,b,c^  Assessed with: self-reported, eCO verified ≤7ppm  Follow up: 6 months | 100 per 1,000 | 57 more per 1,000  (from 35 fewer to 282 more) | **RR 1.57**  (0.65 to 3.82) | 140  (1 RCT) | ⨁◯◯◯  VERY LOW^d,e,f,g,h^ | Date of last search: September 2020. | |
| Reduction in tobacco smoking frequency/quantity: Change in mean number of daily cigarettes smoked since baseline^i,j,k^  Assessed with: self-reported  Follow up: 24 weeks | At 24 weeks, the intervention group had a -9.1 change in mean number of daily cigarettes smoked since baseline and the control group had -5.5. SD were not reported. | | **-** | 249  (1 RCT) | ⨁◯◯◯  VERY LOW^e,f,h,l,m^ | Date of last search: September 2020. | |
| Reduction in tobacco smoking frequency/quantity: Number of daily cigarettes smoked^a,b,c^  Assessed with: self-reported  Follow up: 6 months | The mean number of daily cigarettes smoked was 13.45 (SD 6.49) | MD 0.58 higher  (1.82 lower to 2.98 higher) | **-** | 140  (1 RCT) | ⨁◯◯◯  VERY LOW^d,e,f,h,n^ | Date of last search: September 2020. | |
| Reduction in tobacco smoking frequency/quantity: eCO levels^a,b,c^  Assessed with: measured with Bedfont Micro Smokerlyzers (Bedfont Scientific, Maidstone, UK)  Follow up: 6 months | The mean change in eCO levels was 6.52 (SD 10.24) | MD 8.76 higher  (5.17 higher to 12.35 higher) | **-** | 140  (1 RCT) | ⨁◯◯◯  VERY LOW^d,e,f,h,o^ | Date of last search: September 2020. | |
| Adverse events: Serious adverse events^i,j,k^  Assessed with: self-reported  Follow up: 12 weeks | Serious adverse events were adjudicated by an end points evaluation committee and include death, respiratory, cardiovascular, neuropsychiatric or other events. At 12 weeks, the intervention group had experienced 4 (3.1%) and the control group had experienced 2 (1.7%). | | **-** | 249  (1 RCT) | ⨁◯◯◯  VERY LOW^e,f,h,l,m^ | Date of last search: September 2020. | |
| Adverse events: Serious adverse events^i,j,k^  Assessed with: self-reported  Follow up: 12 to 24 weeks | Serious adverse events were adjudicated by an end points evaluation committee and include death, respiratory, cardiovascular, neuropsychiatric or other events. At 12 to 24 weeks, the intervention group had experienced 2 (1.7%) and the control group had experienced 2 (1.7%). | | **-** | 249  (1 RCT) | ⨁◯◯◯  VERY LOW^e,f,h,l,m^ | Date of last search: September 2020. | |
| Adverse events: Mild adverse events^i,j,k^  Assessed with: self-reported  Follow up: 12 weeks | Mild adverse events included cough, dry mouth, headache, rhinitis, throat irritation, dyspnea, sore throat, light headedness, dizziness, mouth irritation, nausea, indigestion, mouth ulcers, or vertigo. Only the first event for each participant in each category was counted. At 12 weeks, the intervention group had experienced 118 (93%) and the control group had experienced 88 (73%). | | **-** | 249  (1 RCT) | ⨁◯◯◯  VERY LOW^e,f,h,l,m^ | Date of last search: September 2020. | |
| ***The risk in the intervention group** (and its 95% confidence interval) is based on the assumed risk in the comparison group and the **relative effect** of the intervention (and its 95% CI).  **CI:** Confidence interval; **RR:** Risk ratio | | | | | | | |
| **GRADE Working Group grades of evidence** **High certainty:** We are very confident that the true effect lies close to that of the estimate of the effect **Moderate certainty:** We are moderately confident in the effect estimate: The true effect is likely to be close to the estimate of the effect, but there is a possibility that it is substantially different **Low certainty:** Our confidence in the effect estimate is limited: The true effect may be substantially different from the estimate of the effect **Very low certainty:** We have very little confidence in the effect estimate: The true effect is likely to be substantially different from the estimate of effect | | | | | | | |

#### Explanations

1. Population included individuals who decided to participate in a screening program with a high motivation to stop smoking.
2. All participants received a 3-month cessation program that included a cognitive-behavioural intervention that aimed to support participants in changing their behaviour and improving motivation to quit.
3. Each participant received an e-cigarette kit and 12 10-mL liquid cartridges (8 mg/mL nicotine concentration). During the first week, participants could use the e-cigarettes ad libitum. After the first week, they were asked to use the e-cigarette for the next 11 weeks.
4. High risk for incomplete outcome data and selective outcome reporting, unclear for blinding of outcome assessors and allocation concealment. We downrate by -2.0.
5. One study only, we do not downrate this domain.
6. No indirectness, we do not downrate this domain.
7. The optimal information size was not met (total of 18 events) and inadequate sample size (<2000 participants). Confidence interval encompasses both harm and benefit. We downrate this domain by -2.0.
8. Although few studies, literature search was comprehensive. No suspicion of suppression of results of other studies. We do not downrate this domain.
9. Study enrolled adults motivated to quit smoking.
10. All treatment groups received relapse prevention counseling (minimum 30 minutes at baseline, 10 minutes during telephone follow-ups, and 15-20 minutes at clinic visits).
11. Participants randomized to e-cigarettes were supplied with 12 weeks of e-cigarettes (15 or 0 mg nicotine/mL).
12. High risk for blinding of participants/personnel, unclear for blinding of outcome assessors. We downrate by -2.0.
13. Unable to assess confidence intervals and the optimal information size. Inadequate sample size (<400 participants for a continuous outcome). We downrate this domain by -2.0.
14. Confidence interval encompasses both harm and benefit. Inadequate sample size (<400 participants for a continuous outcome). We downrate this domain by -2.0
15. Confidence interval encompasses benefit. The optimal information size not met (total of 140 events) and inadequate sample size (<400 participants for a continuous outcome). We downrate this domain by -1.0.

## Appendix H Table 12. E-cigarette with nicotine vs. Other smoking cessation treatment (NRT choices included nicotine patch, chewing gum, nasal spray, microtab, inhalator and mouth spray): Smoking cessation, reduction, adverse events

E-cigarette with nicotine versus other smoking cessation treatment (NRT choices included nicotine patch, chewing gum, nasal spray, microtab, inhalator and mouth spray)

**Bibliography:** Myers Smith 2022; Date of last search January 2024

| **Certainty assessment** | | | | | | | | **Summary of findings** | | | | | | | | | |
| --- | --- | --- | --- | --- | --- | --- | --- | --- | --- | --- | --- | --- | --- | --- | --- | --- | --- |
| **№ of participants (studies) Follow-up** | **Risk of bias** | **Inconsistency** | **Indirectness** | **Imprecision** | **Publication bias** | **Overall certainty of evidence** | **Study event rates (%)** | | | | **Relative effect (95% CI)** | | | **Anticipated absolute effects** | | | |
|  |  |  |  |  |  |  | **e-cigarettes with nicotine** | | **other treatment** | |  |  |  | **Risk with other treatment** | | | **Risk difference with e-cigarettes with nicotine** |
| **Tobacco use abstinence: Sustained abstinence (follow up: 6 months)^a,b,c^**  Assessed with: self-reported, no more than five cigarettes smoked since the contact at 4 weeks, eCO^C^ verified < 8ppm | | | | | | | | | | | | | | | | | |
| 135  (1 RCT) | not serious^d^ | not serious^e^ | serious^f^ | Serious to very serious^g^ | none^h^ | ⨁⨁◯◯ LOW | 13/68 (19.1%) | | 2/67  (3%) | | **RR 6.4**  (1.5 to 27.3) | | | 30 per 1000 | | | 161 more per 1000  (15 more to 785 more) |
| **Tobacco use abstinence: Self-reported abstinence (follow up: 6 months)^a,b,c^**  Assessed with: self-reported, eCO verified < 8ppm | | | | | | | | | | | | | | | | | |
| 135  (1 RCT) | not serious^d^ | not serious^e^ | serious^f^ | Serious to very serious^g^ | none^h^ | ⨁⨁◯◯ LOW | 20/68  (29.4%) | | 6/67  (9%) | | **RR 3.3**  (1.4 to 7.7) | | 90 more per 1000 | | | | 206 more per 1000  (36 more to 600 more) |
| **Reduction in tobacco smoking frequency/quantity: Reduction in the number of daily cigarettes smoked by at least 50% (follow up: 6 months)^a,b,c^**  Assessed with: self-reported, reduction of at least 50% in the number of cigarettes smoked per day | | | | | | | | | | | | | | | | | |
| 135  (1 RCT) | not serious^d^ | not serious^e^ | serious^f^ | Serious to very serious^g^ | none^h^ | ⨁⨁◯◯ LOW | 45/68  (66.2%) | | 25/67  (37.3%) | | **RR 1.8**  **(1.3 to 2.5)** | | | 373 per 1000 | | | 299 more per 1000  (112 more to 560 more) |
| **Reduction in tobacco smoking frequency/quantity: eCO levels (follow up: 6 months)^a,b,c^**  Assessed with: Reduction in end-expired CO levels of ≥ 50% compared to baseline | | | | | | | | | | | | | | | | | |
| 135  (1 RCT) | not serious^d^ | not serious^e^ | serious^f^ | Serious to very serious^g^ | none^h^ | ⨁⨁◯◯ LOW | 18/68  (26.5%) | | | 4/67  (6%) | | **RR 4.4**  (1.6 to 12.4) | | | 60 per 1000 | 203 more per 1000  (36 more to 681 more) | |
| **Adverse events: Mild adverse events (follow up: 6 months)^a,b,c^**  Assessed with: self-reported | | | | | | | | | | | | | | | | | |
| 135  (1 RCT) | not serious^d^ | not serious^e^ | serious^f^ | Very serious^i^ | none^h^ | ⨁◯◯◯  VERY LOW | At week 24, in the EC arm there was a report of dry mouth (n = 1) and cough/throat/chest irritation (n = 3) while in the NRT arm there was a report of itchiness (n = 1) and nausea (n = 1). | | | | | | | | | | |

**CI:** Confidence interval; **RR**: Risk Ratio

#### Explanations

1. Study participants had a history of unsuccessful quitting with stop smoking medications.
2. No co-interventions reported.
3. Participants in the EC arm were shown three different refillable EC products (Innokin T18E, Smok and TECC mini and were instructed to use one of these or any other product of their choice. At 6 months, 80.7% used 1-10% nicotine, 16.1% used >10% nicotine, and 3.2% used 0% nicotine
4. Issues of bias not substantive enough to warrant downrating.
5. One study only, we do not downrate this domain.
6. Use of allocated products was similar in the two study arms at week 1 and at 4 weeks. Use diverged substantially by 6 months. Among participants who reported EC strength at both baseline and at 6 months, the nicotine content of e-liquids was significantly reduced. We downrate by -1.0.
7. Confidence interval encompasses small but important benefit to large benefit. The optimal information size not met and inadequate sample size (<2000). We downrate by -1.5.
8. Although there were few studies, the literature search was comprehensive. No suspicion of suppression of results of other studies. We do not downrate this domain.
9. Unable to assess confidence intervals and the optimal information size. Inadequate sample size (<400 participants for a continuous outcome). We downrate this domain by -2.0.

| Summary of findings: | | | | | | |
| --- | --- | --- | --- | --- | --- | --- |
| **E-cigarettes with nicotine compared to other interventions (NRT choices included nicotine patch, chewing gum, nasal spray, microtab, inhalator and mouth spray) in the general/mixed population of smokers** | | | | | | |
| **Patient or population**: General/mixed population of smokers  **Setting**: Academic research setting (Queen Mary University of London)  **Intervention**: Electronic cigarettes with nicotine  **Comparison**: Other interventions | | | | | | |
| Outcomes | **Anticipated absolute effects^*^** (95% CI) | | Relative effect (95% CI) | № of participants  (studies) | Certainty of the evidence (GRADE) | Comments |
|  | **Risk with other intervention** | **Risk with e-cigarettes with nicotine** |  |  |  |  |
| Tobacco use abstinence: Sustained abstinence ^a,b,c^  Follow up: 6 months | 29.9 per 1000 | 161.2 more per 1000  (14.9 more to 785.1 more) | RR 6.4  (1.5 to 27.3) | 135  (1 RCT) | ⨁⨁◯◯ LOW^d,e,f,g,h^ | Date of last search: January 2024 |
| Tobacco use abstinence: eCO verified <8ppm ^a,b,c^  Follow up: 6 months | 89.6 more per 1000 | 206 more per 1000  (35.8 more to 600 more) | RR 3.3  (1.4 to 7.7) | 135  (1 RCT) | ⨁⨁◯◯ LOW^d,e,f,g,h^ | Date of last search: January 2024 |
| Reduction in smoking frequency/quantity – at least by 50% per day ^a,b,c^  Follow up: 6 months | 373.1 per 1000 | 298.5 more per 1000  (111.9 more to 559.7 more) | RR 1.8  (1.3 to 2.5) | 135  (1 RCT) | ⨁⨁◯◯ LOW^d,e,f,g,h^ | Date of last search: January 2024 |
| Reduction in smoking frequency/quantity – eCO levels ^a,b,c^  Follow up: 6 months | 59.7 per 1000 | 203 more per 1000  (35.8 more to 680.6 more) | RR 4.4  (1.6 to 12.4) | 135  (1 RCT) | ⨁⨁◯◯ LOW^d,e,f,g,h^ | Date of last search: January 2024 |
| Adverse events (self-reported) ^a,b,c^  Follow up: 6 months | At week 24, in the EC arm there was a report of dry mouth (n = 1) and cough/throat/chest irritation (n = 3) while in the NRT arm there was a report of itchiness (n = 1) and nausea (n = 1). | | - | 135  (1 RCT) | ⨁◯◯◯  VERY LOW^d,e,f,i,h^ | Date of last search: January 2024 |
| ***The risk in the intervention group** (and its 95% confidence interval) is based on the assumed risk in the comparison group and the **relative effect** of the intervention (and its 95% CI).  **CI:** Confidence interval; **RR:** Risk Ratio | | | | | | |
| **GRADE Working Group grades of evidence** **High certainty:** We are very confident that the true effect lies close to that of the estimate of the effect **Moderate certainty:** We are moderately confident in the effect estimate: The true effect is likely to be close to the estimate of the effect, but there is a possibility that it is substantially different **Low certainty:** Our confidence in the effect estimate is limited: The true effect may be substantially different from the estimate of the effect **Very low certainty:** We have very little confidence in the effect estimate: The true effect is likely to be substantially different from the estimate of effect | | | | | | |

#### Explanations

1. Study participants had a history of unsuccessful quitting with stop smoking medications.
2. No co-interventions reported.
3. Participants in the EC arm were shown three different refillable EC products (Innokin T18E, Smok and TECC mini and were instructed to use one of these or any other product of their choice. At 6 months, 80.7% used 1-10% nicotine, 16.1% used >10% nicotine, and 3.2% used 0% nicotine
4. Issues of bias not substantive enough to warrant downrating.
5. One study only, we do not downrate this domain.
6. Use of allocated products was similar in the two study arms at week 1 and at 4 weeks. Use diverged substantially by 6 months. Among participants who reported EC strength at both baseline and at 6 months, the nicotine content of e-liquids was significantly reduced. We downrate by -1.0.
7. Confidence interval encompasses small but important benefit to large benefit. The optimal information size not met and inadequate sample size (<2000). We downrate by -1.5.
8. Although there were few studies, the literature search was comprehensive. No suspicion of suppression of results of other studies. We do not downrate this domain.

Unable to assess confidence intervals and the optimal information size. Inadequate sample size (<400 participants for a continuous outcome). We downrate this domain by -2.0.

1. Unable to assess confidence intervals and the optimal information size. Inadequate sample size (<400 participants for a continuous outcome). We downrate this domain by -2.0.

## Appendix H Table 13. E-cigarette with nicotine vs. Other smoking cessation treatment (Quit advice): Smoking cessation, reduction

E-cigarette with nicotine versus other smoking cessation treatment (Quit advice; printed smoking-cessation materials)

**Bibliography:** Xu 2023; Date of last search January 2024

| **Certainty assessment** | | | | | | | | **Summary of findings** | | | | | | | | | | |
| --- | --- | --- | --- | --- | --- | --- | --- | --- | --- | --- | --- | --- | --- | --- | --- | --- | --- | --- |
| **№ of participants (studies) Follow-up** | **Risk of bias** | **Inconsistency** | **Indirectness** | **Imprecision** | **Publication bias** | **Overall certainty of evidence** | **Study event rates (%)** | | | | **Relative effect (95% CI)** | | | **Anticipated absolute effects** | | | | |
|  |  |  |  |  |  |  | **e-cigarettes with nicotine** | | **Quit advice** | |  |  |  | **Risk with Quit advice** | | | | **Risk difference with e-cigarettes with nicotine** |
| **Tobacco use abstinence: past 30-day smoking (follow up: 6 months)^a,b,c^**  Assessed with: self-reported, Past 30-day switching, defined as a "no" response to the question, "In the past 30 days, have you smoked a cigarette, even one or two puffs?" | | | | | | | | | | | | | | | | | | |
| 837  (1 RCT) | Very serious^d^ | Not serious^e^ | Not serious^f^ | Serious^g^ | none^h^ | ⨁◯◯◯  VERY LOW | 98/566  (17.3%) | | 10/271  (3.7%) | | **RR 4.69**  (2.48 to 8.84) | | | 37 per 1000 | | | | 136 more per 1000  (55 more to 289 more) |
| **Tobacco use abstinence: past 30-day smoking (follow up: 12 months)^a,b,c^**  Assessed with: self-reported, Past 30-day switching, defined as a "no" response to the question, "In the past 30 days, have you smoked a cigarette, even one or two puffs?" | | | | | | | | | | | | | | | | | | |
| 837  (1 RCT) | Very serious^d^ | Not serious^e^ | Not serious^f^ | Serious^i^ | none^h^ | ⨁◯◯◯  VERY LOW | 92/566  (6.1%) | | | 14/271  (5.2%) | **RR 3.14**  (1.82 to 5.41) | | 52 per 1000 | | | 111 more per 1000  (42 more to 228 more) | | |
| **Reduction in tobacco smoking frequency/quantity: Reduction in the number of daily cigarettes smoked (follow up: 6 months)^a,b,c^**  Assessed with: self-reported, number of cigarettes smoked per day | | | | | | | | | | | | | | | | | | |
| 837  (1 RCT) | Very serious^d^ | Not serious^e^ | Not serious^f^ | Very serious^k^ | none^h^ | ⨁◯◯◯  VERY LOW | 63/566  (11.16%) | | 40/271  (14.62%) | | **RR 0.73**  (0.68 to 0.77) | | | 148 per 1000 | | | | 40 fewer per 1000  (from 47 fewer to 34 fewer) |
| **Reduction in tobacco smoking frequency/quantity: Reduction in the number of daily cigarettes smoked (follow up: 12 months)^a,b,c^**  Assessed with: self-reported, number of cigarettes smoked per day | | | | | | | | | | | | | | | | | | |
| 837  (1 RCT) | Very serious^d^ | Not serious^e^ | Not serious^f^ | Very serious^k^ | none^h^ | ⨁◯◯◯  VERY LOW | 199/566  (35.2%) | | | 45/271  (16.4%) | | **RR 0.80**  (0.68 to 0.94) | | | 166.1 per 1000 | | 33 fewer per 1000  (from 53 fewer to 10 fewer) | |

**CI:** Confidence interval; **RR**: Risk Ratio

#### Explanations

1. Study participants recruited were not required to be interested in quitting or ready to quit smoking.
2. No co-interventions reported.
3. Participants in the JUUL group received two devices, two to five packs of pods [four pods per pack, 5% nicotine by weight (59 mg/ml)], based on baseline cigarette consumption
4. 3 of 4 authors have affiliations with JUUL labs which could be added as bias due to Conflicts Of Interest (COI). They were all involved in writing and analysis of the paper. We downrate this domain by -2.0.
5. One study only, we do not downrate this domain.
6. No indirectness, we do not downrate this domain.
7. Confidence interval encompasses small but important benefit to large benefit. The optimal information size was met (a total of 107 events) but inadequate sample size (<2000). We downrate this domain by -1.0.
8. Literature search was comprehensive. No suspicion of suppression of results. We do not downrate this domain.
9. Confidence interval encompasses small but important benefit to large benefit. The optimal information size was met (a total of 106 events) but inadequate sample size (<2000). We downrate this domain by -1.0.
10. Confidence interval encompasses two ranges of effect (moderate harm to small but important harm). The optimal information size was met (a total of 103 events) but inadequate sample size (<2000 participants). We downrate by
11. Confidence interval encompasses harm (moderate harm to small but important harm), Inadequate sample size and optimal information size was not met. We downrate the domain by -2.0.

| Summary of findings: | | | | | | |
| --- | --- | --- | --- | --- | --- | --- |
| **E-cigarettes with nicotine compared to other interventions (Quit advice) in the general/mixed population of smokers** | | | | | | |
| **Patient or population**: General/mixed population of smokers  **Setting**: Research center (Center for Substance Use Research)  **Intervention**: Electronic cigarettes with nicotine  **Comparison**: Other interventions | | | | | | |
| Outcomes | **Anticipated absolute effects^*^** (95% CI) | | Relative effect (95% CI) | № of participants  (studies) | Certainty of the evidence (GRADE) | Comments |
|  | **Risk with Quit advice** | **Risk with e-cigarettes with nicotine** |  |  |  |  |
| Tobacco use abstinence: Past 30-day smoking^a,b,c^  Follow up: 6 months | 36.9 per 1000 | 136.2 more per 1000  (54.6 more to 289.3 more) | **RR 4.69**  (2.48 to 8.84) | 837  (1 RCT) | ⨁◯◯◯  VERY LOW^d,e,f,g,h^ |  |
| Tobacco use abstinence: Past 30-day smoking^a,b,c^  Follow up: 12 months | 51.7 per 1000 | 110.6 more per 1000  (42.4 more to 227.8 more) | **RR 3.14**  (1.82 to 5.41) | 837  (1 RCT) | ⨁◯◯◯  VERY LOW^d,e,f,I,h^ |  |
| Reduction in tobacco smoking/frequency: cigarettes smoked per day^a,b,c^  Follow up: 6 months | 147.6 per 1000 | **39.9 per 1000**  (from 47.2 lower to 33.9 lower) | **RR 0.73**  (0.68 to 0.77) | 837  (1 RCT) | ⨁◯◯◯  VERY LOW^d,e,f,k,h^ |  |
| Reduction in tobacco smoking/frequency: cigarettes smoked per day^a,b,c^  Follow up: 12 months | 166.1 per 1000 | **33.2 per 1000**  (from 53.1 lower to 10 lower) | **RR 0.80**  (0.68 to 0.94) | 837  (1 RCT) | ⨁◯◯◯  VERY LOW^d,e,f,k,h^ |  |
| ***The risk in the intervention group** (and its 95% confidence interval) is based on the assumed risk in the comparison group and the **relative effect** of the intervention (and its 95% CI).  **CI:** Confidence interval; **RR**: Risk Ratio | | | | | | |
| **GRADE Working Group grades of evidence** **High certainty:** We are very confident that the true effect lies close to that of the estimate of the effect **Moderate certainty:** We are moderately confident in the effect estimate: The true effect is likely to be close to the estimate of the effect, but there is a possibility that it is substantially different **Low certainty:** Our confidence in the effect estimate is limited: The true effect may be substantially different from the estimate of the effect **Very low certainty:** We have very little confidence in the effect estimate: The true effect is likely to be substantially different from the estimate of effect | | | | | | |

#### Explanations

1. Study participants recruited were not required to be interested in quitting or ready to quit smoking.
2. No co-interventions reported.
3. Participants in the JUUL group received two devices, two to five packs of pods [four pods per pack, 5% nicotine by weight (59 mg/ml)], based on baseline cigarette consumption
4. 3 of 4 authors have affiliations with JUUL labs which could be added as bias due to Conflicts Of Interest (COI). They were all involved in writing and analysis of the paper. We downrate this domain by -2.0.
5. One study only, we do not downrate this domain.
6. No indirectness, we do not downrate this domain.
7. Confidence interval encompasses small but important benefit to large benefit. The optimal information size was met (a total of 107 events) but inadequate sample size (<2000). We downrate this domain by -1.0.
8. Literature search was comprehensive. No suspicion of suppression of results. We do not downrate this domain.
9. Confidence interval encompasses small but important benefit to large benefit. The optimal information size was met (a total of 106 events) but inadequate sample size (<2000). We downrate this domain by -1.0.
10. Confidence interval encompasses two ranges of effect (moderate harm to small but important harm). The optimal information size was met (a total of 103 events) but inadequate sample size (<2000 participants). We downrate by
11. Confidence interval encompasses harm (moderate harm to small but important harm), Inadequate sample size and optimal information size was not met. We downrate the domain by -2.0.

## Appendix H Table 14. E-cigarette with nicotine vs. No E-cigarette: Smoking cessation, reduction

E-cigarette with nicotine versus No E-cigarette

**Bibliography:** Carpenter 2023; Date of last search January 2024

| **Certainty assessment** | | | | | | | **Summary of findings** | | | | | | | | | | | | |  |
| --- | --- | --- | --- | --- | --- | --- | --- | --- | --- | --- | --- | --- | --- | --- | --- | --- | --- | --- | --- | --- |
| **№ of participants (studies) Follow-up** | **Risk of bias** | **Inconsistency** | **Indirectness** | **Imprecision** | **Publication bias** | **Overall certainty of evidence** | **Study event rates (%)** | | | | **Relative effect (95% CI)** | | | | **Anticipated absolute effects** | | | | |  |
|  |  |  |  |  |  |  | **e-cigarettes with nicotine** | **No e-cigarettes** | | |  |  |  |  | **Risk with No e-cigarettes** | | | **Risk difference with e-cigarettes with nicotine** | |  |
| **Tobacco use abstinence: Point-prevalence abstinence (follow up: 6 months)^a,b,c^**  Assessed with: Abstinence from cigarettes - General | | | | | | | | | | | | | | | | | | | |  |
| 638  (1 RCT) | Very serious^d^ | Not serious^e^ | Serious^f^ | Serious^g^ | none^h^ | ⨁◯◯◯  VERY LOW | 58/427  (14%) | 17/211  (8%) | | | **RR 1.68**  (1 to 2.82) | | | | 81 per 1000 | | | 55 more per 1000  (0 to 147 more) | |  |
| **Tobacco use abstinence: Point-prevalence abstinence (follow up: 6 months)^a,b,c^**  Assessed with: Abstinence from cigarettes – High Motivation to quit group | | | | | | | | | | | | | | | | | | | |  |
| 174  (1 RCT) | Very serious^d^ | Not serious^e^ | Serious^f^ | Very serious^i^ | none^h^ | ⨁◯◯◯  VERY LOW | 24/116  (21%) | 10/58  (17%) | | | **RR 1.2**  (0.61 to 2.33) | | | 172 per 1000 | | | 34 more per 1000  (67 fewer to 229 more) | | |  |
| **Tobacco use abstinence: Point-prevalence abstinence (follow up: 6 months)^a,b,c^**  Assessed with: Abstinence from cigarettes – Low Motivation to quit group | | | | | | | | | | | | | | | | | | | |  |
| 464  (1 RCT) | Very serious^d^ | Not serious^e^ | Serious^f^ | Serious to very serious^j^ | none^h^ | ⨁◯◯◯  VERY LOW | 34/311  (11%) | 7/153  (5%) | | | **RR 2.38**  (1.08 to 5.26) | | | | 46 per 1000 | | | 63 more per 1000  (4 more to 195 more) | |  |
| **Tobacco use abstinence: Floating abstinence (follow up: 6 months)^a,b,c^**  Assessed with: having ever achieved 7-days of non-smoking throughout follow-up (General) | | | | | | | | | | | | | | | | | | | |  |
| 638  (1 RCT) | Very serious^d^ | Not serious^e^ | Serious^f^ | Serious^k^ | none^h^ | ⨁◯◯◯  VERY LOW | 72/427  (17%) | 26/211  (12%) | | | **RR 1.36**  (0.9 to 2.07) | | | | 123 per 1000 | | | 44 more per 1000  (12 fewer to 132 more) | |  |
| **Tobacco use abstinence: Floating abstinence (follow up: 6 months)^a,b,c^**  Assessed with: having ever achieved 7-days of non-smoking throughout follow-up (High motivation to quit group) | | | | | | | | | | | | | | | | | | | |  |
| 174  (1 RCT) | Very serious^d^ | Not serious^e^ | Serious^f^ | Very serious^l^ | none^h^ | ⨁◯◯◯  VERY LOW | 34/116  (29%) | 10/58  (17%) | | | **RR 1.7**  (0.9 to 3.19) | | | | 172 per 1000 | | | 121 more per 1000  (17 fewer to 378 more) | |  |
| **Tobacco use abstinence: Floating abstinence (follow up: 6 months)^a,b,c^**  Assessed with: having ever achieved 7-days of non-smoking throughout follow-up (Low motivation to quit group) | | | | | | | | | | | | | | | | | | | |  |
| 464  (1 RCT) | Very serious^d^ | Not serious^e^ | Serious^f^ | Very serious^m^ | none^h^ | ⨁◯◯◯  VERY LOW | 38/311  (12%) | 16/153  (11%) | | | **RR 1.16**  (0.67 to 2.02) | | | | 105 per 1000 | | | 17 more per 1000  (35 fewer to 107 more) | |  |
| **Reduction in tobacco smoking frequency/quantity: Reduction in the number of daily cigarettes smoked by at least 50% (follow up: 6 months)^a,b,c^**  Assessed with: self-reported, ≥50% reduction in cigarettes per day (CPD) - General | | | | | | | | | | | | | | | | | | | |  |
| 638  (1 RCT) | Very serious^d^ | Not serious^e^ | Serious^f^ | Serious^n^ | none^h^ | ⨁◯◯◯  VERY LOW | 119/427  (28%) | 38/211  (18%) | | | **RR 1.54**  (1.11 to 2.14) | | | | 180 per 1000 | | | 97 more per 1000  (20 more to 205 more) | |  |
| **Reduction in tobacco smoking frequency/quantity: Reduction in the number of daily cigarettes smoked by at least 50% (follow up: 6 months)^a,b,c^**  Assessed with: self-reported, ≥50% reduction in cigarettes per day (CPD) – High motivation to quit group | | | | | | | | | | | | | | | | | | | |  |
| 174  (1 RCT) | Very serious^d^ | Not serious^e^ | Serious^f^ | Very serious^o^ | none^h^ | ⨁◯◯◯  VERY LOW | 44/116  (38%) | | 15/58  (26%) | | | **RR 1.46**  (0.89 to 2.4) | | | | 259 per 1000 | | 119 more per 1000  (28 fewer to 362 more) | |  |
| **Reduction in tobacco smoking frequency/quantity: Reduction in the number of daily cigarettes smoked by at least 50% (follow up: 6 months)^a,b,c^**  Assessed with: self-reported, ≥50% reduction in cigarettes per day (CPD) - Low motivation to quit group | | | | | | | | | | | | | | | | | | | |  |
| 464  (1 RCT) | Very serious^d^ | Not serious^e^ | Serious^f^ | Serious^p^ | none^h^ | ⨁◯◯◯  VERY LOW | 75/311  (24%) | | 23/153  (15%) | | | **RR 1.6**  (1.04 to 2.45) | | | | 150 per 1000 | | 90 more per 1000  (6 more to 218 more) | |  |
| **Adverse events: Mild adverse events (follow up: 6 months)^a,b,c^**  Assessed with: self-reported | | | | | | | | | | | | | | | | | | | |  |
| 638  (1 RCT) | Very serious^d^ | Not serious^e^ | Serious^f^ | Serious to very serious^q^ | none^h^ | ⨁◯◯◯  VERY LOW | 180/427  (42%) | | | 86/211  (41%) | | | **RR 1.03**  (0.84 to 1.25) | | 408 per 1000 | | | | 12 more per 1000  (65 fewer to 102 more) | |

#### Explanations

1. Study participants recruited were not required to be interested in quitting or ready to quit smoking.
2. No co-interventions reported.
3. Participants in the JUUL group received two devices, two to five packs of pods [four pods per pack, 5% nicotine by weight (59 mg/ml)], based on baseline cigarette consumption
4. Since no masking of participants was involved during randomization, we downrate the domain by -2.0.
5. One study only, we do not downrate this domain.
6. Participants in the e-cigs group were provided with the NJoy device, which was a closed tank system, sufficiently powered (1000 mAh) with 3 ml pre-filled nicotine (15 mg/ml). The product was provided in two ∼2-week shipments but with the general aim that participants had to self-decide if and how to use them. They were also advised if they want to reduce or quit smoking entirely. We downrate the domain by -1.0.
7. Confidence interval encompasses little to no difference to large benefit. The optimal information size was not met and the sample size was inadequate. We downrate this domain by -1.0.
8. Literature search was comprehensive. No suspicion of suppression of results. We do not downrate this domain.
9. Confidence interval encompasses both harm (moderate harm) and benefit (moderate benefit). The optimal information size was not met and inadequate sample size (<2000). We downrate this domain by -2.0.
10. Confidence interval encompasses two ranges of effect (small but important benefit to large benefit). The optimal information size was not met and inadequate sample size (<2000 participants). We downrate this domain by -1.5.
11. Confidence interval encompasses two ranges of effect (small but important benefit to large benefit). The optimal information size was met (a total of 98 events) but inadequate sample size (<2000 participants). We downrate this domain by -1.0.
12. Confidence interval encompasses two ranges of effect (small but important harm to large benefit). The optimal information size was not met (a total of 44 events) but inadequate sample size (<2000 participants). We downrate this domain by -2.0.
13. Confidence interval encompasses two ranges of effect (Moderate harm to moderate benefit). The optimal information size was not met (a total of 54 events) but inadequate sample size (<2000 participants). We downrate this domain by -2.0.
14. Confidence interval encompasses two ranges of effect (small but important benefit to large benefit). The optimal information size was met (a total of 157 events) but inadequate sample size (<2000 participants). We downrate this domain by -1.0.
15. Confidence interval encompasses two ranges of effect (small but important harm to large benefit). The optimal information size was not met (a total of 59 events) and inadequate sample size (<2000 participants). We downrate this domain by -2.0.
16. Confidence interval encompasses two ranges of effect (small but important benefit to large benefit). The optimal information size was met (a total of 98 events) but inadequate sample size (<2000 participants). We downrate this domain by -1.0.
17. Confidence interval encompasses two ranges of effect (moderate harm to moderate benefit). The optimal information size was met but the sample size was inadequate. We downrate by -1.5.

| Summary of findings: | | | | | | |
| --- | --- | --- | --- | --- | --- | --- |
| **E-cigarettes with nicotine compared to other interventions (Quit advice) in the general/mixed population of smokers** | | | | | | |
| **Patient or population**: General/mixed population of smokers  **Setting**: Research center (Center for Substance Use Research)  **Intervention**: Electronic cigarettes with nicotine  **Comparison**: Other interventions | | | | | | |
| Outcomes | **Anticipated absolute effects^*^** (95% CI) | | Relative effect (95% CI) | № of participants  (studies) | Certainty of the evidence (GRADE) | Comments |
|  | **Risk with no intervention** | **Risk with e-cigarettes with nicotine** |  |  |  |  |
| Tobacco use abstinence: Point prevalence (General population) ^a,b,c^  Follow up: 6 months | 80.6 per 1000 | 54.8 more per 1000  (0 to 146.6 more) | **RR 1.68**  (1 to 2.82) | 638  (1 RCT) | ⨁◯◯◯  VERY LOW^d,e,f,g,h^ |  |
| Tobacco use abstinence: Point prevalence (High Motivation to quit group) ^a,b,c^  Follow up: 6 months | 172.4 per 1000 | 34.5 more per 1000  (67.2 fewer to 229.3 more) | **RR 1.2**  (0.61 to 2.33) | 174  (1 RCT) | ⨁◯◯◯  VERY LOW^d,e,f,I,h^ |  |
| Tobacco use abstinence: Point prevalence (Low Motivation to quit group) ^a,b,c^  Follow up: 6 months | 45.8 per 1000 | 63.1 more per 1000  (3.7 more to 194.9 more) | **RR 2.38**  (1.08 to 5.26) | 464  (1 RCT) | ⨁◯◯◯  VERY LOW^d,e,f,j,h^ |  |
| Tobacco use abstinence: Floating abstinence (General population) ^a,b,c^  Follow up: 6 months | 123.2 per 1000 | 44.4 more per 1000  (12.3 fewer to 131.8 more) | **RR 1.36**  (0.9 to 2.07) | 638  (1 RCT) | ⨁◯◯◯  VERY LOW^d,e,f,k,h^ |  |
| Tobacco use abstinence: Floating abstinence (High Motivation to quit group) ^a,b,c^  Follow up: 6 months | 172.4 per 1000 | 120.7 more per 1000  (17.2 fewer to 377.6 more) | **RR 1.7**  (0.9 to 3.19) | 174  (1 RCT) | ⨁◯◯◯  VERY LOW^d,e,f,l,h^ |  |
| Tobacco use abstinence: Floating abstinence (Low Motivation to quit group) ^a,b,c^  Follow up: 6 months | 104.6 per 1000 | 16.7 more per 1000  (34.5 fewer to 106.7 more) | **RR 1.16**  (0.67 to 2.02) | 464  (1 RCT) | ⨁◯◯◯  VERY LOW^d,e,f,m,h^ |  |
| Reduction in tobacco smoking frequency/quantity: reduction in the number of daily cigarettes smoked (General population) ^a,b,c^  Follow up: 6 months | 180.1 per 1000 | 97.3 more per 1000  (19.8 more to 205.3 more) | **RR 1.54**  (1.11 to 2.14) | 638  (1 RCT) | ⨁◯◯◯  VERY LOW^d,e,f,n,h^ |  |
| Reduction in tobacco smoking frequency/quantity: reduction in the number of daily cigarettes smoked (High Motivation to quit group) ^a,b,c^  Follow up: 6 months | 258.6 per 1000 | 119 more per 1000  (28.4 fewer to 362.1more) | **RR 1.46**  (0.89 to 2.4) | 174  (1 RCT) | ⨁◯◯◯  VERY LOW^d,e,f,o,h^ |  |
| Reduction in tobacco smoking frequency/quantity: reduction in the number of daily cigarettes smoked (Low Motivation to quit group) ^a,b,c^  Follow up: 6 months | 150.3 per 1000 | 90.2 more per 1000  (6 more to 218 more) | **RR 1.6**  (1.04 to 2.45) | 464  (1 RCT) | ⨁◯◯◯  VERY LOW^d,e,f,p,h^ |  |
| Adverse events ^a,b,c^  Follow up: 6 months | Within e-cigarette with nicotine group, 180 people (42%) reported a total of 360 adverse events (AEs), of which 7(2%) were severe, 113 (31%) were moderate and 232 (64%) were mild. Most common AEs: Headaches (12%) and increased phlegm (12%). One serious event observed: asthma-induced hospitalization; possibly attributed to increased nebulizer use and/or e-cigs  Within control group, 86 people (41%) reported a total of 197 AEs, of which 7 (4%) were severe, 60 (30%) were moderate, and 124 (63%) were mild/ Most common reported AEs: cough (20%), increased phlegm (18%) and headaches (8.1%). | | - | 638  (1 RCT) | ⨁◯◯◯  VERY LOW^d,e,f,q,h^ |  |
| ***The risk in the intervention group** (and its 95% confidence interval) is based on the assumed risk in the comparison group and the **relative effect** of the intervention (and its 95% CI).  **CI:** Confidence interval; **RR**: Risk Ratio | | | | | | |
| **GRADE Working Group grades of evidence** **High certainty:** We are very confident that the true effect lies close to that of the estimate of the effect **Moderate certainty:** We are moderately confident in the effect estimate: The true effect is likely to be close to the estimate of the effect, but there is a possibility that it is substantially different **Low certainty:** Our confidence in the effect estimate is limited: The true effect may be substantially different from the estimate of the effect **Very low certainty:** We have very little confidence in the effect estimate: The true effect is likely to be substantially different from the estimate of effect | | | | | | |

#### Explanations

1. Study participants recruited were not required to be interested in quitting or ready to quit smoking.
2. No co-interventions reported.
3. Participants in the JUUL group received two devices, two to five packs of pods [four pods per pack, 5% nicotine by weight (59 mg/ml)], based on baseline cigarette consumption
4. Since no masking of participants was involved during randomization, we downrate the domain by -2.0.
5. One study only, we do not downrate this domain.
6. Participants in the e-cigs group were provided with the NJoy device, which was a closed tank system, sufficiently powered (1000 mAh) with 3 ml pre-filled nicotine (15 mg/ml). The product was provided in two ∼2-week shipments but with the general aim that participants had to self-decide if and how to use them. They were also advised if they want to reduce or quit smoking entirely. We downrate the domain by -1.0.
7. Confidence interval encompasses little to no difference to large benefit. The optimal information size was not met, and the sample size was inadequate. We downrate this domain by -1.0.
8. Literature search was comprehensive. No suspicion of suppression of results. We do not downrate this domain.
9. Confidence interval encompasses both harm (moderate harm) and benefit (moderate benefit). The optimal information size was not met and inadequate sample size (<2000). We downrate this domain by -2.0.
10. Confidence interval encompasses two ranges of effect (small but important benefit to large benefit). The optimal information size was not met and inadequate sample size (<2000 participants). We downrate this domain by -1.5.
11. Confidence interval encompasses two ranges of effect (small but important benefit to large benefit). The optimal information size was met (a total of 98 events) but inadequate sample size (<2000 participants). We downrate this domain by -1.0.
12. Confidence interval encompasses two ranges of effect (small but important harm to large benefit). The optimal information size was not met (a total of 44 events) but inadequate sample size (<2000 participants). We downrate this domain by -2.0.
13. Confidence interval encompasses two ranges of effect (Moderate harm to moderate benefit). The optimal information size was not met (a total of 54 events) but inadequate sample size (<2000 participants). We downrate this domain by -2.0.
14. Confidence interval encompasses two ranges of effect (small but important benefit to large benefit). The optimal information size was met (a total of 157 events) but inadequate sample size (<2000 participants). We downrate this domain by -1.0.
15. Confidence interval encompasses two ranges of effect (small but important harm to large benefit). The optimal information size was not met (a total of 59 events) and inadequate sample size (<2000 participants). We downrate this domain by -2.0.
16. Confidence interval encompasses two ranges of effect (small but important benefit to large benefit). The optimal information size was met (a total of 98 events) but inadequate sample size (<2000 participants). We downrate this domain by -1.0.
17. Confidence interval encompasses two ranges of effect (moderate harm to moderate benefit). The optimal information size was met but the sample size was inadequate. We downrate by -1.5.

## Appendix H Table 15. E-cigarette with nicotine vs. other smoking cessation intervention (Usual Care) Smoking cessation, reduction, Quality of Life, adverse events

E-cigarette with nicotine plus fact sheet versus Usual care

**Bibliography:** Dawkins 2020; Date of last search January 2024

| **Certainty assessment** | | | | | | | | | **Summary of findings** | | | | | | | | | | | | | | |
| --- | --- | --- | --- | --- | --- | --- | --- | --- | --- | --- | --- | --- | --- | --- | --- | --- | --- | --- | --- | --- | --- | --- | --- |
| **№ of participants (studies) Follow-up** | **Risk of bias** | | **Inconsistency** | **Indirectness** | **Imprecision** | **Publication bias** | **Overall certainty of evidence** | **Study event rates (%)** | | | | | **Relative effect (95% CI)** | | | | **Anticipated absolute effects** | | | | | | |
|  |  |  |  |  |  |  |  | **e-cigarettes with nicotine** | | **Usual care** | | |  |  |  |  | **Risk with Usual care** | | | | | **Risk difference with e-cigarettes with nicotine** | |
| **Tobacco use abstinence: Sustained abstinence (follow up: 6 months)^a,b,c^**  Assessed with: CO-validated (<8 ppm [from 2-weeks post-quit date allowing up to 5 slips] Per-protocol analysis) | | | | | | | | | | | | | | | | | | | | | | | |
| 80  (1 RCT) | Very serious^d^ | | Not serious^e^ | Serious^f^ | Very serious^g^ | none^h^ | ⨁◯◯◯  VERY LOW | 3/35  (9%) | | 0/12  (0%) | | | **RR 2.52**  (0.13 to 45.69) | | | | 0 | | | | | 0 | |
| **Tobacco use abstinence: Sustained abstinence (follow up: 6 months)^a,b,c^**  Assessed with: CO-validated (<8 ppm [from 2-weeks post-quit date allowing up to 5 slips] Intention to treat analysis) | | | | | | | | | | | | | | | | | | | | | | | |
| 80  (1 RCT) | Very serious^d^ | | Not serious^e^ | Serious^f^ | Very serious^g^ | none^h^ | ⨁◯◯◯  VERY LOW | 3/48  (6.2%) | | 0/32  (0%) | | | | | **RR 4.71**  (0.25 to 88.3) | | | | 0 | | | | 0 |
| **Tobacco use abstinence: Point-prevalence abstinence (follow up: 6 months)^a,b,c^**  Assessed with: 7-day point prevalence (Per-protocol analysis) | | | | | | | | | | | | | | | | | | | | | | | |
| 80  (1 RCT) | | Very serious^d^ | Not serious^e^ | Serious^f^ | Very serious^g^ | none^h^ | ⨁◯◯◯  VERY LOW | 3/35  (9%) | | 0/12  (0%) | | | **RR 2.52**  (0.13 to 45.69) | | | | 0 | | | | | 0 | |
| **Reduction in tobacco smoking frequency/quantity: Reduction in the number of daily cigarettes smoked by at least 50% (follow up: 6 months)^a,b,c^**  Assessed with: self-reported, 50% reduction in cigarettes smoked per day (per protocol analysis) | | | | | | | | | | | | | | | | | | | | | | | |
| 80  (1 RCT) | Very serious^d^ | | Not serious^e^ | Serious^f^ | Very serious^i^ | none^h^ | ⨁◯◯◯  VERY LOW | 15/35  (43%) | | 3/12  (25%) | | | **RR 1.71**  (0.59 to 4.9) | | | | 250 per 1000 | | | | | 178 more per 1000  (103 fewer to 975 more) | |
| **Reduction in tobacco smoking frequency/quantity: eCO levels (follow up: 6 months)^a,b,c^**  Assessed with: Self-reported, 50% reduction in expired CO (per protocol analysis) | | | | | | | | | | | | | | | | | | | | | | | |
| 80  (1 RCT) | Very serious^d^ | | Not serious^e^ | Serious^f^ | Very serious^i^ | none^h^ | ⨁◯◯◯  VERY LOW | 7/35  (20%) | | | 3/12  (25%) | | | **RR 0.80**  (0.24 to 2.61) | | | | 250 per 1000 | | | 50 fewer per 1000  (190 fewer to 403 more) | | |
| **Adverse events: Mild adverse events (follow up: 6 months)^a,b,c^**  Assessed with: self-reported, Mental health status measured using the 9-item Patient Health Questionnaire (PHQ-9) for depression and | | | | | | | | | | | | | | | | | | | | | | | |
| 80  (1 RCT) | Very serious^d^ | | Not serious^e^ | Serious^f^ | Very serious^g^ | none^h^ | ⨁◯◯◯  VERY LOW | 40 | | | | 40 | | | | - | | | | **EC group:** The Mean (SD) scores for mental health at 24 weeks follow for the PHQ-9 questionnaire 7.12 (7.22).  **Usual care group**: The Mean (SD) scores for mental health at 24 weeks follow up was 10.82 (7.23) for the PHQ-9 questionnaire. | | | |
| **Adverse events: Mild adverse events (follow up: 6 months)^a,b,c^**  Assessed with: self-reported, Mental health status measured using the 7-item Generalised Anxiety Disorder (GAD) questionnaire | | | | | | | | | | | | | | | | | | | | | | | |
| 80  (1 RCT) | Very serious^d^ | | Not serious^e^ | Serious^f^ | Very serious^g^ | none^h^ | ⨁◯◯◯  VERY LOW | 40 | | | | 40 | | | | - | | | | **EC group:** The Mean (SD) scores for mental health at 24 weeks follow up was 5.63(6.34) for the GAD questionnaire  **Usual care group**: The Mean (SD) scores for mental health at 24 weeks follow up was 12.70 (4.42) for the GAD questionnaire | | | |
| **Quality of Life: Health Related Quality of Life (EQ5D-3L; per-protocol analysis) (follow up: 6 months)^a,b,c^**  Assessed with: Self-reported, measured using the EQ5D-3L [descriptive system converted to a utility value based on UK population tariff, ranging from 0 (death) to 1 (perfect health). | | | | | | | | | | | | | | | | | | | | | | | |
| 80  (1 RCT) | Very serious^d^ | | Not serious^e^ | Serious^f^ | Very serious^g^ | none^h^ | ⨁◯◯◯  VERY LOW | EC group: The Mean (SD) QoL score at 24 weeks follow-up was 0.653 (0.36)  Usual care group: The Mean (SD) QoL score at 24 weeks follow-up was 0.691(0.238); MD: 0.03 lower (0.18 lower points to 0.10 higher) | | | | | | | | | | | | | | | |
| **Quality of Life: Health Related Quality of Life (HRQoL EQ - Visual Analogue Scales (VAS) (follow up: 6 months)^a,b,c^**  Assessed with: Self-reported, HRQoL EQ VAS (perceived health on the day of administration, ranging from 0 (death) to 100 (perfect health). | | | | | | | | | | | | | | | | | | | | | | | |
| 80  (1 RCT) | Very serious^d^ | | Not serious^e^ | Serious^f^ | Very serious^g^ | none^h^ | ⨁◯◯◯  VERY LOW | EC group: The Mean (SD) QoL score at 24 weeks follow-up was 61.8 (21.6)  Usual care group: The Mean (SD) QoL score at 24 weeks follow-up was  61 (22.5); MD: 0.8 higher (from 9.17 points lower to 10.77 higher) | | | | | | | | | | | | | | | |

**CI:** Confidence interval; **RR**: Risk Ratio; **Mean (SD)**: Mean (Standard deviation)

#### Explanations

1. Motivation to quit smoking in study participants varied considerably; although, 6.3% reported not wanting to stop smoking.
2. No co-interventions reported
3. Study participants received E-cigarette starter kit (a tank-style refillable EC with a choice of nicotine strength e-liquid (12 or 18 mg/ml and flavours) and guide to e-cigarettes fact sheet
4. High risk for randomization, blinding of outcome assessors and allocation concealment. We downrate by -2.0.
5. One study only, we do not downrate this domain.
6. Participants in the intervention arm were provided with a starter kit comprising of a tank-style refillable EC with a choice of a) nicotine strength e-liquid [12 & 18 mg/mL] and b) flavours [3 options]. Since participants were advised to try EC and nicotine strengths of their own, we downrate the domain by -1.0.
7. Unable to assess confidence intervals. The optimal information size and the sample size was not met. We downrate by -2.0.
8. Literature search was comprehensive. No suspicion of suppression of results. We do not downrate this domain.
9. Confidence interval encompasses two ranges of effect (Moderate harm to large benefit). The Optimal information size was not met and the inadequate sample size (<2000 participants). We downrate this domain by -2.0.

| Summary of findings: | | | | | | |
| --- | --- | --- | --- | --- | --- | --- |
| **E-cigarettes with nicotine compared to other interventions (Quit advice) in the general/mixed population of smokers** | | | | | | |
| **Patient or population**: General/mixed population of smokers  **Setting**: Homeless centres  **Intervention**: Electronic cigarettes with nicotine  **Comparison**: Other interventions (Usual care) | | | | | | |
| Outcomes | **Anticipated absolute effects^*^** (95% CI) | | Relative effect (95% CI) | № of participants  (studies) | Certainty of the evidence (GRADE) | Comments |
|  | **Risk with usual care** | **Risk with e-cigarettes with nicotine** |  |  |  |  |
| Tobacco use abstinence: Sustained abstinence (per-protocol analysis)^a,b,c^  Follow up: 6 months | 0 | 0 | **RR 2.52**  (0.13 to 45.69) | 80  (1 RCT) | ⨁◯◯◯  VERY LOW^d,e,f,g,h^ |  |
| Tobacco use abstinence: Sustained abstinence (Intention to treat [ITT] analysis) ^a,b,c^  Follow up: 6 months | 0 | 0 | **RR 4.71**  (0.25 to 88.3) | 80  (1 RCT) | ⨁◯◯◯  VERY LOW^d,e,f,g,h^ |  |
| Tobacco use abstinence: Point-prevalence abstinence (Intention to treat [ITT] analysis) ^a,b,c^  Follow up: 6 months | 0 | 0 | **RR 2.52**  (0.13 to 45.69) | 80  (1 RCT) | ⨁◯◯◯  VERY LOW^d,e,f,g,h^ |  |
| Reduction in Tobacco smoking frequency/quantity ^a,b,c^  Follow up: 6 months | 250 per 1000 | 177.5 more per 1000  (102.5 fewer to 975 more) | **RR 1.71**  (0.59 to 4.9) | 80  (1 RCT) | ⨁◯◯◯  VERY LOW^d,e,f,I,h^ |  |
| Reduction in Tobacco smoking frequency/quantity: eCO levels ^a,b,c^  Follow up: 6 months | 250 per 1000 | 50 fewer per 1000  (190 fewer to 402.5 more) | **RR 0.80**  (0.24 to 2.61) | 80  (1 RCT) | ⨁◯◯◯  VERY LOW^d,e,f,I,h^ |  |
| Adverse events (Mild) ^a,b,c^  Follow up: 6 months | **EC group:** The Mean (SD) scores for mental health at 24 weeks follow for the PHQ-9 questionnaire 7.12 (7.22).  **Usual care group**: The Mean (SD) scores for mental health at 24 weeks follow up was 10.82 (7.23) for the PHQ-9 questionnaire. | | - | 80  (1 RCT) | ⨁◯◯◯  VERY LOW^d,e,f,g,h^ |  |
| Adverse events (Mild) ^a,b,c^  Follow up: 6 months | **EC group:** The Mean (SD) scores for mental health at 24 weeks follow up was 5.63(6.34) for the GAD questionnaire  **Usual care group**: The Mean (SD) scores for mental health at 24 weeks follow up was 12.70 (4.42) for the GAD questionnaire | | - | 80  (1 RCT) | ⨁◯◯◯  VERY LOW^d,e,f,g,h^ |  |
| Quality of Life (EQ5D-3L; per-protocol analysis) ^a,b,c^ | EC group: The Mean (SD) QoL score at 24 weeks follow-up was 0.653 (0.36)  Usual care group: The Mean (SD) QoL score at 24 weeks follow-up was 0.691(0.238) | | - | 80  (1 RCT) | ⨁◯◯◯  VERY LOW^d,e,f,g,h^ |  |
| Quality of Life HRQoL EQ - Visual Analogue Scales (VAS) ^a,b,c^ | EC group: The Mean (SD) QoL score at 24 weeks follow-up was 61.8 (21.6)  Usual care group: The Mean (SD) QoL score at 24 weeks follow-up was  61 (22.5) | | - | 80  (1 RCT) | ⨁◯◯◯  VERY LOW^d,e,f,g,h^ |  |
| ***The risk in the intervention group** (and its 95% confidence interval) is based on the assumed risk in the comparison group and the **relative effect** of the intervention (and its 95% CI).  **CI:** Confidence interval; **MD:** Mean difference | | | | | | |
| **GRADE Working Group grades of evidence** **High certainty:** We are very confident that the true effect lies close to that of the estimate of the effect **Moderate certainty:** We are moderately confident in the effect estimate: The true effect is likely to be close to the estimate of the effect, but there is a possibility that it is substantially different **Low certainty:** Our confidence in the effect estimate is limited: The true effect may be substantially different from the estimate of the effect **Very low certainty:** We have very little confidence in the effect estimate: The true effect is likely to be substantially different from the estimate of effect | | | | | | |
| **CI:** Confidence interval; **RR**: Risk Ratio; **Mean (SD)**: Mean (Standard deviation) | | | | | | |

#### Explanations

1. Motivation to quit smoking in study participants varied considerably; although, 6.3% reported not wanting to stop smoking.
2. No co-interventions reported
3. Study participants received E-cigarette starter kit (a tank-style refillable EC with a choice of nicotine strength e-liquid (12 or 18 mg/ml and flavours) and guide to e-cigarettes fact sheet
4. High risk for randomization, blinding of outcome assessors and allocation concealment. We downrate by -2.0.
5. One study only, we do not downrate this domain.
6. Participants in the intervention arm were provided with a starter kit comprising of a tank-style refillable EC with a choice of a) nicotine strength e-liquid [12 & 18 mg/mL] and b) flavours [3 options]. Since participants were advised to try EC and nicotine strengths of their own, we downrate the domain by -1.0.
7. Unable to assess confidence intervals. The optimal information size and the sample size was not met. We downrate by -2.0.
8. Literature search was comprehensive. No suspicion of suppression of results. We do not downrate this domain.
9. Confidence interval encompasses two ranges of effect (Moderate harm to large benefit). The Optimal information size was not met and the inadequate sample size (<2000 participants). We downrate this domain by -2.0.

## Appendix H Table 16. E-cigarette with nicotine + Support (psychological counselling) vs. E-cigarette without nicotine + Support (psychological counselling) Smoking cessation, reduction, adverse events

E-cigarette with nicotine + psychological counselling Vs. E-cigarette without nicotine + psychological counselling

**Bibliography:** Lucchiari 2022; Date of last search January 2024

| **Certainty assessment** | | | | | | | | **Summary of findings** | | | | | | | | |
| --- | --- | --- | --- | --- | --- | --- | --- | --- | --- | --- | --- | --- | --- | --- | --- | --- |
| **№ of participants (studies) Follow-up** | **Risk of bias** | **Inconsistency** | **Indirectness** | **Imprecision** | **Publication bias** | **Overall certainty of evidence** | **Study event rates (%)** | | | | **Relative effect (95% CI)** | | **Anticipated absolute effects** | | | |
|  |  |  |  |  |  |  | **e-cigarettes with nicotine + Support** | | **e-cigarettes without nicotine + Support** | |  |  | **e-cigarettes without nicotine + Support** | | | **Risk difference with e-cigarettes with nicotine + Support** |
| **Tobacco use abstinence: Complete abstinence (follow up: 12 months)^a,b,c^**  Assessed with: Self-reported, validated by eCO levels, that must be under or equal 7 ppm | | | | | | | | | | | | | | | | |
| 140  (1 RCT) | Serious^d^ | Not serious^e^ | Not serious^f^ | Very serious^g^ | none^h^ | ⨁◯◯◯  VERY LOW | 15/60  (25%) | | 15/58  (26%) | | **RR 0.96**  (0.52 to 1.79) | | 259 per 1000 | | | 10 fewer per 1000  (124 fewer to 204 more) |
| **Reduction in tobacco smoking frequency/quantity: Daily cigarettes smoked (follow up: 12 months)^a,b,c^**  Assessed with: Self-reported, Mean number of daily cigarettes smoked | | | | | | | | | | | | | | | | |
| 140  (1 RCT) | Serious^d^ | Not serious^e^ | Not serious^f^ | Serious^i^ | none^h^ | ⨁⨁◯◯ LOW | 60 | | | 58 | | - | | The mean reduction in smoking frequency was 13.71 (7.22). | Reduction by MD 2.4(4.88 greater reduction to 0.05) | |
| **Adverse events: Mild adverse events (follow up: 12 months)^a,b,c^**  Assessed with: self-reported, measured using hospital anxiety and depression scale (HADS) | | | | | | | | | | | | | | | | |
| 140  (1 RCT) | Serious^d^ | Not serious^e^ | Not serious^f^ | Serious to very serious^j^ | none^h^ | ⨁◯◯◯  VERY LOW | E-cigs with Nicotine plus support group: The Mean (SD) scores for mental health at 12 months follow up was 12.17 (2.20) for Anxiety and 9.13 (1.57) for Depression in the Hospital Anxiety and Depression Scale (HADS).  E-cigs without Nicotine plus support group: The Mean (SD) scores for mental health at 12 months follow up was 12.45 (2.37) for Anxiety and 8.90 (1.81) for Depression in the Hospital Anxiety and Depression Scale (HADS). | | | | | | | | | |
| **CI:** Confidence interval; **RR**: Risk Ratio; **Mean (SD)**: Mean (Standard deviation); **MD:** Mean difference | | | | | | | | | | | | | | | | |

#### Explanations

1. Study participants included had motivational score > 10 (i.e., a high motivation to stop smoking) and not be treated at a smoking center.
2. Psychological counselling was reported as co-intervention provided in both the groups.
3. Study participants received E-cigarette starter kit (a tank-style refillable EC with an 8 mg/ml nicotine strength e-liquid (12 10-ml liquid cartridges) in the intervention group and E-cigarette starter kit (a tank-style refillable EC with a 0 mg/ml nicotine strength e-liquid (12 10-ml liquid cartridges) in the control group
4. There were some concerns related to sequence generation and allocation concealment. Therefore, we rate down the domain by -1.0.
5. One study only. We do not downrate this domain.
6. No indirectness, we do not downrate this domain.
7. Confidence interval encompasses two ranges (moderate harm and moderate benefit). The optimal information size and the sample size was not met. We downrate this domain by -2.0.
8. Literature search was comprehensive. No suspicion of suppression of results. We do not downrate this domain.
9. Confidence interval encompasses little to no difference. The optimal information size not met and inadequate sample size (<400 participants for a continuous outcome). We downrate this domain by -1.0.
10. Unable to assess confidence intervals. Unclear sample size analyzed but a maximum of 300 total participants (with several missing participants) could be included. Thus, the optimal information size cannot be met. We cannot rate this domain.

| Summary of findings: | | | | | | |
| --- | --- | --- | --- | --- | --- | --- |
| **E-cigarettes with nicotine compared to other interventions (Quit advice) in the general/mixed population of smokers** | | | | | | |
| **Patient or population**: Chronic smokers with high motivation to quit  **Setting**: Academic research setting (University of Milan & European Institute of Oncology)  **Intervention**: Electronic cigarettes with nicotine + psychological counselling  **Comparison**: Electronic cigarettes without nicotine + psychological counselling | | | | | | |
| Outcomes | **Anticipated absolute effects^*^** (95% CI) | | Relative effect (95% CI) | № of participants  (studies) | Certainty of the evidence (GRADE) | Comments |
|  | **Risk with e-cigarettes without nicotine + Support** | **Risk with e-cigarettes with nicotine** |  |  |  |  |
| Tobacco use abstinence: Complete abstinence^a,b,c^  Follow up: 12 months | 259 per 1000 | 10.3 fewer per 1000  (124.1 fewer to 204.3more) | **RR 0.96**  (0.52 to 1.79) | 140  (1 RCT) | ⨁◯◯◯  VERY LOW^d,e,f,g,h^ |  |
| Reduction in tobacco smoking frequency/quantity: Daily cigarettes smoked^a,b,c^  Follow up: 12 months | Smokers in intervention arm smoked a mean of 16.18 tobacco cigarettes (SD=7.23) versus a mean of 13.71 (7.22) cigarettes smoked in control arm at month 12. | | - | 140  (1 RCT) | ⨁⨁◯◯ LOW^d,e,f,I,h^ |  |
| Adverse events: Mild^a,b,c^  Follow up: 12 months | E-cigs with Nicotine plus support group: The Mean (SD) scores for mental health at 12 months follow up was 12.17 (2.20) for Anxiety and 9.13 (1.57) for Depression in the Hospital Anxiety and Depression Scale (HADS).  E-cigs without Nicotine plus support group: The Mean (SD) scores for mental health at 12 months follow up was 12.45 (2.37) for Anxiety and 8.90 (1.81) for Depression in the Hospital Anxiety and Depression Scale (HADS). | | - | 140  (1 RCT) | ⨁◯◯◯  VERY LOW^d,e,f,j,h^ |  |
| ***The risk in the intervention group** (and its 95% confidence interval) is based on the assumed risk in the comparison group and the **relative effect** of the intervention (and its 95% CI).  **CI:** Confidence interval; **RR**: Risk Ratio; **Mean (SD)**: Mean (Standard deviation); **MD:** Mean difference | | | | | | |
| **GRADE Working Group grades of evidence** **High certainty:** We are very confident that the true effect lies close to that of the estimate of the effect **Moderate certainty:** We are moderately confident in the effect estimate: The true effect is likely to be close to the estimate of the effect, but there is a possibility that it is substantially different **Low certainty:** Our confidence in the effect estimate is limited: The true effect may be substantially different from the estimate of the effect **Very low certainty:** We have very little confidence in the effect estimate: The true effect is likely to be substantially different from the estimate of effect | | | | | | |
|  | | | | | | |

#### Explanations

1. Study participants included had motivational score > 10 (i.e., a high motivation to stop smoking) and not be treated at a smoking center.
2. Psychological counselling was reported as co-intervention provided in both the groups.
3. Study participants received E-cigarette starter kit (a tank-style refillable EC with an 8 mg/ml nicotine strength e-liquid (12 10-ml liquid cartridges) in the intervention group and E-cigarette starter kit (a tank-style refillable EC with a 0 mg/ml nicotine strength e-liquid (12 10-ml liquid cartridges) in the control group
4. There were some concerns related to sequence generation and allocation concealment. Therefore, we rate down the domain by -1.0.
5. One study only. We do not downrate this domain.
6. No indirectness, we do not downrate this domain.
7. Confidence interval encompasses two ranges (moderate harm and moderate benefit). The optimal information size and the sample size was not met. We downrate this domain by -2.0.
8. Literature search was comprehensive. No suspicion of suppression of results. We do not downrate this domain.
9. Confidence interval encompasses little to no difference. The optimal information size not met and inadequate sample size (<400 participants for a continuous outcome). We downrate this domain by -1.0.
10. Unable to assess confidence intervals. Unclear sample size analyzed but a maximum of 300 total participants (with several missing participants) could be included. Thus, the optimal information size cannot be met. We cannot rate this domain.

## Appendix H Table 17. E-cigarette with nicotine + Support (psychological counselling) vs. Support (psychological counselling) Smoking cessation, reduction

E-cigarette with nicotine + psychological counselling Vs. psychological counselling

**Bibliography:** Lucchiari 2022; Date of last search January 2024

| **Certainty assessment** | | | | | | | | **Summary of findings** | | | | | | | | |
| --- | --- | --- | --- | --- | --- | --- | --- | --- | --- | --- | --- | --- | --- | --- | --- | --- |
| **№ of participants (studies) Follow-up** | **Risk of bias** | **Inconsistency** | **Indirectness** | **Imprecision** | **Publication bias** | **Overall certainty of evidence** | **Study event rates (%)** | | | | **Relative effect (95% CI)** | | **Anticipated absolute effects** | | | |
|  |  |  |  |  |  |  | **e-cigarettes with nicotine + Support** | | **Support** | |  |  | **Risk with Support** | | **Risk difference with e-cigarettes with nicotine + Support** | |
| **Tobacco use abstinence: Complete abstinence (follow up: 12 months)^a,b,c^**  Assessed with: Self-reported, validated by eCO levels, that must be under or equal 7 ppm | | | | | | | | | | | | | | | | |
| 140  (1 RCT) | Serious^d^ | Not serious^e^ | Not serious^f^ | Very serious^g^ | none^h^ | ⨁◯◯◯  VERY LOW | 15/60  (25%) | | 10/60  (16.7%) | | **RR 1.5**  (0.73 to 3.06) | | 167 per 1000 | | 83 more per 1000  (45 fewer to 343 more) | |
| **Reduction in tobacco smoking frequency/quantity: Daily cigarettes smoked (follow up: 12 months)^a,b,c^**  Assessed with: Self-reported, Mean number of daily cigarettes smoked | | | | | | | | | | | | | | | | |
| 140  (1 RCT) | Serious^d^ | Not serious^e^ | Not serious^f^ | Serious^i^ | none^h^ | ⨁⨁◯◯ LOW | 60 | | | 60 | - | The mean reduction in smoking frequency was 13.93 (7.20). | | Reduction by MD 2.2(0.35 to 4.85 greater reduction) | |  |

**CI:** Confidence interval; **MD:** Mean difference

#### Explanations

1. Study participants included had motivational score > 10 (i.e., a high motivation to stop smoking) and not be treated at a smoking center.
2. Psychological counselling was reported as co-intervention provided in both the groups.
3. Study participants received E-cigarette starter kit (a tank-style refillable EC with a 8 mg/ml nicotine strength e-liquid (12 10-ml liquid cartridges) in the intervention group and E-cigarette starter kit (a tank-style refillable EC with a 0 mg/ml nicotine strength e-liquid (12 10-ml liquid cartridges) in the control group
4. There were some concerns related to sequence generation and allocation concealment. Therefore, we rate down the domain by -1.0.
5. One study only. We do not downrate this domain.
6. No indirectness, we do not downrate this domain.
7. Confidence interval encompasses two ranges (Moderate harm and large benefit). The optimal information size was not met (a total of 25 events) and inadequate sample size (<2000 participants). We downrate this domain by -2.0.
8. Literature search was comprehensive. No suspicion of suppression of results. We do not downrate this domain.
9. Confidence interval encompasses little to no difference. The optimal information size not met and inadequate sample size (<400 participants for a continuous outcome). We downrate this domain by -1.0.

| Summary of findings: | | | | | | |
| --- | --- | --- | --- | --- | --- | --- |
| **E-cigarettes with nicotine compared to other interventions (Quit advice) in the general/mixed population of smokers** | | | | | | |
| **Patient or population**: Chronic smokers with high motivation to quit  **Setting**: Academic research setting (University of Milan & European Institute of Oncology)  **Intervention**: Electronic cigarettes with nicotine + psychological counselling  **Comparison**: Electronic cigarettes without nicotine + psychological counselling | | | | | | |
| Outcomes | **Anticipated absolute effects^*^** (95% CI) | | Relative effect (95% CI) | № of participants  (studies) | Certainty of the evidence (GRADE) | Comments |
|  | **Risk with no intervention** | **Risk with e-cigarettes with nicotine** |  |  |  |  |
| Tobacco use abstinence: Complete abstinence^a,b,c^  Follow up: 12 months | 167 per 1000 | 83 more per 1000  (45 fewer to 343 more) | **RR 1.5**  (0.73 to 3.06) | 140  (1 RCT) | ⨁◯◯◯  VERY LOW^d,e,f,g,h^ |  |
| Reduction in tobacco smoking frequency/quantity: Daily cigarettes smoked^a,b,c^  Follow up: 12 months | E-cigs with Nicotine plus support group: The Mean (SD) scores for mental health at 12 months follow up was 12.17 (2.20) for Anxiety and 9.13 (1.57) for Depression in the Hospital Anxiety and Depression Scale (HADS).  Support group only: The Mean (SD) scores for mental health at 12 months follow up was 12.12 (2.24) for Anxiety and 8.32 (1.37) for Depression in the Hospital Anxiety and Depression Scale (HADS). | | - | 140  (1 RCT) | ⨁⨁◯◯ LOW^d,e,f,i,h^ |  |
| ***The risk in the intervention group** (and its 95% confidence interval) is based on the assumed risk in the comparison group and the **relative effect** of the intervention (and its 95% CI).  **CI:** Confidence interval; **MD:** Mean difference | | | | | | |
| **GRADE Working Group grades of evidence** **High certainty:** We are very confident that the true effect lies close to that of the estimate of the effect **Moderate certainty:** We are moderately confident in the effect estimate: The true effect is likely to be close to the estimate of the effect, but there is a possibility that it is substantially different **Low certainty:** Our confidence in the effect estimate is limited: The true effect may be substantially different from the estimate of the effect **Very low certainty:** We have very little confidence in the effect estimate: The true effect is likely to be substantially different from the estimate of effect | | | | | | |
|  | | | | | | |

#### Explanations

1. Study participants included had motivational score > 10 (i.e., a high motivation to stop smoking) and not be treated at a smoking center.
2. Psychological counselling was reported as co-intervention provided in both the groups.
3. Study participants received E-cigarette starter kit (a tank-style refillable EC with a 8 mg/ml nicotine strength e-liquid (12 10-ml liquid cartridges) in the intervention group and E-cigarette starter kit (a tank-style refillable EC with a 0 mg/ml nicotine strength e-liquid (12 10-ml liquid cartridges) in the control group
4. There were some concerns related to sequence generation and allocation concealment. Therefore, we rate down the domain by -1.0.
5. One study only. We do not downrate this domain.
6. No indirectness, we do not downrate this domain.
7. Confidence interval encompasses two ranges (Moderate harm and large benefit). The optimal information size was not met (a total of 25 events) and inadequate sample size (<2000 participants). We downrate this domain by -2.0.
8. Literature search was comprehensive. No suspicion of suppression of results. We do not downrate this domain.
9. Confidence interval encompasses little to no difference. The optimal information size not met and inadequate sample size (<400 participants for a continuous outcome). We downrate this domain by -1.0.

## Appendix H Table 18. E-cigarette with nicotine + vs. Non-nicotine cigarette substitute Smoking cessation, reduction

E-cigarette with nicotine Vs. Non-nicotine cigarette substitute (plastic tube with no electronics or aerosol)

**Bibliography:** Foulds 2022; Date of last search January 2024

| **Certainty assessment** | | | | | | | | **Summary of findings** | | | | | | | | | | |
| --- | --- | --- | --- | --- | --- | --- | --- | --- | --- | --- | --- | --- | --- | --- | --- | --- | --- | --- |
| **№ of participants (studies) Follow-up** | **Risk of bias** | **Inconsistency** | **Indirectness** | **Imprecision** | **Publication bias** | **Overall certainty of evidence** | **Study event rates (%)** | | | | | **Relative effect (95% CI)** | | **Anticipated absolute effects** | | | | |
|  |  |  |  |  |  |  | **e-cigarettes with nicotine** | | **Non-nicotine cigarette substitute** | | |  |  | **Risk with Non-nicotine cigarette substitute** | | | | **Risk difference with e-cigarettes with nicotine** |
| **Tobacco use abstinence: 7-day point prevalence abstinence (follow up: 6 months)^a,b,c^**  Assessed with: Self-reported, biochemically validated by eCO<10 ppm | | | | | | | | | | | | | | | | | | |
| 260  (1 RCT) | Serious^d^ | Not serious^e^ | Not serious^f^ | Serious to very serious^g^ | none^h^ | ⨁◯◯◯  VERY LOW | 14/130  (10.8%) | | 4/130  (3.1%) | | | **RR 3.5**  (1.2 to 10.4) | | 31 per 1000 | | | | 77 more per 1000  (6 more to 289 more) |
| **Tobacco use abstinence: 28+days abstinence (follow up: 6 months)^a,b,c^**  Assessed with: Self-reported, biochemically validated by eCO<10 ppm | | | | | | | | | | | | | | | | | | |
| 260  (1 RCT) | Serious^d^ | Not serious^e^ | Not serious^f^ | Serious to very serious^i^ | none^h^ | ⨁◯◯◯  VERY LOW | 10/130  (7.7%) | | | 2/130  (1.5%) | | | **RR 5.5**  (1.2 to 24.3) | | 15 per 1000 | | 69 more per 1000  (3 more to 358 more) | |
| **Tobacco use abstinence: Total number of days being totally abstinent (follow up: 6 months)^a,b,c^**  Assessed with: Self-reported, Mean days on which participants reported being abstinent from cigarettes from week 1 to week 24 | | | | | | | | | | | | | | | | | | |
| 260  (1 RCT) | Serious^d^ | Not serious^e^ | Not serious^f^ | Serious^j^ | none^h^ | ⨁⨁◯◯ LOW | 130 | | | | 130 | - | | | | The mean number of days where patient reported being totally abstinent was 5.3 (18.5). | MD reported between groups was 10.29 (3.2 to 17.4 greater). | |

**CI:** Confidence interval; **MD:** Mean difference; **RR**: Risk Ratio

#### Explanations

1. Study participants included had no plans to quit smoking within 6 months.
2. No co-intervention reported.
3. Study participants received an eGo-style ENDS with nicotine liquid (36 mg/ml) either with tobacco or menthol flavour of their choice in the intervention group and a cigarette-shaped plastic tube with no electronics or aerosol as a cigarette substitute (CS).
4. There were some concerns related to sequence generation and allocation concealment. Therefore, we rate down the domain by -1.0.
5. One study only. We do not downrate this domain.
6. No indirectness, we do not downrate this domain.
7. Confidence interval encompasses two ranges of effect (small but important benefit to large benefit. The optimal information size was not met (a total of 18 events) and inadequate sample size. We downrate this domain by -1.5.
8. Literature search was comprehensive. No suspicion of suppression of results. We do not downrate this domain.
9. Confidence interval encompasses two ranges of effect (small but important benefit to large benefit. The optimal information size was not met (a total of 12 events) and inadequate sample size. We downrate this domain by -1.5.
10. Confidence interval encompasses one range of effect (small but important benefit to little to no difference). The optimal information size not met and inadequate sample size (<400 participants for a continuous outcome). We downrate this domain by -1.0.

| Summary of findings: | | | | | | |
| --- | --- | --- | --- | --- | --- | --- |
| **E-cigarettes with nicotine compared to other interventions (Quit advice) in the general/mixed population of smokers** | | | | | | |
| **Patient or population**: Current smokers with no plans to quit  **Setting**: Academic medical centres (Penn State Hershey and Virginia Commonwealth University  **Intervention**: Electronic cigarettes with nicotine  **Comparison**: Non-nicotine cigarette substitute (plastic tube with no electronics or aerosol) | | | | | | |
| Outcomes | **Anticipated absolute effects^*^** (95% CI) | | Relative effect (95% CI) | № of participants  (studies) | Certainty of the evidence (GRADE) | Comments |
|  | **Risk with Non-nicotine cigarette substitute** | **Risk with e-cigarettes with nicotine** |  |  |  |  |
| Tobacco use abstinence: 7-day point prevalence abstinence^a,b,c^  Follow up: 6 months | 31 per 1000 | 77 more per 1000  (6 more to 289 more) | **RR 3.5**  (1.2 to 10.4) | 520  (1 RCT) | ⨁◯◯◯  VERY LOW^d,e,f,g,h^ |  |
| Tobacco use abstinence: 28+ days abstinence^a,b,c^  Follow up: 6 months | 15 per 1000 | 69 more per 1000  (3 more to 358 more) | **RR 5.5**  (1.2 to 24.3) | 520  (1 RCT) | ⨁◯◯◯  VERY LOW^d,e,f,I,h^ |  |
| Tobacco use abstinence: Total number of days being totally abstinent^a,b,c^  Follow up: 6 months | The Mean (SD) number of days in the e-cigs with nicotine group where the participants reported being totally abstinent was 15.6 (36.4) and that in the control group was 5.3 (18.5). Difference of mean (95% CI) reported between both groups was **10.29 (3.2 to 17.4)**. | | - | 520  (1 RCT) | ⨁⨁◯◯ LOW^d,e,f,j,h^ |  |
| ***The risk in the intervention group** (and its 95% confidence interval) is based on the assumed risk in the comparison group and the **relative effect** of the intervention (and its 95% CI).  **CI:** Confidence interval; **MD:** Mean difference; **RR**: Risk Ratio | | | | | | |
| **GRADE Working Group grades of evidence** **High certainty:** We are very confident that the true effect lies close to that of the estimate of the effect **Moderate certainty:** We are moderately confident in the effect estimate: The true effect is likely to be close to the estimate of the effect, but there is a possibility that it is substantially different **Low certainty:** Our confidence in the effect estimate is limited: The true effect may be substantially different from the estimate of the effect **Very low certainty:** We have very little confidence in the effect estimate: The true effect is likely to be substantially different from the estimate of effect | | | | | | |
|  | | | | | | |

#### Explanations

1. Study participants included had no plans to quit smoking within 6 months.
2. No co-intervention reported.
3. Study participants received an eGo-style ENDS with nicotine liquid (36 mg/ml) either with tobacco or menthol flavour of their choice in the intervention group and a cigarette-shaped plastic tube with no electronics or aerosol as a cigarette substitute (CS).
4. There were some concerns related to sequence generation and allocation concealment. Therefore, we rate down the domain by -1.0.
5. One study only. We do not downrate this domain.
6. No indirectness, we do not downrate this domain.
7. Confidence interval encompasses two ranges of effect (small but important benefit to large benefit. The optimal information size was not met (a total of 18 events) and inadequate sample size. We downrate this domain by -1.5.
8. Literature search was comprehensive. No suspicion of suppression of results. We do not downrate this domain.
9. Confidence interval encompasses two ranges of effect (small but important benefit to large benefit. The optimal information size was not met (a total of 12 events) and inadequate sample size. We downrate this domain by -1.5.
10. Confidence interval encompasses one range of effect (small but important benefit to little to no difference). The optimal information size not met and inadequate sample size (<400 participants for a continuous outcome). We downrate this domain by -1.0.

## Appendix H Table 19. E-cigarette with nicotine + vs. E-cigarette without nicotine Smoking cessation, reduction

E-cigarette with nicotine Vs. E-cigarette without nicotine

**Bibliography:** Foulds 2022; Date of last search January 2024

| **Certainty assessment** | | | | | | | | **Summary of findings** | | | | | | | | | | |
| --- | --- | --- | --- | --- | --- | --- | --- | --- | --- | --- | --- | --- | --- | --- | --- | --- | --- | --- |
| **№ of participants (studies) Follow-up** | **Risk of bias** | **Inconsistency** | **Indirectness** | **Imprecision** | **Publication bias** | **Overall certainty of evidence** | **Study event rates (%)** | | | | | **Relative effect (95% CI)** | | **Anticipated absolute effects** | | | | |
|  |  |  |  |  |  |  | **e-cigarettes with nicotine** | | **e-cigarettes without nicotine** | | |  |  | **Risk with e-cigarettes without nicotine** | | | | **Risk difference with e-cigarettes with nicotine** |
| **Tobacco use abstinence: 7-day point prevalence abstinence (follow up: 6 months)^a,b,c^**  Assessed with: Self-reported, biochemically validated by eCO<10 ppm | | | | | | | | | | | | | | | | | | |
| 260  (1 RCT) | Serious^d^ | Not serious^e^ | Not serious^f^ | Serious to very serious^g^ | none^h^ | ⨁◯◯◯  VERY LOW | 14/130  (10.8%) | | 1/130  (0.8%) | | | **RR 14**  (1.9 to 104.9) | | 8 per 1000 | | | | 100 more per 1000  (7 more to 799 more) |
| **Tobacco use abstinence: 28+days abstinence (follow up: 12 months)^a,b,c^**  Assessed with: Self-reported, biochemically validated by eCO<10 ppm | | | | | | | | | | | | | | | | | | |
| 260  (1 RCT) | Serious^d^ | Not serious^e^ | Not serious^f^ | Serious to very serious^i^ | none^h^ | ⨁◯◯◯  VERY LOW | 10/130  (7.7%) | | | 1/130  (0.8%) | | | **RR 11**  (1.4 to 84) | | 8 per 1000 | | 77 more per 1000  (3 more to 638 more) | |
| **Tobacco use abstinence: Total number of days being totally abstinent (follow up: 12 months)^a,b,c^**  Assessed with: Self-reported, Mean days on which participants reported being abstinent from cigarettes from week 1 to week 24 | | | | | | | | | | | | | | | | | | |
| 260  (1 RCT) | Serious^d^ | Not serious^e^ | Not serious^f^ | Serious^j^ | none^h^ | ⨁⨁◯◯ LOW | 130 | | | | 130 | - | | | | The mean number of days where patient reported being totally abstinent was 4.7 (17). | MD reported between groups was 10.87 (3.9 to 17.8 greater). | |

**CI:** Confidence interval; **MD:** Mean difference; **RR**: Risk Ratio

#### Explanations

1. Study participants included had no plans to quit smoking within 6 months.
2. No co-intervention reported.
3. Study participants received an eGo-style ENDS with nicotine liquid (8 or 36 mg/ml) either with tobacco or menthol flavour of their choice in the intervention group and a e-cigarette without nicotine (placebo; 0mg/ml).
4. There were some concerns related to sequence generation and allocation concealment. Therefore, we rate down the domain by -1.0.
5. One study only. We do not downrate this domain.
6. No indirectness, we do not downrate this domain.
7. Confidence interval encompasses two ranges of effect (small but important benefit to large benefit. The optimal information size was not met (a total of 15 events) and inadequate sample size. We downrate this domain by -1.5.
8. Literature search was comprehensive. No suspicion of suppression of results. We do not downrate this domain.
9. Confidence interval encompasses two ranges of effect (small but important benefit to large benefit. The optimal information size was not met (a total of 11 events) and inadequate sample size. We downrate this domain by -1.5.
10. Confidence interval encompasses one range of effect (small but important benefit to little to no difference). The optimal information size not met and inadequate sample size (<400 participants for a continuous outcome). We downrate this domain by -1.0.

| Summary of findings: | | | | | | |
| --- | --- | --- | --- | --- | --- | --- |
| **E-cigarettes with nicotine compared to other interventions (Quit advice) in the general/mixed population of smokers** | | | | | | |
| **Patient or population**: Current smokers with no plans to quit  **Setting**: Academic medical centres (Penn State Hershey and Virginia Commonwealth University  **Intervention**: Electronic cigarettes with nicotine  **Comparison**: Electronic cigarettes without nicotine | | | | | | |
| Outcomes | **Anticipated absolute effects^*^** (95% CI) | | Relative effect (95% CI) | № of participants  (studies) | Certainty of the evidence (GRADE) | Comments |
|  | **Risk with e-cigarettes without nicotine** | **Risk with e-cigarettes with nicotine** |  |  |  |  |
| Tobacco use abstinence: 7-day point prevalence abstinence^a,b,c^  Follow up: 6 months | 8 per 1000 | 100 more per 1000  (7 more to 799 more) | **RR 14**  (1.9 to 104.9) | 520  (1 RCT) | ⨁◯◯◯  VERY LOW^d,e,f,g,h^ |  |
| Tobacco use abstinence: 28+ days abstinence ^a,b,c^  Follow up: 12 months | 8 per 1000 | 77 more per 1000  (3 more to 638 more) | **RR 11**  (1.4 to 84) | 520  (1 RCT) | ⨁◯◯◯  VERY LOW^d,e,f,i,h^ |  |
| Tobacco use abstinence: Total number of days being totally abstinent ^a,b,c^  Follow up: 12 months | The Mean (SD) number of days in the e-cigs with nicotine group where the participants reported being totally abstinent was 15.6 (36.4) and that in the control group was 4.7 (17). Difference of mean (95% CI) reported between both groups was **10.87 (3.9 to 17.8)**. | | - | 520  (1 RCT) | ⨁⨁◯◯ LOW^d,e,f,j,h^ |  |
| ***The risk in the intervention group** (and its 95% confidence interval) is based on the assumed risk in the comparison group and the **relative effect** of the intervention (and its 95% CI).  **CI:** Confidence interval; **MD:** Mean difference; **RR**: Risk Ratio | | | | | | |
| **GRADE Working Group grades of evidence** **High certainty:** We are very confident that the true effect lies close to that of the estimate of the effect **Moderate certainty:** We are moderately confident in the effect estimate: The true effect is likely to be close to the estimate of the effect, but there is a possibility that it is substantially different **Low certainty:** Our confidence in the effect estimate is limited: The true effect may be substantially different from the estimate of the effect **Very low certainty:** We have very little confidence in the effect estimate: The true effect is likely to be substantially different from the estimate of effect | | | | | | |
|  | | | | | | |

#### Explanations

1. Study participants included had no plans to quit smoking within 6 months.
2. No co-intervention reported.
3. Study participants received an eGo-style ENDS with nicotine liquid (8 or 36 mg/ml) either with tobacco or menthol flavour of their choice in the intervention group and a e-cigarette without nicotine (placebo; 0mg/ml).
4. There were some concerns related to sequence generation and allocation concealment. Therefore, we rate down the domain by -1.0.
5. One study only. We do not downrate this domain.
6. No indirectness, we do not downrate this domain.
7. Confidence interval encompasses two ranges of effect (small but important benefit to large benefit. The optimal information size was not met (a total of 15 events) and inadequate sample size. We downrate this domain by -1.5.
8. Literature search was comprehensive. No suspicion of suppression of results. We do not downrate this domain.
9. Confidence interval encompasses two ranges of effect (small but important benefit to large benefit. The optimal information size was not met (a total of 11 events) and inadequate sample size. We downrate this domain by -1.5.
10. Confidence interval encompasses one range of effect (small but important benefit to little to no difference). The optimal information size not met and inadequate sample size (<400 participants for a continuous outcome). We downrate this domain by -1.0.
