# Supplementary material for: Effectiveness of e-cigarettes as a stop smoking intervention in adults: a systematic review
Source: Syst Rev. 2024 Jun 29;13:168. doi: 10.1186/s13643-024-02572-7 (PMC11218295; doi:10.1186/s13643-024-02572-7)
Supplement: Supplementary file 9 — Additional file 9: Appendix 9: Included analyses. [file 13643_2024_2572_MOESM9_ESM.docx]

# **Appendix I. Included analyses**

|  |  |  |  |  | **GRADE rating** | | | | | |  |
| --- | --- | --- | --- | --- | --- | --- | --- | --- | --- | --- | --- |
| **Study** | **Populations** | **Intervention** | **Comparator** | **Timepoint** | **Abstinence** | **Reduction** | **QoL** | **AE** | **Wt. gain** | **∆ Emot State** | **Results; Number of studies; Number of participants** |
| **Carpenter 2017 & Cravo 2016** | G/M | E-cig with nicotine | No intervention | 12 to 16 weeks |  |  |  | V. LOW^4,5^ |  |  | See GRADE table; n=2; N=1808 |
| **Cravo 2016** | G/M | E-cig with nicotine | No intervention | 12 to 16 weeks |  |  |  |  | V. LOW |  | MD 0.2 kg higher (3.53 lower to 3.93 higher); n=1; N=408 |
| **Adriaens 2014** | G/M | E-cig with nicotine | Waitlist | 1 to 8 weeks |  |  |  | V. LOW ^6^ |  |  | No difference; n=1; N=48 |
| **Holliday 2019** | G/M | E-cig with nicotine + usual care | Usual care | 6 months | V. LOW |  |  |  |  |  | RD 100 more per 1,000 (from 18 fewer to 649 more); n=1; N=80 |
|  | G/M | E-cig with nicotine + usual care | Usual care | 6 months |  | V. LOW ^3,7,8^ |  |  |  |  | CO: MD 6.2 ppm lower (12.21 lower to 0.19 lower); n=1; N=80 |
|  |  |  |  |  |  |  |  |  |  |  | SC: MD 25.1 ng/mL lower (93.48 lower to 43.28 higher); n=1; N=80 |
|  |  |  |  |  |  |  |  |  |  |  | SA: MD 0.6 ng/mL lower (1.54 lower to 0.34 higher); n=1; N=80 |
|  | G/M | E-cig with nicotine + usual care | Usual care | 6 months |  |  | V. LOW |  |  |  | MD 1.4 points higher (5.9 lower to 8.7 higher); n=1; N=80 |
|  | G/M | E-cig with nicotine + usual care | Usual care | 6 months |  |  |  | V. LOW |  |  | See GRADE table; n=1; N=80 |
|  | G/M | E-cig with nicotine + usual care | Usual care | 6 months |  |  |  |  |  | V. LOW | MD 0 points (4.4 lower to 4.4 higher); n=1; N=80 |
| **Dawkins, 2020** | G/M | E-cig with nicotine + fact sheet | Usual care | 24 weeks | V. LOW |  |  |  |  |  | No difference; n=1, N=80 |
|  |  |  |  |  | V. LOW |  |  |  |  |  | No difference; n=1, N=80 |
|  |  |  |  |  | V. LOW |  |  |  |  |  | No difference; n=1, N=80 |
|  | G/M | E-cig with nicotine + fact sheet | Usual care | 24 weeks |  | V. LOW |  |  |  |  | RD 178 more per 1000 (from 103 fewer to 975 more); n=1; N=80 |
|  |  |  |  |  |  | V. LOW |  |  |  |  | RD 50 fewer per 1000 (from 190 fewer to 403 more); n=1; N=80 |
|  | G/M | E-cig with nicotine + fact sheet | Usual care | 24 weeks |  |  | V. LOW |  |  |  | EC group: Mean (SD) score: 0.653(0.36) & Usual group: Mean (SD) score: 0.691 (0.238)); n=1; N=80 |
|  |  |  |  |  |  |  | V. LOW |  |  |  | EC group: Mean (SD) score: 61.8 (21.6) & Usual group: Mean (SD) score: 61 (22.5); n=1; N=80 |
|  | G/M | E-cig with nicotine + fact sheet | Usual care | 24 weeks |  |  |  | V. LOW |  |  | EC group: Mean (SD) score: 5.63 (6.34) & Usual group: Mean (SD) score: 12.70 (4.42); n=1; N=80 |
|  | G/M | E-cig with nicotine + fact sheet | Usual care |  |  |  |  | V. LOW |  |  | EC group: Mean (SD) score: 7.12 (7.22) & Usual group: Mean (SD) score: 10.82 (7.23); n=1; N=80 |
| **Xu, 2023** | G/M | E-cig with nicotine | Quit advice | 6 months | V. LOW |  |  |  |  |  | RD 136 ore per 1000 (from 55 more to 289 more); n=1; N=837 |
|  |  |  |  | 12 months | V. LOW |  |  |  |  |  | RD 111 more per 1000 (from 42 more to 228 more); n=1; N=837 |
|  | G/M | E-cig with nicotine | Quit advice | 6 months |  | V. LOW |  |  |  |  | RD 40 fewer per 1000 (from 47 fewer to 34 fewer); n=1; N=837 |
|  |  |  |  | 12 months |  | V. LOW |  |  |  |  | RD 33 fewer per 1000 (from 53 fewer to 10 fewer); n=1; N=837 |
| **Myers Smith, 2022** | G/M | E-cig with nicotine | NRT (choices included nicotine patch, chewing gum, nasal spray, microtab, inhalator and mouth spray) | 6 months | LOW |  |  |  |  |  | RD 161 more per 1000 (from 15 more to 785 more); n=1; N=135 |
|  |  |  |  |  | LOW |  |  |  |  |  | RD 206 more per 1000 (from 36 more to 600 more); n=1; N=135 |
|  | G/M | E-cig with nicotine | NRT (choices included nicotine patch, chewing gum, nasal spray, microtab, inhalator and mouth spray) | 6 months |  | LOW |  |  |  |  | RD 299 more per 1000 (from 112 more to 560 more); n=1; N=135 |
|  |  |  |  |  |  | LOW |  |  |  |  | RD 203 more per 1000 (from 36 more to 681 more); n=1; N=135 |
|  | G/M | E-cig with nicotine | NRT (choices included nicotine patch, chewing gum, nasal spray, microtab, inhalator and mouth spray) | 6 months |  |  |  | LOW |  |  | See GRADE table; n=1; N=135 |
| **Baldassarri 2018** | MtQ | E-cig with nicotine + standard care | E-cig with no nicotine + standard care | 24 weeks | V. LOW |  |  |  |  |  | RD 100 more per 1,000 (from 59 fewer to 871 more); n=1; N=40 |
|  | MtQ | E-cig with nicotine + standard care | E-cig with no nicotine + standard care | 24 weeks |  | V. LOW ^2^ |  |  |  |  | MD 2.54 higher (4.62 lower to 9.7 higher); n=1; N=40 |
|  | MtQ | E-cig with nicotine + standard care | E-cig with no nicotine + standard care | 24 weeks |  |  |  | V. LOW |  |  | See GRADE tables; n=1; N=40 |
| **Caponnetto 2013 &  Russo 2016** | NMtQ | E-cigarettes with nicotine | E-cigarettes with no nicotine | 24 weeks | V. LOW |  |  |  |  |  | 60 more per 1,000 (from 7 fewer to 232 more); n=1; N=300 |
|  | NMtQ | E-cigarettes with nicotine | E-cigarettes with no nicotine | 52 weeks | V. LOW |  |  |  |  |  | 70 more per 1,000 (from 1 fewer to 270 more); n=1; N=300 |
|  | NMtQ | E-cigarettes with nicotine | E-cigarettes with no nicotine | 24 weeks |  | V. LOW ^1^ |  |  |  |  | 30 more per 1,000 (from 47 fewer to 162 more); n=1; N=300 |
|  | NMtQ | E-cigarettes with nicotine | E-cigarettes with no nicotine | 52 weeks |  | V. LOW ^1^ |  |  |  |  | 25 fewer per 1,000 (from 72 fewer to 68 more); n=1; N=300 |
|  | NMtQ | E-cigarettes with nicotine | E-cigarettes with no nicotine | 52 weeks |  | UTD^3^ |  |  |  |  | See GRADE table; n=1; N=183 |
|  | NMtQ | E-cigarettes with nicotine | E-cigarettes with no nicotine | 52 weeks |  |  |  | UTD |  |  | See GRADE table; n=1; N=NR |
|  | NMtQ | E-cigarettes with nicotine | E-cigarettes with no nicotine | 52 weeks |  |  |  |  | V. LOW |  | See GRADE table; n=1; N=NR |
| **Lucchiari, 2022** | MtQ | E-cigarettes with nicotine + support (psychological counselling) | E-cigarettes with no nicotine (Placebo-control) + support (psychological counselling) | 12 months | V. LOW |  |  |  |  |  | RD 10 fewer per 1000 (from 124 fewer to 204 more); n=1; N=140 |
|  |  | E-cigarettes with nicotine + support (psychological counselling) | E-cigarettes with no nicotine (Placebo-control) + support (psychological counselling) | 12 months |  | LOW |  |  |  |  | Reduction by MD 2.4 (from 4.88 greater reduction to 0.05); n=1; N=140 |
|  | MtQ | E-cigarettes with nicotine + support (psychological counselling) | E-cigarettes with no nicotine (Placebo-control) + support (psychological counselling) | 12 months |  |  |  | V. LOW |  |  | See GRADE tables; n=1; N=140 |
|  |  | E-cigarettes with nicotine + support (psychological counselling) | Support (psychological counselling) | 12 months | V. LOW |  |  |  |  |  | RD 83 more per 1000 (from 45 fewer to 343 more); n=1; N=140 |
|  | MtQ | E-cigarettes with nicotine + support (psychological counselling) | Support (psychological counselling) | 12 months |  | LOW |  |  |  |  | Reduction by MD 2.2 (0.35 to 4.85 |
| **Foulds, 2022** | NMtQ | E-cigarettes with nicotine | Cigarette substitute (plastic tube with no electronics or aerosol) | 24 weeks | V. LOW |  |  |  |  |  | RD 77 more per 1000 (from 6 more to 289 more); n=1; N=260 |
|  |  | E-cigarettes with nicotine | Cigarette substitute (plastic tube with no electronics or aerosol) | 24 weeks | V. LOW |  |  |  |  |  | RD 69 more per 1000 (from 3 more to 358 more); n=1; N=260 |
|  |  | E-cigarettes with nicotine | Cigarette substitute (plastic tube with no electronics or aerosol) | 24 weeks | LOW |  |  |  |  |  | MD 10.29 (from 3.2 greater to 17.4 greater); n=1; N=260 |
|  |  | E-cigarettes with nicotine | Placeno electronic cigarettes | 24 weeks | V. LOW |  |  |  |  |  | RD 100 more per 1000 (from 7 more to 799 more); n=1; N=260 |
|  |  | E-cigarettes with nicotine | Placeno electronic cigarettes | 24 weeks | V. LOW |  |  |  |  |  | RD 77 more per 1000 (from 3 more to 638 more); n=1; N=260 |
|  |  | E-cigarettes with nicotine | Placeno electronic cigarettes | 24 weeks | LOW |  |  |  |  |  | MD 10.87 (from 3.9 greater to 17.8 greater); n=1; N=260 |
| **Carpenter, 2023** | G/M | E-cigarettes with nicotine | No E-cigarettes | 6 months | V. LOW |  |  |  |  |  | RD 55 more per 1000 (from 0 to 147 more); n=1; N=638 |
|  | MtQ | E-cigarettes with nicotine | No E-cigarettes | 6 months | V. LOW |  |  |  |  |  | RD 34 more per 1000 (from 67 fewer to 229 more); n=1; N=174 |
|  |  |  |  |  | V. LOW |  |  |  |  |  | RD 63 more per 1000 (from 4 more to 195 more); n=1; N=464 |
|  | G/M | E-cigarettes with nicotine | No E-cigarettes | 6 months | V. LOW |  |  |  |  |  | RD 44 more per 1000 (from 12 fewer to 132 more); n=1; N=638 |
|  | MtQ | E-cigarettes with nicotine | No E-cigarettes | 6 months | V. LOW |  |  |  |  |  | RD 121 more per 1000 (from 17 fewer to 378 more); n=1; N=174 |
|  |  |  |  |  | V. LOW |  |  |  |  |  | RD 17 more per 1000 (35 fewer to 107 more); n=1; N=464 |
|  | G/M | E-cigarettes with nicotine | No E-cigarettes | 6 months |  | V. LOW |  |  |  |  | RD 97 more per 1000 (from 20 more to 205 more); n=1; N=638 |
|  | MtQ | E-cigarettes with nicotine | No E-cigarettes | 6 months |  | V. LOW |  |  |  |  | RD 119 more per 1000 (from 28 fewer to 362 more); n=1; N=174 |
|  |  |  |  |  |  | V. LOW |  |  |  |  | RD 90 more per 1000 (from 6 more to 218 more); n=1; N=464 |
|  | MtQ | E-cigarettes with nicotine | No E-cigarettes |  |  |  |  | V. LOW |  |  | RD 12 more per 1000 (from 65 fewer to 102 more); n=1; N=638 |
| **Bullen 2013** | MtQ | E-cig with nicotine + behavioural support | E-cig with no nicotine + behavioural support | 6 months | LOW |  |  |  |  |  | 32 more people per 1000 (from 19 fewer to 196 more); n=1, N=657 |
|  | MtQ | E-cig with nicotine + behavioural support | E-cig with no nicotine + behavioural support | 6 months | LOW |  |  |  |  |  | 9 fewer per 1000 (from 90 fewer to 125 more); n=1, N=657 |
| **Lucchiari 2020** | MtQ | E-cig with nicotine + behavioural support | E-cig with no nicotine + behavioural support | 6 months | V. LOW |  |  |  |  |  | 28 more per 1000 (from 69 fewer to 229 more); n=1, N=140 |
| **Bullen 2013** | MtQ | E-cig with nicotine + behavioural support | E-cig with no nicotine + behavioural support | 6 months |  | MOD |  |  |  |  | 118 more per 1000 (from 18 fewer to 298 more); n=1, N=657 |
| **Eisenberg 2020** | MtQ | E-cig with nicotine + behavioural support | E-cig with no nicotine + behavioural support | 24 weeks |  | V. LOW |  |  |  |  | See GRADE table; n=1, N=249 |
| **Lucchiari 2020** | MtQ | E-cig with nicotine + behavioural support | E-cig with no nicotine + behavioural support | 6 months |  | V. LOW |  |  |  |  | MD 3.02 lower (5.42 lower to 0.62 lower); n=1, N=140 |
|  | MtQ | E-cig with nicotine + behavioural support | E-cig with no nicotine + behavioural support | 6 months |  | V. LOW |  |  |  |  | CO: MD 3.27 higher (6.56 lower to 0.02 higher); n=1; N=140 |
| **Eisenberg 2020** | MtQ | E-cig with nicotine + behavioural support | E-cig with no nicotine + behavioural support | 12 weeks |  |  |  | V. LOW |  |  | See GRADE table; n=1; N=249 |
|  | MtQ | E-cig with nicotine + behavioural support | E-cig with no nicotine + behavioural support | 12 to 24 weeks |  |  |  | V. LOW |  |  | See GRADE table; n=1; N=249 |
|  | MtQ | E-cig with nicotine + behavioural support | E-cig with no nicotine + behavioural support | 12 weeks |  |  |  | V. LOW |  |  | See GRADE table; n=1; N=249 |
| **Bullen 2013** | MtQ | E-cig with nicotine + behavioural support | E-cig with no nicotine + behavioural support | 6 months |  |  |  | MOD |  |  | See GRADE table; n=1; N=657 |
|  | MtQ | E-cig with nicotine + behavioural support | E-cig with no nicotine + behavioural support | 6 months |  |  |  | MOD |  |  | See GRADE table; n=1; N=657 |
| **Lucchiari 2020** | MtQ | E-cig with nicotine + behavioural support | E-cig with no nicotine + behavioural support | 3 & 6 months |  |  |  | V. LOW |  |  | See GRADE table; n=1; N=140 |
| **Lucchiari 2020** | MtQ | E-cig with nicotine + behavioural support | No intervention +behavioural support | 6 months | V. LOW |  |  |  |  |  | 86 more per 1000 (from 21 fewer to 338 more); n=1; N=140 |
| **Eisenberg 2020** | MtQ | E-cig with nicotine + behavioural support | No intervention +behavioural support | 24 weeks |  | V. LOW |  |  |  |  | See GRADE table; n=1; N=249 |
| **Lucchiari 2020** | MtQ | E-cig with nicotine + behavioural support | No intervention +behavioural support | 6 months |  | V. LOW |  |  |  |  | MD 2.44 lower (from 4.59 lower to 0.29 lower); n=1; N=140 |
|  | MtQ | E-cig with nicotine + behavioural support | No intervention +behavioural support | 6 months |  | V. LOW |  |  |  |  | CO: MD 4.51 higher (from 1.42 higher to 7.60 higher); n=1; N=140 |
| **Eisenberg 2020** | MtQ | E-cig with nicotine + behavioural support | No intervention +behavioural support | 12 weeks |  |  |  | V. LOW |  |  | See GRADE table; n=1; N=249 |
|  | MtQ | E-cig with nicotine + behavioural support | No intervention +behavioural support | 12 to 24 weeks |  |  |  | V. LOW |  |  | See GRADE table; n=1; N=249 |
|  | MtQ | E-cig with nicotine + behavioural support | No intervention + behavioural support | 12 weeks |  |  |  | V. LOW |  |  | See GRADE table; n=1; N=249 |
| **Walker 2020** | MtQ | E-cig with nicotine + behavioural support + nicotine patch | E-cig with no nicotine + behavioural support + nicotine patch | 6 months | MOD |  |  |  |  |  | 30 more per 1000 (from 1 more to 79 more); n=1; N=999 |
|  | MtQ | E-cig with nicotine + behavioural support + nicotine patch | E-cig with no nicotine + behavioural support + nicotine patch | 6 months | MOD |  |  |  |  |  | 72 more per 1000 (from 23 more to 138 more); n=1; N=999 |
|  | MtQ | E-cig with nicotine + behavioural support + nicotine patch | E-cig with no nicotine + behavioural support + nicotine patch | 6 months | MOD |  |  |  |  |  | 72 more per 1000 (from 18 more to 140 more); n=1; N=999 |
|  | MtQ | E-cig with nicotine + behavioural support + nicotine patch | E-cig with no nicotine + behavioural support + nicotine patch | 6 months |  | MOD |  |  |  |  | MD 0 lower (0.06 lower to 0.06 higher); n=1; N=999 |
|  | MtQ | E-cig with nicotine + behavioural support + nicotine patch | E-cig with no nicotine + behavioural support + nicotine patch | 6 months |  | MOD |  |  |  |  | 60 more per 1000 (from 4 fewer to 132 more); n=1; N=999 |
|  | MtQ | E-cig with nicotine + behavioural support + nicotine patch | E-cig with no nicotine + behavioural support + nicotine patch | 6 months |  |  |  | LOW |  |  | 12 fewer per 1000 (from 27 fewer to 16 more); n=1; N=999 |
|  | MtQ | E-cig with nicotine + behavioural support + nicotine patch | E-cig with no nicotine + behavioural support + nicotine patch | 6 months |  |  |  | MOD |  |  | See GRADE table; n=1; N=999 |
|  | MtQ | E-cig with nicotine + behavioural support + nicotine patch | E-cig with no nicotine + behavioural support + nicotine patch | 6 months |  |  |  |  | MOD |  | MD 0.7 lower (0.76 lower to 0.64 lower); n=1; N=999 |
|  | MtQ | E-cig with nicotine + behavioural support + nicotine patch | E-cig with no nicotine + behavioural support + nicotine patch | 6 months |  |  |  | MOD |  |  | See GRADE table; n=1; N=999 |
|  | MtQ | E-cig with nicotine + behavioural support + nicotine patch | behavioural support + nicotine patch | 6 months | LOW |  |  |  |  |  | 46 more per 1000 (from 2 fewer to 200 more); n=1; N=625 |
|  | MtQ | E-cig with nicotine + behavioural support + nicotine patch | behavioural support + nicotine patch | 6 months | LOW |  |  |  |  |  | 98 more per 1000 (from 15 more to 252 more); n=1; N=625 |
|  | MtQ | E-cig with nicotine + behavioural support + nicotine patch | behavioural support + nicotine patch | 6 months | LOW |  |  |  |  |  | 127 more per 1000 (from 30 more to 288 more); n=1; N=625 |
|  | MtQ | E-cig with nicotine + behavioural support + nicotine patch | behavioural support + nicotine patch | 6 months |  | MOD |  |  |  |  | MD 0.3 lower (0.48 lower to 0.12 lower); n=1; N=625 |
|  | MtQ | E-cig with nicotine + behavioural support + nicotine patch | behavioural support + nicotine patch | 6 months |  | LOW |  |  |  |  | 179 more per 1000 (from 61 more to 340 more); n=1; N=625 |
|  | MtQ | E-cig with nicotine + behavioural support + nicotine patch | behavioural support + nicotine patch | 6 months |  |  |  | LOW |  |  | 8 fewer per 1000 (from 15 fewer to 84 more); n=1; N=625 |
|  | MtQ | E-cig with nicotine + behavioural support + nicotine patch | behavioural support + nicotine patch | 6 months |  |  |  | MOD |  |  | See GRADE table; n=1; N=625 |
|  | MtQ | E-cig with nicotine + behavioural support + nicotine patch | behavioural support + nicotine patch | 6 months |  |  |  |  | MOD |  | MD 0.7 lower (0.88 lower to 0.52 lower); n=1; N=625 |
|  | MtQ | E-cig with nicotine + behavioural support + nicotine patch | behavioural support + nicotine patch | 6 months |  |  |  | MOD |  |  | See GRADE table; n=1; N=625 |
|  | MtQ | E-cig with no nicotine + behavioural support + nicotine patch | behavioural support + nicotine patch | 6 months | LOW |  |  |  |  |  | 16 more per 1000 (from 12 fewer to 109 more); n=1;N=624 |
|  | MtQ | E-cig with no nicotine + behavioural support + nicotine patch | behavioural support + nicotine patch | 6 months | LOW |  |  |  |  |  | 26 more per 1000 (from 24 fewer to 122 more); n=1; N=624 |
|  | MtQ | E-cig with no nicotine + behavioural support + nicotine patch | behavioural support + nicotine patch | 6 months | LOW |  |  |  |  |  | 55 more per 1000 (from 15 fewer to 171 more); n=1; N=624 |
|  | MtQ | E-cig with no nicotine + behavioural support + nicotine patch | behavioural support + nicotine patch | 6 months |  | MOD |  |  |  |  | MD 0.3 lower (0.48 lower to 0.12 lower); n=1; N=624 |
|  | MtQ | E-cig with no nicotine + behavioural support + nicotine patch | behavioural support + nicotine patch | 6 months |  | LOW |  |  |  |  | 125 more per 1000 (from 20 more to 269 more); n=1; N=624 |
|  | MtQ | E-cig with no nicotine + behavioural support + nicotine patch | behavioural support + nicotine patch | 6 months |  |  |  | LOW |  |  | 20 more per 1000 (from 11 fewer to 121 more); n=1; N=624 |
|  | MtQ | E-cig with no nicotine + behavioural support + nicotine patch | behavioural support + nicotine patch | 6 months |  |  |  | MOD |  |  | See GRADE table; n=1; N=624 |
|  | MtQ | E-cig with no nicotine + behavioural support + nicotine patch | behavioural support + nicotine patch | 6 months |  |  |  |  | LOW |  | MD 0 lower (0.18 lower to 0.18 higher); n=1; N=624 |
|  | MtQ | E-cig with no nicotine + behavioural support + nicotine patch | behavioural support + nicotine patch | 6 months |  |  |  | MOD |  |  | See GRADE table; n=1; N=624 |
| **Lucchiari 2020** | MtQ | E-cig with no nicotine + behavioural support | behavioural support | 6 months | V. LOW |  |  |  |  |  | 57 more per 1000 (from 35 fewer to 282 more); n=1; N=140 |
| **Eisenberg 2020** | MtQ | E-cig with no nicotine + behavioural support | behavioural support | 6 months |  | V. LOW |  |  |  |  | See GRADE table; n=1; N=249 |
| **Lucchiari 2020** | MtQ | E-cig with no nicotine + behavioural support | behavioural support | 6 months |  | V. LOW |  |  |  |  | MD 0.58 higher (1.82 lower to 2.98 higher); n=1; N=140 |
|  | MtQ | E-cig with no nicotine + behavioural support | behavioural support | 6 months |  | V. LOW |  |  |  |  | CO: MD 1.24 lower (2.38 lower to 4.86 higher); n=1; N=140 |
| **Eisenberg 2020** | MtQ | E-cig with no nicotine + behavioural support | behavioural support | 12 weeks |  |  |  | V. LOW |  |  | See GRADE table; n=1; N=249 |
|  | MtQ | E-cig with no nicotine + behavioural support | behavioural support | 12 to 24 weeks |  |  |  | V. LOW |  |  | See GRADE table; n=1; N=249 |
|  | MtQ | E-cig with no nicotine + behavioural support | behavioural support | 12 weeks |  |  |  | V. LOW |  |  | See GRADE table; n=1; N=249 |

Abbreviations: AE=adverse event; E-cig=Electronic cigarette; Emot=Emotional; G/M=General/mixed population of smokers; MOD=Moderate; MtQ=Motivated to quit; NMtQ=Not motivated to quit; UTD=Unable to determine; Wt.=Weight; V. Low-Very low; MOD=Moderate

1. Reduction in cigarettes/day of >50% of baseline or cessation
2. Reduction in number cigarettes/day
3. Reduction in CO
4. Adverse events (general)
5. Serious adverse events
6. Complaints
7. Salivary cotinine (SC)
8. Salivary anabasine (SA)
