# Supplementary material for: Effectiveness of e-cigarettes as a stop smoking intervention in adults: a systematic review
Source: Syst Rev. 2024 Jun 29;13:168. doi: 10.1186/s13643-024-02572-7 (PMC11218295; doi:10.1186/s13643-024-02572-7)
Supplement: Supplementary file 10 — Additional file 10: Appendix 10. Stakeholder feedback. [file 13643_2024_2572_MOESM10_ESM.docx]

## **Appendix J**: **Stakeholder feedback**

**Document sent to stakeholders**

Thank you again for reviewing the evidence review manuscript: **Effectiveness of e-cigarettes as a stop smoking intervention in adults: a systematic review.** This systematic review focusses on the benefits and harms of electronic cigarettes as a smoking cessation aid in adults aged 18 years and older. These results will then be used to help inform guidelines for the Canadian Task Force on Preventive Health Care’s (CTFPHC).

**INSTRUCTIONS:**

- Please use this form to provide feedback on the evidence review and return this form no later than **January 15, 2024,** to **taskforce.admin@CTFPHC.onmicrosoft.com**
- If you have any questions related to the review process, contact Greg Traversy at [**gregory.traversy@phac-aspc.gc.ca**](mailto:gregory.traversy@phac-aspc.gc.ca)
- Please check the appropriate box to answer the questions and elaborate in the space provided if necessary.

| **Question 1** | **Yes** | **No** |
| --- | --- | --- |
| Are the objectives and methods of this evidence review clear? | **​​☐​** | **​​☐​** |
|  | **Comments:** | |
| **Question 2** | **Yes** | **No** |
| Were the results clearly stated? | **​​☐​** | **​​☐​** |
|  | **Comments:** | |
| **Question 3** | **Yes** | **No** |
| Are the conclusions in the review supported by the data that were reviewed? | **​​☐​** | **​​☐​** |
|  | **Comments:** | |
| **Question 4** |  | |
| Do you have any additional comments? |  | |

**Stakeholder feedback**

| **Question** | **Comment** | **Response** | **Reviewer** |
| --- | --- | --- | --- |
| **Question 1**  Are the objectives and methods of these evidence reviews clear? | **Abstract/** Idem as the part I (see comments on overview of systematic reviews)  **Introduction /** To minimize redundancy with part I (that already presents prevalence and burden of tobacco smoking), introducing the paper with information on e-cigarette products and consumption might be interesting: range of innovations (regarding devices, products, liquids), utilization and by whom, for what purposes (youths – “new smokers that use vape to consume nicotine” vs adults “traditional (cigarette) smokers that use vape to stop smoking”) …  Some suggestions and minor comments are included directly in the manuscript.  **Methods /** Some details could be added: exclusion of heated tobacco, specification on populations included (e.g., pregnant women), number of selected articles according to source (databases vs others). | Thank you for your comments, we have considered your notes in the introduction. | Reviewer #1 |
| **Question 3**  Are the conclusions in the reviews supported by the data that were reviewed? | Recent reviews and meta-analyses draw conclusions that differ from yours, notably in terms of strength and level of certainty of evidence. The number of included studies (mainly due to publication dates and discrepancies between authors’ judgment on certainty of evidence for some studies) may explain the difference (?) | Thank you for this note, we acknowledge that GRADE is a subjective tool used to help inform judgements on the certainty of evidence and can be the source of observed differences. | Reviewer #1 |
| **Question 4**  Do you have any additional comments? | Another important concern for healthcare providers (notably first-line) about e-cigarettes is how to manage vaping cessation. With the growing popularity of this type of device and the variety of products (salt, liquid), in addition to other novelty (like the nicotine pouch) now available on the market, clinical guidance on nicotine dependence is a more and more pressing issue to address. | Thank you for this note. We have not considered nicotine dependence as our outcome of interest and is outside the scope of this review. | Reviewer #1 |
| **General comments on the systematic review of e-cig** | Good and rigorous work. I do not have a lot of comments on this part unless recent systematic reviews and meta-analyses considering evidence up to 2022-2023 are now available. Given that this work reviewed scientific evidence up to 2020 (almost all resulting in low certainty of evidence), how will the working group manage this issue in order to provide up-to-date clinical guidance on the use of e-cigarettes for smoking cessation? | Thank you for your comment. We have updated our search strategy to include more relevant evidence. | Reviewer #1 |
| **Question 1**  Are the objectives and methods of these evidence reviews clear? | Objectives and Methods are very clear. One methodological limitation sticks out as potentially concerning in the e-cig review: “Additionally, we were unable to retrieve a full text publication for 45 records. However, the likelihood of any of these being of interest is low, as we searched the bibliographies of 12 relevant systematic reviews published from 2016 to 2019, none of which included any of these studies” It assumes the lack of bias and the comprehensiveness of previous SRs, rather than imagining previous SRs facing similar limitations with regards to access, as the current one. Perhaps more description of these 45 records could help to assuage the concern. | Thank you for this note. We have reworded the statement in the discussion and have added details on the list of excluded studies which can be found in Appendix D. | Reviewer #2 |
| **Question 3**  Are the conclusions in the reviews supported by the data that were reviewed? | Yes. The conclusions are well supported, and the strength of the findings is carefully described. | Thank you for your comment. | Reviewer #2 |
| **Question 4**  Do you have any additional comments? | IT was a pleasure to review these reviews, and I look forward to their publication, as I can see much potential for practical use in my and my colleagues’ work. Thank you for the opportunity. | Thank you for your comment. | Reviewer #2 |
| **Question 1**  Are the objectives and methods of these evidence reviews clear? | The methods used are consistent with current procedures employed in reviews and the objectives of both papers are clearly stated. However, the reader who is aware of other published work in this field (e.g. Hartmann-Boyce et al., 2022; Lindson et al., 2023) may wonder why the reviews refer to the 2016-2020 period, therefore excluding recent research. | Thank you for your comment. We have updated our search strategy to include more relevant evidence. | Reviewer #3 |
| **Question 2**  Were the results clearly stated? | There are some discrepancies in the way results are reported. It would be preferable to use the same exact manner for all sections of the reviews. | Thank you for your comment, we have reviewed the formatting. | Reviewer #3 |
| **Question 3**  Are the conclusions in the reviews supported by the data that were reviewed? | The discussion and conclusion sections could benefit from the addition of recommendations based on the findings of the reviews. What does it mean concretely from a clinical standpoint to have low to very low evidence certainty, and to what extent and purposes can these observations be applied to current smoking cessation practices? | Thank you for this note, we have clarified in the discussion section. | Reviewer #3 |
| **Question 4**  Do you have any additional comments? | In the Background section of both reviews, it would be preferable to refer to CCHS data instead of CTNS. It is widely acknowledged that CCHS provides more valid estimates of tobacco use prevalence in Canada. | Thank you for this note. Since the CTNS data is the recent one (i.e., 2022), we preferred using it. | Reviewer #3 |
| **Question 1**  Are the objectives and methods of these evidence reviews clear? | The objective is clear. The methods do not meet the objective.  See accompanying note | Thank you for this note. We have applied the *a priori* PICO criteria to determine our included studies (i.e., RCTs for benefits and RCTs/observational studies for harms). Including results from other study designs that do not meet our inclusion criteria is outside the scope of this review. We have also excluded results from studies assessing comparative effectiveness as that lies outside the scope of our review. | Reviewer #4 and #5 |
| **Question 3**  Are the conclusions in the reviews supported by the data that were reviewed? | The analysis mistook some harms as benefits.  The review added little to the body of available knowledge.  The conclusions are more vague than those supported by evidence  Because of flaws in the methods, the review inappropriately concluded that e-cigarettes “probably” increase smoking cessation.  See accompanying note. | Thank you for this note. Based on the categorization mentioned in the eligibility criteria, smoking abstinence or reduced smoking was considered a benefit; however, uncertainties surrounding dual intervention use are being flagged in the guideline and as one of the limitations in the manuscript. | Reviewer #4 and #5 |
| **Question 4**  Do you have any additional comments? | The study did not consider the impact of e-cigarettes on relapse.  There was little transparency to allow an assessment of tobacco-industry interference  The study did not consider the risks of continuing nicotine addiction. | Thank you for this note. We have not considered nicotine dependence or relapse as our outcome of interest and is outside the scope of this review. For individual studies, we have considered industry funding during our risk of bias assessment. Any funding received for this review is reported under “Funding” at the end of the manuscript. | Reviewer #4 and #5 |
| **Question 1**  Are the objectives and methods of these evidence reviews clear? | Yes, very clear as stated on the report. | Thank you for your comment. | Reviewer #6 |
| **Question 2**  Were the results clearly stated? | Yes, very clear as stated on the report. | Thank you for your comment. | Reviewer #6 |
| **Question 3**  Are the conclusions in the reviews supported by the data that were reviewed? | Yes, this review’s conclusions are supported by the data that were reviewed. | Thank you for your comment. | Reviewer #6 |
| **Question 4**  Do you have any additional comments? | I am not an expert in assessing evidence reviews, but they seemed clear from my perspective. I understand the need to limit the smoking cessation intervention review to the general population and those with mental health issues, because of the vast scope of the studies available. However, since so many people who smoke have co-morbidities (cancer, heart disease, diabetes), I think it will be important to consider and articulate how the evidence for the general population could be extended/generalized to people with other diseases when making the recommendations. | Thank you, this is an important note to consider for future research. | Reviewer #7 |
| **Question 3**  Are the conclusions in the reviews supported by the data that were reviewed? | I agree with the review’s “Conclusion” that the need for more research to understand the long-term benefits and potential harms of e-cigarettes is a critical evidence gap with respect to the use of e-cigarettes as a smoking cessation intervention. | Thank you for your comment. | Reviewer #8 |
| **Question 4**  Do you have any additional comments? | I think there’s an underlying definitional (or even foundational) question that the review might address, even if it remains unanswered – is switching from combustible tobacco products to ENDS smoking cessation? Or is the goal cessation of all smoking; or should that be the goal? | Thank you for your comment. Based on the defined *a priori* PICO criteria of our review, our outcome of interest is tobacco use abstinence using e-cigs as a smoking cessation aid and not the cessation of all smoking.  this note. | Reviewer #8 |
| **Question 1**  Are the objectives and methods of these evidence reviews clear? | Methods were notably clear and having the protocol registered with PROSPERO is a strength. | Thank you for your comment. | Reviewer #9 |
| **Question 2**  Were the results clearly stated? | Appreciated the consistency with which each set of results was presented. Would be helpful to have the forest plots available as a figure. | Thank you for your comment. Due to clinical and methodological heterogeneity (i.e., different types of e-cigarettes, their doses and combinations, duration of interventions, varied outcome reporting), we were unable to perform meta-analyses, subgroup analysis, sensitivity analysis, and evaluate small study effects. | Reviewer #9 |
| **Question 3**  Are the conclusions in the reviews supported by the data that were reviewed? | Agree with the strengths discussed. The discussion of limitations was thorough but neglected to mention a phenomenon I’ve observed in clinical practice where patients actually end up increasing their overall consumption of nicotine when switching to a vape due to easier access and less restrictions on use. This is a possible direction for future research, as I doubt it is currently reported in the literature | Thank you for this note. The comment has been added as a limitation in the discussion part of our manuscript. | Reviewer #9 |
| **Question 4**  Do you have any additional comments? | Overall, a well-done systematic review. | Thank you for your comment. | Reviewer #9 |
| **Question 1**  Are the objectives and methods of these evidence reviews clear? | The objectives of this systematic review were clearly articulated, outlining the questions that the paper seeks to assess.  The systemic paper has done a good job in explaining the methods.  However, it lacked a detailed discussion on the randomization process of the included studies, including whether participants were aware of their treatment group and how this awareness might have influenced the study’s outcome. In addition, what had been done to control/mitigate potential confounders in those studies. Finally, the paper did not discuss whether the researchers had control over assigning treatment to participants in those studies. It was not very clear if those studies followed the rules/principles of RCT. | Thank you for your comment. Details on the RoB analysis can be found in the manuscript and Appendix G. | Reviewer #10 |
| **Question 2**  Were the results clearly stated? | Results of the systematic review were clearly stated and discussed.  However, some studies provided behavioural support for both group, and this might make it challenging to solely attribute the outcomes of those studies to e-cigarette. | Thank you for your comment. | Reviewer #10 |
| **Question 3**  Are the conclusions in the reviews supported by the data that were reviewed? | Conclusion was supported by the results. Researchers’ acknowledgement of the short duration of the studies was crucial, as it help readers/policy makers to take the result of this review with caution. From the conclusion part: “Consequently, the lack of evidence on long-term benefits (cessation) and potential harms of e-cigarette use in this review suggests an evidence gap that further necessitates more research”. | Thank you for your comment. | Reviewer #10 |
| **Question 4**  Do you have any additional comments? | Even though it is not the objective of this review, it would have been important to mention already established effects of e-cigarette, such as Vaping-associated lung injuries (VALI), which occurred in Canada and the United States in recent years so that reader will understand the potential negative effects for non-smoker considering vaping. | Thank you, this is an important point to consider for future research and reviews. | Reviewer #10 |
| **Question 4**  Do you have any additional comments? | The concern I have always had is a lack of standardized products. The General population cannot be guaranteed that the dosage as purchased is the correct dosage. No quality control or regulation. | Thank you, this is an important point to consider. | Reviewer #11 |
| **Question 1**  Are the objectives and methods of these evidence reviews clear? | Overall, the description of methods was clear. However, it is not clearly explained why the subgroup of populations with comorbidities were excluded. This is a potentially clinically relevant population so it would be useful to understand why any studies evaluating the effectiveness of e-cigarettes would be removed. | Thank you, this is an important note to consider for future research. | Reviewer #12 |
| **Question 4**  Do you have any additional comments? | The search strategy date is quite dated, and I think this reduces the potential relevance of its conclusions. In the absence of updating the search strategy, it would be important to provide a more detailed comparison of this review and the 2022 published review in the discussion section. | Thank you for your comment. We have updated our search strategy to include more relevant evidence. | Reviewer #12 |
| **Question 2**  Were the results clearly stated? | Very. | Thank you for the comment. | Reviewer #13 |
| **Question 1**  Are the objectives and methods of these evidence reviews clear? | Clear methods and ratings, appreciate description of AMSTAR 2, Grades of Evidence and reasons for exclusion. | Thank you for the comment. | Reviewer #14 |
| **Question 4**  Do you have any additional comments? | I am curious why you chose to limit data to September 24^th^, 2020?  Some minor flaws in how international guidelines are sourced  I note you chose language of no conflicts vs authors do not report/or disclose conflict of interest | Thank you for your comment. We currently have updated our search strategy and are in the midst of screening process. | Reviewer #14 |
| **Question 1**  Are the objectives and methods of these evidence reviews clear? | The review objective is “benefits and harms” of e-cigs. The benefits and harms are not defined. From review of the ‘results section’ benefits and harms are poorly defined. The abstract needs greater specificity | Thank you for your comment. We have revised the categorization of benefits and harms in Appendix C to clarify. | Reviewer #15 |
| **Question 3**  Are the conclusions in the reviews supported by the data that were reviewed? | low to moderate evidence for benefit/harm – the conclusion should be perhaps “studies do not provide any evidence to make any claims of benefit/harm of vapes and ability for systemic reviews of vaping is limited. | Thank you, this is an important point to consider. | Reviewer #15 |
| **Question 4**  Do you have any additional comments? | no nicotine vapes are registered smoking cessation products – the paper needs to be far more critical with the language around claims for vapes benefits/harms as cessation products; more critical analysis of studies making claims about nicotine vapes. | Thank you, this is an important point to consider. | Reviewer #15 |
